# Dual Nickel/Photoredox-Catalyzed Asymmetric Carbosulfonylation of Alkenes

**DOI:** 10.1021/jacs.3c00744

**Published:** 2023-05-30

**Authors:** Xiaoyong Du, Iván Cheng-Sánchez, Cristina Nevado

**Affiliations:** Department of Chemistry, University of Zurich, Winterthurerstrasse 190, Zurich, CH 8057, Switzerland

## Abstract

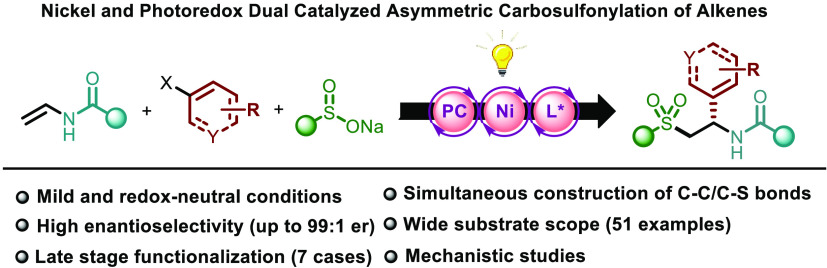

An asymmetric three-component carbosulfonylation of alkenes is presented here. The reaction, involving the simultaneous formation of a C–C and a C–S bond across the π-system, uses a dual nickel/photoredox catalytic system to produce both β-aryl and β-alkenyl sulfones in high yields and with excellent levels of stereocontrol (up to 99:1 er). This protocol exhibits a broad substrate scope and excellent functional group tolerance and its synthetic potential has been demonstrated by successful applications toward pharmacologically relevant molecules. A broad array of control experiments supports the involvement of a secondary alkyl radical intermediate generated through radical addition of a sulfonyl radical to the double bond. Moreover, stoichiometric and cross-over experiments further suggest an underlying Ni(0)/Ni(I)/Ni(III) pathway operative in these transformations.

## Introduction

**Figure 1:**
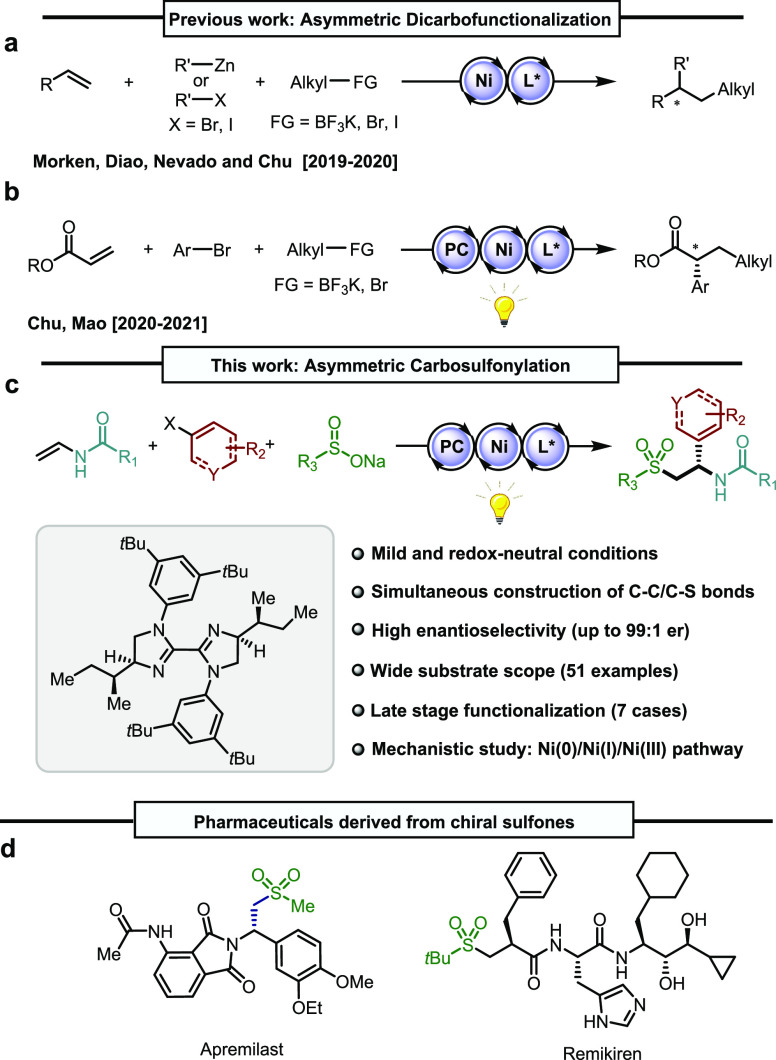
Asymmetric three-component dicarbofunctionalization of olefins (a) via nickel catalysis and (b) via nickel and photoredox dual catalysis. (c) This work: nickel and photoredox dual catalyzed asymmetric carbosulfonylation of olefins. (d) Biologically relevant pharmaceuticals containing chiral sulfones.

As abundant and readily available feedstocks, olefins have been recently extolled as prominent platforms for the formation of C–C and C–X bonds. Intermolecular difunctionalizations of alkenes represent a powerful and versatile entry to molecular complexity as they enable the stepwise or simultaneous formation of multiple bonds and σ-bonds across the π-system, with the potential to attain high levels of both regio- and stereocontrol.^1^ Processes involving two-electron pathways typically rely on noble transition-metal catalysts, which operate with sensitive organometallic reagents under oftentimes harsh conditions. These limitations have fostered the development of strategies involving radical species, which have gained significant traction in the past years.^2^ In this context, nickel-catalyzed processes have witnessed a meteoric growth owing to the ability of this metal to undergo oxidative addition and prevent β-hydride elimination.^3^ Thus, numerous Ni-catalyzed intermolecular difunctionalizations of alkenes have been developed in recent years.^4^ However, asymmetric variants have only recently started to emerge and are still scarce.^5,6^ In 2019, the Morken group reported a seminal example of an enantioselective nickel-catalyzed intermolecular dicarbofunctionalization of vinyl boronic esters combining organozinc reagents and alkyl iodides.^7^ Subsequent transformations have also been achieved via reductive, nickel-catalyzed, enantioselective cross-electrophile couplings. Works from Diao et al.,^8^ Chu et al.,^9^ and our own group^10^ have showcased that styrenes, vinyl amides, and allylic esters, respectively, are suitable partners in these transformations (Figure 1a). Further, dual photoredox/nickel catalytic approaches have also bore fruit showcasing alkyl trifluoroborates and alkyl bromides as radical precursors as nicely demonstrated by the Chu^11^ and Mao^12^ groups among others (Figure 1b). Interestingly, all the abovementioned methods describe dicarbofunctionalization processes. In sharp contrast, examples of the simultaneous formation of C–C and C–X bonds across the π-system are much less abundant,^4a,4s,13^ and more importantly, enantioselective versions are yet to be reported. Early studies from our own^4s^ and the Rueping^14^ group have shown the potential application of nickel catalysis in the arylsulfonylation of olefins and dienes.

Here, a dual nickel/photoredox catalytic system has been unraveled enabling the simultaneous formation a C–C and a C–S bond across the π-system with excellent levels of both regio- and absolute stereocontrol (Figure 1c). The reaction proceeds under mild conditions with a broad substrate scope. This redox-neutral asymmetric carbosulfonylation of alkenes represents a *de novo* entry to enantioenriched sulfones,^15^ which are considered privileged motifs in natural products and pharmaceuticals, as illustrated by FDA-approved drugs such as PDE4 inhibitor Apremilast^16^ and the renin inhibitor Remikiren (Figure 1d).^17^

## Results and Discussion

*N*-Vinylbenzamide, 4-iodoanisole, and sodium benzenesulfinate were chosen as model substrates to identify the optimal reaction conditions (Table 1).^18^ The reaction occurred smoothly in the presence of chiral biimidazoline (BiIM)-nickel dibromide complex (**L1**NiBr_2_)^6e,19^ with 4-CzIPN as an organic photocatalyst under light irradiation in DMSO at 0 °C, giving sulfone **1** in 79% yield in almost racemic form (58:42 er, Table 1, entry 1). In sharp contrast, no reaction was observed in other commonly used solvents such as CH_3_CN or THF, which we ascribed to the lack of solubility of sodium benzenesulfinate in those media (Table 1, entries 2–3). To overcome this problem, we explored the use of crown ethers aiming to sequester the corresponding Na^+^ cations and thereby increase the solubility of the S-donor partner.

**Table 1:**
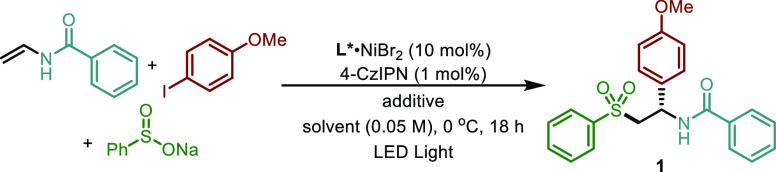

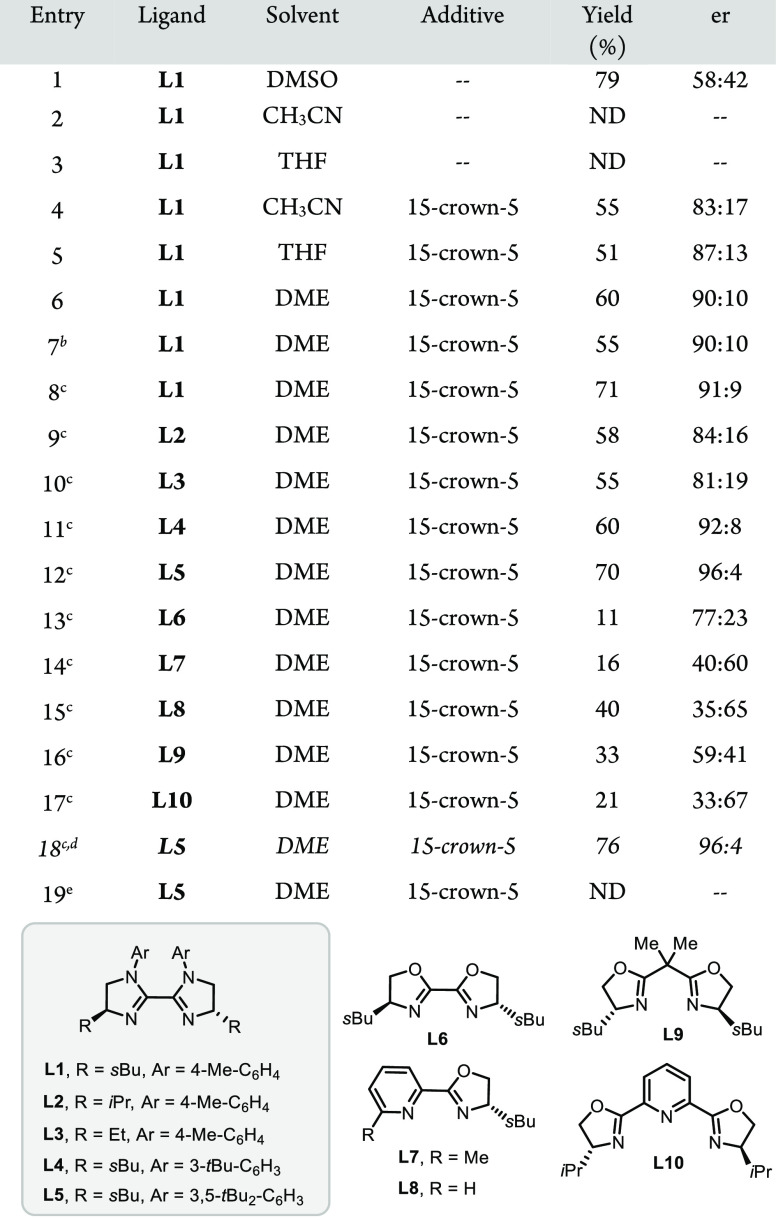
Optimization of the Reaction Conditions^a^

a Reactions were carried out with vinyl amide (0.2 mmol), aryl iodide (0.1 mmol), PhSO_2_Na (0.2 mmol), L·NiBr_2_ (10 mol %), 4-CzIPN (1 mol %), additive (0.9 mmol), solvent [0.05 M], 34 W blue LED, 0 °C, 18 h. Isolated yields after column chromatography. Enantiomeric ratios (er) were determined by HPLC with a chiral stationary phase.

b –20 °C.

c DME [0.025 M].

d Vinyl amide/aryl iodide/PhSO_2_Na = 2/1/2, 390 nm 45 W Kessil LED, 48 h.

e No nickel, no PC, or no light. DME: dimethoxyethane. ND: not detected.

Various solvents were then tested in the presence of 15-crown-5 with DME furnishing compound **1** in 60% yield and 90:10 er (Table 1, entries 4–6). Lowering the reaction temperature to −20 °C or decreasing the reaction concentration by two-fold had no beneficial effect on the enantioselectivity (Table 1, entries 7–8). Screening of different chiral ligands showed that BiIM templates (**L1-L5**) were generally more effective than those based on oxazoline motifs (**L6-L10**; Table 1, entries 9–17) with the sterically demanding *N*-3,5-di-*t*BuC_6_H_3_-*s*BuBiIM ligand (**L5**) offering the best output both in terms of yield (70%) and stereocontrol (96:4 er) (Table 1, entry 12). Finally, adjusting the reaction stoichiometry to two equivalents of both alkene and sulfinate and 1 equivalent of iodoarene as well as the irradiation conditions (from 456 to 390 nm for 48 h) slightly improved the reaction outcome furnishing **1** in 76% yield with 96:4 er (Table 1, entry 18). Control experiments further confirmed that the nickel catalyst, the photocatalyst, and light were all essential for a successful outcome (Table 1, entry 19).

With the optimized reaction conditions in hand, the scope of aryl halides was investigated next (Table 2a). A variety of aryl iodides bearing both electron-donating as well as electron-withdrawing groups in the *para* and *meta* positions were amenable to the reaction protocol, furnishing the difunctionalized products **1–16** in moderate to good isolated yields (50 to 81%) and excellent enantioselectivities (94:6 to 97:3 er). Functional groups including halides, cyanides, ketones, esters, aldehydes, boronic esters, and pyrroles were compatible with the redox-neutral reaction conditions. Notably, the method also worked efficiently for more challenging *ortho-*substituted aryl iodides, as demonstrated by the reactions producing compounds **17** (*o*-OMe) and **18** (*o*-F), which proceeded in 63 and 58% isolated yields and 95:5 er, respectively. Iodobenzene, β-iodonaphthalene, and 5-iodo-1-indanone were also suitable substrates, furnishing the corresponding carbosulfonylated products **19**–**21** in good yields with excellent levels of stereocontrol. Further, 4-bromotrifluorotoluene was also subjected to the standard reaction conditions. To our delight, the corresponding arylsulfonylated product **7** could be obtained in comparable yield and er to those observed with the iodide, thus highlighting the potential of this transformation to accommodate aryl bromides as efficient reaction partners. Interestingly, heteroaryl halides bearing 1,4-benzodioxane, quinoline, thiophene, pyrimidine, and dibenzo[*b*,*d*]thiophene moieties were successfully applied in this synergistic protocol, affording sulfones **22–26** with high enantioselectivity (up to 96:4 er). It should be noted that our system also works well with alkenyl bromides as enantioenriched β*-*alkenyl sulfones **27–29** could also be isolated in synthetically useful yields and enantiomeric ratios (Table 2b).

**Table 2:**
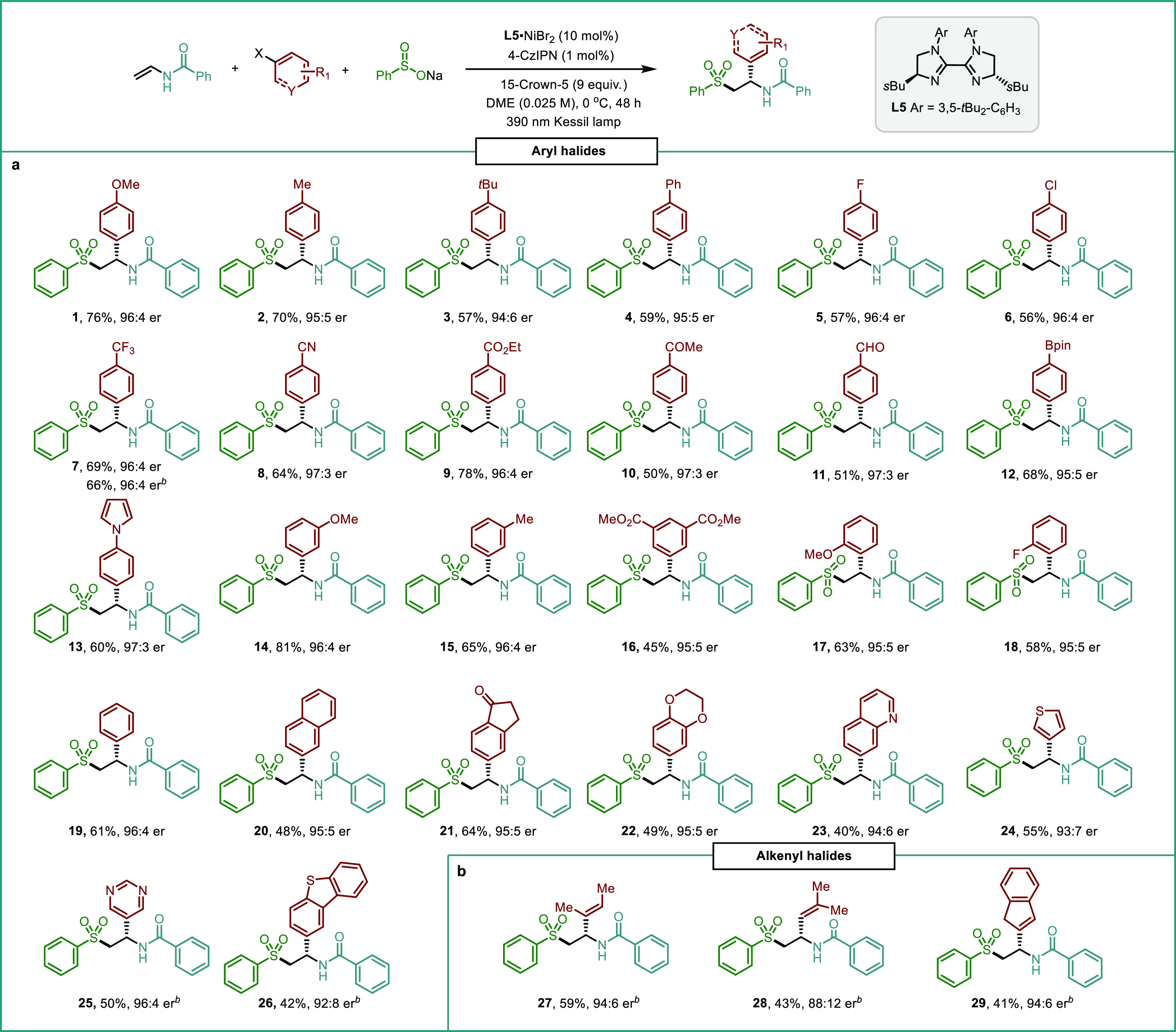
Scope of Aryl and Alkenyl Halides^a^

a Reaction conditions: aryl or alkenyl halide (0.1 mmol), vinyl amide (0.2 mmol), sulfinate precursor (0.2 mmol), **L5**·NiBr_2_ (10 mol %), 4-CzIPN (1 mol %), 15-crown-5 (0.9 mmol), DME [0.025 M], 390 nm 45 W Kessil LED, 0 °C, 48 h. Isolated yields after column chromatography. Enantiomeric ratios (er) were determined by HPLC with a chiral stationary phase.

b The corresponding bromides were used as reaction partners.

Different alkene acceptors and sulfinate precursors were evaluated next. A wide range of vinyl amides delivered the corresponding chiral α-aryl sulfones in moderate to good yields and with excellent enantioselectivities (Table 3). Enamides bearing electron-donating (**30–32**) as well as electron-withdrawing groups (**33–40**) at different positions of the aromatic ring were smoothly difunctionalized to give the desired products in 45–85% yields and 94:6–97:3 er values. In addition, β*-*naphthalene carboxamide and 5-chloro-*N*-vinylnicotinamide were also suitable substrates for this asymmetric transformation delivering compounds **41** and **42** in 62 and 60% yield and 97:3 and 93:7 er, respectively. Notably, alkyl amides were also compatible with the reaction conditions as demonstrated by the isolation of compound **43** in 62% yield (97:3 er). To our delight, vinyl carbamates were also suitable partners in our system, delivering the corresponding adducts **44** and **45** in moderate yield and high enantioselectivities (Table 3b). In contrast, 1,1-and 1,2-disubstituted internal olefins were found to be unreactive under the standard conditions (see Table S8 in the Supporting Information for unsuccessful olefins). We were delighted to see that different arylsulfinates bearing methyl, chloro, and amido groups were compatible with our reaction conditions affording the corresponding chiral α-aryl sulfones **46–48** in good yields and high enantioselectivities (Table 3c, up to 99:1 er). Moreover, alkyl sulfinates could also be incorporated in the reaction protocol. Sodium 3-methoxy-3-oxopropane-1-sulfinate reacted with 4-methoxy-*N*-vinylbenzamide and three different aryl iodides (4-CN, 4-OMe and 3-OMe-benzene) showing the versatility of the reaction. The corresponding arylsulfonylated products **49–51** were successfully obtained in synthetically useful yields with excellent levels of absolute stereocontrol (Table 3c).

**Table 3:**
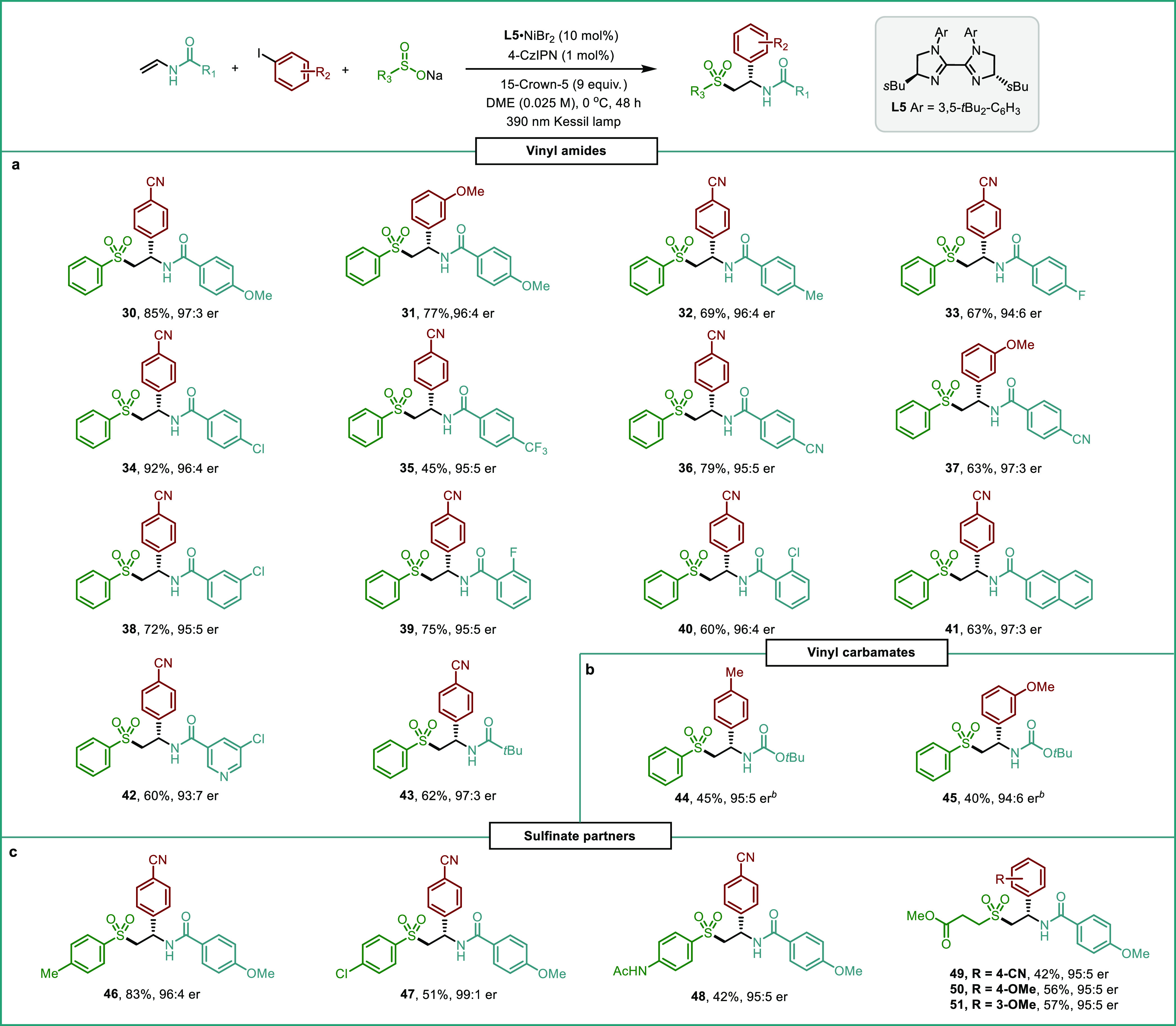
Scope of Alkenes and Sulfinates^a^

a Reaction conditions: aryl iodide (0.1 mmol), alkene (0.2 mmol), sulfinate precursor (0.2 mmol), **L5**·NiBr_2_ (10 mol %), 4-CzIPN (1 mol %), 15-crown-5 (0.9 mmol), DME [0.025 M], 390 nm 45 W Kessil LED, 0 °C, 48 h. Isolated yields after column chromatography. Enantiomeric ratios (er) were determined by HPLC with a chiral stationary phase.

b 20 mol % of **L5**·NiBr_2_.

To demonstrate the synthetic utility of this asymmetric carbosulfonylation of alkenes, we set out to apply this protocol to more structurally complex reaction partners featuring motifs commonly found in natural products and pharmaceutically active molecules (Table 4). Aryl iodides bearing a probenecid motif, an inhibitor of renal excretion of most β-lactam antibiotics,^20^ and an adamantane motif, which is usually introduced into active drugs in order to increase their lipophilicity and improve their pharmacological properties,^21^ were converted to the corresponding products **52** and **53**, respectively, in good yields and excellent enantioselectivities. It should be noted that the absolute configuration of the difunctionalized products was unambiguously confirmed by X-ray diffraction analysis of (*S*)-**52**. More complex substrates bearing additional stereocenters were also compatible with the mild conditions and no loss of existing stereochemical information was observed. Diacetone-d-glucose and l-phenylalanine derivatives were tolerated, furnishing the corresponding chiral sulfones in 62 and 73% yield with 96:4 and 95:5 dr, respectively (**54, 55**). A Boc-β-alanine-Gly dipeptide derivative was prepared following the standard procedure. The difunctionalized product **56** could be isolated in excellent yield (83%) and enantiomeric ratio (96:4). A cholesterol derivative was also applied in the reaction delivering adduct **57** in 43% yield and 96:4 dr. Tocopherol derived iodide reacted with *N*-vinylbenzamide and sodium benzenesulfinate to give the desired product **58** in 40% yield and 96:4 dr.

**Table 4:**
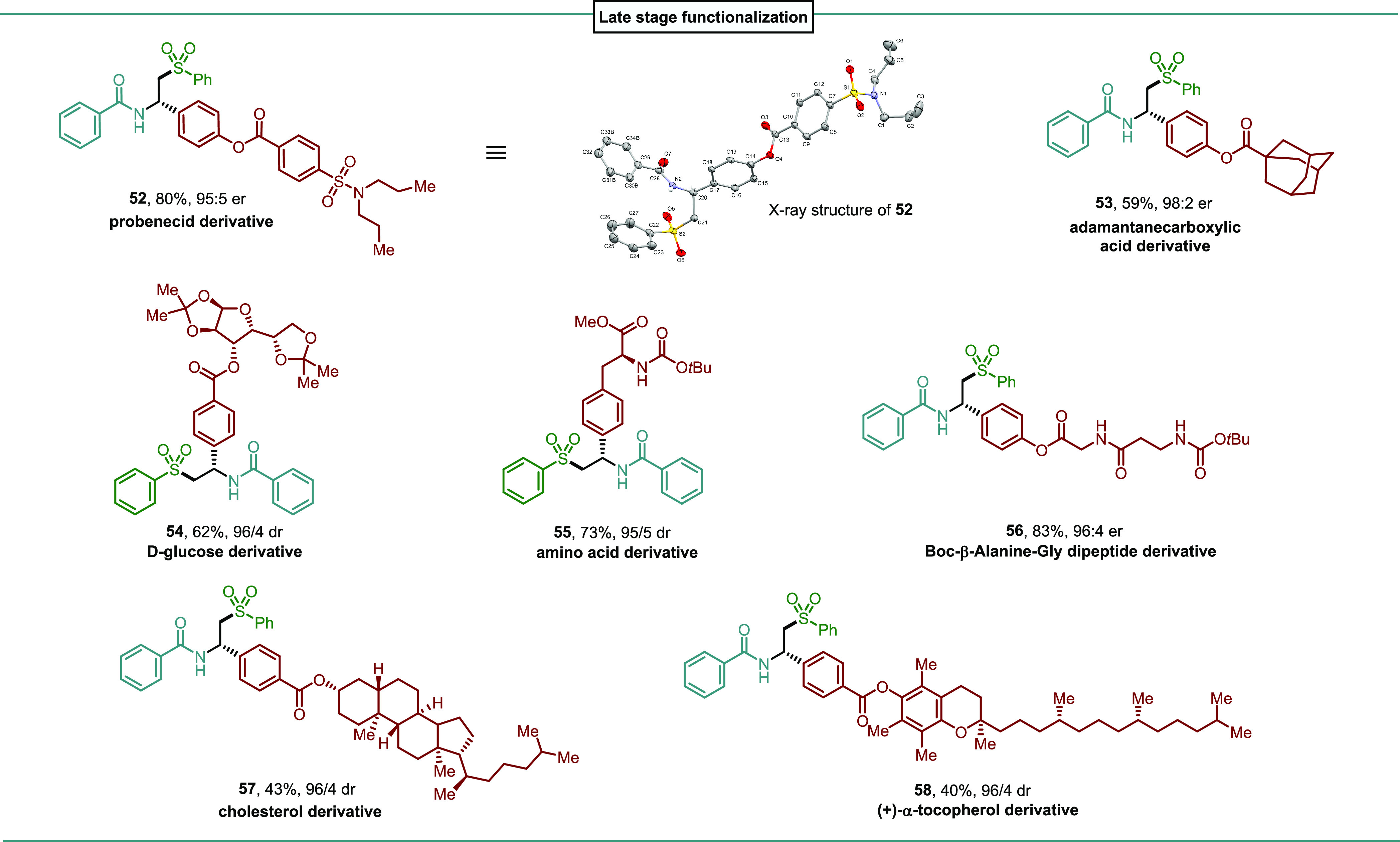
Late-Stage Modification of Complex Molecules^a^

a Reaction conditions: Aryl iodide (0.1 mmol), vinyl amide (0.2 mmol), sulfinate precursor (0.2 mmol), **L5**·NiBr_2_ (10 mol %), 4-CzIPN (1 mol %), 15-crown-5 (0.9 mmol), DME [0.025 M], 390 nm 45 W Kessil LED, 0 °C, 48 h. Isolated yields after column chromatography. Enantiomeric ratios (er) were determined by HPLC with a chiral stationary phase.

**Figure 2:**
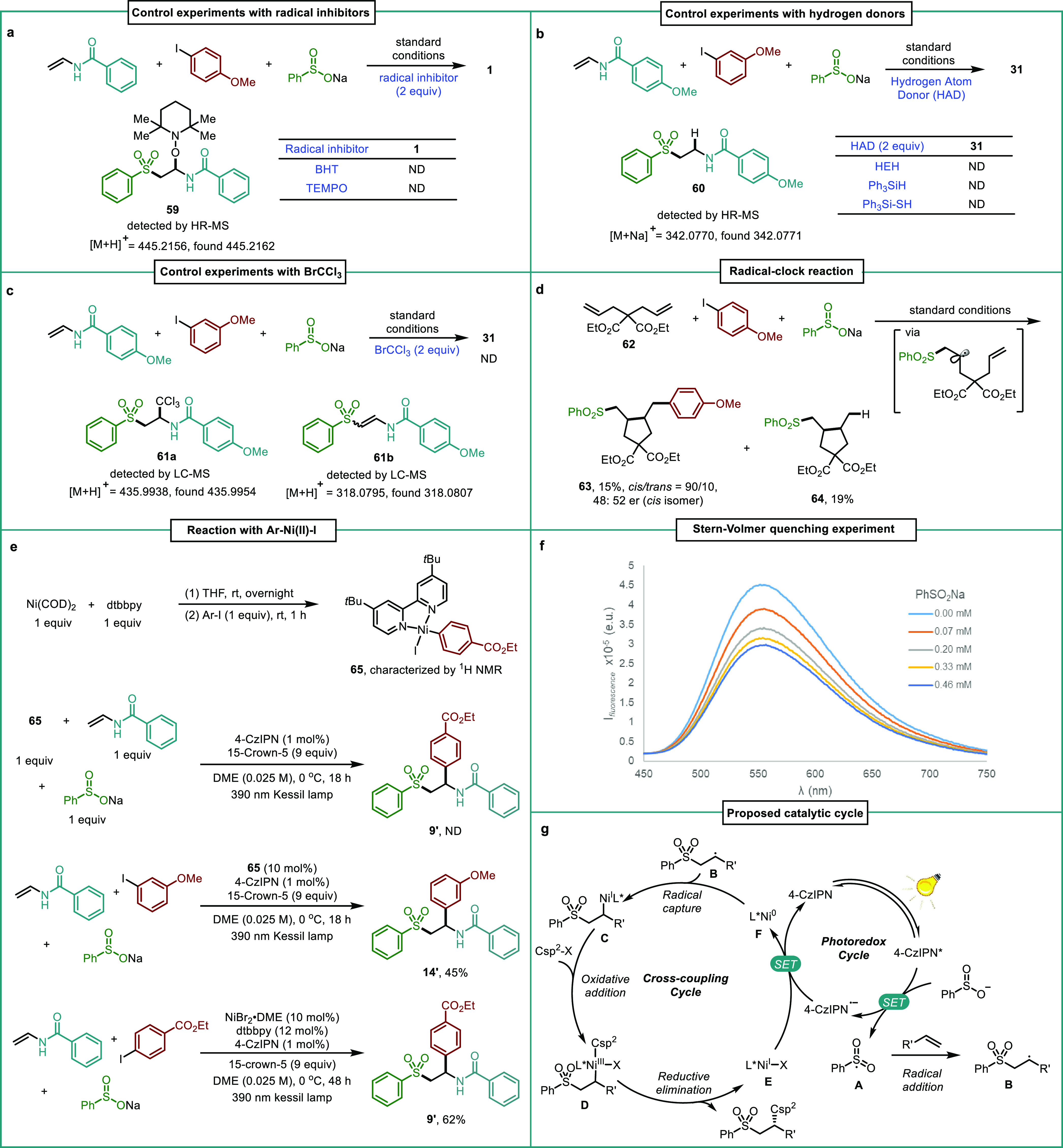
Mechanistic investigations. (a) Control experiments with radical inhibitors. (b) Control experiments with hydrogen donors. (c) Control experiments with other radical precursors. (d) Radical-clock reaction. (e) Reaction with Ar-Ni(II)-I complex **65**. (f) Stern–Volmer quenching experiment. (g) Proposed catalytic cycle. Control experiments (a−c) were conducted under the standard conditions using 10 mol % of the photocatalyst.

To elucidate the mechanism of this dual nickel/photoredox-catalyzed asymmetric carbosulfonylation, additional experiments were conducted, the results of which are summarized in Figure 2.^18^ When two equivalents of a radical scavenger, such as TEMPO or BHT were added, no β-aryl sulfone was formed while TEMPO adduct **59** was observed by HR-MS (Figure 2a). The reaction was also completely inhibited in the presence of several hydrogen atom donors (HAD) such as Hantzsch ester, triphenylsilane, or triphenylsilanethiol. Moreover, the hydrosulfonylation adduct **60** was detected by HR-MS in the presence of two equivalents of the latter HAD, as shown in Figure 2b. When the reaction was carried out in the presence of BrCCl_3_, the corresponding alkyl-CCl_3_ adduct **61a** as well as elimination product **61b** could be detected in the mixture (Figure 2c, see also the SI). These results hint towards the formation of a sulfonyl radical that, upon addition to the olefin, delivers a new carbon-centered radical intermediate that can be intercepted in the presence of the abovementioned additives. A radical-clock experiment was designed involving diene **62**. The reaction with 4-iodoanisole and PhSO_2_Na proceeded with a sequential radical addition/cyclization/aryl coupling process to afford the 5-*exo* cyclized product **63** in 15% yield (*cis/trans* 90:10, 48:52 er) along with non-coupling byproduct **64** (Figure 2d). This *cis/trans* ratio was consistent with the involvement of a radical intermediate in this reaction.^4v,9,14^

To shed light on the nature of the active nickel species in the reaction, several experiments were also carried out. First, Ar-Ni(II)-I complex **65** was prepared and used in a stoichiometric fashion in the reaction of vinyl amide and sodium benzenesulfonate. No conversion to the corresponding difunctionalization product **9′** could be observed in the reaction mixture (Figure 2e), thus ruling out aryl-Ni(II) species as productive intermediates in this trasformation.^14^ In contrast, a cross-over reaction with 3-iodoanisol occurred smoothly in the presence of a catalytic amount of complex **65**, giving **14′** in 45% yield. Further, using the dtbbpy ligand, the corresponding racemic arylsulfonylated product **9′** could be obtained in 62% yield. This result highlights that the lack of reactivity observed for **65** in terms of aryl group transfer is not due to the use of a dtbbpy ligand itself. Overall, these experiments suggest that, if formed, Ar-Ni(II) complex **65** can generate low-valence nickel(I) species that can complete the cycle.^22^ Stern–Volmer experiments were done with vinyl amide, 4-iodoanisole, benzenesulfinic acid sodium salt, and Ni complex (**L5·**NiBr_2_). The results showed that benzenesulfinic acid as well as the nickel complex could quench the photocatalyst, thus supporting the abovementioned notion (Figure 2f and see also the SI).

Based on the mechanistic investigations described above and previous examples in the literature,^4s,14,23^ a plausible mechanism of the nickel/photoredox dual catalyzed asymmetric carbosulfonylation of olefins is proposed. The reaction involves two interconnected catalytic cycles, a photoredox and a cross-coupling cycle (Figure 2g). The photoredox cycle starts by photoexcitation of 4-CzIPN upon irradiation with light to give the excited catalyst, which oxidizes sodium benzenesulfinate by single-electron transfer (SET) to generate the sulfonyl radical **A** (*E*_1/2_ (PhO_2_S^•^/PhSO_2_Na) = −0.37 vs SCE)^24^ and 4-CzIPN^•–^ (*E*_1/2_ (PC*/PC^•–^) = +1.43 vs SCE).^25^

Radical addition of the sulfonyl radical **A** to the C=C bond then produces a secondary alkyl radical **B**, which is rapidly captured by Ni(0) complex **F** to afford (alkyl)Ni(I) species **C**. Subsequent oxidative addition of the aryl iodide to intermediate **C** gives (aryl)(alkyl)Ni(III) intermediate **D**. The high-valence Ni(III) intermediate **D** undergoes facile reductive elimination to furnish the carbosulfonylation product and Ni(I)-X species **E**. Reduction of **E** by 4-CzIPN^•–^ through SET regenerates the ground-state photocatalyst (*E*_1/2_ (PC/PC^•–^) = −1.24 vs SCE)^25^ as well as Ni(0) species **F** (*E*_p_ (Ni(I)/Ni(0)) = −1.17 vs SCE)^19b^ to close both catalytic cycles.

In conclusion, an enantioselective three-component carbosulfonylation reaction of olefins with sodium arenesulfinates and aryl (and alkenyl) halides combining photoredox and nickel catalysis is reported here. The visible light-induced synergistic platform allows the facile construction of a wide spectrum of enantioenriched β-aryl and β-alkenyl sulfones with high efficiency and excellent enantioselectivity (up to 92% yield and up to 99:1 er) from readily accessible starting materials under mild conditions. Our protocol exhibits a high functional group tolerance and was readily extended to diverse complex molecules. Mechanistic investigations support the involvement of a secondary alkyl radical generated through radical addition of a sulfonyl radical to the double bond. Moreover, control experiments suggest that a Ni(0)/Ni(I)/Ni(III) catalytic cycle might be operative in these transformations.

## Supporting Information Available

Experimental procedures, characterization data, NMR spectra, HPLC traces, and crystallographic data (PDF)
