# Supplementary material for: Dual Nickel/Photoredox-Catalyzed Asymmetric Carbosulfonylation of Alkenes
Source: J Am Chem Soc. 2023 May 30;145(23):12532–40. doi: 10.1021/jacs.3c00744 (PMC10273242; doi:10.1021/jacs.3c00744)
Supplement: Supplementary file 1 — ja3c00744_si_001.pdf [file ja3c00744_si_001.pdf]

Dual Nickel/Photoredox-Catalyzed Asymmetric Carbosulfonylation of Alkenes

Xiaoyong Du, Iván Cheng-Sánchez and Cristina Nevado*

Department of Chemistry, University of Zurich, Winterthurerstrasse 190, CH 8057, Zurich, Switzerland.

Content

1. General Information 2

2. Asymmetric Carbosulfonylation of Alkenes via Nickel/Photoredox Dual Catalysis 3

3. Late-stage Functionalization 41

4. Mechanistic Studies 51

5. References 71

6. NMR Spectra 72

7. HPLC Spectra 134

1. General Information

Unless otherwise stated, reactions were carried out using dry solvents under nitrogen atmosphere. Starting materials were purchased from Aldrich, Fluka and TCI. Pentane, hexane and ethyl acetate were purchased with HPLC quality, degassed by purging thoroughly with nitrogen and dried over activated molecular sieves of appropriate size. Alternatively, tetrahydrofuran, acetonitrile and dichloromethane were dried using a solvent purification system (Pure Solv PS-MD-4EN, Innovative Technology Inc.) equipped with alumina drying columns under argon. Conversion was monitored by thin layer chromatography (TLC) using Merck TLC silica gel 60 F254 and visualized by UV-light at 254 nm. Flash column chromatography was performed over silica gel (230-400 mesh). All NMR spectra were recorded on AV2-400 or AV2-500 MHz Bruker spectrometers. Chemical shifts are given in ppm and the spectra are calibrated using the residual chloroform signals (7.26 ppm for ^{1}H NMR and 77.0 ppm for ^{13}C NMR) and the residual dichloromethane signals (2.05 ppm for ^{1}H NMR and 29.84 ppm, for ^{13}C NMR). Multiplicities are abbreviated as follows: singlet (s), doublet (d), triplet (t), quartet (q), doublet-doublet (dd), quintet (quint), septet (sept), multiplet (m), and broad (b). Infrared spectra were recorded on a JASCO FT/IR-4100 spectrometer. Absorptions are reported in wavenumber (cm-1). High-resolution electrospray ionization and electronic impact mass spectrometry were performed on a Finnigan MAT 900 (Thermo Finnigan, San Jose, CA; USA) double focusing magnetic sector mass spectrometer. Ten spectra were acquired. A mass accuracy ≤ 2 ppm was obtained in the peak matching acquisition mode by using a solution containing 2 <l PEG200, 2 <l PPG450, and 1.5 mg NaOAc (all obtained from Sigma-Aldrich, CH-Buchs) dissolved in 100 mL MeOH (HPLC Supra grade, Scharlau, E-Barcelona) as internal standard. Melting points were measured on melting point operators: MPM-MHV from Müller + Krempel AG. The enantiomeric ratios were determined by chiral HPLC analysis performed on JASCO HPLC system equipped with a PU-980 pump, a UV-970 detector, measured at 254 nm and a chiral column. Optical rotations were measured on a JASCO P-1010 polarimeter.

2. Asymmetric Carbosulfonylation of Alkenes via Nickel/Photoredox Dual Catalysis

2.1 Optimization

General procedure for optimization

An oven-dried 7.5 mL screw-cap vial equipped with a magnetic stirring bar was charged with the vinyl amide (0.1 mmol, 1 equiv), $PhSO_2Na$ (0.15 mmol, 1.5 equiv), 4-methoxyiodobenzene (0.2 mmol, 2 equiv), 4-CzIPN (0.001 mmol, 1 mol%) and **L**·$NiBr_2$ complex[1] (0.01 mmol, 10 mol%) and then introduced into a glove box filled with nitrogen. There, dry DME (2 mL) and 15-crown-5 (0.9 mmol, 9 equiv) were sequentially added. The reaction vessel was then capped and removed from the glove box to be stirred (1000 rpm) under irradiation at 0 ºC for 48 h. To quench the reaction, a saturated aq. solution of NaCl (1 mL) was added and the resulting mixture was extracted with EtOAc (3x2mL). The organic phase was concentrated in a rotary evaporator and the residue was purified by column chromatography on silica gel. The er values were determined by HPLC with a chiral column. The set-up of the photoredox reaction is shown in Figure S1. In addition, a Julabo chiller was used to keep the temperature inside the photoreactor at 0 °C.

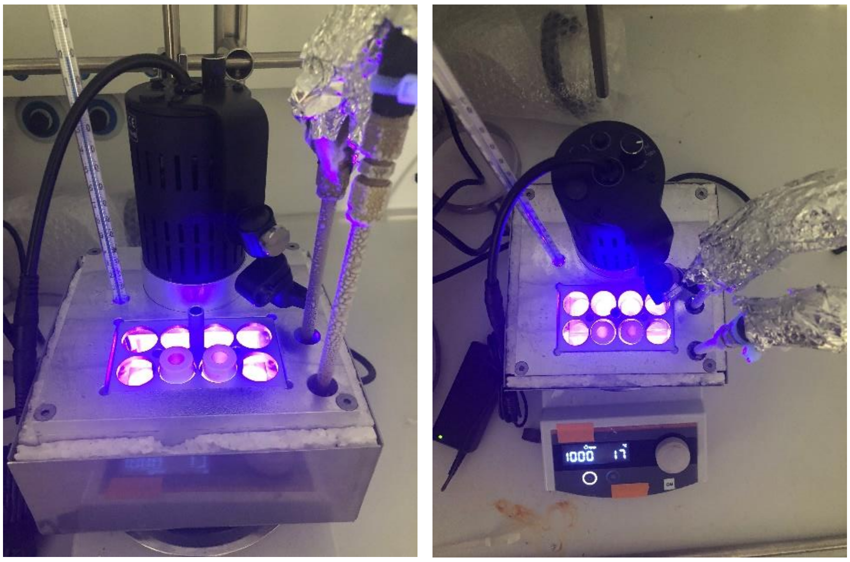

Figure S1. Reaction set-up

Table S1. Solvent screening[a]

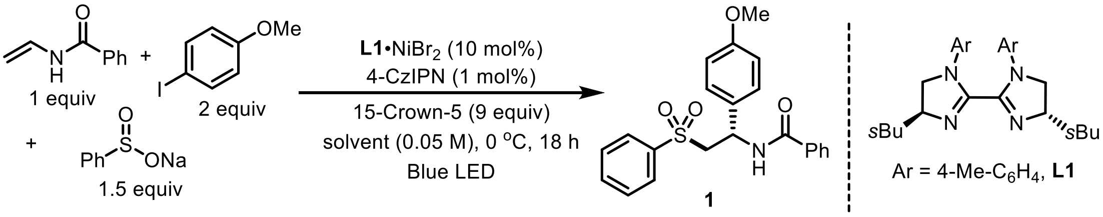

Entry	Solvent	Yield of **1** [%]	er
1[b]	CH_3CN	ND	--
2[b]	THF	ND	--
3[b]	Et_2O	ND	--
4[b]	DCE	ND	--
5[b]	DMSO	79	58:42
6[b]	DMA	58	62:38
7	CH_3CN	55	83:17
8	THF	51	87:13
9	Toluene	12	87:13
10	DCE	26	84:16
11	Acetone	53	88:12
12	EA	59	86:14
13	DME	60	90:10
14	Et_2O	52	86:14
15	Benzene	50	84:16
16	1,4-dioxane	20	89:11

[a]Reactions were carried out with vinyl amide (0.1 mmol), aryl iodide (0.2 mmol), $PhSO_2Na$ (0.15 mmol), **L1**·$NiBr_2$ (10 mol %), 4-CzIPN (1 mol %), 15-crown-5 (0.9 mmol), solvent [0.05 M], 34W Blue LED, 0 °C, 18 h. Isolated yields after column chromatography. Enantiomeric ratios (er) were determined by HPLC with a chiral stationary phase. [b]Without 15-crown-5.

Table S2. Temperature screening[a]

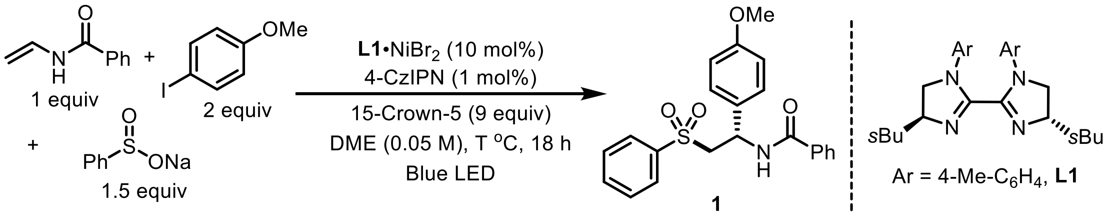

Entry	Temperature [°C]	Yield of **1** [%]	er
1	0	60	90:10
2	-10	57	90:10
3	-20	55	90:10
4	-30	49	91:9

[a]Reactions were carried out with vinyl amide (0.1 mmol), aryl iodide (0.2 mmol), $PhSO_2Na$ (0.15 mmol), **L1**·$NiBr_2$ (10 mol %), 4-CzIPN (1 mol %), 15-crown-5 (0.9 mmol), DME [0.05 M], 34W Blue LED, 18 h. Isolated yields after column chromatography. Enantiomeric ratios (er) were determined by HPLC with a chiral stationary phase.

Table S3. Concentration screening[a]

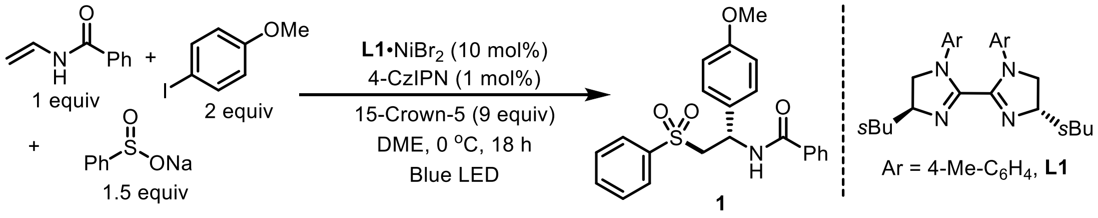

Entry	Conc. [M]	Yield of **1** [%]	er
1	0.05	60	90:10
2	0.1	53	88:12
3	0.025	71	91:9
4	0.01	54	92:8

[a]Reactions were carried out with vinyl amide (0.1 mmol), aryl iodide (0.2 mmol), $PhSO_2Na$ (0.15 mmol), **L1**·$NiBr_2$ (10 mol %), 4-CzIPN (1 mol %), 15-crown-5 (0.9 mmol), DME, 34W Blue LED, 0 °C, 18 h. Isolated yields after column chromatography. Enantiomeric ratios (er) were determined by HPLC with a chiral stationary phase.

Table S4. Ligand screening[a]

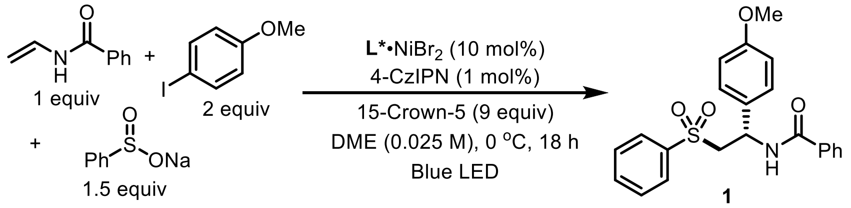

1

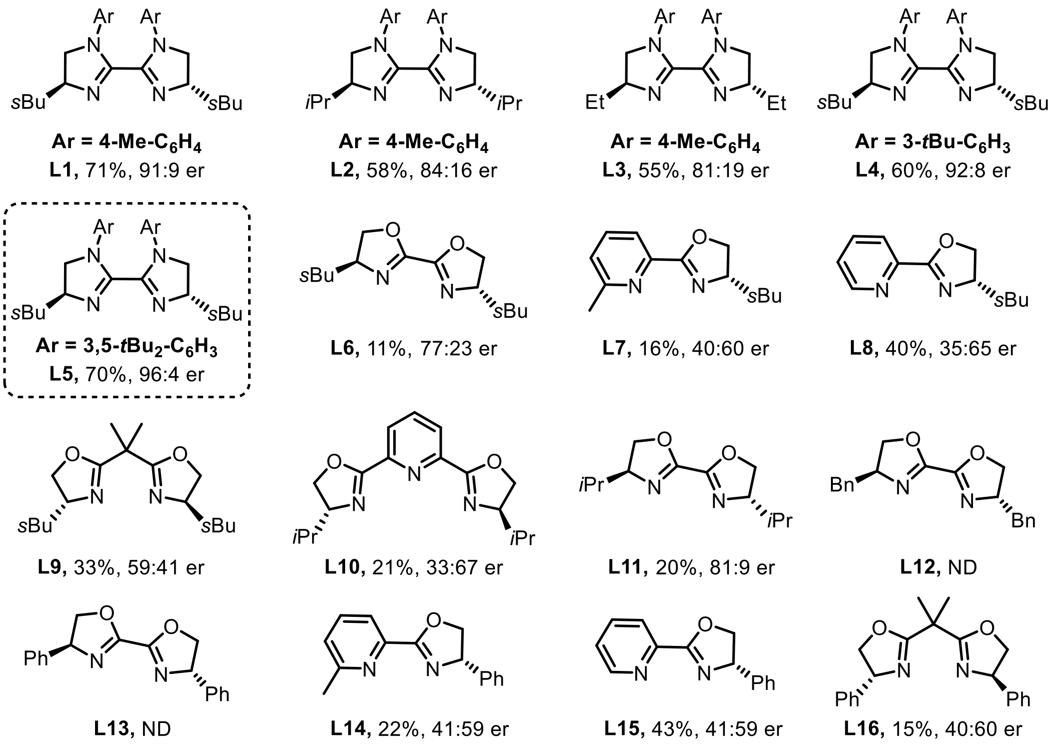

[a]Reactions were carried out with vinyl amide (0.1 mmol), aryl iodide (0.2 mmol), $PhSO_2Na$ (0.15 mmol), **L***·$NiBr_2$ (10 mol %), 4-CzIPN (1 mol %), 15-crown-5 (0.9 mmol), DME (0.025 M), 34W Blue LED, 0 °C, 18 h. Isolated yields after column chromatography. Enantiomeric ratios (er) were determined by HPLC with a chiral stationary phase.

Table S5. Light source screening[a]

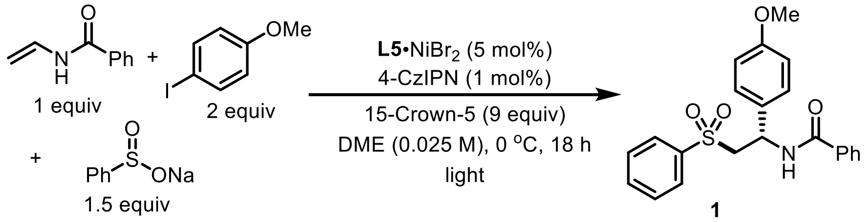

Entry	Light	Yield of **1** [%]	er
1	390 nm kessil lamp	57	96:4
2	427 nm kessil lamp	56	95:5
3	440 nm kessil lamp	47	95:5
4	456 nm kessil lamp	58	93:7
5	Blue LED	48	95:5
6[b]	Blue LED	50	95:5

[a]Reactions were carried out with vinyl amide (0.1 mmol), aryl iodide (0.2 mmol), $PhSO_2Na$ (0.15 mmol), **L5**·$NiBr_2$ (5 mol %), 4-CzIPN (1 mol %), 15-crown-5 (0.9 mmol), DME (0,025 M), 0 °C, 18 h. ^{1}H NMR yield with 1,3,5-trimethoxybenzene as internal standard. Enantiomeric ratios (er) were determined by HPLC with a chiral stationary phase. [b]3 mol% of 4-CzIPN.

Table S6. Screening of reaction stoichiometry, time and catalyst loading[a]

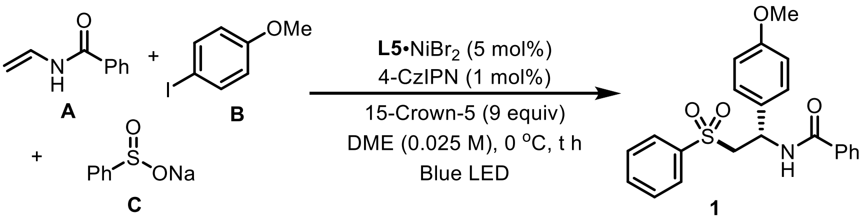

Entry	Ratio (**A**/**B**/**C**)	Yield of **1** [%]	er
1	1/2/1.5	48	95:5
2	2/1/2	64	95:5
3	2/2/1	60	95:5
4[b,c]	2/1/2	77	95:5
5[b,c,d]	2/1/2	82 (76)[e]	96:4

[a]Reactions were carried out with vinyl amide, aryl iodide, $PhSO_2Na$, **L5**·$NiBr_2$ (5 mol %), 4-CzIPN (1 mol %), 15-crown-5 (0.9 mmol), DME (0,025 M), 0 °C, 18 h. ^{1}H NMR yield with 1,3,5-trimethoxybenzene as internal standard. Enantiomeric ratios (er) were determined by HPLC with a chiral stationary phase. [b]48 h. [c]Using 10 mol% of **L5**·$NiBr_2$. [d]390 nm kessil lamp. [e]Isolated yields after column chromatography.

Table S7. Control experiments[a]

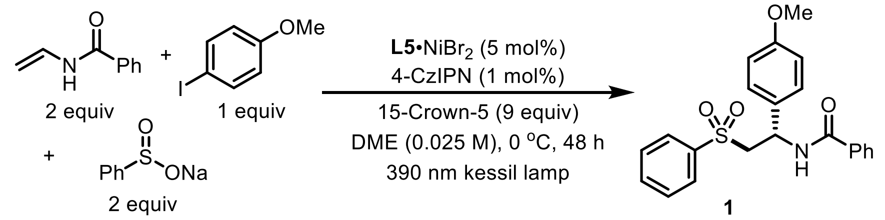

Entry	Reaction component omitted	Yield of **1** [%]	er
1	none	76	96:4
2	nickel	0	--
3	photocatalyst	0	--
4	light	0	--

[a]Reactions were carried out with vinyl amide (0.2 mmol), aryl iodide (0.1 mmol), $PhSO_2Na$ (0.2 mmol), **L5**·$NiBr_2$ (5 mol %), 4-CzIPN (1 mol %), 15-crown-5 (0.9 mmol), DME (0,025 M), 0 °C, 48 h. Isolated yields after column chromatography.

Table S8. Unsuccessful partners in the asymmetric carbosulfonylation.

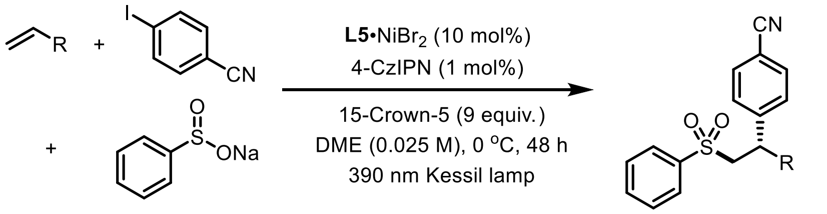

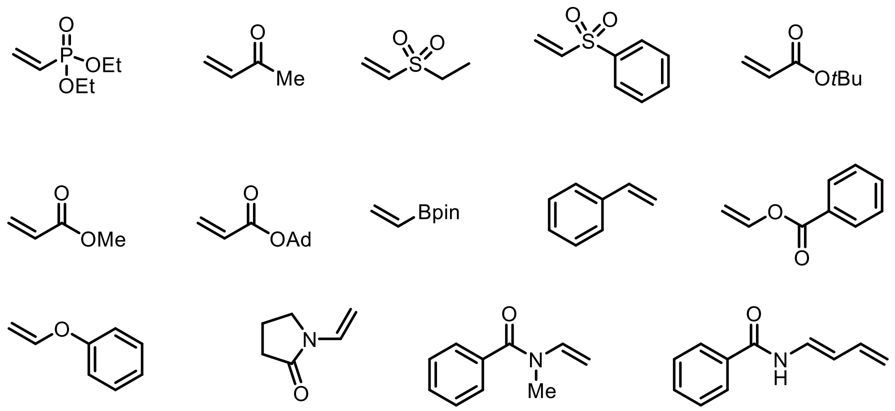

[a]Reactions were carried out with alkenes (0.2 mmol), aryl iodide (0.1 mmol), $PhSO_2Na$ (0.2 mmol), **L5**·$NiBr_2$ (5 mol %), 4-CzIPN (1 mol %), 15-crown-5 (0.9 mmol), DME (0,025 M), 0 °C, 48 h. Isolated yields after column chromatography.

2.2 Synthesis of vinyl amides

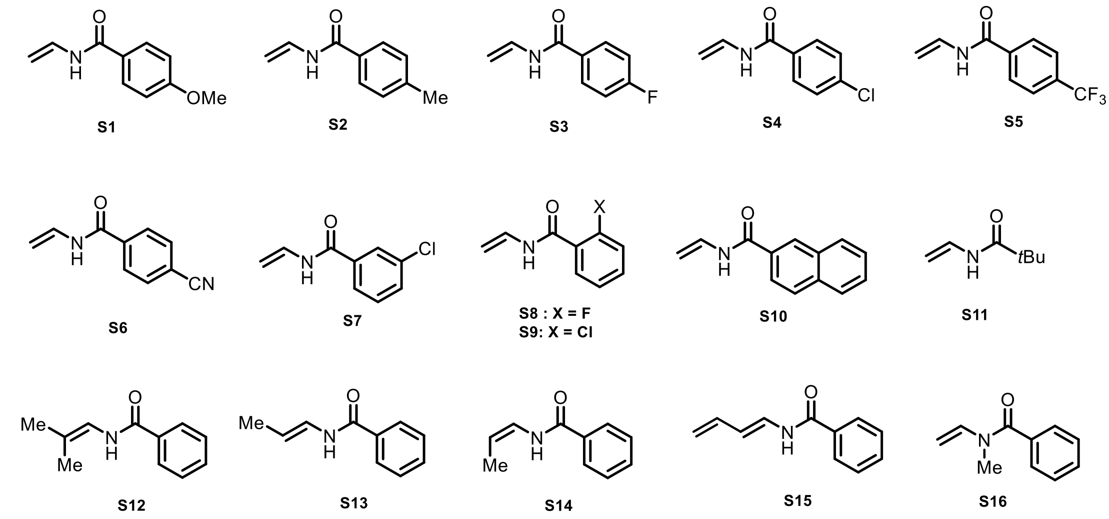

Vinyl amides **S1**,[2] **S2**,[2] **S3**,[2] **S4**,[2] **S5**,[2] **S6**,[2] **S7**,[3] **S8**,[3] **S9**,[3] **S10**,[2] **S11**,[4] **S12**,[5] **S13**,[6] **S14**,[6] **S15**[7] and **S16**[8] were prepared according to reported procedures and analytical data were in agreement with previously reported values.

2.3 General Procedure for Asymmetric Carbosulfonylation of Alkenes.

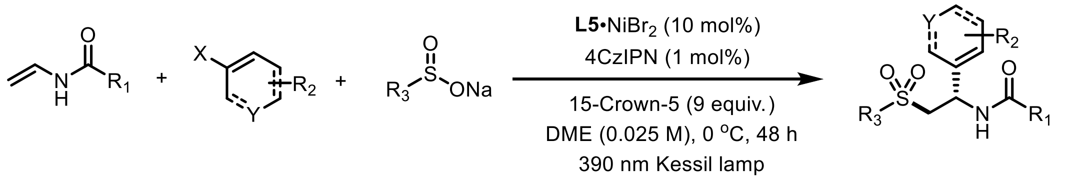

An oven-dried 7.5 mL screw-cap vial equipped with a magnetic stirring bar was charged with the vinyl amide (0.2 mmol, 2 equiv), $PhSO_2Na$ (0.2 mmol, 2 equiv), aryl iodide or alkenyl bromide (If solid, 0.1 mmol, 1 equiv), 4-CzIPN (0.001 mmol, 1 mol%) and **L5**·$NiBr_2$ complex[1] (0.01 mmol, 10 mol%) and then introduced into a glove box filled with nitrogen. There, dry DME (4 mL) and 15-crown-5 (0.9 mmol, 9 equiv) were sequentially added. The reaction vessel was then capped and removed from the glove box. After that, aryl iodide or alkenyl bromide (if liquid, 0.1 mmol, 1 equiv) was added. The reaction was stirred (1000 rpm) under irradiation with 390 nm kessil lamp at 0 °C for 48 h. To quench the reaction, a saturated aq. solution of NaCl (1 mL) was added and

the resulting mixture was extracted with EtOAc (3x2mL). The organic phase was concentrated in a rotary evaporator and the residue was purified by column chromatography on silica gel. The er values were determined by HPLC with a chiral column.

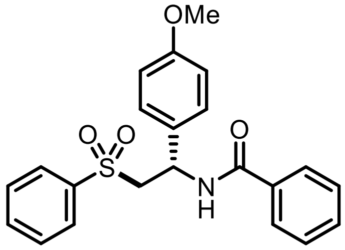

(*S*)-*N*-(1-(4-Methoxyphenyl)-2-(phenylsulfonyl)ethyl)benzamide (1). White solid, 76% yield, 96:4 er, $[\alpha]_D^{25}$ = -24.60 (c = 0.14, $CHCl_3$). ^1H NMR (400 MHz, $CDCl_3$) δ 7.86 – 7.77 (m, 4H), 7.60 (d, *J* = 7.5 Hz, 1H), 7.55 – 7.42 (m, 5H), 7.32 (d, *J* = 6.6 Hz, 1H), 7.17 (d, *J* = 8.7 Hz, 2H), 6.79 (d, *J* = 8.7 Hz, 2H), 5.41 (ddd, *J* = 8.8, 6.6, 4.5 Hz, 1H), 3.83 (dd, *J* = 14.7, 8.8 Hz, 1H), 3.76 (s, 3H), 3.59 (dd, *J* = 14.8, 4.5 Hz, 1H). ^{13}C NMR (101 MHz, $CDCl_3$) δ 166.8, 159.5, 139.0, 134.1, 133.9, 132.0, 131.0, 129.5, 128.8, 128.1, 127.6, 127.3, 114.4, 60.5, 55.4, 49.8. IR (film): ν (cm^{-1}) 3396, 2967, 2914, 1747, 1296, 1170, 1156, 988, 789, 751. HR-MS (ESI) calculated $[M+H]^+$ for $C_{22}H_{22}NO_4S^+$ = 396.12641, found: 396.12630. The enantiomeric excess of **1** was determined by HPLC analysis on Chiralpak AD-H column. Conditions: hexane/isopropanol = 70/30, flow rate = 1.0 mL/min, uv-vis detection at λ = 254 nm, t_R = 15.1 min (major), 28.7 min (minor).

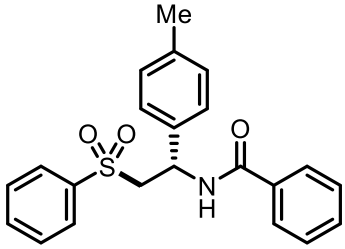

(*S*)-*N*-(2-(Phenylsulfonyl)-1-(*p*-tolyl)ethyl)benzamide (2). White solid, 70% yield, 95:5 er, $[\alpha]_D^{25}$ = -31.17 (c = 0.13, $CHCl_3$). ^1H NMR (400 MHz, $CDCl_3$) δ 7.84 – 7.76

(m, 4H), 7.58 (t, J = 7.5 Hz, 1H), 7.45 (ddt, J = 22.9, 15.1, 7.2 Hz, 6H), 7.11 (d, J = 8.2 Hz, 2H), 7.04 (d, J = 7.9 Hz, 2H), 5.49 – 5.40 (m, 1H), 3.84 (dd, J = 14.8, 9.1 Hz, 1H), 3.55 (dd, J = 14.8, 4.2 Hz, 1H), 2.27 (s, 3H). ^{13}C NMR (101 MHz, $CDCl_3$) δ 166.8, 139.0, 137.9, 136.1, 134.0, 133.9, 131.9, 129.7, 129.5, 128.7, 128.1, 127.3, 126.2, 60.5, 49.9, 21.1. IR (film): ν (cm^{-1}) 3324, 2921, 1633, 1537, 1295, 1084, 823, 741. HR-MS (ESI) calculated $[M+H]^+$ for $C_{22}H_{22}NO_3S^+$ = 380.13149, found: 380.13135. The enantiomeric excess of **2** was determined by HPLC analysis on Chiralpak AD-H column. Conditions: hexane/isopropanol = 80/20, flow rate = 1.0 mL/min, uv-vis detection at λ = 220 nm, t_R = 23.3 min (major), 38.9 min (minor).

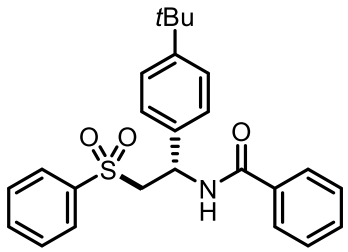

(*S*)-*N*-(1-(4-(Tert-butyl)phenyl)-2-(phenylsulfonyl)ethyl)benzamide (3). White solid, 57% yield, 94:6 er, $[\alpha]_D^{25}$ = -32.23 (c = 0.17, $CHCl_3$). ^{1}H NMR (400 MHz, $CDCl_3$) δ 7.79 – 7.74 (m, 2H), 7.71 (dd, J = 8.4, 1.3 Hz, 2H), 7.53 – 7.42 (m, 2H), 7.38 (td, J = 7.5, 7.1, 2.8 Hz, 4H), 7.29 (d, J = 6.9 Hz, 1H), 7.23 – 7.14 (m, 3H), 7.08 (d, J = 8.4 Hz, 2H), 5.40 (ddd, J = 8.7, 6.8, 4.3 Hz, 1H), 3.78 (dd, J = 14.8, 8.7 Hz, 1H), 3.55 (dd, J = 14.8, 4.3 Hz, 1H), 1.19 (s, 9H). ^{13}C NMR (101 MHz, $CDCl_3$) δ 166.8, 151.1, 139.1, 135.7, 134.0, 133.9, 132.0, 129.5, 128.8, 128.1, 127.3, 126.0, 126.0, 60.5, 49.8, 34.6, 31.4. IR (film): ν (cm^{-1}) 3349, 3279, 2926, 1644, 1534, 1324, 1295, 1142, 1085, 752, 723. HR-MS (ESI) calculated $[M+H]^+$ for $C_{25}H_{28}NO_3S^+$ = 422.17844, found: 422.17814. The enantiomeric excess of **3** was determined by HPLC analysis on Chiralpak AD-H column. Conditions: hexane/isopropanol = 80/20, flow rate = 1.0 mL/min, uv-vis detection at λ = 210 nm, t_R = 14.1 min (major), 25.9 min (minor).

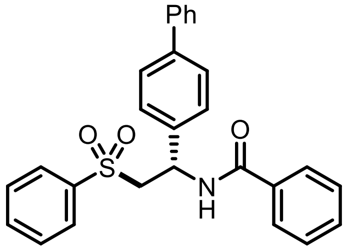

(*S*)-*N*-(1-([1,1'-Biphenyl]-4-yl)-2-(phenylsulfonyl)ethyl)benzamide (4). White solid, 59% yield, 95:5 er, $[\alpha]_D^{25}$ = -32.46 (c = 0.16, $CHCl_3$). ^1H NMR (400 MHz, $CDCl_3$) δ 7.81 – 7.75 (m, 2H), 7.75 – 7.69 (m, 2H), 7.54 – 7.31 (m, 13H), 7.30 – 7.24 (m, 1H), 7.23 – 7.18 (m, 2H), 5.46 (ddd, *J* = 8.7, 6.7, 4.1 Hz, 1H), 3.81 (dd, *J* = 14.8, 8.9 Hz, 1H), 3.56 (dd, *J* = 14.9, 4.2 Hz, 1H). ^{13}C NMR (101 MHz, $CDCl_3$) δ 166.9, 141.2, 140.6, 139.0, 137.8, 134.1, 133.8, 132.1, 129.5, 128.9, 128.8, 128.1, 127.8, 127.6, 127.3, 127.2, 126.7, 60.5, 50.0. IR (film): ν (cm^{-1}) 3304, 2980, 2957, 2360, 1732, 1637, 1265, 1083, 839, 746. HR-MS (ESI) calculated $[M-H]^-$ for $C_{27}H_{22}NO_3S^-$ = 440.13259, found: 440.13292. The enantiomeric excess of **4** was determined by HPLC analysis on Chiralpak AD-H column. Conditions: hexane/isopropanol = 70/30, flow rate = 1.0 mL/min, uv-vis detection at λ = 254 nm, t_R = 16.9 min (major), 30.5 min (minor).

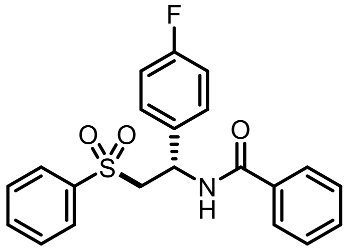

(*S*)-*N*-(1-(4-Fluorophenyl)-2-(phenylsulfonyl)ethyl)benzamide (5). White solid, 57% yield, 96:4 er, $[\alpha]_D^{25}$ = -30.37 (c = 0.22, $CHCl_3$). ^1H NMR (400 MHz, $CDCl_3$) δ 7.82 (ddd, *J* = 9.7, 8.4, 1.3 Hz, 4H), 7.65 – 7.59 (m, 1H), 7.57 – 7.41 (m, 6H), 7.25 – 7.19 (m, 2H), 6.93 (t, *J* = 8.6 Hz, 2H), 5.46 (ddd, *J* = 8.9, 6.5, 4.2 Hz, 1H), 3.82 (dd, *J* = 14.8, 9.0 Hz, 1H), 3.57 (dd, *J* = 14.8, 4.3 Hz, 1H). ^{13}C NMR (101 MHz, $CDCl_3$) δ 166.9, 162.4 (d, *J* = 247.1 Hz), 138.9, 134.8 (d, *J* = 3.4 Hz), 134.2, 133.6, 132.1, 129.6, 128.8, 128.1, 128.1, 127.3, 115.9 (d, *J* = 21.7 Hz), 60.4, 49.7. ^{19}F NMR (377 MHz, $CDCl_3$) δ -113.9. IR (film): ν (cm^{-1}) 3328, 2980, 2958, 1636, 1525, 1304, 1278, 1140, 1082, 707.

HR-MS (ESI) calculated $[M+H]^+$ for $C_{21}H_{19}FNO_3S^+$ = 384.10642, found: 384.10619. The enantiomeric excess of **5** was determined by HPLC analysis on Chiralpak AD-H column. Conditions: hexane/isopropanol = 80/20, flow rate = 1.0 mL/min, uv-vis detection at λ = 220 nm, t_R = 19.3 min (major), 48.1 min (minor).

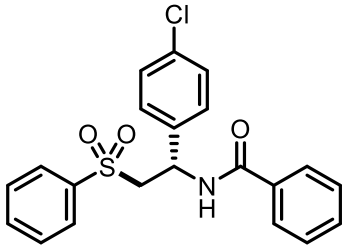

(*S*)-*N*-(1-(4-Chlorophenyl)-2-(phenylsulfonyl)ethyl)benzamide (6). White solid, 56% yield, 96:4 er, $[\alpha]_D^{25}$ = -53.00 (c = 0.22, $CHCl_3$). ^1H NMR (400 MHz, $CDCl_3$) δ 7.81 (ddd, *J* = 14.4, 8.4, 1.3 Hz, 4H), 7.66 – 7.58 (m, 2H), 7.55 – 7.41 (m, 5H), 7.23 – 7.13 (m, 4H), 5.46 (ddd, *J* = 8.9, 6.6, 4.1 Hz, 1H), 3.81 (dd, *J* = 14.8, 8.9 Hz, 1H), 3.55 (dd, *J* = 14.8, 4.1 Hz, 1H). ^{13}C NMR (101 MHz, $CDCl_3$) δ 166.9, 138.8, 137.5, 134.2, 134.0, 133.5, 132.2, 129.6, 129.2, 128.8, 128.0, 127.7, 127.3, 60.2, 49.7. IR (film): ν (cm^{-1}) 3320, 2980, 2360, 1636, 1509, 1304, 1141, 1082, 838, 744. HR-MS (ESI) calculated $[M+H]^+$ for $C_{21}H_{19}ClNO_3S^+$ = 400.07687, found: 400.07704. The enantiomeric excess of **6** was determined by HPLC analysis on Chiralpak AD-H column. Conditions: hexane/isopropanol = 80/20, flow rate = 1.0 mL/min, uv-vis detection at λ = 220 nm, t_R = 21.6 min (major), 54.4 min (minor).

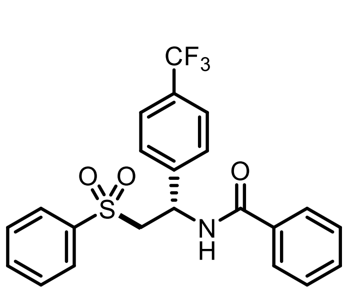

(*S*)-*N*-(2-(Phenylsulfonyl)-1-(4-(trifluoromethyl)phenyl)ethyl)benzamide (7). White solid, 69% yield, 96:4 er, $[\alpha]_D^{25}$ = -37.63 (c = 0.22, $CHCl_3$). ^1H NMR (400 MHz, $CDCl_3$) δ 7.86 (d, *J* = 8.1 Hz, 2H), 7.80 – 7.72 (m, 3H), 7.61 (t, *J* = 7.5 Hz, 1H), 7.55

(t, J = 7.4 Hz, 1H), 7.52 – 7.43 (m, 6H), 7.35 (d, J = 8.3 Hz, 2H), 5.56 (ddd, J = 8.9, 6.4, 4.0 Hz, 1H), 3.83 (dd, J = 14.8, 8.8 Hz, 1H), 3.65 – 3.55 (m, 1H). ^{13}C NMR (101 MHz, $CDCl_3$) δ 166.9, 142.8, 138.8, 134.4, 133.4, 132.3, 130.4 (q, J = 32.7 Hz), 129.7, 128.9, 128.0, 127.3, 126.7, 126.0 (q, J = 3.8 Hz), 125.3, 122.6, 60.1, 50.0. ^{19}F NMR (377 MHz, $CDCl_3$) δ -62.7. IR (film): ν (cm^{-1}) 3320, 2983, 1621, 1524, 1305, 1158, 1085, 841, 736. HR-MS (ESI) calculated $[M+H]^+$ for $C_{22}H_{19}F_3NO_3S^+$ = 434.10323, found: 434.10284. The enantiomeric excess of **7** was determined by HPLC analysis on Chiralpak AD-H column. Conditions: hexane/isopropanol = 80/20, flow rate = 1.0 mL/min, uv-vis detection at λ = 220 nm, t_R = 16.0 min (major), 44.8 min (minor).

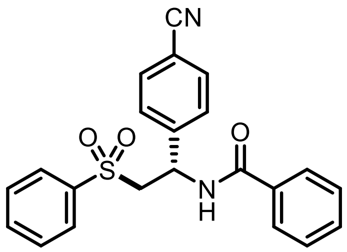

(*S*)-*N*-(1-(4-Cyanophenyl)-2-(phenylsulfonyl)ethyl)benzamide (8). White solid, 64% yield, 97:3 er, $[\alpha]_D^{25}$ = -62.54 (c = 0.13, $CHCl_3$). ^{1}H NMR (400 MHz, $CDCl_3$) δ 7.89 – 7.80 (m, 4H), 7.78 (d, J = 6.2 Hz, 1H), 7.68 – 7.63 (m, 1H), 7.59 – 7.44 (m, 7H), 7.39 (d, J = 8.4 Hz, 2H), 5.52 (ddd, J = 9.5, 6.1, 3.9 Hz, 1H), 3.78 (dd, J = 14.8, 9.0 Hz, 1H), 3.57 (dd, J = 14.7, 3.9 Hz, 1H). ^{13}C NMR (101 MHz, $CDCl_3$) δ 167.0, 144.3, 138.7, 134.6, 133.1, 132.8, 132.4, 129.8, 129.0, 128.0, 127.3, 127.2, 118.4, 112.1, 59.8, 50.2. IR (film): ν (cm^{-1}) 3279, 2229, 1636, 1536, 1302, 1143, 882, 724. HR-MS (ESI) calculated $[M+H]^+$ for $C_{22}H_{19}N_2O_3S^+$ = 391.11109, found: 391.11102. The enantiomeric excess of **8** was determined by HPLC analysis on Chiralpak AD-H column. Conditions: hexane/isopropanol = 70/30, flow rate = 1.0 mL/min, uv-vis detection at λ = 254 nm, t_R = 17.6 min (major), 49.6 min (minor).

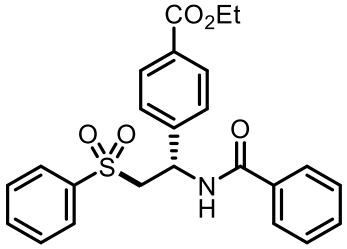

Ethyl (*S*)-4-(1-benzamido-2-(phenylsulfonyl)ethyl)benzoate (9). White solid, 78% yield, 96:4 er, $[\alpha]_D^{25}$ = -53.36 (c = 0.22, $CHCl_3$). ^1H NMR (400 MHz, $CDCl_3$) δ 7.96 – 7.90 (m, 2H), 7.88 – 7.79 (m, 4H), 7.66 – 7.58 (m, 2H), 7.56 – 7.43 (m, 5H), 7.31 (d, *J* = 8.4 Hz, 2H), 5.51 (ddd, *J* = 9.7, 6.3, 4.0 Hz, 1H), 4.35 (q, *J* = 7.1 Hz, 2H), 3.81 (dd, *J* = 14.8, 9.2 Hz, 1H), 3.58 (dd, *J* = 14.8, 4.1 Hz, 1H), 1.37 (t, *J* = 7.1 Hz, 3H). ^{13}C NMR (101 MHz, $CDCl_3$) δ 166.9, 166.1, 143.9, 138.8, 134.3, 133.5, 132.2, 130.4, 130.3, 129.7, 128.9, 128.1, 127.3, 126.3, 61.2, 60.1, 50.2, 14.4. IR (film): ν (cm^{-1}) 3412, 2980, 2360, 1715, 1523, 1294, 1266, 1141, 1082, 745. HR-MS (ESI) calculated $[M-H]^-$ for $C_{24}H_{22}NO_5S^-$ = 436.12242, found: 436.12279. The enantiomeric excess of **9** was determined by HPLC analysis on Chiralpak OD-H column. Conditions: hexane/isopropanol = 75/25, flow rate = 0.5 mL/min, uv-vis detection at λ = 254 nm, t_R = 68.3 min (minor), 74.9 min (major).

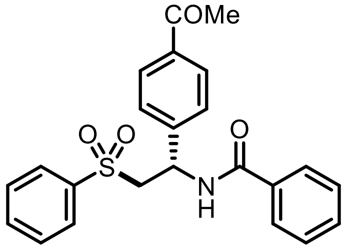

(*S*)-*N*-(1-(4-Acetylphenyl)-2-(phenylsulfonyl)ethyl)benzamide (10). White solid, 50% yield, 97:3 er, $[\alpha]_D^{25}$ = -44.02 (c = 0.16, $CHCl_3$). ^1H NMR (400 MHz, $CDCl_3$) δ 7.83 (ddd, *J* = 8.7, 6.9, 1.9 Hz, 6H), 7.74 – 7.66 (m, 1H), 7.65 – 7.59 (m, 1H), 7.55 – 7.42 (m, 5H), 7.36 (dd, *J* = 8.4, 3.4 Hz, 2H), 5.52 (ddd, *J* = 9.9, 6.4, 3.6 Hz, 1H), 3.89 – 3.74 (m, 1H), 3.66 – 3.52 (m, 1H), 2.55 (d, *J* = 3.1 Hz, 3H). ^{13}C NMR (101 MHz, $CDCl_3$) δ 197.5, 166.9, 144.2, 138.8, 136.9, 134.4, 133.4, 132.2, 129.7, 129.1, 128.9, 128.8, 128.1, 127.5, 127.3, 126.6, 60.0, 50.2, 26.8. IR (film): ν (cm^{-1}) 3404, 2927, 1675, 1489, 1291,

1082, 855, 750. HR-MS (ESI) calculated [M+H]$^+$ for $C_{23}H_{22}NO_4S^+$ = 408.12641, found: 408.12649. The enantiomeric excess of **10** was determined by HPLC analysis on Chiralpak AD-H column. Conditions: hexane/isopropanol = 60/40, flow rate = 1.0 mL/min, uv-vis detection at λ = 254 nm, t_R = 12.4 min (major), 36.1 min (minor).

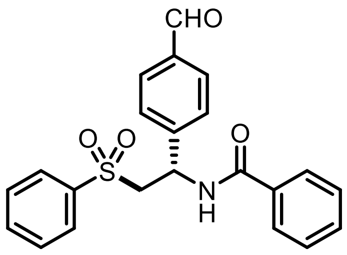

(*S*)-*N*-(1-(4-Formylphenyl)-2-(phenylsulfonyl)ethyl)benzamide (11). White solid, 51% yield, 97:3 er, $[\alpha]_D^{25}$ = -43.12 (c = 0.13, $CHCl_3$). ^1H NMR (400 MHz, $CDCl_3$) δ 9.96 (s, 1H), 7.91 – 7.86 (m, 2H), 7.81 (dd, *J* = 14.1, 7.6 Hz, 4H), 7.72 (d, *J* = 6.2 Hz, 1H), 7.66 – 7.60 (m, 1H), 7.59 – 7.54 (m, 1H), 7.53 – 7.42 (m, 6H), 5.53 (ddd, *J* = 9.6, 6.0, 3.9 Hz, 1H), 3.80 (dd, *J* = 14.8, 9.1 Hz, 1H), 3.60 (dd, *J* = 14.7, 4.0 Hz, 1H). ^{13}C NMR (101 MHz, $CDCl_3$) δ 191.5, 166.8, 145.5, 138.6, 136.1, 134.3, 133.2, 132.2, 130.3, 129.6, 128.8, 127.9, 127.2, 126.9, 59.9, 50.2. IR (film): ν (cm^{-1}) 3351, 2980, 2360, 1732, 1696, 1640, 1521, 1295, 1143, 1085, 840, 798, 751. HR-MS (ESI) calculated [M+H]$^+$ for $C_{22}H_{20}NO_4S^+$ = 394.11076, found: 394.11082. The enantiomeric excess of **11** was determined by HPLC analysis on Chiralpak AD-H column. Conditions: hexane/isopropanol = 70/30, flow rate = 1.0 mL/min, uv-vis detection at λ = 220 nm, t_R = 19.0 min (major), 46.4 min (minor).

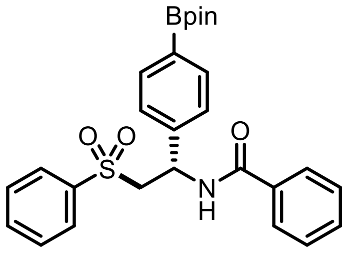

(*S*)-*N*-(2-(Phenylsulfonyl)-1-(4-(4,4,5,5-tetramethyl-1,3,2-dioxaborolan-2-yl)phenyl)ethyl)benzamide (12). White solid, 68% yield, 95:5 er, $[\alpha]_D^{25}$ = -27.41 (c

= 0.15, $CHCl_3$). ^1H NMR (400 MHz, $CDCl_3$) δ 7.82 (ddd, J = 11.8, 8.4, 1.3 Hz, 4H), 7.71 (d, J = 8.2 Hz, 2H), 7.63 – 7.57 (m, 1H), 7.55 – 7.41 (m, 6H), 7.24 (d, J = 7.9 Hz, 2H), 5.44 (ddd, J = 10.3, 6.6, 4.1 Hz, 1H), 3.79 (dd, J = 14.8, 9.2 Hz, 1H), 3.59 (dd, J = 14.8, 4.2 Hz, 1H), 1.32 (s, 12H), 1.24 (s, 4H). ^{13}C NMR (101 MHz, $CDCl_3$) δ 166.8, 142.0, 138.8, 135.6, 134.2, 133.8, 132.1, 129.6, 128.8, 128.1, 127.3, 125.6, 84.0, 60.4, 50.4, 29.9, 25.0, 25.0, 24.9. IR (film): ν (cm^{-1}) 3288, 1747, 1533, 1341, 1157, 1070, 988, 798, 751. HR-MS (ESI) calculated $[M+H]^+$ for $C_{27}H_{31}BNO_5S^+$ = 492.20105, found: 492.20098. The enantiomeric excess of **12** was determined by HPLC analysis on Chiralpak AD-H column. Conditions: hexane/isopropanol = 80/20, flow rate = 1.0 mL/min, uv-vis detection at λ = 220 nm, t_R = 31.8 min (major), 42.2 min (minor).

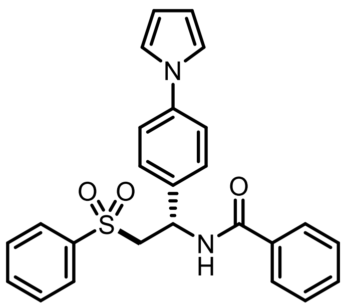

(*S*)-*N*-(1-(4-(1H-Pyrrol-1-yl)phenyl)-2-(phenylsulfonyl)ethyl)benzamide (13). White solid, 60% yield, 97:3 er, $[\alpha]_D^{25}$ = -19.20 (c = 0.19, $CHCl_3$). ^1H NMR (400 MHz, $CDCl_3$) δ 7.83 (ddd, J = 13.8, 8.4, 1.3 Hz, 4H), 7.66 – 7.56 (m, 2H), 7.55 – 7.41 (m, 5H), 7.31 – 7.21 (m, 4H), 7.00 (t, J = 2.2 Hz, 2H), 6.33 (t, J = 2.2 Hz, 2H), 5.52 (ddd, J = 8.9, 6.6, 4.1 Hz, 1H), 3.87 (dd, J = 14.8, 8.9 Hz, 1H), 3.60 (dd, J = 14.8, 4.1 Hz, 1H). ^{13}C NMR (101 MHz, $CDCl_3$) δ 166.9, 140.5, 138.9, 136.2, 134.2, 133.6, 132.1, 129.6, 128.8, 128.1, 127.6, 127.3, 120.9, 119.3, 110.8, 60.3, 49.7. IR (film): ν (cm^{-1}) 3343, 2980, 2360, 1731, 1646, 1521, 1509, 1289, 1143, 834, 722. HR-MS (ESI) calculated $[M+H]^+$ for $C_{25}H_{23}N_2O_3S^+$ = 431.14239, found: 431.14253. The enantiomeric excess of **13** was determined by HPLC analysis on Chiralpak AD-H column. Conditions: hexane/isopropanol = 70/30, flow rate = 1.0 mL/min, uv-vis detection at λ = 254 nm, t_R = 22.0 min (major), 52.6 min (minor).

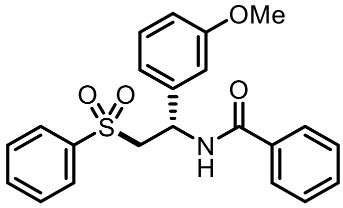

(*S*)-*N*-(1-(3-Methoxyphenyl)-2-(phenylsulfonyl)ethyl)benzamide (14). White solid, 81% yield, 96:4 er, $[\alpha]_D^{25}$ = -25.54 (c = 0.15, $CHCl_3$). ^{1}H NMR (400 MHz, $CDCl_3$) δ 7.88 – 7.78 (m, 4H), 7.63 – 7.58 (m, 1H), 7.49 (ddt, *J* = 15.2, 13.3, 7.3 Hz, 5H), 7.40 (d, *J* = 6.7 Hz, 1H), 7.18 (t, *J* = 8.2 Hz, 1H), 6.83 (d, *J* = 7.8 Hz, 1H), 6.75 (d, *J* = 7.2 Hz, 2H), 5.43 (ddd, *J* = 9.1, 6.5, 4.2 Hz, 1H), 3.80 (dd, *J* = 14.8, 9.1 Hz, 1H), 3.73 (s, 3H), 3.60 (dd, *J* = 14.8, 4.2 Hz, 1H). ^{13}C NMR (101 MHz, $CDCl_3$) δ 166.8, 160.1, 140.6, 138.9, 134.1, 133.8, 132.1, 130.2, 129.5, 128.8, 128.1, 127.3, 118.5, 113.4, 112.3, 60.5, 55.4, 50.2. IR (film): ν (cm^{-1}) 3315, 2955, 1639, 1541, 1300, 1287, 1139, 1052, 863, 761, 740. HR-MS (ESI) calculated $[M+H]^+$ for $C_{22}H_{22}NO_4S^+$ = 396.12641, found: 396.12620. The enantiomeric excess of **14** was determined by HPLC analysis on Chiralpak AD-H column. Conditions: hexane/isopropanol = 80/20, flow rate = 1.0 mL/min, uv-vis detection at λ = 210 nm, t_R = 24.9 min (major), 41.4 min (minor).

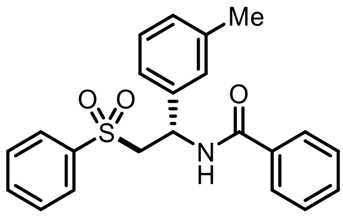

(*S*)-*N*-(2-(Phenylsulfonyl)-1-(*m*-tolyl)ethyl)benzamide (15). White solid, 65% yield, 96:4 er, $[\alpha]_D^{25}$ = -30.00 (c = 0.22, $CHCl_3$). ^{1}H NMR (400 MHz, $CDCl_3$) δ 7.82 (ddd, *J* = 15.7, 8.4, 1.3 Hz, 4H), 7.62 – 7.57 (m, 1H), 7.55 – 7.43 (m, 5H), 7.40 (d, *J* = 6.7 Hz, 1H), 7.15 (t, *J* = 7.9 Hz, 1H), 7.07 – 6.99 (m, 3H), 5.43 (ddd, *J* = 9.0, 6.6, 4.2 Hz, 1H), 3.81 (dd, *J* = 14.8, 9.1 Hz, 1H), 3.59 (dd, *J* = 14.8, 4.2 Hz, 1H), 2.26 (s, 3H). ^{13}C NMR (101 MHz, $CDCl_3$) δ 166.8, 139.0, 138.9, 138.8, 134.1, 133.9, 132.0, 129.5, 129.0, 129.0, 128.8, 128.1, 127.3, 127.0, 123.3, 60.5, 50.2, 21.5. IR (film): ν (cm^{-1}) 3351, 2980, 1645, 1525, 1293, 1134, 1084, 864, 743. HR-MS (ESI) calculated $[M+H]^+$ for $C_{22}H_{22}NO_3S^+$ = 380.13149, found: 380.13152. The enantiomeric excess of **15** was

determined by HPLC analysis on Chiralpak AD-H column. Conditions: hexane/isopropanol = 85/15, flow rate = 1.0 mL/min, uv-vis detection at λ = 210 nm, t_R = 26.4 min (major), 40.4 min (minor).

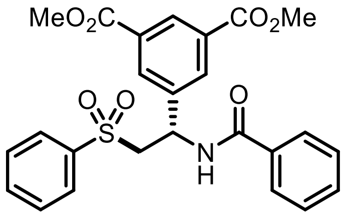

Dimethyl (*S*)-5-(1-benzamido-2-(phenylsulfonyl)ethyl)isophthalate (16). White solid, 45% yield, 95:5 er, $[\alpha]_D^{25}$ = -16.45 (c = 0.22, $CHCl_3$). ^1H NMR (400 MHz, $CDCl_3$) δ 8.53 (t, *J* = 1.5 Hz, 1H), 8.13 (d, *J* = 1.5 Hz, 2H), 7.88 – 7.79 (m, 4H), 7.74 (d, *J* = 6.1 Hz, 1H), 7.65 – 7.57 (m, 1H), 7.49 (tt, *J* = 17.9, 7.7 Hz, 5H), 5.55 (ddd, *J* = 9.8, 6.1, 4.0 Hz, 1H), 3.91 (s, 6H), 3.84 (dd, *J* = 14.7, 9.5 Hz, 1H), 3.58 (dd, *J* = 14.7, 4.0 Hz, 1H). ^{13}C NMR (126 MHz, $CDCl_3$) δ 167.0, 165.9, 140.5, 138.7, 134.4, 132.3, 131.7, 131.4, 130.5, 129.7, 128.9, 128.1, 127.4, 59.9, 52.7, 50.0. IR (film): ν (cm^{-1}) 3388, 2980, 2972, 1732, 1715, 1699, 1395, 1248, 1147, 1134, 706. HR-MS (ESI) calculated $[M-H]^-$ for $C_{25}H_{22}NO_7S^-$ = 480.11225, found: 480.11262. The enantiomeric excess of **16** was determined by HPLC analysis on Chiralpak OJ-H column. Conditions: hexane/isopropanol = 60/40, flow rate = 1.0 mL/min, uv-vis detection at λ = 210 nm, t_R = 28.5 min (major), 59.1 min (minor).

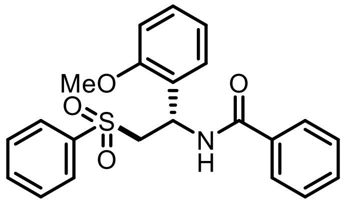

(*S*)-*N*-(1-(2-Methoxyphenyl)-2-(phenylsulfonyl)ethyl)benzamide (17). White solid, 63% yield, 95:5 er, $[\alpha]_D^{25}$ = -14.77 (c = 0.16, $CHCl_3$). ^1H NMR (400 MHz, $CDCl_3$) δ 7.83 – 7.77 (m, 2H), 7.75 – 7.70 (m, 2H), 7.58 – 7.39 (m, 7H), 7.26 – 7.23 (m, 1H), 7.18 (td, *J* = 7.6, 1.7 Hz, 1H), 6.89 (t, *J* = 6.9 Hz, 1H), 6.66 (d, *J* = 8.2 Hz, 1H), 5.68 (td, *J* = 8.0, 4.7 Hz, 1H), 4.02 (dd, *J* = 14.8, 7.8 Hz, 1H), 3.72 (s, 3H), 3.69 (dd, *J* =

14.8, 4.8 Hz, 1H). ^{13}C NMR (101 MHz, $CDCl_3$) δ 166.4, 156.5, 139.2, 134.2, 133.6, 131.9, 129.6, 129.1, 128.8, 128.5, 128.0, 127.2, 125.9, 121.1, 110.8, 58.6, 55.4, 47.3. IR (film): ν (cm^{-1}) 3351, 2930, 1636, 1521, 1489, 1295, 1238, 1140, 1082, 860, 751. HR-MS (ESI) calculated $[M+H]^+$ for $C_{22}H_{22}NO_4S^+$ = 396.12641, found: 396.12638. The enantiomeric excess of **17** was determined by HPLC analysis on Chiralpak AD-H column. Conditions: hexane/isopropanol = 80/20, flow rate = 1.0 mL/min, uv-vis detection at λ = 254 nm, t_R = 23.2 min (minor), 51.8 min (major).

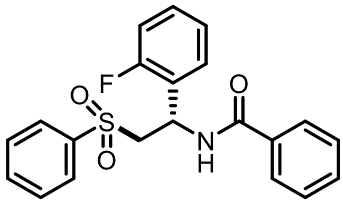

(*S*)-*N*-(1-(2-Fluorophenyl)-2-(phenylsulfonyl)ethyl)benzamide (18). White solid, 58% yield, 95:5 er, $[\alpha]_D^{25}$ = -35.35 (c = 0.13, $CHCl_3$). ^1H NMR (400 MHz, $CDCl_3$) δ 7.87 – 7.82 (m, 2H), 7.81 – 7.75 (m, 2H), 7.61 – 7.52 (m, 2H), 7.51 – 7.42 (m, 5H), 7.35 (td, *J* = 7.7, 2.1 Hz, 1H), 7.25 – 7.17 (m, 1H), 7.09 (td, *J* = 7.6, 1.2 Hz, 1H), 6.88 (ddd, *J* = 11.1, 8.2, 1.2 Hz, 1H), 5.72 (td, *J* = 7.9, 4.4 Hz, 1H), 3.96 (dd, *J* = 14.8, 8.2 Hz, 1H), 3.67 (dd, *J* = 14.8, 4.4 Hz, 1H). ^{13}C NMR (101 MHz, $CDCl_3$) δ 166.6, 160.4 (d, *J* = 245.4 Hz), 138.9, 134.1, 133.6, 132.2, 130.2 (d, *J* = 8.7 Hz), 129.5, 128.9, 128.7 (d, *J* = 4.1 Hz), 128.0, 127.3, 125.6 (d, *J* = 12.8 Hz), 124.7 (d, *J* = 3.2 Hz), 116.0, 115.8, 58.8, 58.8, 46.1. IR (film): ν (cm^{-1}) 3320, 2980, 2360, 1645, 1251, 1154, 1145, 1083, 834, 745. HR-MS (ESI) calculated $[M+H]^+$ for $C_{21}H_{19}FNO_3S^+$ = 384.10642, found: 384.10632. The enantiomeric excess of **18** was determined by HPLC analysis on Chiralpak AD-H column. Conditions: hexane/isopropanol = 80/20, flow rate = 1.0 mL/min, uv-vis detection at λ = 220 nm, t_R = 14.3 min (major), 27.5 min (minor).

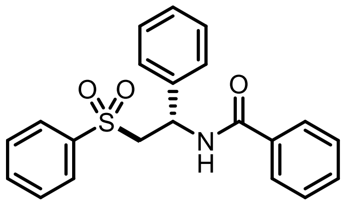

(*S*)-*N*-(1-Phenyl-2-(phenylsulfonyl)ethyl)benzamide (19). White solid, 61% yield,

96:4 er, $[\alpha]_D^{25}$ = -32.46 (c = 0.16, $CHCl_3$). ^1H NMR (400 MHz, $CDCl_3$) δ 7.87 – 7.78 (m, 4H), 7.59 (t, *J* = 7.5 Hz, 1H), 7.55 – 7.40 (m, 6H), 7.28 – 7.19 (m, 5H), 5.48 (ddd, *J* = 9.3, 6.7, 4.1 Hz, 1H), 3.83 (dd, *J* = 14.8, 9.1 Hz, 1H), 3.58 (dd, *J* = 14.8, 4.0 Hz, 1H). ^{13}C NMR (101 MHz, $CDCl_3$) δ 166.8, 139.0, 138.9, 134.1, 133.8, 132.0, 129.6, 129.1, 128.8, 128.2, 128.1, 127.3, 126.3, 60.5, 50.2. IR (film): ν (cm^{-1}) 3251, 2158, 1636, 1142, 736. HR-MS (ESI) calculated $[M+H]^+$ for $C_{21}H_{20}NO_3S^+$ = 366.11584, found: 366.11574. The enantiomeric excess of **19** was determined by HPLC analysis on Chiralpak AD-H column. Conditions: hexane/isopropanol = 80/20, flow rate = 1.0 mL/min, uv-vis detection at λ = 220 nm, t_R = 21.3 min (major), 35.4 min (minor).

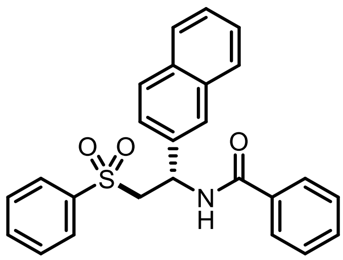

(*S*)-*N*-(1-(Naphthalen-2-yl)-2-(phenylsulfonyl)ethyl)benzamide (20). White solid, 48% yield, 95:5 er, $[\alpha]_D^{25}$ = -46.92 (c = 0.19, $CHCl_3$). ^1H NMR (400 MHz, $CDCl_3$) δ 7.91 – 7.85 (m, 2H), 7.81 – 7.68 (m, 6H), 7.61 – 7.43 (m, 7H), 7.37 (t, *J* = 7.9 Hz, 2H), 7.30 (dd, *J* = 8.6, 1.8 Hz, 1H), 5.65 (ddd, *J* = 8.7, 6.6, 4.1 Hz, 1H), 3.92 (dd, *J* = 14.9, 8.8 Hz, 1H), 3.70 (dd, *J* = 14.8, 4.2 Hz, 1H). ^{13}C NMR (101 MHz, $CDCl_3$) δ 166.8, 138.9, 136.1, 134.0, 133.8, 133.3, 133.1, 132.1, 129.4, 129.1, 128.9, 128.1, 128.1, 127.7, 127.3, 126.6, 126.5, 125.4, 123.9, 60.4, 50.3. IR (film): ν (cm^{-1}) 3320, 2980, 1645, 1532, 1286, 1251, 1146, 1084, 762. HR-MS (ESI) calculated $[M+H]^+$ for $C_{25}H_{22}NO_3S^+$ = 416.13149, found: 416.13165. The enantiomeric excess of **20** was determined by HPLC analysis on Chiralpak AD-H column. Conditions: hexane/isopropanol = 80/20, flow rate = 1.0 mL/min, uv-vis detection at λ = 220 nm, t_R = 23.5 min (major), 54.5 min (minor).

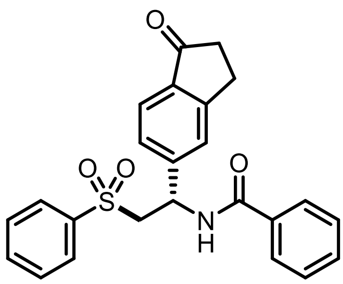

(*S*)-*N*-(1-(4-(Tert-butyl)phenyl)-2-(phenylsulfonyl)ethyl)benzamide (21). White solid, 64% yield, 95:5 er, $[\alpha]_D^{25}$ = -4.08 (c = 0.19, $CHCl_3$). ^{1}H NMR (400 MHz, $CDCl_3$) δ 7.81 (ddd, *J* = 9.8, 8.4, 1.3 Hz, 4H), 7.73 (d, *J* = 6.6 Hz, 1H), 7.65 (d, *J* = 1.9 Hz, 1H), 7.62 – 7.54 (m, 2H), 7.53 – 7.44 (m, 3H), 7.40 (t, *J* = 7.6 Hz, 3H), 5.53 (ddd, *J* = 10.1, 6.5, 4.0 Hz, 1H), 3.85 (dd, *J* = 14.7, 9.3 Hz, 1H), 3.56 (dd, *J* = 14.7, 4.0 Hz, 1H), 3.11 – 3.02 (m, 2H), 2.71 – 2.60 (m, 2H). ^{13}C NMR (101 MHz, $CDCl_3$) δ 206.8, 166.9, 155.1, 139.2, 138.8, 137.7, 134.2, 133.5, 133.4, 132.1, 129.6, 128.8, 128.1, 127.4, 127.3, 121.1, 60.3, 49.9, 36.6, 25.7. IR (film): ν (cm^{-1}) 3351, 2980, 2360, 1715, 1654, 1523, 1137, 711. HR-MS (ESI) calculated $[M+H]^+$ for $C_{24}H_{22}NO_4S^+$ = 420.12641, found: 420.12638. The enantiomeric excess of **21** was determined by HPLC analysis on Chiralpak AD-H column. Conditions: hexane/isopropanol = 70/30, flow rate = 1.0 mL/min, uv-vis detection at λ = 220 nm, t_R = 19.2 min (major), 29.8 min (minor).

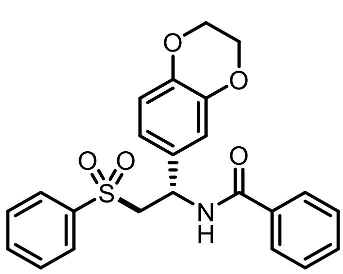

(*S*)-*N*-(1-(2,3-Dihydrobenzo[b][1,4]dioxin-6-yl)-2-(phenylsulfonyl)ethyl)benzamide (22). White solid, 49% yield, 95:5 er, $[\alpha]_D^{25}$ = -9.25 (c = 0.19, $CHCl_3$). ^{1}H NMR (400 MHz, $CDCl_3$) δ 7.81 (ddd, *J* = 5.5, 3.9, 2.2 Hz, 4H), 7.62 – 7.57 (m, 1H), 7.53 – 7.36 (m, 6H), 6.77 – 6.67 (m, 3H), 5.35 (ddd, *J* = 8.9, 6.6, 4.3 Hz, 1H), 4.18 (s, 4H), 3.79 (dd, *J* = 14.8, 8.9 Hz, 1H), 3.55 (dd, *J* = 14.8, 4.3 Hz, 1H). ^{13}C NMR (101 MHz, $CDCl_3$) δ 166.8, 143.8, 143.4, 139.0, 134.0, 133.8, 132.2, 132.0, 129.5, 128.8, 128.1, 127.3, 119.3, 117.8, 115.2, 64.4, 64.4, 60.5, 49.6. IR (film):

ν (cm^{-1}) 3396, 2980, 2971, 2342, 1648, 1540, 1525, 1489, 1084, 842, 712. HR-MS (ESI) calculated $[M+H]^+$ for $C_{23}H_{22}NO_5S^+$ = 424.12132, found: 424.12094. The enantiomeric excess of **22** was determined by HPLC analysis on Chiralpak AD-H column. Conditions: hexane/isopropanol = 70/30, flow rate = 1.0 mL/min, uv-vis detection at λ = 254 nm, t_R = 19.5 min (major), 25.7 min (minor).

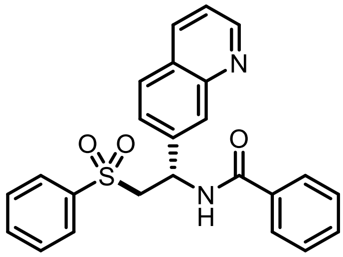

(*S*)-*N*-(2-(Phenylsulfonyl)-1-(quinolin-7-yl)ethyl)benzamide (23). White solid, 40% yield, 94:6 er, $[\alpha]_D^{25}$ = +13.77 (c = 0.16, $CHCl_3$). ^{1}H NMR (500 MHz, $CDCl_3$) δ 8.87 (d, *J* = 4.6 Hz, 1H), 8.11 – 8.04 (m, 1H), 8.02 – 7.95 (m, 1H), 7.88 (t, *J* = 7.4 Hz, 2H), 7.85 – 7.72 (m, 4H), 7.59 – 7.42 (m, 5H), 7.38 (t, *J* = 7.8 Hz, 3H), 5.75 – 5.67 (m, 1H), 3.96 (dd, *J* = 14.8, 8.9 Hz, 1H), 3.68 (dd, *J* = 14.8, 4.3 Hz, 1H). ^{13}C NMR (126 MHz, $CDCl_3$) δ 167.0, 150.6, 147.5, 138.8, 137.3, 136.6, 134.2, 133.5, 132.2, 130.3, 129.5, 128.9, 128.2, 128.0, 127.7, 127.4, 125.5, 121.7, 60.2, 50.1. IR (film): ν (cm^{-1}) 3404, 2976, 2889, 1698, 1660, 1525, 1395, 1387, 1288, 1151, 1137, 954, 706. HR-MS (ESI) calculated $[M+H]^+$ for $C_{24}H_{21}N_2O_3S^+$ = 417.12674, found: 417.12651. The enantiomeric excess of **23** was determined by HPLC analysis on Chiralpak AD-H column. Conditions: hexane/isopropanol = 60/40, flow rate = 1.0 mL/min, uv-vis detection at λ = 220 nm, t_R = 11.9 min (major), 24.5 min (minor).

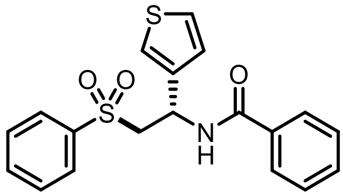

(*S*)-*N*-(2-(Phenylsulfonyl)-1-(thiophen-3-yl)ethyl)benzamide (24). White solid, 55% yield, 93:7 er, $[\alpha]_D^{25}$ = +3.85 (c = 0.23, $CHCl_3$). ^{1}H NMR (400 MHz, $CDCl_3$) δ 7.85 – 7.74 (m, 4H), 7.61 – 7.49 (m, 2H), 7.45 (q, *J* = 7.6 Hz, 4H), 7.42 – 7.35 (m, 1H), 7.19

– 7.13 (m, 2H), 6.87 (dd, *J* = 4.9, 1.6 Hz, 1H), 5.68 (td, *J* = 7.5, 4.7 Hz, 1H), 3.92 (dd, *J* = 14.8, 7.7 Hz, 1H), 3.68 (dd, *J* = 14.8, 4.5 Hz, 1H). ^{13}C NMR (101 MHz, $CDCl_3$) δ 166.8, 139.6, 139.3, 134.0, 133.8, 132.1, 129.5, 128.8, 128.0, 127.3, 127.0, 125.8, 122.2, 59.9, 46.2. IR (film): ν (cm^{-1}) 3388, 2980, 2360, 1715, 1698, 1661, 1524, 1489, 1285, 1139, 1083, 843, 712. HR-MS (ESI) calculated $[M-H]^-$ for $C_{19}H_{16}NO_3S_2^-$ = 370.05771, found: 370.05792. The enantiomeric excess of **24** was determined by HPLC analysis on Chiralpak AD-H column. Conditions: hexane/isopropanol = 80/20, flow rate = 1.0 mL/min, uv-vis detection at λ = 220 nm, t_R = 25.5 min (major), 51.5 min (minor).

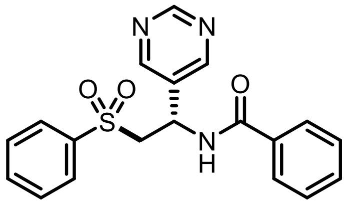

(*S*)-*N*-(2-(Phenylsulfonyl)-1-(pyrimidin-5-yl)ethyl)benzamide (25). White solid, 50% yield, 96:4 er, $[\alpha]_D^{25}$ = -6.68 (c = 0.10, $CHCl_3$). ^{1}H NMR (400 MHz, $CDCl_3$) δ 9.09 (s, 1H), 8.70 (s, 2H), 7.88 – 7.77 (m, 5H), 7.69 – 7.63 (m, 1H), 7.59 – 7.51 (m, 3H), 7.50 – 7.43 (m, 2H), 5.61 – 5.49 (m, 1H), 3.90 (dd, *J* = 14.6, 8.7 Hz, 1H), 3.62 (dd, *J* = 14.7, 4.1 Hz, 1H). ^{13}C NMR (101 MHz, $CDCl_3$) δ 167.2, 158.4, 155.4, 138.5, 134.8, 132.9, 132.6, 129.9, 129.0, 128.0, 127.3, 59.1, 46.8. IR (film): ν (cm^{-1}) 3370, 2980, 1639, 1567, 1415, 1286, 1145, 1085, 850, 782, 747. HR-MS (ESI) calculated $[M+H]^+$ for $C_{19}H_{18}N_3O_3S^+$ = 368.10634, found: 368.10657. The enantiomeric excess of **25** was determined by HPLC analysis on Chiralpak OD-H column. Conditions: hexane/isopropanol = 40/60, flow rate = 1.0 mL/min, uv-vis detection at λ = 220 nm, t_R = 13.9 min (minor), 18.7 min (major).

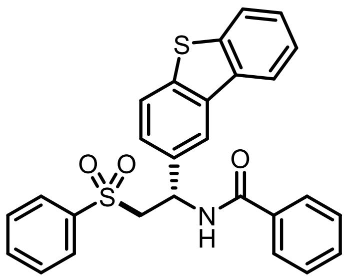

(*S*)-*N*-(1-(Dibenzo[b,d]thiophen-2-yl)-2-(phenylsulfonyl)ethyl)benzamide (26).

White solid, 42% yield, 92:8 er, $[\alpha]_D^{25}$ = -35.1 (c = 0.09, $CHCl_3$). ^1H NMR (400 MHz, $CDCl_3$) δ 8.04 (dd, *J* = 7.7, 1.7 Hz, 1H), 7.99 (d, *J* = 1.9 Hz, 1H), 7.91 – 7.86 (m, 2H), 7.82 (dd, *J* = 6.9, 2.2 Hz, 1H), 7.80 – 7.75 (m, 2H), 7.69 (d, *J* = 8.3 Hz, 1H), 7.65 (d, *J* = 6.5 Hz, 1H), 7.57 – 7.52 (m, 1H), 7.50 – 7.42 (m, 5H), 7.37 (t, *J* = 7.7 Hz, 2H), 7.31 (dd, *J* = 8.3, 1.9 Hz, 1H), 5.67 (ddd, *J* = 8.8, 6.6, 4.3 Hz, 1H), 3.96 (dd, *J* = 14.8, 8.8 Hz, 1H), 3.70 (dd, *J* = 14.9, 4.4 Hz, 1H). ^{13}C NMR (101 MHz, $CDCl_3$) δ 166.9, 140.0, 139.4, 138.9, 136.0, 135.4, 135.1, 134.0, 133.7, 132.1, 129.4, 128.9, 128.0, 127.3, 127.2, 124.8, 124.6, 123.4, 123.0, 121.9, 119.5, 60.7, 50.4. IR (film): ν (cm^{-1}) 3328, 2980, 1734, 1640, 1527, 1394, 1281, 1141, 1084, 824, 734. HR-MS (ESI) calculated $[M+H]^+$ for $C_{27}H_{22}NO_3S_2^+$ = 472.10356, found: 472.10363. The enantiomeric excess of **26** was determined by HPLC analysis on Chiralpak AD-H column. Conditions: hexane/isopropanol = 70/30, flow rate = 1.0 mL/min, uv-vis detection at λ = 220 nm, t_R = 13.8 min (major), 64.1 min (minor).

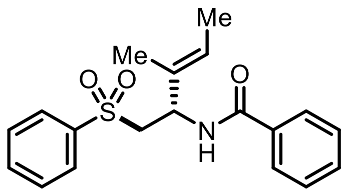

(*S,E*)-*N*-(3-Methyl-1-(phenylsulfonyl)pent-3-en-2-yl)benzamide (27). White solid, 59% yield, 94:6 er, $[\alpha]_D^{25}$ = -4.83 (c = 0.16, $CHCl_3$). ^1H NMR (400 MHz, $CDCl_3$) δ 7.92 – 7.87 (m, 2H), 7.82 (dd, *J* = 8.5, 1.4 Hz, 2H), 7.66 – 7.60 (m, 1H), 7.57 – 7.48 (m, 3H), 7.44 (t, *J* = 7.4 Hz, 2H), 7.19 (d, *J* = 7.3 Hz, 1H), 5.52 (qt, *J* = 6.9, 1.4 Hz, 1H), 4.80 (q, *J* = 3.7 Hz, 1H), 3.62 (dd, *J* = 14.8, 8.2 Hz, 1H), 3.40 (dd, *J* = 14.8, 4.3 Hz, 1H), 1.53 (d, *J* = 6.7 Hz, 3H), 1.47 (s, 3H). ^{13}C NMR (101 MHz, $CDCl_3$) δ 166.6, 139.2, 134.1, 134.0, 131.9, 131.9, 129.4, 128.8, 128.3, 127.2, 122.4, 58.0, 52.4, 13.4, 13.3. IR (film): ν (cm^{-1}) 3343, 2980, 2971, 2342, 1639, 1541, 1384, 1262, 1147, 1082, 955, 711. HR-MS (ESI) calculated $[M+Na]^+$ for $C_{19}H_{21}NO_3SNa^+$ = 366.11344, found: 366.11328. The enantiomeric excess of **27** was determined by HPLC analysis on Chiralpak AD-H column. Conditions: hexane/isopropanol = 90/10, flow rate = 0.8 mL/min, uv-vis detection at λ = 210 nm, t_R = 35.7 min (major), 54.9 min (minor).

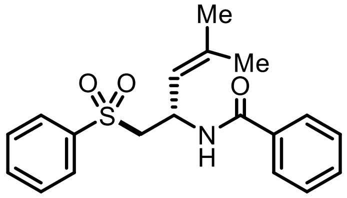

(*S*)-*N*-(4-Methyl-1-(phenylsulfonyl)pent-3-en-2-yl)benzamide (28). Colorless oil, 43% yield, 88:12 er, $[\alpha]_D^{25}$ = -5.62 (c = 0.17, $CHCl_3$). ^{1}H NMR (500 MHz, $CDCl_3$) δ 7.93 – 7.87 (m, 2H), 7.77 – 7.71 (m, 2H), 7.63 – 7.59 (m, 1H), 7.56 – 7.48 (m, 3H), 7.43 (t, *J* = 7.5 Hz, 2H), 6.76 (d, *J* = 6.8 Hz, 1H), 5.38 (dt, *J* = 8.9, 1.4 Hz, 1H), 5.13 (dtd, *J* = 8.9, 6.8, 4.9 Hz, 1H), 3.64 (dd, *J* = 14.5, 6.4 Hz, 1H), 3.44 (dd, *J* = 14.5, 4.9 Hz, 1H), 1.66 (dd, *J* = 7.5, 1.5 Hz, 6H). ^{13}C NMR (126 MHz, $CDCl_3$) δ 166.6, 140.0, 137.6, 134.0, 133.9, 131.8, 129.4, 128.7, 127.9, 127.1, 122.0, 59.4, 45.2, 25.7, 18.3.IR (film): ν (cm^{-1}) 3646, 3328, 2980, 1734, 1638, 1543, 1285, 1141, 1084, 738. HR-MS (ESI) calculated $[M+H]^+$ for $C_{19}H_{22}NO_3S^+$ = 344.13149, found: 344.13138. The enantiomeric excess of **28** was determined by HPLC analysis on Chiralpak IC column. Conditions: hexane/isopropanol = 40/60, flow rate = 1.0 mL/min, uv-vis detection at λ = 220 nm, t_R = 6.0 min (major), 9.0 min (minor).

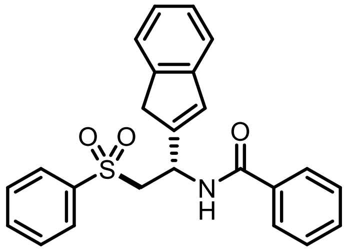

(*S*)-*N*-(1-(1H-Inden-2-yl)-2-(phenylsulfonyl)ethyl)benzamide (29). White solid, 41% yield, 94:6 er, $[\alpha]_D^{25}$ = +53.48 (c = 0.03, $CHCl_3$). ^{1}H NMR (400 MHz, $CDCl_3$) δ 7.88 – 7.80 (m, 4H), 7.57 – 7.45 (m, 4H), 7.42 – 7.37 (m, 3H), 7.26 (s, 2H), 7.24 – 7.19 (m, 1H), 7.13 (td, *J* = 7.3, 1.3 Hz, 1H), 6.74 (d, *J* = 1.6 Hz, 1H), 5.59 – 5.48 (m, 1H), 3.85 (dd, *J* = 14.8, 7.3 Hz, 1H), 3.70 (dd, *J* = 14.8, 4.3 Hz, 1H), 3.32 (d, *J* = 21.4 Hz, 1H), 3.07 (d, *J* = 21.6 Hz, 1H). ^{13}C NMR (101 MHz, $CDCl_3$) δ 166.8, 145.3, 143.7, 142.9, 139.3, 134.0, 133.8, 132.1, 129.4, 129.4, 128.9, 128.1, 127.3, 126.7, 125.1, 123.7, 121.3, 59.1, 46.9, 39.1. IR (film): ν (cm^{-1}) 3661, 3328, 2974, 2874, 1741, 1578, 1489,

1306, 1221, 1085, 856, 775. HR-MS calculated $[M+H]^+$ for $C_{24}H_{22}NO_3S^+$ = 404.13049, found: 404.13174. The enantiomeric excess of **29** was determined by HPLC analysis on Chiralpak AD-H column. Conditions: hexane/isopropanol = 70/30, flow rate = 1.0 mL/min, uv-vis detection at λ = 220 nm, t_R = 17.1 min (minor), 24.9 min (major).

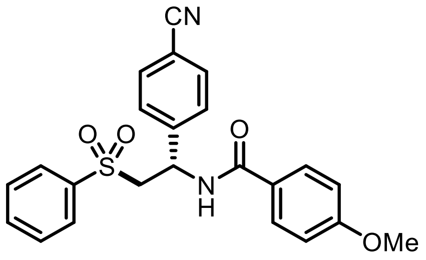

(*S*)-*N*-(1-(4-Cyanophenyl)-2-(phenylsulfonyl)ethyl)-4-methoxybenzamide (30). White solid, 85% yield, 97:3 er, $[\alpha]_D^{25}$ = -50.54 (c = 0.21, $CHCl_3$). ^1H NMR (500 MHz, $CDCl_3$) δ 7.81 (s, 4H), 7.70 (d, *J* = 6.2 Hz, 1H), 7.65 (t, *J* = 7.5 Hz, 1H), 7.57 – 7.48 (m, 4H), 7.38 (d, *J* = 8.3 Hz, 2H), 6.95 (d, *J* = 8.9 Hz, 2H), 5.49 (ddd, *J* = 9.8, 6.4, 3.7 Hz, 1H), 3.87 (s, 3H), 3.78 (dd, *J* = 14.7, 9.0 Hz, 1H), 3.56 (dd, *J* = 14.8, 3.7 Hz, 1H). ^{13}C NMR (126 MHz, $CDCl_3$) δ 166.6, 163.0, 144.6, 138.7, 134.5, 132.8, 129.8, 129.2, 128.0, 127.2, 125.4, 118.5, 114.1, 112.0, 59.9, 55.6, 50.2. IR (film): ν (cm^{-1}) 3358, 2971, 2888, 2360, 2342, 1733, 1506, 1459, 1382, 1253, 1148, 1083, 955, 836. HR-MS (ESI) calculated $[M-H]^-$ for $C_{23}H_{19}N_2O_4S^-$ = 419.10710, found: 419.10719. The enantiomeric excess of **30** was determined by HPLC analysis on Chiralpak AD-H column. Conditions: hexane/isopropanol = 50/50, flow rate = 1.0 mL/min, uv-vis detection at λ = 220 nm, t_R = 13.5 min (major), 43.9 min (minor).

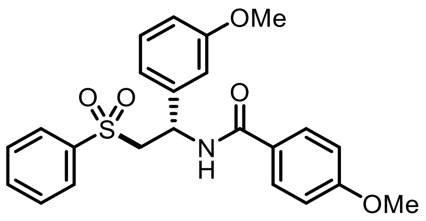

(*S*)-4-methoxy-*N*-(1-(3-methoxyphenyl)-2-(phenylsulfonyl)ethyl)benzamide (31). White solid, 77% yield, 96:4 er, $[\alpha]_D^{25}$ = -7.68 (c = 0.19, $CHCl_3$). ^1H NMR (500 MHz, $CDCl_3$) δ 7.79 (t, *J* = 9.1 Hz, 4H), 7.59 (t, *J* = 7.5 Hz, 1H), 7.47 (t, *J* = 7.8 Hz, 2H), 7.36

(d, J = 6.5 Hz, 1H), 7.16 (t, J = 7.9 Hz, 1H), 6.92 (d, J = 8.7 Hz, 2H), 6.81 (d, J = 7.6 Hz, 1H), 6.77 – 6.71 (m, 2H), 5.45 – 5.38 (m, 1H), 3.85 (s, 3H), 3.81 (dd, J = 15.1, 8.9 Hz, 1H), 3.72 (s, 3H), 3.58 (dd, J = 14.9, 4.2 Hz, 1H). ^{13}C NMR (126 MHz, $CDCl_3$) δ 166.4, 162.6, 160.0, 140.8, 138.9, 134.1, 130.1, 129.5, 129.2, 128.1, 126.1, 118.5, 114.0, 113.3, 112.3, 60.5, 55.6, 55.3, 50.1. IR (film): ν (cm^{-1}) 3304, 2980, 2972, 2360, 2333, 1508, 1392, 1250, 1155, 1144, 1071, 955, 792. HR-MS (ESI) calculated $[M+H]^+$ for $C_{23}H_{24}NO_5S^+$ = 426.13697, found: 426.13648. The enantiomeric excess of **31** was determined by HPLC analysis on Chiralpak AD-H column. Conditions: hexane/isopropanol = 70/30, flow rate = 1.0 mL/min, uv-vis detection at λ = 254 nm, t_R = 22.9 min (major), 30.9 min (minor).

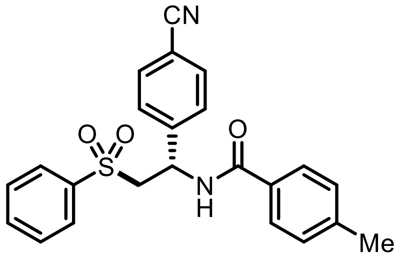

(*S*)-*N*-(1-(4-Cyanophenyl)-2-(phenylsulfonyl)ethyl)-4-methylbenzamide (32). White solid, 69% yield, 96:4 er, $[\alpha]_D^{25}$ = -39.10 (c = 0.19, $CHCl_3$). ^{1}H NMR (400 MHz, $CDCl_3$) δ 7.81 (d, J = 7.2 Hz, 2H), 7.75 (d, J = 8.2 Hz, 3H), 7.65 (t, J = 7.5 Hz, 1H), 7.57 – 7.48 (m, 4H), 7.38 (d, J = 8.4 Hz, 2H), 7.28 – 7.25 (m, 2H), 5.50 (ddd, J = 9.5, 6.0, 3.9 Hz, 1H), 3.79 (dd, J = 14.7, 9.0 Hz, 1H), 3.57 (dd, J = 14.7, 3.9 Hz, 1H), 2.42 (s, 3H). ^{13}C NMR (126 MHz, $CDCl_3$) δ 166.9, 144.5, 143.0, 138.7, 134.5, 132.8, 130.3, 129.7, 129.6, 128.0, 127.3, 127.2, 118.5, 112.1, 59.8, 50.1, 21.7. IR (film): ν (cm^{-1}) 3320, 2980, 2972, 2342, 2331, 1542, 1393, 1383, 1250, 1146, 1085, 954, 743. HR-MS (ESI) calculated $[M+H]^+$ for $C_{23}H_{21}N_2O_3S^+$ = 405.12674, found: 405.12678. The enantiomeric excess of **32** was determined by HPLC analysis on Chiralpak AD-H column. Conditions: hexane/isopropanol = 60/40, flow rate = 1.0 mL/min, uv-vis detection at λ = 210 nm, t_R = 15.1 min (major), 44.3 min (minor).

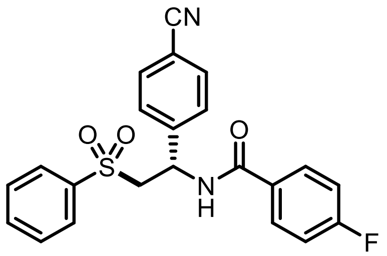

(*S*)-*N*-(1-(4-Cyanophenyl)-2-(phenylsulfonyl)ethyl)-4-fluorobenzamide (33). White solid, 67% yield, 94:6 er, $[\alpha]_D^{25}$ = -25.07 (c = 0.19, $CHCl_3$). ^{1}H NMR (500 MHz, $CDCl_3$) δ 7.93 – 7.86 (m, 2H), 7.82 (d, *J* = 8.1 Hz, 2H), 7.76 (d, *J* = 6.0 Hz, 1H), 7.66 (t, *J* = 7.4 Hz, 1H), 7.55 (dd, *J* = 15.3, 8.1 Hz, 4H), 7.39 (d, *J* = 8.3 Hz, 2H), 7.16 (t, *J* = 8.6 Hz, 2H), 5.51 (dt, *J* = 9.7, 4.9 Hz, 1H), 3.77 (dd, *J* = 14.7, 9.1 Hz, 1H), 3.57 (dd, *J* = 14.7, 3.8 Hz, 1H). ^{13}C NMR (126 MHz, $CDCl_3$) δ 166.0, 165.4 (d, *J* = 253.3 Hz), 144.3, 138.6, 134.6, 132.9, 129.8, 129.8 (d, *J* = 8.9 Hz), 129.4 (d, *J* = 3.2 Hz), 128.0, 127.1, 118.4, 116.1 (d, *J* = 22.0 Hz), 112.2, 59.8, 50.3. ^{19}F NMR (377 MHz, $CDCl_3$) δ -106.69. IR (film): ν (cm^{-1}) 3368, 2360, 2227, 1639, 1505, 1330, 1293, 1144, 848, 724. HR-MS (ESI) calculated $[M+H]^+$ for $C_{22}H_{18}FN_2O_3S^+$ = 409.10167, found: 409.10185. The enantiomeric excess of **33** was determined by HPLC analysis on Chiralpak AD-H column. Conditions: hexane/isopropanol = 60/40, flow rate = 1.0 mL/min, uv-vis detection at λ = 220 nm, t_R = 13.2 min (major), 27.6 min (minor).

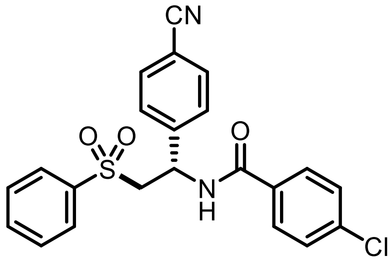

(*S*)-4-Chloro-*N*-(1-(4-cyanophenyl)-2-(phenylsulfonyl)ethyl)benzamide (34). White solid, 92% yield, 96:4 er, $[\alpha]_D^{25}$ = -36.32 (c = 0.17, $CHCl_3$ ^{1}H NMR (400 MHz, $CDCl_3$) δ 7.86 – 7.75 (m, 5H), 7.70 – 7.64 (m, 1H), 7.55 (t, *J* = 7.9 Hz, 4H), 7.43 (d, *J* = 8.5 Hz, 2H), 7.38 (d, *J* = 8.3 Hz, 2H), 5.51 (ddd, *J* = 9.6, 6.1, 3.7 Hz, 1H), 3.79 (dd, *J* = 14.7, 9.2 Hz, 1H), 3.56 (dd, *J* = 14.7, 3.8 Hz, 1H). ^{13}C NMR (126 MHz, $CDCl_3$) δ 166.0, 144.2, 138.8, 138.6, 134.6, 132.9, 131.5, 129.8, 129.2, 128.8, 128.0, 127.2, 118.4,

112.2, 59.7, 50.3. IR (film): ν (cm^{-1}) 3367, 2926, 2360, 1719, 1638, 1542, 1327, 1069, 755. HR-MS (ESI) calculated [M+H]$^+$ for $C_{22}H_{18}ClN_2O_3S^+$ = 425.07212, found: 425.07185. The enantiomeric excess of **34** was determined by HPLC analysis on Chiralpak AD-H column. Conditions: hexane/isopropanol = 60/40, flow rate = 1.0 mL/min, uv-vis detection at λ = 220 nm, t_R = 18.1 min (major), 38.1 min (minor).

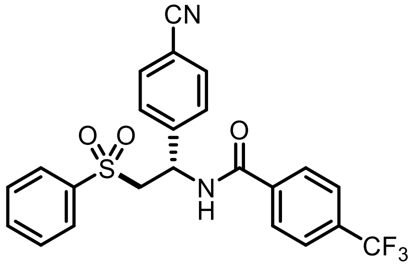

(*S*)-*N*-(1-(4-Cyanophenyl)-2-(phenylsulfonyl)ethyl)-4-(trifluoromethyl)benzamide (35). White solid, 45% yield, 95:5 er, $[\alpha]_D^{25}$ = -13.97 (c = 0.16, $CHCl_3$). ^{1}H NMR (500 MHz, $CDCl_3$) δ 7.99 (d, *J* = 8.1 Hz, 2H), 7.88 (d, *J* = 6.2 Hz, 1H), 7.83 (d, *J* = 7.9 Hz, 2H), 7.74 (d, *J* = 8.1 Hz, 2H), 7.68 (t, *J* = 7.5 Hz, 1H), 7.62 – 7.51 (m, 4H), 7.40 (d, *J* = 8.3 Hz, 2H), 5.54 (tt, *J* = 7.3, 3.4 Hz, 1H), 3.78 (dd, *J* = 14.8, 9.3 Hz, 1H), 3.57 (dd, *J* = 14.3, 3.1 Hz, 1H). ^{13}C NMR (126 MHz, $CDCl_3$) δ 165.8, 144.0, 138.6, 136.4, 134.7, 134.1 (q, *J* = 32.9 Hz), 132.9, 129.9, 128.0, 127.8, 127.1, 126.0 (q, *J* = 3.6 Hz), 123.7 (q, *J* = 272.6 Hz), 118.3, 112.4, 59.6, 50.4. IR (film): ν (cm^{-1}) 3268, 2360, 2341, 1641, 1331, 1146, 1061, 853, 729. HR-MS (ESI) calculated [M-H]$^-$ for $C_{23}H_{16}F_3N_2O_3S^-$ = 457.08392, found: 457.08428. The enantiomeric excess of **35** was determined by HPLC analysis on Chiralpak AD-H column. Conditions: hexane/isopropanol = 60/40, flow rate = 1.0 mL/min, uv-vis detection at λ = 220 nm, t_R = 18,8 min (major), 40.6 min (minor).

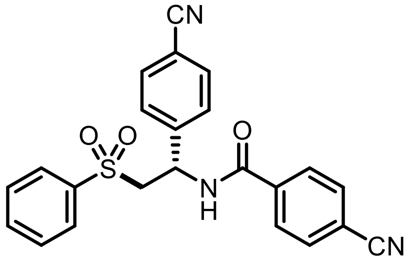

(*S*)-4-Cyano-*N*-(1-(4-cyanophenyl)-2-(phenylsulfonyl)ethyl)benzamide (36). White solid, 79% yield, 95:5 er, $[\alpha]_D^{25}$ = -41.36 (c = 0.21, $CHCl_3$). ^{1}H NMR (400 MHz, $CDCl_3$) δ 8.01 – 7.95 (m, 2H), 7.91 (d, *J* = 6.0 Hz, 1H), 7.86 – 7.81 (m, 2H), 7.80 – 7.75 (m, 2H), 7.73 – 7.65 (m, 1H), 7.62 – 7.52 (m, 4H), 7.40 (d, *J* = 8.1 Hz, 2H), 5.55 (ddd, *J* = 9.5, 5.9, 3.7 Hz, 1H), 3.78 (dd, *J* = 14.6, 9.5 Hz, 1H), 3.55 (dd, *J* = 14.6, 3.8 Hz, 1H). ^{13}C NMR (101 MHz, $CDCl_3$) δ 165.3, 143.9, 138.5, 137.1, 134.8, 132.9, 132.8, 129.9, 128.1, 128.0, 127.2, 118.3, 118.0, 115.9, 112.4, 59.5, 50.4. IR (film): ν (cm^{-1}) 2980, 2972, 2360, 2342, 1733, 1541, 1472, 1457, 1251, 1146, 1083, 1071, 954, 762. HR-MS (ESI) calculated $[M+H]^+$ for $C_{23}H_{18}N_3O_3S^+$ = 416.10634, found: 416.10633. The enantiomeric excess of **36** was determined by HPLC analysis on Chiralpak AD-H column. Conditions: hexane/isopropanol = 50/50, flow rate = 1.0 mL/min, uv-vis detection at λ = 210 nm, t_R = 18.8 min (major), 32.2 min (minor).

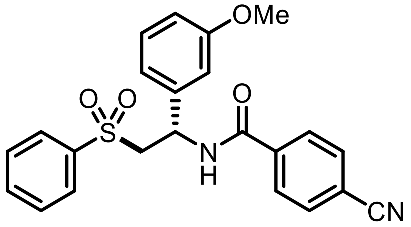

(*S*)-4-Cyano-*N*-(1-(3-methoxyphenyl)-2-(phenylsulfonyl)ethyl)benzamide (37). White solid, 63% yield, 97:3 er, $[\alpha]_D^{25}$ = -35.56 (c = 0.20, $CHCl_3$). ^{1}H NMR (500 MHz, $CDCl_3$) δ 7.96 (d, *J* = 8.3 Hz, 2H), 7.82 (d, *J* = 8.6 Hz, 2H), 7.75 (d, *J* = 8.3 Hz, 2H), 7.64 (t, *J* = 7.5 Hz, 1H), 7.53 (dt, *J* = 15.7, 7.2 Hz, 3H), 7.20 (t, *J* = 7.9 Hz, 1H), 6.82 (d, *J* = 7.2 Hz, 1H), 6.76 (d, *J* = 7.9 Hz, 2H), 5.47 (dq, *J* = 10.2, 4.2 Hz, 1H), 3.78 (dd, *J* = 14.8, 9.4 Hz, 1H), 3.74 (s, 3H), 3.57 (dd, *J* = 14.8, 3.9 Hz, 1H). ^{13}C NMR (126 MHz, $CDCl_3$) δ 165.1, 160.2, 140.1, 138.8, 137.7, 134.4, 132.7, 130.3, 129.7, 128.1, 118.3, 118.1, 115.6, 113.4, 112.5, 60.2, 55.4, 50.4. IR (film): ν (cm^{-1}) 3320, 1639, 1289, 1257, 1046, 848, 809, 781. HR-MS (ESI) calculated $[M+H]^+$ for $C_{23}H_{21}N_2O_4S^+$ = 421.12165, found: 421.12165. The enantiomeric excess of **37** was determined by HPLC analysis on Chiralpak IC column. Conditions: hexane/isopropanol = 50/50, flow rate = 1.0 mL/min, uv-vis detection at λ = 220 nm, t_R = 42.0 min (major), 73.2 min (minor).

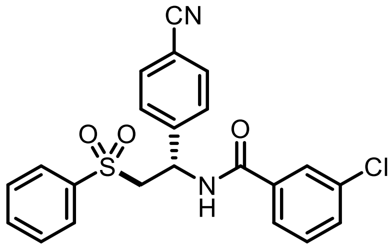

(*S*)-3-Chloro-*N*-(1-(4-cyanophenyl)-2-(phenylsulfonyl)ethyl)benzamide (38). White solid, 72% yield, 95:5 er, $[\alpha]_D^{25}$ = -20.53 (c = 0.16, $CHCl_3$). ^1H NMR (400 MHz, $CDCl_3$) δ 7.84 (d, *J* = 7.2 Hz, 2H), 7.81 – 7.76 (m, 2H), 7.73 (dt, *J* = 7.8, 1.4 Hz, 1H), 7.68 – 7.63 (m, 1H), 7.53 (dt, *J* = 13.4, 7.1 Hz, 5H), 7.41 – 7.35 (m, 3H), 5.53 (ddd, *J* = 9.7, 6.3, 3.7 Hz, 1H), 3.84 (dd, *J* = 14.9, 9.5 Hz, 1H), 3.56 (dd, *J* = 14.7, 3.8 Hz, 1H). ^{13}C NMR (126 MHz, $CDCl_3$) δ 165.7, 144.2, 138.6, 135.1, 135.0, 134.6, 132.9, 132.4, 130.2, 129.8, 128.1, 127.7, 127.2, 125.3, 118.4, 112.2, 59.6, 50.2. IR (film): ν (cm^{-1}) 3354, 3176, 2922, 2360, 2230, 1568, 1536, 1429, 1122, 1086, 757. HR-MS (ESI) calculated $[M+H]^+$ for $C_{22}H_{18}ClN_2O_3S^+$ = 425.07212, found: 425.07238. The enantiomeric excess of **38** was determined by HPLC analysis on Chiralpak AD-H column. Conditions: hexane/isopropanol = 60/40, flow rate = 1.0 mL/min, uv-vis detection at λ = 220 nm, t_R = 18.1 min (major), 38.1 min (minor).

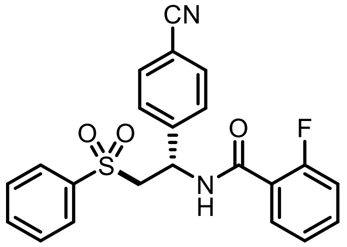

(*S*)-*N*-(1-(4-Cyanophenyl)-2-(phenylsulfonyl)ethyl)-2-fluorobenzamide (39). White solid, 75% yield, 95:5 er, $[\alpha]_D^{25}$ = +20.93 (c = 0.20, $CHCl_3$). ^1H NMR (500 MHz, $CDCl_3$) δ 7.99 (tt, *J* = 13.0, 6.2 Hz, 2H), 7.81 (d, *J* = 8.3 Hz, 2H), 7.59 (dd, *J* = 15.7, 7.8 Hz, 3H), 7.49 (q, *J* = 7.6 Hz, 3H), 7.42 (d, *J* = 8.3 Hz, 2H), 7.25 (d, *J* = 5.2 Hz, 1H), 7.17 (dd, *J* = 12.3, 8.2 Hz, 1H), 5.64 (dq, *J* = 10.0, 5.6, 4.8 Hz, 1H), 3.83 (dd, *J* = 14.8, 8.6 Hz, 1H), 3.59 (dd, *J* = 14.8, 4.1 Hz, 1H). ^{13}C NMR (126 MHz, $CDCl_3$) δ 163.0 (d, *J* = 3.1 Hz), 161.1 (d, *J* = 248.2 Hz), 144.2, 139.0, 134.3, 134.2 (d, *J* = 9.4 Hz), 132.8,

132.2 (d, *J* = 1.9 Hz), 129.6, 128.0, 127.3, 125.1 (d, *J* = 3.1 Hz), 120.0 (d, *J* = 11.1 Hz), 118.4, 116.4 (d, *J* = 24.6 Hz), 112.2, 60.0, 50.1. ^{19}F NMR (377 MHz, $CDCl_3$) δ -112.5. IR (film): ν (cm^{-1}) 3267, 2361, 2230, 1655, 1483, 1450, 1334, 1220, 1086, 890, 792. HR-MS (ESI) calculated $[M+H]^+$ for $C_{22}H_{18}FN_2O_3S^+$ = 409.10167, found: 409.10187. The enantiomeric excess of **39** was determined by HPLC analysis on Chiralpak AD-H column. Conditions: hexane/isopropanol = 60/40, flow rate = 1.0 mL/min, uv-vis detection at λ = 210 nm, t_R = 14.4 min (major), 35.3 min (minor).

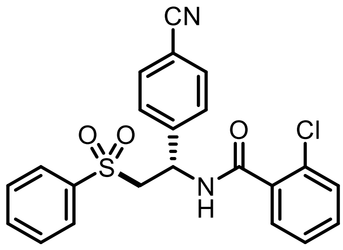

(*S*)-2-Chloro-*N*-(1-(4-cyanophenyl)-2-(phenylsulfonyl)ethyl)benzamide (40). White solid, 60% yield, 96:4 er, $[\alpha]_D^{25}$ = -3.00 (c = 0.18, $CHCl_3$). ^{1}H NMR (500 MHz, $CDCl_3$) δ 7.81 (d, *J* = 7.8 Hz, 2H), 7.71 (d, *J* = 7.6 Hz, 1H), 7.64 (d, *J* = 7.5 Hz, 1H), 7.59 (d, *J* = 8.1 Hz, 3H), 7.52 (t, *J* = 7.7 Hz, 2H), 7.43 (t, *J* = 9.4 Hz, 4H), 7.36 (t, *J* = 7.3 Hz, 2H), 5.62 (q, *J* = 6.5, 6.0 Hz, 1H), 3.82 (dd, *J* = 14.8, 8.5 Hz, 1H), 3.60 (dd, *J* = 14.8, 4.2 Hz, 1H). ^{13}C NMR (126 MHz, $CDCl_3$) δ 166.1, 138.9, 134.4, 133.8, 132.8, 132.1, 131.1, 130.6, 130.5, 129.7, 128.7, 128.0, 127.4, 127.4, 118.4, 112.3, 59.9, 50.2. IR (film): ν (cm^{-1}) 3295, 2922, 2360, 1592, 1455, 1433, 1309, 1127, 1037, 857, 787. HR-MS (ESI) calculated $[M+H]^+$ for $C_{22}H_{18}ClN_2O_3S^+$ = 425.07212, found: 425.07218. The enantiomeric excess of **40** was determined by HPLC analysis on Chiralpak AD-H column. Conditions: hexane/isopropanol = 60/40, flow rate = 1.0 mL/min, uv-vis detection at λ = 210 nm, t_R = 13.8 min (major), 20.0 min (minor).

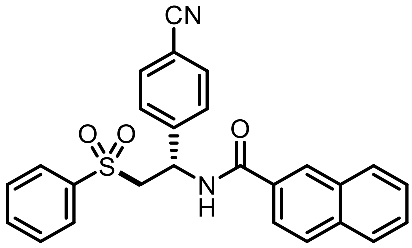

(*S*)-*N*-(1-(4-Cyanophenyl)-2-(phenylsulfonyl)ethyl)-2-naphthamide (41). White solid, 63% yield, 97:3 er, $[\alpha]_D^{25}$ = -20.53 (c = 0.20, $CHCl_3$). ^{1}H NMR (500 MHz, $CDCl_3$) δ 8.38 (s, 1H), 7.93 (dt, *J* = 26.4, 7.9 Hz, 5H), 7.84 (d, *J* = 7.8 Hz, 2H), 7.57 (tt, *J* = 30.0, 7.6 Hz, 7H), 7.43 (d, *J* = 8.0 Hz, 2H), 5.58 (dt, *J* = 9.6, 5.0 Hz, 1H), 3.85 (dd, *J* = 14.8, 9.0 Hz, 1H), 3.62 (dd, *J* = 14.9, 3.9 Hz, 1H). ^{13}C NMR (126 MHz, $CDCl_3$) δ 167.1, 144.4, 138.7, 135.2, 134.6, 132.8, 132.7, 130.3, 129.8, 129.3, 128.9, 128.2, 128.2, 128.1, 128.0, 127.2, 127.1, 123.5, 118.5, 112.2, 59.9, 50.3. IR (film): ν (cm^{-1}) 3315, 2227, 1639, 1527, 1294, 1142, 825, 778. HR-MS (ESI) calculated $[M+H]^+$ for $C_{26}H_{21}N_2O_3S^+$ = 441.12674, found: 441.12686. The enantiomeric excess of **41** was determined by HPLC analysis on Chiralpak AD-H column. Conditions: hexane/isopropanol = 40/60, flow rate = 1.0 mL/min, uv-vis detection at λ = 220 nm, t_R = 14.4 min (major), 46.6 min (minor).

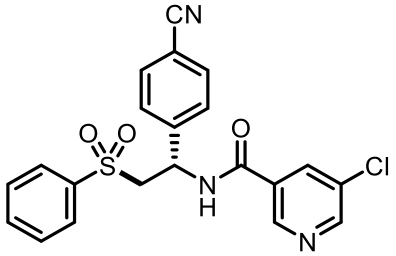

(*S*)-5-Chloro-*N*-(1-(4-cyanophenyl)-2-(phenylsulfonyl)ethyl)nicotinamide (42). White solid, 60% yield yield, 93:7 er, $[\alpha]_D^{25}$ = +12.55 (c = 0.08, $CHCl_3$). ^{1}H NMR (400 MHz, CD_3CN) δ 8.47 (dd, *J* = 4.9, 1.0 Hz, 1H), 7.88 – 7.84 (m, 2H), 7.72 (d, *J* = 8.1 Hz, 1H), 7.69 – 7.65 (m, 2H), 7.65 – 7.61 (m, 1H), 7.57 – 7.46 (m, 6H), 5.59 (ddd, *J* = 10.0, 8.0, 3.8 Hz, 1H), 3.96 (dd, *J* = 14.9, 10.1 Hz, 1H), 3.69 (dd, *J* = 14.9, 3.8 Hz, 1H). ^{13}C NMR (101 MHz, CD_3CN) δ 164.7, 153.0, 152.0, 146.1, 145.3, 140.7, 135.5, 134.0, 130.9, 129.4, 129.2, 123.4, 121.8, 119.7, 113.0, 59.8, 50.9. IR (film): ν (cm^{-1})

3318, 2980, 1732, 1645, 1521, 1473, 1382, 1250, 1142, 954, 785. HR-MS (ESI) calculated $[M+H]^+$ for $C_{21}H_{17}ClN_3O_3S^+$ = 426.06737, found: 426.06754. The enantiomeric excess of **42** was determined by HPLC analysis on Chiralpak AD-H column. Conditions: hexane/isopropanol = 50/50, flow rate = 1.0 mL/min, uv-vis detection at λ = 220 nm, t_R = 8.3 min (major), 15.8 min (minor).

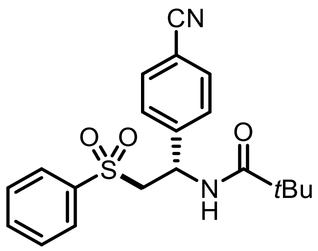

(*S*)-*N*-(1-(4-Cyanophenyl)-2-(phenylsulfonyl)ethyl)pivalamide (43). White solid, 62% yield, 97:3 er, $[\alpha]_D^{25}$ = -10.48 (c = 0.22, $CHCl_3$). ^1H NMR (400 MHz, $CDCl_3$) δ 7.83 – 7.75 (m, 2H), 7.72 – 7.64 (m, 1H), 7.54 (dd, *J* = 8.5, 6.9 Hz, 4H), 7.32 – 7.28 (m, 2H), 7.23 (d, *J* = 5.9 Hz, 1H), 5.29 (ddd, *J* = 9.4, 5.8, 3.9 Hz, 1H), 3.64 (dd, *J* = 14.7, 9.1 Hz, 1H), 3.45 (dd, *J* = 14.6, 3.9 Hz, 1H), 1.26 (s, 9H). ^{13}C NMR (101 MHz, $CDCl_3$) δ 178.6, 144.8, 138.8, 134.5, 132.8, 129.7, 128.0, 126.9, 118.5, 112.0, 59.8, 49.7, 39.0, 27.5. IR (film): ν (cm^{-1}) 3403, 2928, 1655, 1265, 1143, 1085, 841, 702. HR-MS (ESI) calculated $[M+Cl]^-$ for $C_{20}H_{22}ClN_2O_3S^-$ = 405.10451, found: 405.10473. The enantiomeric excess of **43** was determined by HPLC analysis on Chiralpak AD-H column. Conditions: hexane/isopropanol = 60/40, flow rate = 1.0 mL/min, uv-vis detection at λ = 220 nm, t_R = 7.4 min (major), 14.7 min (minor).

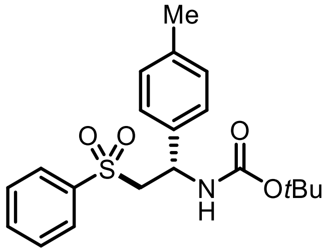

Tert-butyl (*S*)-(2-(phenylsulfonyl)-1-(p-tolyl)ethyl)carbamate (44). White solid, 45% yield, 95:5 er, $[\alpha]_D^{25}$ = +8.49 (c = 0.10, $CHCl_3$). ^1H NMR (400 MHz, $CDCl_3$) δ 7.84 –

7.76 (m, 2H), 7.60 (d, *J* = 7.6 Hz, 1H), 7.53 – 7.45 (m, 2H), 7.06 (s, 4H), 5.38 (s, 1H), 5.03 – 4.94 (m, 1H), 3.65 (s, 1H), 3.47 (dd, *J* = 14.6, 4.7 Hz, 1H), 2.29 (s, 3H), 1.41 (s, 9H). ^{13}C NMR (126 MHz, $CDCl_3$) δ 154.8, 139.4, 137.9, 136.7, 133.8, 129.6, 129.4, 128.2, 126.2, 61.1, 29.9, 28.5, 21.2. IR (film): ν (cm^{-1}) 3380, 2980, 2359, 1734, 1653, 1522, 1380, 1259, 1140, 957, 787. HR-MS (ESI) calculated $[M+Na]^+$ for $C_{20}H_{25}NO_4SNa^+$ = 398.13965, found: 398.13960. The enantiomeric excess of **44** was determined by HPLC analysis on Chiralpak AD-H column. Conditions: hexane/isopropanol = 60/40, flow rate = 1.0 mL/min, uv-vis detection at λ = 220 nm, t_R = 6.8 min (major), 9.3 min (minor).

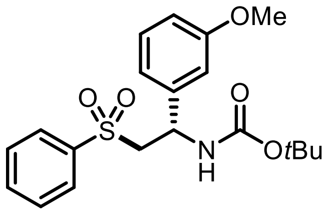

Tert-butyl (*S*)-(1-(3-methoxyphenyl)-2-(phenylsulfonyl)ethyl)carbamate (45). White solid, 40% yield, 94:6 er, $[\alpha]_D^{25}$ = +18.27 (c = 0.10, $CHCl_3$). ^{1}H NMR (400 MHz, $CDCl_3$) δ 7.84 – 7.78 (m, 2H), 7.64 – 7.58 (m, 1H), 7.50 (ddt, *J* = 9.1, 7.6, 1.8 Hz, 2H), 7.17 (t, *J* = 7.9 Hz, 1H), 6.77 – 6.72 (m, 2H), 6.71 – 6.67 (m, 1H), 5.44 (s, 1H), 5.00 (ddd, *J* = 8.8, 6.9, 4.5 Hz, 1H), 3.74 (s, 3H), 3.64 (dd, *J* = 14.5, 9.4 Hz, 1H), 3.48 (dd, *J* = 14.6, 4.5 Hz, 1H), 1.41 (s, 9H). ^{13}C NMR (101 MHz, $CDCl_3$) δ 160.0, 139.3, 133.9, 130.1, 129.4, 128.1, 118.4, 113.5, 112.0, 61.0, 55.4, 28.4, 28.4. IR (film): ν (cm^{-1}) 3382, 3067, 2980, 1687, 1519, 1301, 1259, 1142, 1020, 866, 740. HR-MS calculated $[M+Na]^+$ for $C_{20}H_{25}NO_5SNa^+$ = 414.13456, found: 414.13471. The enantiomeric excess of **45** was determined by HPLC analysis on Chiralpak AD-H column. Conditions: hexane/isopropanol = 40/60, flow rate = 1.0 mL/min, uv-vis detection at λ = 220 nm, t_R = 6.2 min (major), 8.0 min (minor).

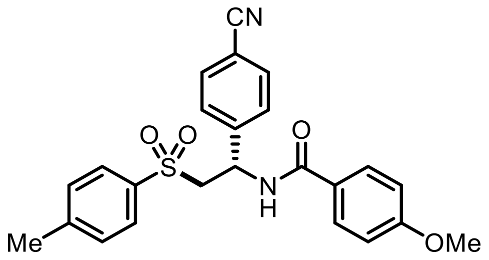

(*S*)-*N*-(1-(4-Cyanophenyl)-2-tosylethyl)-4-methoxybenzamide (46). White solid, 83% yield, 96:4 er, $[\alpha]_D^{25}$ = -43.00 (c = 0.20, $CHCl_3$). ^1H NMR (400 MHz, $CDCl_3$) δ 7.83 (d, *J* = 8.9 Hz, 2H), 7.71 – 7.64 (m, 3H), 7.56 (d, *J* = 8.4 Hz, 2H), 7.37 (d, *J* = 8.3 Hz, 2H), 7.30 (d, *J* = 8.0 Hz, 2H), 6.97 (d, *J* = 8.8 Hz, 2H), 5.41 (dt, *J* = 9.2, 4.5 Hz, 1H), 3.88 (s, 3H), 3.70 (dd, *J* = 14.7, 9.1 Hz, 1H), 3.53 (dd, *J* = 14.7, 3.9 Hz, 1H), 2.43 (s, 3H). ^{13}C NMR (101 MHz, $CDCl_3$) δ 166.5, 163.0, 145.9, 144.7, 135.6, 132.8, 130.4, 129.2, 128.1, 127.1, 125.4, 118.5, 114.2, 112.0, 60.0, 55.6, 50.3, 21.8. IR (film): ν (cm^{-1}) 3305, 1629, 1607, 1506, 1259, 1139, 1029, 848, 770. HR-MS (ESI) calculated $[M+Cl]^-$ for $C_{24}H_{22}ClN_2O_4S^-$ = 469.09943, found: 469.09967. The enantiomeric excess of **46** was determined by HPLC analysis on Chiralpak AD-H column. Conditions: hexane/isopropanol = 60/40, flow rate = 1.0 mL/min, uv-vis detection at λ = 254 nm, t_R = 17.0 min (major), 55.9 min (minor).

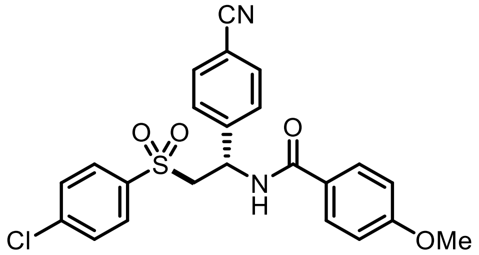

(*S*)-*N*-(2-((4-Chlorophenyl)sulfonyl)-1-(4-cyanophenyl)ethyl)-4-methoxybenzamide (47). White solid, 51% yield, 99:1 er, $[\alpha]_D^{25}$ = -20.37 (c = 0.19, $CHCl_3$). ^1H NMR (400 MHz, $CDCl_3$) δ 7.80 – 7.73 (m, 4H), 7.61 – 7.56 (m, 2H), 7.53 – 7.46 (m, 3H), 7.42 – 7.37 (m, 2H), 6.98 – 6.92 (m, 2H), 5.50 (ddd, *J* = 9.6, 6.2, 3.9 Hz, 1H), 3.87 (s, 3H), 3.80 (dd, *J* = 14.8, 9.2 Hz, 1H), 3.55 (dd, *J* = 14.7, 4.0 Hz, 1H). ^{13}C NMR (126 MHz, $CDCl_3$) δ 166.5, 163.0, 144.4, 141.6, 137.1, 132.9, 130.1, 129.6, 129.2, 127.2, 125.3, 118.4, 114.2, 112.2, 59.9, 55.6, 50.1. IR (film): ν (cm^{-1}) 3338, 2925,

2228, 1605, 1535, 1330, 1111, 1068, 749. HR-MS (ESI) calculated $[M+Cl]^-$ for $C_{23}H_{19}Cl_2N_2O_4S^-$ = 489.04481, found: 489.04495. The enantiomeric excess of **47** was determined by HPLC analysis on Chiralpak AD-H column. Conditions: hexane/isopropanol = 40/60, flow rate = 1.0 mL/min, uv-vis detection at λ = 254 nm, t_R = 9.8 min (major), 45.4 min (minor).

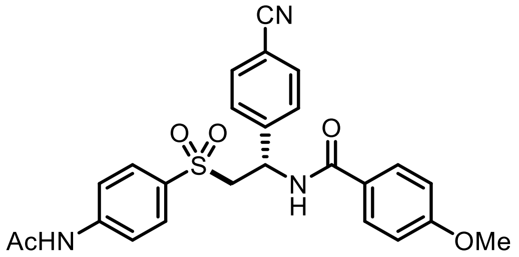

(*S*)-*N*-(2-((4-Acetamidophenyl)sulfonyl)-1-(4-cyanophenyl)ethyl)-4-methoxybenzamide (48). White solid, 42% yield, 95:5 er, $[\alpha]_D^{25}$ = -6.28 (c = 0.14, $CHCl_3$). ^1H NMR (400 MHz, CD_3CN) δ 8.56 (s, 1H), 7.76 – 7.71 (m, 2H), 7.68 – 7.61 (m, 4H), 7.60 – 7.55 (m, 2H), 7.50 – 7.47 (m, 2H), 7.32 (d, *J* = 8.0 Hz, 1H), 6.94 – 6.89 (m, 2H), 5.53 (ddd, *J* = 10.8, 8.0, 3.7 Hz, 1H), 3.92 (dd, *J* = 14.9, 10.2 Hz, 1H), 3.83 (s, 3H), 3.61 (dd, *J* = 14.9, 3.5 Hz, 1H), 2.07 (s, 3H). ^{13}C NMR (101 MHz, CD_3CN) δ 170.5, 166.9, 163.8, 147.4, 145.6, 134.2, 133.9, 130.7, 130.3, 129.0, 127.1, 120.1, 119.8, 114.9, 112.6, 60.4, 56.6, 50.8, 24.9. IR (film): ν (cm^{-1}) 3358, 2972, 2932, 2360, 2342, 1732, 1653, 1506, 1499, 1383, 1253, 1148, 1083, 955, 836. HR-MS (ESI) calculated $[M-H]^+$ for $C_{25}H_{22}N_3O_5S^-$ = 476.12857, found: 476.12899. The enantiomeric excess of **48** was determined by HPLC analysis on Chiralpak AD-H column. Conditions: hexane/isopropanol = 70/30, flow rate = 1.0 mL/min, uv-vis detection at λ = 254 nm, t_R = 13.6 min (major), 53.5 min (minor).

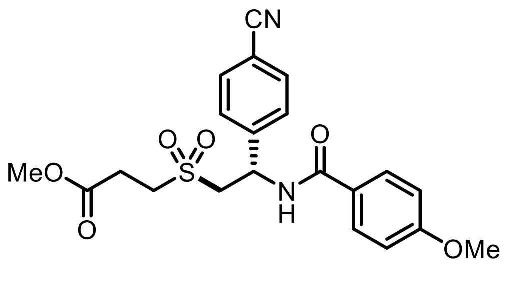

Methyl (*S*)-3-((2-(4-cyanophenyl)-2-(4-methoxybenzamido)ethyl)sulfonyl)propanoate (49). White solid, 42% yield, 95:5 er, $[\alpha]_D^{25}$ = -0.60 (c = 0.08, CD_3CN). ^{1}H NMR (400 MHz, $CDCl_3$) δ 7.78 (d, *J* = 9.0 Hz, 2H), 7.75 – 7.68 (m, 3H), 7.61 (d, *J* = 8.3 Hz, 2H), 6.98 (d, *J* = 9.0 Hz, 2H), 5.72 (ddd, *J* = 9.9, 7.7, 4.3 Hz, 1H), 3.82 (s, 3H), 3.76 (dd, *J* = 14.5, 9.9 Hz, 1H), 3.64 (s, 3H), 3.56 (dd, *J* = 14.6, 4.3 Hz, 1H), 3.34 (td, *J* = 7.4, 1.9 Hz, 2H), 2.78 (t, *J* = 7.3 Hz, 2H). ^{13}C NMR (101 MHz, CD_3CN) δ 172.3, 167.3, 163.9, 147.4, 134.0, 130.5, 129.1, 127.4, 119.9, 115.1, 112.7, 57.6, 56.6, 53.2, 50.6, 50.0, 27.9. IR (film): ν (cm^{-1}) 3349, 2932, 2235, 1731, 1629, 1605, 1505, 1256, 1177, 1122, 980, 837, 767. HR-MS (ESI) calculated $[M+H]^+$ for $C_{21}H_{23}N_2O_6S^+$ = 431.12713, found: 431.12722. The enantiomeric excess of **49** was determined by HPLC analysis on Chiralpak AD-H column. Conditions: hexane/isopropanol = 40/60, flow rate = 1.0 mL/min, uv-vis detection at λ = 254 nm, t_R = 16.4 min (major), 42.7 min (minor).

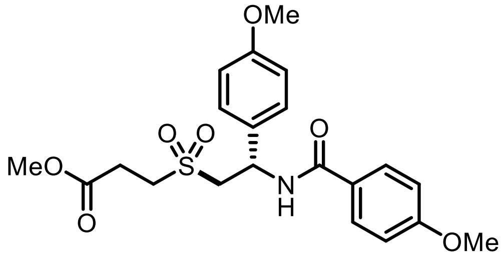

Methyl (*S*)-3-((2-(4-methoxybenzamido)-2-(4-methoxyphenyl)ethyl)sulfonyl)propanoate (50). White solid, 56% yield, 95:5 er, $[\alpha]_D^{25}$ = +1.36 (c = 0.09, $CHCl_3$). ^{1}H NMR (400 MHz, $CDCl_3$) δ 7.79 (d, *J* = 9.0 Hz, 2H), 7.45 (d, *J* = 7.3 Hz, 1H), 7.33 (d, *J* = 8.7 Hz, 2H), 6.91 (dd, *J* = 8.9, 6.6 Hz, 4H), 5.71 (td, *J* = 7.3, 4.9 Hz, 1H), 3.84 (s, 3H), 3.79 (s, 4H), 3.71 (s, 3H), 3.51 (dd, *J* = 15.0, 4.9 Hz, 1H), 3.10 (td, *J* = 7.2, 2.2 Hz, 2H), 2.79 (td, *J* = 7.1, 1.2 Hz, 2H). ^{13}C NMR (101 MHz, $CDCl_3$) δ 170.9, 166.3, 162.6, 159.6, 130.8, 129.0, 127.6, 125.9, 114.6, 113.9, 58.2, 55.4, 55.4, 52.5, 49.6, 49.1, 26.9. IR (film): ν (cm^{-1}) 3354, 2980, 1743, 1632, 1498, 1253, 1178, 1131, 851, 788. HR-MS (ESI) calculated $[M+H]^+$ for $C_{21}H_{26}NO_7S^+$ = 436.14245, found: 436.14253. The enantiomeric excess of **50** was determined by HPLC analysis on Chiralpak AD-H column. Conditions: hexane/isopropanol = 50/50,

flow rate = 1.0 mL/min, uv-vis detection at λ = 254 nm, t_R = 15.5 min (major), 30.0 min (minor).

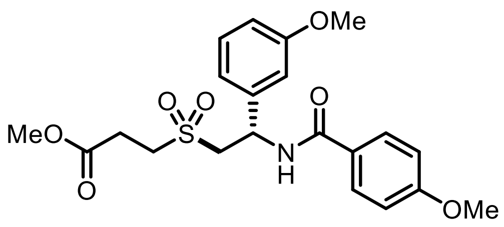

Methyl (*S*)-3-((2-(4-methoxybenzamido)-2-(3-methoxyphenyl)ethyl)sulfonyl)propanoate (51). White solid, 57% yield, 95:5 er, $[\alpha]_D^{25}$ = -20.20 (c = 0.08, $CHCl_3$). ^1H NMR (400 MHz, $CDCl_3$) δ 7.81 (d, *J* = 8.9 Hz, 2H), 7.57 – 7.49 (m, 1H), 7.30 (t, *J* = 7.9 Hz, 1H), 6.99 (dt, *J* = 7.6, 0.8 Hz, 1H), 6.96 – 6.90 (m, 3H), 6.87 – 6.80 (m, 1H), 5.75 (td, *J* = 7.6, 4.7 Hz, 1H), 3.85 (s, 3H), 3.80 (s, 3H), 3.77 (d, *J* = 7.5 Hz, 1H), 3.71 (s, 3H), 3.52 (dd, *J* = 15.0, 4.7 Hz, 1H), 3.10 (td, *J* = 7.2, 1.7 Hz, 2H), 2.82 – 2.75 (m, 2H). ^{13}C NMR (101 MHz, $CDCl_3$) δ 171.0, 166.5, 162.7, 160.3, 140.6, 130.5, 129.2, 125.9, 118.5, 114.0, 113.7, 112.5, 58.4, 55.6, 55.5, 52.6, 49.7, 49.6, 27.1. IR (film): ν (cm^{-1}) 3359, 2952, 1742, 1632, 1600, 1525, 1496, 1309, 1252, 1178, 1132, 851, 788. HR-MS (ESI) calculated $[M+H]^+$ for $C_{21}H_{26}NO_7S^+$ = 436.14245, found: 436.14245. The enantiomeric excess of **51** was determined by HPLC analysis on Chiralpak AD-H column. Conditions: hexane/isopropanol = 40/60, flow rate = 1.0 mL/min, uv-vis detection at λ = 210 nm, t_R = 13.1 min (major), 15.3 min (minor).

3. Late-stage Functionalization

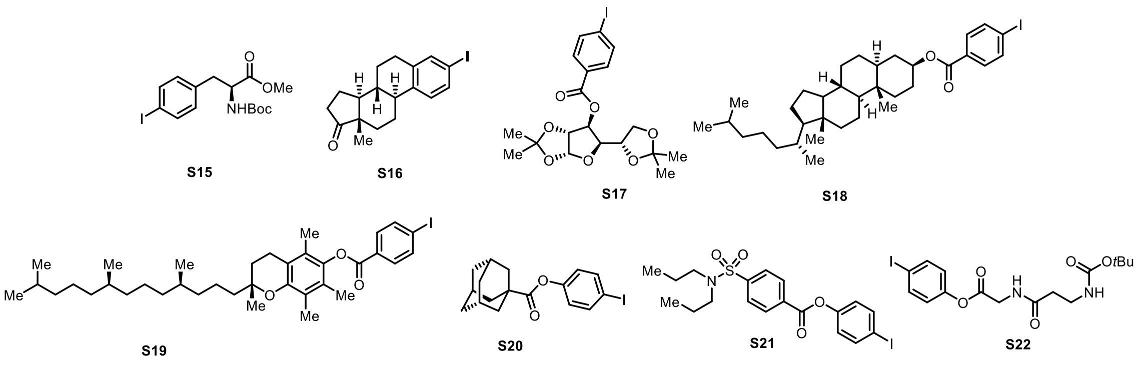

Iodo derivatives **S15**,[3] **S16**,[3] **S17**,[9] **S18**,[10] **S19**,[11] **S20**[12] and **S21**[12] were prepared according to reported procedures and analytical data were in agreement with previously reported values.

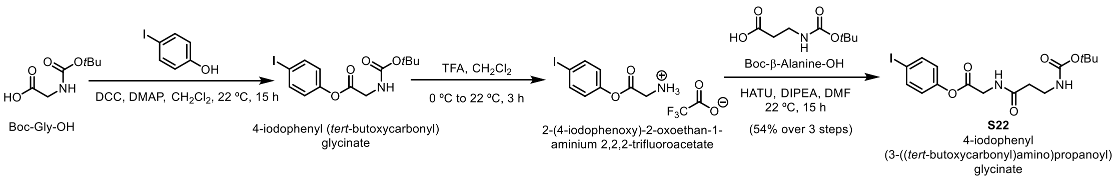

4-Iodophenyl (3-((tert-butoxycarbonyl)amino)propanoyl) glycinate (S22). Boc-Gly-OH (172 mg, 0.983 mmol, 1.0 equiv), 4-iodophenol (216 mg, 0.983 mmol, 1.0 equiv), DCC (223 mg, 1.08 mmol, 1.1 equiv) and DMAP (6 mg, 0.05 mmol, 0.05 equiv) were dissolved in CH_2Cl_2 (15 mL) and the reaction mixture was stirred at 25 °C for 15 h. After this time, the mixture was diluted with a saturated aqueous NH_4Cl solution and the mixture extracted with CH_2Cl_2 twice. The combined organic layers were washed with brine, dried over anhydrous $MgSO_4$, filtered and the solvent evaporated under reduced pressure to afford crude 4-iodophenyl (*tert*-butoxycarbonyl)glycinate (~0.983 mmol) which was used in the next step without further purification. Crude 4-iodophenyl (*tert*-butoxycarbonyl)glycinate (371 mg, 0.983 mmol, 1.0 equiv) obtained above was dissolved in CH_2Cl_2 (10 mL) and the resulting solution was cooled to 0 ºC. Then, TFA (7.3 mL, 98.3 mmol, 100 equiv) was added at 0 ºC and the reaction mixture was stirred for 3 h at 25 ºC. After this time, the excess of TFA and the organic solvent were removed under reduced pressure to afford crude 2-(4-iodophenoxy)-2-oxoethan-1-aminium

2,2,2-trifluoroacetate (~0.983 mmol) which was used in the next step without further purification. Crude 2-(4-iodophenoxy)-2-oxoethan-1-aminium 2,2,2-trifluoroacetate (500 mg, 0.983 mmol, 1.0 equiv) obtained above, Boc-β-Ala-OH (186 mg, 0.983 mmol, 1.0 equiv) and HATU (561 mg, 1.48 mmol, 1.5 equiv) were dissolved in DMF (3 mL) and then DIPEA (0.507 mL, 2.95 mmol, 3.0 equiv) was added. The reaction mixture was stirred at 25 ºC for 15 h. After this time, the mixture was diluted with a saturated aqueous NH_4Cl solution and extracted with EtOAc twice. The combined organic layers were washed with brine, dried over anhydrous $MgSO_4$, filtered and the solvent evaporated under reduced pressure. The crude product was purified by flash column chromatography (silica gel, 20% EtOAc in hexanes → 80% EtOAc in hexanes) to give 4-iodophenyl (3-((*tert*-butoxycarbonyl)amino)propanoyl)glycinate (240 mg, 54% over 3 steps) as a white foam.

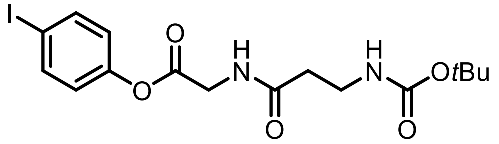

4-Iodophenyl (3-((tert-butoxycarbonyl)amino)propanoyl)glycinate (S22). white foam, 54% yield over three steps. ^{1}H NMR (400 MHz, $CDCl_3$) δ 7.70 (d, *J* = 8.9 Hz, 2H), 6.88 (d, *J* = 9.0 Hz, 2H), 6.28 (s, 1H), 5.13 (s, 1H), 4.28 (d, *J* = 5.4 Hz, 2H), 3.51 – 3.36 (m, 2H), 2.50 (t, *J* = 6.0 Hz, 2H), 1.42 (s, 9H). ^{13}C NMR (101 MHz, $CDCl_3$) δ 172.0, 168.4, 156.3, 150.2, 138.8, 123.6, 90.5, 41.6, 36.4, 28.5. IR (film): ν (cm^{-1}) 3396, 2967, 2914, 1747, 1296, 1170, 1156, 988, 789, 751. HR-MS (ESI) calculated $[M+H]^+$ for $C_{16}H_{22}IN_2O_5^+$ = 449.05679, found: 449.05688.

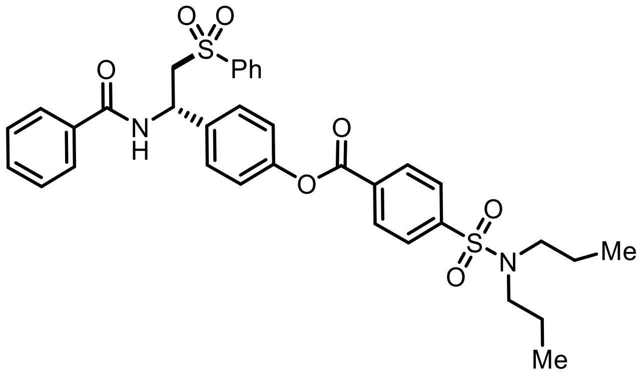

(*S*)-4-(1-Benzamido-2-(phenylsulfonyl)ethyl)phenyl 4-(*N,N*-dipropylsulfamoyl)benzoate (52). White solid, 80% yield, 95:5 er, $[\alpha]_D^{25}$ = -3.21 (c = 0.17, $CHCl_3$). ^1H NMR (500 MHz, $CDCl_3$) δ 8.28 (d, *J* = 8.5 Hz, 2H), 7.93 (d, *J* = 8.5 Hz, 2H), 7.88 – 7.81 (m, 4H), 7.63 (t, *J* = 7.5 Hz, 1H), 7.56 – 7.50 (m, 4H), 7.46 (td, *J* = 7.6, 2.9 Hz, 2H), 7.33 (dd, *J* = 8.8, 2.5 Hz, 2H), 7.12 (dd, *J* = 8.7, 2.6 Hz, 2H), 5.51 (ddd, *J* = 9.7, 6.5, 4.0 Hz, 1H), 3.83 (dd, *J* = 14.8, 9.2 Hz, 1H), 3.61 (dt, *J* = 14.8, 3.6 Hz, 1H), 3.16 – 3.08 (m, 4H), 1.56 (h, *J* = 7.5 Hz, 4H), 0.88 (t, *J* = 7.4 Hz, 6H). ^{13}C NMR (126 MHz, $CDCl_3$) δ 166.9, 163.8, 150.4, 145.2, 138.9, 137.1, 134.3, 133.6, 132.7, 132.2, 130.9, 129.7, 128.9, 128.1, 127.7, 127.3, 127.3, 122.1, 60.3, 50.1, 49.8, 22.1, 11.3. IR (film): ν (cm^{-1}) 3394, 2968, 1753, 1698, 1664, 1533, 1509, 1488, 1341, 1201, 899, 751. HR-MS (ESI) calculated $[M+Cl]^-$ for $C_{23}H_{19}Cl_2N_2O_4S^-$ = 489.04481, found: 489.04495. The enantiomeric excess of **52** was determined by HPLC analysis on Chiralpak AD-H column. Conditions: hexane/isopropanol = 60/40, flow rate = 1.0 mL/min, uv-vis detection at λ = 210 nm, t_R = 19.6 min (major), 34.8 min (minor). Crystals of **52** suitable for X-ray diffraction were obtained from a mixture of hexane/EA.

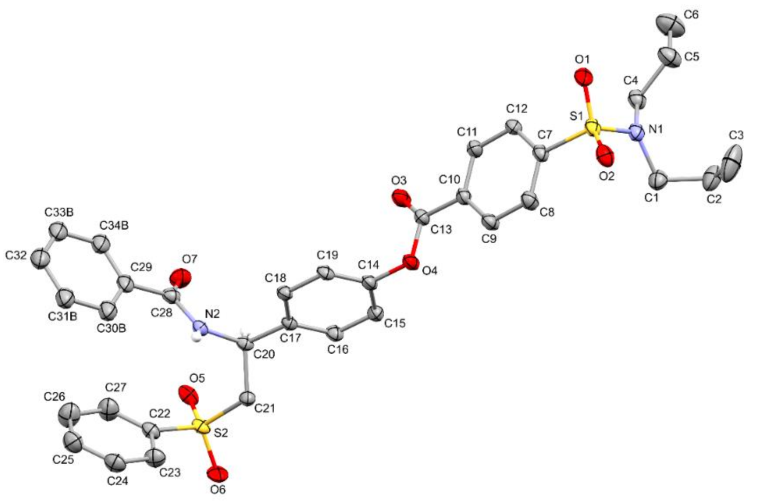

Figure S2. The molecular structure of **52** with the atomic numbering scheme. Displacement ellipsoids are drawn at the 30% probability level. Disorders and selected hydrogen atoms were removed for clarity.

Table S9 Crystal data and structure refinement for 52.

Identification code	xiao2707
Empirical formula	$C_{34}H_{36}N_2O_7S_2$
Formula weight	648.77
Temperature/K	160(1)
Crystal system	triclinic
Space group	P1
a/Å	5.4109(2)
b/Å	8.3457(3)
c/Å	18.5255(5)
α/°	101.705(3)
β/°	96.627(3)
γ/°	96.771(3)
Volume/Å^3	805.07(5)
Z	1
ρ_{calc}g/cm^3	1.338
μ/mm^{-1}	1.925
F(000)	342.0
Crystal size/mm^3	0.26 × 0.05 × 0.04
Radiation	Cu Kα (λ = 1.54184)
2Θ range for data collection/°	4.922 to 153.156
Index ranges	-6 ≤ h ≤ 6, -10 ≤ k ≤ 10, -23 ≤ l ≤ 20
Reflections collected	27212
Independent reflections	5942 [R_{int} = 0.0482, R_{sigma} = 0.0332]
Data/restraints/parameters	5942/369/438
Goodness-of-fit on F^2	1.065
Final R indexes [I>=2σ (I)]	R_1 = 0.0573, wR_2 = 0.1525
Final R indexes [all data]	R_1 = 0.0617, wR_2 = 0.1591
Largest diff. peak/hole / e Å^{-3}	0.51/-0.48
Flack parameter	0.005(18)

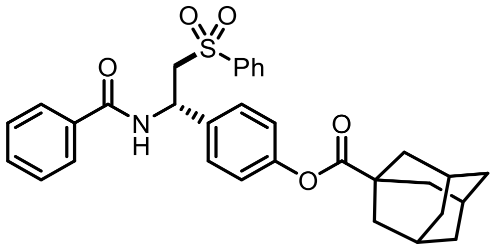

4-((*S*)-1-Benzamido-2-(phenylsulfonyl)ethyl)phenyl (*3S,5S,7S*)-adamantane-1-carboxylate (53). White solid, 59% yield, 98:2 er, $[\alpha]_D^{25}$ = -0.69 (c = 0.19, $CHCl_3$). ^1H NMR (500 MHz, $CDCl_3$) δ 7.81 (d, *J* = 7.5 Hz, 4H), 7.61 (tt, *J* = 7.2, 1.4 Hz, 1H), 7.56 – 7.47 (m, 3H), 7.47 – 7.38 (m, 3H), 7.22 (td, *J* = 5.3, 2.5 Hz, 2H), 6.96 – 6.90 (m, 2H), 5.45 (ddd, *J* = 9.9, 6.5, 4.2 Hz, 1H), 3.82 – 3.75 (m, 1H), 3.55 (ddd, *J* = 14.8, 7.6, 3.7 Hz, 1H), 2.07 (p, *J* = 2.9 Hz, 3H), 2.02 (d, *J* = 2.9 Hz, 6H), 1.80 – 1.72 (m, 6H). ^{13}C NMR (126 MHz, $CDCl_3$) δ 176.2, 166.8, 150.9, 138.9, 136.3, 134.2, 133.7, 132.0, 129.6, 128.8, 128.1, 127.4, 127.3, 122.2, 60.3, 49.7, 41.2, 38.9, 36.6, 28.0. IR (film): ν (cm^{-1}) 3656, 2968, 1646, 1525, 1286, 1236, 1084, 952, 720. HR-MS (ESI) calculated $[M+Cl]^-$ for $C_{23}H_{19}Cl_2N_2O_4S^-$ = 489.04481, found: 489.04495. The enantiomeric excess of **53** was determined by HPLC analysis on Chiralpak AD-H column. Conditions: hexane/isopropanol = 60/40, flow rate = 1.0 mL/min, uv-vis detection at λ = 220 nm, t_R = 18.0 min (major), 30.7 min (minor).

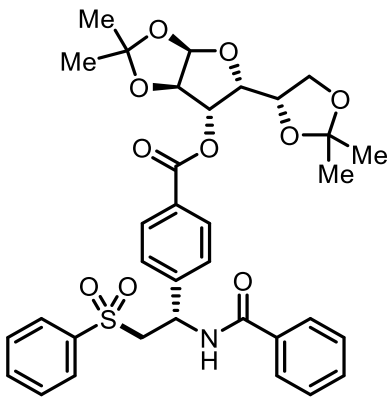

(*3aR,5R,6S,6aR*)-5-((*S*)-2,2-Dimethyl-1,3-dioxolan-4-yl)-2,2-dimethyltetrahydrofuro[2,3-d][1,3]dioxol-6-yl 4-((*S*)-1-benzamido-2-(phenylsulfonyl)ethyl)benzoate (54). White solid, 62% yield, 96/4 dr, $[\alpha]_D^{25}$ = -30.24 (c = 0.19, $CHCl_3$). ^1H NMR (500 MHz, $CDCl_3$) δ 7.93 (d, *J* = 8.3 Hz, 2H), 7.85

(dd, J = 12.4, 7.6 Hz, 4H), 7.73 (d, J = 6.4 Hz, 1H), 7.64 (d, J = 14.9 Hz, 1H), 7.51 (ddd, J = 30.5, 13.8, 7.5 Hz, 5H), 7.37 (d, J = 8.4 Hz, 2H), 5.93 (d, J = 3.7 Hz, 1H), 5.56 – 5.46 (m, 2H), 4.59 (d, J = 3.6 Hz, 1H), 4.31 (d, J = 3.2 Hz, 2H), 4.14 – 4.07 (m, 2H), 3.82 (dd, J = 14.8, 9.2 Hz, 1H), 3.59 (dd, J = 14.8, 3.9 Hz, 1H), 1.56 (s, 3H), 1.41 (s, 3H), 1.32 (s, 3H), 1.27 (s, 3H). ^{13}C NMR (126 MHz, $CDCl_3$) δ 167.0, 164.7, 144.8, 138.7, 134.4, 133.4, 132.3, 130.5, 129.7, 129.4, 128.9, 128.1, 127.3, 126.5, 112.5, 109.6, 105.3, 83.5, 80.1, 72.7, 67.4, 60.0, 50.2, 31.6, 27.0, 26.9, 26.3, 25.4. HR-MS (ESI) calculated $[M+Cl]^-$ for $C_{23}H_{19}Cl_2N_2O_4S^-$ = 489.04481, found: 489.04495. IR (film): ν (cm^{-1}) 3358, 2333, 1716, 1521, 1264, 1096, 1073, 709. The enantiomeric excess of **54** was determined by HPLC analysis on Chiralpak AD-H column. Conditions: hexane/isopropanol = 60/40, flow rate = 1.0 mL/min, uv-vis detection at λ = 254 nm, t_R = 17.2 min (major), 36.8 min (minor).

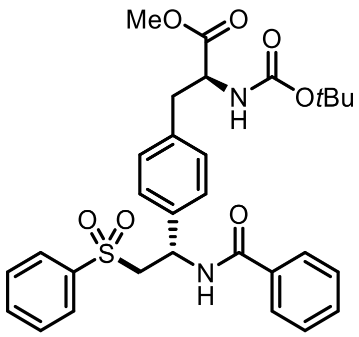

Methyl (*S*)-3-(4-((*S*)-1-benzamido-2-(phenylsulfonyl)ethyl)phenyl)-2-((tert-butoxycarbonyl)amino)propanoate (55). White solid, 73% yield, 95/5 dr. $[\alpha]_D^{25}$ = +32.45 (c = 0.17, $CHCl_3$). ^{1}H NMR (500 MHz, $CDCl_3$) δ 7.82 (t, J = 7.5 Hz, 4H), 7.60 (d, J = 7.5 Hz, 1H), 7.54 – 7.41 (m, 6H), 7.17 (d, J = 7.7 Hz, 2H), 7.02 (d, J = 7.7 Hz, 2H), 5.45 (dq, J = 10.1, 5.4, 4.5 Hz, 1H), 4.92 (d, J = 8.4 Hz, 1H), 4.53 (d, J = 7.0 Hz, 1H), 3.82 (dd, J = 14.9, 9.0 Hz, 1H), 3.70 (s, 3H), 3.59 (dd, J = 15.1, 4.2 Hz, 1H), 3.08 – 2.93 (m, 2H), 1.39 (s, 9H). ^{13}C NMR (126 MHz, $CDCl_3$) δ 172.3, 166.8, 155.2, 138.9, 137.8, 136.2, 134.2, 133.8, 132.0, 130.0, 129.6, 128.8, 128.1, 127.3, 126.6, 80.2, 60.3, 54.4, 52.4, 49.8, 37.9, 31.6, 28.4. IR (film): ν (cm^{-1}) 3320, 2250, 1747, 1705, 1518, 1489, 1164, 801, 712. HR-MS (ESI) calculated $[M+Cl]^-$ for $C_{23}H_{19}Cl_2N_2O_4S^-$ = 489.04481, found: 489.04495. The enantiomeric excess of **55** was determined by HPLC

analysis on Chiralpak OJ-H column. Conditions: hexane/isopropanol = 75/25, flow rate = 0.8 mL/min, uv-vis detection at λ = 220 nm, t_R = 34.5 min (major), 65.0 min (minor).

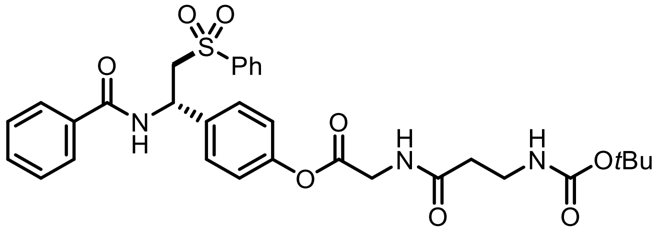

(*S*)-4-(1-Benzamido-2-(phenylsulfonyl)ethyl)phenyl (3-((tert-butoxycarbonyl)amino)propanoyl)glycinate (56). White solid, 83% yield, 96:4 er, $[\alpha]_D^{25}$ = -1.52 (c = 0.13, $CHCl_3$). ^{1}H NMR (400 MHz, $CDCl_3$) δ 7.82 – 7.74 (m, 4H), 7.70 – 7.63 (m, 1H), 7.60 – 7.55 (m, 1H), 7.52 – 7.44 (m, 3H), 7.40 (td, *J* = 6.8, 1.7 Hz, 2H), 7.21 (d, *J* = 8.8 Hz, 2H), 6.93 (d, *J* = 8.6 Hz, 2H), 6.64 – 6.53 (m, 1H), 5.49 (ddd, *J* = 9.3, 6.8, 3.8 Hz, 1H), 5.19 (s, 1H), 4.22 (d, *J* = 5.6 Hz, 2H), 3.88 – 3.70 (m, 1H), 3.55 – 3.48 (m, 1H), 3.38 (d, *J* = 6.0 Hz, 2H), 2.43 (t, *J* = 6.1 Hz, 2H), 1.40 (s, 9H). ^{13}C NMR (101 MHz, $CDCl_3$) δ 172.1, 168.6, 166.9, 156.3, 150.0, 138.8, 137.1, 134.3, 133.6, 132.1, 129.7, 128.8, 128.1, 127.6, 127.3, 121.9, 79.6, 70.8, 60.3, 49.7, 41.6, 36.3, 28.5. IR (film): ν (cm^{-1}) 3302, 2980, 1646, 1530, 1370, 1250, 1143, 1083, 843, 743. HR-MS (ESI) calculated $[M+Na]^+$ for $C_{31}H_{35}N_3O_8SNa^+$ = 632.20371, found: 632.20370. The enantiomeric excess of **56** was determined by HPLC analysis on Chiralpak AD-H column. Conditions: hexane/isopropanol = 80/20, flow rate = 1.0 mL/min, uv-vis detection at λ = 220 nm, t_R = 32.4 min (major), 76.8 min (minor).

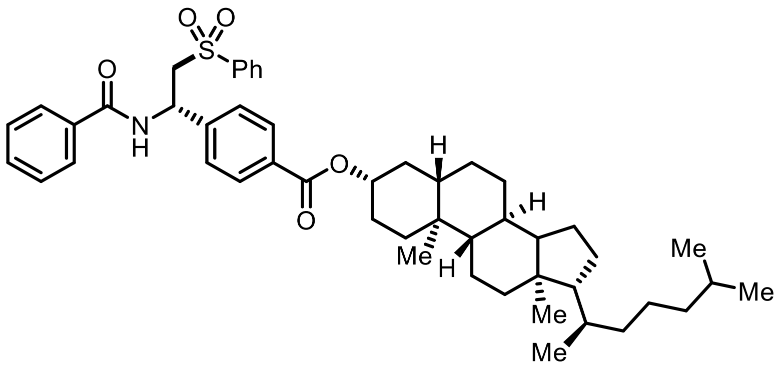

(*3S,5S,8R,9S,10S,13R,17R*)-10,13-Dimethyl-17-((*R*)-6-methylheptan-2-yl)hexadecahydro-1H-cyclopenta[a]phenanthren-3-yl 4-((*S*)-1-benzamido-2-

(phenylsulfonyl)ethyl)benzoate (57). White solid. 43% yield, 96/4 dr. $[\alpha]_D^{25}$ = +8.96 (c = 0.18, $CHCl_3$). ^{1}H NMR (400 MHz, $CDCl_3$) δ 7.91 (d, *J* = 8.5 Hz, 2H), 7.87 – 7.79 (m, 4H), 7.65 – 7.59 (m, 2H), 7.56 – 7.43 (m, 5H), 7.31 (d, *J* = 8.4 Hz, 2H), 5.50 (ddd, *J* = 9.8, 6.3, 3.9 Hz, 1H), 4.96 – 4.86 (m, 1H), 3.81 (dd, *J* = 14.8, 9.2 Hz, 1H), 3.58 (dd, *J* = 14.8, 4.0 Hz, 1H), 2.03 – 1.44 (m, 13H), 1.34 (s, 7H), 1.22 (s, 2H), 1.18 – 0.96 (m, 9H), 0.89 (s, 3H), 0.87 – 0.84 (m, 9H), 0.65 (s, 3H). ^{13}C NMR (126 MHz, $CDCl_3$) δ 166.9, 165.6, 143.7, 138.8, 134.3, 133.5, 132.2, 130.9, 130.3, 129.7, 128.9, 128.1, 127.3, 126.2, 74.7, 60.1, 56.6, 56.4, 54.4, 50.2, 44.8, 42.7, 40.1, 39.7, 36.9, 36.3, 35.9, 35.7, 35.6, 34.2, 32.1, 28.8, 28.4, 28.2, 27.7, 24.4, 24.0, 23.0, 22.7, 21.4, 18.8, 12.4, 12.2. IR (film): ν (cm^{-1}) 3373, 2980, 2919, 1730, 1645, 1525, 1309, 1274, 1237, 1144, 1086, 743. HR-MS (ESI) calculated $[M+Cl]^-$ for $C_{23}H_{19}Cl_2N_2O_4S^-$ = 489.04481, found: 489.04495. The enantiomeric excess of **57** was determined by HPLC analysis on Chiralpak AD-H column. Conditions: hexane/isopropanol = 60/40, flow rate = 1.0 mL/min, uv-vis detection at λ = 254 nm, t_R = 8.0 min (major), 20.0 min (minor).

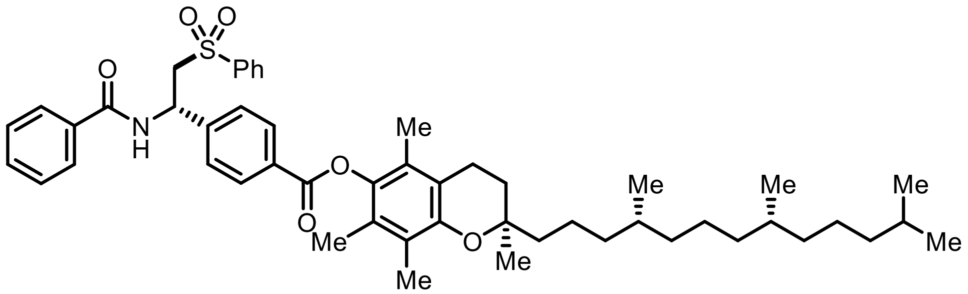

(*R*)-2,5,7,8-Tetramethyl-2-((*4R,8R*)-4,8,12-trimethyltridecyl)chroman-6-yl 4-((*S*)-1-benzamido-2-(phenylsulfonyl)ethyl)benzoate (58). White solid. 40% yield, 96/4 dr. $[\alpha]_D^{25}$ = -8.66 (c = 0.22, $CHCl_3$). ^{1}H NMR (400 MHz, $CDCl_3$) δ 8.17 – 8.10 (m, 2H), 7.89 (dd, *J* = 7.0, 1.6 Hz, 2H), 7.85 – 7.80 (m, 2H), 7.71 (d, *J* = 6.3 Hz, 1H), 7.66 – 7.60 (m, 1H), 7.58 – 7.46 (m, 5H), 7.40 (d, *J* = 8.4 Hz, 2H), 5.55 (ddd, *J* = 9.5, 6.1, 4.0 Hz, 1H), 3.84 (dd, *J* = 14.8, 9.0 Hz, 1H), 3.64 (dd, *J* = 14.8, 4.0 Hz, 1H), 2.61 (t, *J* = 6.8 Hz, 2H), 2.11 (s, 3H), 2.02 (s, 3H), 1.98 (s, 3H), 1.83 (dt, *J* = 13.9, 7.1 Hz, 2H), 1.54 (dt, *J* = 13.3, 6.7 Hz, 6H), 1.43 – 1.27 (m, 8H), 1.24 – 1.00 (m, 10H), 0.89 – 0.81 (m, 12H). ^{13}C NMR (126 MHz, $CDCl_3$) δ 167.0, 164.6, 149.7, 144.5, 140.6, 138.8, 134.4, 133.4,

132.3, 131.0, 129.7, 129.5, 128.9, 128.1, 127.3, 126.9, 126.5, 125.2, 123.3, 117.7, 75.3, 60.1, 50.3, 39.5, 37.6, 37.6, 37.4, 32.9, 32.9, 32.8, 28.1, 25.0, 24.6, 22.9, 22.8, 21.2, 20.8, 19.9, 19.8, 13.2, 12.3, 12.0. IR (film): ν (cm^{-1}) 3363, 2916, 1736, 1641, 1526, 1446, 1307, 1292, 1017, 746. HR-MS (ESI) calculated $[M+Cl]^-$ for $C_{23}H_{19}Cl_2N_2O_4S^-$ = 489.04481, found: 489.04495. The enantiomeric excess of **58** was determined by HPLC analysis on Chiralpak AD-H column. Conditions: hexane/isopropanol = 70/30, flow rate = 1.0 mL/min, uv-vis detection at λ = 254 nm, t_R = 6.0 min (major), 10.0 min (minor).

4. Mechanistic Studies

4.1 Control experiments with radical inhibitors

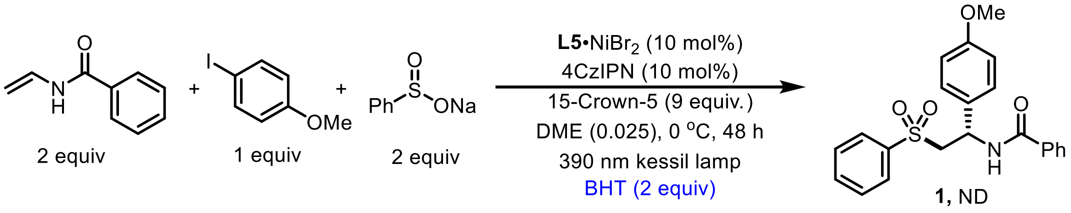

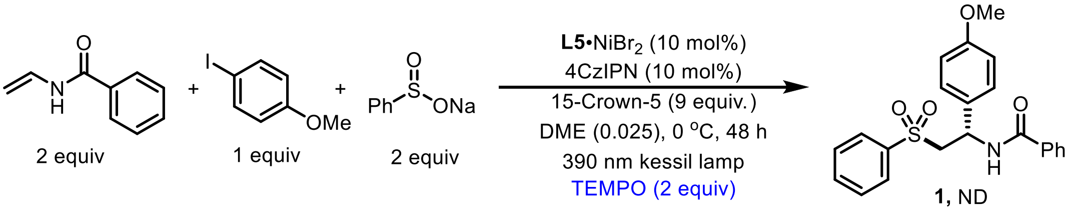

An oven-dried 7.5 mL screw-cap vial equipped with a magnetic stirring bar was charged with the vinyl amide (0.2 mmol, 2 equiv), $PhSO_2Na$ (0.2 mmol, 2 equiv), 4-iodoanisole (0.1 mmol, 1 equiv), 4-CzIPN (0.01 mmol, 10 mol%), **L5**·$NiBr_2$ (0.01 mmol, 10 mol%) and radical inhibitor (0.2 mmol, 2 equiv) and then introduced into a glove box filled with nitrogen. There, dry DME (4 mL) and 15-crown-5 (0.9 mmol, 9 equiv) were sequentially added. The reaction vessel was then capped and removed from the glove box. The reaction was stirred (1000 rpm) under irradiation with 390 nm kessil lamp at 0 ºC for 48 h. After that, the reaction mixture was concentrated in rotary evaporator and the residue was analyzed by HR-MS an LC-MS.

Addition of BHT or TEMPO completely shut down the desired transformation, and an alkyl-TEMPO adduct **59** was detected by HR-MS and LC-MS (Figure S3 & S4).

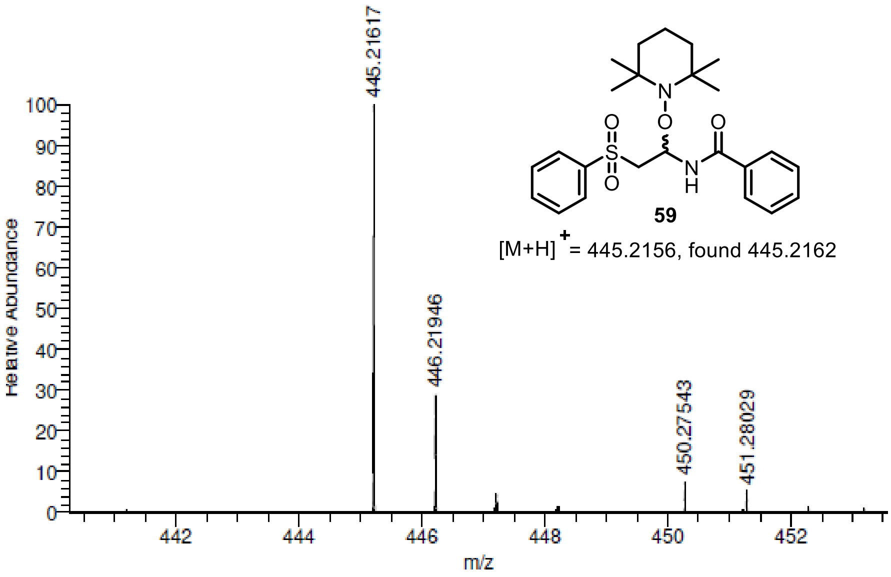

Figure S3. HR-MS for alkyl-TEMPO adduct **59**

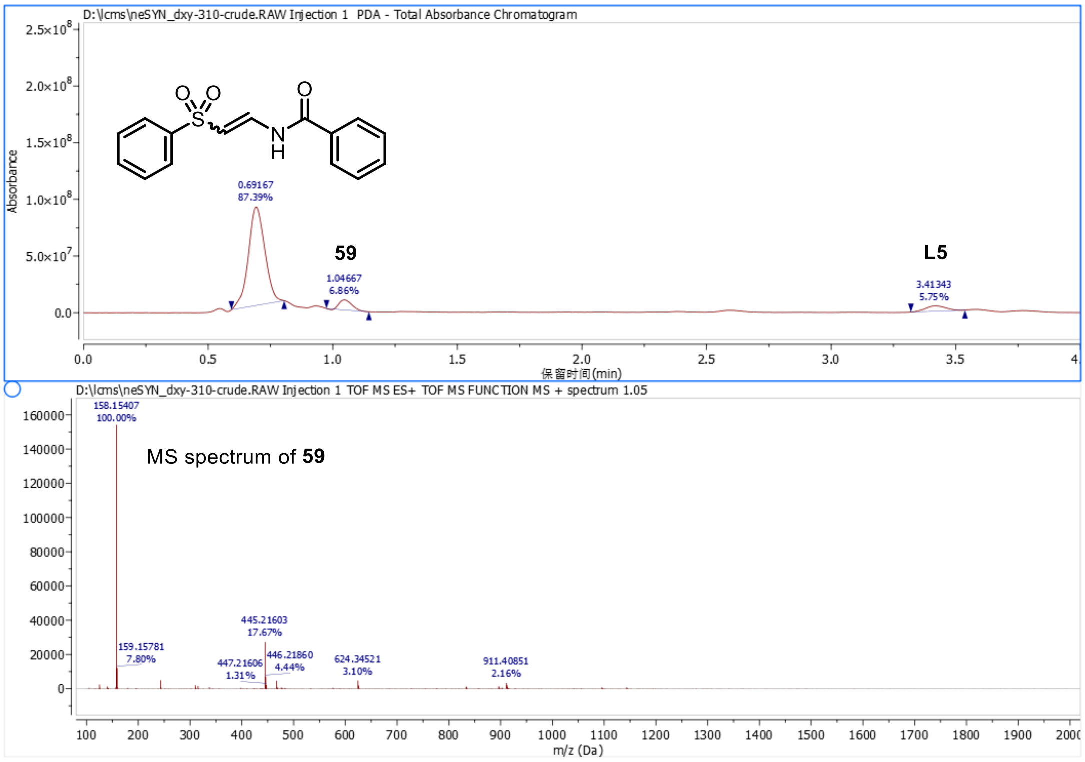

Figure S4. LC-MS of crude reaction mixture

4.2 Control experiments with hydrogen atom donors

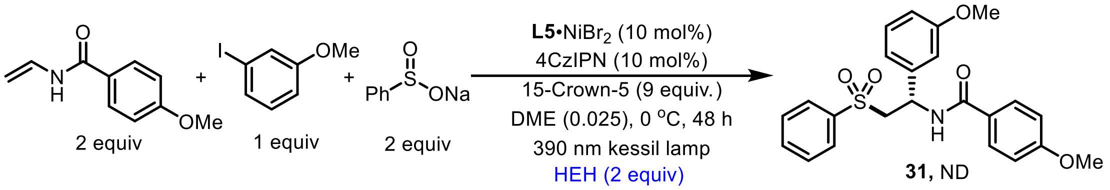

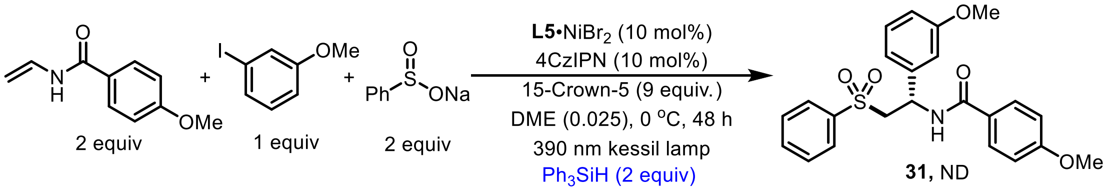

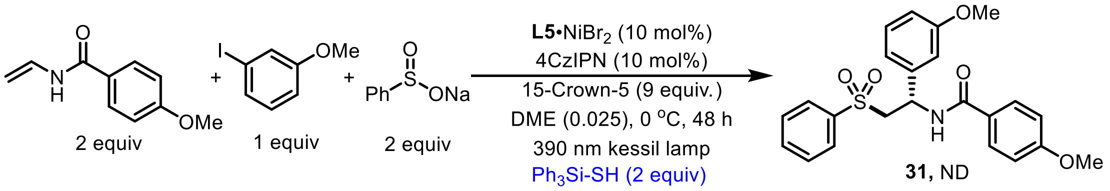

An oven-dried 7.5 mL screw-cap vial equipped with a magnetic stirring bar was charged with the vinyl amide (0.2 mmol, 2 equiv), $PhSO_2Na$ (0.2 mmol, 2 equiv), 3-Iodoanisole (0.1 mmol, 1 equiv), 4-CzIPN (0.01 mmol, 10 mol%), **L5**·$NiBr_2$ (0.01 mmol, 10 mol%) and hydrogen atom donor (0.2 mmol, 2 equiv) and then introduced into a glove box filled with nitrogen. There, dry DME (4 mL) and 15-crown-5 (0.9 mmol, 9 equiv) were sequentially added. The reaction vessel was then capped and removed from the glove box. The reaction was stirred (1000 rpm) under irradiation with 390 nm kessil lamp at 0 °C for 48 h. After that, the reaction mixture was concentrated in rotary evaporator and the residue was analyzed by HR-MS.

Addition of Hantzsch ester, triphenylsilane or triphenylsilanethiol completely shut down the desired transformation, an alkyl-H adduct **60** was observed by HR-MS in the presence of two equivalents of Triphenylsilanethiol (Figure S5).

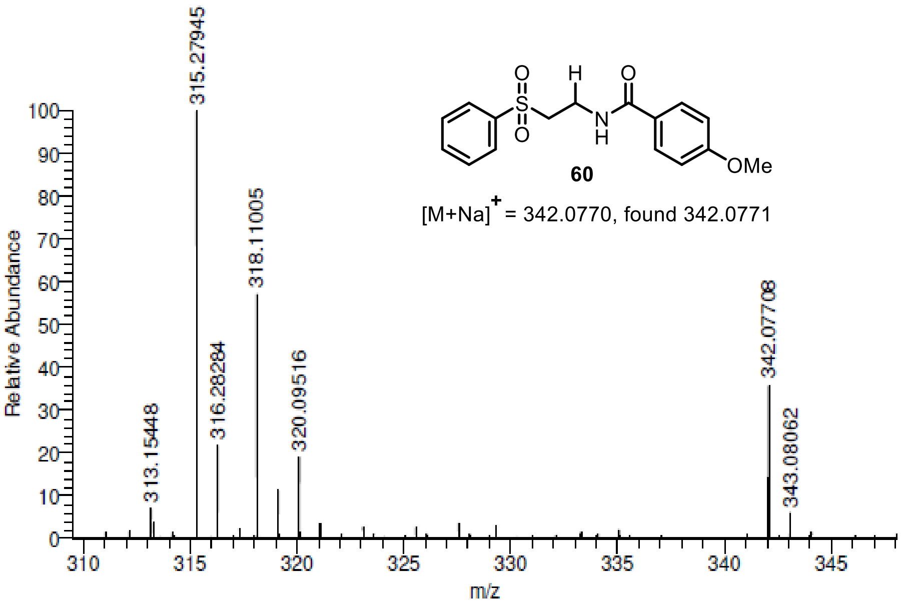

Figure S5. HR-MS for alkyl-H adduct **60**

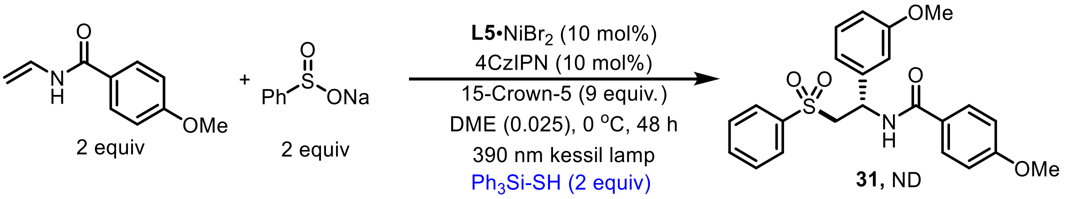

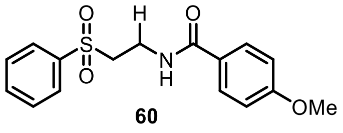

detected by HR-MS

[M+Na]$^+$ = 342.0770, found 342.0769

An oven-dried 7.5 mL screw-cap vial equipped with a magnetic stirring bar was charged with the vinyl amide (0.2 mmol, 2 equiv), $PhSO_2Na$ (0.2 mmol, 2 equiv), 4-CzIPN (0.01 mmol, 10 mol%), **L5**·$NiBr_2$ (0.01 mmol, 10 mol%) and Ph_3Si-SH (0.2 mmol, 2 equiv) and then introduced into a glove box filled with nitrogen. There, dry DME (4 mL) and 15-crown-5 (0.9 mmol, 9 equiv) were sequentially added. The reaction vessel was then capped and removed from the glove box. The reaction was stirred (1000 rpm) under irradiation with 390 nm kessil lamp at 0 °C for 48 h. After that, the reaction mixture was concentrated in rotary evaporator and the residue was analyzed by HR-MS. The same alkyl-H adduct **60** was observed by HR-MS in the absence of 3-Iodoanisole.

4.3 Control experiments with $BrCCl_3$

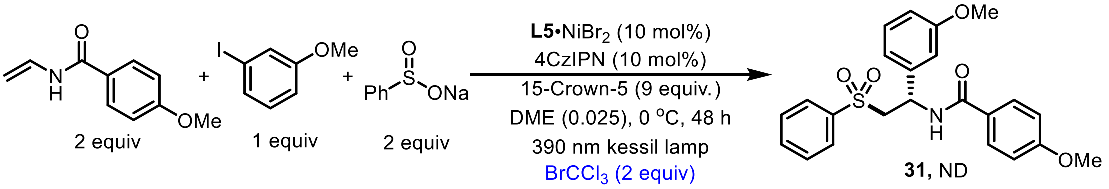

An oven-dried 7.5 mL screw-cap vial equipped with a magnetic stirring bar was charged with the vinyl amide (0.2 mmol, 2 equiv), $PhSO_2Na$ (0.2 mmol, 2 equiv), 3-Iodoanisole (0.1 mmol, 1 equiv), 4-CzIPN (0.01 mmol, 10 mol%), **L5**·$NiBr_2$ (0.01 mmol, 10 mol%) and then introduced into a glove box filled with nitrogen. There, dry DME (4 mL) and 15-crown-5 (0.9 mmol, 9 equiv) were sequentially added. The reaction vessel was then capped and removed from the glove box. After that, $BrCCl_3$ (0.2 mmol, 2 equiv) was added to the reaction mixture. The reaction was stirred (1000 rpm) under irradiation with 390 nm kessil lamp at 0 °C for 48 h. After that, the reaction mixture was concentrated in rotary evaporator and the residue was analyzed by LC-MS and HR-MS.

Addition of $BrCCl_3$ completely shut down the desired transformation, alkyl-CCl_3 adduct **61a** and alkenyl sulfone **61b** were observed by LC-MS in the presence of two equivalents of $BrCCl_3$ (Figure S6 & S7).

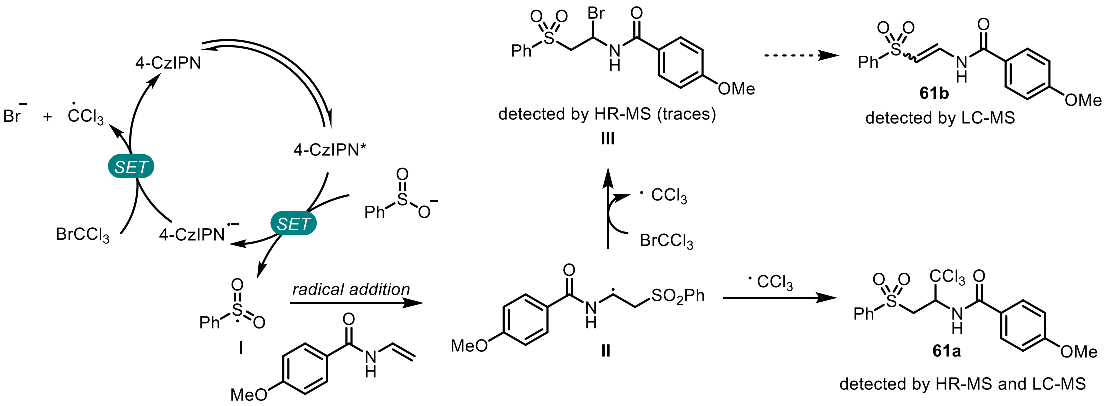

Figure S6. Proposed pathways to form **61a** and **61b**

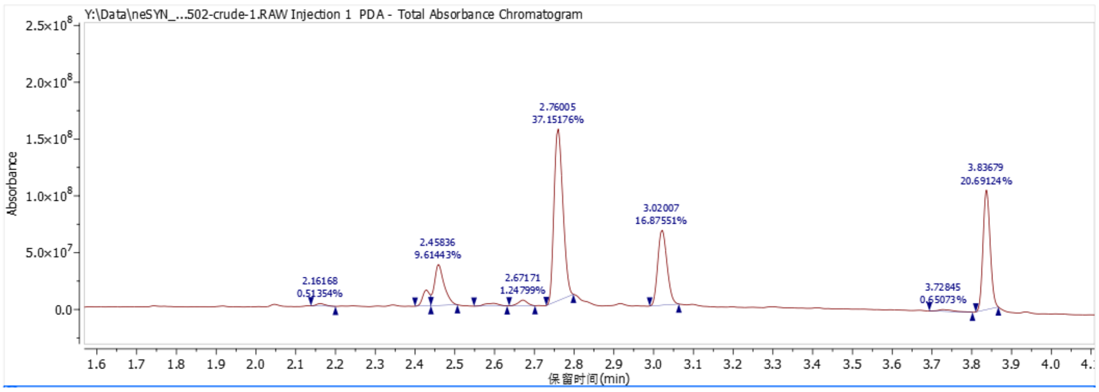

The following compounds were detected by LC-MS:

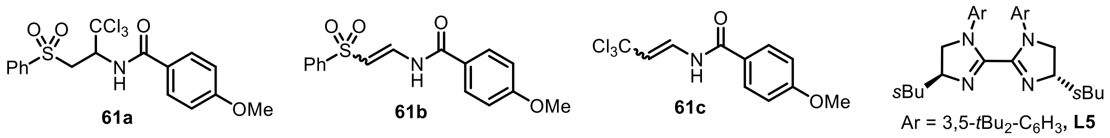

Figure S7. LC-MS spectrum of the reaction mixture

4.4 Radical clock reaction

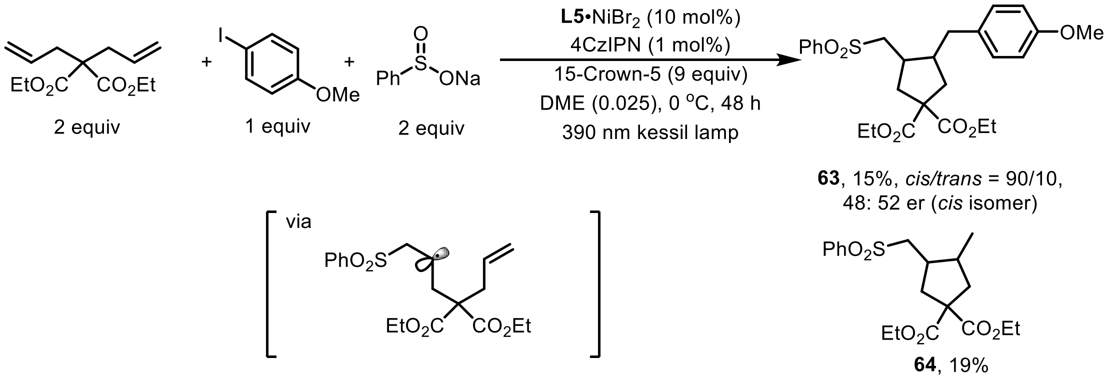

An oven-dried 7.5 mL screw-cap vial equipped with a magnetic stirring bar was charged with $PhSO_2Na$ (0.2 mmol, 2 equiv), 4-Iodoanisole (0.1 mmol, 1 equiv), 4-CzIPN (0.01 mmol, 10 mol%), **L5**·$NiBr_2$ (0.01 mmol, 10 mol%) and then introduced into a glove box filled with nitrogen. There, dry DME (4 mL) and 15-crown-5 (0.9 mmol, 9 equiv) were sequentially added. The reaction vessel was then capped and removed from the glove box. After that, diethyl diallylmalonate (0.2 mmol, 2 equiv) was added to the reaction mixture. The reaction was stirred (1000 rpm) under irradiation with 390 nm kessil lamp at 0 °C for 48 h. To quench the reaction, a saturated aq. solution of NaCl (1 mL) was added and the resulting mixture was extracted with EtOAc

(3x2mL). The organic phase was concentrated in rotary evaporator and the residue was purified by column chromatography on silica gel. The er values were determined by HPLC with a chiral column.

The obtain of 5-*exo* cyclized product **63** revealed the involvement of a radical intermediate in this reaction.

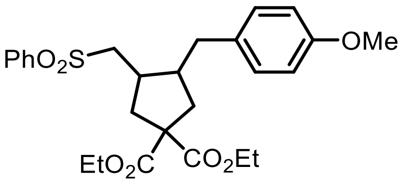

Diethyl 3-(4-methoxybenzyl)-4-((phenylsulfonyl)methyl)cyclopentane-1,1-dicarboxylate (63). Colorless oil, 15% yield, *cis/trans* = 90/10, 48:52 er (cis isomer). ^{1}H NMR (500 MHz, $CDCl_3$) δ 7.89 (dd, J = 7.1, 1.4 Hz, 2H), 7.67 – 7.63 (m, 1H), 7.56 (t, J = 7.7 Hz, 2H), 7.02 (d, J = 8.6 Hz, 2H), 6.78 (d, J = 8.6 Hz, 2H), 4.22 – 4.11 (m, 4H), 3.77 (s, 3H), 3.28 (dd, J = 14.0, 5.1 Hz, 1H), 3.16 (dd, J = 14.0, 8.4 Hz, 1H), 2.62 (dd, J = 13.3, 5.2 Hz, 1H), 2.58 – 2.49 (m, 2H), 2.43 (dt, J = 11.4, 5.7 Hz, 1H), 2.36 – 2.15 (m, 3H), 2.07 (dd, J = 14.2, 6.1 Hz, 1H), 1.24 (t, J = 7.2 Hz, 3H), 1.20 (t, J = 7.2 Hz, 3H). ^{13}C NMR (126 MHz, $CDCl_3$) δ 172.8, 172.3, 158.1, 139.7, 133.8, 131.8, 129.9, 129.5, 128.1, 114.0, 61.8, 61.7, 58.3, 56.2, 55.4, 44.0, 38.3, 37.7, 37.0, 33.9, 14.2, 14.1. HR-MS (ESI) calculated $[M+Na]^+$ for $C_{26}H_{32}O_7SNa^+$ = 511.17610, found: 511.17484. The enantiomeric excess of **63** was determined by HPLC analysis on Chiralpak AD-H column. Conditions: hexane/isopropanol = 70/30, flow rate = 1.0 mL/min, uv-vis detection at λ = 220 nm, t_R = 38.3 min (minor), 45.7 min (major).

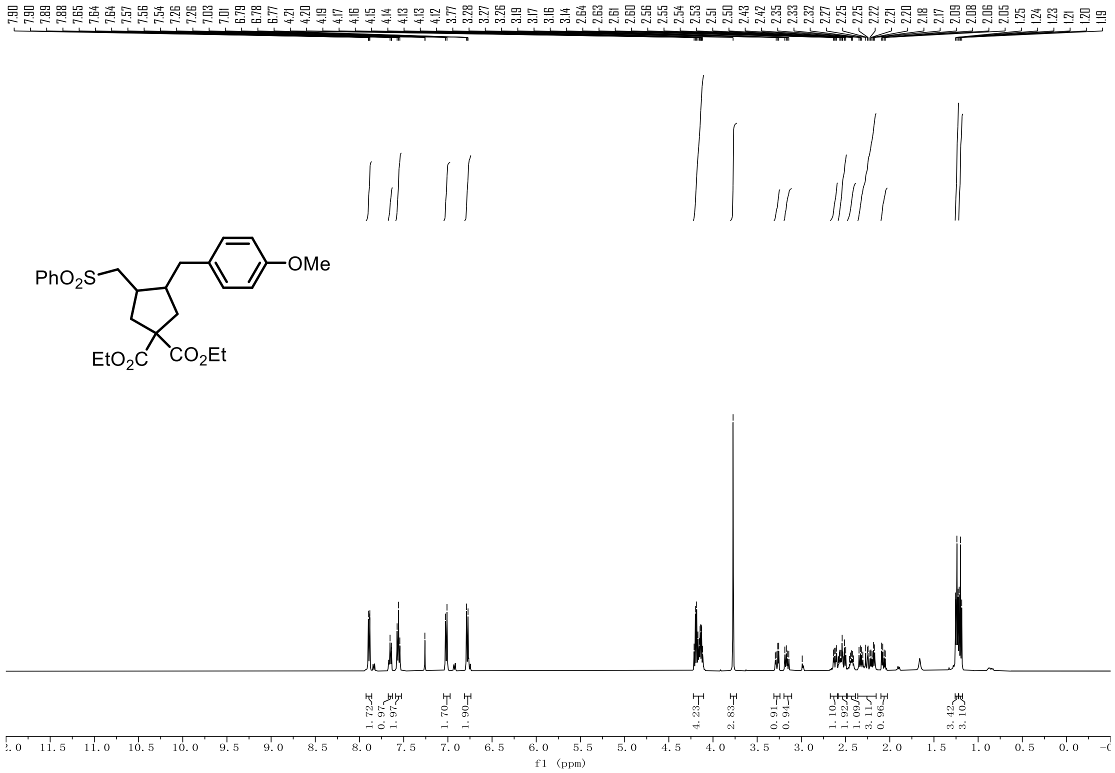

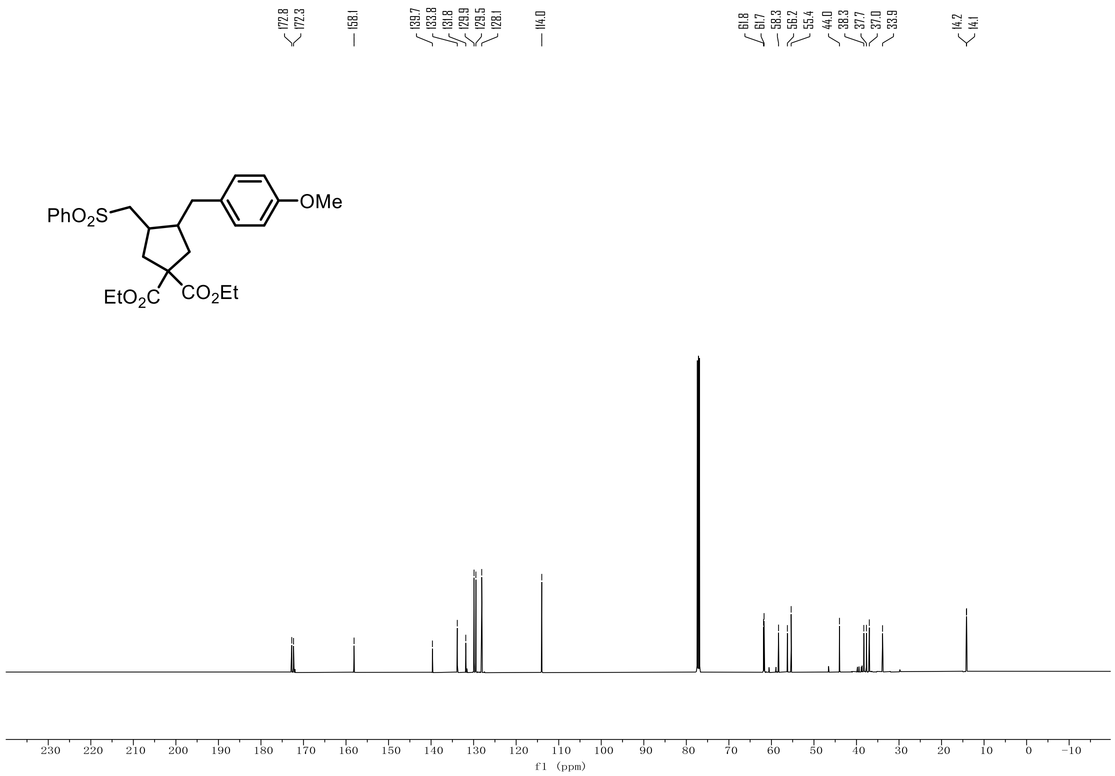

Figure S8. ^{1}H NMR and ^{13}C NMR spectrum of **63**

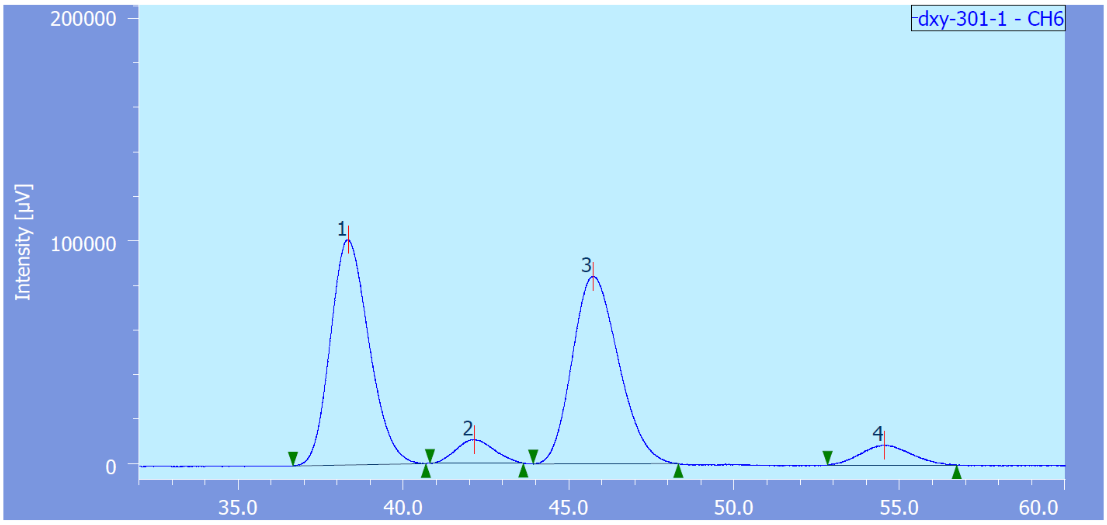

#	Peak Name	CH	tR [min]	Area [µV·sec]	Height [µV]	Area%	Height%	Quantity	NTP	Resolution	Symmetry Factor	Warning
1	Unknown	6	38.333	8235129	101267	45.129	49.388	N/A	5103	1.759	1.176	
2	Unknown	6	42.140	860768	10679	4.717	5.208	N/A	5904	1.505	1.090	
3	Unknown	6	45.727	8206036	84243	44.970	41.086	N/A	5000	3.227	1.202	
4	Unknown	6	54.543	945975	8853	5.184	4.318	N/A	5693	N/A	1.085	

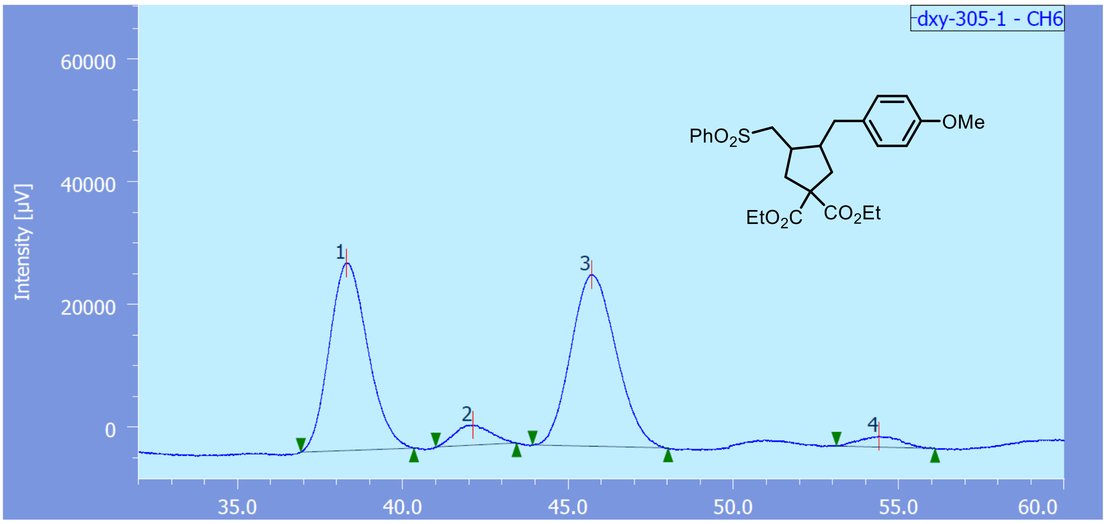

#	Peak Name	CH	tR [min]	Area [µV·sec]	Height [µV]	Area%	Height%	Quantity	NTP	Resolution	Symmetry Factor	Warning
1	Unknown	6	38.297	2473006	30552	44.626	48.058	N/A	5095	1.794	1.187	
2	Unknown	6	42.123	251046	3282	4.530	5.163	N/A	6247	1.532	1.053	
3	Unknown	6	45.703	2660235	27924	48.005	43.924	N/A	5127	3.555	1.149	
4	Unknown	6	54.403	157304	1815	2.839	2.855	N/A	8548	N/A	1.151	

Figure S9. HPLC spectrum of **63**

4.5 Stern-Volmer quenching experiments

Stern-Volmer quenching experiments were carried out using a 0.033 mM solution of photocatalyst 4-CzIPN and variable concentrations (0.07, 0.13, 0.20, 0.27 mM) of N-vinylbenzamide, 4-iodoanisole and **L5**$NiBr_2$ in anhydrous CH_3CN. Samples consisting of noted concentration of quencher were prepared and degassed by sparging with argon for 10 minutes. The intensity of the emission peak at 566 nm (λex = 390 nm) expressed as the ratio I_0/I, where I_0 is the emission intensity of 4-CzIPN at 566 nm in the absence of a quencher and I is the observed intensity, as a function of the quencher concentration was measured.

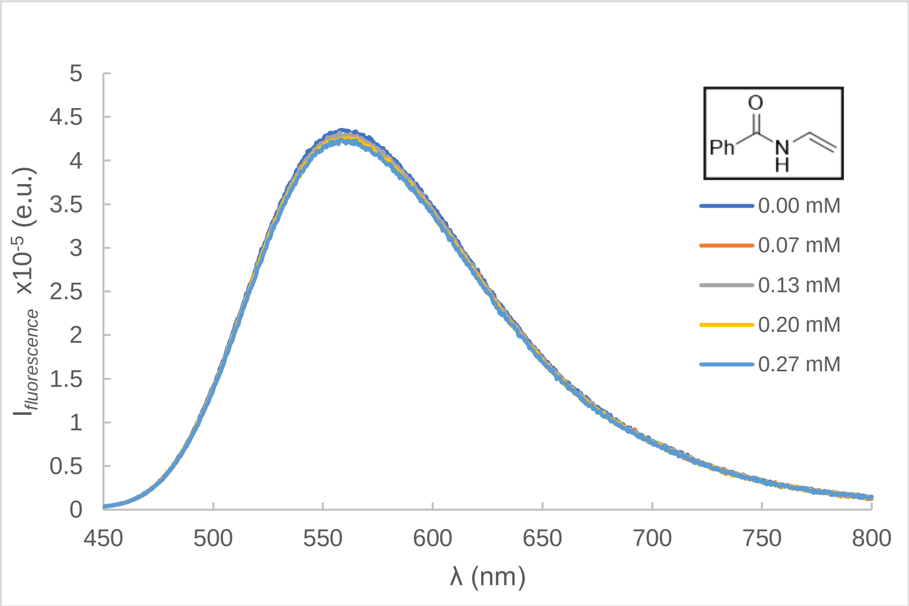

Figure S10. Emission spectra of 4-CzIPN (0.033 mM) at different concentrations of N-vinylbenzamide

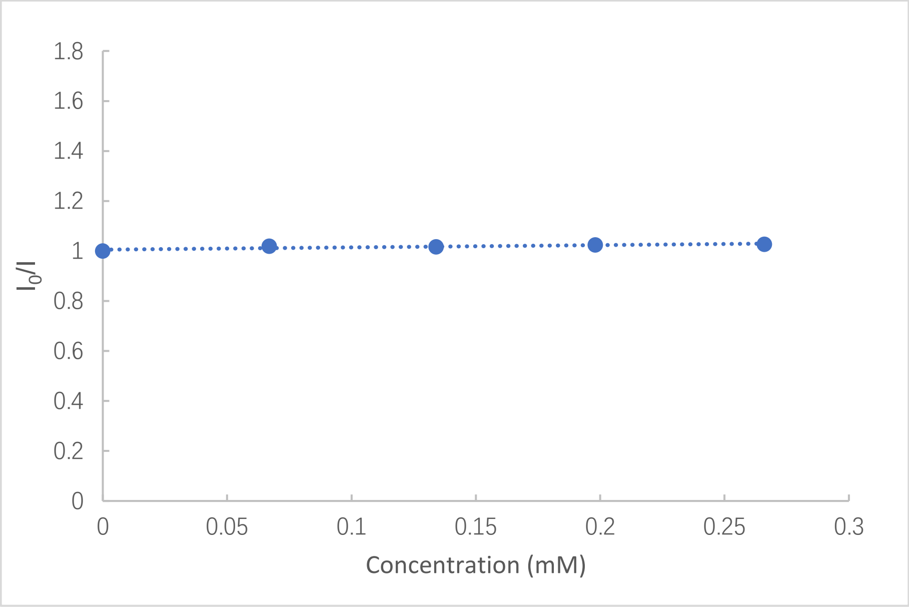

Figure S11. Stern-Volmer plot of 4-CzIPN (0.033 mM) at different concentrations of N-vinylbenzamide

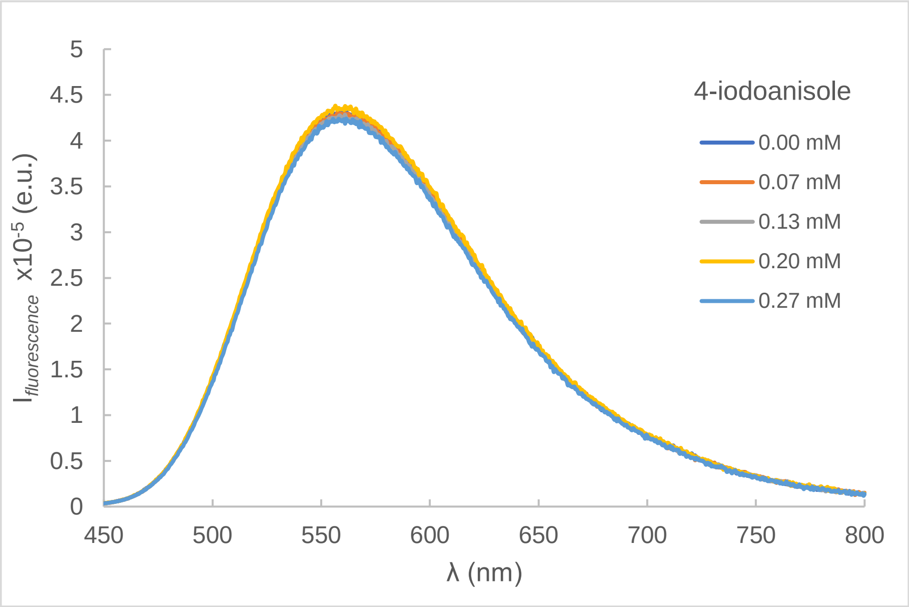

Figure S12. Emission spectra of 4-CzIPN (0.033 mM) at different concentrations of 4-iodoanisole

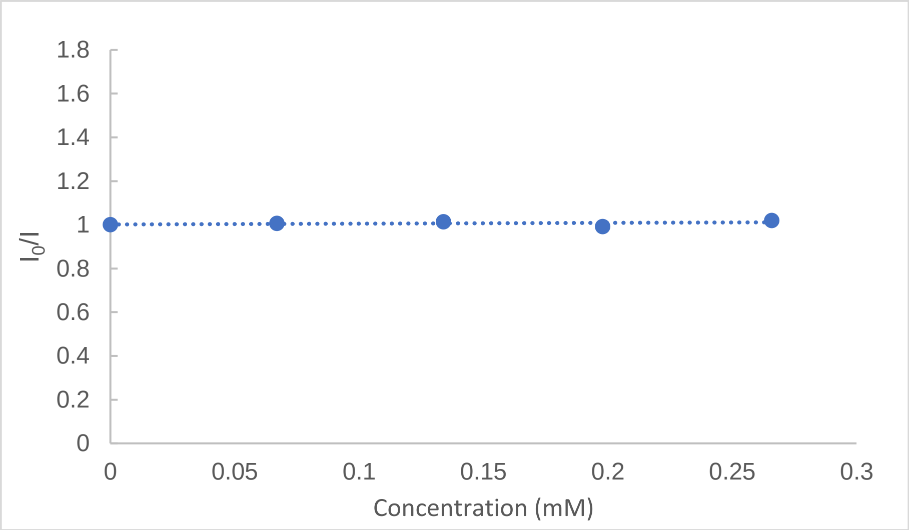

Figure S13. Stern-Volmer plot of 4-CzIPN (0.033 mM) at different concentrations of 4-iodoanisole

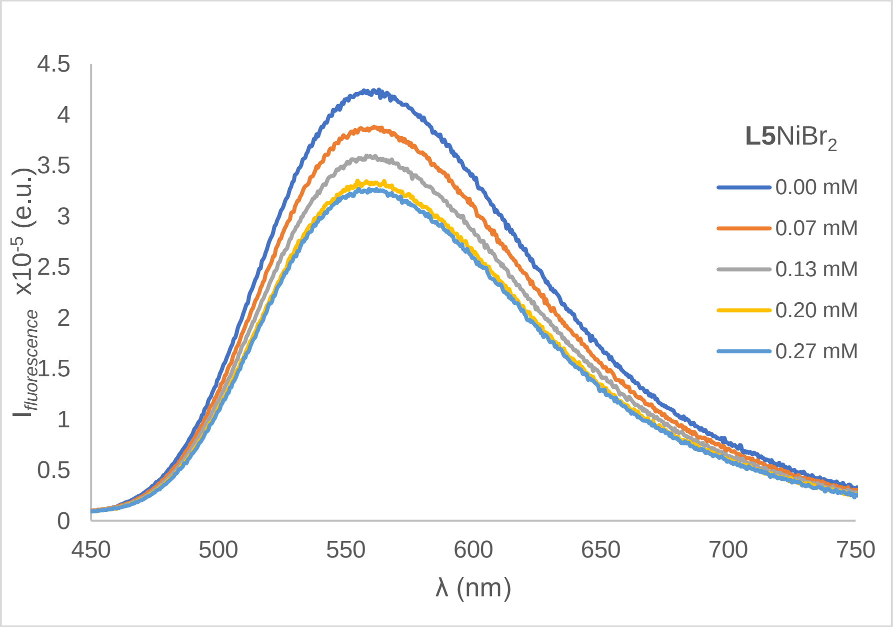

Figure S14. Emission spectra of 4-CzIPN (0.033 mM) at different concentrations of **L5**$NiBr_2$

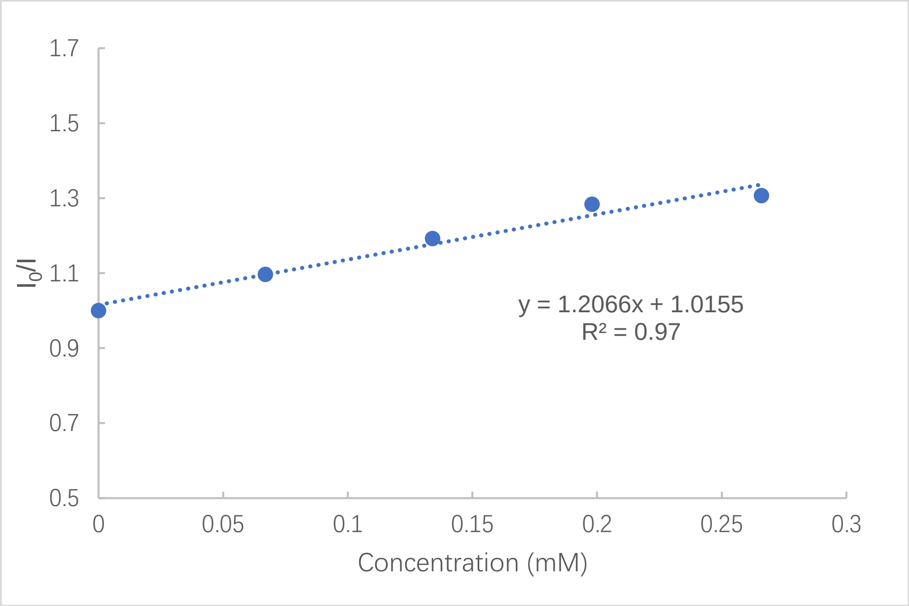

Figure S15. Stern-Volmer plot of 4-CzIPN (0.033 mM) at different concentrations of **L5**$NiBr_2$

Stern-Volmer quenching experiments were carried out using a 0.007 mM solution of photocatalyst 4-CzIPN and variable concentrations (0.07, 0.20, 0.33, 0.46 mM) of sodium benzenesulfinate in anhydrous DMF. Samples consisting of noted concentration of quencher were prepared and degassed by sparging with argon for 10 minutes. The intensity of the emission peak at 555 nm (λex = 370 nm) expressed as the ratio I_0/I, where I_0 is the emission intensity of 4-CzIPN at 555 nm in the absence of a quencher and I is the observed intensity, as a function of the quencher concentration was measured.

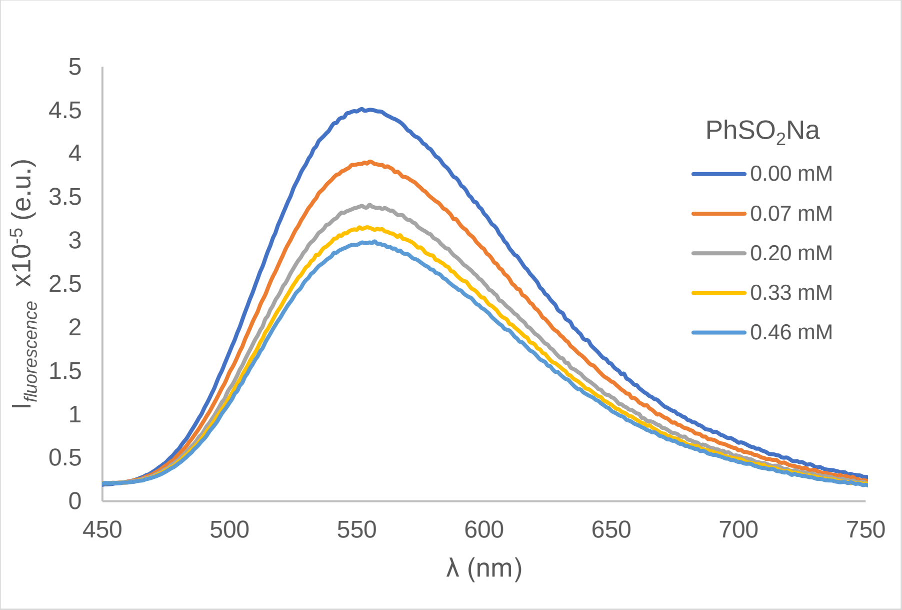

Figure S16. Emission spectra of 4-CzIPN (0.007 mM) at different concentrations of sodium benzenesulfinate

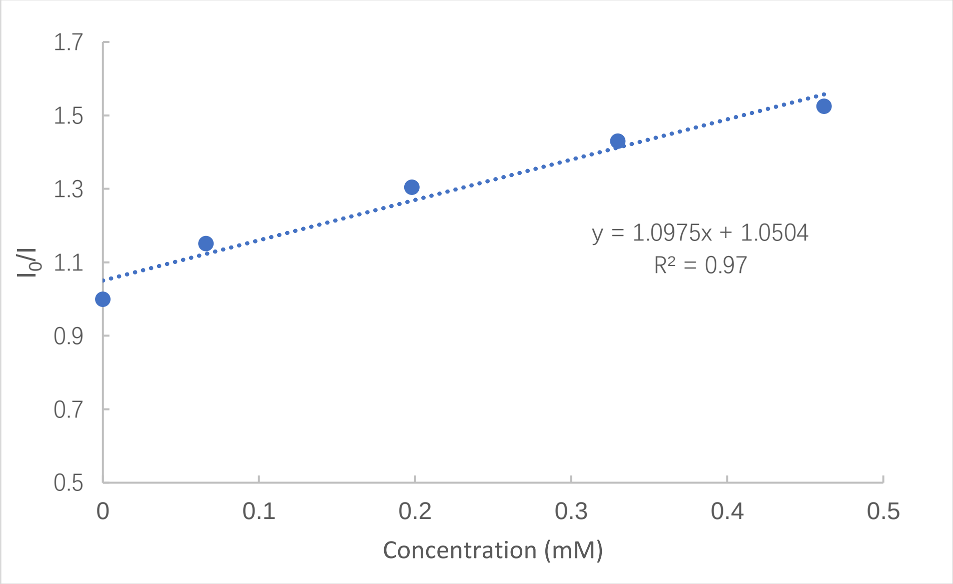

Figure S17. Stern-Volmer plot of 4-CzIPN (0.007 mM) at different concentrations of sodium benzenesulfinate

4.6 Reaction with Ar-Ni(II)-I complex.

Synthesis of Ar-Ni(II)-I complex

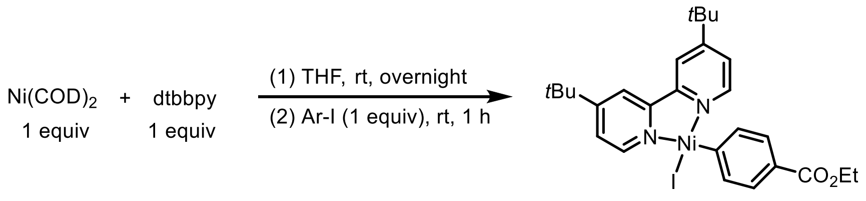

In a glove box, a suspension of Ni(cod)$_2$ (138 mg, 0.5 mmol, 1 equiv) in 3 mL of dry THF was stirred for 1 minutes in a 20 mL oven-dried 20 mL screw-cap vial, at which point a solution of dtbbpy (134 mg, 0.5 mmol, 1 equiv) in 2 mL of dry THF was added dropwise. The resulting mixture was allowed to stir overnight at ambient temperature. Ethyl 4-iodobenzoate (83.7 uL, 0.5 mmol, 1 equiv) was added via syringe. The resultant mixture was allowed to stir for another 1 h. The solvent was removed under vacuum, and the residue was filtrated with a fritted funnel. The solid was washed with ether (3×3mL), pentane (3×3mL) and dried in vacuum. Complex **65** was obtained as orange -red solid in 76% yield (230 mg), which was stored in the glove box at -20 °C. ^{1}H NMR (400 MHz, CD_2Cl_2) δ 9.51 (s, 1H), 7.82 (d, *J* = 10.4 Hz, 2H), 7.75 (d, *J* = 8.1 Hz, 2H), 7.52 (d, *J* = 7.9 Hz, 2H), 7.43 (d, *J* = 5.1 Hz, 1H), 7.15 (s, 1H), 6.86 (d, *J* = 6.8 Hz, 1H), 4.28 (q, *J* = 7.4 Hz, 2H), 1.39 – 1.30 (m, 21H). IR (film): ν (cm^{-1}) 1738 (ester C=O υ), 1687, 1411 (aromatic C-C υ), 1270, 1170, 1012 (aromatic C-H δ), 923, 880, 760 (*t*-Bu C-H δ).

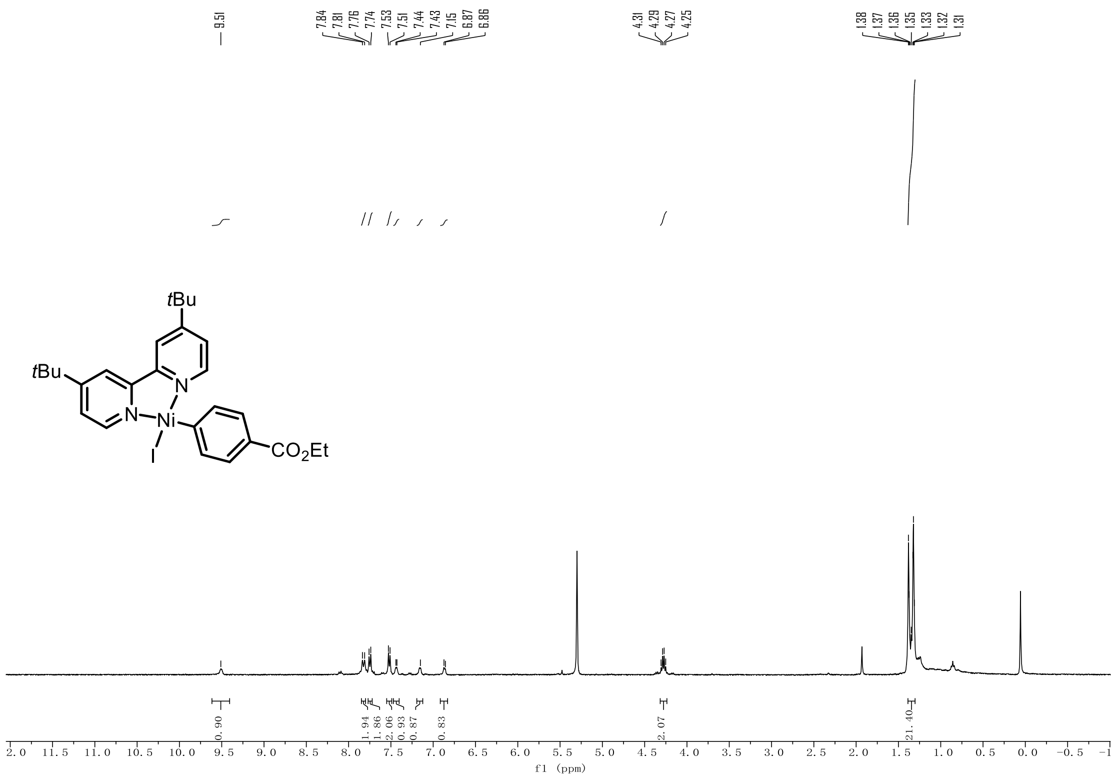

Figure S18. ^{1}H NMR spectrum of **65**

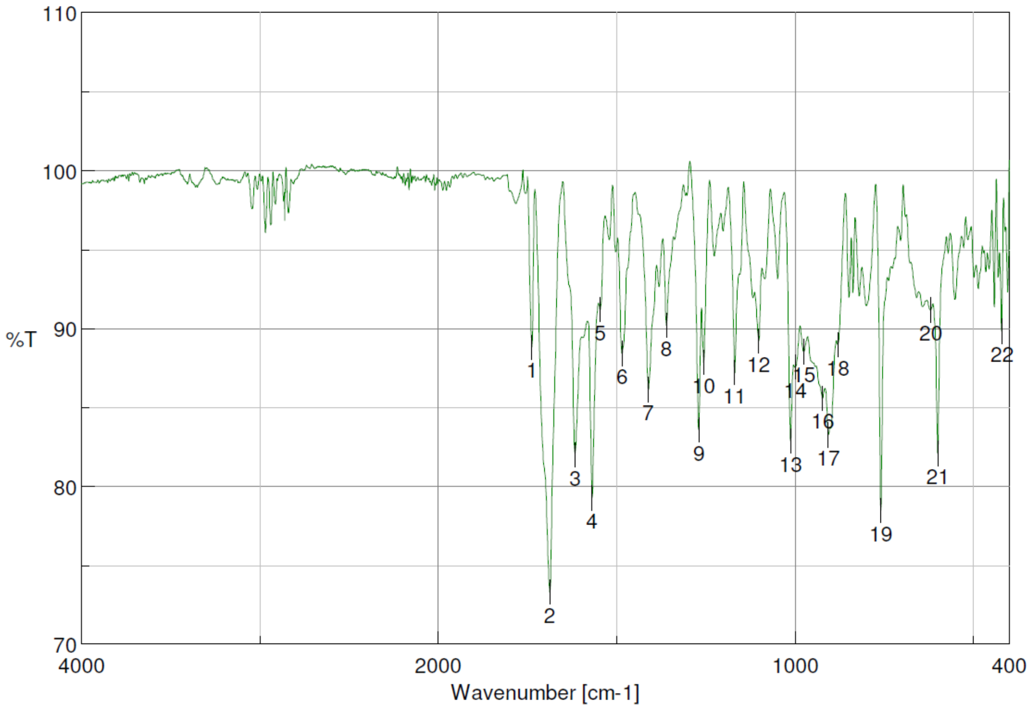

Figure S19. IR spectrum of **65**

Stoichiometric reaction with Ar-Ni(II)-I complex

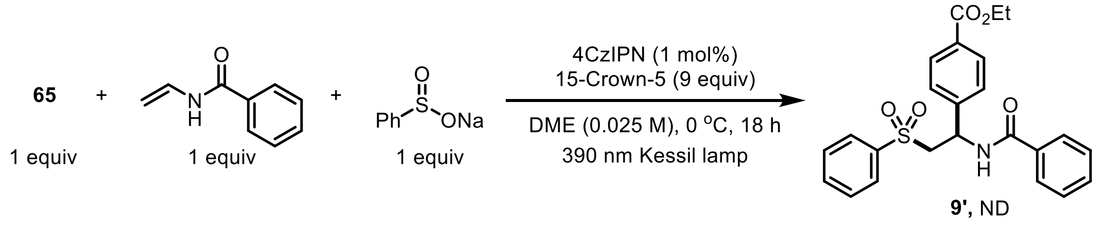

An oven-dried 7.5 mL screw-cap vial equipped with a magnetic stirring bar was charged with the vinyl amide (0.03 mmol, 1equiv), $PhSO_2Na$ (0.03 mmol, 1 equiv) and 4-CzIPN (0.0003 mmol, 1 mol%) and then introduced into a glove box filled with nitrogen. There, **65** (0.03 mmol, 1 equiv) was added in the vial followed by dry DME (1.2 mL) and 15-crown-5 (0.27 mmol, 9 equiv). The reaction vessel was then capped and removed from the glove box. The reaction was stirred (1000 rpm) under irradiation with 390 nm kessil lamp at 0 °C for 18 h. After that, the reaction mixture was concentrated in rotary evaporator and the residue was analyzed by ^{1}H NMR with 1,3,5-Trimethoxybenzene (0.01 mmol) as internal standard (Figure S20). The crude ^{1}H NMR showed no product generated. And after calculation, it is revealed that the vinyl amide remained there without consumption. This result revealed that Ni(II)–aryl species may not be involved in the catalytic cycle, deeming a Ni(0)/Ni(II)/Ni(III) catalytic cycle unlikely.

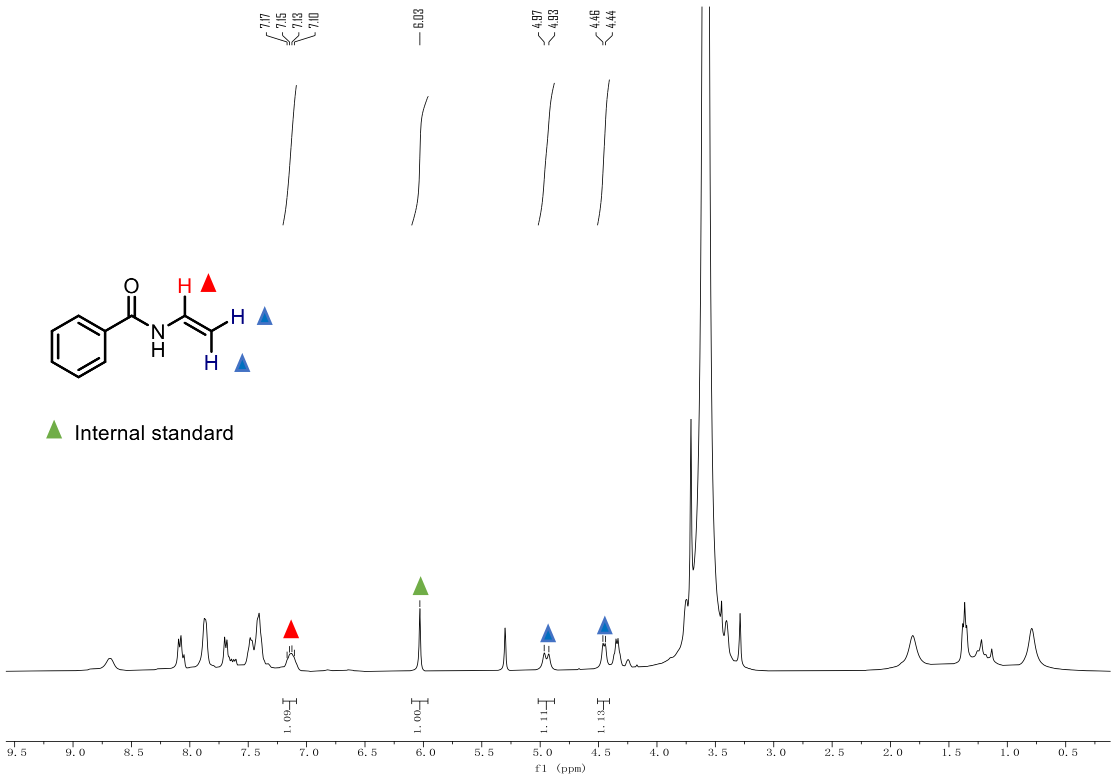

Figure S20. ^{1}H NMR spectrum of crude reaction mixture with internal standard.

Cross-over reaction with catalytic amount of Ar-Ni(II)-I complex

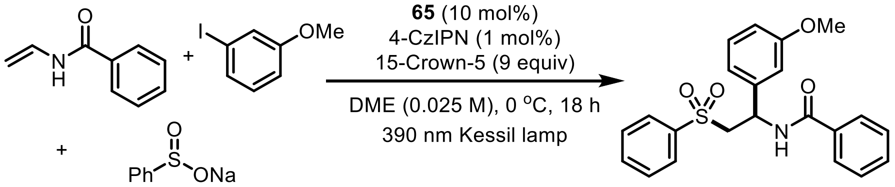

An oven-dried 7.5 mL screw-cap vial equipped with a magnetic stirring bar was charged with the vinyl amide (0.2 mmol, 2 equiv), $PhSO_2Na$ (0.2 mmol, 2 equiv), 4-CzIPN (0.001 mmol, 1 mol%) and **65** (0.01 mmol, 10 mol%) and then introduced into a glove box filled with nitrogen. There, dry DME (4 mL) and 15-crown-5 (0.9 mmol, 9 equiv) were sequentially added. The reaction vessel was then capped and removed from the glove box. After that, 3-iodoanisole (0.1 mmol, 1 equiv) was added. The reaction was stirred (1000 rpm) under irradiation with 390 nm kessil lamp at 0 °C for 48 h. After that, the reaction mixture was concentrated in rotary evaporator and the residue was analyzed by ^{1}H NMR with mesitylene (0.043 mmol) as internal standard (Figure S21).

The corresponding racemic product **14'** could be obtained in 45% yield. This results revealed that such Ni(II) complex **65** could generate Ni(I) species through disproportionation and then enter the cycle again.

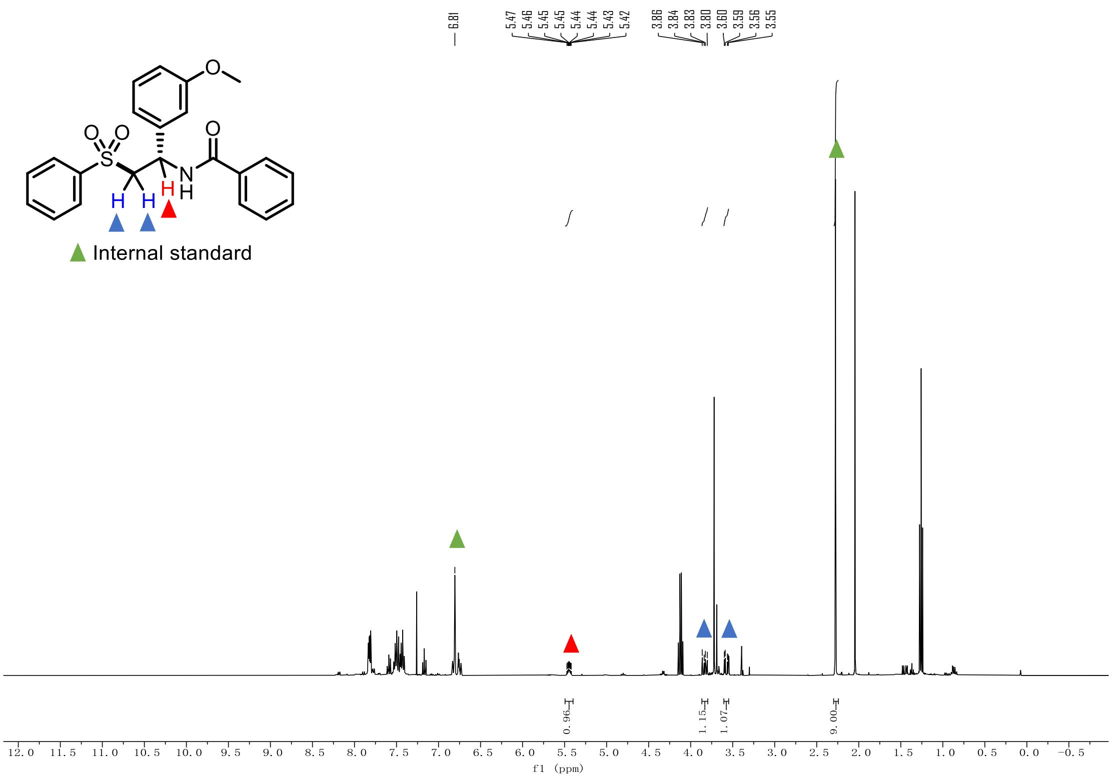

Figure S21. ^{1}H NMR spectrum of crude reaction mixture with internal standard.

Control experiment using dtbbpy ligand

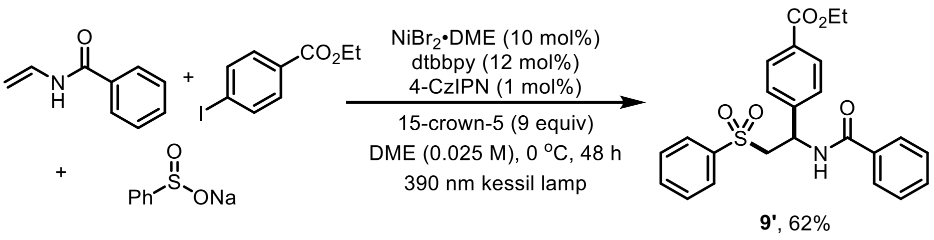

An oven-dried 7.5 mL screw-cap vial equipped with a magnetic stirring bar was charged with the vinyl amide (0.2 mmol, 2 equiv), $PhSO_2Na$ (0.2 mmol, 2 equiv), 4-CzIPN (0.001 mmol, 1 mol%), $NiBr_2$·DME (0.01 mmol, 10 mol%) and dtbbpy (0.012 mmol, 12 mol%) and then introduced into a glove box filled with nitrogen. There, dry DME (4 mL) and 15-crown-5 (0.9 mmol, 9 equiv) were sequentially added. The

reaction vessel was then capped and removed from the glove box. After that, ethyl 4-iodobenzoate (0.1 mmol, 1 equiv) was added. The reaction was stirred (1000 rpm) under irradiation with 390 nm kessil lamp at 0 °C for 48 h. After that, the reaction mixture was concentrated in vacuum and the residue was analyzed by ^{1}H NMR with 1,3,5-Trimethoxybenzene (0.04 mmol) as internal standard. The corresponding racemic arylsulfonylated product **9'** could be obtained in 62% yield. This result highlights that the lack of reactivity observed for **65** in terms of aryl group transfer is not due to the use of a dtbbpy ligand itself.

Control experiment using $Ni(COD)_2$

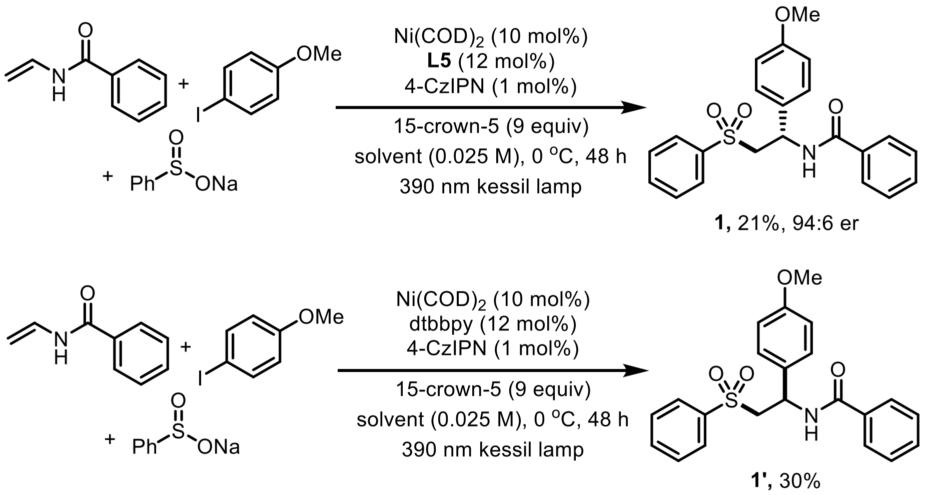

An oven-dried 7.5 mL screw-cap vial equipped with a magnetic stirring bar was charged with the vinyl amide (0.2 mmol, 2 equiv), $PhSO_2Na$ (0.2 mmol, 2 equiv), 4-iodoanisole (0.1 mmol, 1 equiv), 4-CzIPN (0.001 mmol, 1 mol%), $Ni(COD)_2$ (0.01 mmol, 10 mol%) and **L5** or dtbbpy (0.012 mmol, 12 mol%) and then introduced into a glove box filled with nitrogen. There, dry DME (4 mL) and 15-crown-5 (0.9 mmol, 9 equiv) were sequentially added. The reaction vessel was then capped and removed from the glove box. The reaction was stirred (1000 rpm) under irradiation with 390 nm kessil lamp at 0 °C for 48 h. After that, the reaction mixture was concentrated in vacuum and the residue was analyzed by ^{1}H NMR with mesitylene (0.043 mmol) as internal standard (0.04 mmol) as internal standard.

5. References

(1) For the synthesis of nickel complexes, see: Wei, X.; Shu, W.; Garcia-Dominguez, A.; Merino, E.; Nevado, C. Asymmetric Ni-Catalyzed Radical Relayed Reductive Coupling. *J. Am. Chem. Soc.* **2020**, *142*, 13515.

(2) Sun, R.; Yang, X.; Chen, X.; Zhang, C.; Zhao, X.; Wang, X.; Zheng, X.; Yuan, M.; Fu, H.; Li, R.; Chen, H. *Org. Lett.* **2018**, *20*, 6755.

(3) Cuesta-Galisteo, S.; Schoergenhumer, J.; Wei, X.; Merino, E.; Nevado, C. *Angew. Chem., Int. Ed.* **2021**, *60*, 1605.

(4) Archibald, S. C.; Fleming, I. *J. Chem. Soc., Perkin Trans. 1* **1993**, 751.

(5) Xu, Y.; Liu, X.-Y.; Wang, Z.-Q.; Tang, L.-F. *Tetrahedron Lett.* **2017**, *58*, 1788.

(6) Ribereau, P.; Delamare, M.; Celanire, S.; Queguiner, G. *Tetrahedron Lett.* **2001**, *42*, 3571.

(7) Neuschütz, K.; Simone, J-M.; Thyrann, T.; Neier, R. *Helv. Chim. Acta* **2000**, *83*, 2712.

(8) He, Y.; Du, C.; Han, J.; Han, J.; Zhu, C.; Xie, J. *Chin. J. Chem*. **2022**, *40*, 1546.

(9) Zhang, P.; Wang, C.; Cui, M.; Du, M.; Li, W.; Jia, Z.; Zhao, Q. *Org. Lett.* **2020**, *22*, 1149.

(10) Schneider, H. J.; Philippi, K. *Chem. Ber.* **1984**, *117*, 3056.

(11) Mukhopadhyay, S.; Batra, S. *Chem. - Eur. J.* **2018**, *24*, 14622.

(12) Zhu, C.; Yue, H.; Maity, B.; Atodiresei, I.; Cavallo, L.; Rueping, M. *Nat. Catal.* **2019**, *2*, 678.

6. NMR Spectra

(*S*)-*N*-(1-(4-Methoxyphenyl)-2-(phenylsulfonyl)ethyl)benzamide (1).

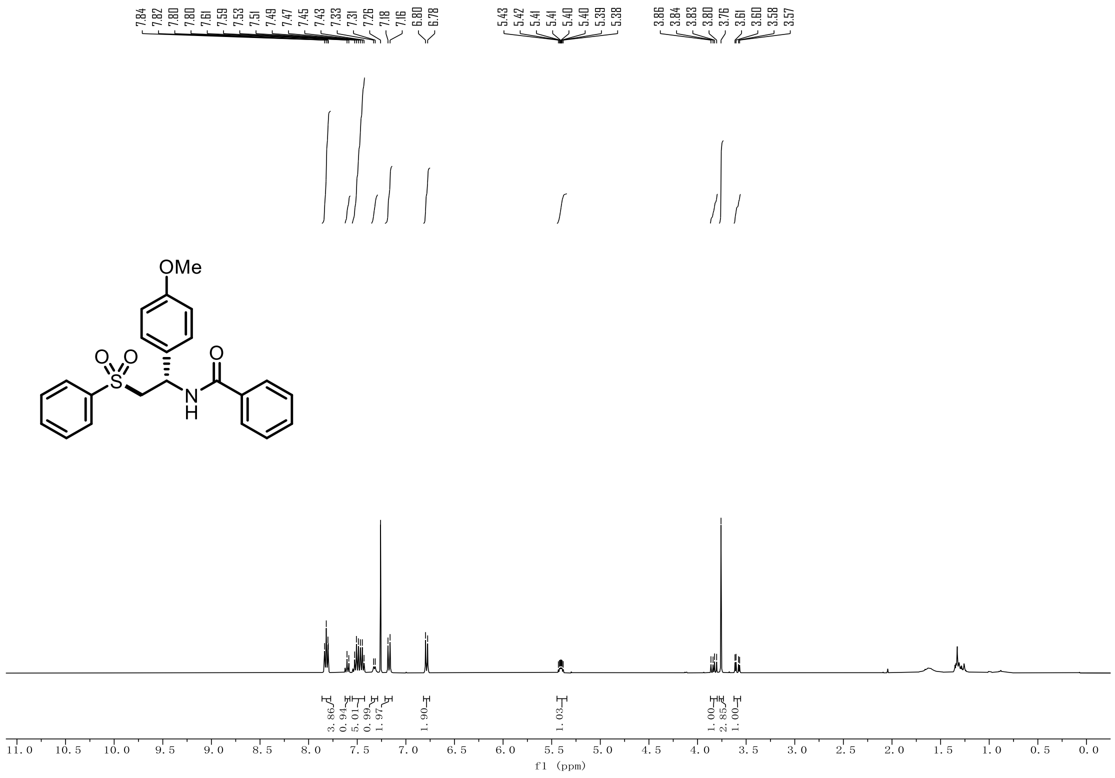

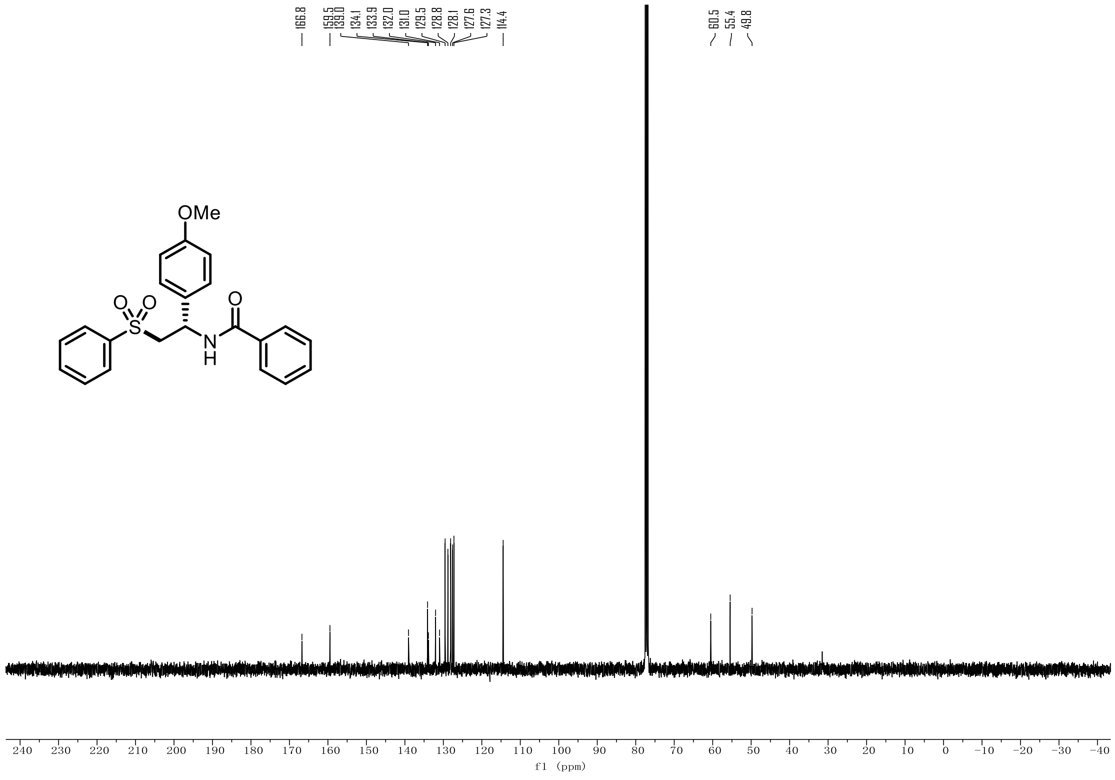

(*S*)-*N*-(2-(Phenylsulfonyl)-1-(*p*-tolyl)ethyl)benzamide (2).

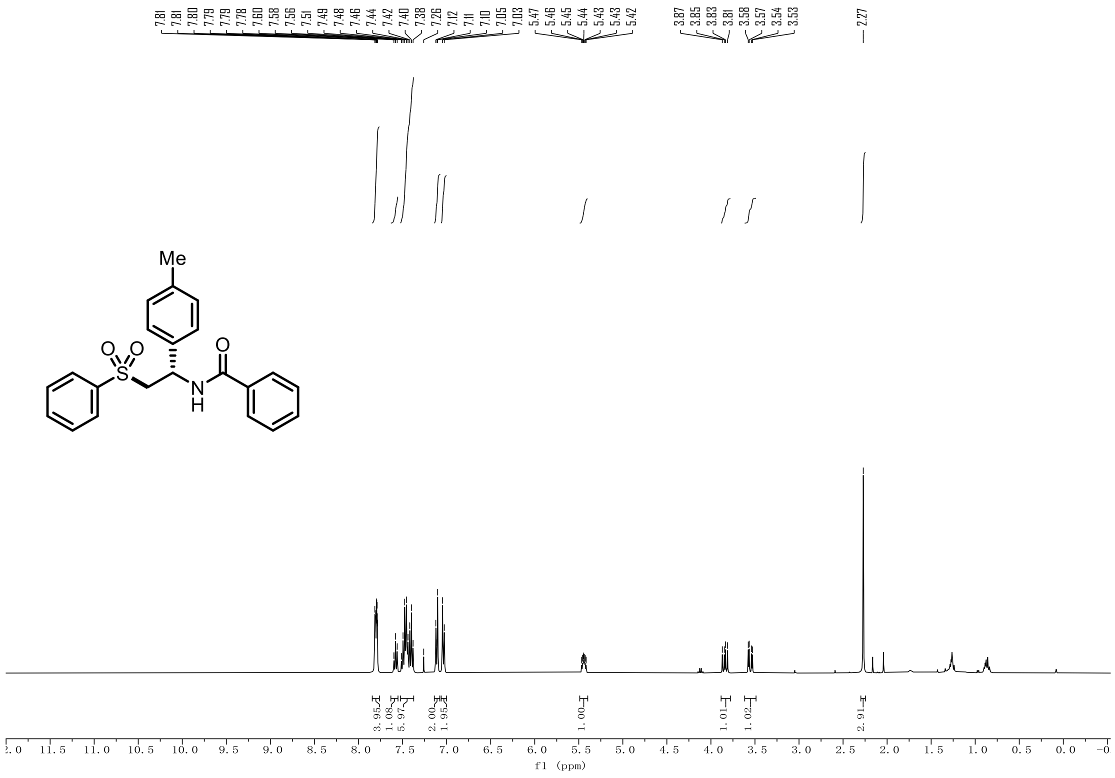

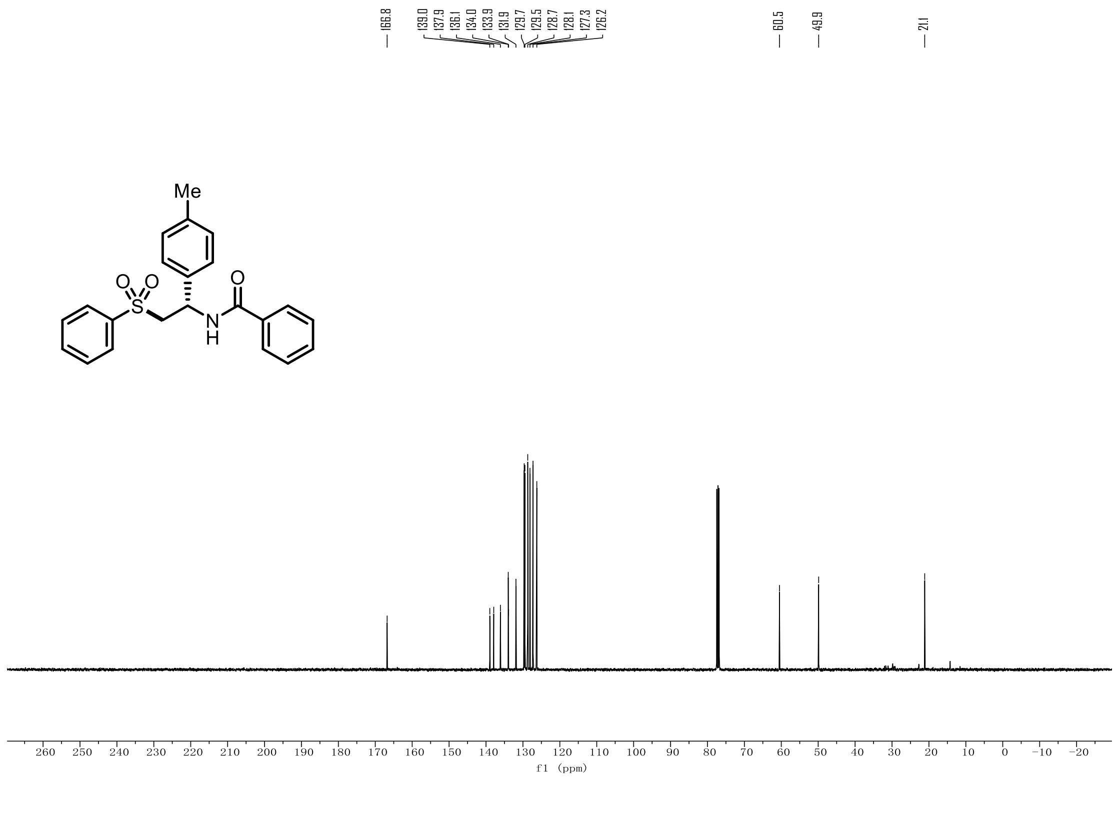

(*S*)-*N*-(1-(4-(Tert-butyl)phenyl)-2-(phenylsulfonyl)ethyl)benzamide (3).

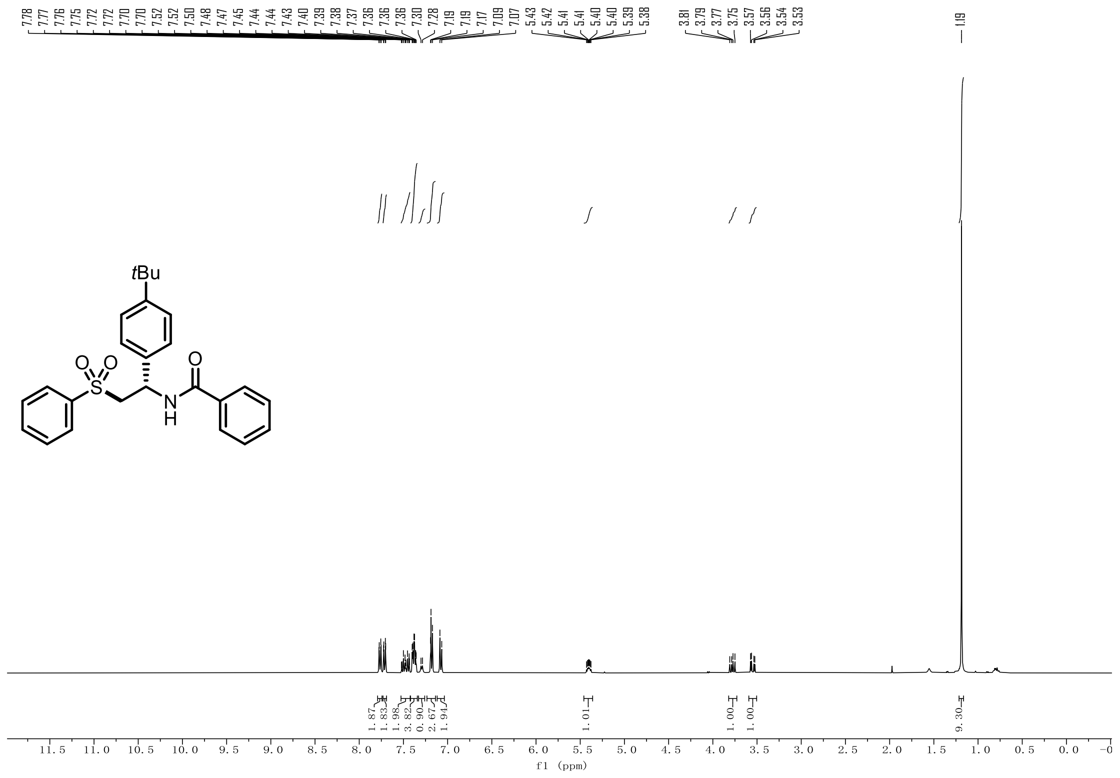

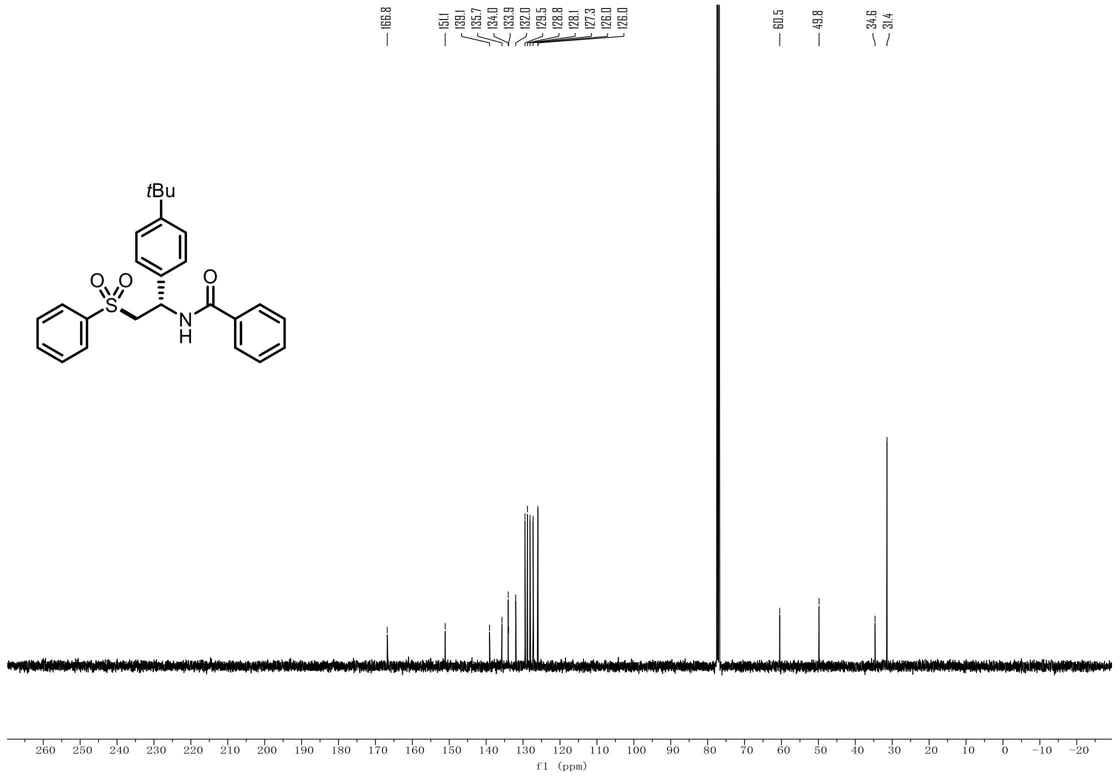

(*S*)-*N*-(1-([1,1'-Biphenyl]-4-yl)-2-(phenylsulfonyl)ethyl)benzamide (4).

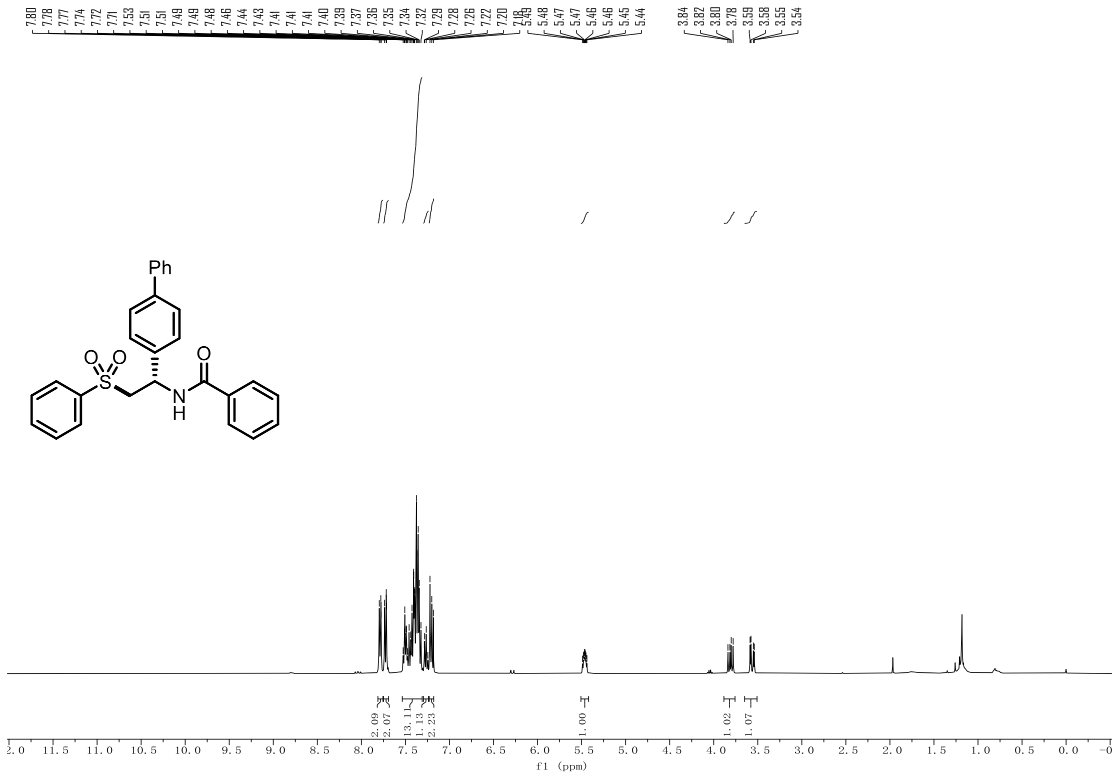

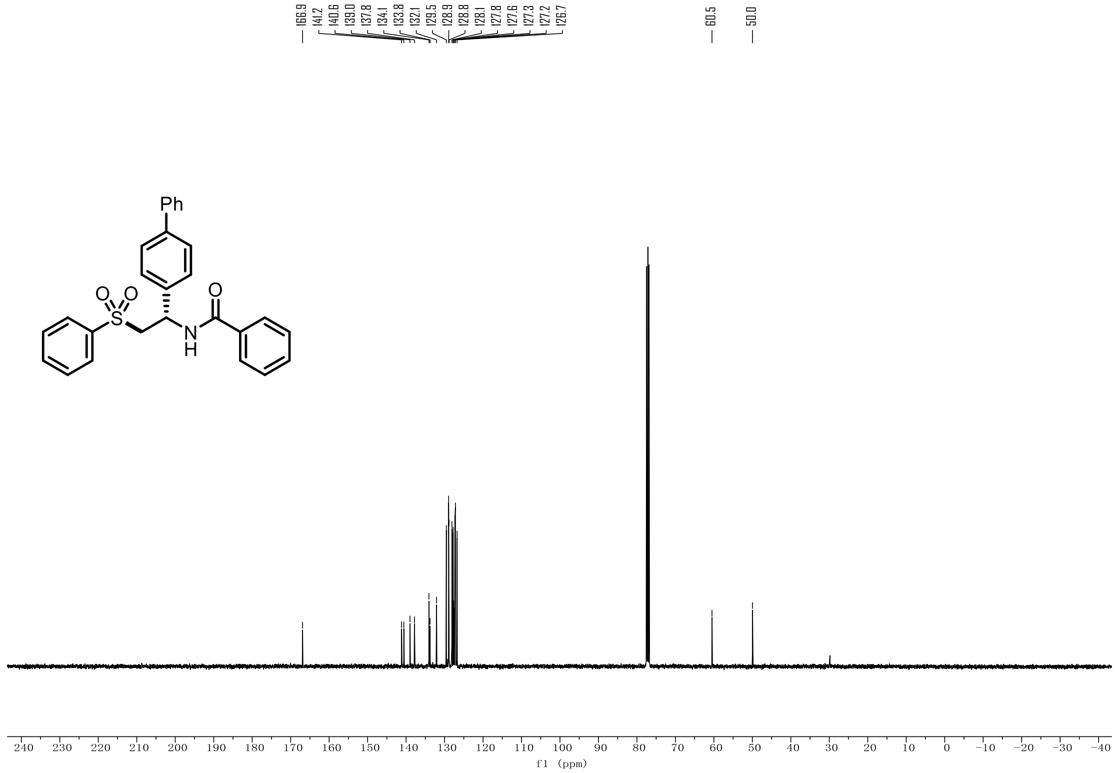

(*S*)-*N*-(1-(4-Fluorophenyl)-2-(phenylsulfonyl)ethyl)benzamide (5).

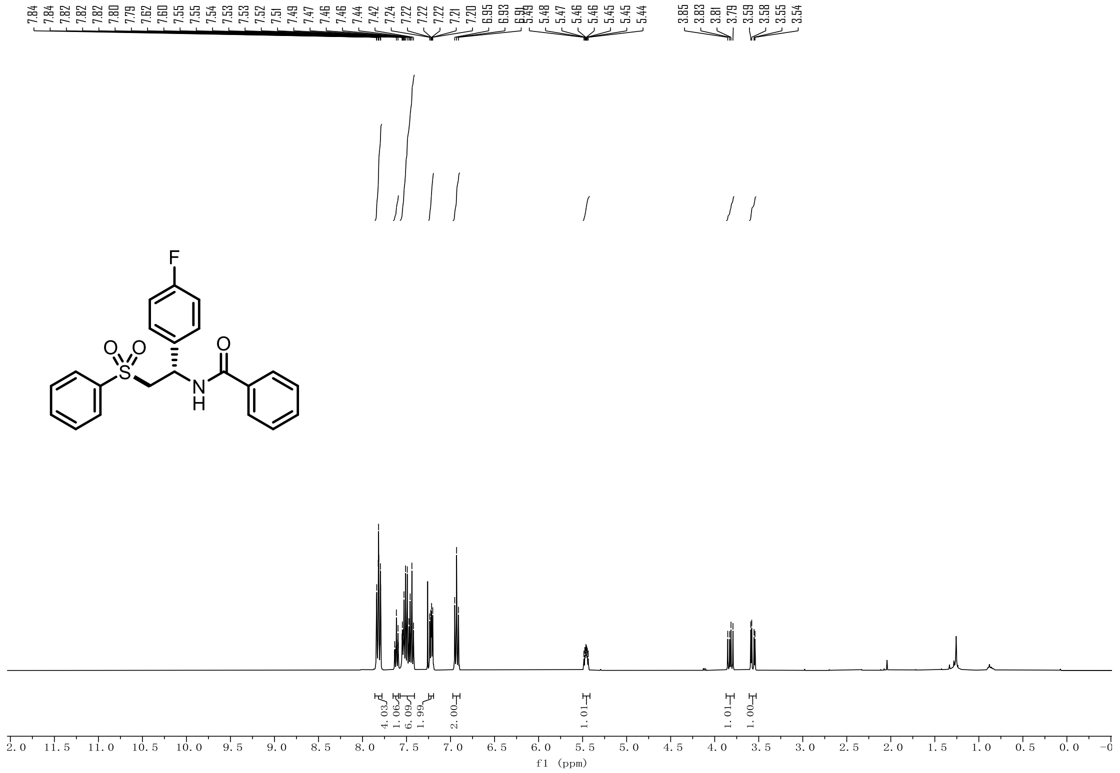

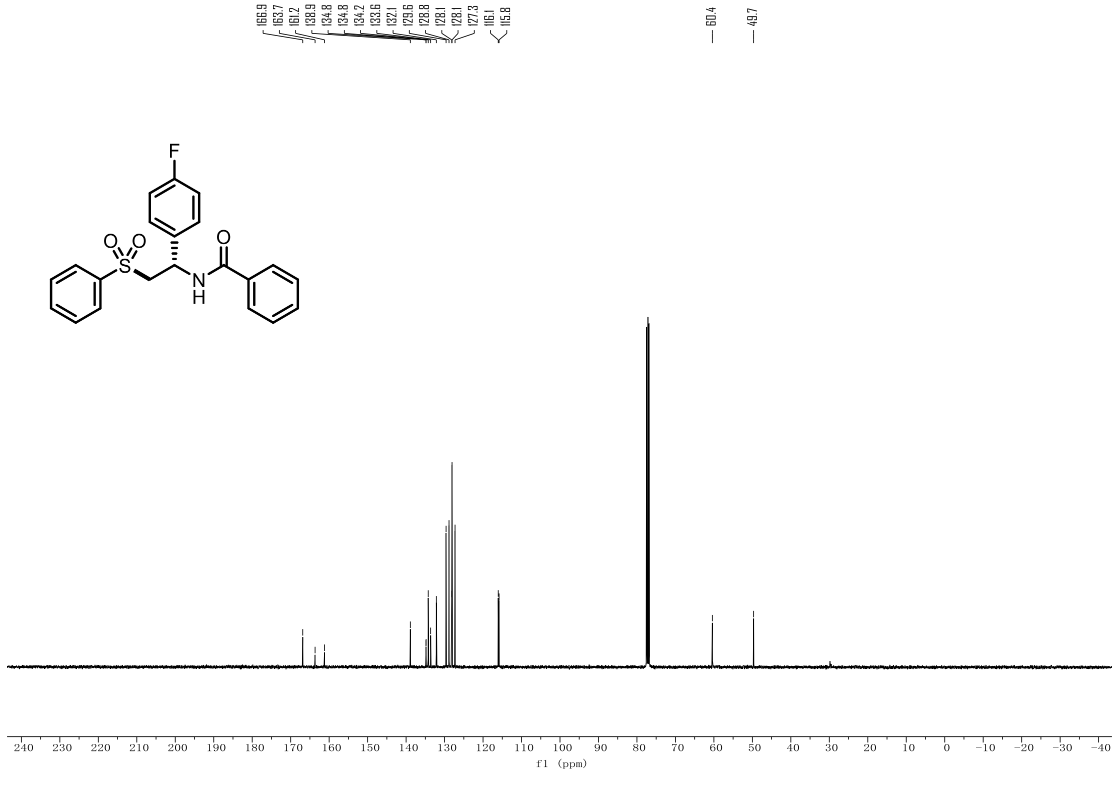

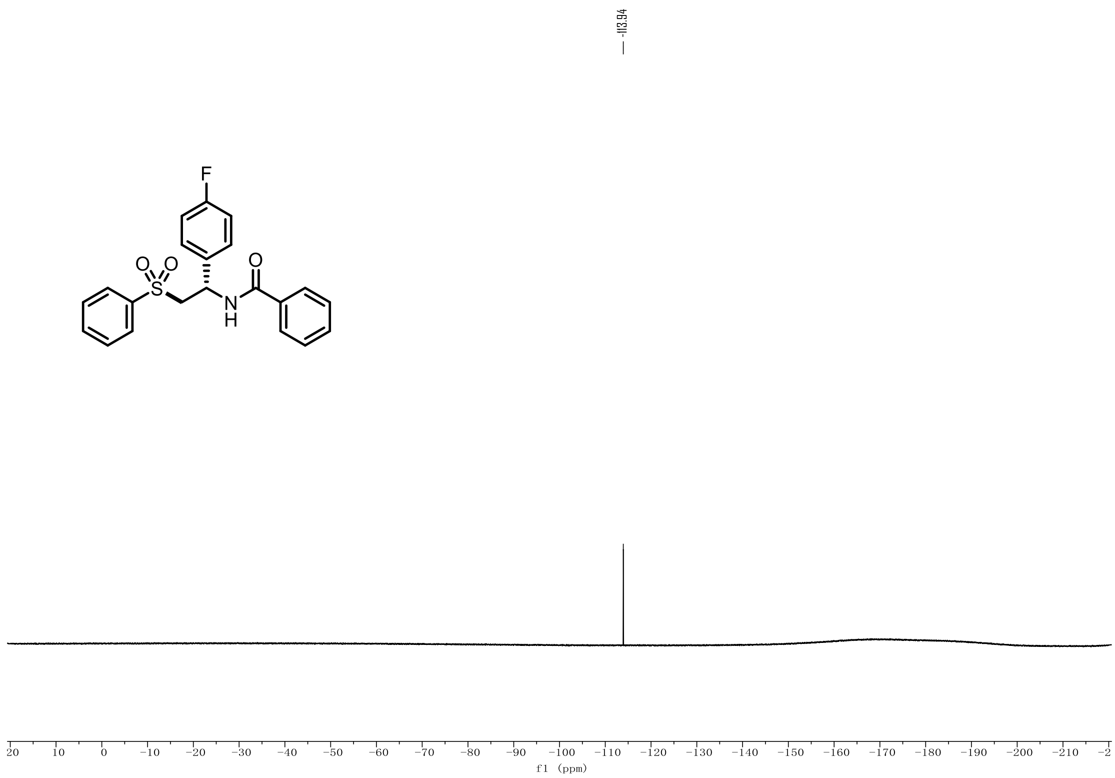

(*S*)-*N*-(1-(4-Chlorophenyl)-2-(phenylsulfonyl)ethyl)benzamide (6).

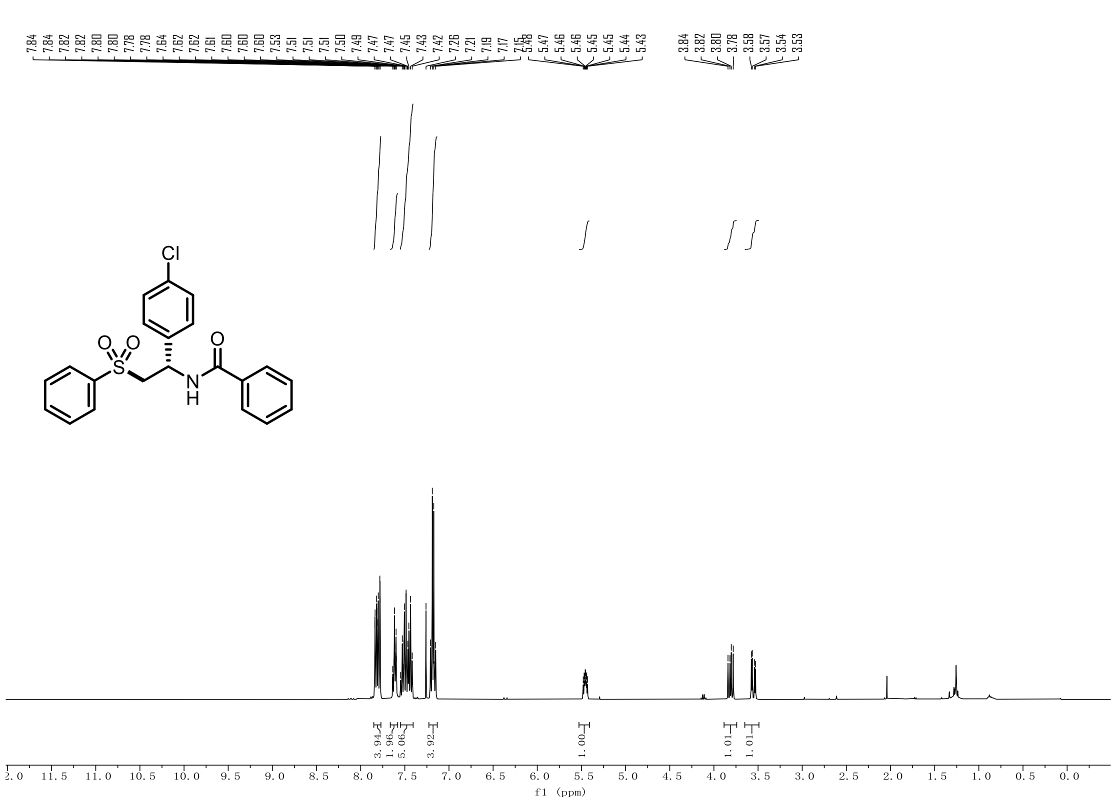

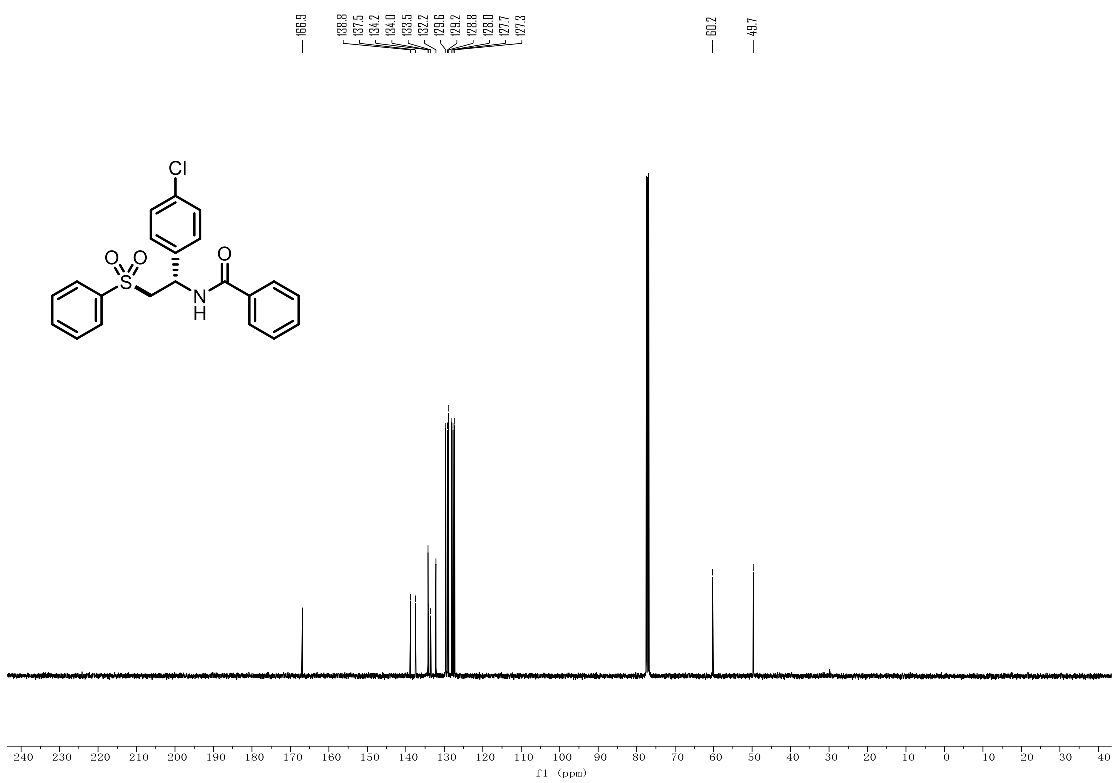

(*S*)-*N*-(2-(Phenylsulfonyl)-1-(4-(trifluoromethyl)phenyl)ethyl)benzamide (7).

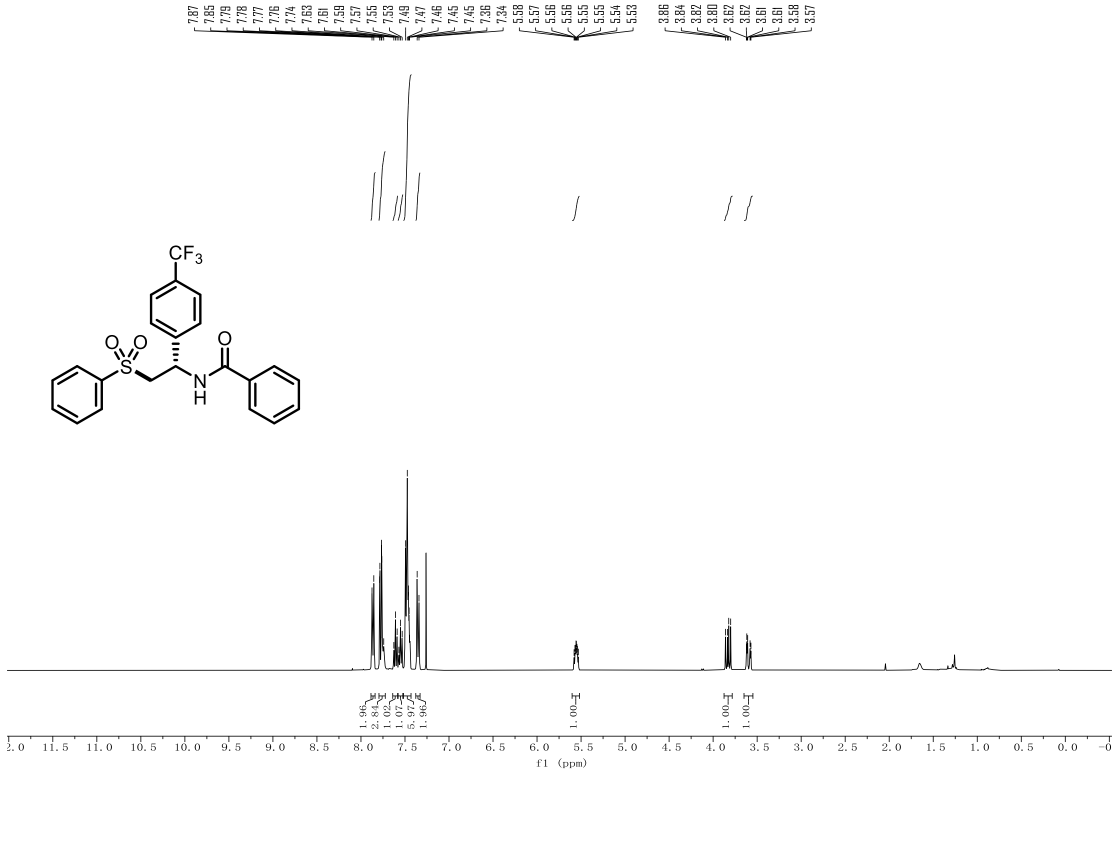

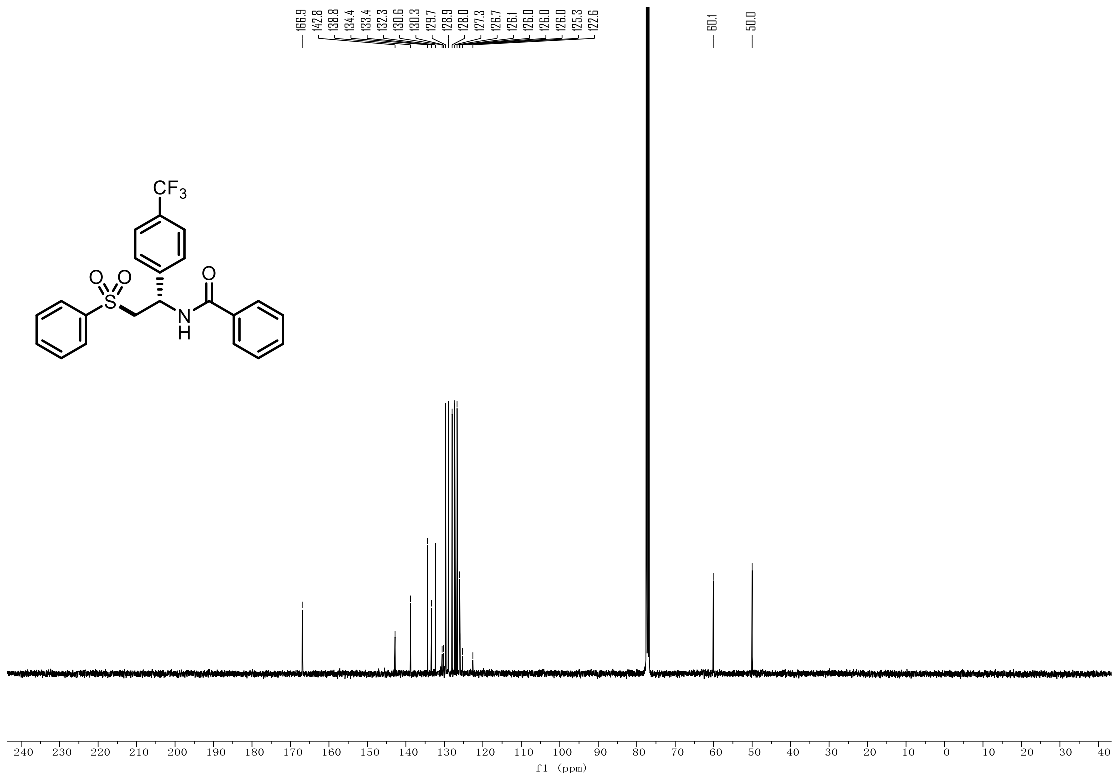

166.9
142.8
138.8
134.4
133.4
132.3
130.6
130.3
129.7
128.9
128.0
127.3
126.7
126.1
126.0
126.0
126.0
125.3
122.6
60.1
50.0
CF3
O
S
O
O
N
H
f1 (ppm)

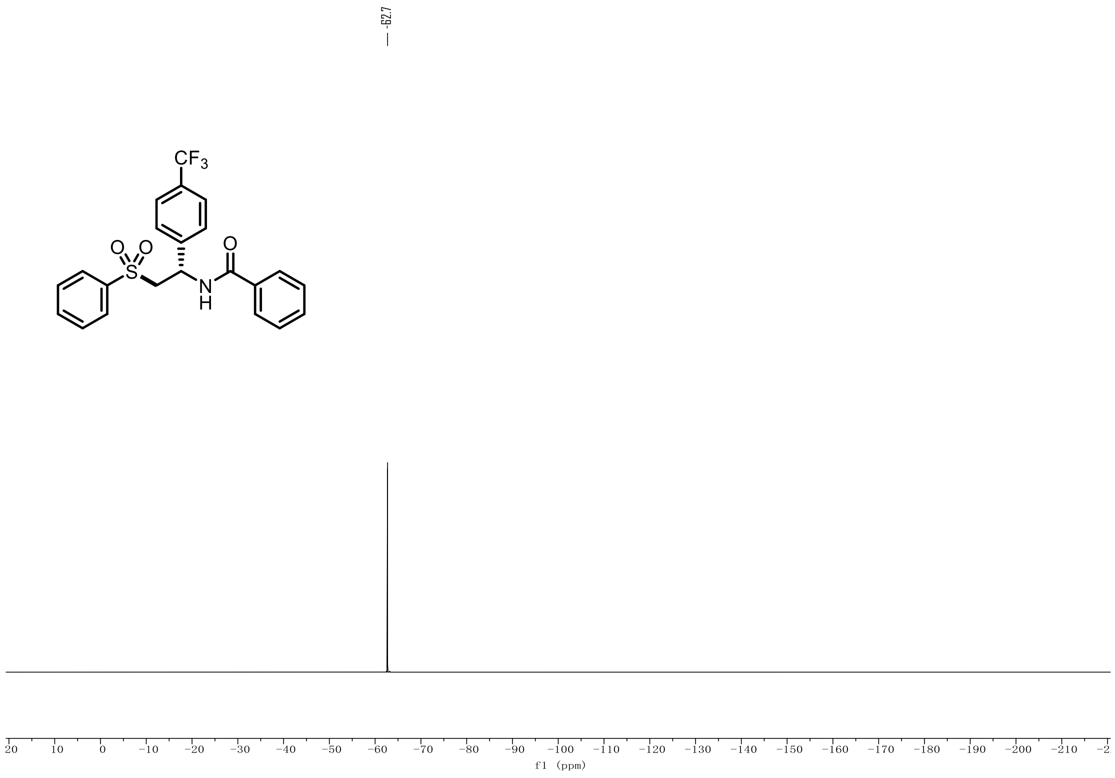

-62.7
CF3
O
S
O
O
N
H
f1 (ppm)

(*S*)-*N*-(1-(4-Cyanophenyl)-2-(phenylsulfonyl)ethyl)benzamide (8).

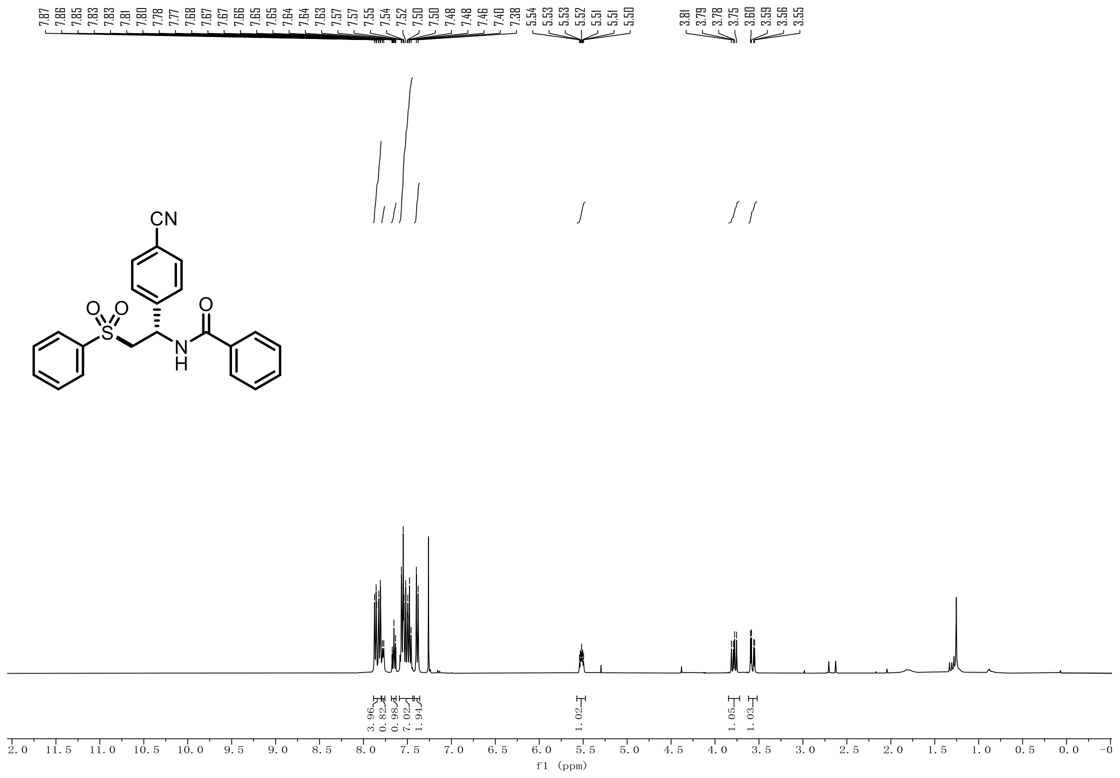

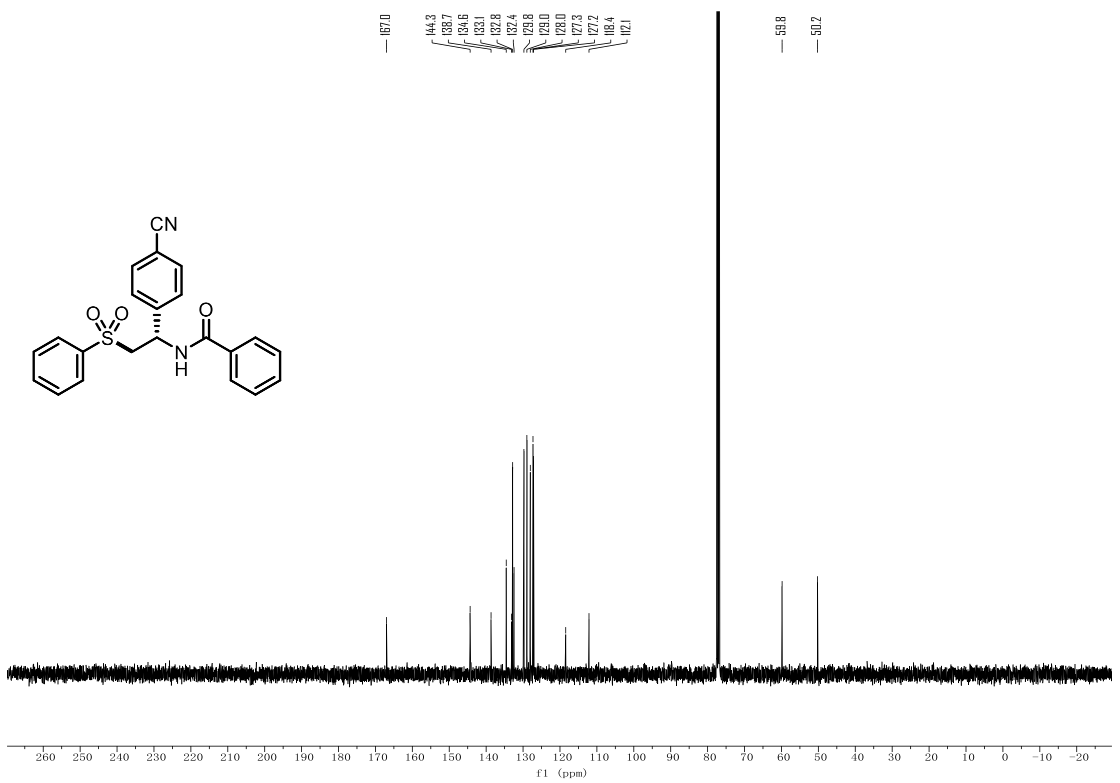

Ethyl (*S*)-4-(1-benzamido-2-(phenylsulfonyl)ethyl)benzoate (9).

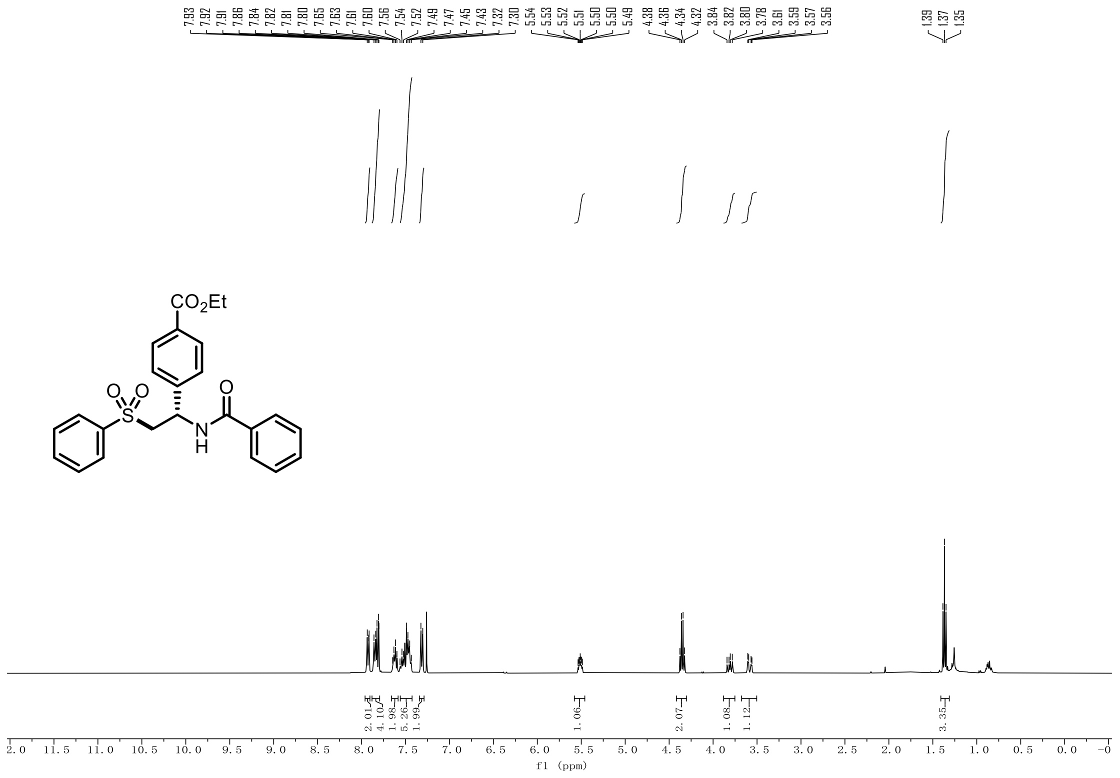

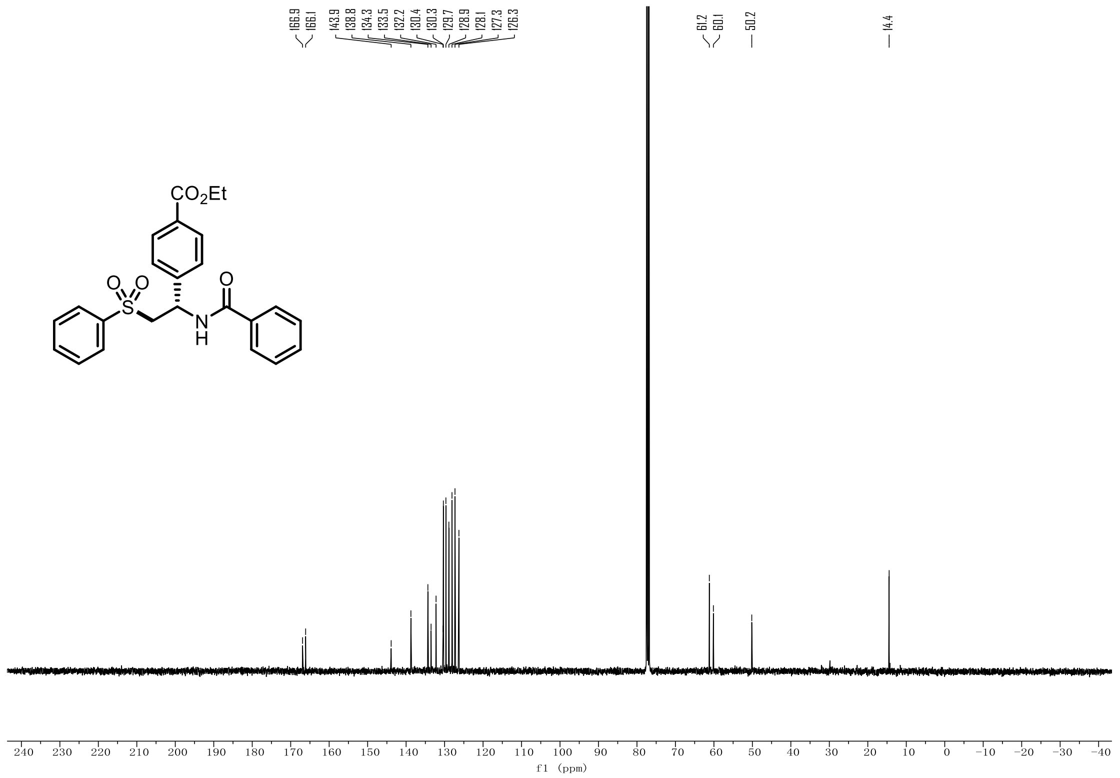

(*S*)-*N*-(1-(4-Acetylphenyl)-2-(phenylsulfonyl)ethyl)benzamide (10).

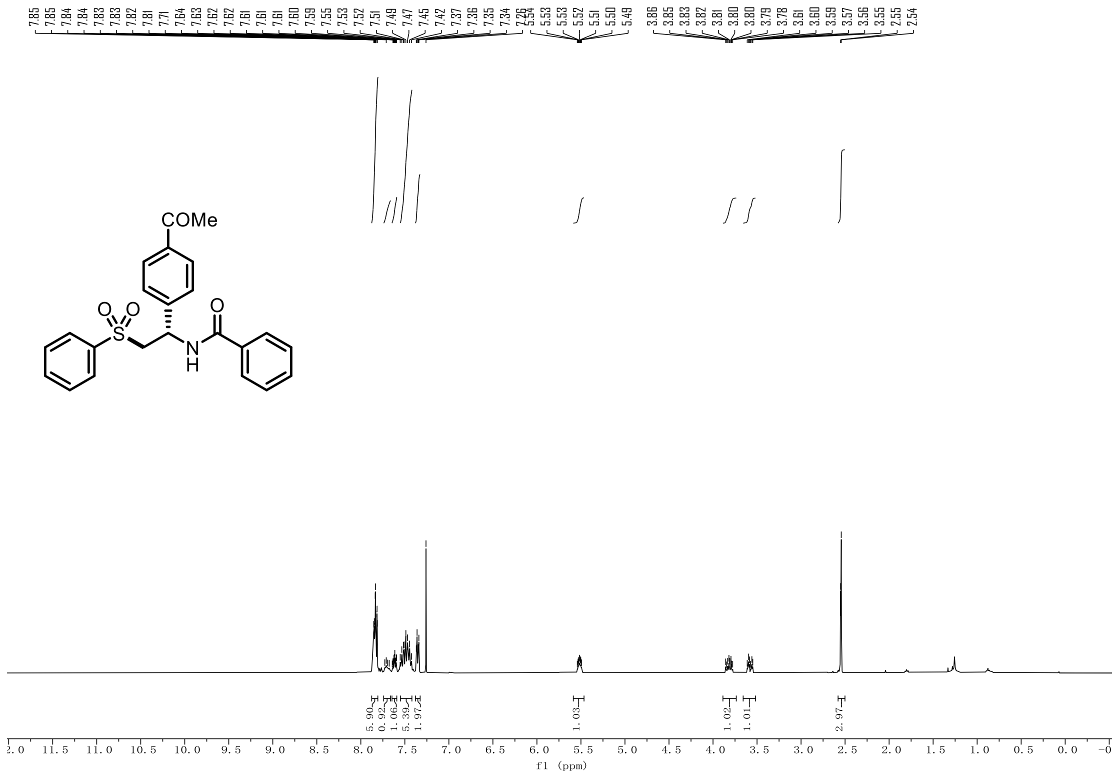

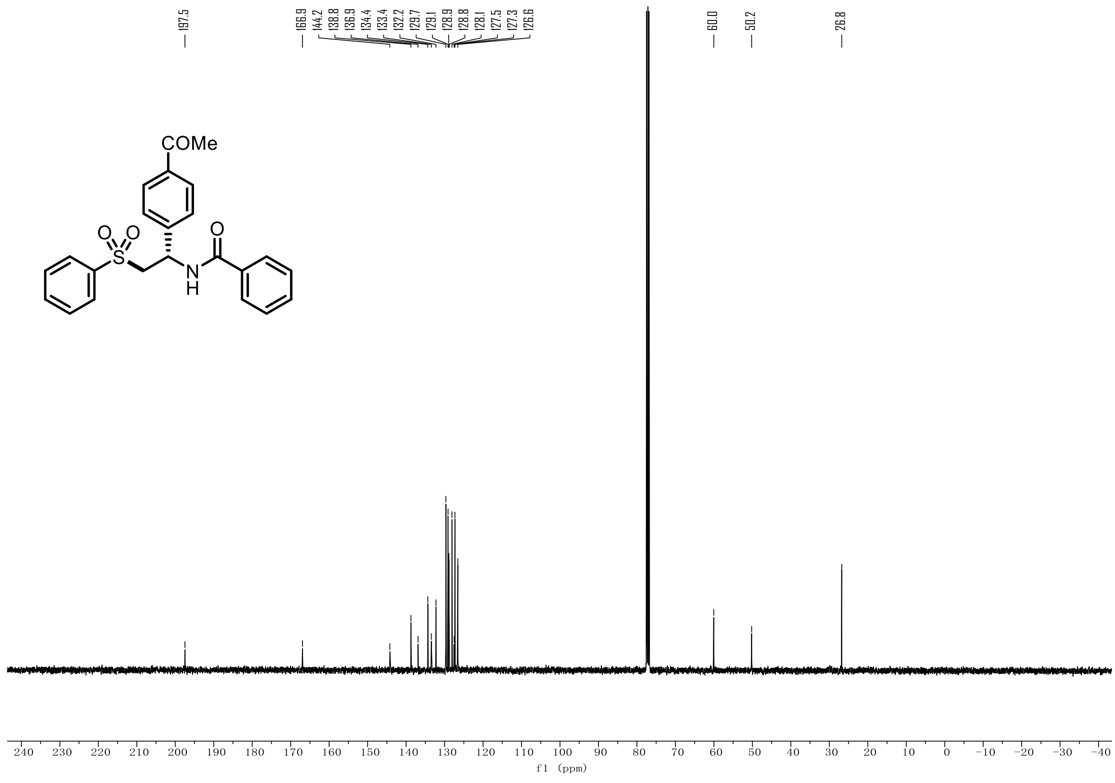

(*S*)-*N*-(1-(4-Formylphenyl)-2-(phenylsulfonyl)ethyl)benzamide (11).

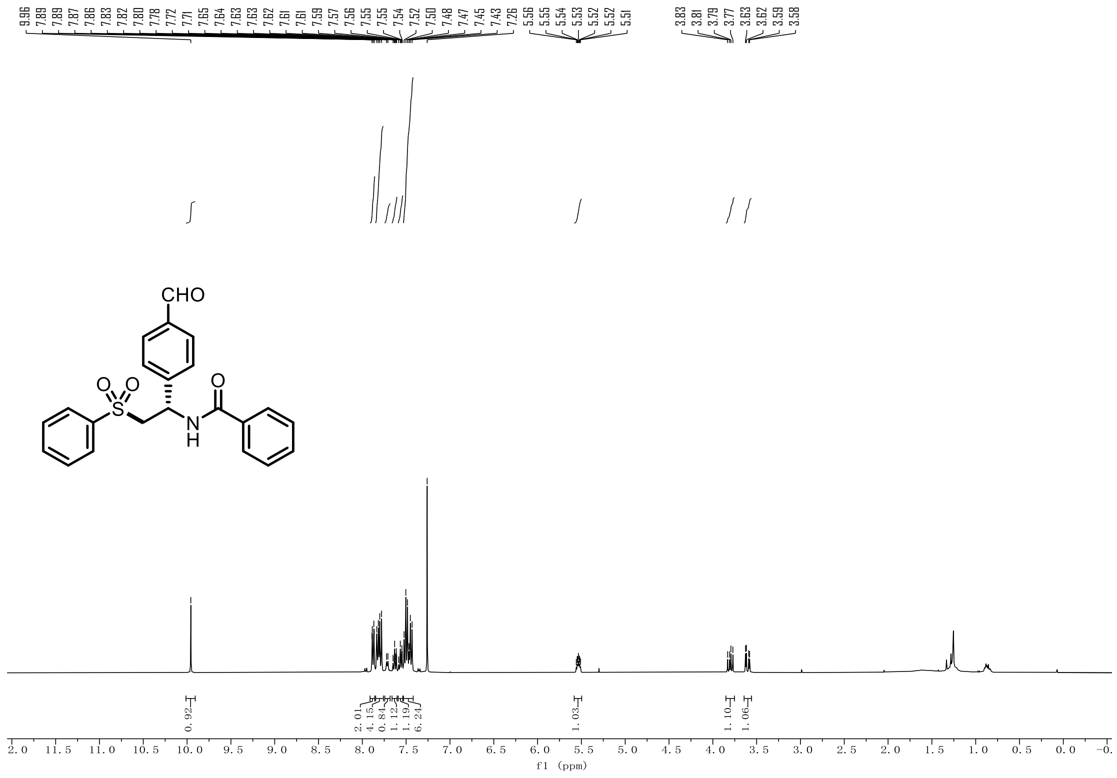

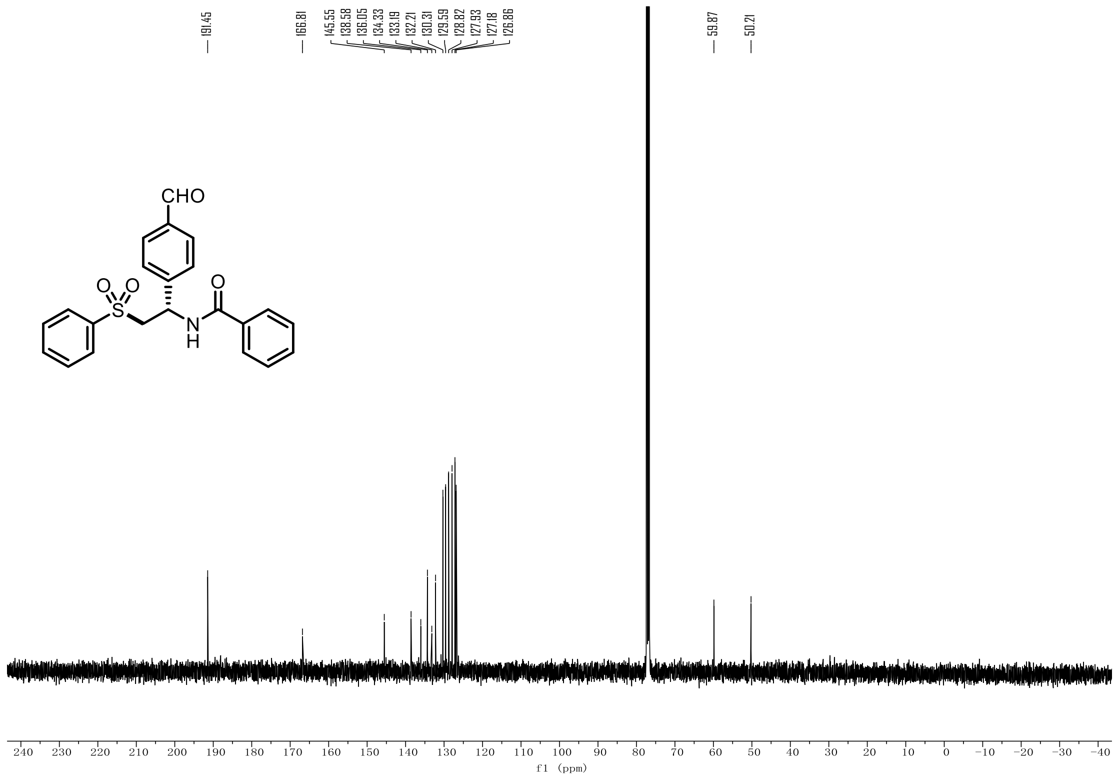

(*S*)-*N*-(2-(Phenylsulfonyl)-1-(4-(4,4,5,5-tetramethyl-1,3,2-dioxaborolan-2-yl)phenyl)ethyl)benzamide (12).

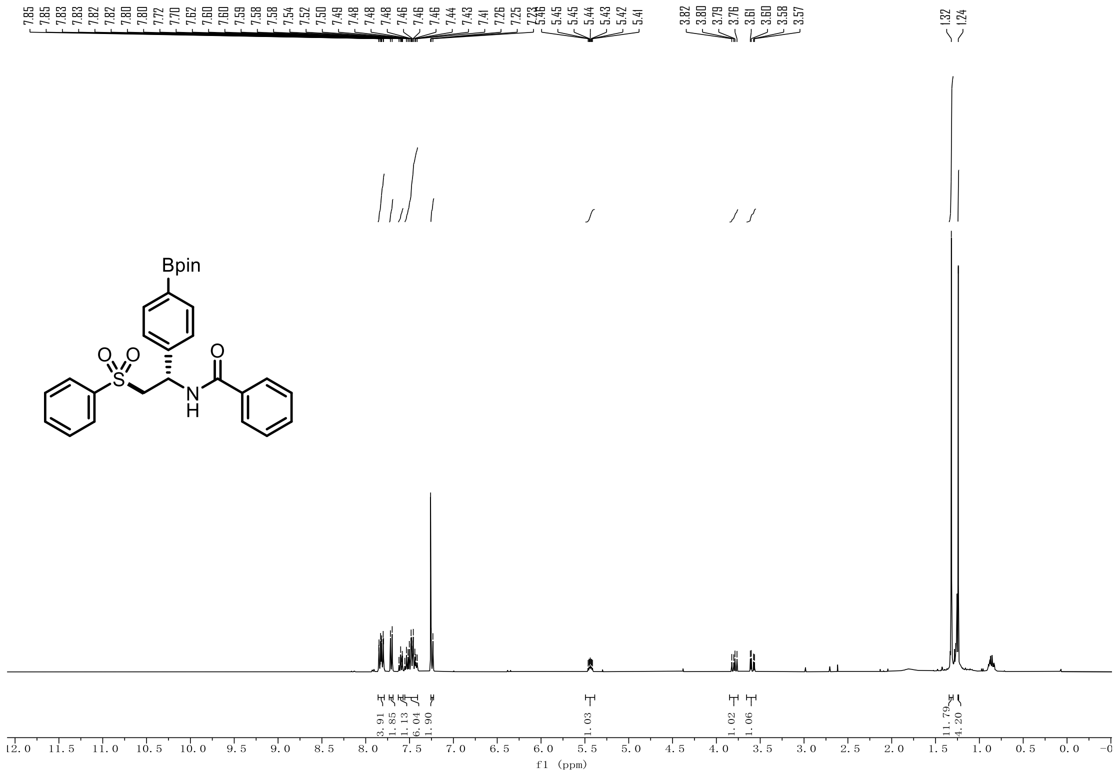

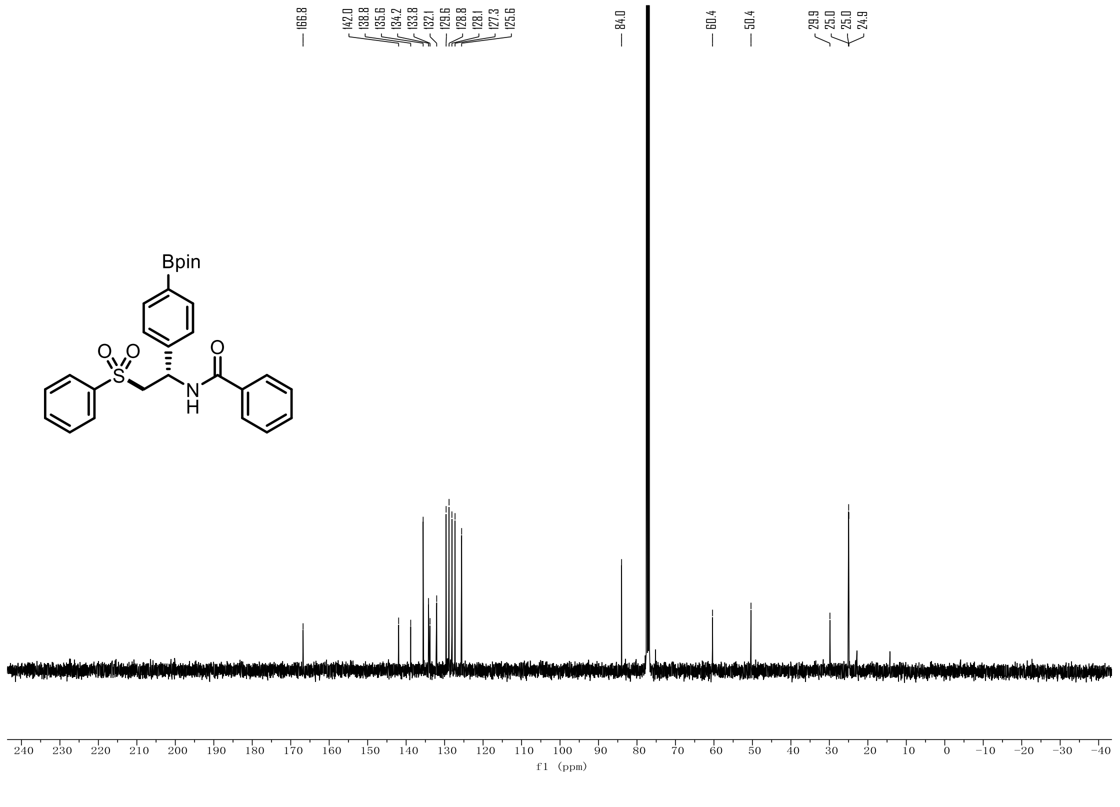

(*S*)-*N*-(1-(4-(1H-Pyrrol-1-yl)phenyl)-2-(phenylsulfonyl)ethyl)benzamide (13).

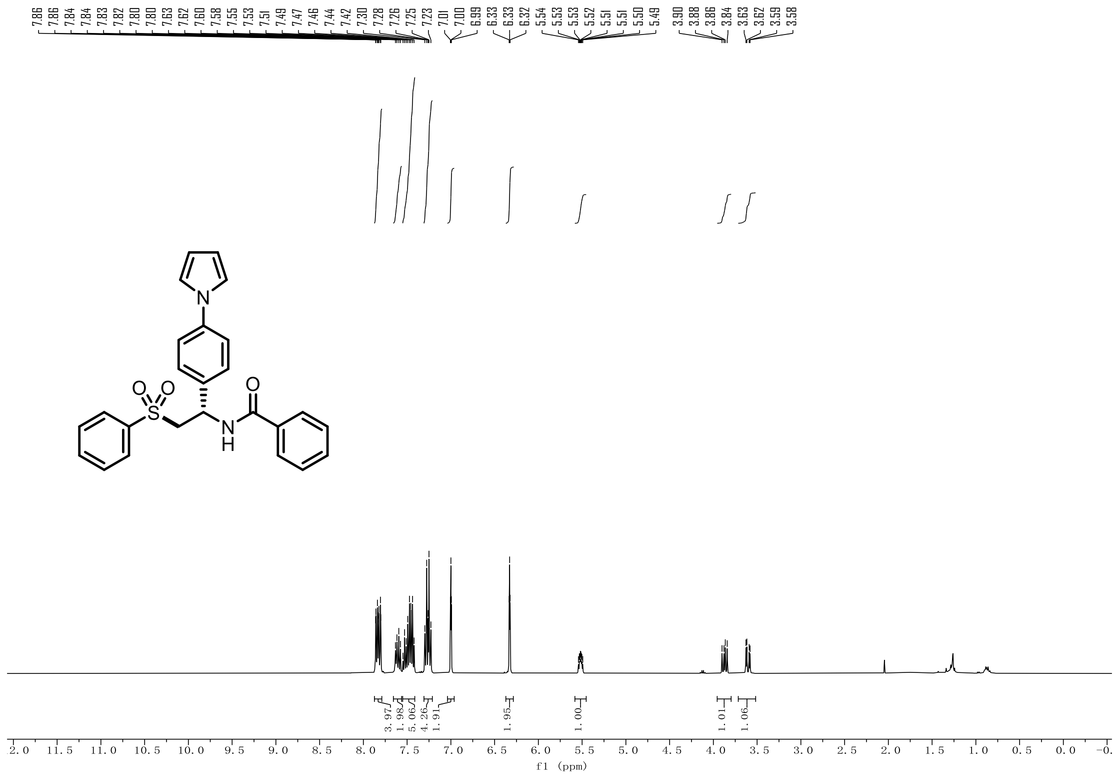

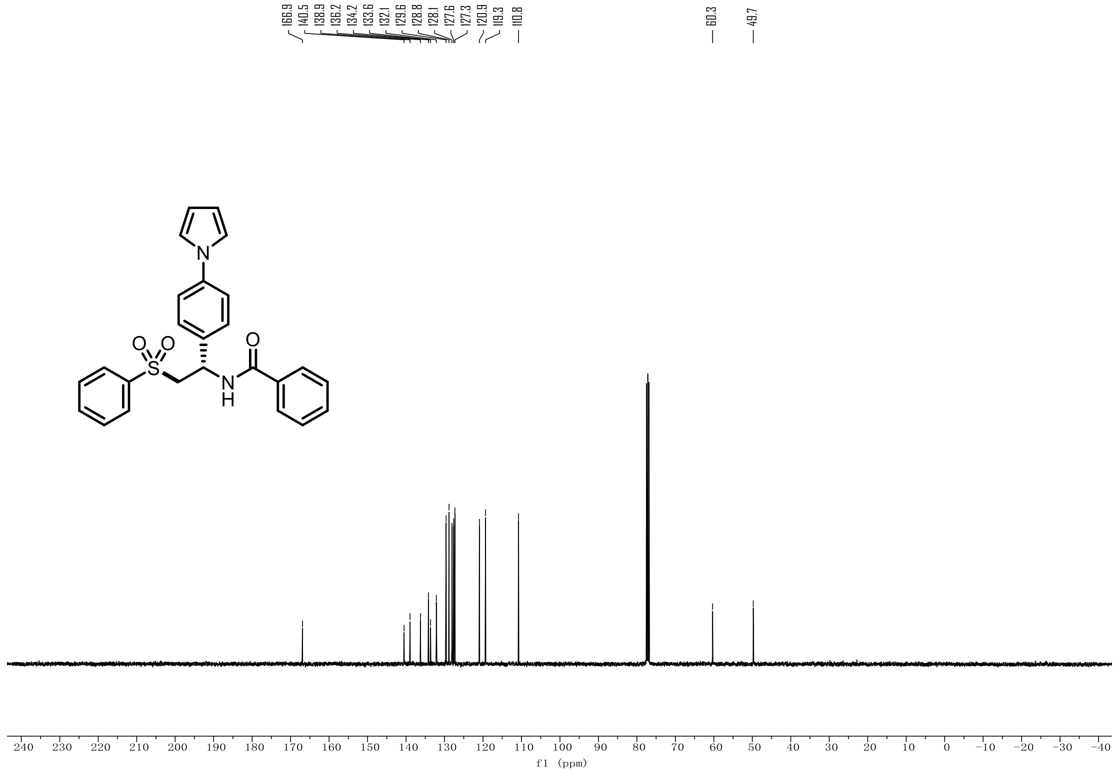

(*S*)-*N*-(1-(3-Methoxyphenyl)-2-(phenylsulfonyl)ethyl)benzamide (14).

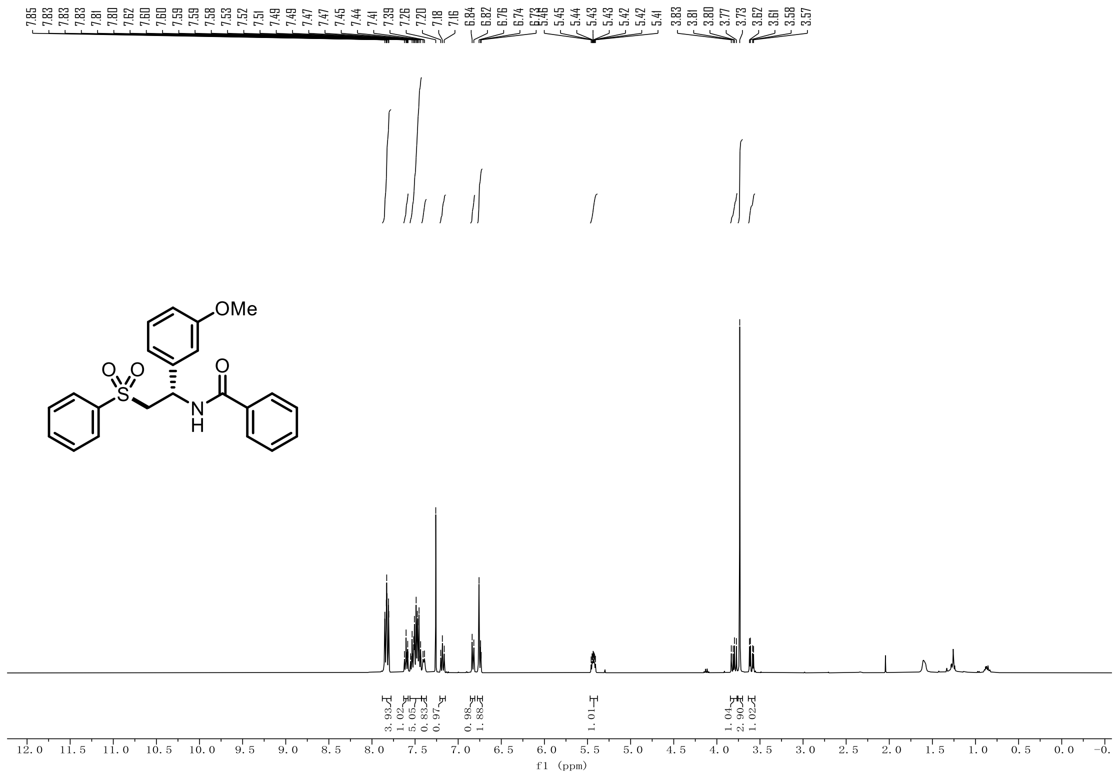

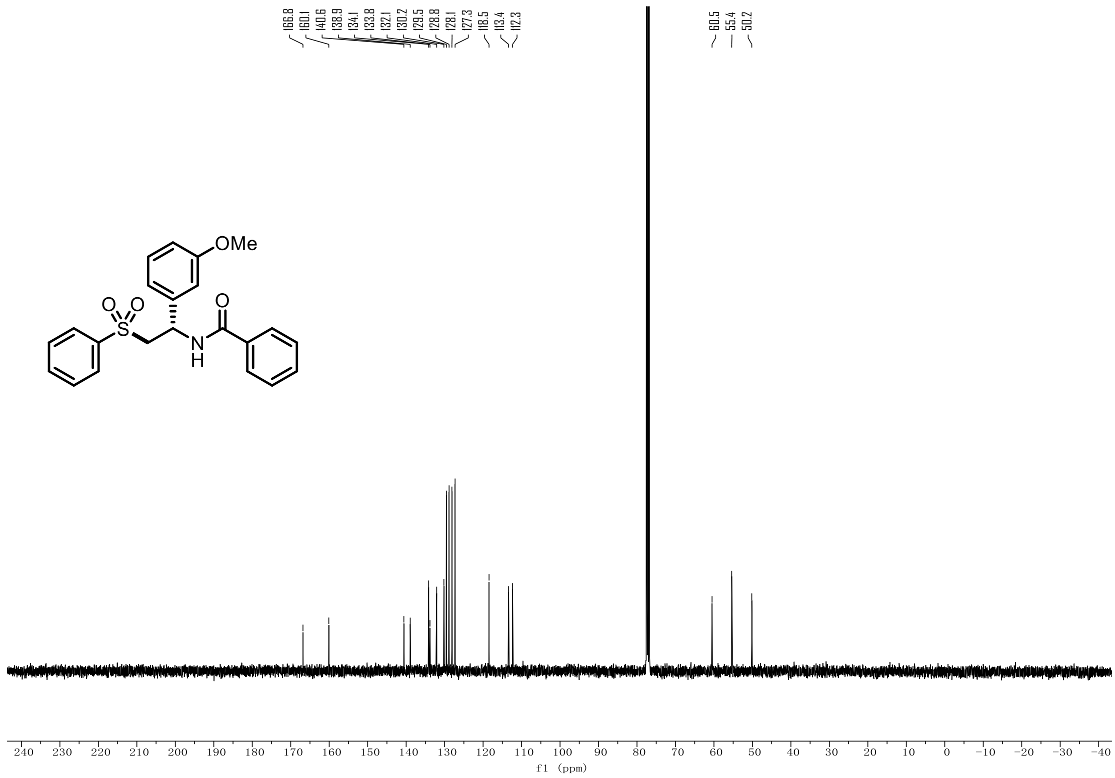

(*S*)-*N*-(2-(Phenylsulfonyl)-1-(*m*-tolyl)ethyl)benzamide (15).

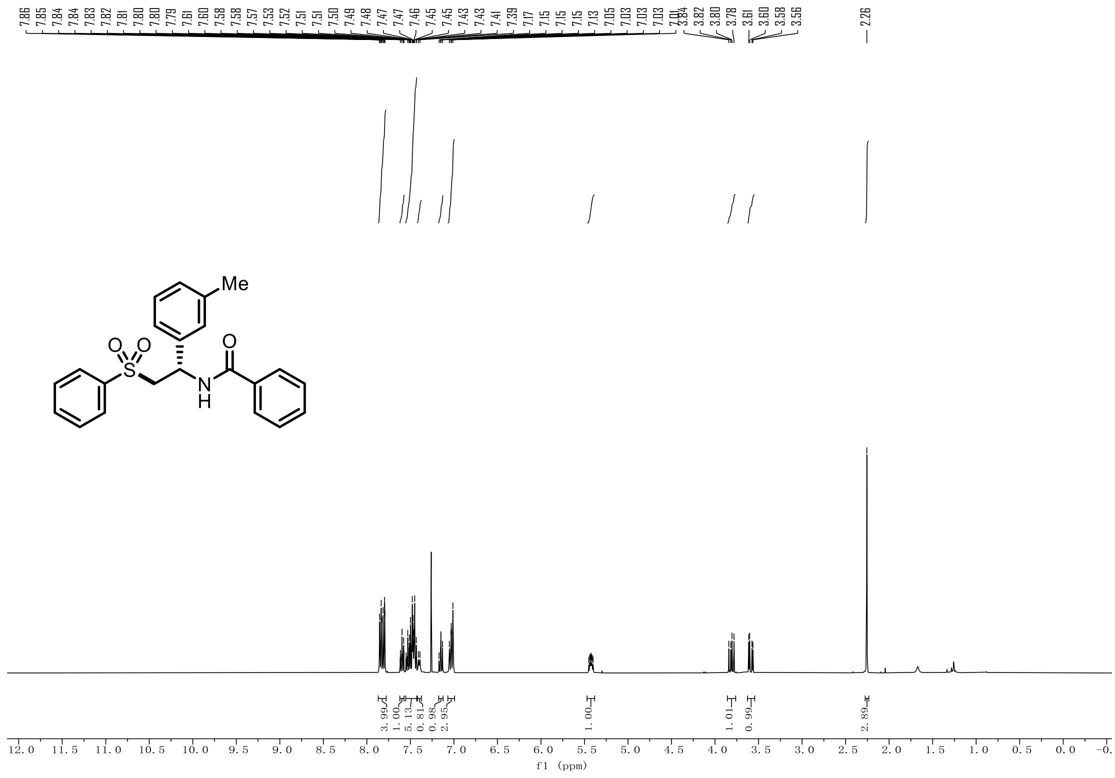

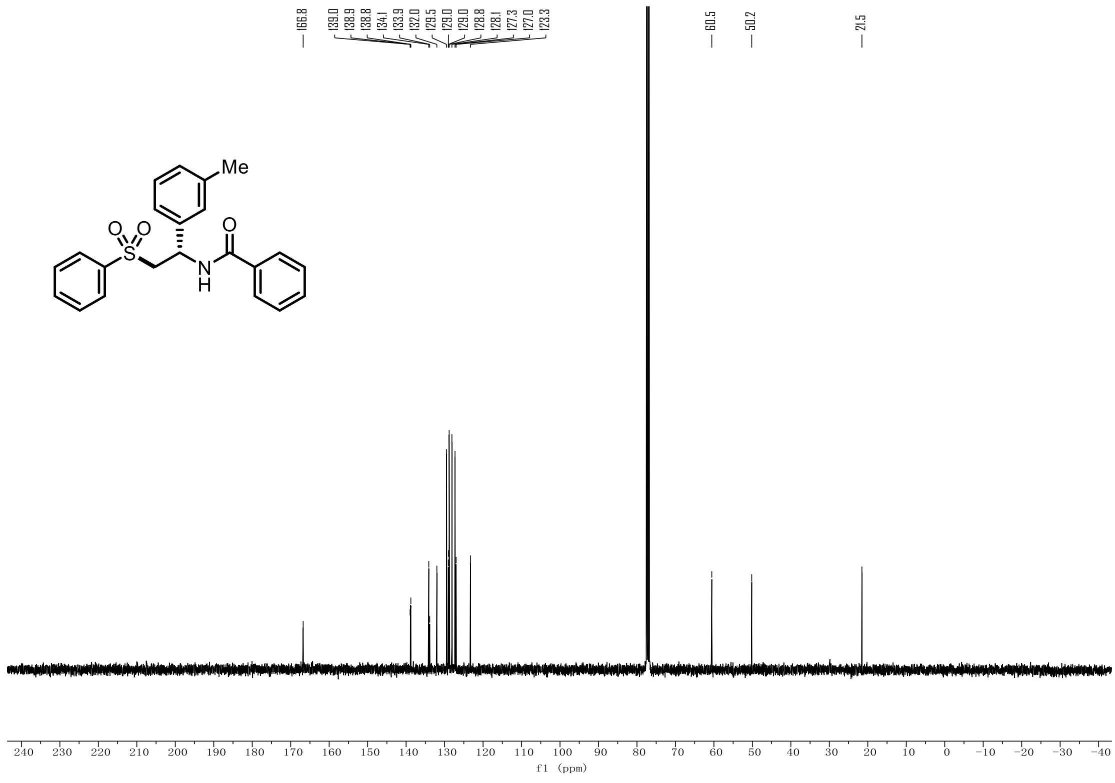

Dimethyl (*S*)-5-(1-benzamido-2-(phenylsulfonyl)ethyl)isophthalate (16).

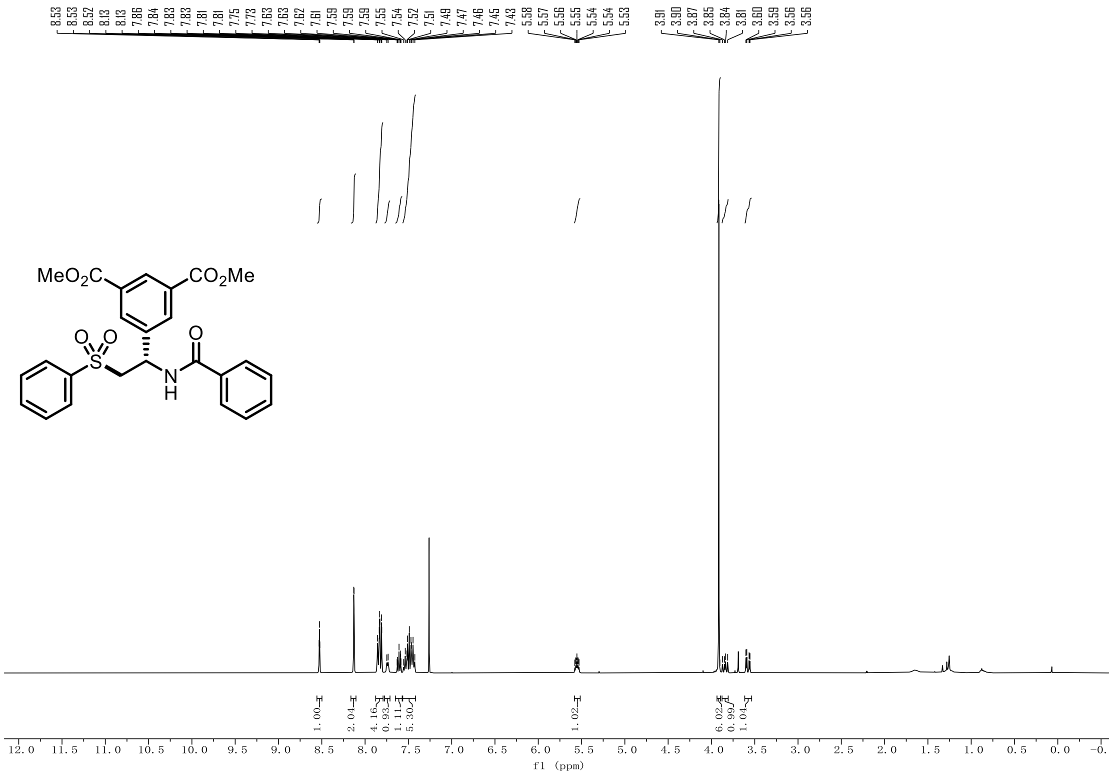

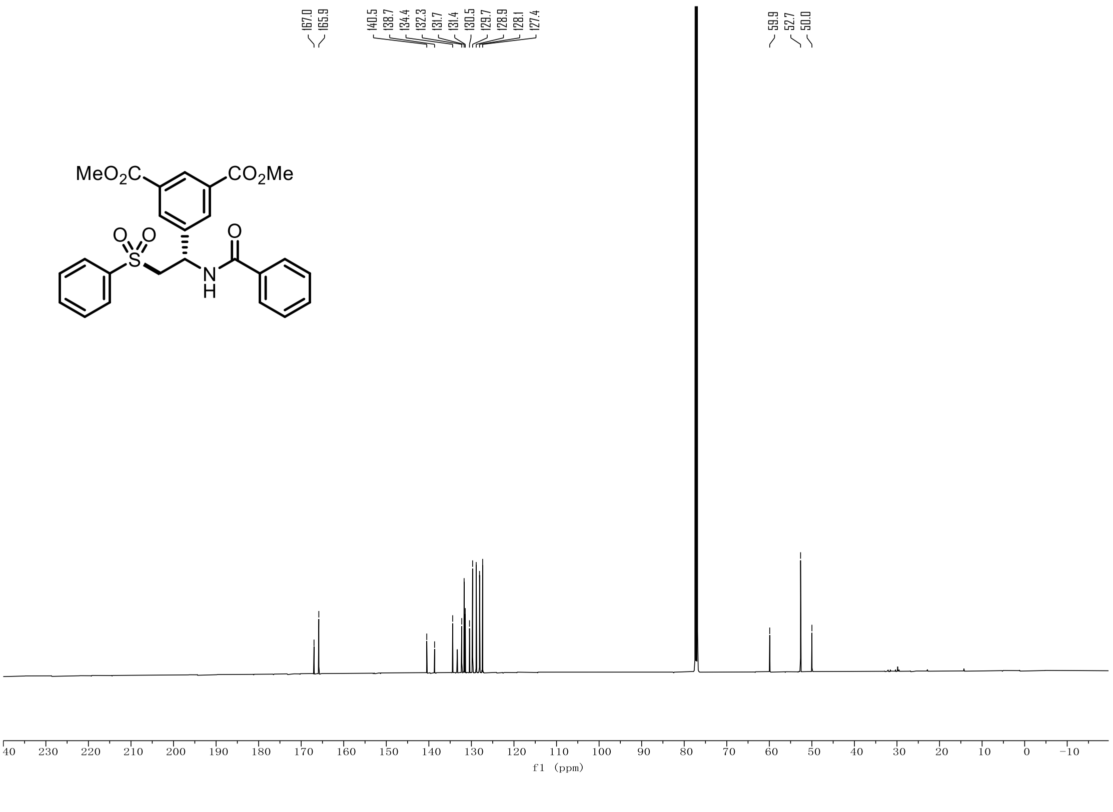

(*S*)-*N*-(1-(2-Methoxyphenyl)-2-(phenylsulfonyl)ethyl)benzamide (17).

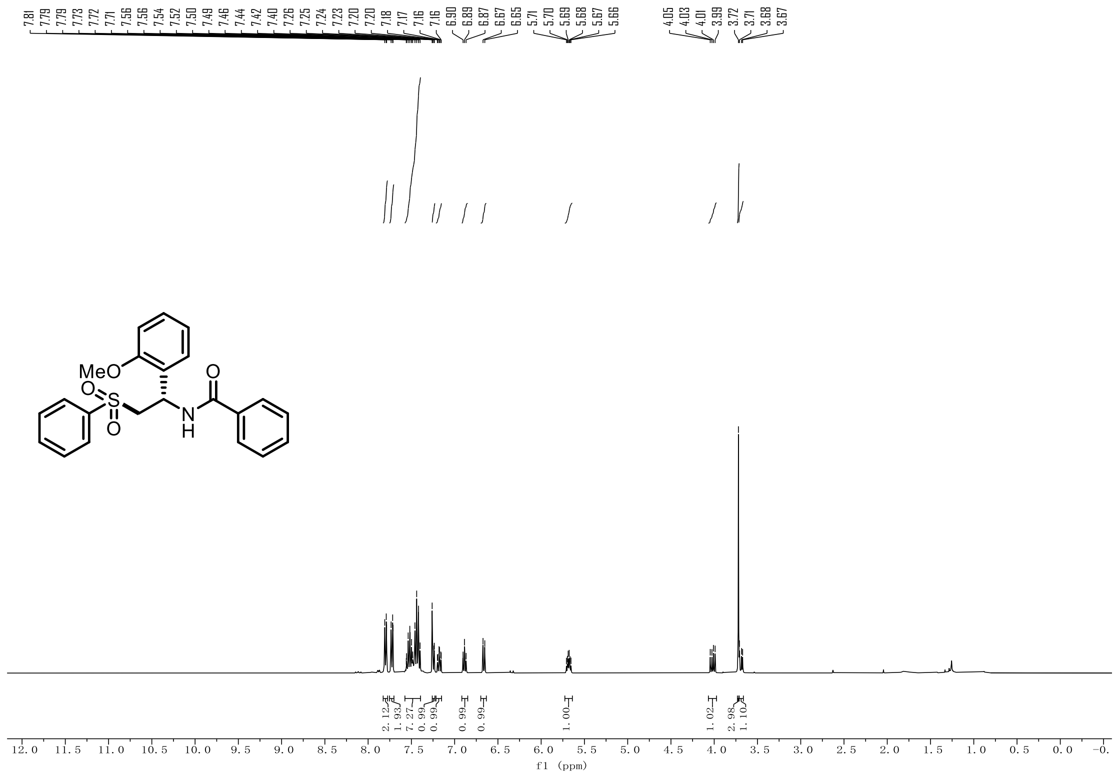

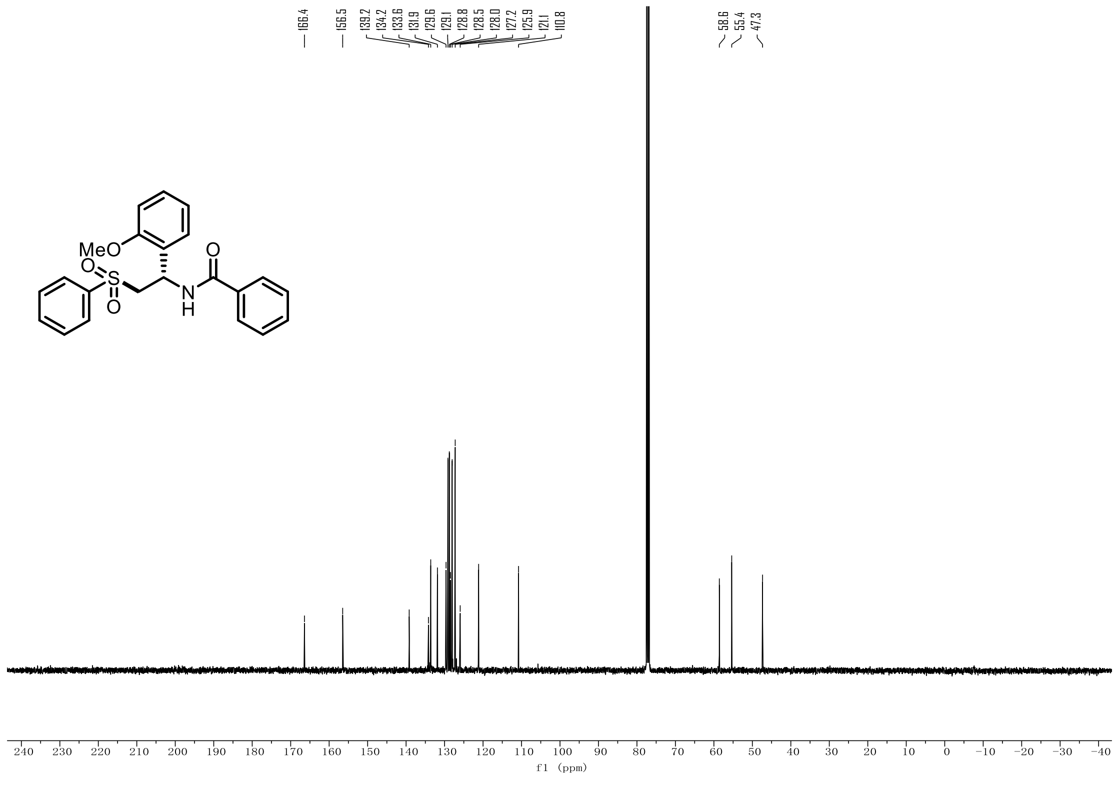

(*S*)-*N*-(1-(2-Fluorophenyl)-2-(phenylsulfonyl)ethyl)benzamide (18)

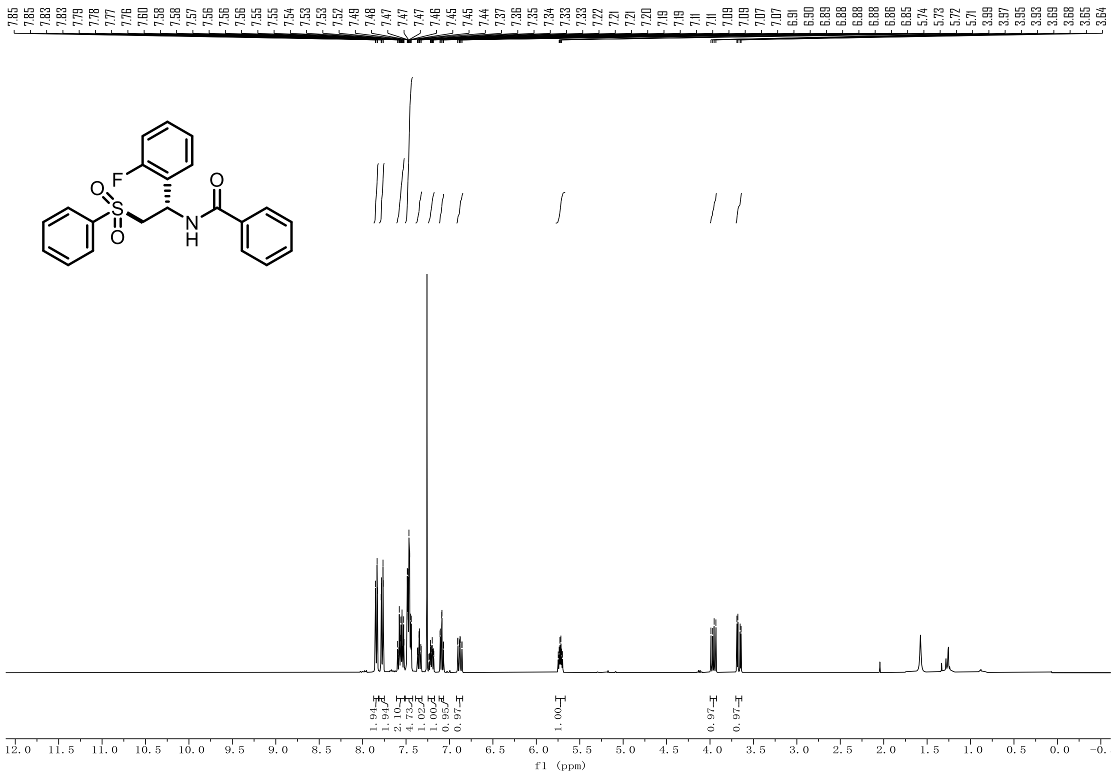

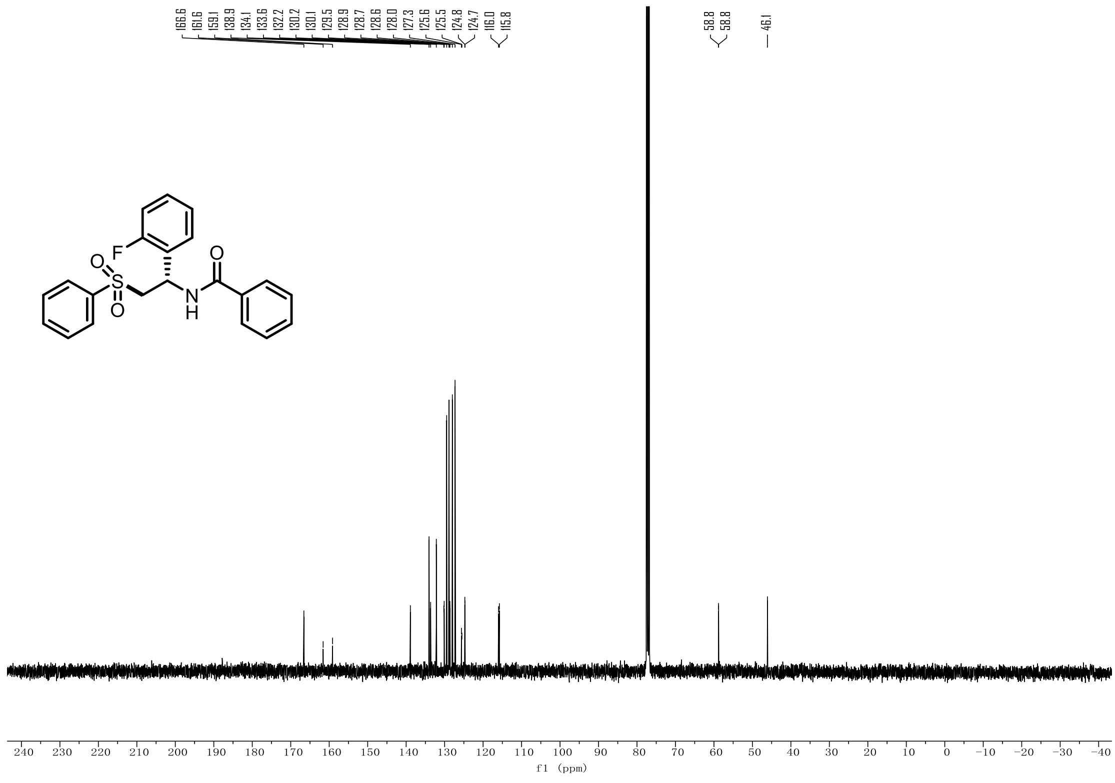

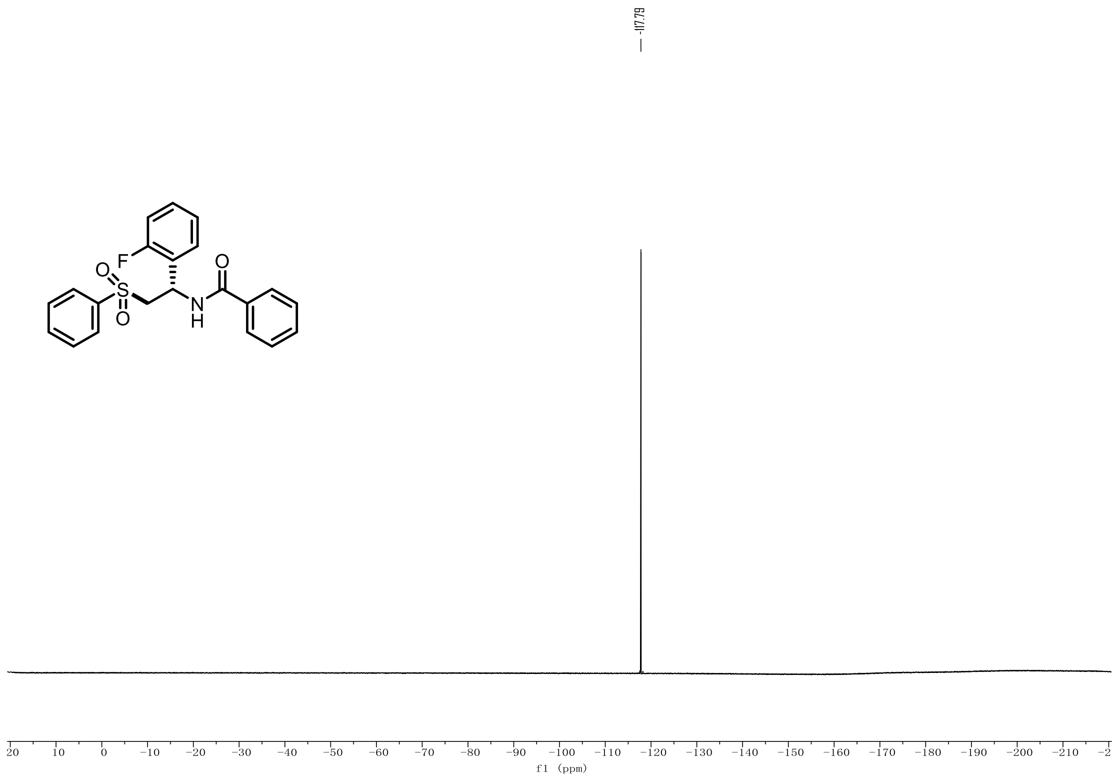

(*S*)-*N*-(1-Phenyl-2-(phenylsulfonyl)ethyl)benzamide (19).

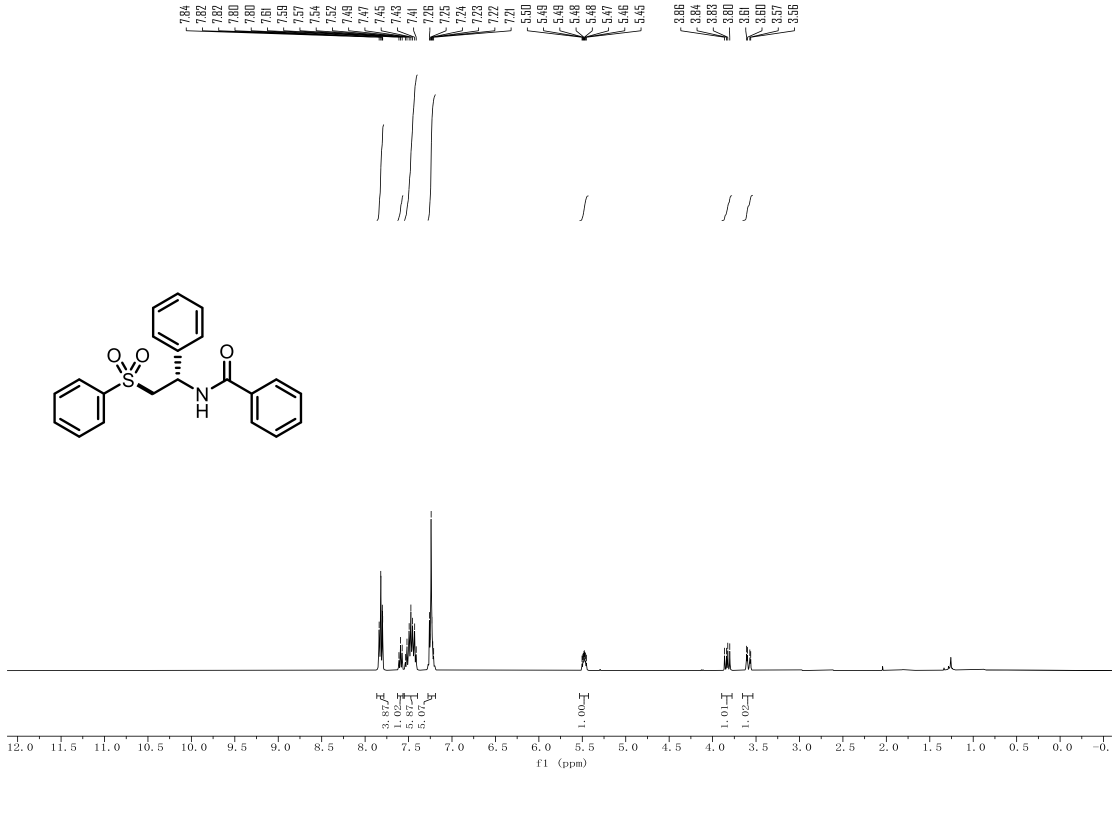

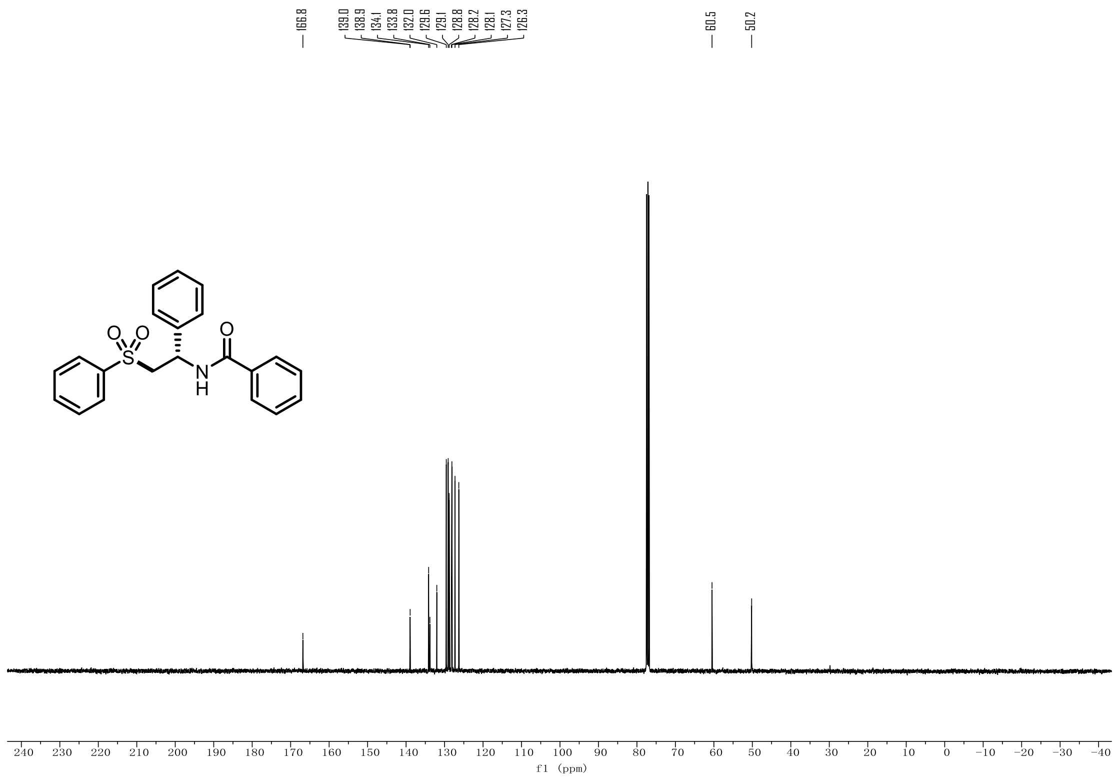

(*S*)-*N*-(1-(naphthalen-2-yl)-2-(phenylsulfonyl)ethyl)benzamide (20).

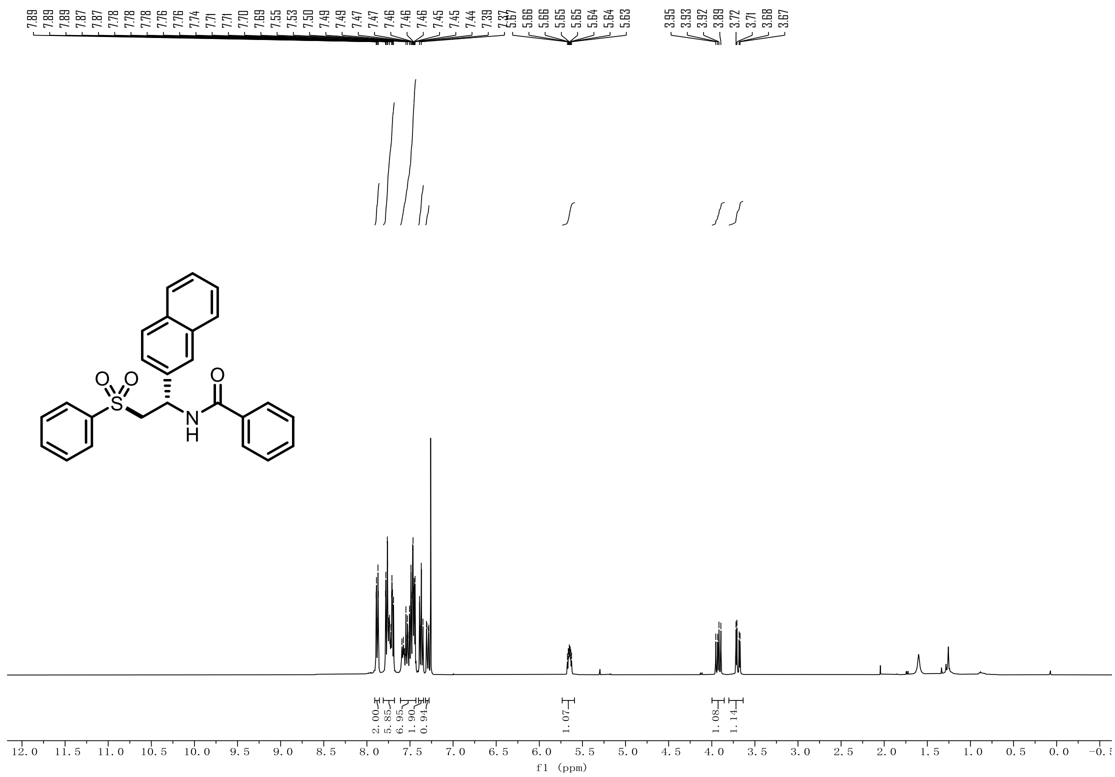

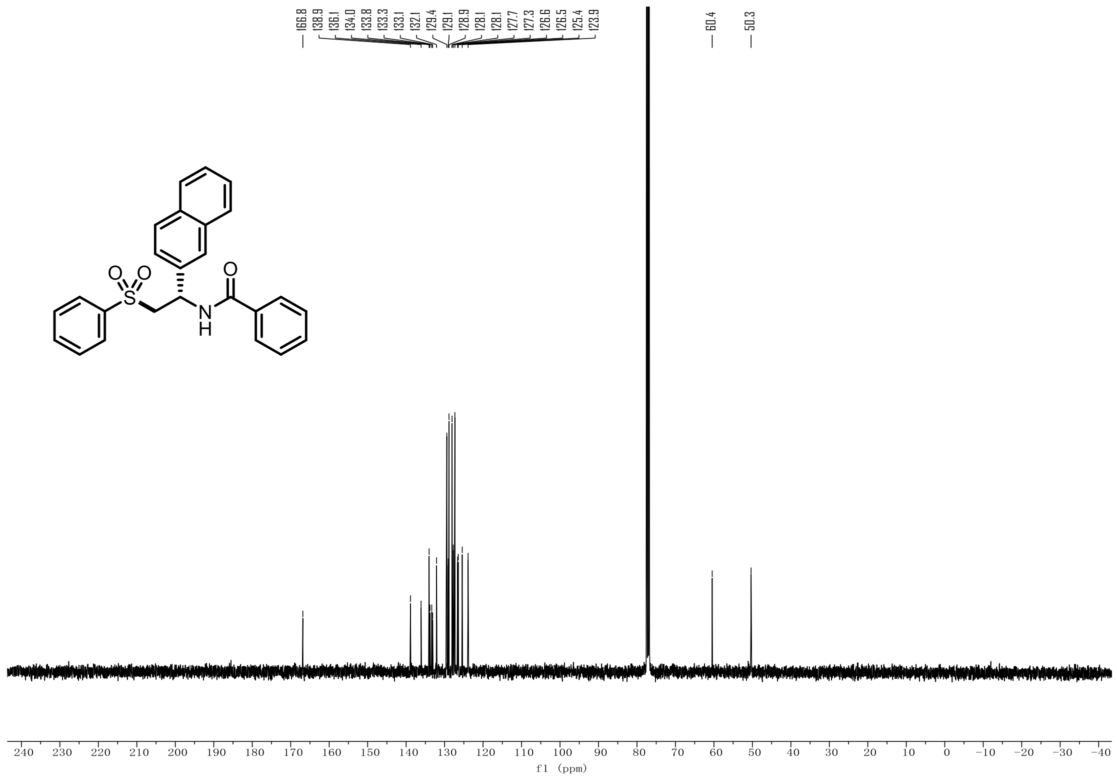

(*S*)-*N*-(1-(4-(Tert-butyl)phenyl)-2-(phenylsulfonyl)ethyl)benzamide (21).

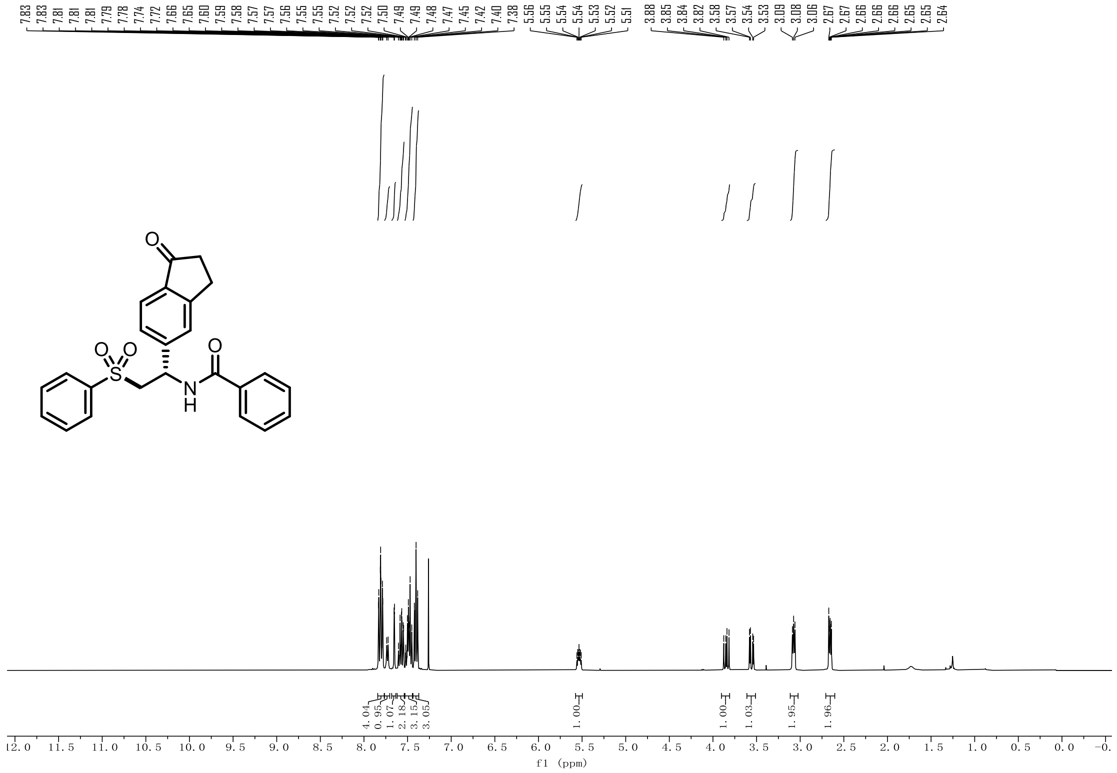

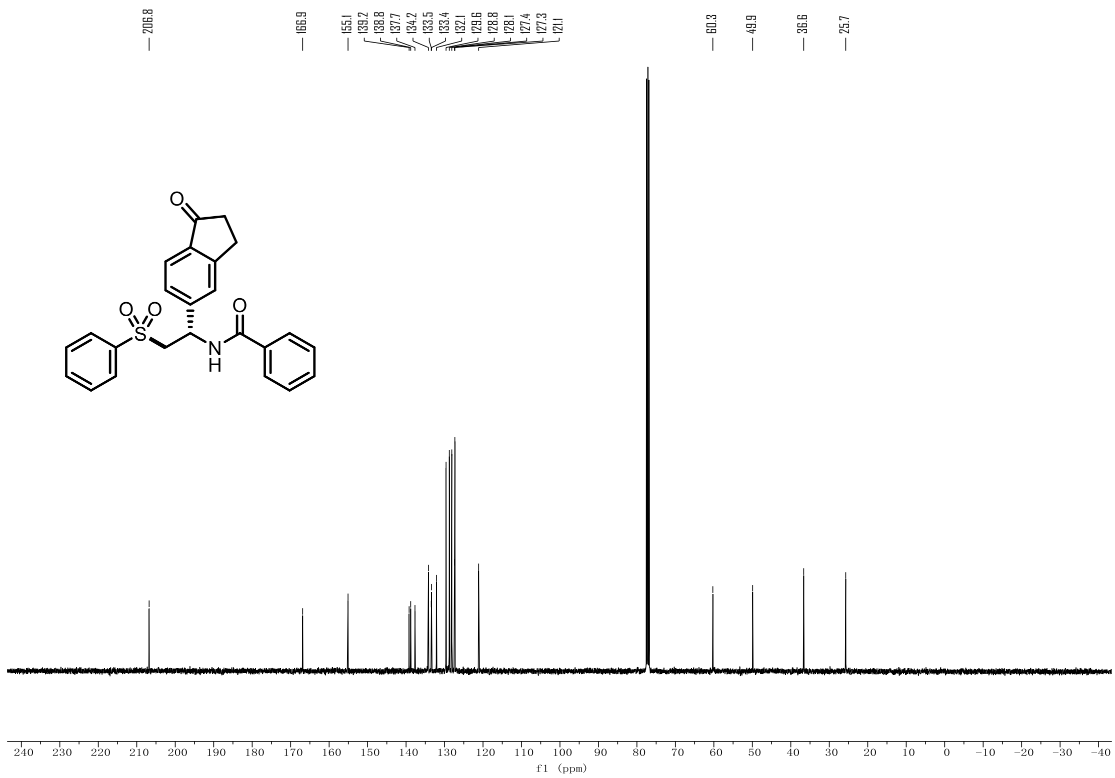

(*S*)-*N*-(1-(2,3-Dihydrobenzo[b][1,4]dioxin-6-yl)-2-(phenylsulfonyl)ethyl)benzamide (22).

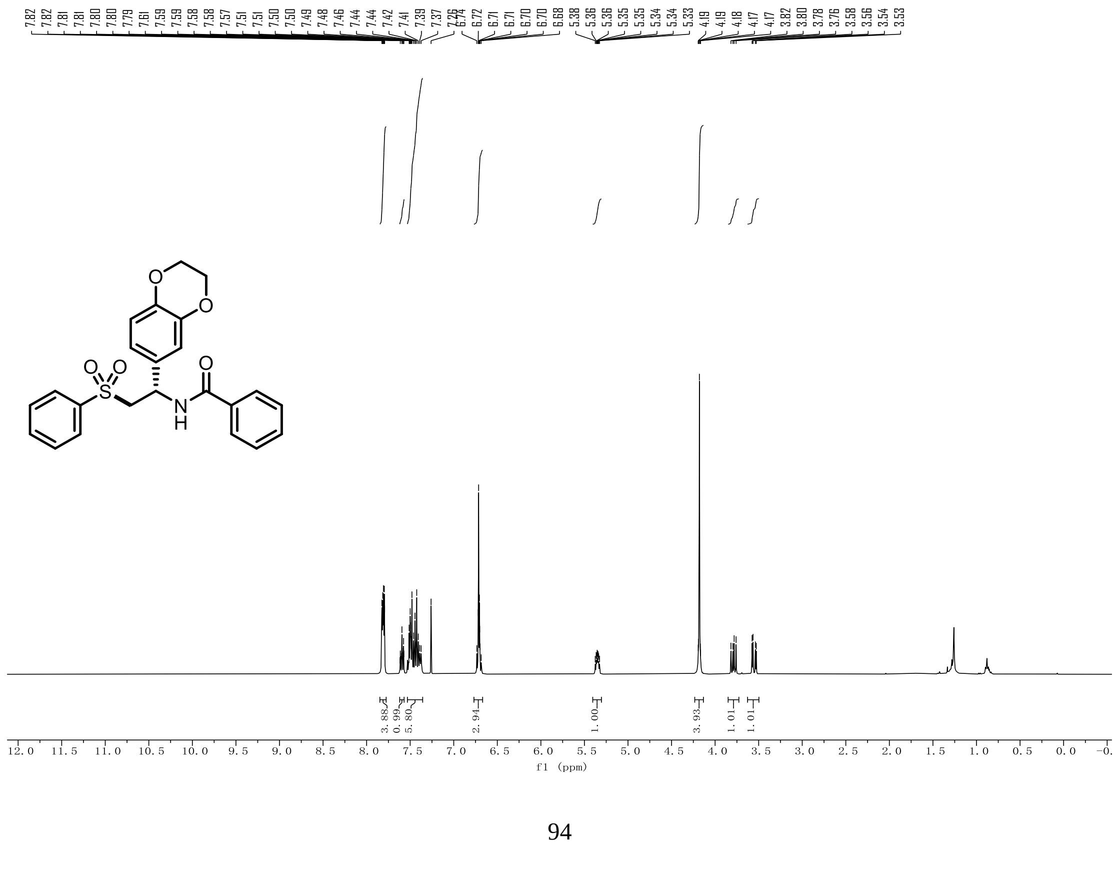

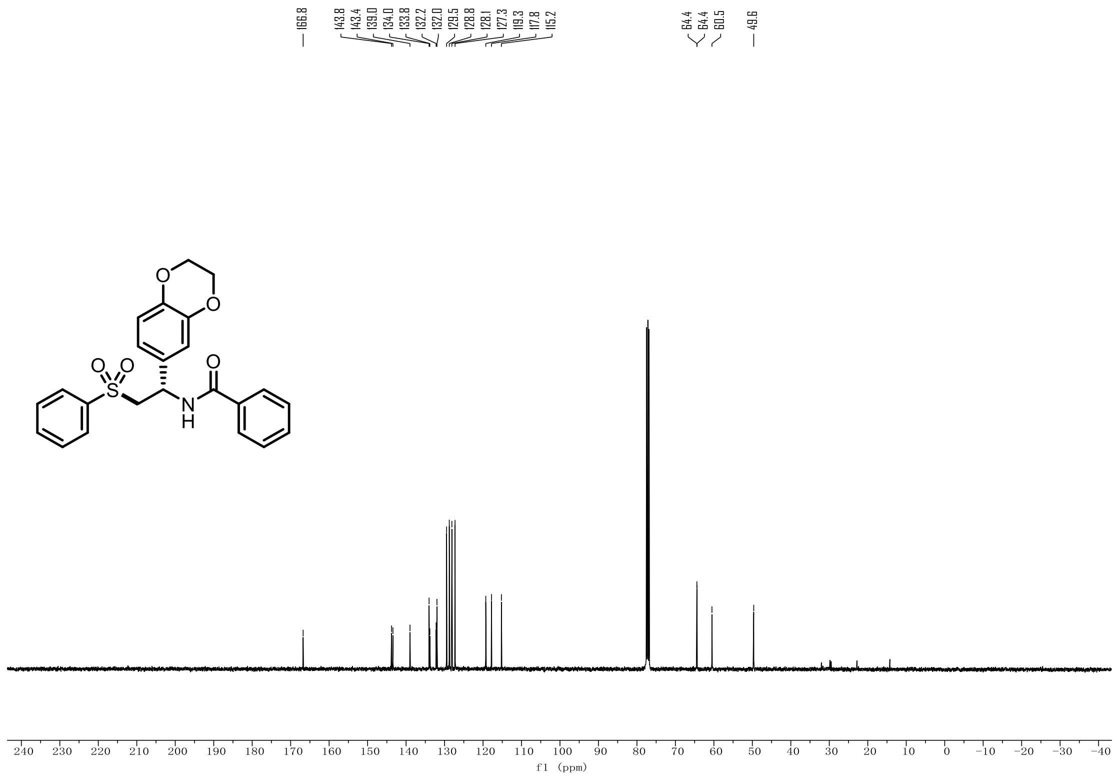

(*S*)-*N*-(2-(Phenylsulfonyl)-1-(quinolin-7-yl)ethyl)benzamide (23).

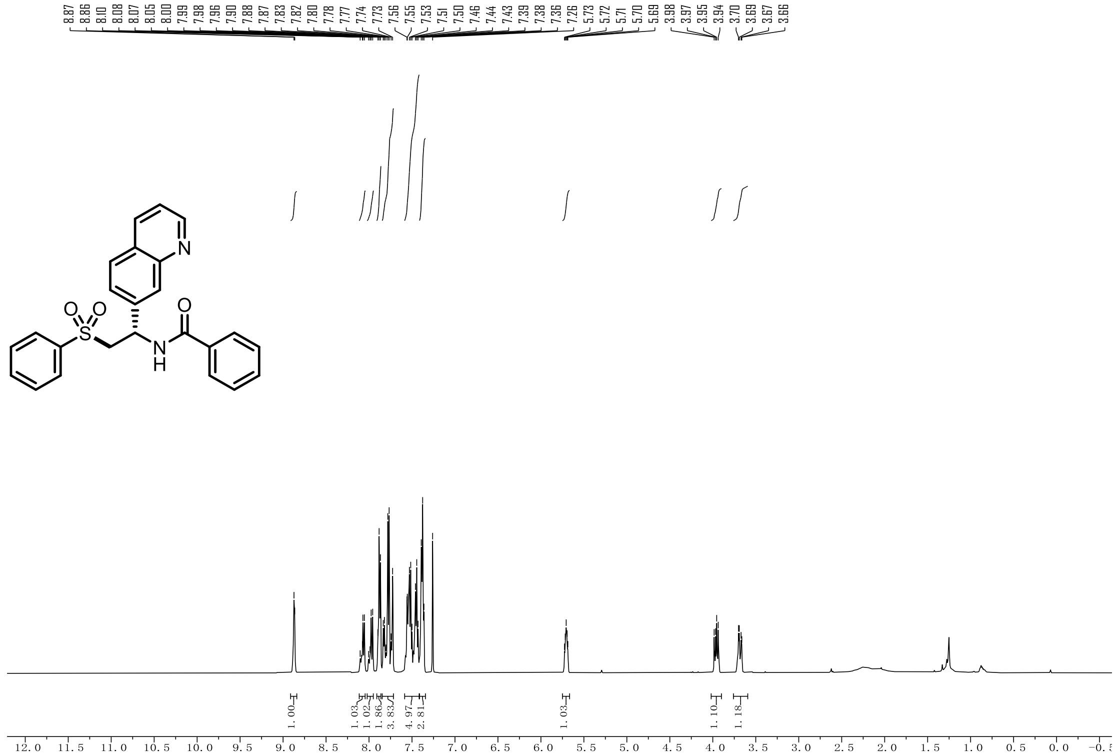

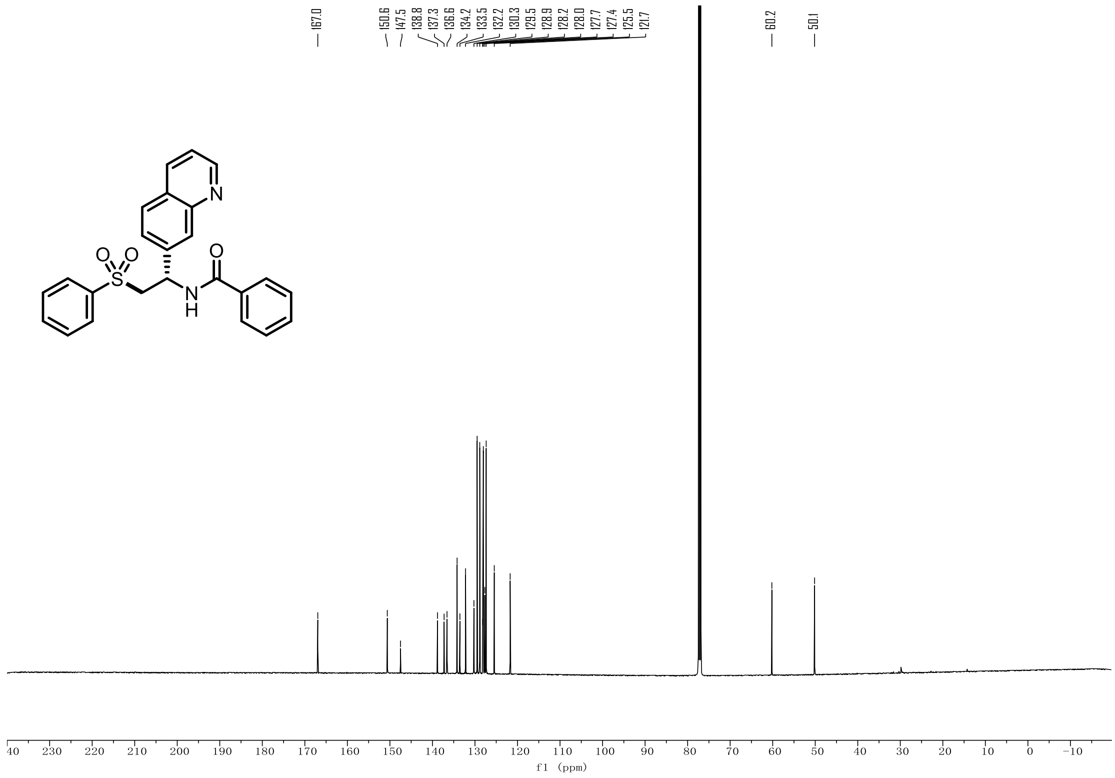

(*S*)-*N*-(2-(Phenylsulfonyl)-1-(thiophen-3-yl)ethyl)benzamide (24).

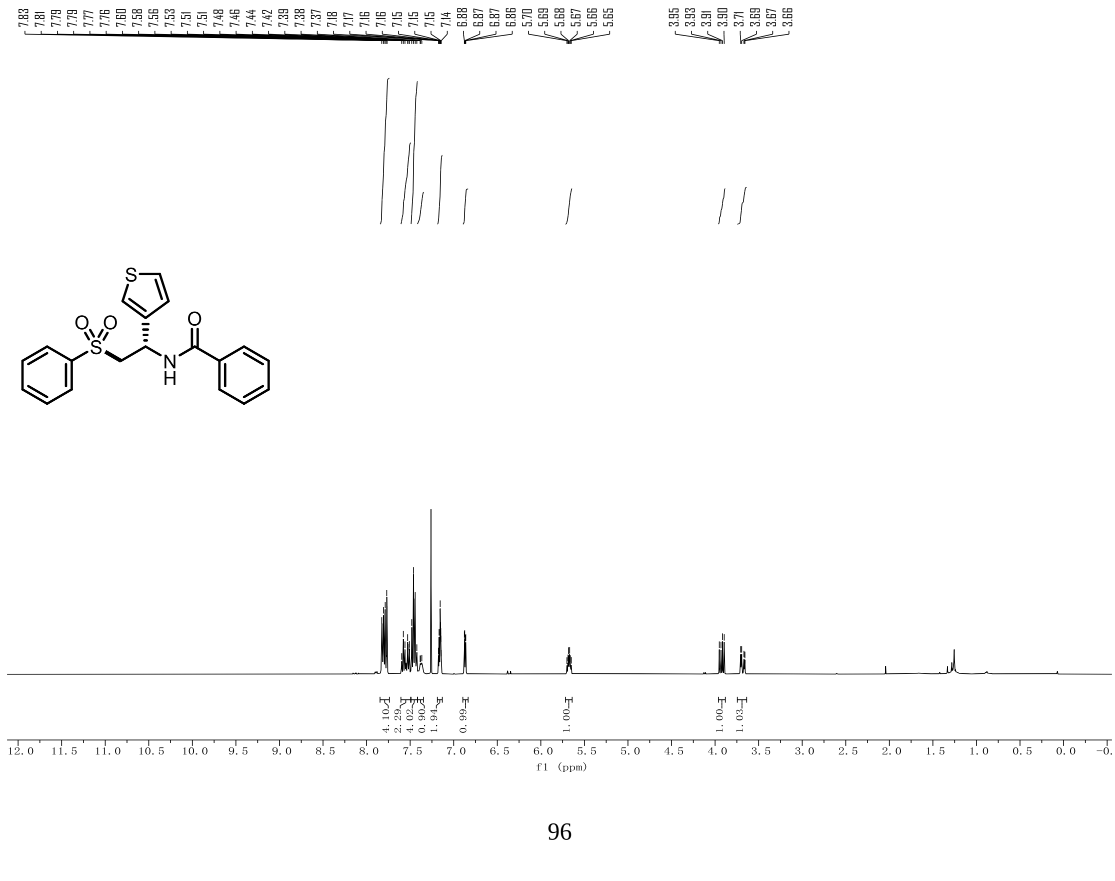

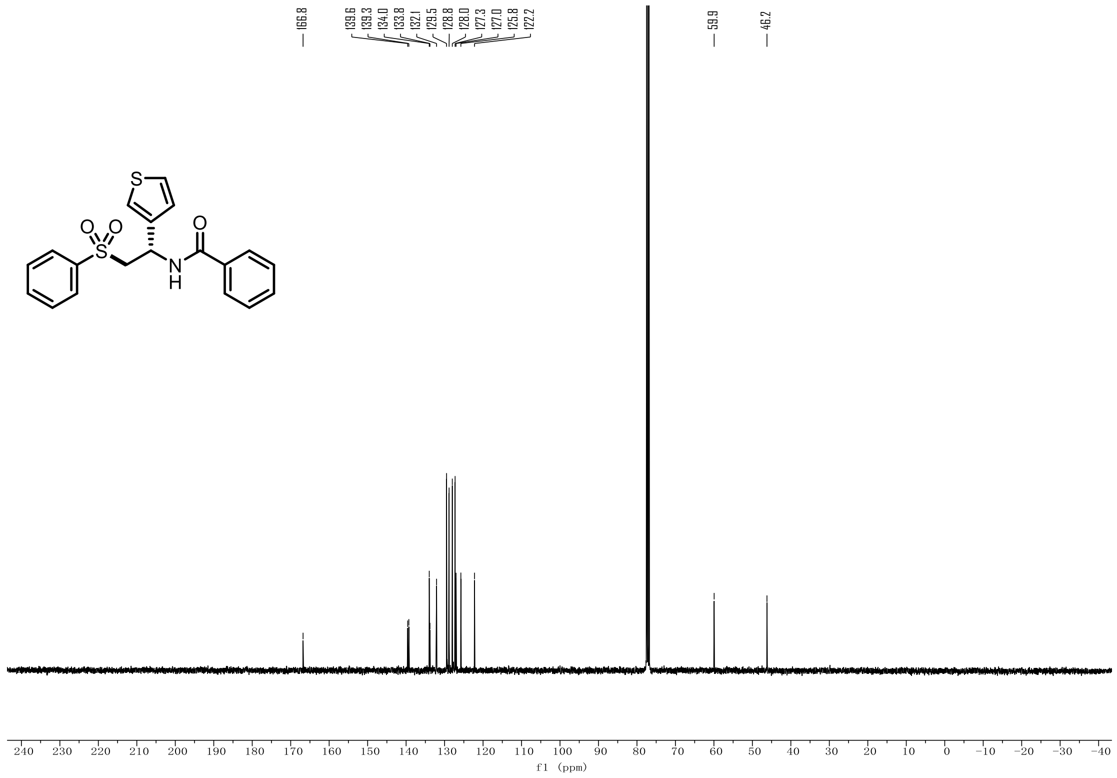

(*S*)-*N*-(2-(Phenylsulfonyl)-1-(pyrimidin-5-yl)ethyl)benzamide (25)

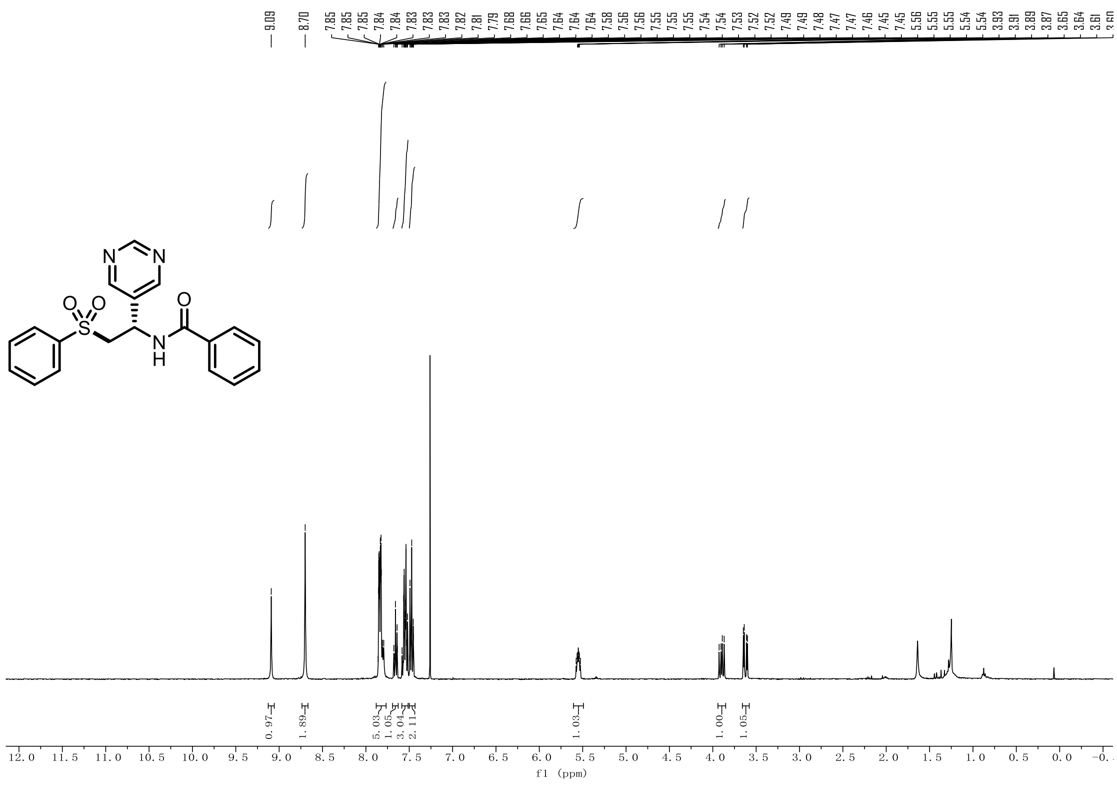

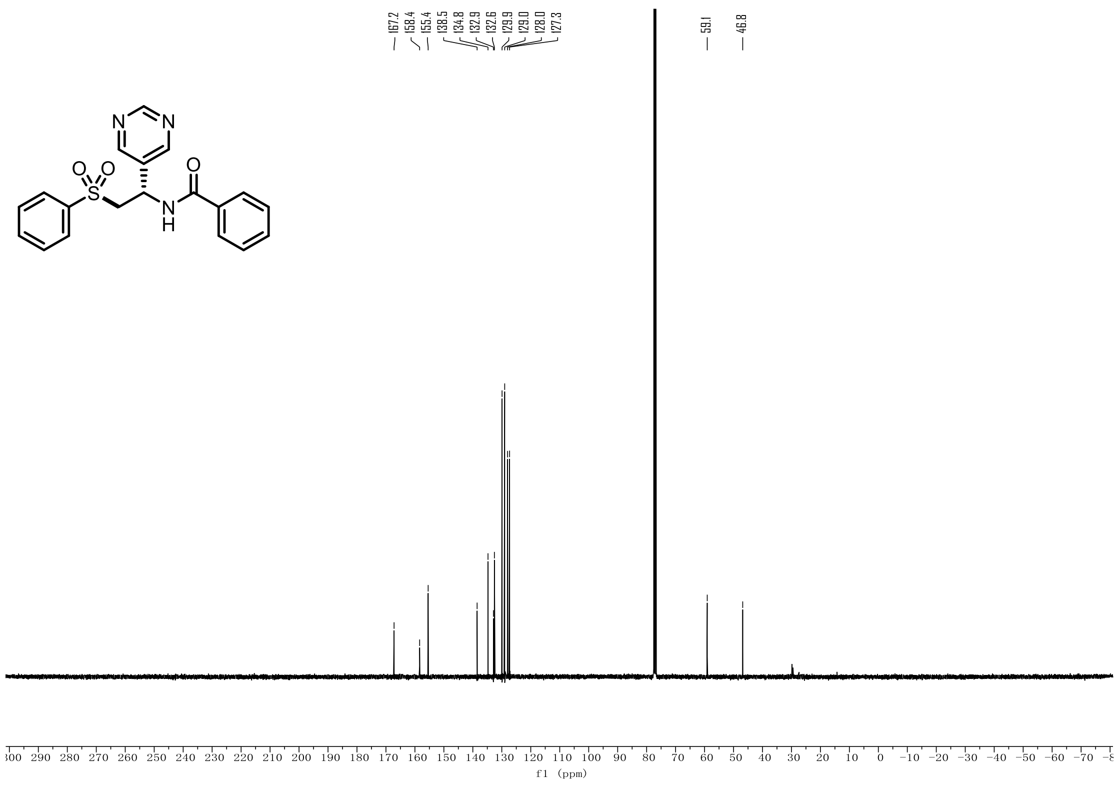

(*S*)-*N*-(1-(Dibenzo[b,d]thiophen-2-yl)-2-(phenylsulfonyl)ethyl)benzamide (26)

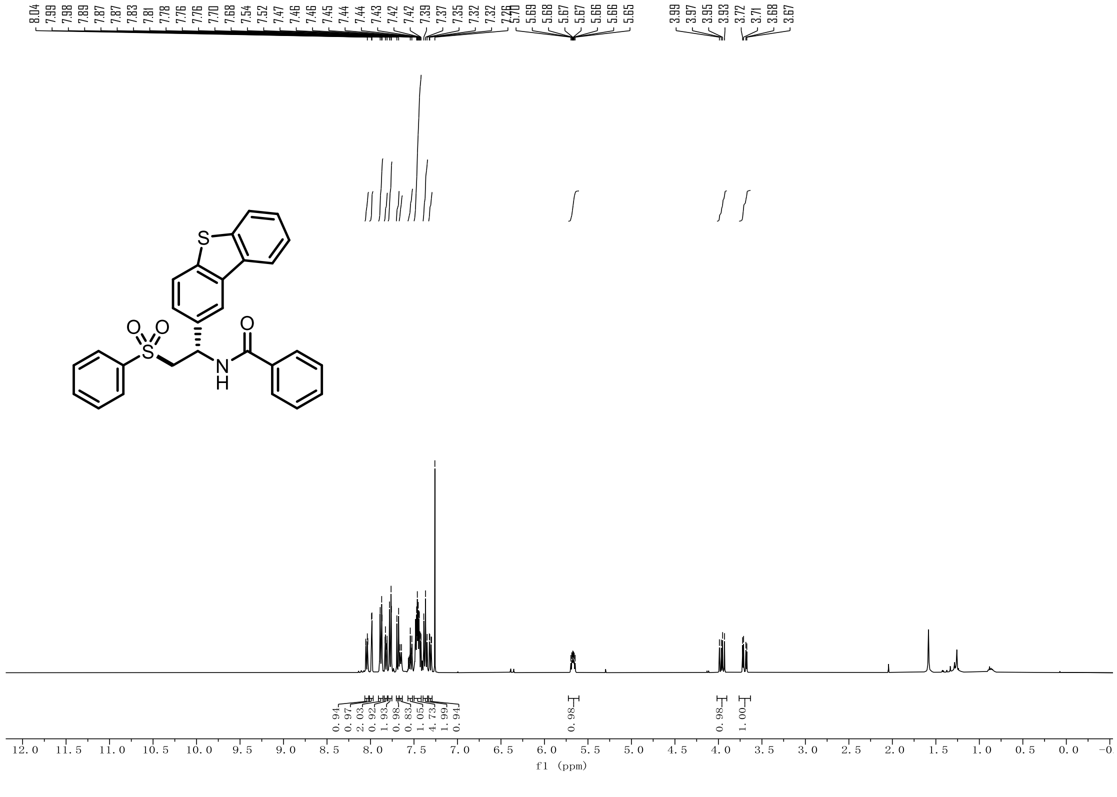

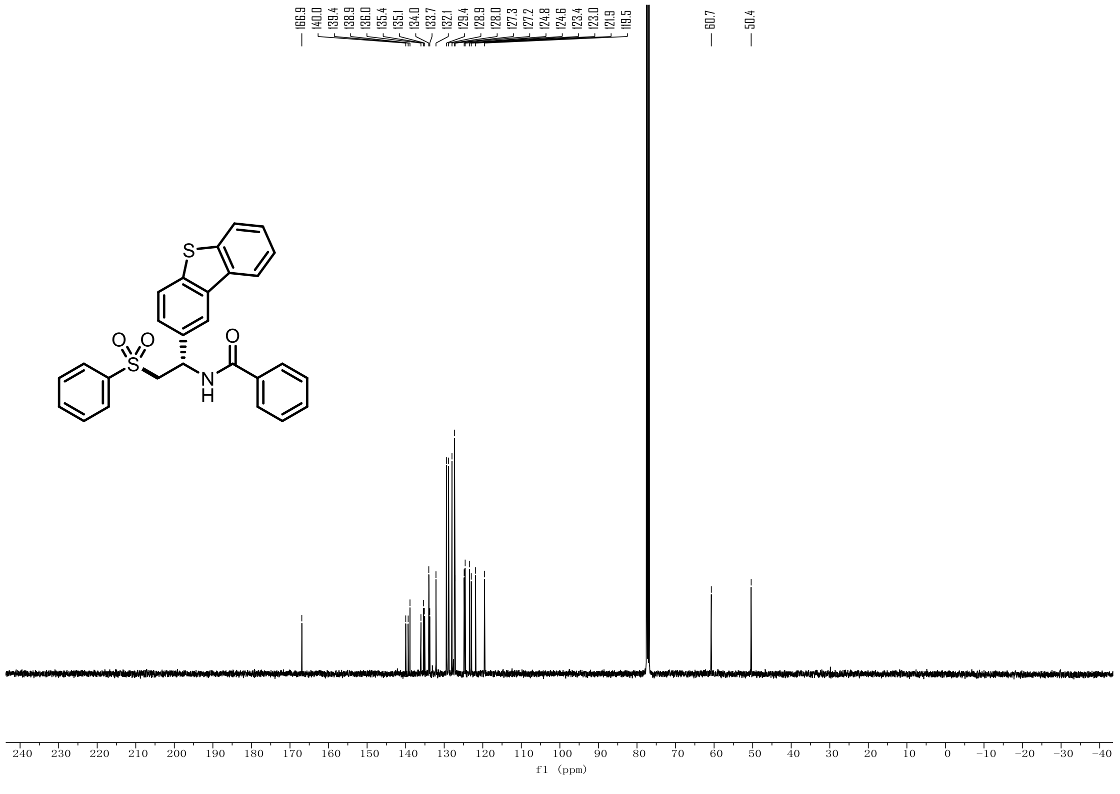

(*S,E*)-*N*-(3-Methyl-1-(phenylsulfonyl)pent-3-en-2-yl)benzamide (27).

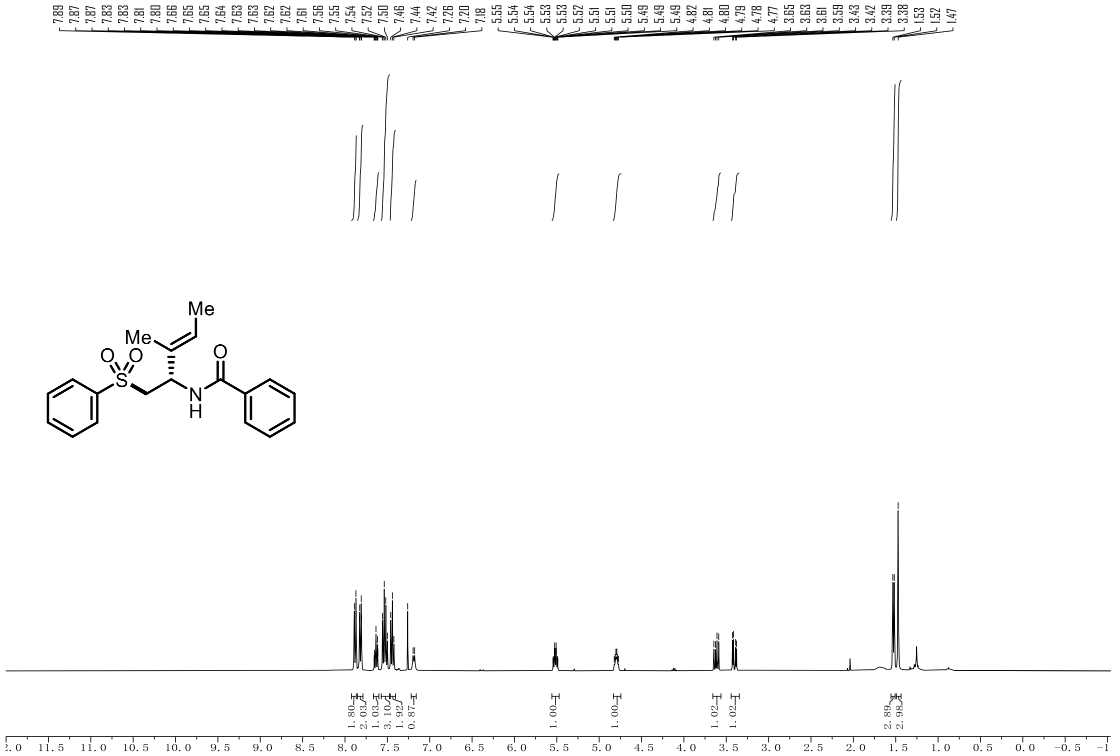

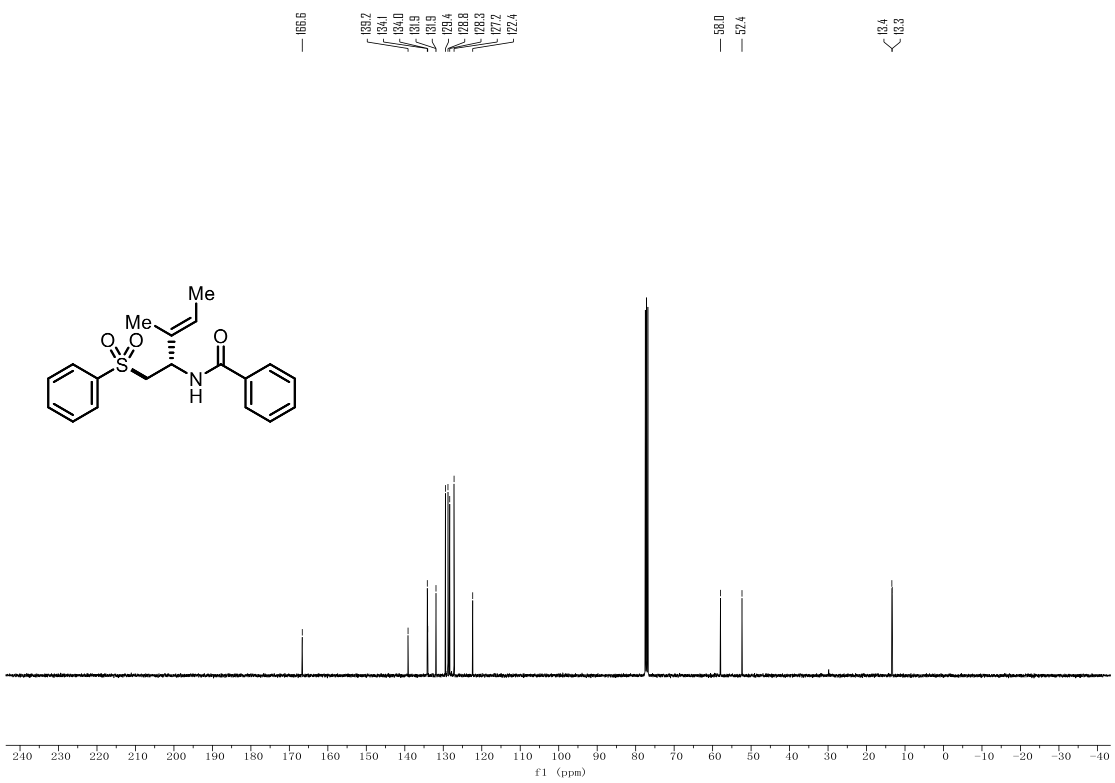

(*S*)-*N*-(4-Methyl-1-(phenylsulfonyl)pent-3-en-2-yl)benzamide (28)

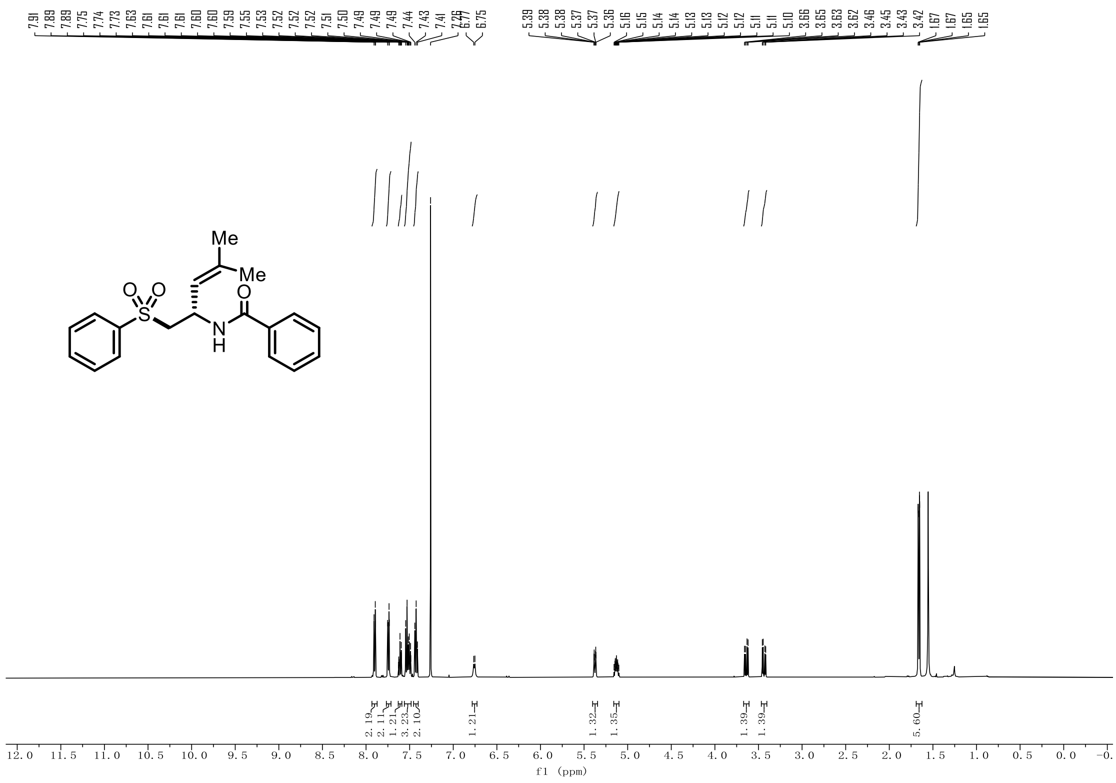

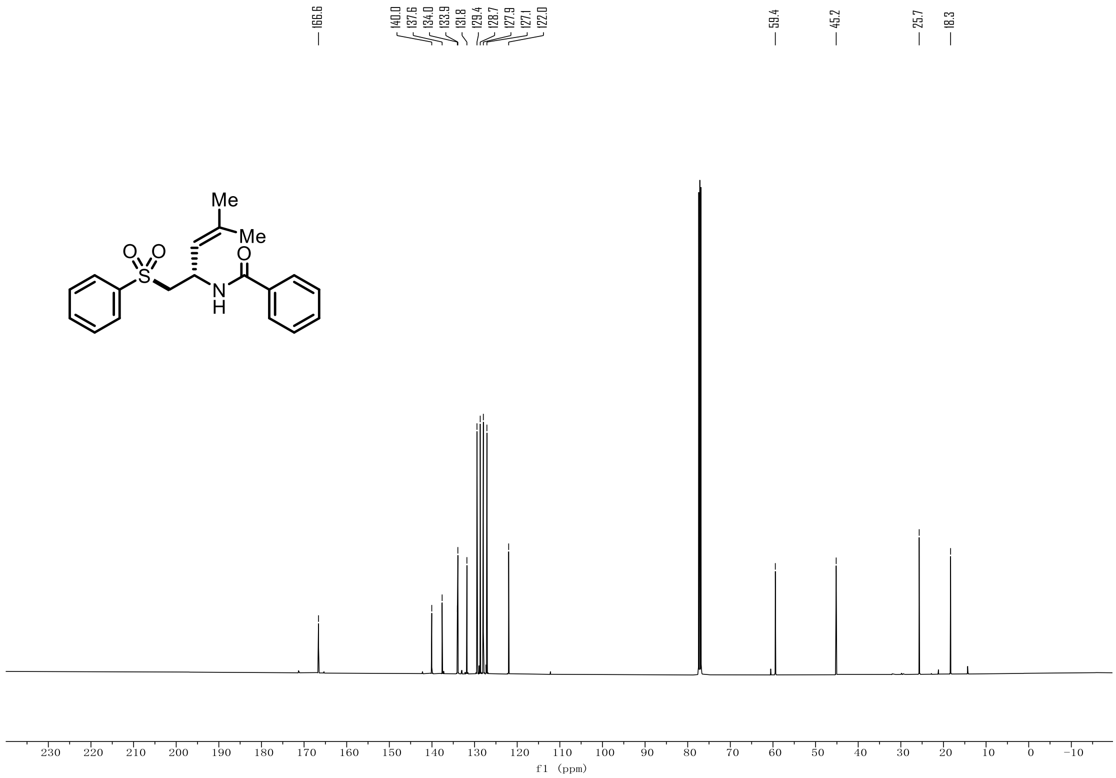

(*S*)-*N*-(1-(1H-Inden-2-yl)-2-(phenylsulfonyl)ethyl)benzamide (29)

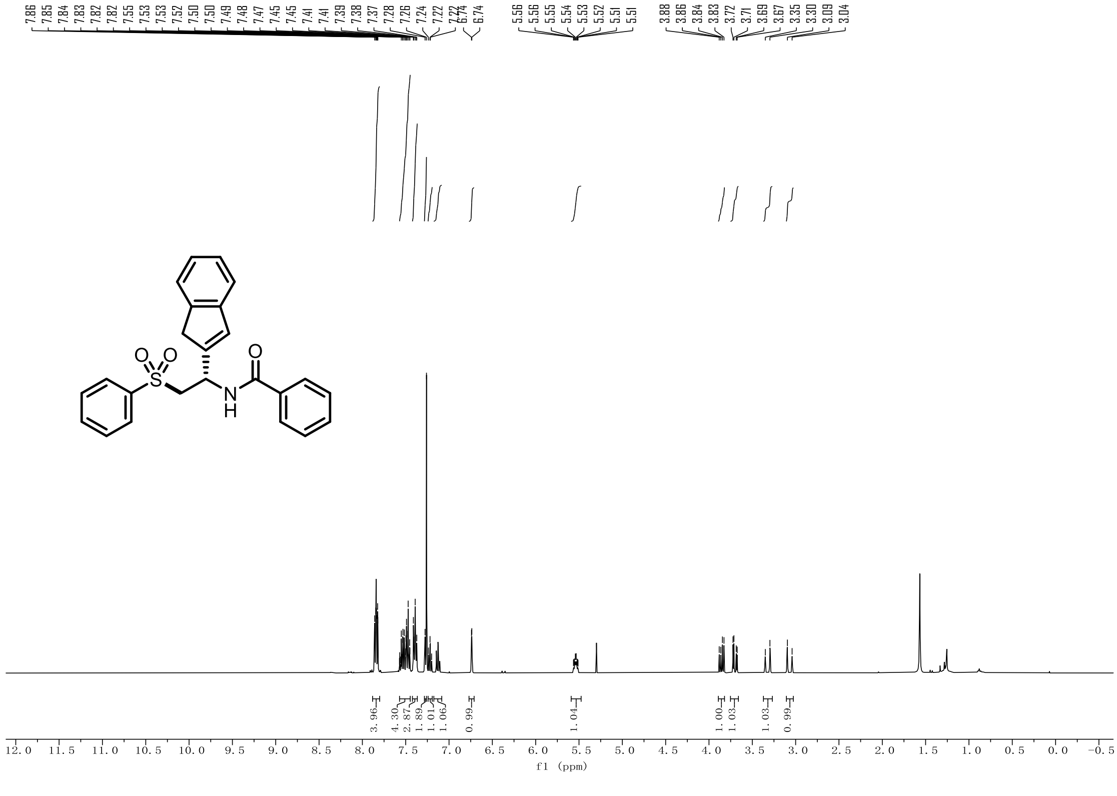

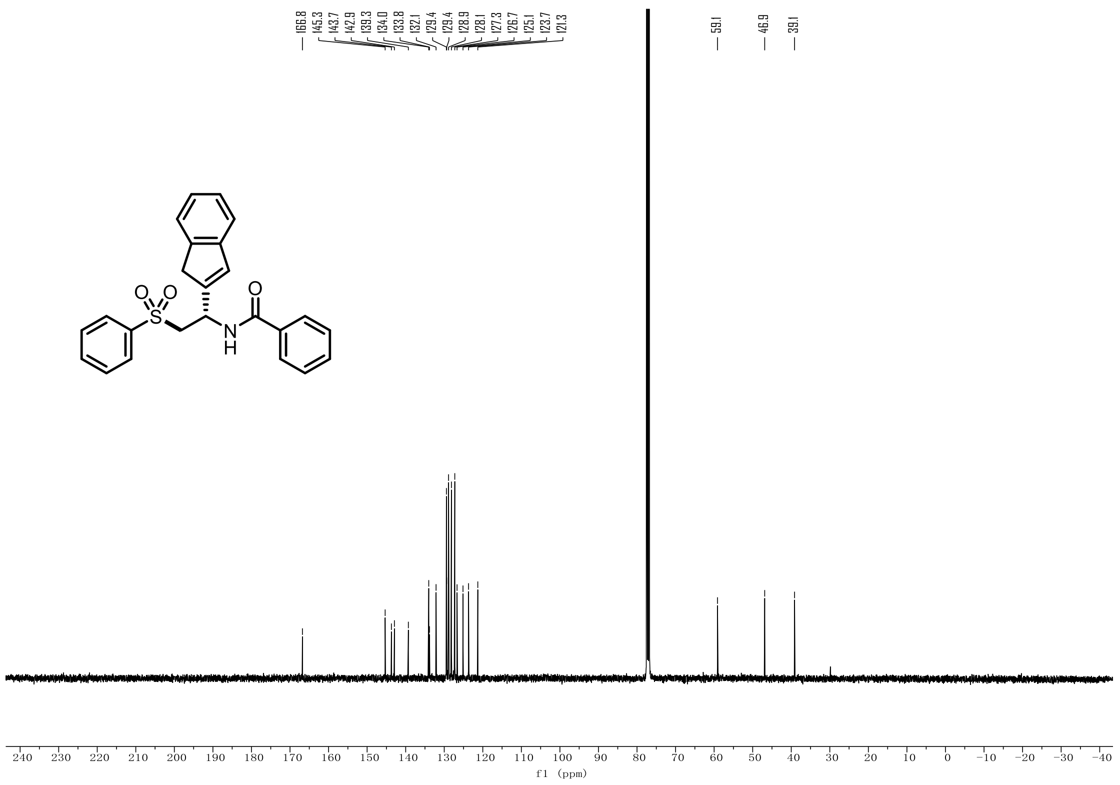

(*S*)-*N*-(1-(4-Cyanophenyl)-2-(phenylsulfonyl)ethyl)-4-methoxybenzamide (30).

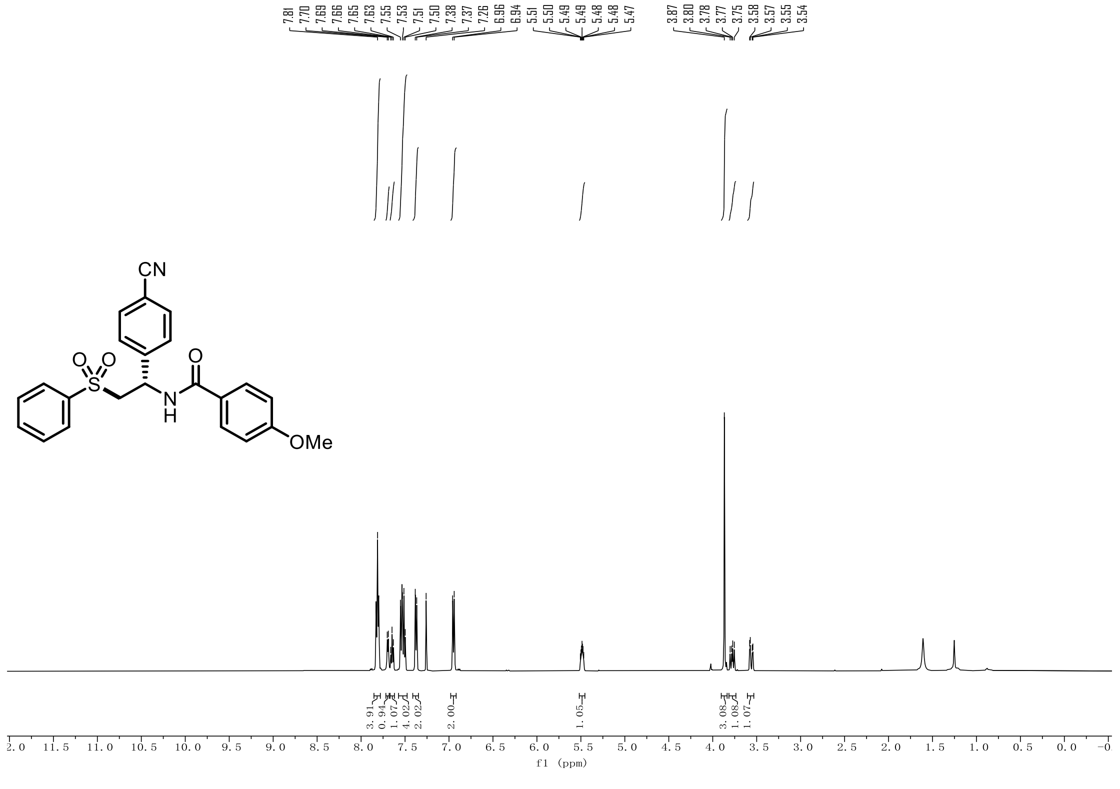

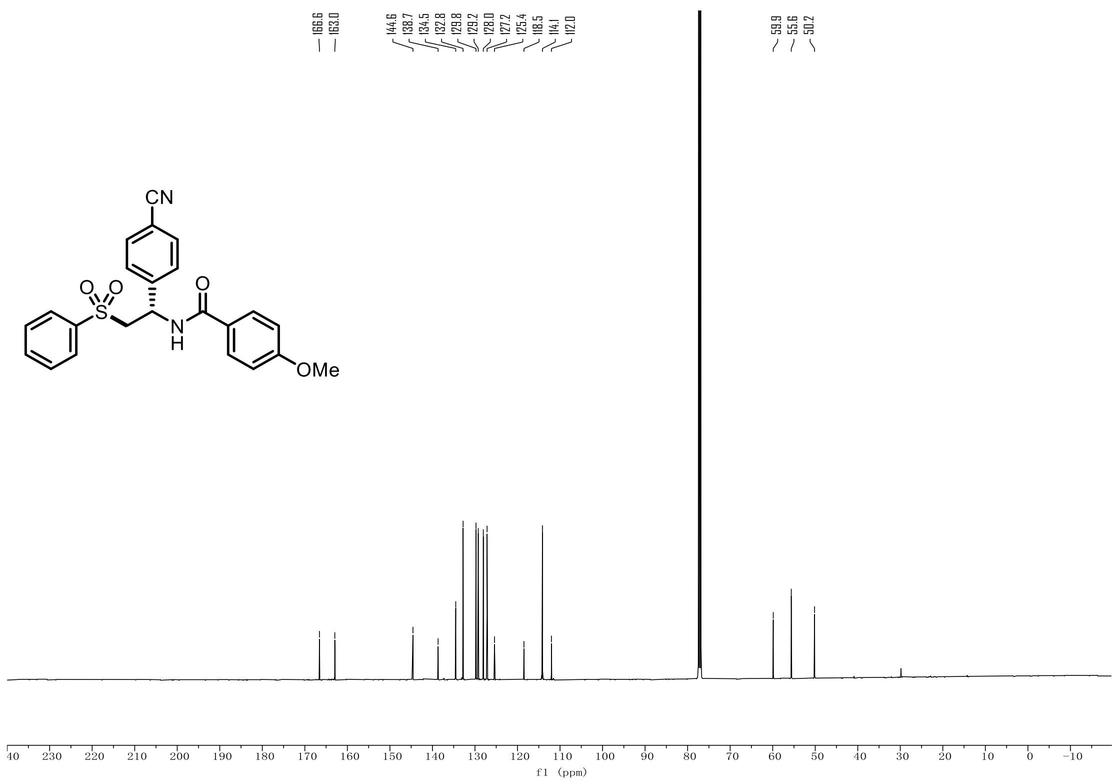

(*S*)-4-Methoxy-*N*-(1-(3-methoxyphenyl)-2-(phenylsulfonyl)ethyl)benzamide (31).

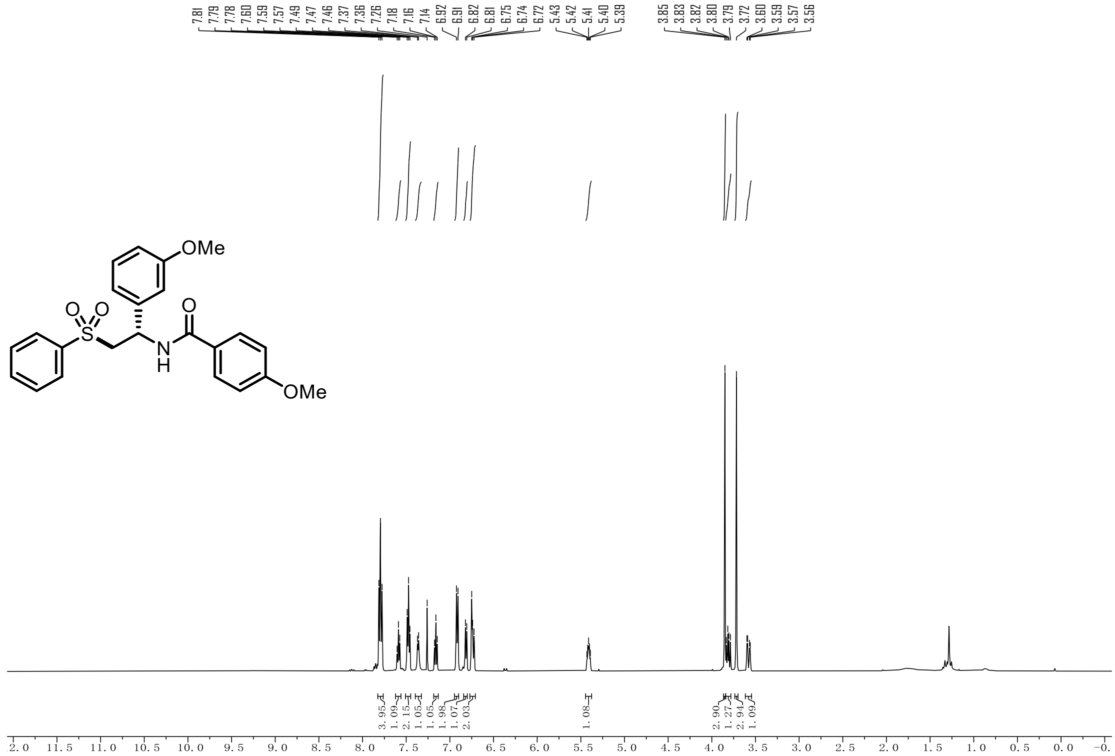

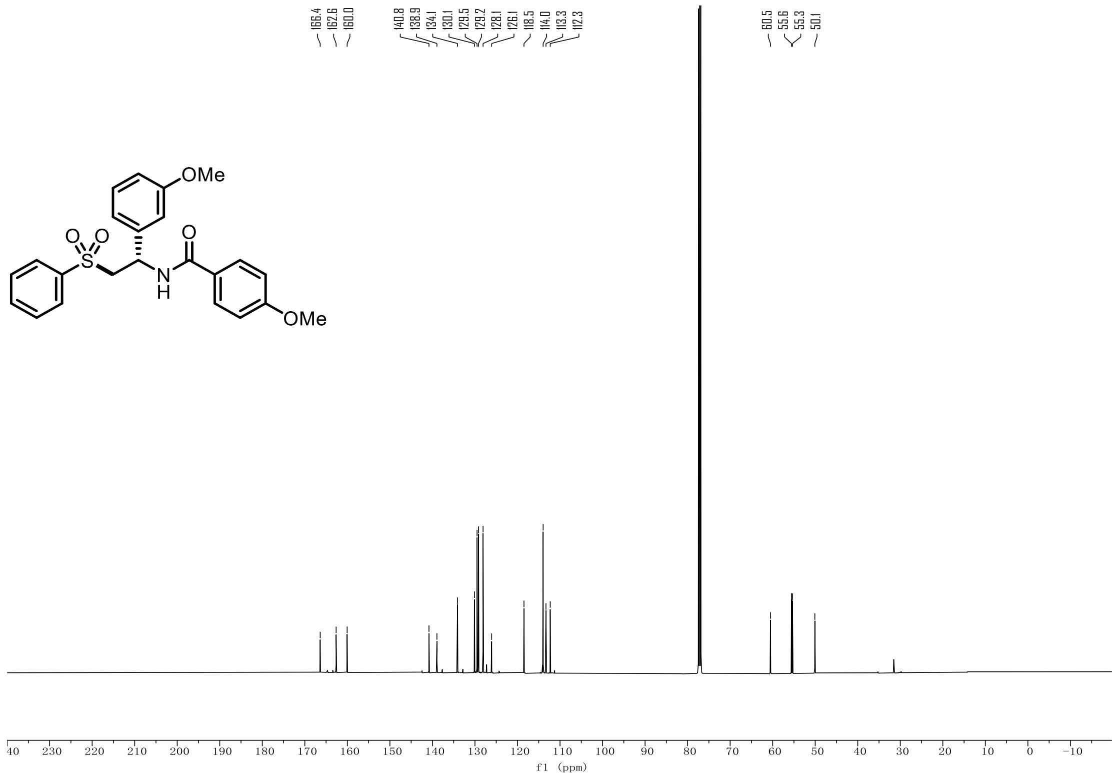

(*S*)-*N*-(1-(4-Cyanophenyl)-2-(phenylsulfonyl)ethyl)-4-methylbenzamide (32).

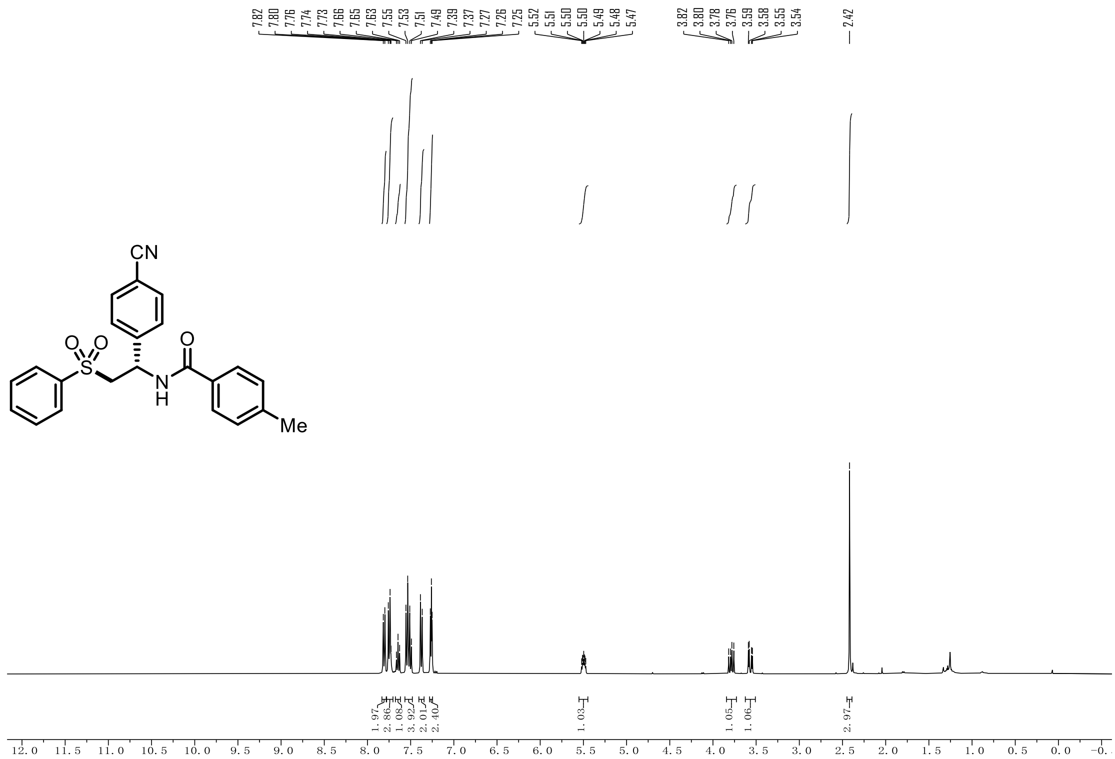

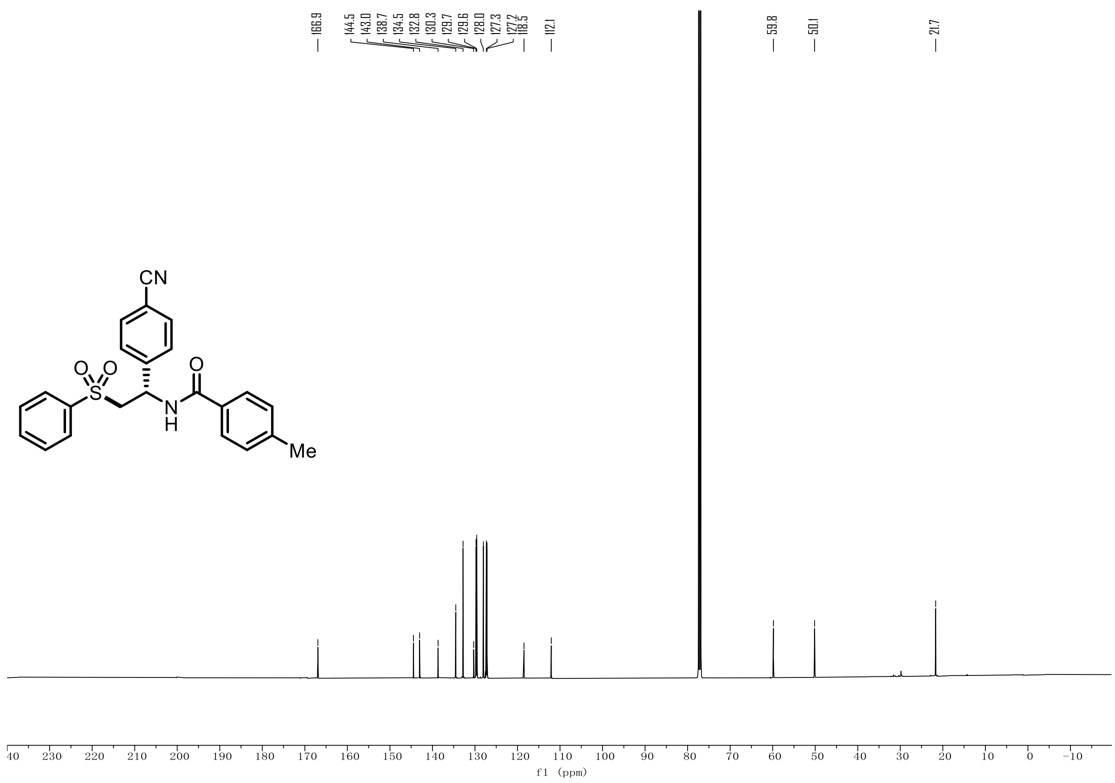

(*S*)-*N*-(1-(4-Cyanophenyl)-2-(phenylsulfonyl)ethyl)-4-fluorobenzamide (33).

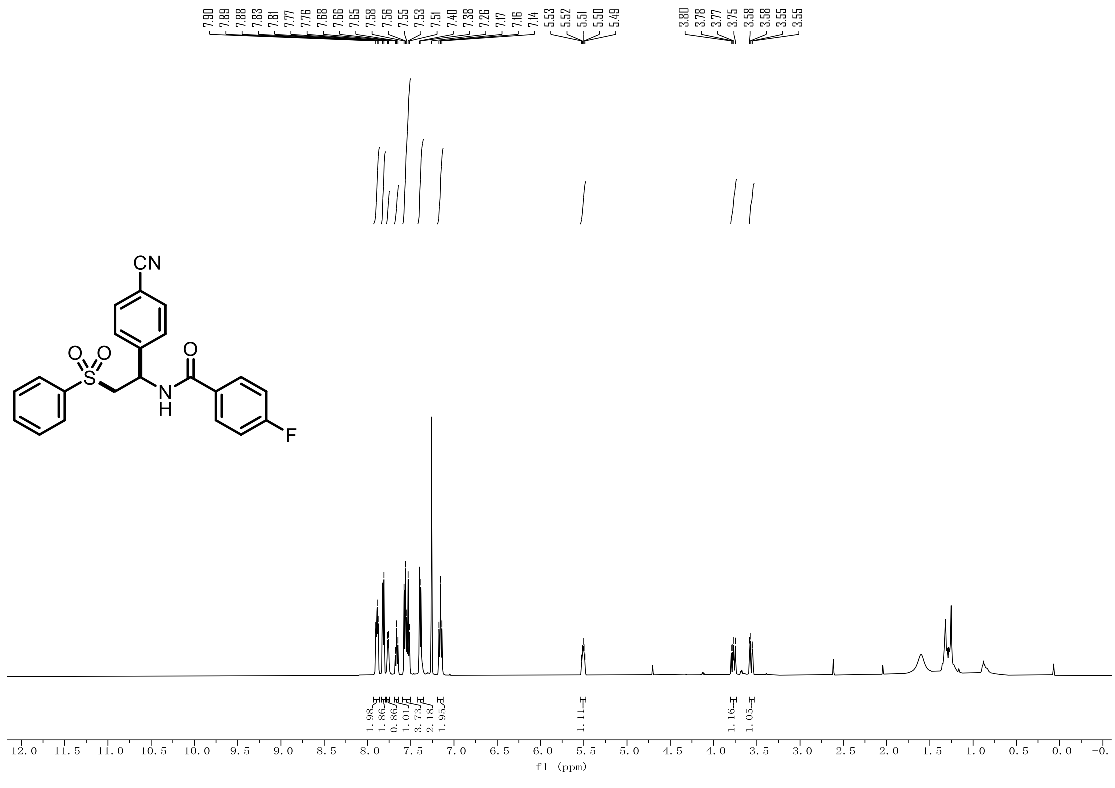

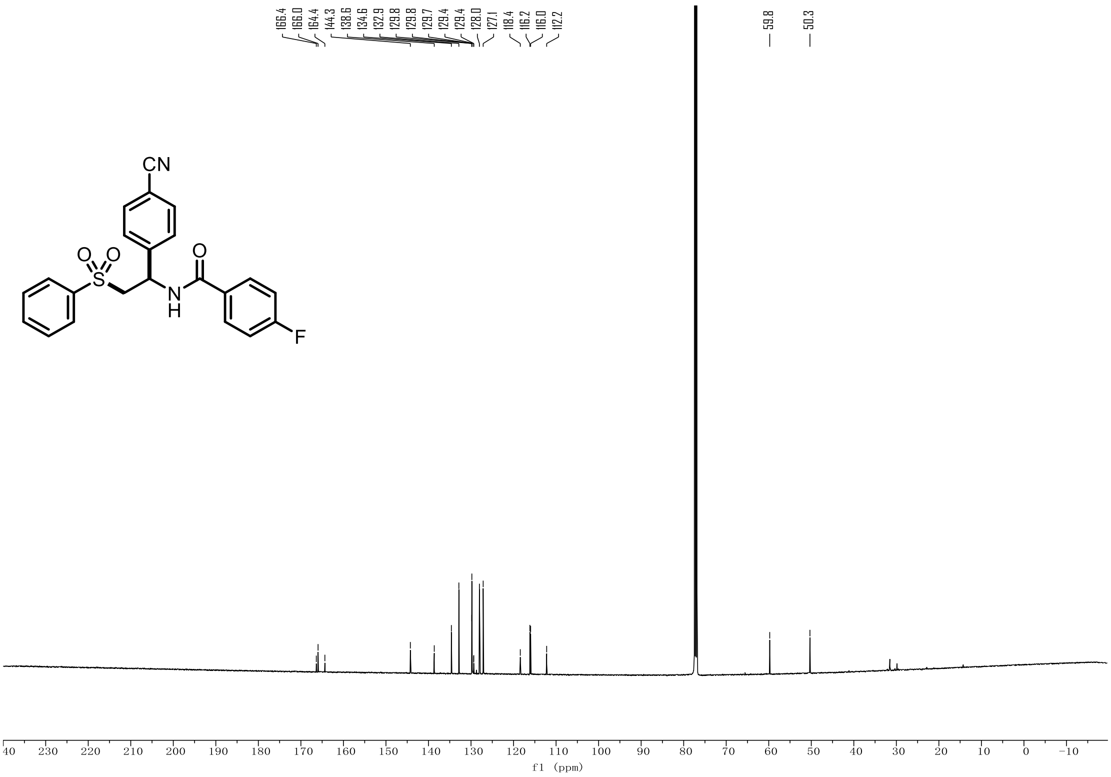
166.4
166.0
164.4
144.3
138.6
134.6
132.9
129.8
129.8
129.7
129.4
129.4
128.0
127.1
118.4
116.2
116.0
112.2
59.8
50.3
CN
O
S
O
O
N
H
F
f1 (ppm)

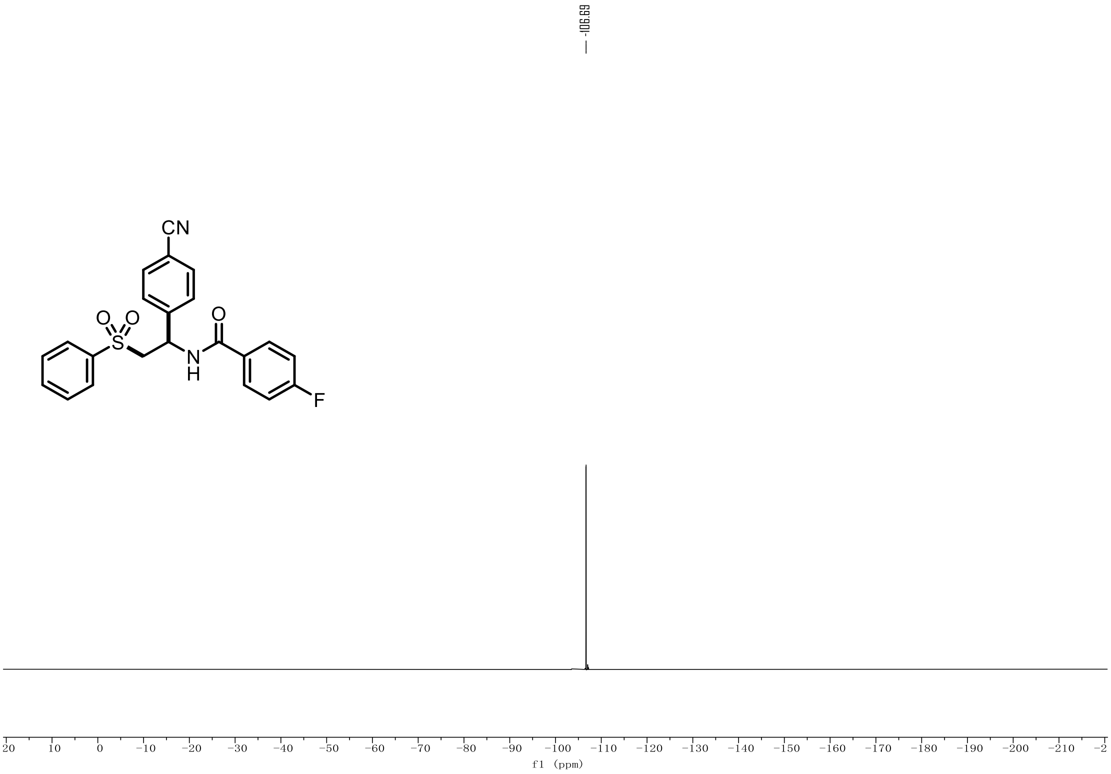
-106.69
CN
O
S
O
O
N
H
F
f1 (ppm)

(*S*)-4-chloro-*N*-(1-(4-cyanophenyl)-2-(phenylsulfonyl)ethyl)benzamide (34).

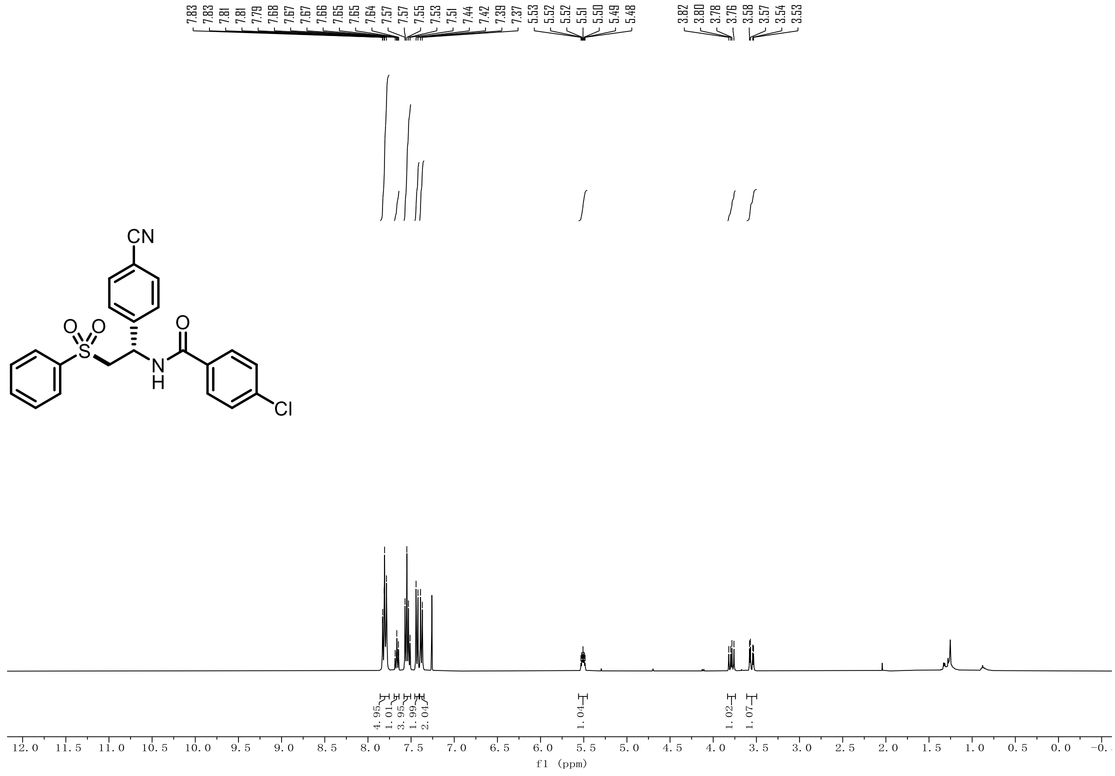

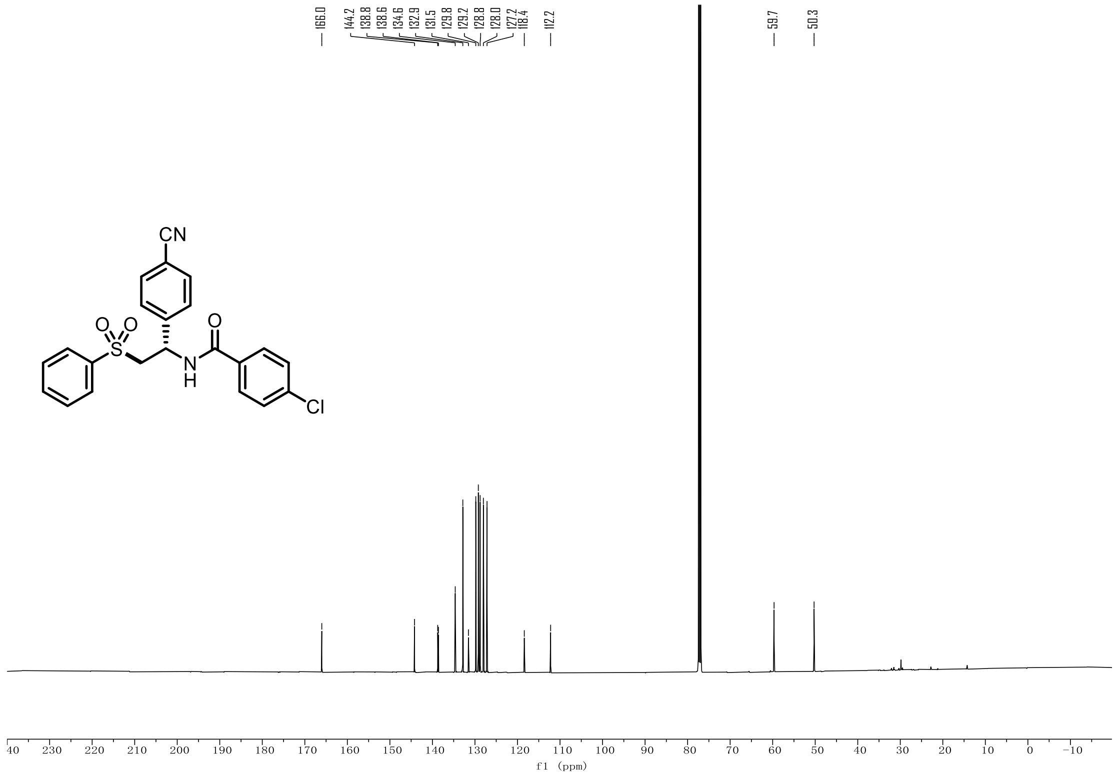

(*S*)-*N*-(1-(4-Cyanophenyl)-2-(phenylsulfonyl)ethyl)-4-(trifluoromethyl)benzamide (35).

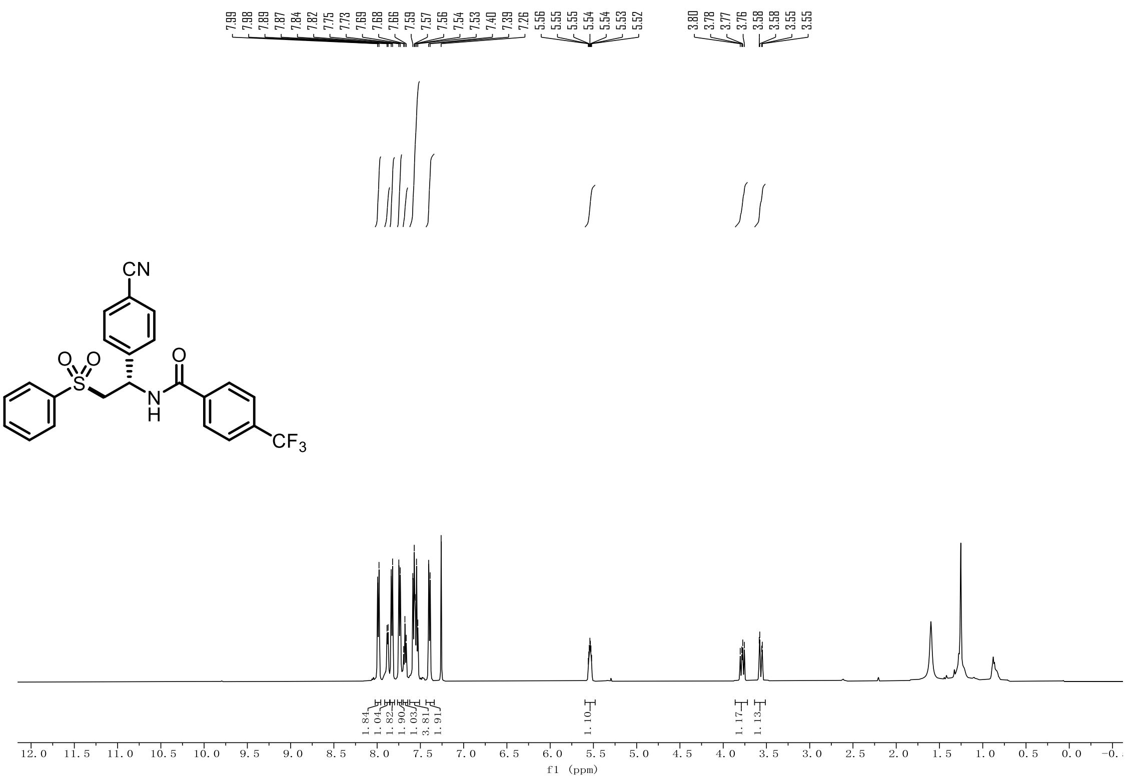

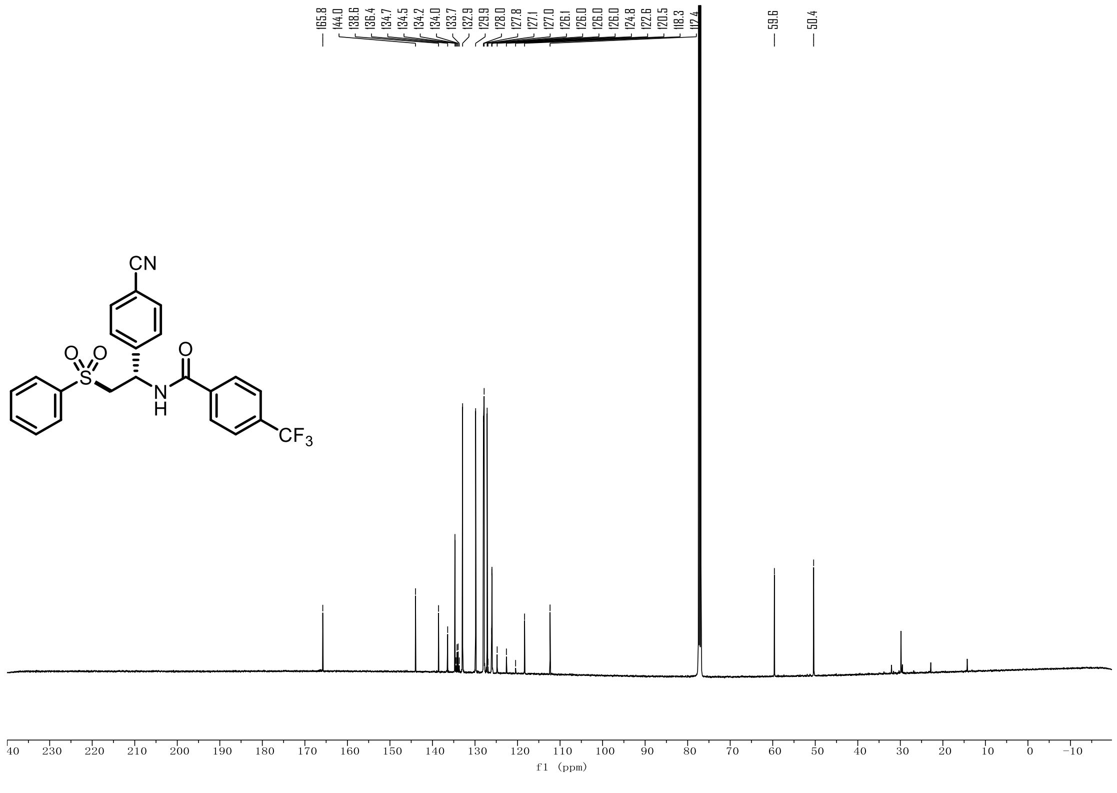

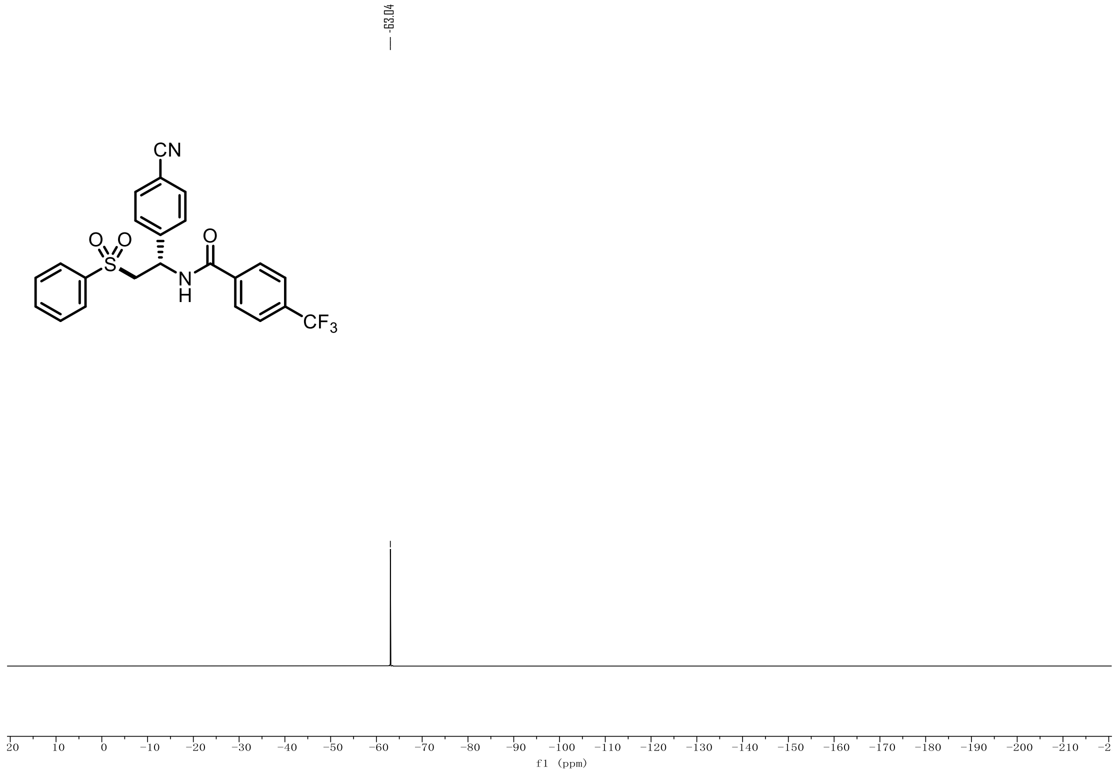

(*S*)-4-Cyano-*N*-(1-(4-cyanophenyl)-2-(phenylsulfonyl)ethyl)benzamide (36).

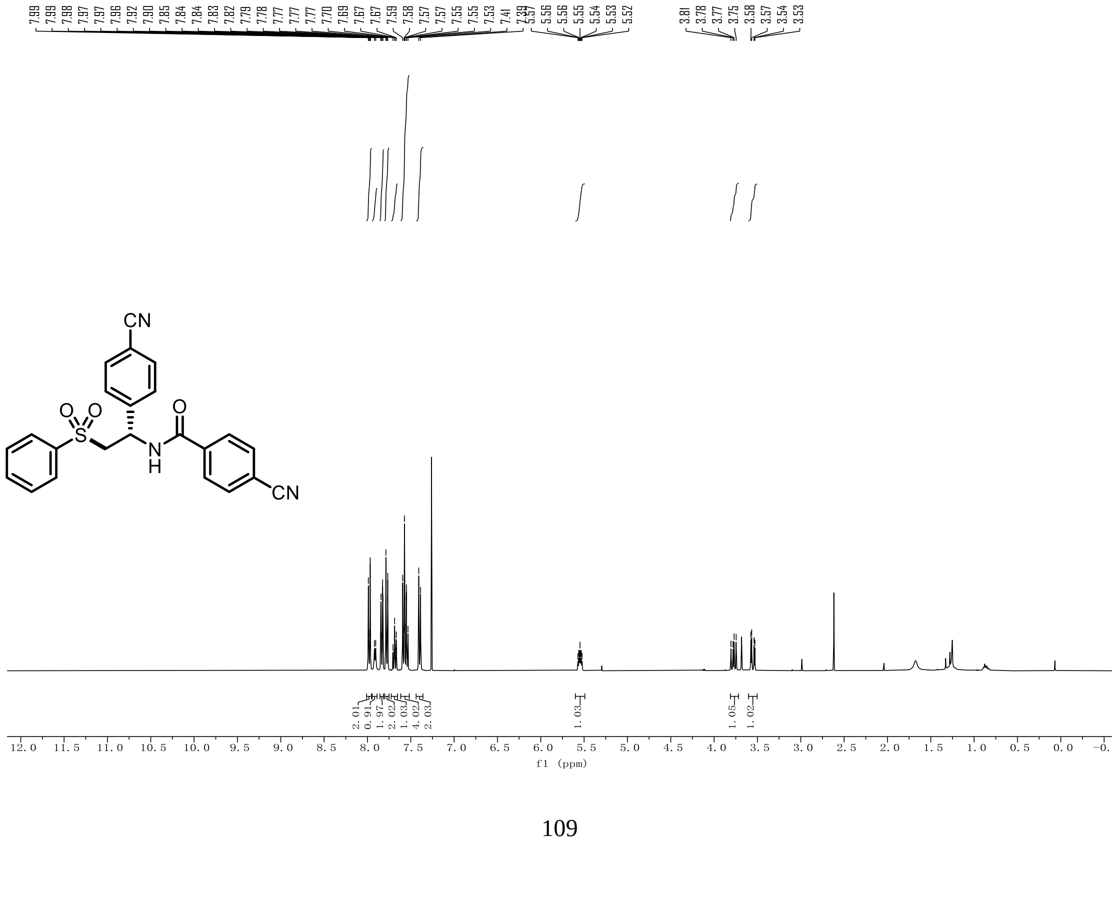

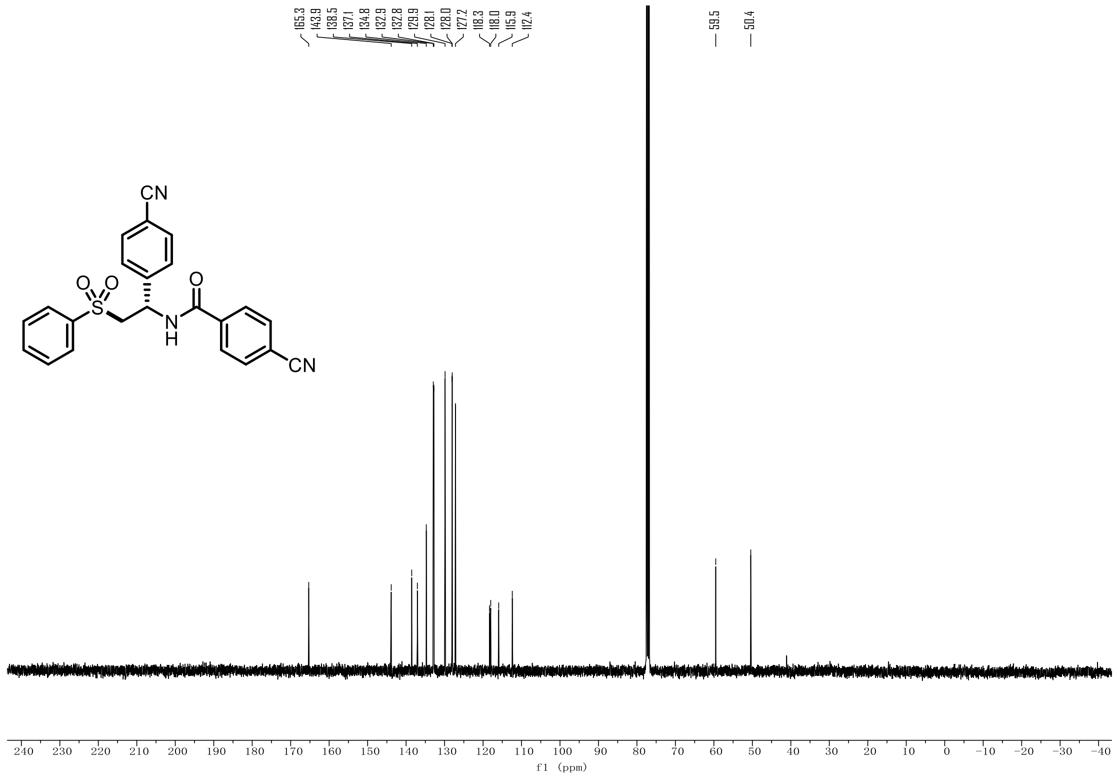

(*S*)-4-Cyano-*N*-(1-(3-methoxyphenyl)-2-(phenylsulfonyl)ethyl)benzamide (37).

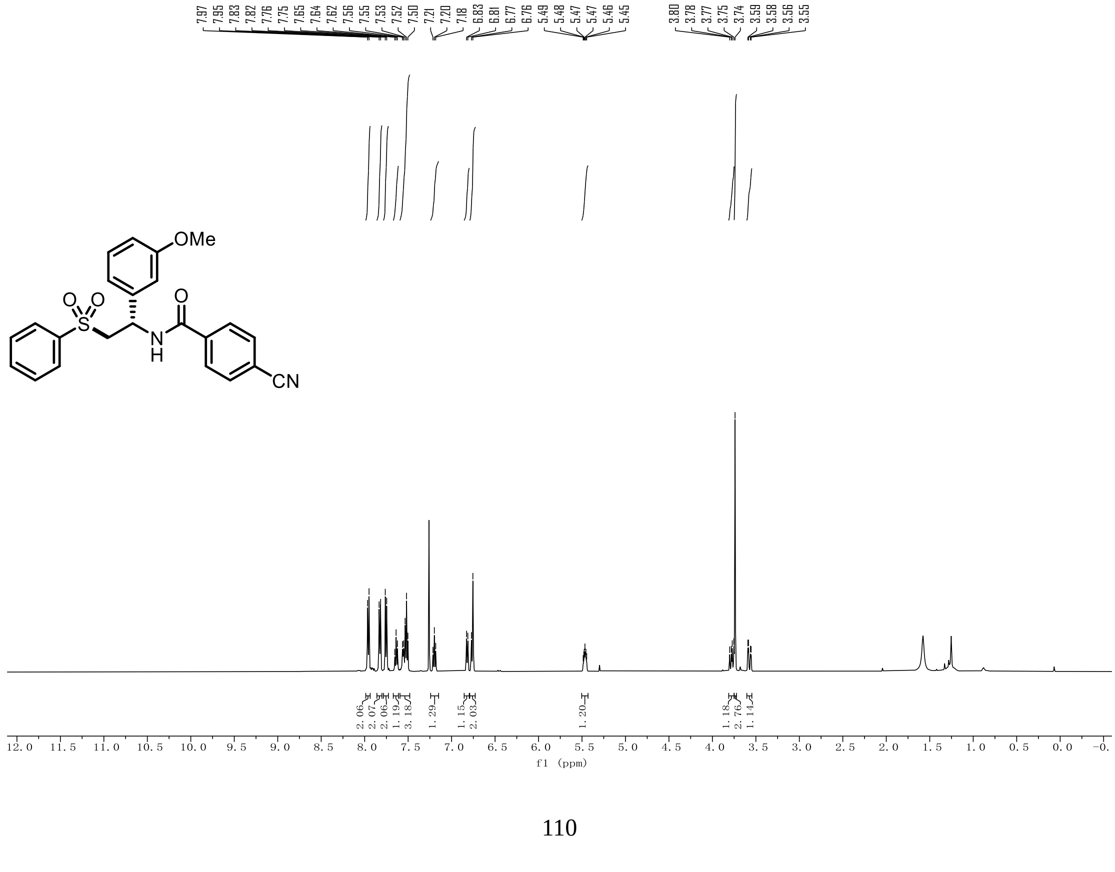

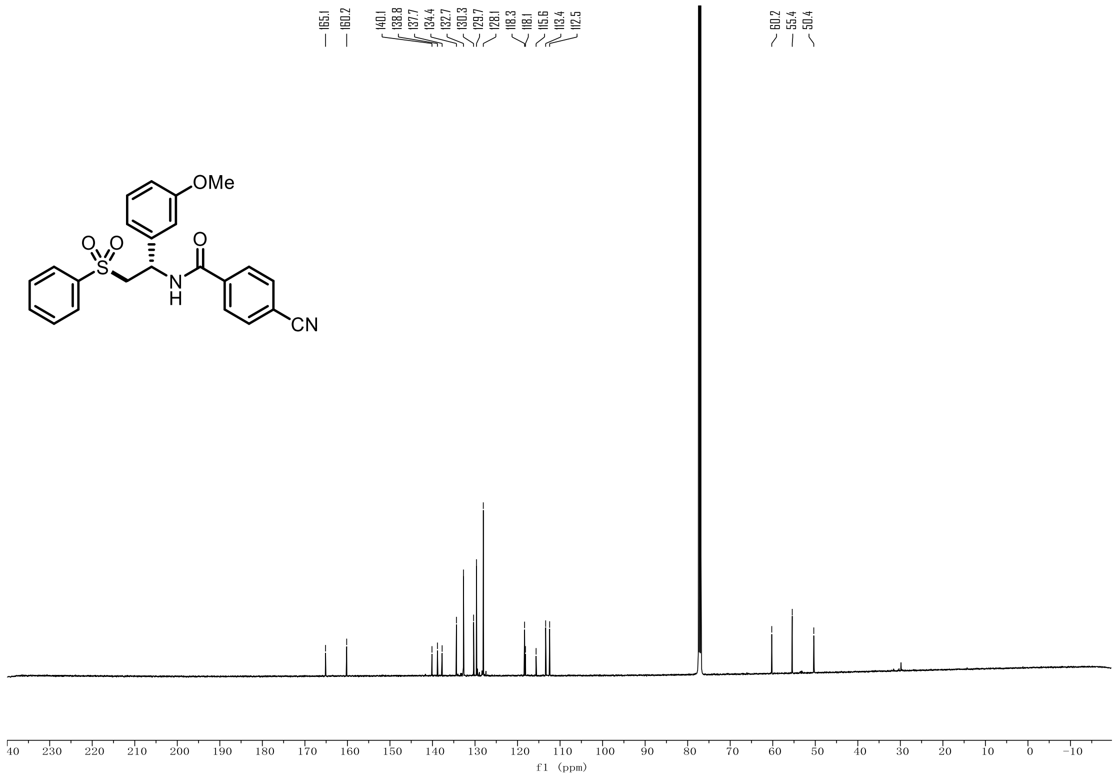

(*S*)-3-Chloro-*N*-(1-(4-cyanophenyl)-2-(phenylsulfonyl)ethyl)benzamide (38).

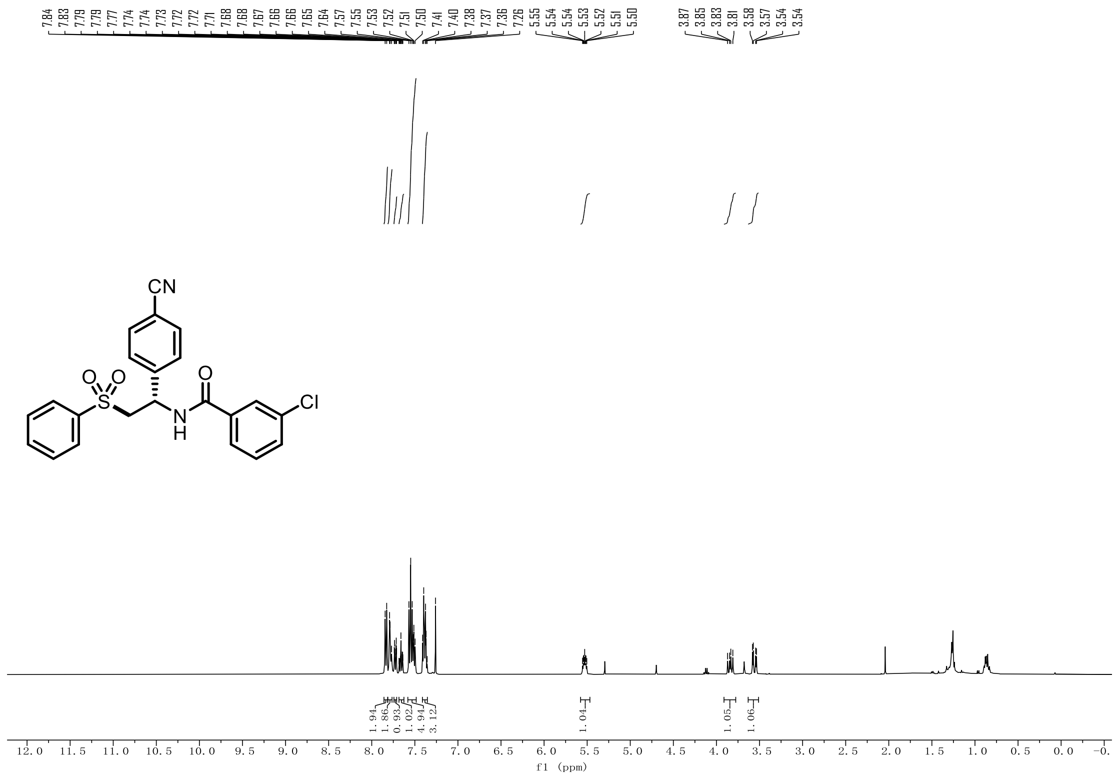

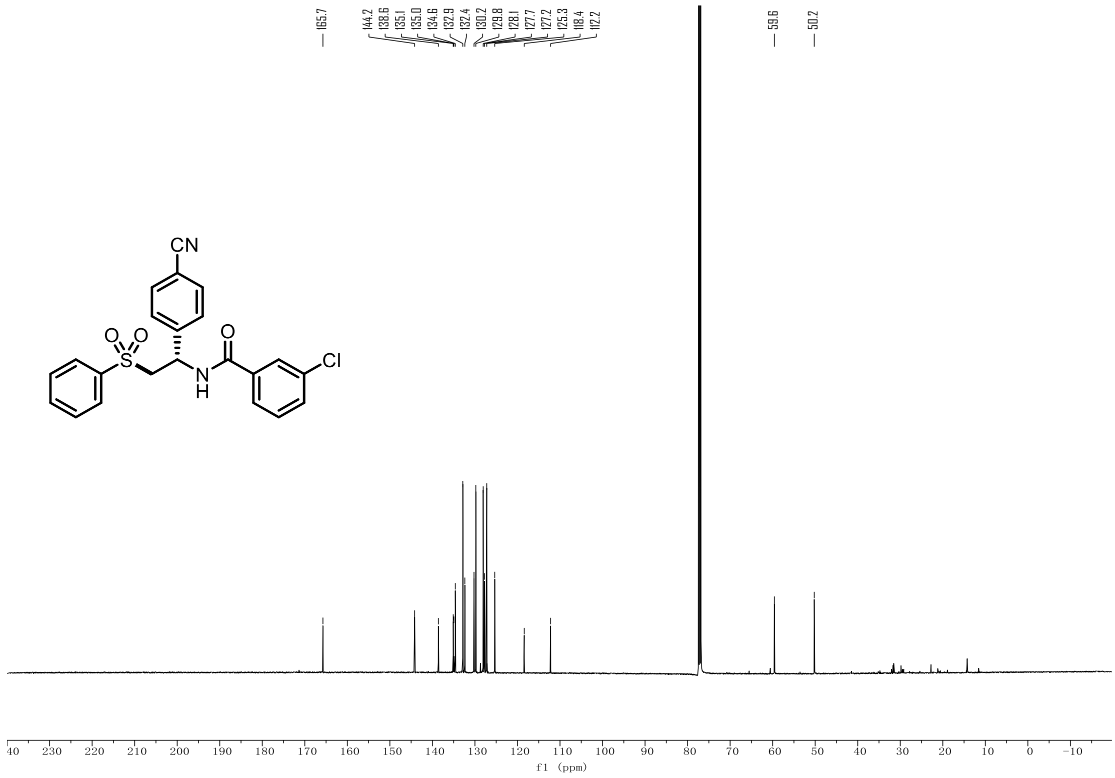

(*S*)-*N*-(1-(4-Cyanophenyl)-2-(phenylsulfonyl)ethyl)-2-fluorobenzamide (39).

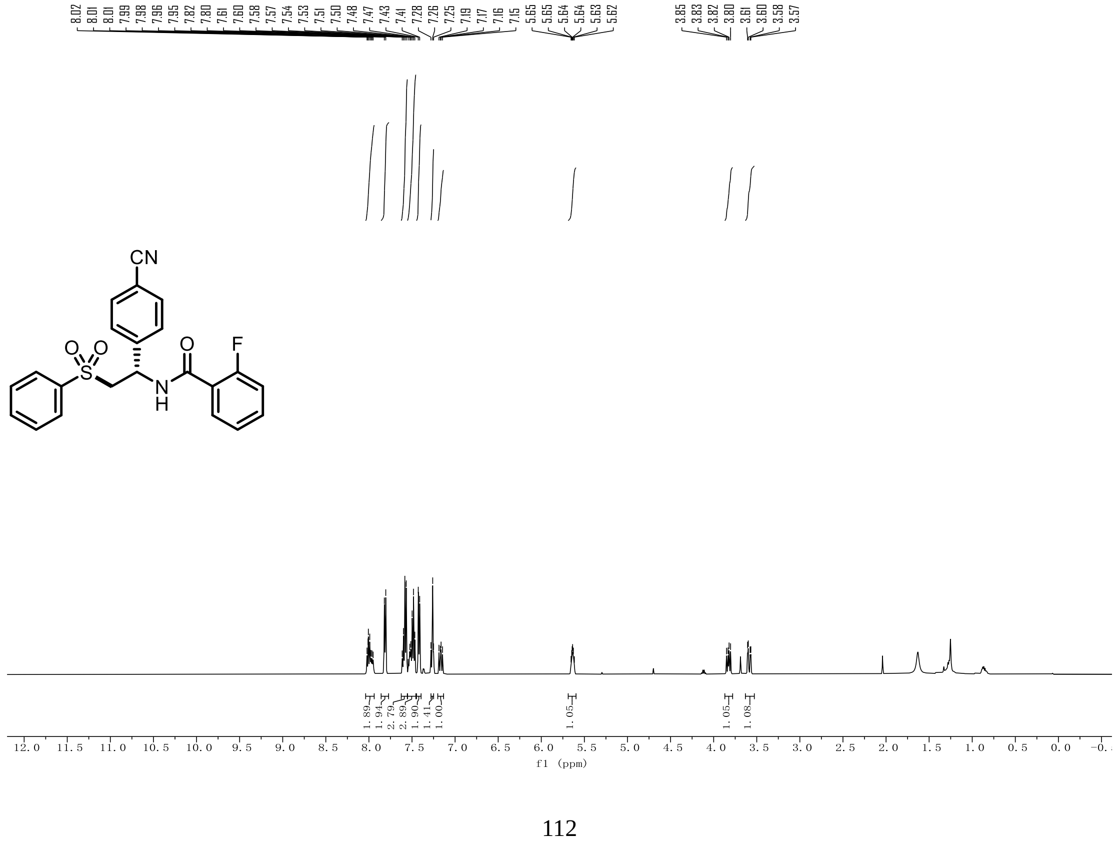

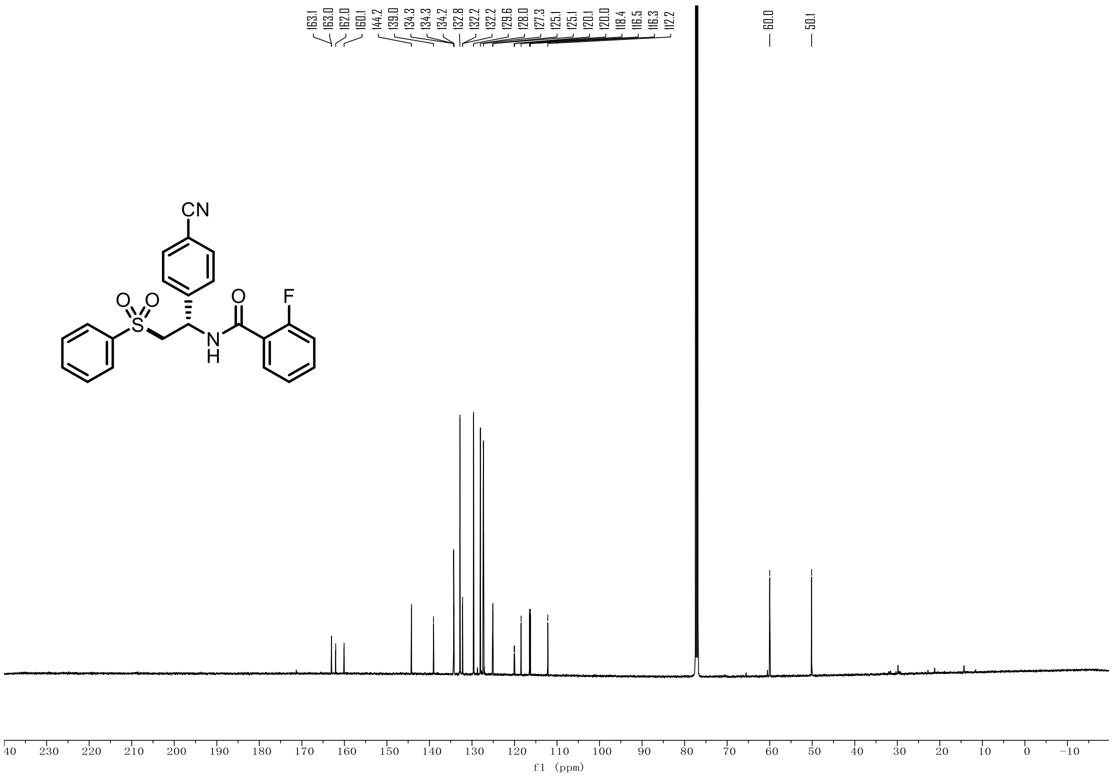
163.1
163.0
162.0
160.1
144.2
139.0
134.3
134.3
134.2
132.8
132.2
132.2
129.6
128.0
127.3
125.1
125.1
120.1
120.0
118.4
116.5
116.3
112.2
60.0
50.1
CN
O
F
S
N
H
f1 (ppm)

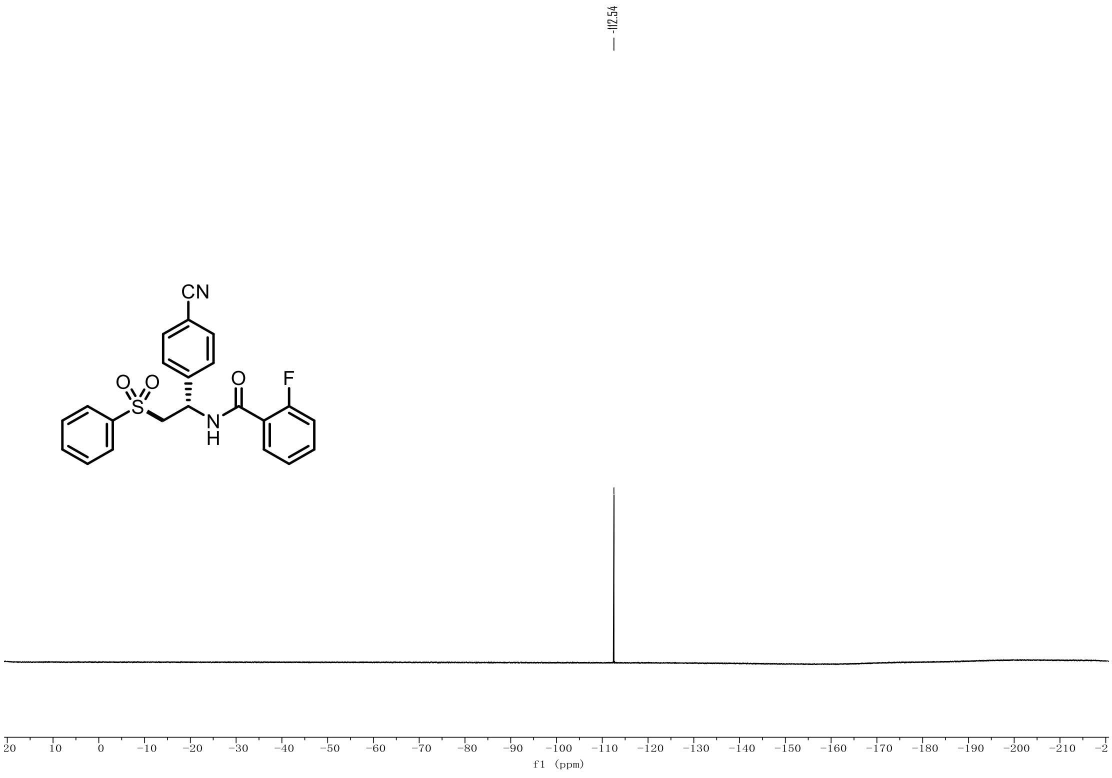
-112.54
CN
O
F
S
N
H
f1 (ppm)

(*S*)-2-Chloro-*N*-(1-(4-cyanophenyl)-2-(phenylsulfonyl)ethyl)benzamide (40).

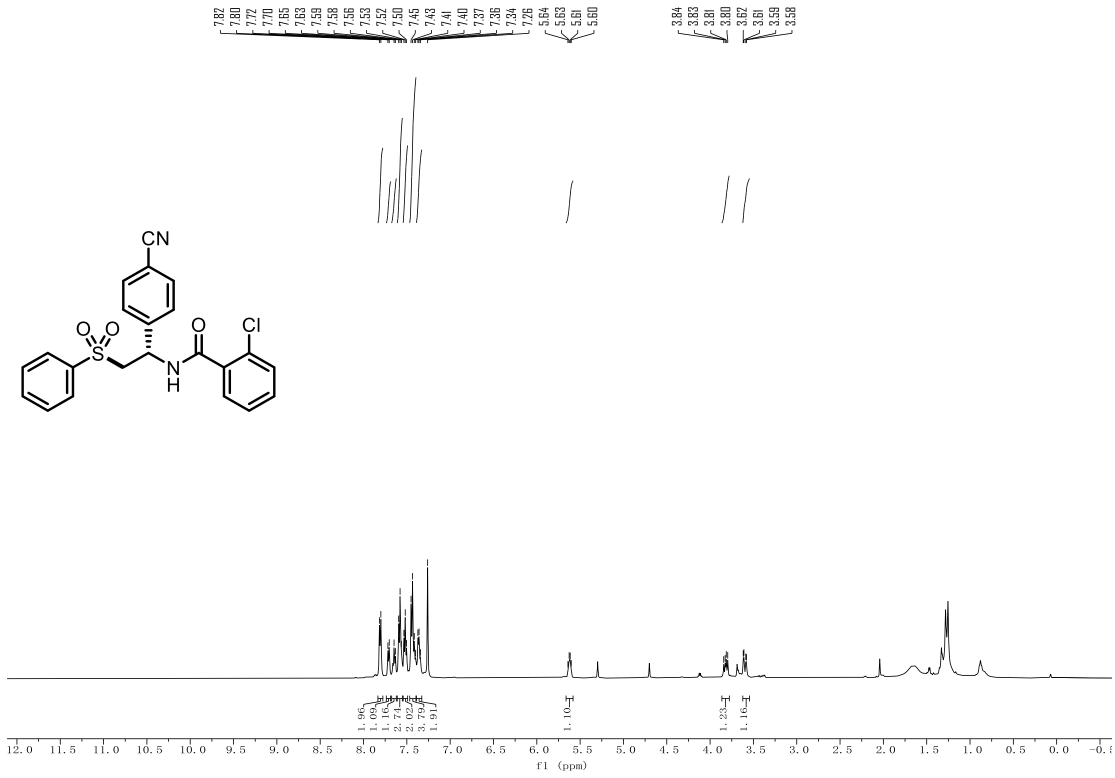

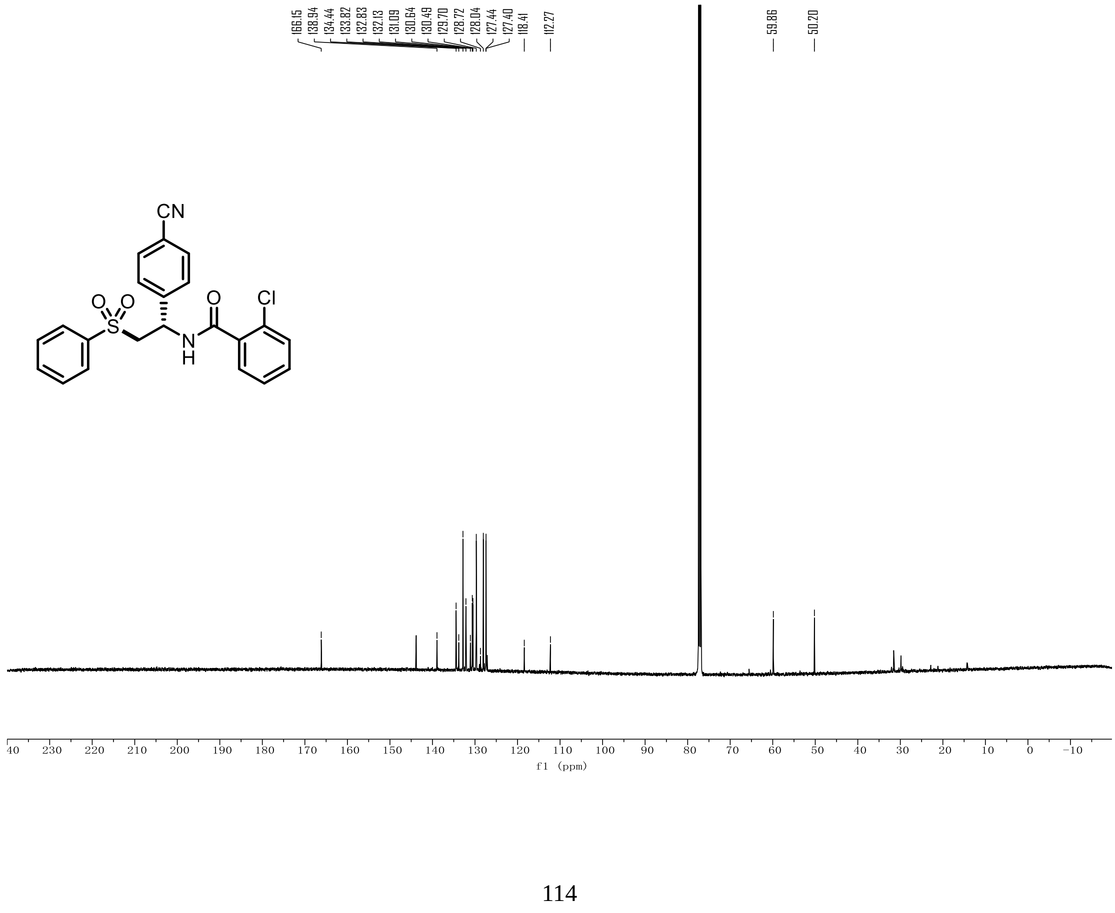

(*S*)-*N*-(1-(4-Cyanophenyl)-2-(phenylsulfonyl)ethyl)-2-naphthamide (41).

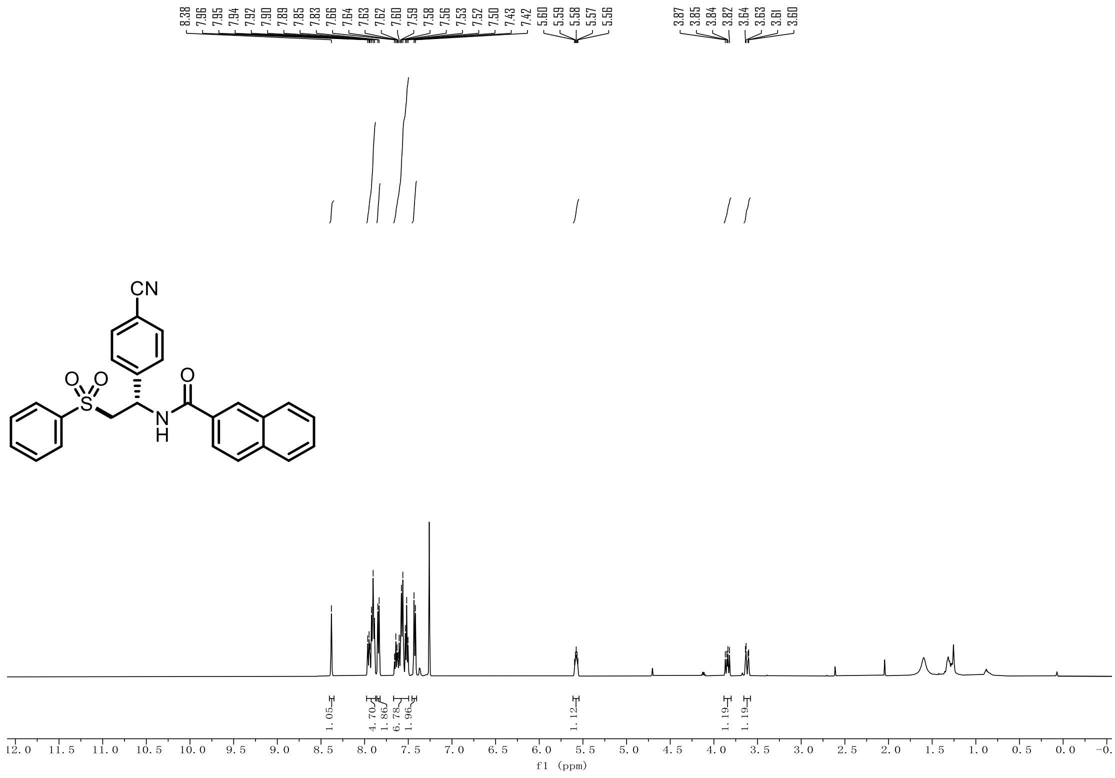

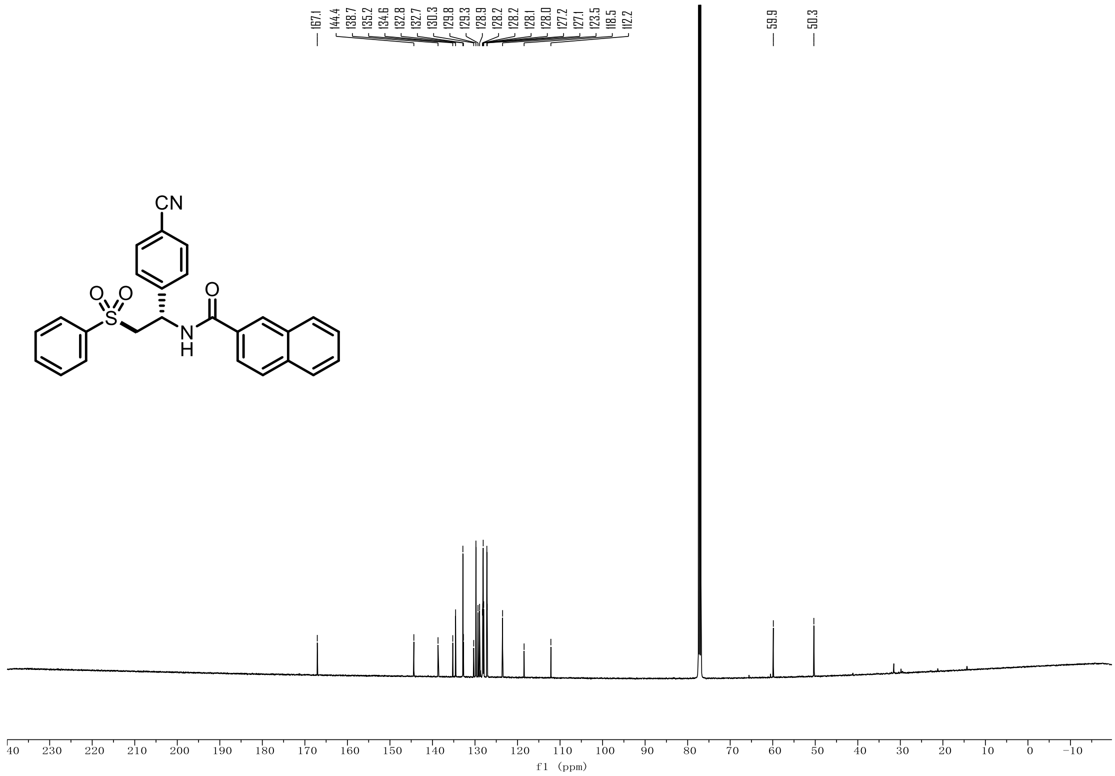

(*S*)-5-Chloro-*N*-(1-(4-cyanophenyl)-2-(phenylsulfonyl)ethyl)nicotinamide (42)

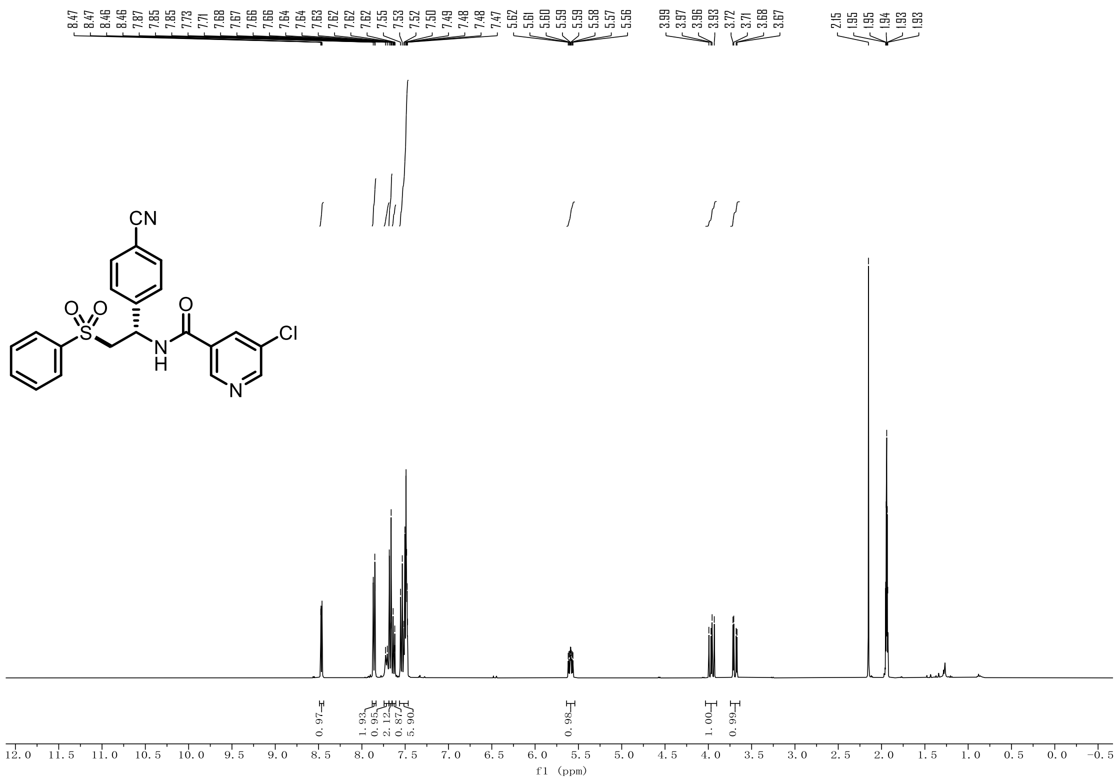

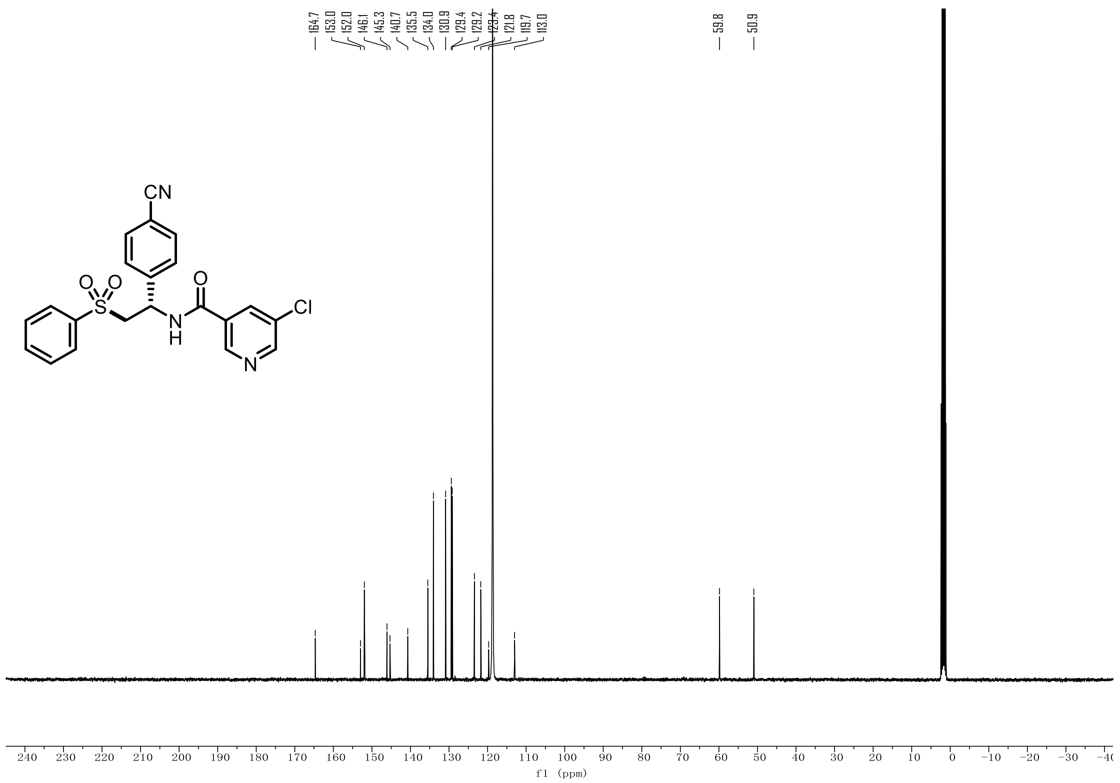

(*S*)-*N*-(1-(4-Cyanophenyl)-2-(phenylsulfonyl)ethyl)pivalamide (43).

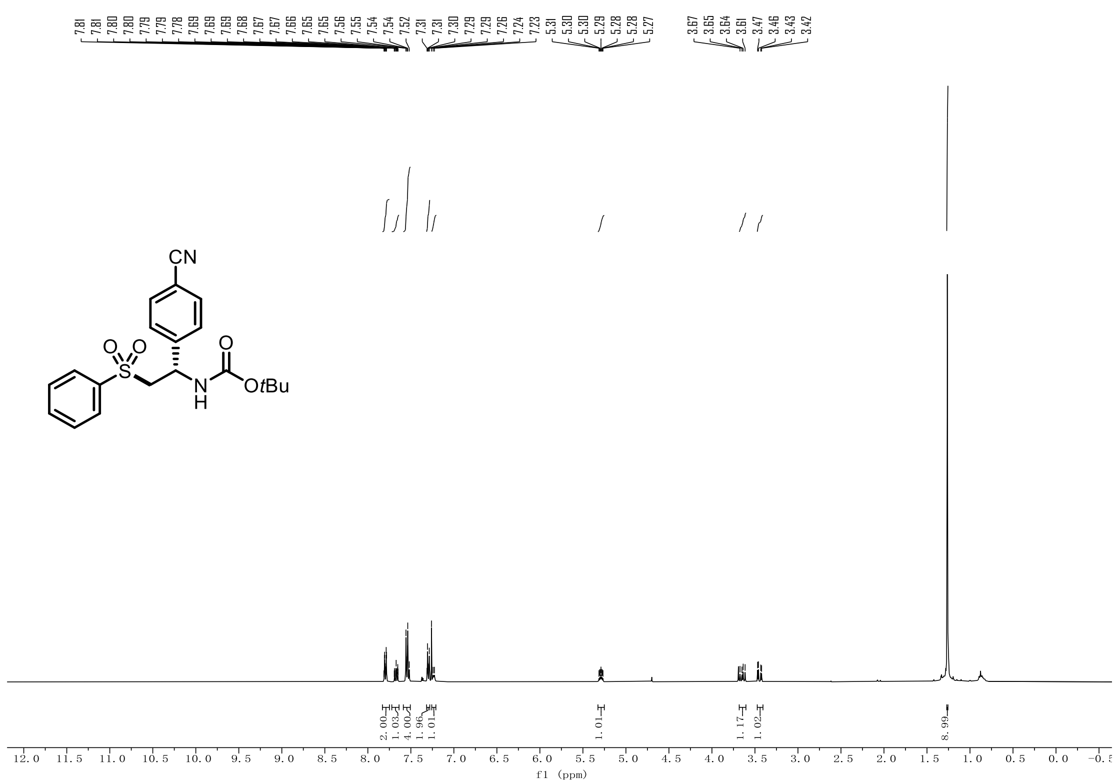

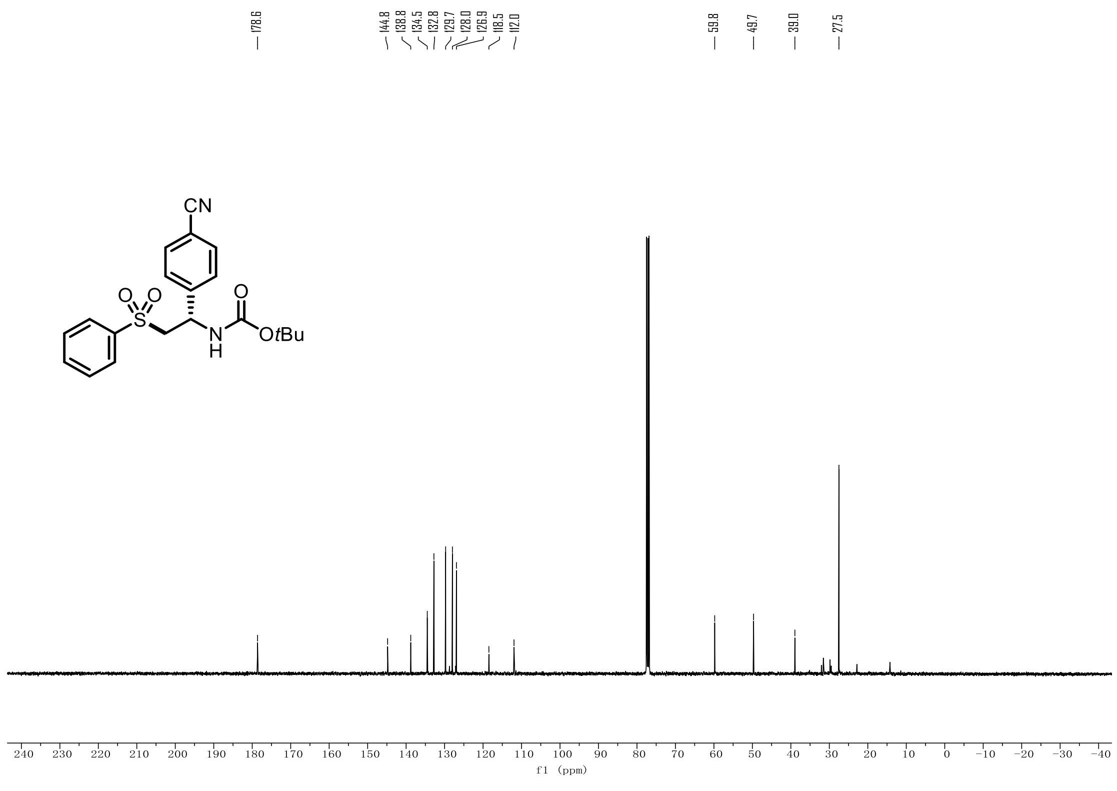

Tert-butyl (*S*)-(2-(phenylsulfonyl)-1-(p-tolyl)ethyl)carbamate (44)

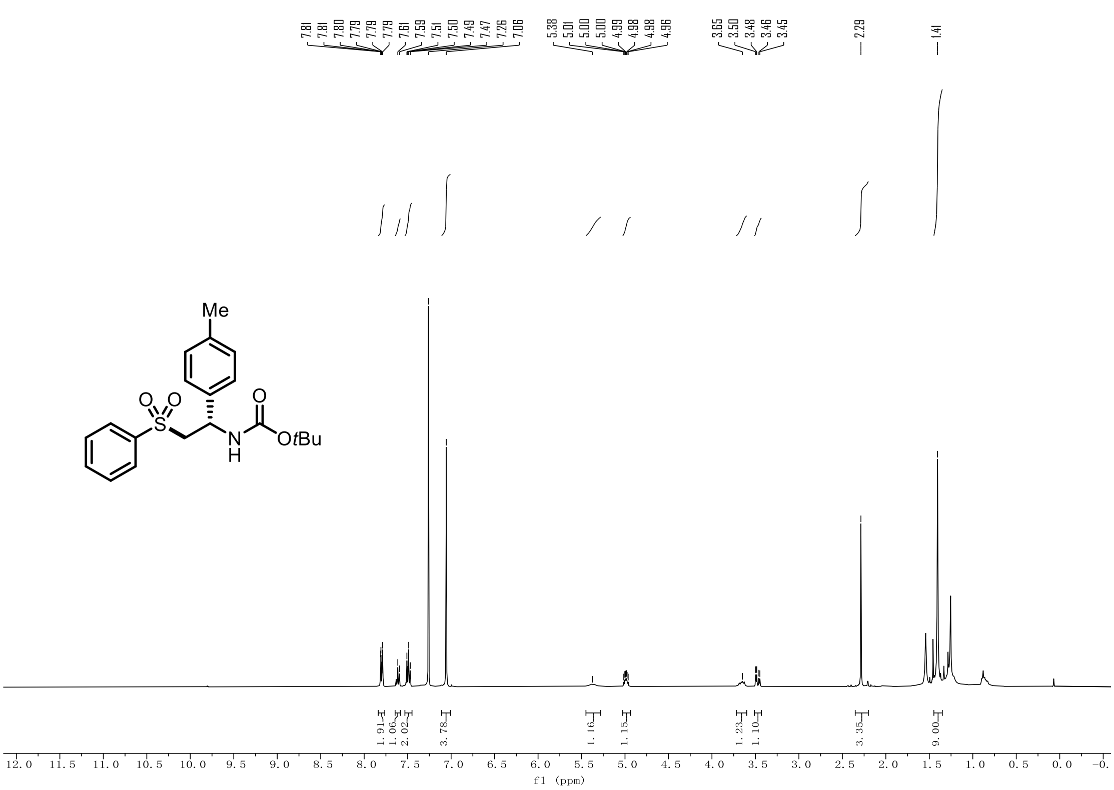

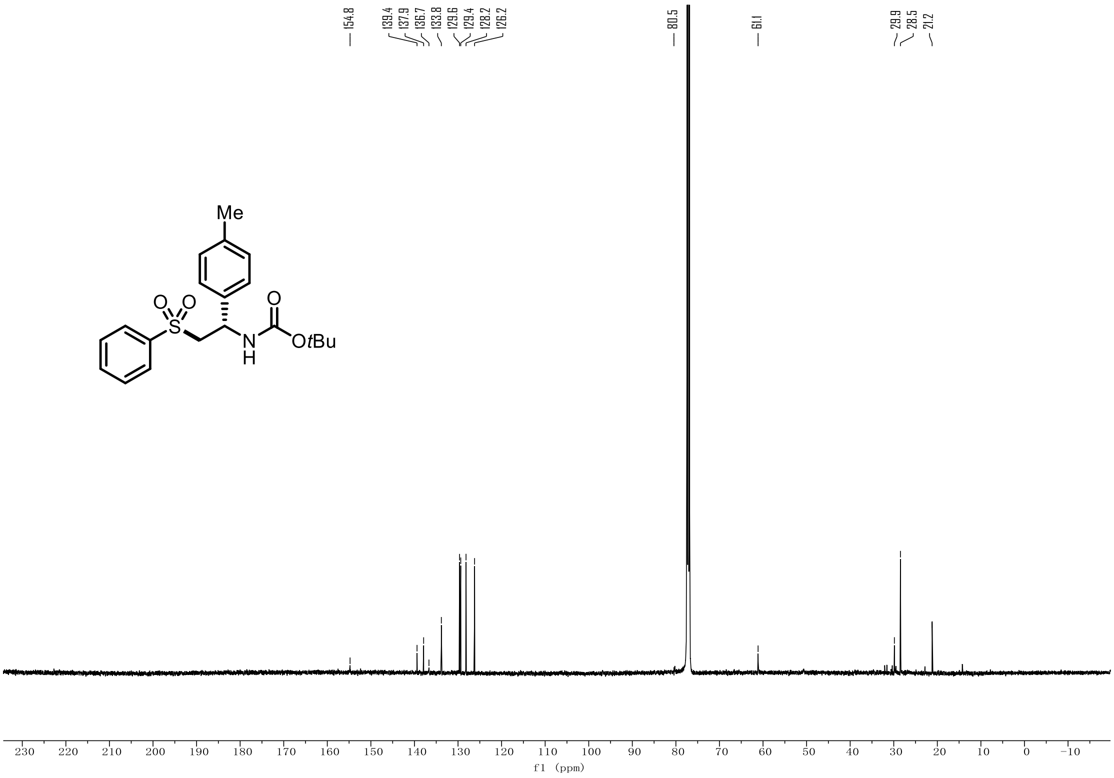

Tert-butyl (*S*)-(1-(3-methoxyphenyl)-2-(phenylsulfonyl)ethyl)carbamate (45)

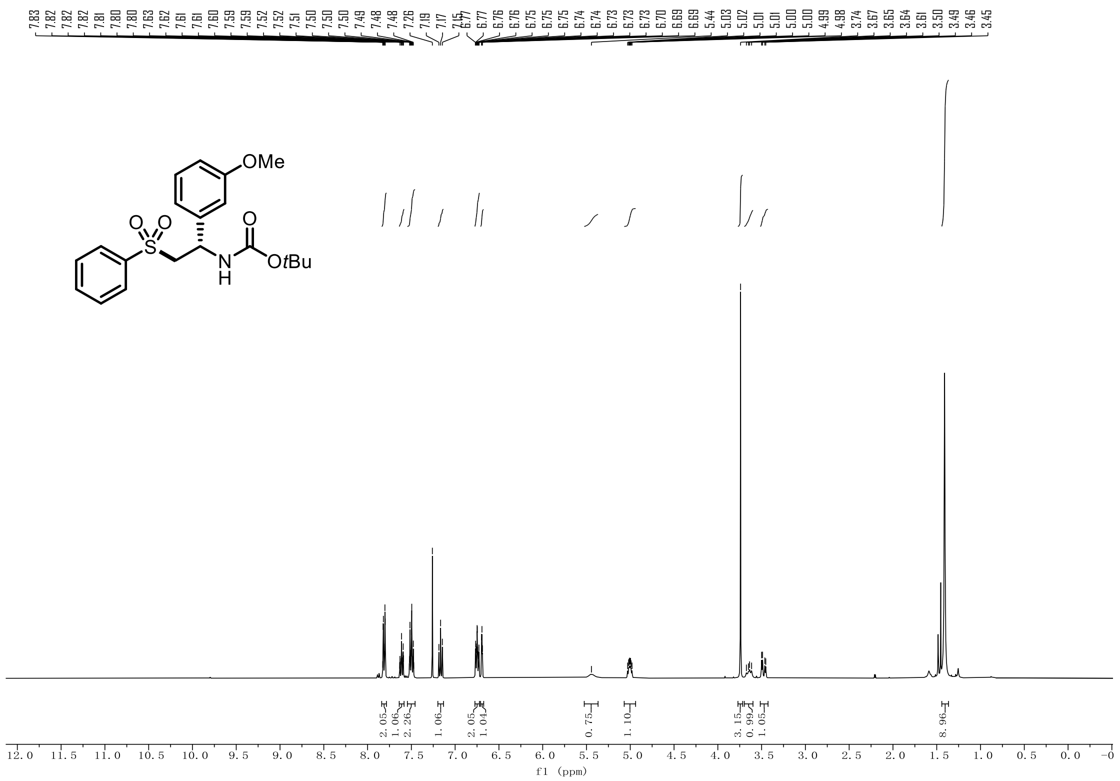

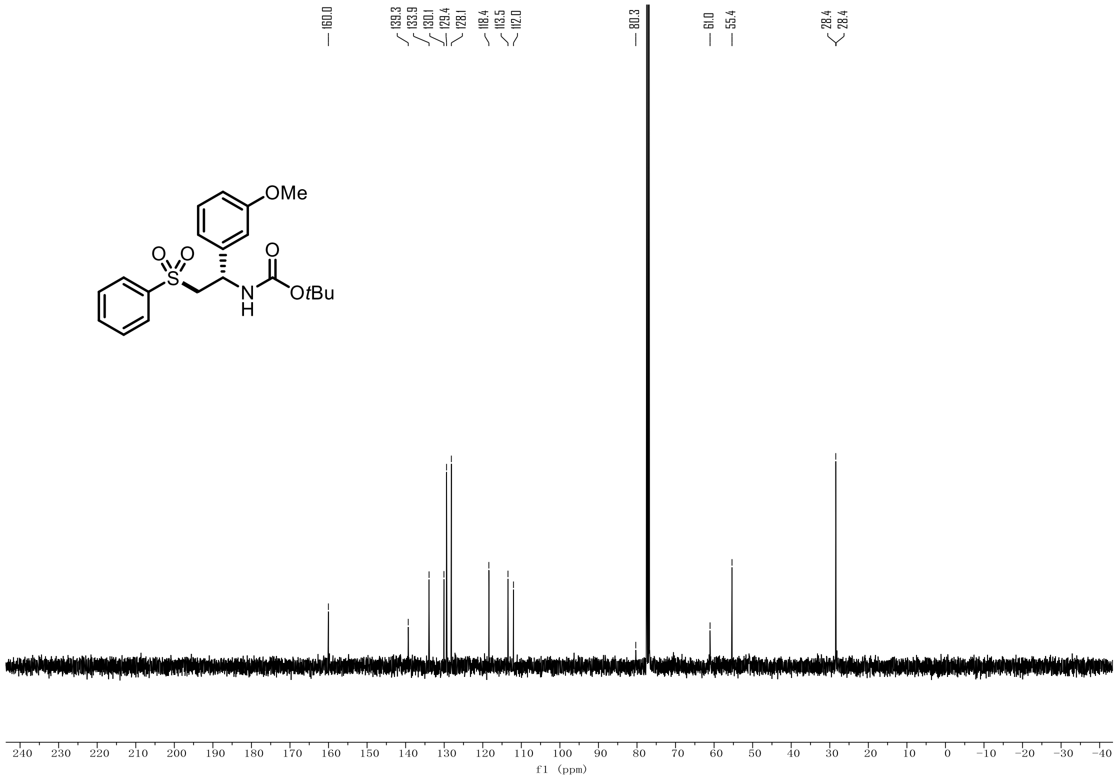

(*S*)-*N*-(1-(4-Cyanophenyl)-2-tosylethyl)-4-methoxybenzamide (46).

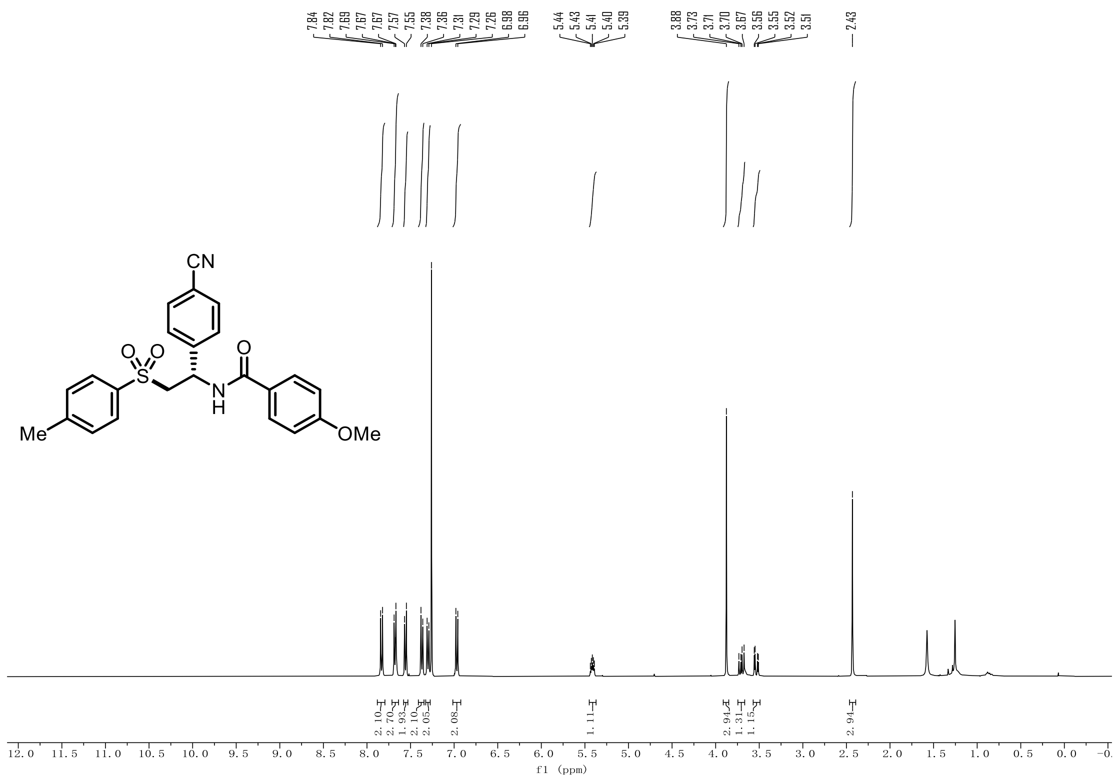

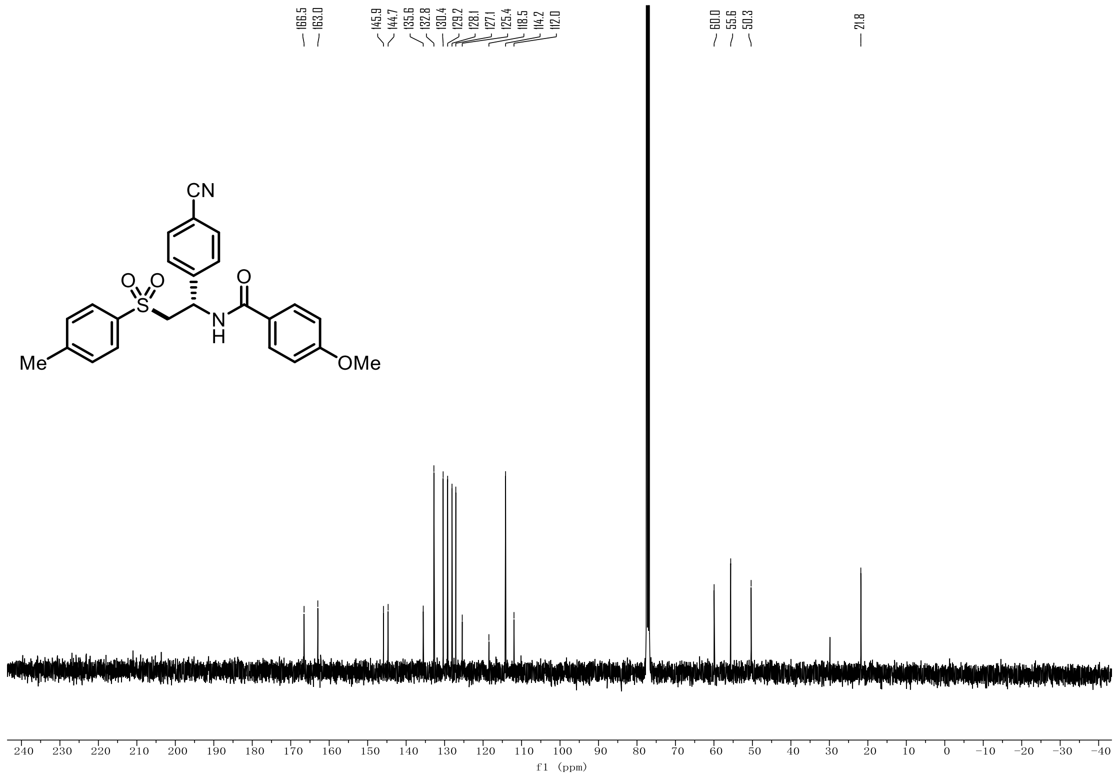

(*S*)-*N*-(2-((4-Chlorophenyl)sulfonyl)-1-(4-cyanophenyl)ethyl)-4-methoxybenzamide (47).

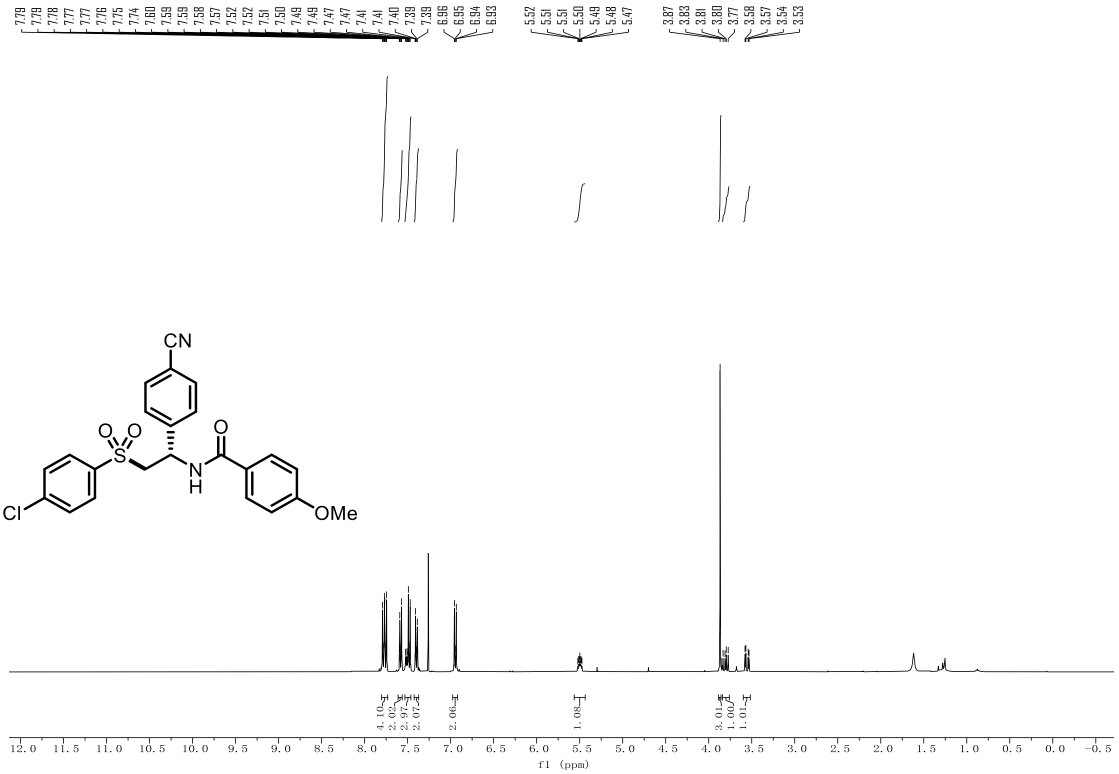

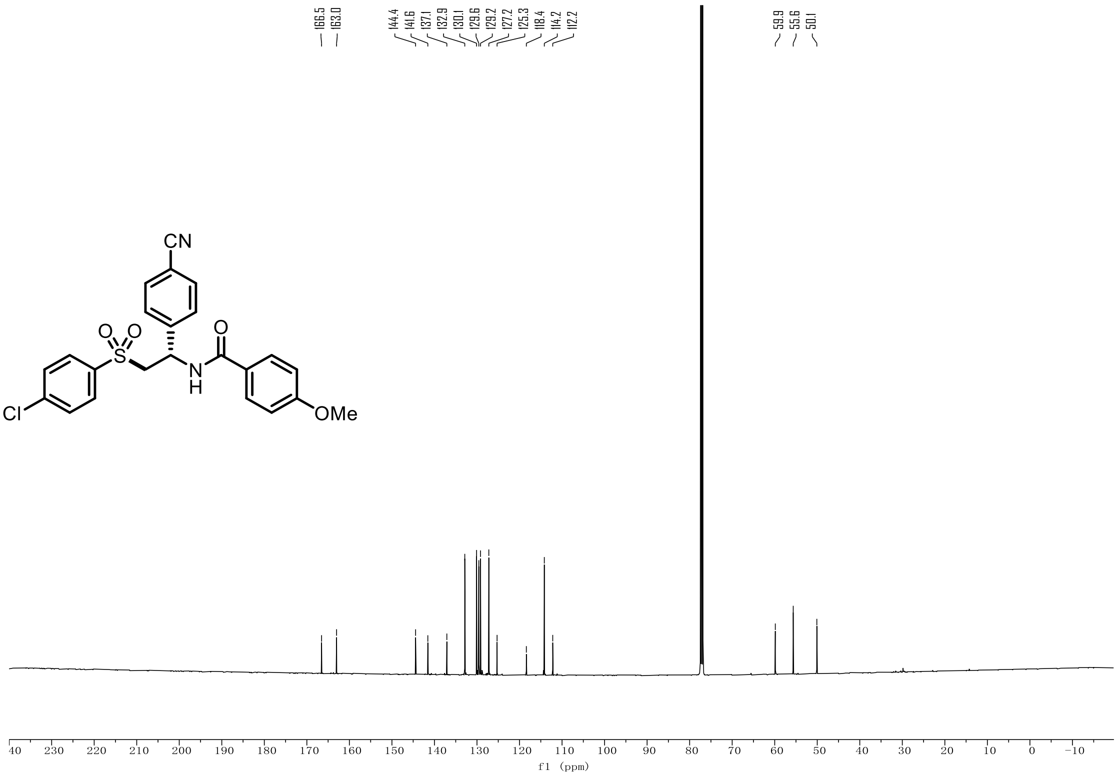

(*S*)-*N*-(2-((4-Acetamidophenyl)sulfonyl)-1-(4-cyanophenyl)ethyl)-4-methoxybenzamide (48).

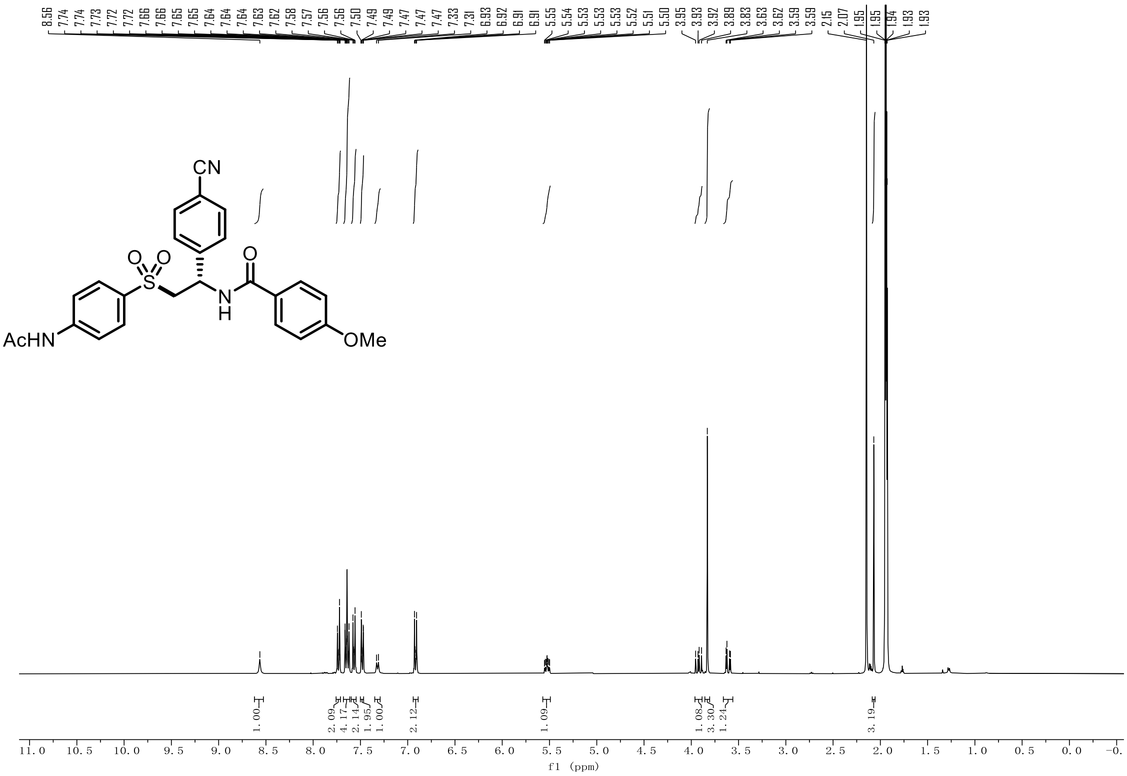

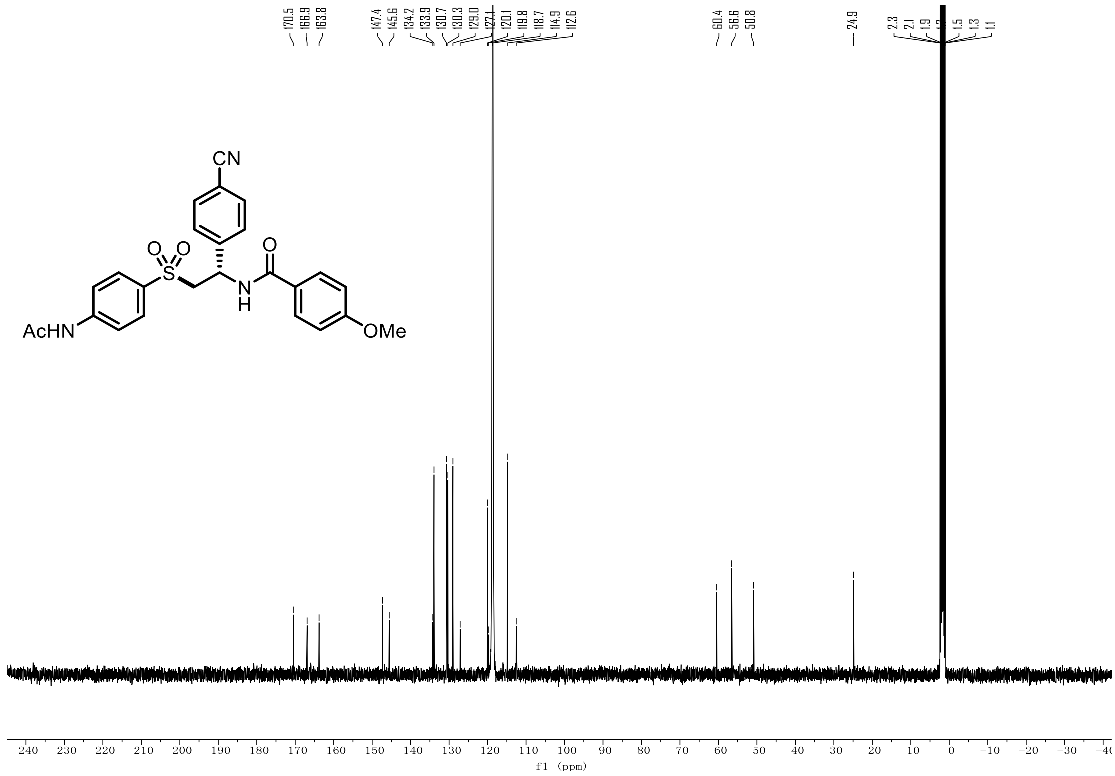

Methyl (*S*)-3-((2-(4-cyanophenyl)-2-(4-methoxybenzamido)ethyl)sulfonyl)propanoate (49)

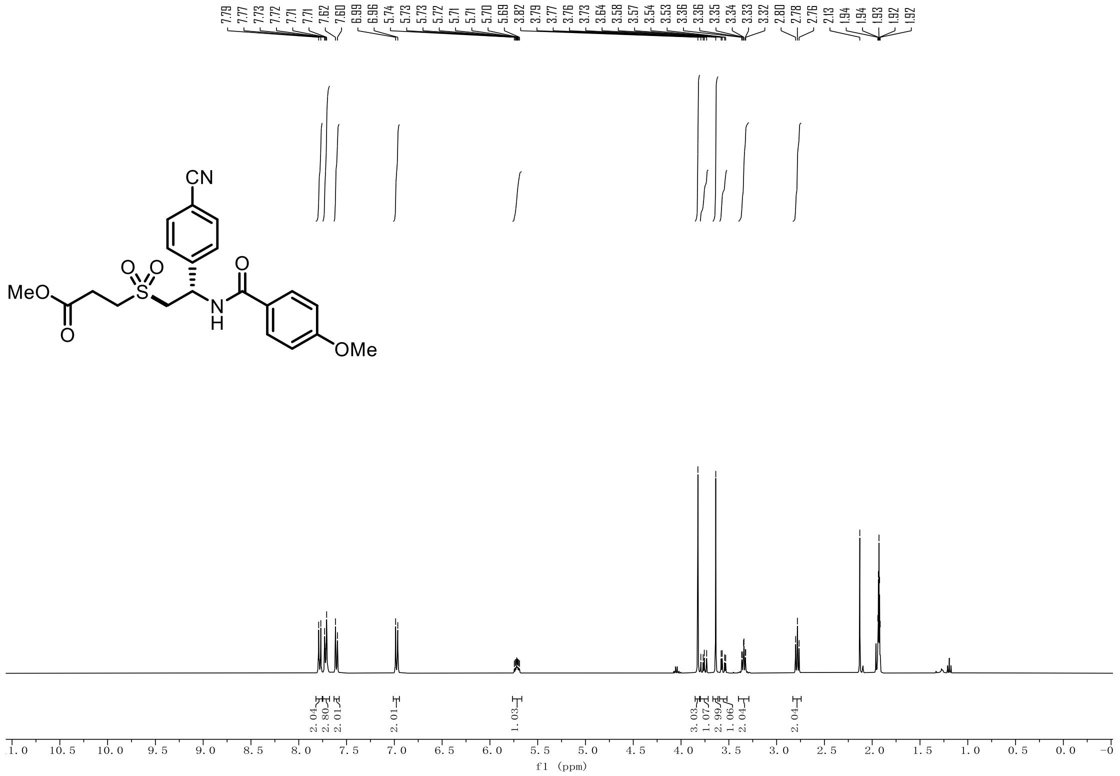

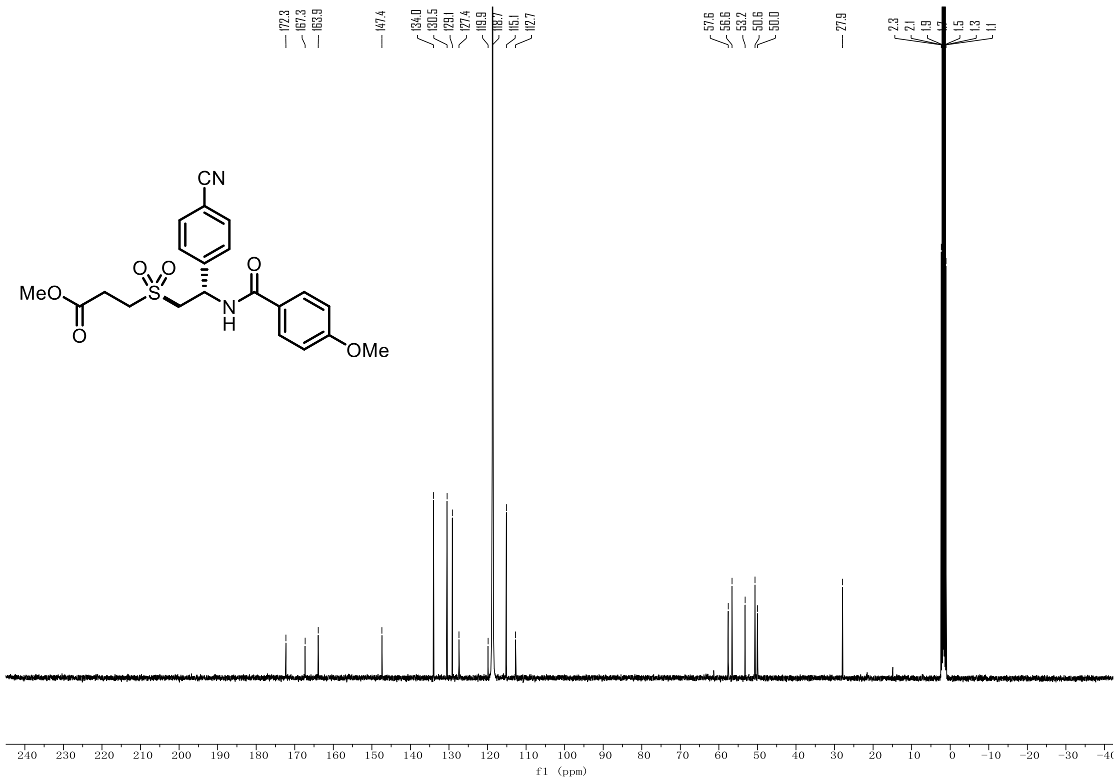

Methyl (*S*)-3-((2-(4-methoxybenzamido)-2-(4-methoxyphenyl)ethyl)sulfonyl)propanoate (50)

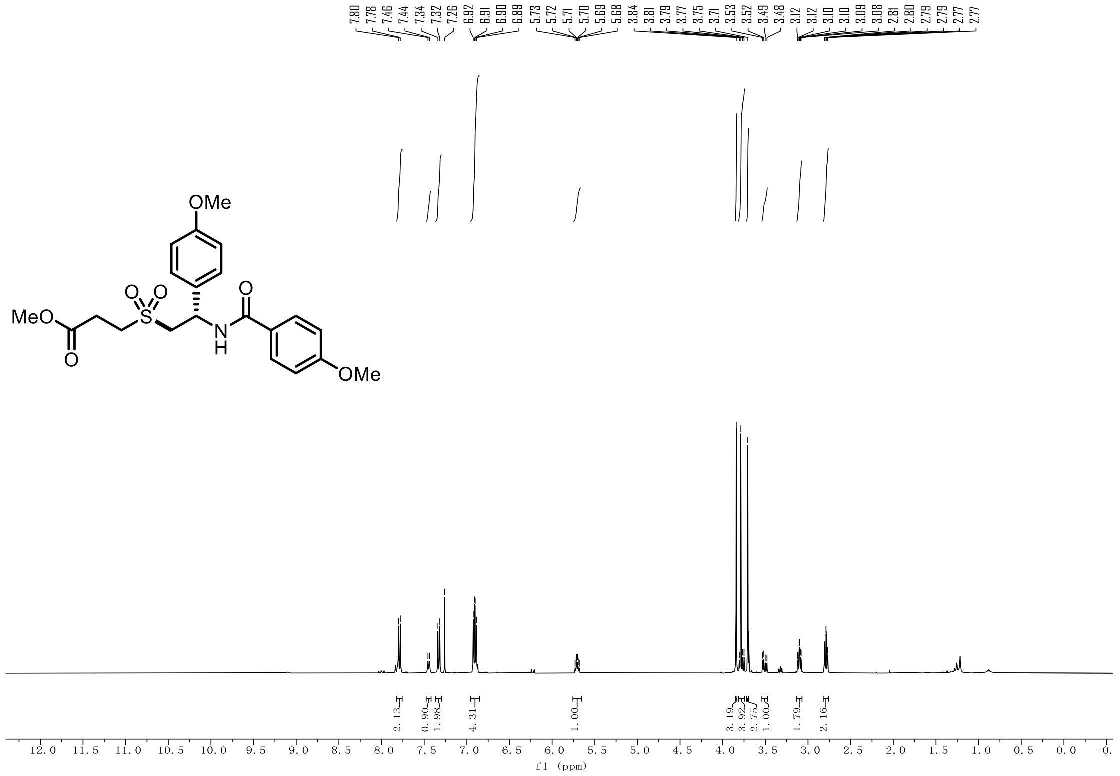

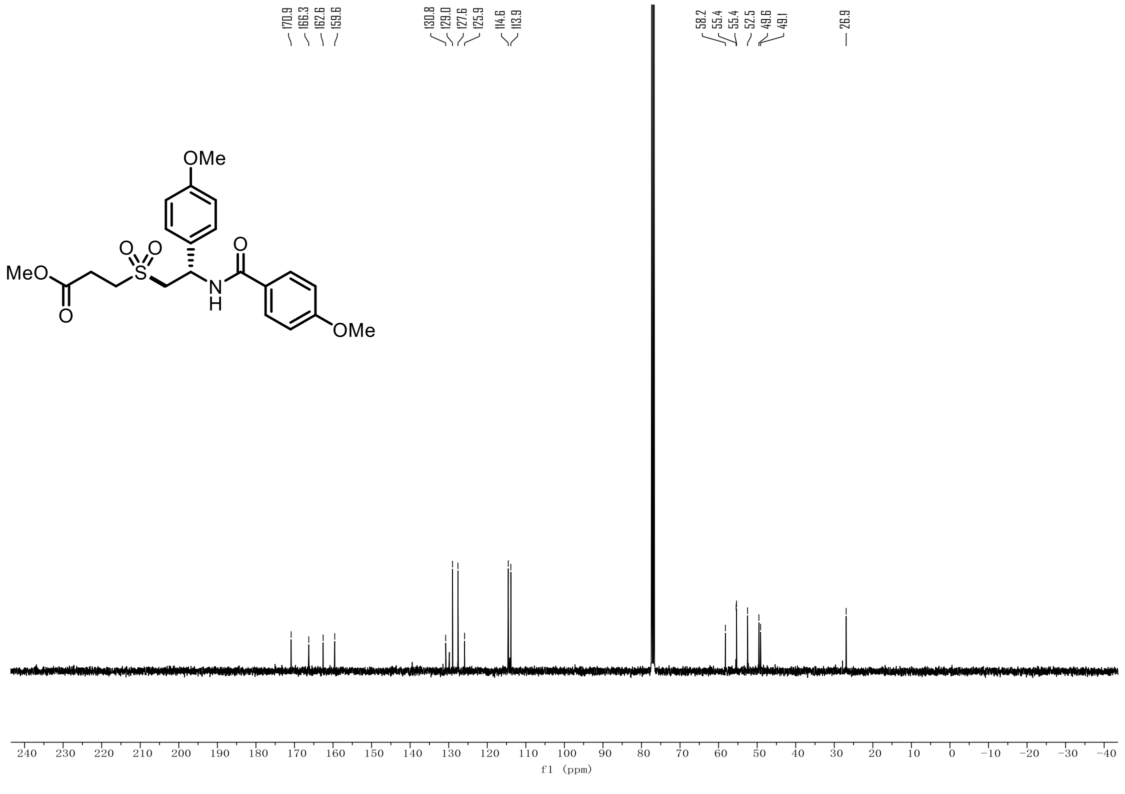

Methyl (*S*)-3-((2-(4-methoxybenzamido)-2-(3-methoxyphenyl)ethyl)sulfonyl)propanoate (51)

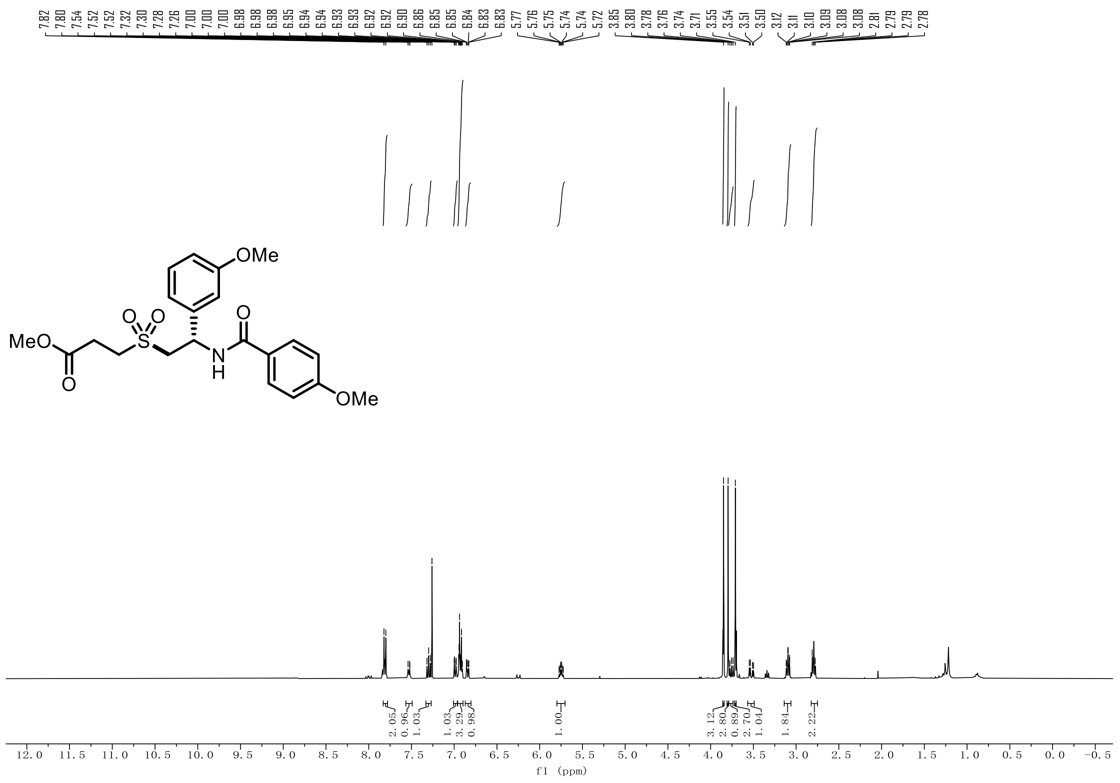

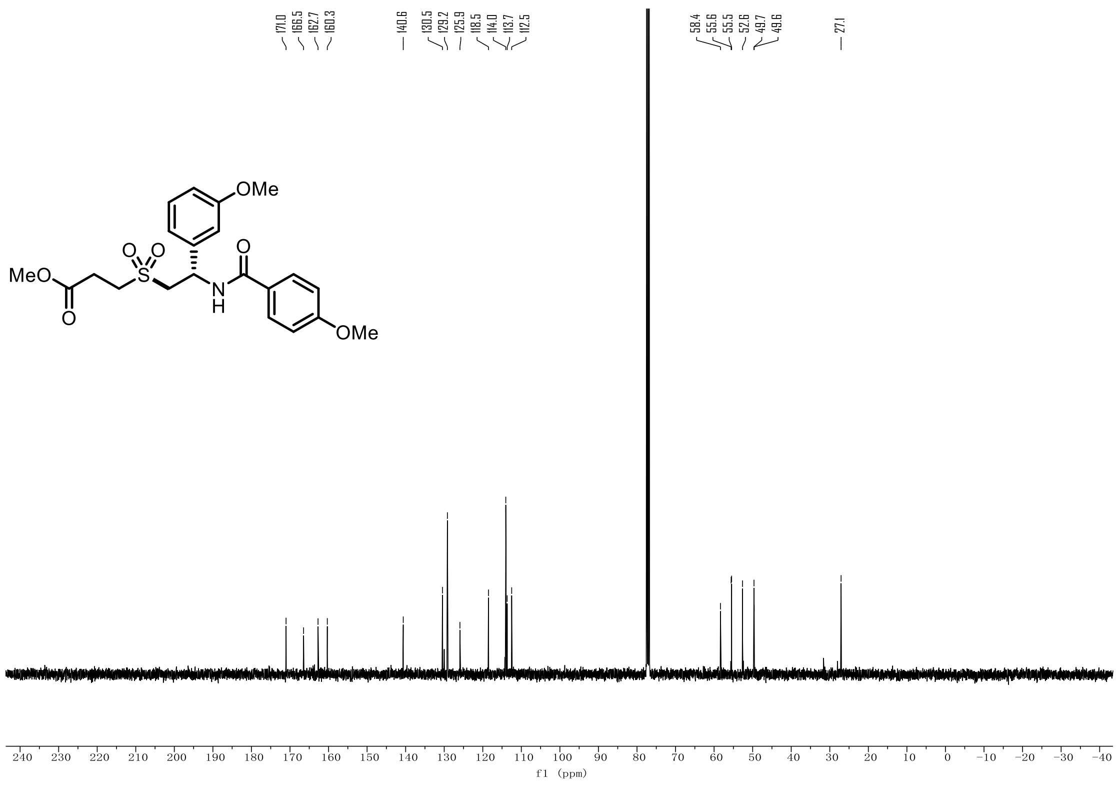

4-Iodophenyl (3-((tert-butoxycarbonyl)amino)propanoyl)glycinate (S22)

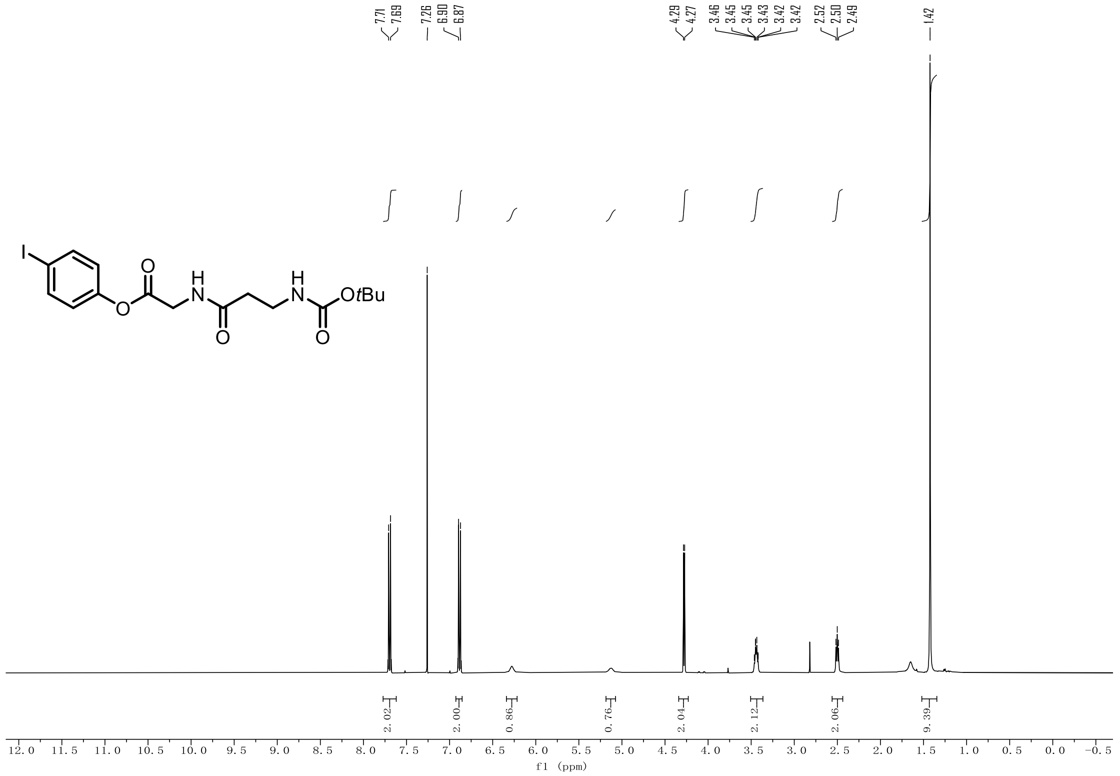

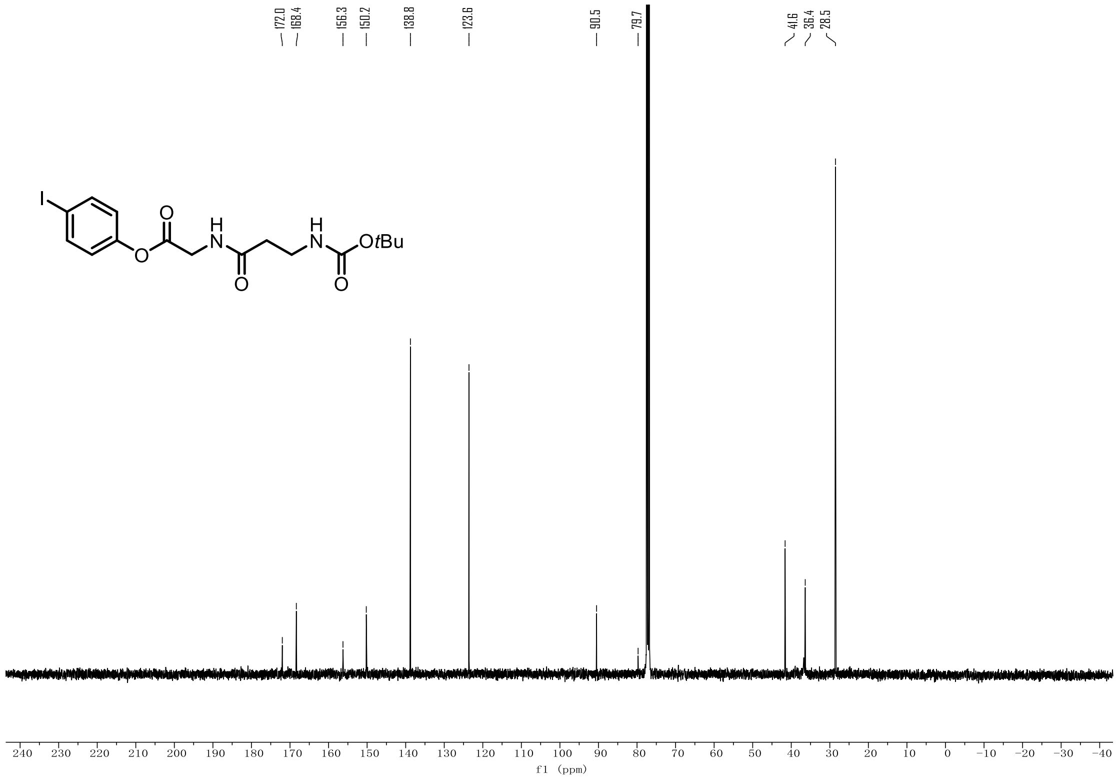

(*S*)-4-(1-Benzamido-2-(phenylsulfonyl)ethyl)phenyl 4-(*N*,*N*-dipropylsulfamoyl)benzoate (52).

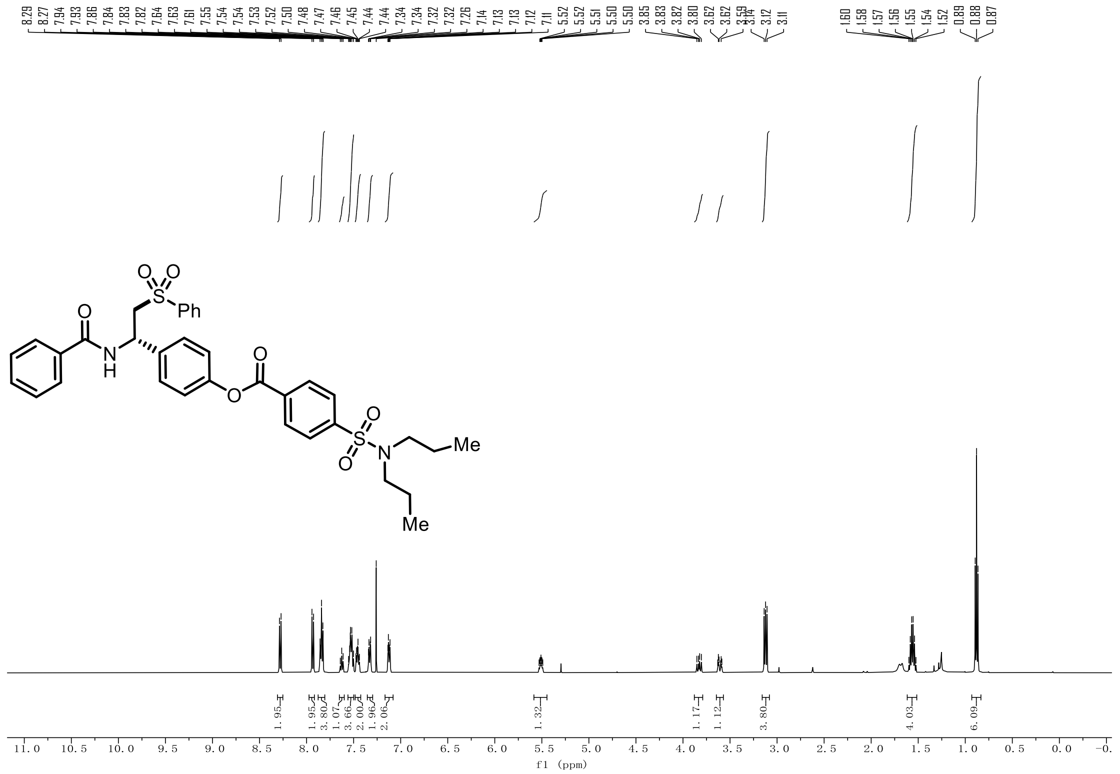

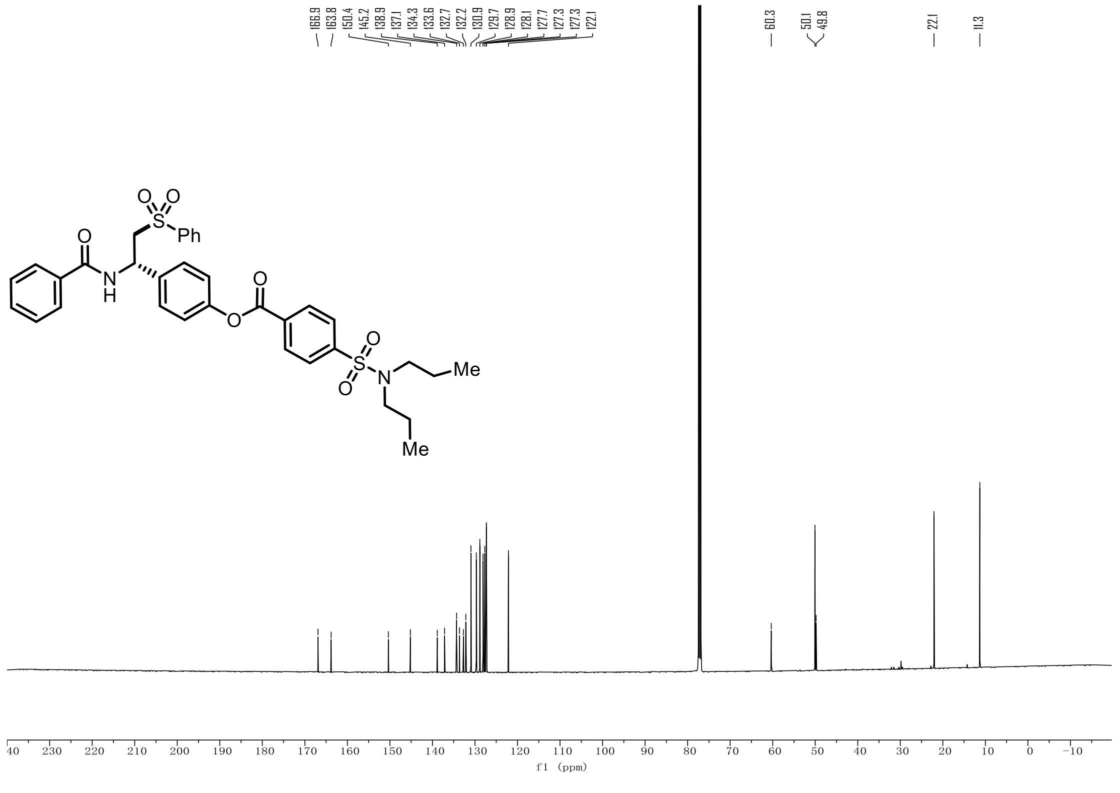

4-((*S*)-1-Benzamido-2-(phenylsulfonyl)ethyl)phenyl (*3S,5S,7S*)-adamantane-1-carboxylate (53).

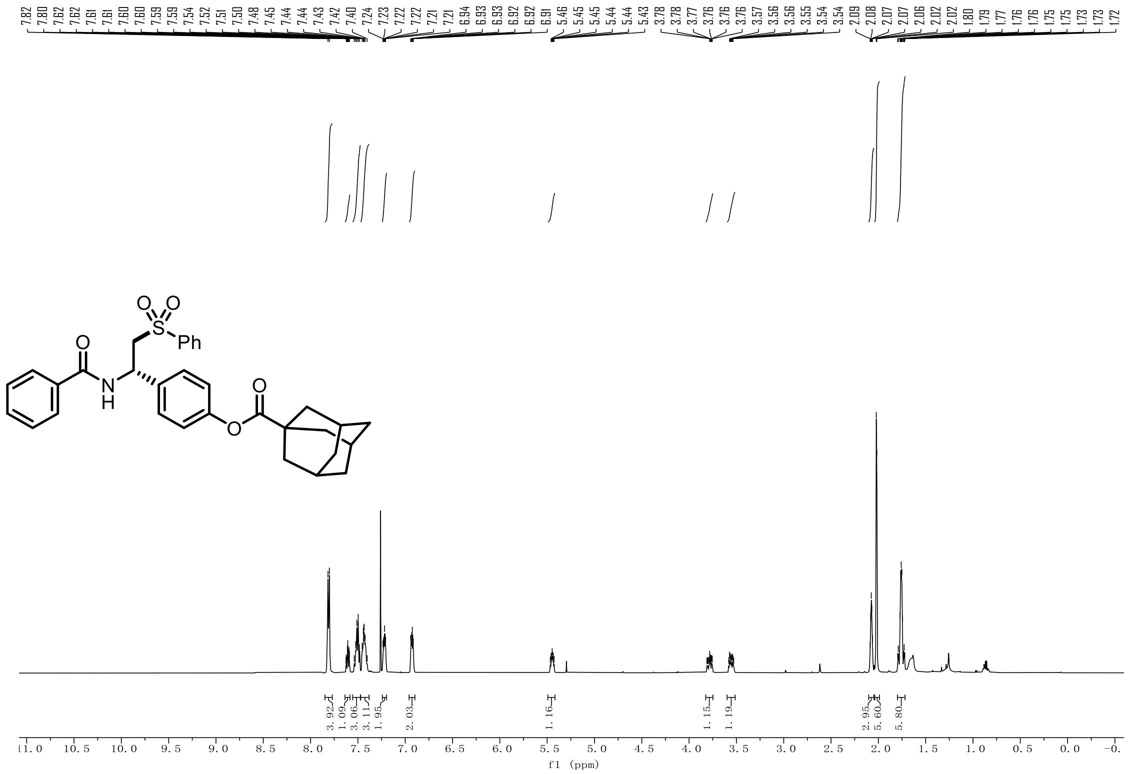

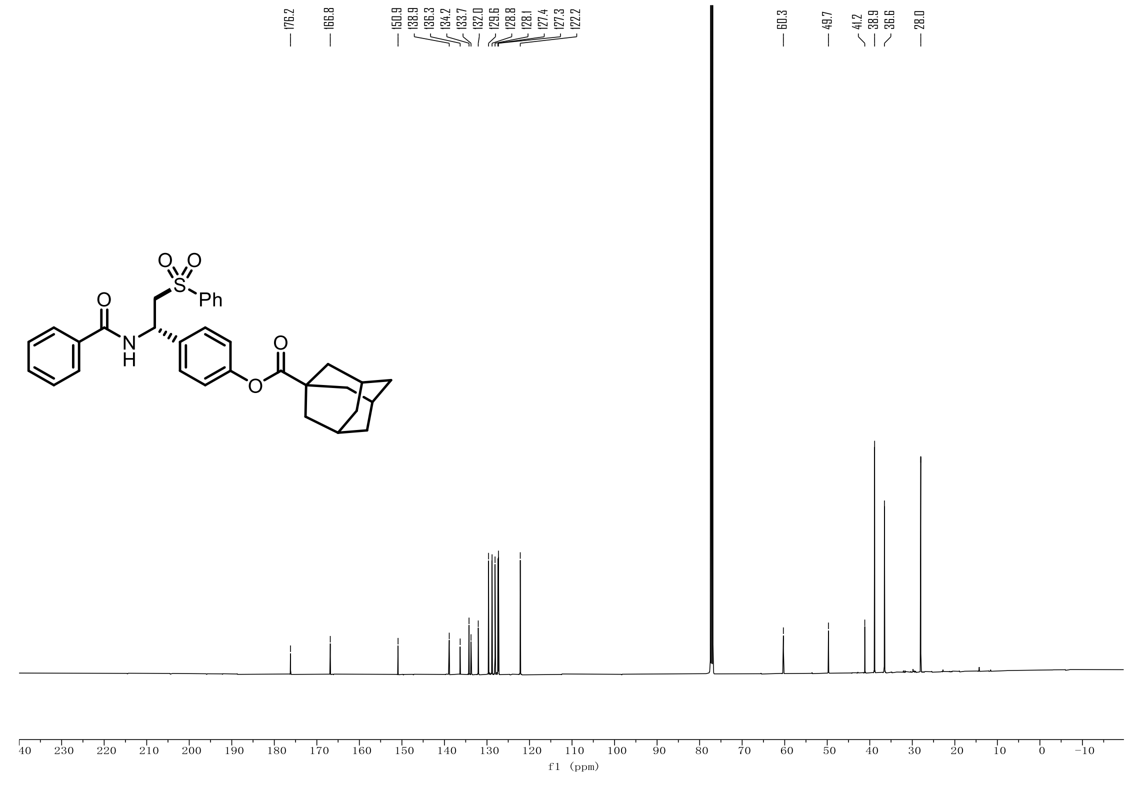

(*3aR,5R,6S,6aR*)-5-((*S*)-2,2-Dimethyl-1,3-dioxolan-4-yl)-2,2-dimethyltetrahydrofuro[2,3-d][1,3]dioxol-6-yl 4-((*S*)-1-benzamido-2-(phenylsulfonyl)ethyl)benzoate (54).

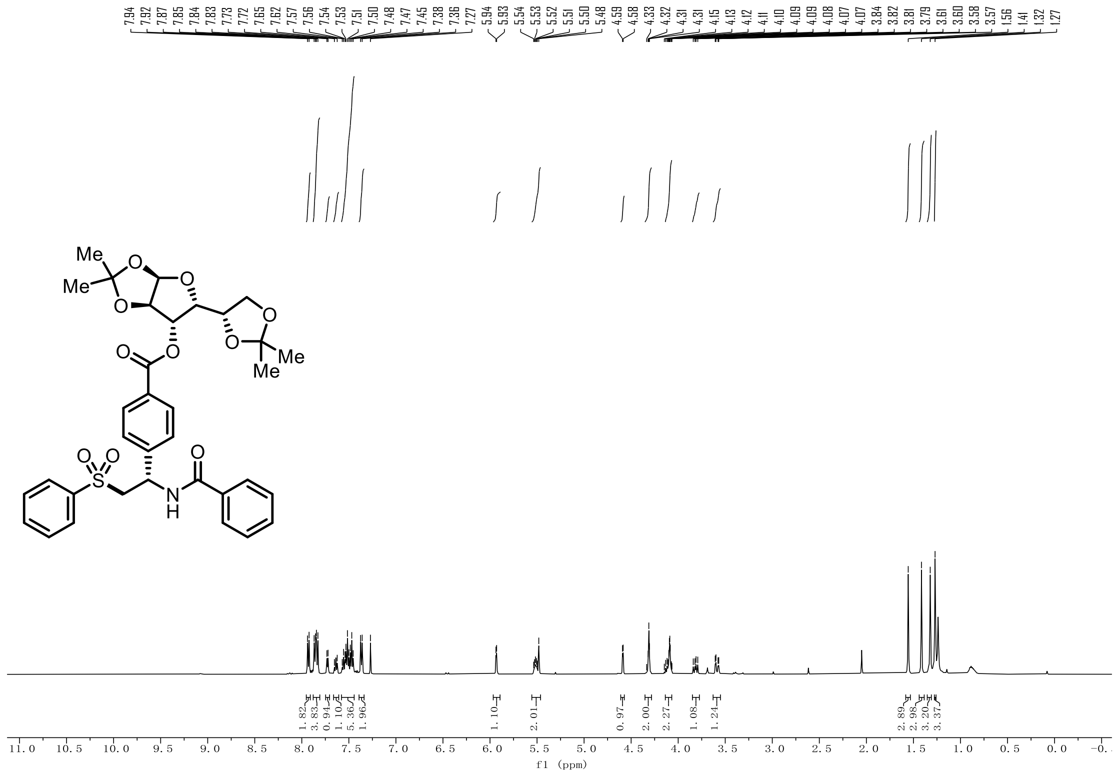

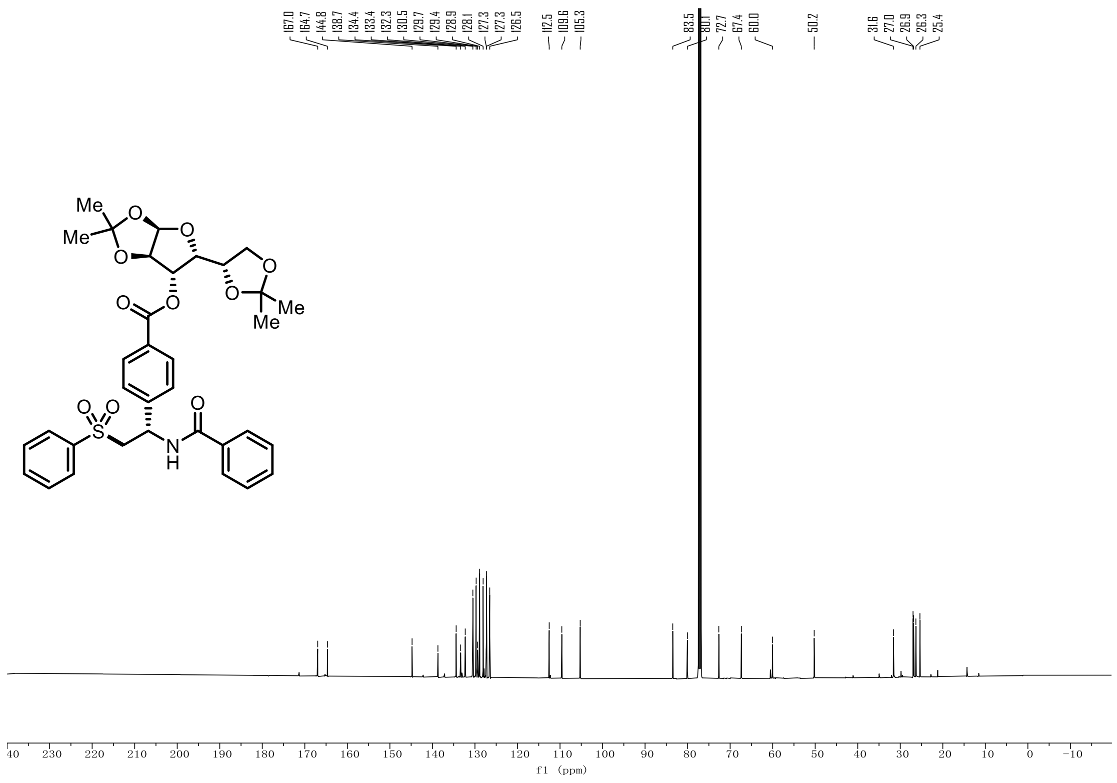

Methyl (*S*)-3-(4-((*S*)-1-benzamido-2-(phenylsulfonyl)ethyl)phenyl)-2-((tert-butoxycarbonyl)amino)propanoate (55).

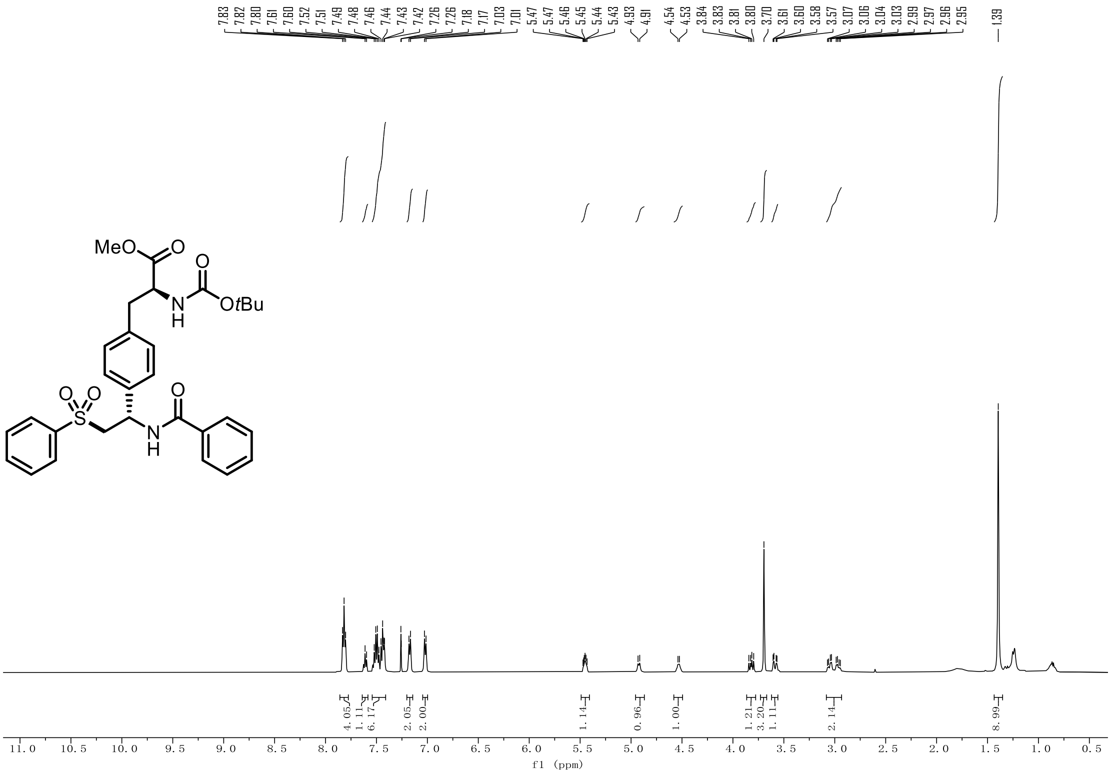

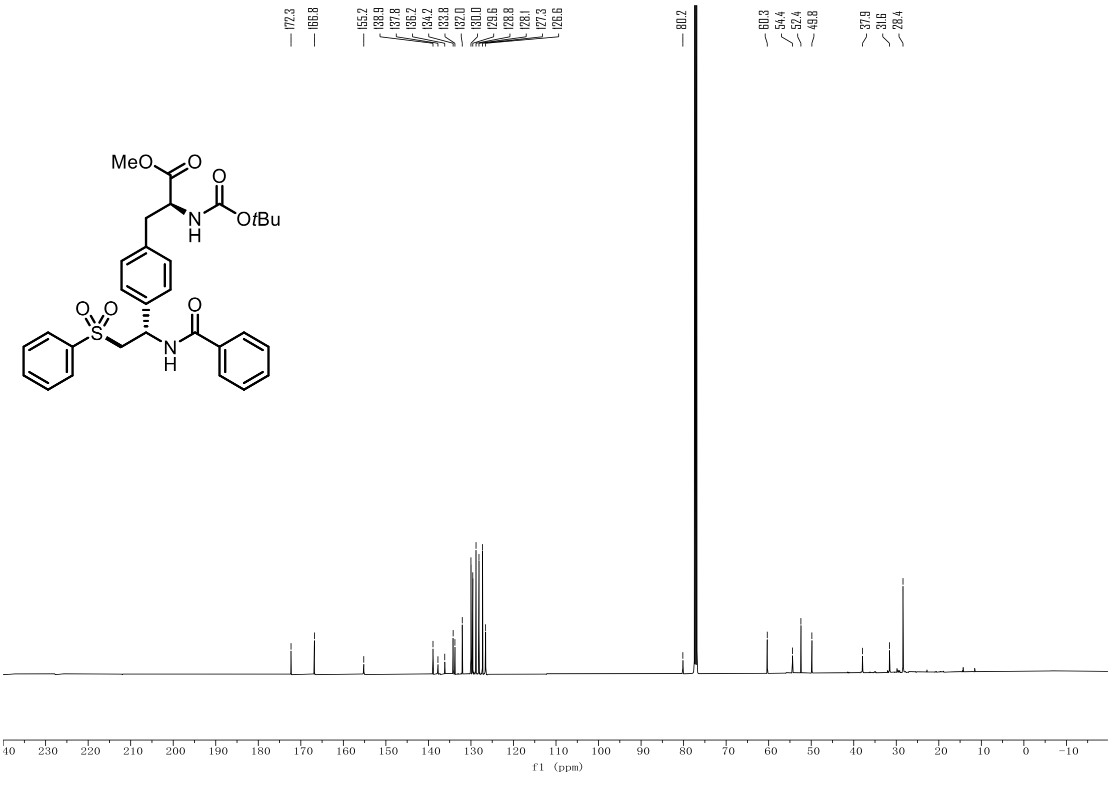

(*S*)-4-(1-Benzamido-2-(phenylsulfonyl)ethyl)phenyl (3-((tert-butoxycarbonyl)amino)propanoyl)glycinate (56)

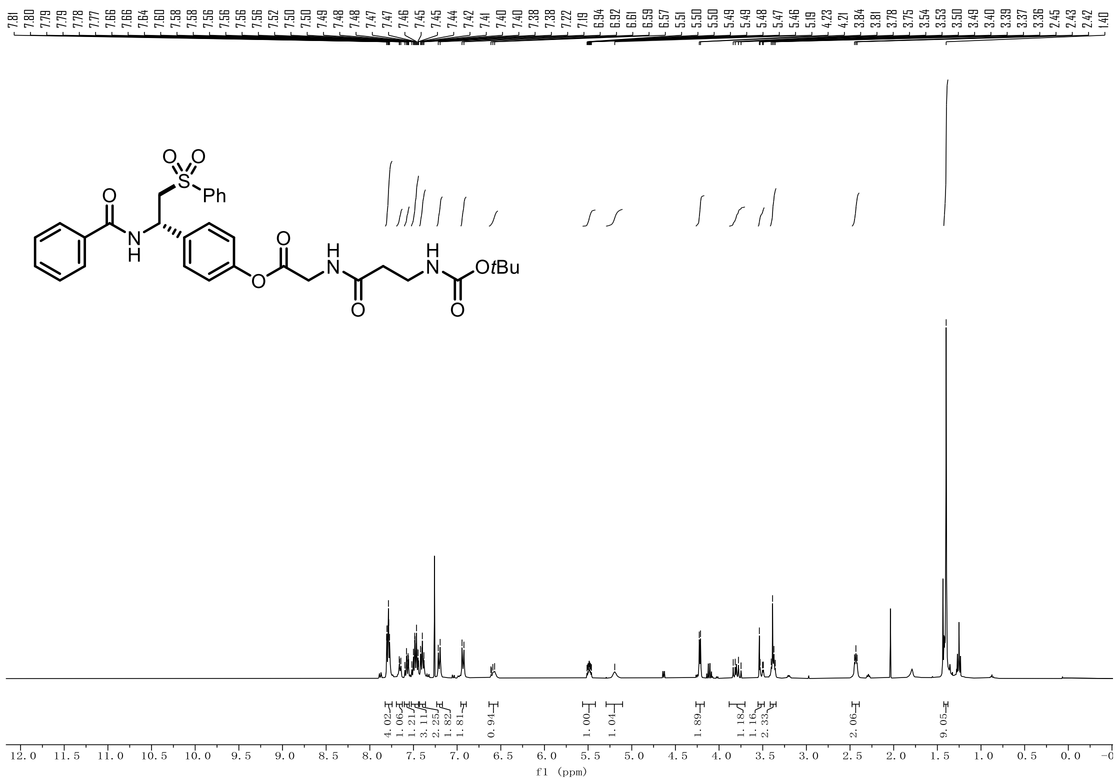

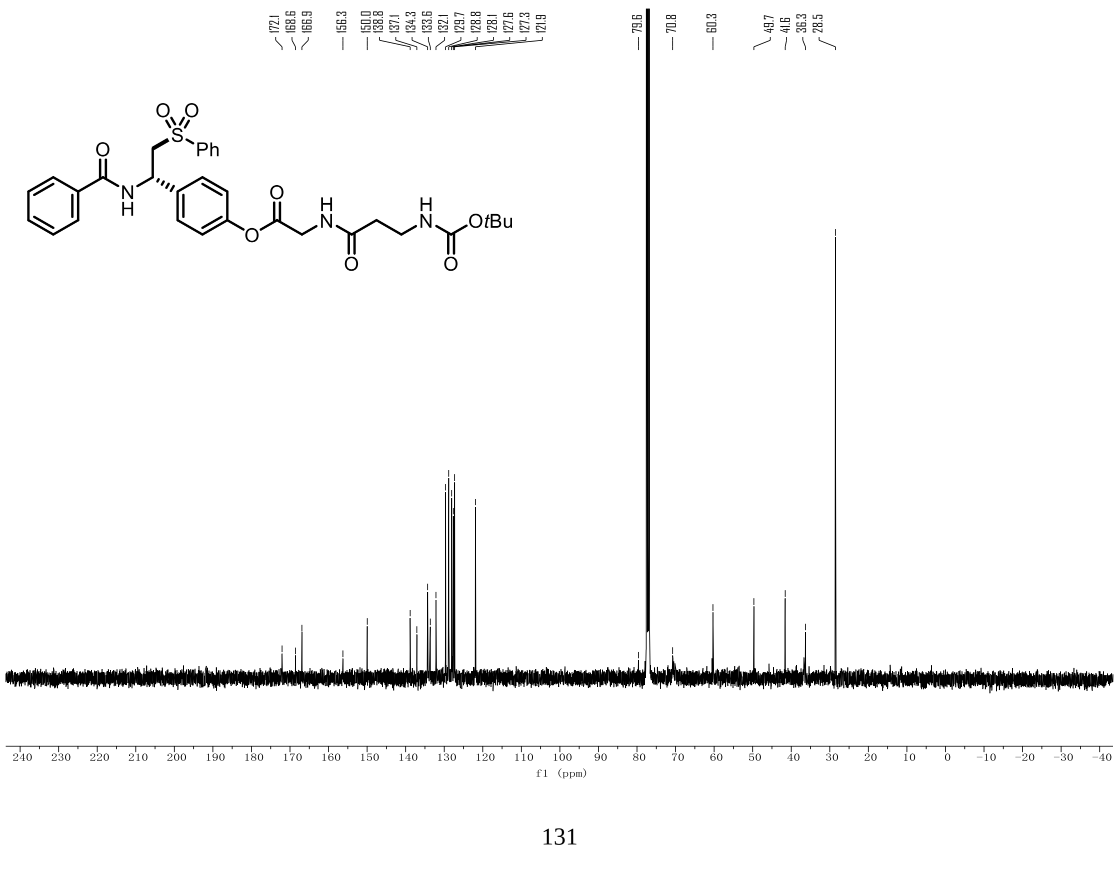

(*3S,5S,8R,9S,10S,13R,17R*)-10,13-Dimethyl-17-((*R*)-6-methylhepta*N*-2-yl)hexadecahydro-1H-cyclopenta[a]phenanthren-3-yl 4-((*S*)-1-benzamido-2-(phenylsulfonyl)ethyl)benzoate (57).

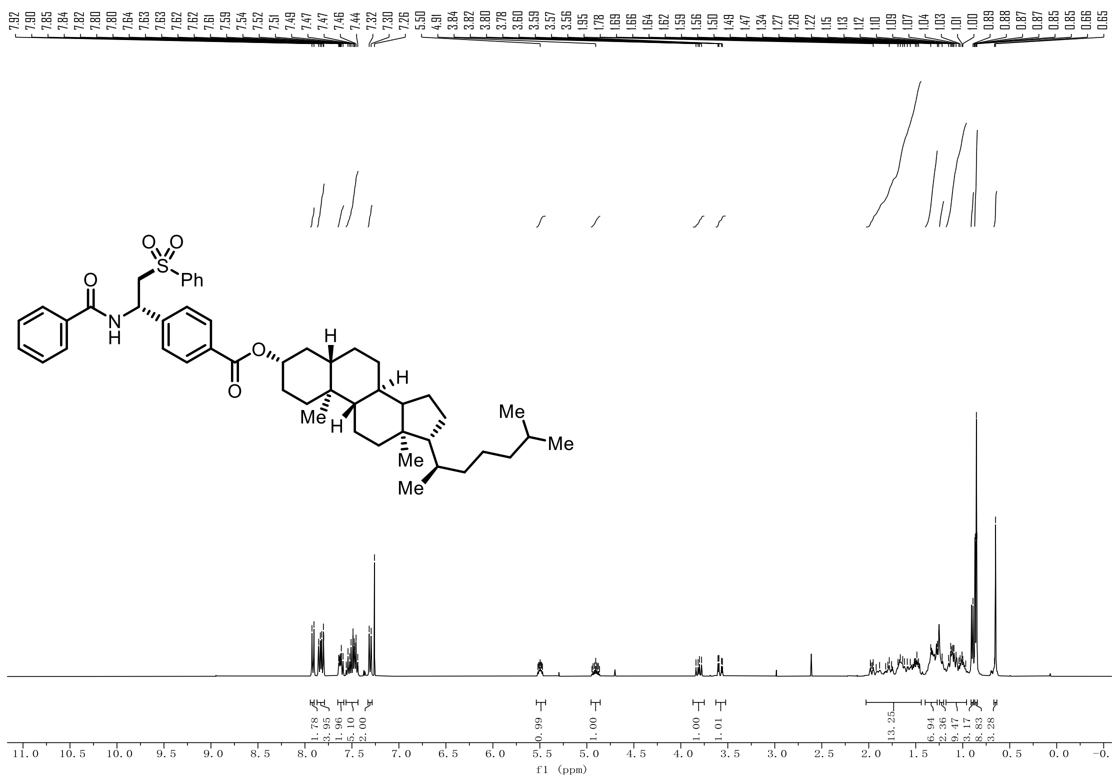

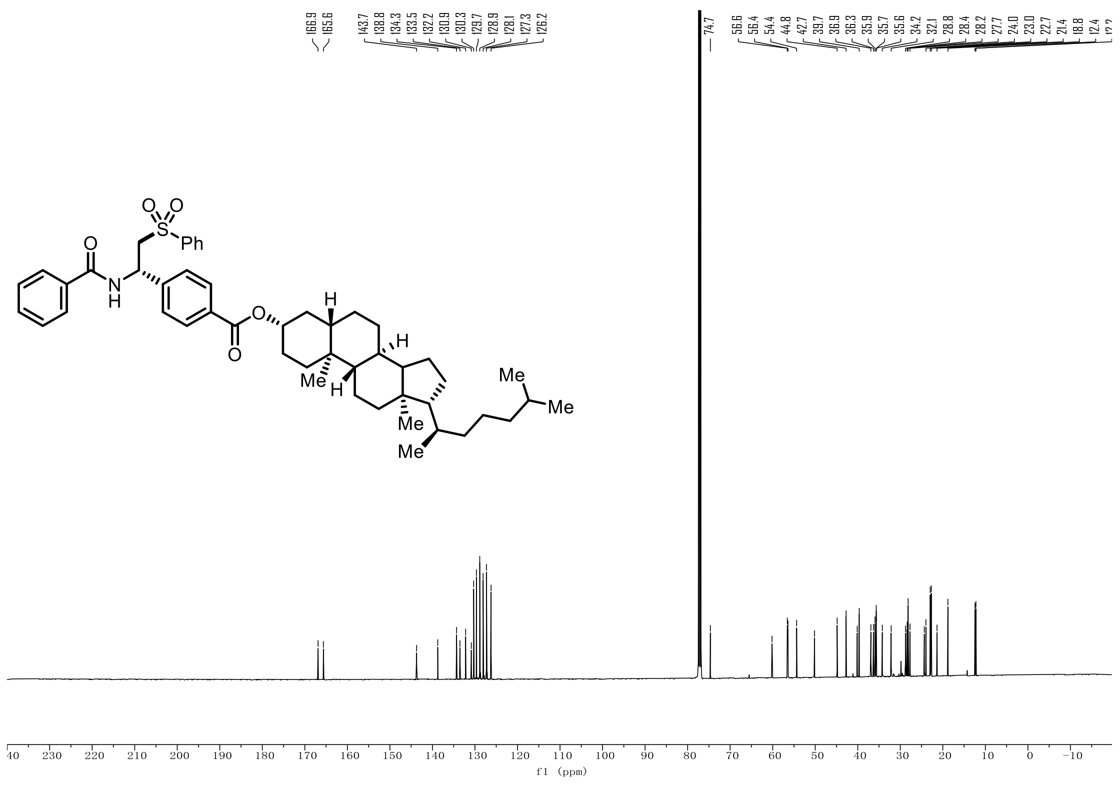

(***R***)**-2,5,7,8-Tetramethyl-2-((*4R,8R*)-4,8,12-trimethyltridecyl)chroman-6-yl 4-((*S*)-1-benzamido-2-(phenylsulfonyl)ethyl)benzoate (58)**.

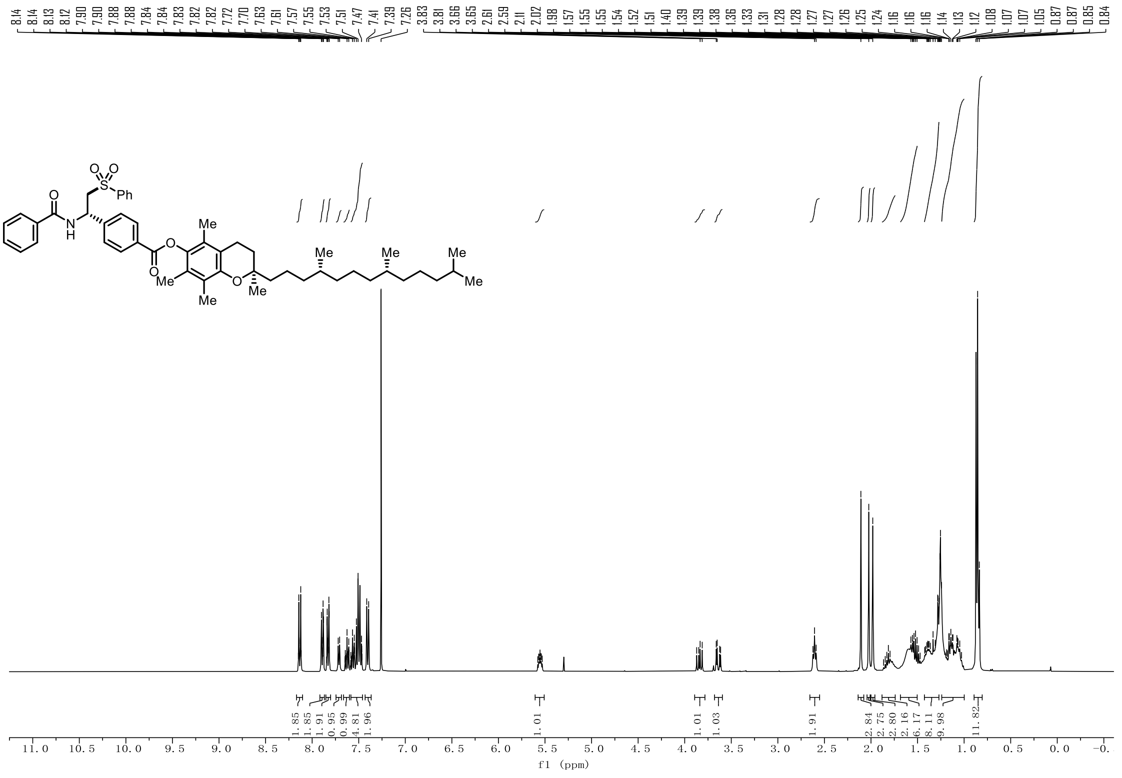

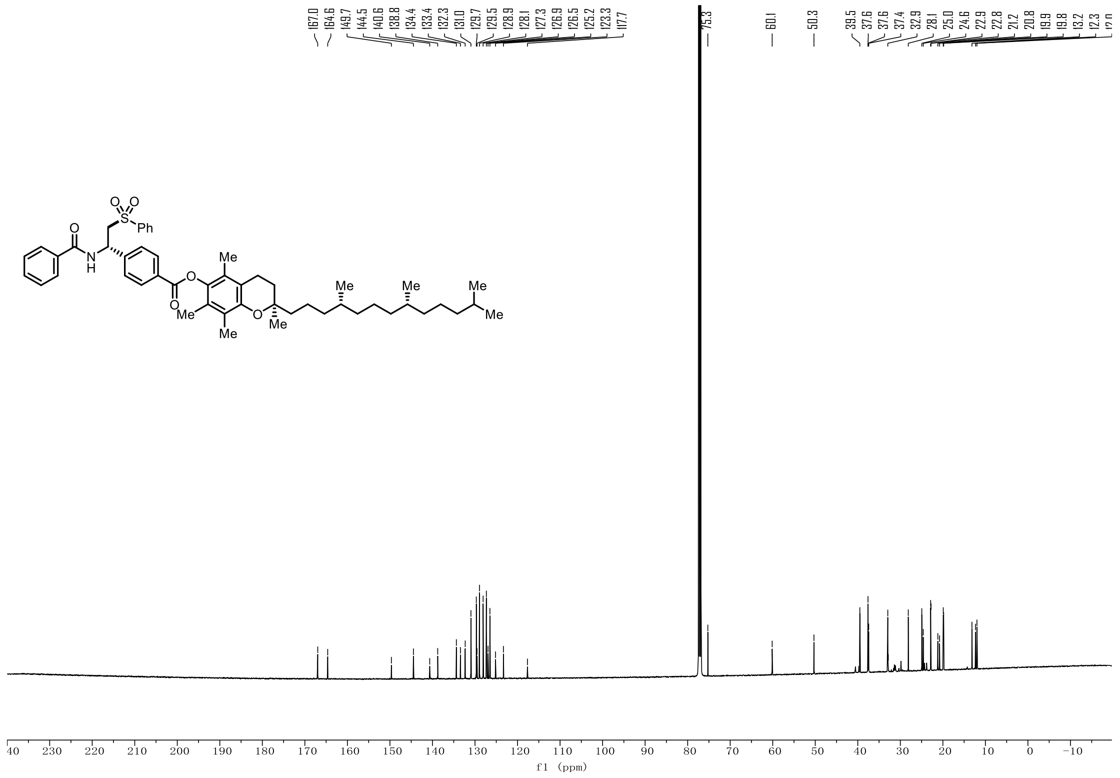

7. HPLC Spectra

(*S*)-*N*-(1-(4-Methoxyphenyl)-2-(phenylsulfonyl)ethyl)benzamide (1).

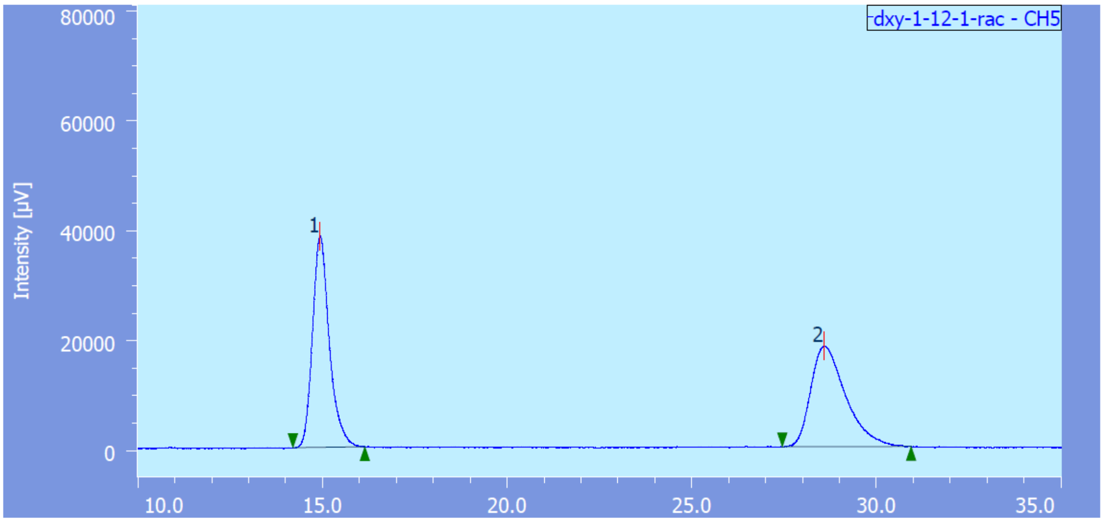

#	Peak Name	CH	tR [min]	Area [µV·sec]	Height [µV]	Area%	Height%	Quantity	NTP	Resolution	Symmetry Factor	Warning
1	Unknown	5	14.937	1217010	38431	50.163	67.703	N/A	5673	11.056	1.278	
2	Unknown	5	28.580	1209115	18333	49.837	32.297	N/A	4623	N/A	1.529	

HPLC spectrum of rac-**1**

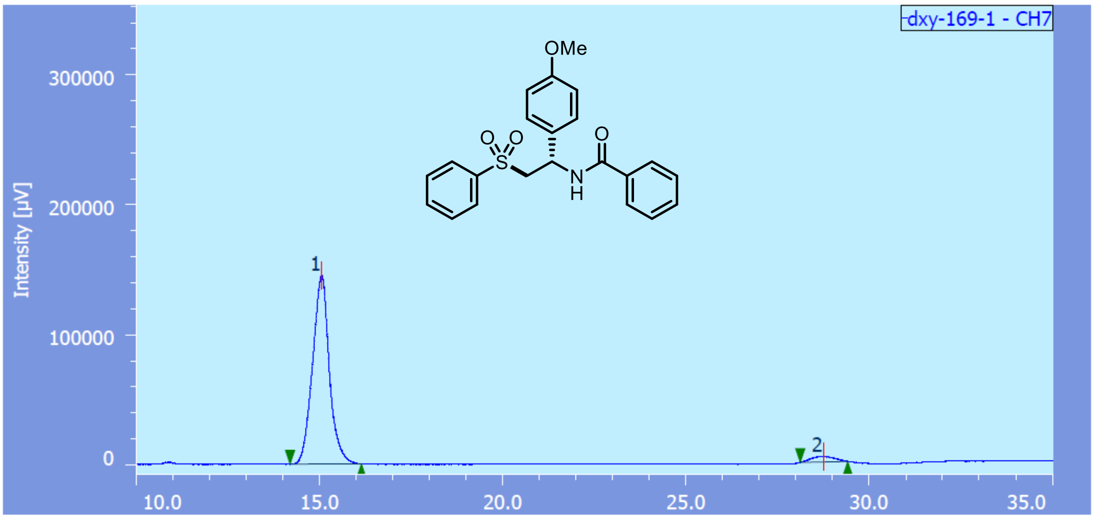

#	Peak Name	CH	tR [min]	Area [µV·sec]	Height [µV]	Area%	Height%	Quantity	NTP	Resolution	Symmetry Factor	Warning
1	Unknown	7	15.053	4615347	145038	95.972	97.102	N/A	5686	13.257	1.045	
2	Unknown	7	28.743	193722	4328	4.028	2.898	N/A	8167	N/A	1.026	

HPLC spectrum of **1**

(*S*)-*N*-(2-(Phenylsulfonyl)-1-(*p*-tolyl)ethyl)benzamide (2).

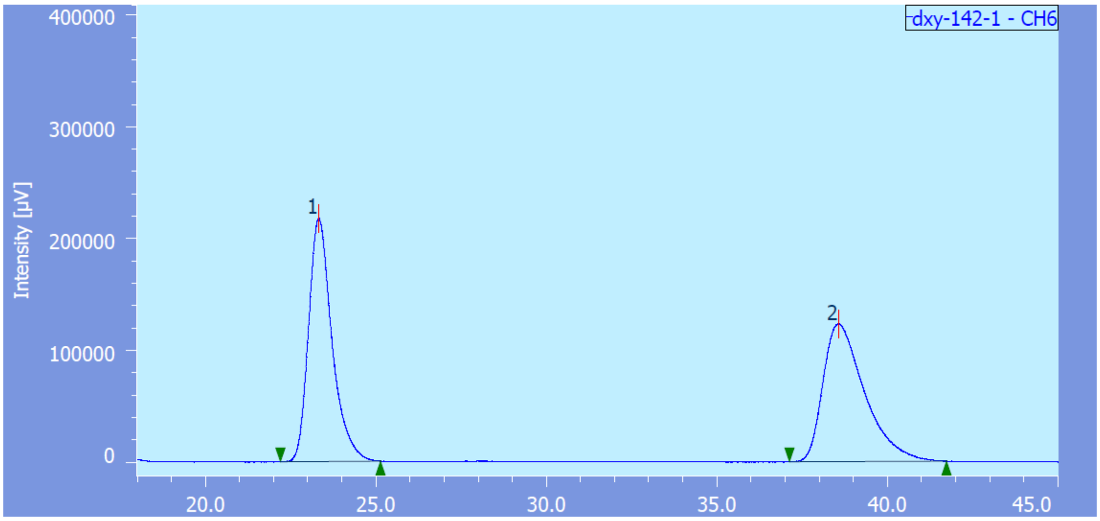

#	Peak Name	CH	tR [min]	Area [µV·sec]	Height [µV]	Area%	Height%	Quantity	NTP	Resolution	Symmetry Factor	Warning
1	Unknown	6	23.317	10387586	217265	49.871	63.838	N/A	6033	9.082	1.362	
2	Unknown	6	38.553	10441218	123075	50.129	36.162	N/A	5081	N/A	1.635	

HPLC spectrum of rac-**2**

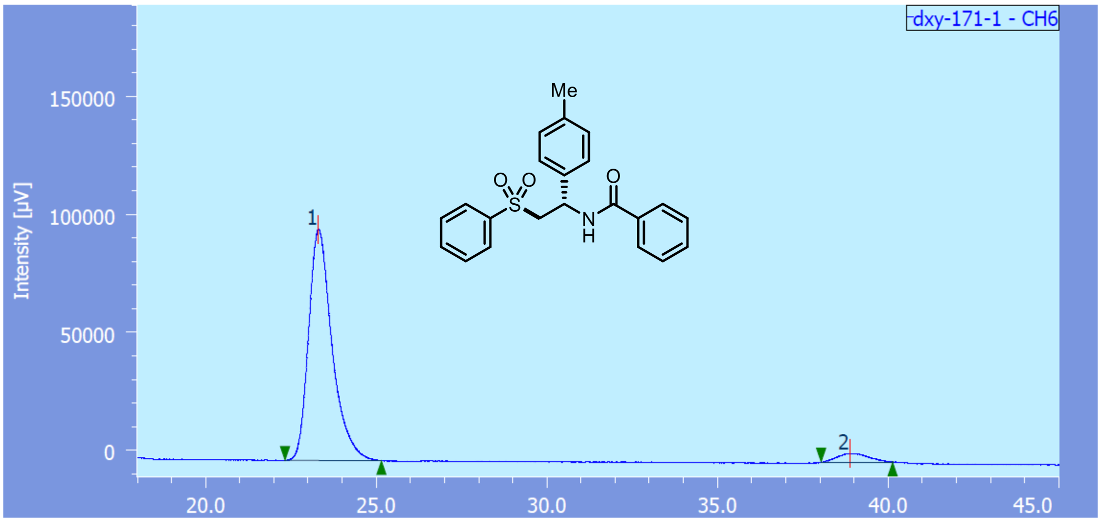

#	Peak Name	CH	tR [min]	Area [µV·sec]	Height [µV]	Area%	Height%	Quantity	NTP	Resolution	Symmetry Factor	Warning
1	Unknown	6	23.303	4680013	97878	95.231	96.404	N/A	6037	10.506	1.395	
2	Unknown	6	38.870	234351	3651	4.769	3.596	N/A	7703	N/A	1.265	

HPLC spectrum of **2**

(*S*)-*N*-(1-(4-(Tert-butyl)phenyl)-2-(phenylsulfonyl)ethyl)benzamide (3).

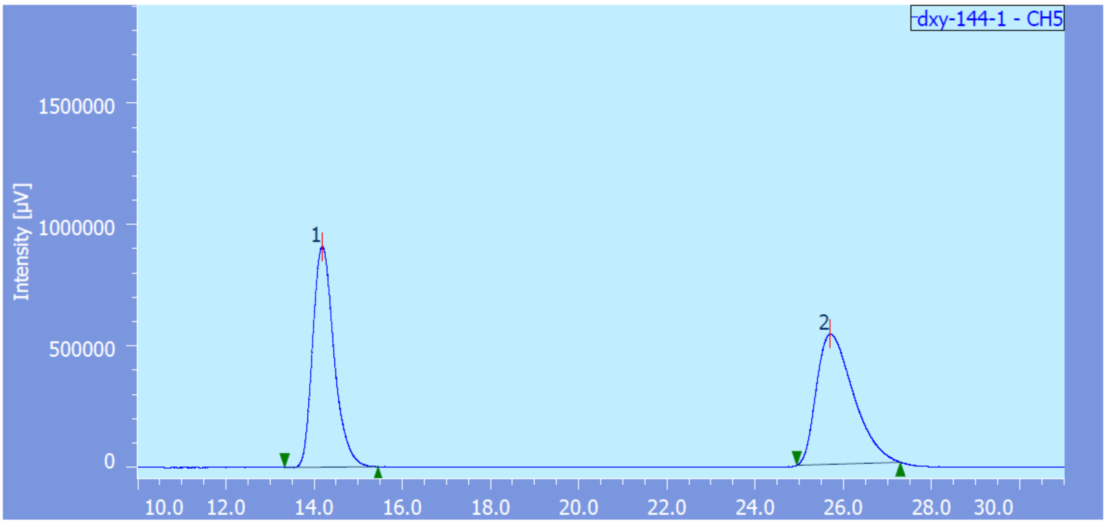

#	Peak Name	CH	tR [min]	Area [µV·sec]	Height [µV]	Area%	Height%	Quantity	NTP	Resolution	Symmetry Factor	Warning
1	Unknown	5	14.183	30592332	909084	49.341	62.868	N/A	4300	9.570	1.295	
2	Unknown	5	25.697	31409337	536943	50.659	37.132	N/A	4412	N/A	1.554	

HPLC spectrum of rac-**3**

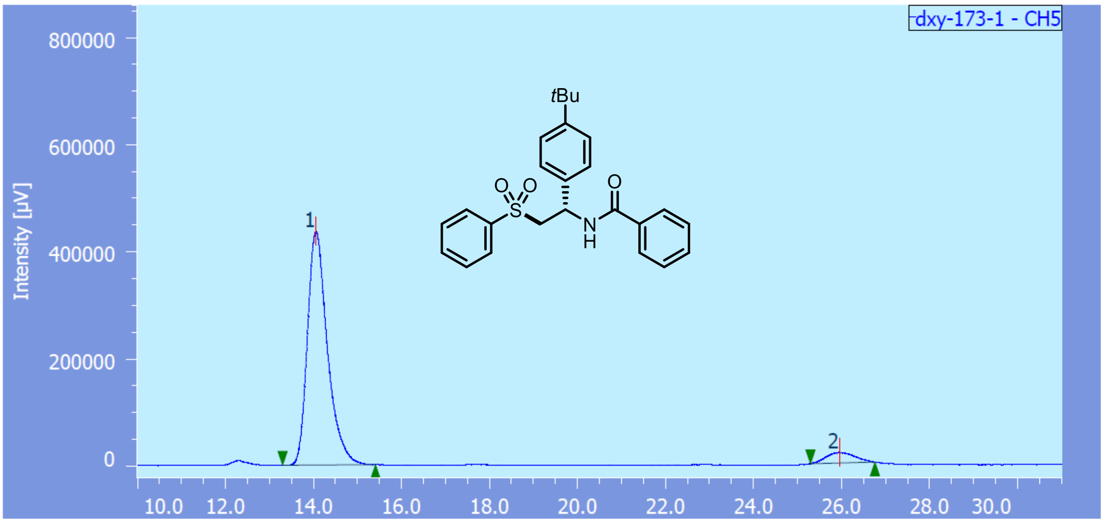

#	Peak Name	CH	tR [min]	Area [µV·sec]	Height [µV]	Area%	Height%	Quantity	NTP	Resolution	Symmetry Factor	Warning
1	Unknown	5	14.057	13767447	436544	93.865	95.650	N/A	5017	11.632	1.376	
2	Unknown	5	25.947	899881	19851	6.135	4.350	N/A	6828	N/A	1.115	

HPLC spectrum of **3**

(*S*)-*N*-(1-([1,1'-Biphenyl]-4-yl)-2-(phenylsulfonyl)ethyl)benzamide (4).

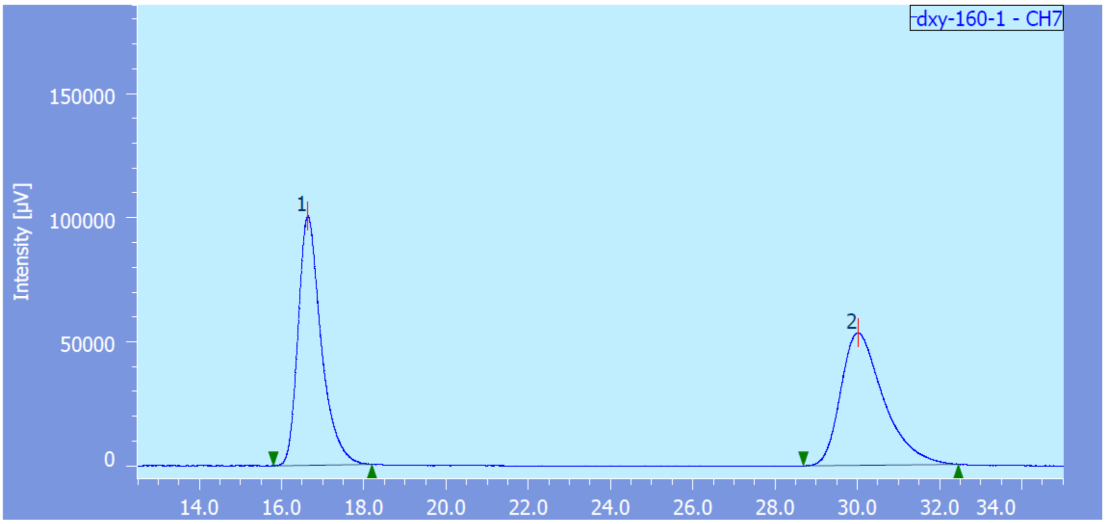

#	Peak Name	CH	tR [min]	Area [µV·sec]	Height [µV]	Area%	Height%	Quantity	NTP	Resolution	Symmetry Factor	Warning
1	Unknown	7	16.637	3907085	100411	50.240	65.262	N/A	4667	9.543	1.401	
2	Unknown	7	30.003	3869746	53446	49.760	34.738	N/A	4279	N/A	1.477	

HPLC spectrum of rac-**4**

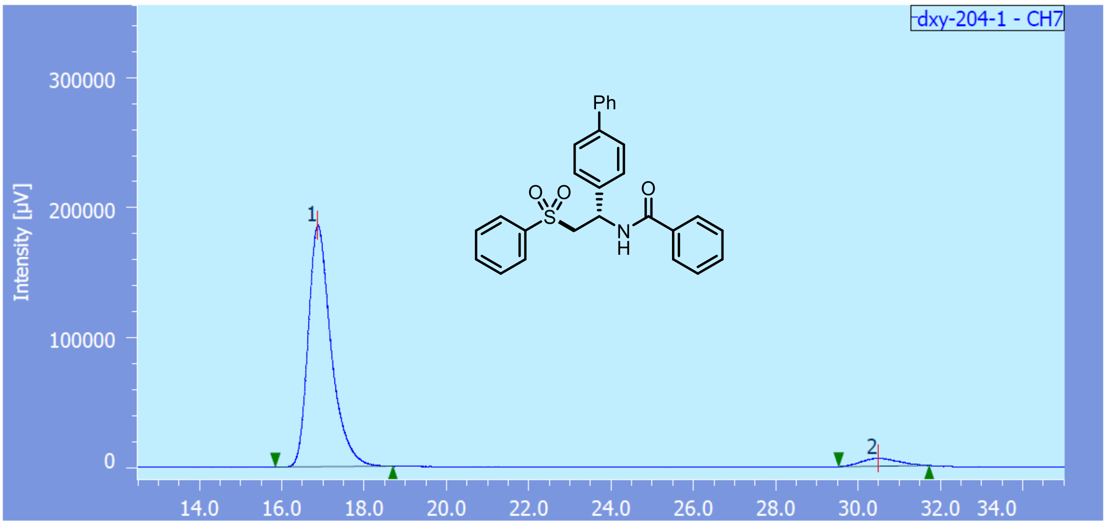

#	Peak Name	CH	tR [min]	Area [µV·sec]	Height [µV]	Area%	Height%	Quantity	NTP	Resolution	Symmetry Factor	Warning
1	Unknown	7	16.877	7195195	185811	94.997	96.886	N/A	4869	10.148	1.395	
2	Unknown	7	30.477	378902	5972	5.003	3.114	N/A	5024	N/A	1.184	

HPLC spectrum of **4**

(*S*)-*N*-(1-(4-(Tert-butyl)phenyl)-2-(phenylsulfonyl)ethyl)benzamide (5).

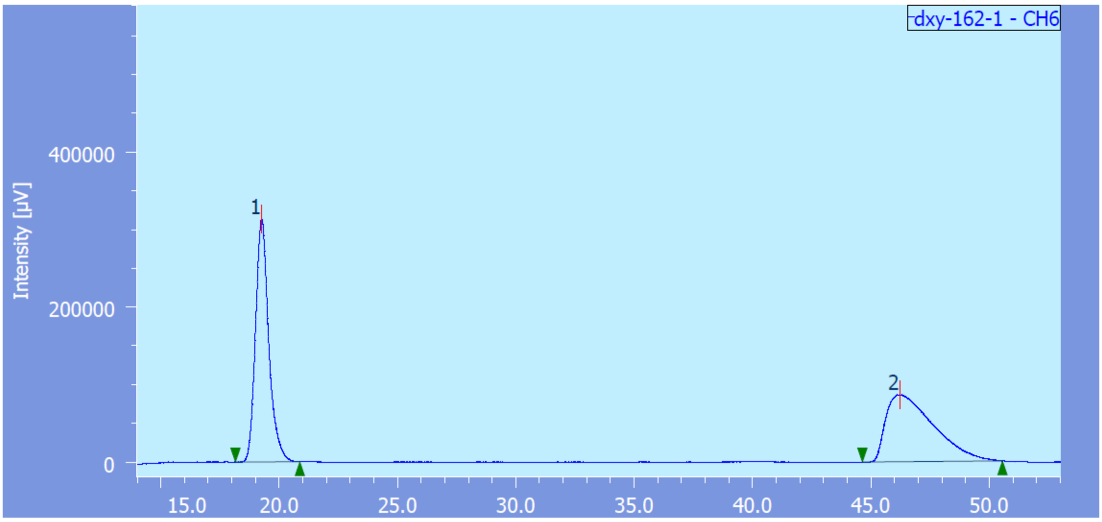

#	Peak Name	CH	tR [min]	Area [µV·sec]	Height [µV]	Area%	Height%	Quantity	NTP	Resolution	Symmetry Factor	Warning
1	Unknown	6	19.263	12192270	313441	50.181	78.363	N/A	6327	11.388	1.244	
2	Unknown	6	46.213	12104418	86546	49.819	21.637	N/A	2395	N/A	2.196	

HPLC spectrum of rac-**5**

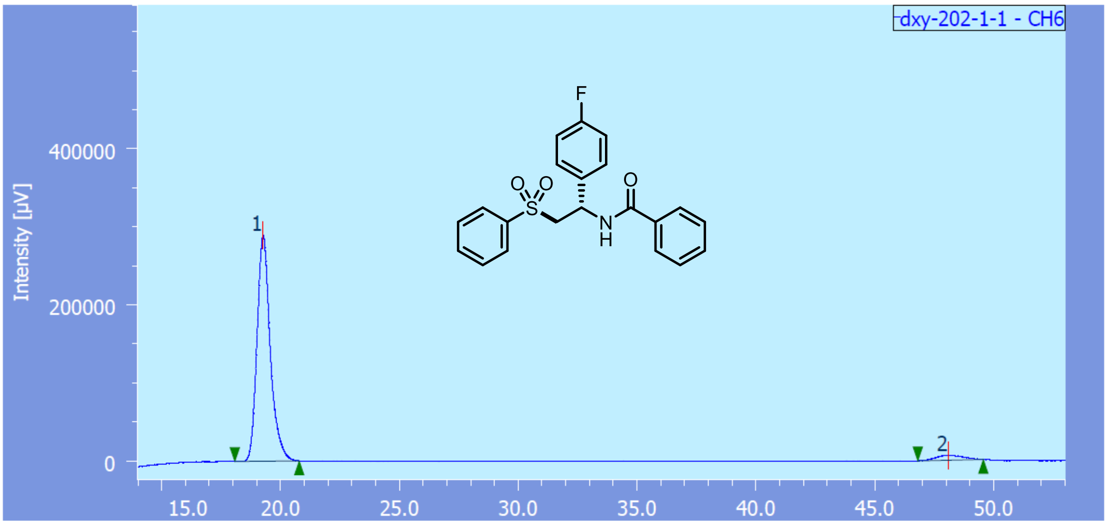

#	Peak Name	CH	tR [min]	Area [µV·sec]	Height [µV]	Area%	Height%	Quantity	NTP	Resolution	Symmetry Factor	Warning
1	Unknown	6	19.267	11201532	289249	95.690	97.879	N/A	6384	17.968	1.272	
2	Unknown	6	48.090	504501	6267	4.310	2.121	N/A	7293	N/A	1.132	

HPLC spectrum of **5**

(S)-*N*-(1-(4-chlorophenyl)-2-(phenylsulfonyl)ethyl)benzamide (6).

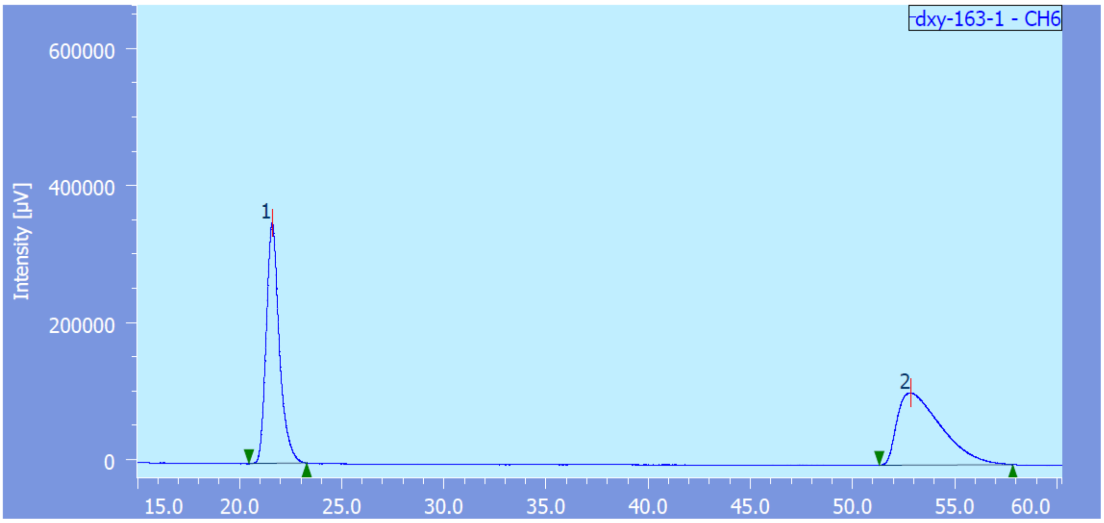

#	Peak Name	CH	tR [min]	Area [μV·sec]	Height [μV]	Area%	Height%	Quantity	NTP	Resolution	Symmetry Factor	Warning
1	Unknown	6	21.593	15648457	351128	49.846	76.938	N/A	6041	12.313	1.300	
2	Unknown	6	52.857	15745363	105250	50.154	23.062	N/A	2821	N/A	2.064	

HPLC spectrum of rac-**6**

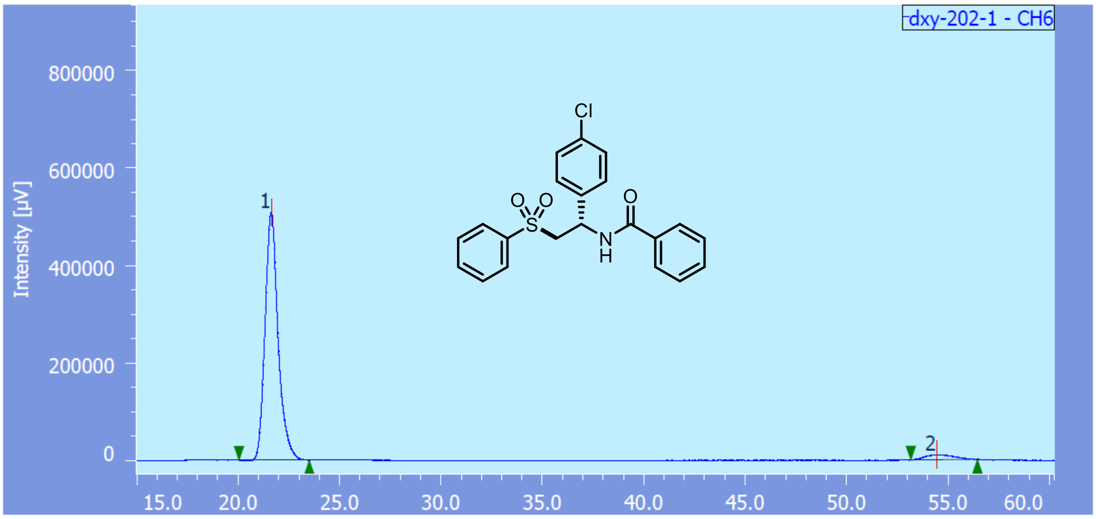

#	Peak Name	CH	tR [min]	Area [μV·sec]	Height [μV]	Area%	Height%	Quantity	NTP	Resolution	Symmetry Factor	Warning
1	Unknown	6	21.637	22832273	508394	95.634	97.970	N/A	5948	17.076	1.232	
2	Unknown	6	54.430	1042391	10534	4.366	2.030	N/A	6365	N/A	1.314	

HPLC spectrum of **6**

(*S*)-*N*-(2-(Phenylsulfonyl)-1-(4-(trifluoromethyl)phenyl)ethyl)benzamide (7).

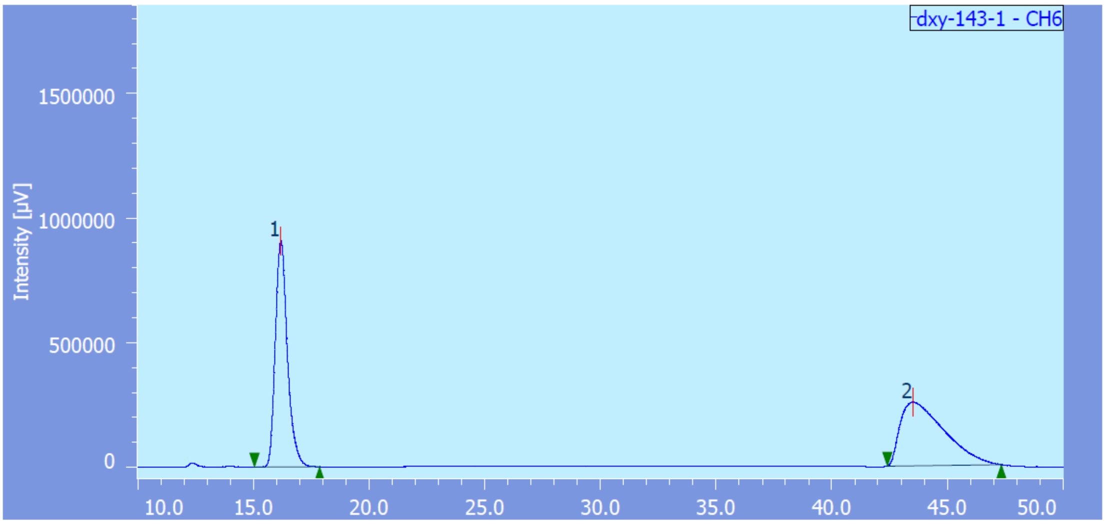

#	Peak Name	CH	tR [min]	Area [µV·sec]	Height [µV]	Area%	Height%	Quantity	NTP	Resolution	Symmetry Factor	Warning
1	Unknown	6	16.193	32897293	905204	49.257	78.052	N/A	4943	12.067	1.273	
2	Unknown	6	43.510	33890134	254538	50.743	21.948	N/A	2314	N/A	2.168	

HPLC spectrum of rac-**7**

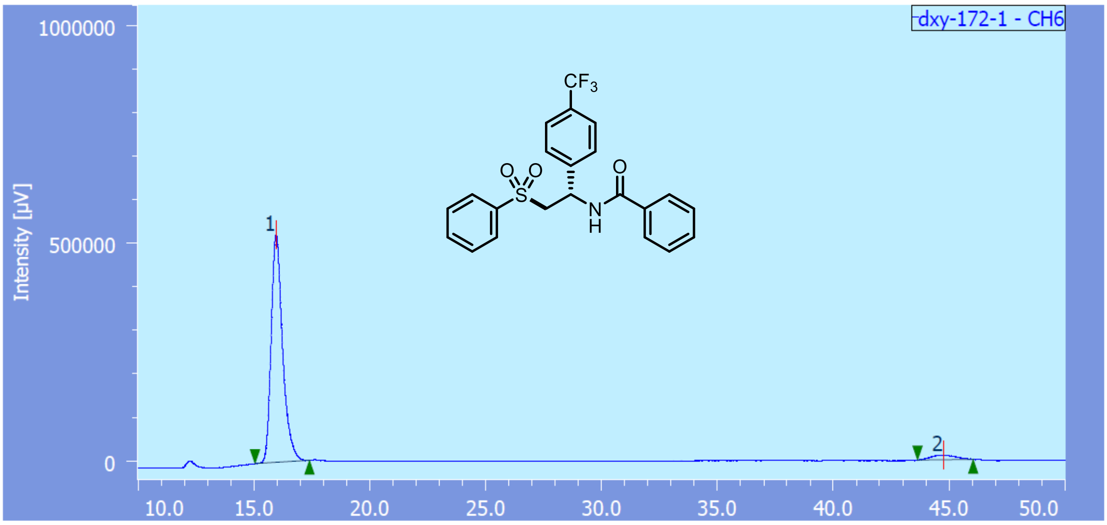

#	Peak Name	CH	tR [min]	Area [µV·sec]	Height [µV]	Area%	Height%	Quantity	NTP	Resolution	Symmetry Factor	Warning
1	Unknown	6	15.963	17974597	522757	95.769	98.045	N/A	5488	19.240	1.353	
2	Unknown	6	44.750	794043	10421	4.231	1.955	N/A	7007	N/A	1.075	

HPLC spectrum of **7**

(*S*)-*N*-(1-(4-Cyanophenyl)-2-(phenylsulfonyl)ethyl)benzamide (8).

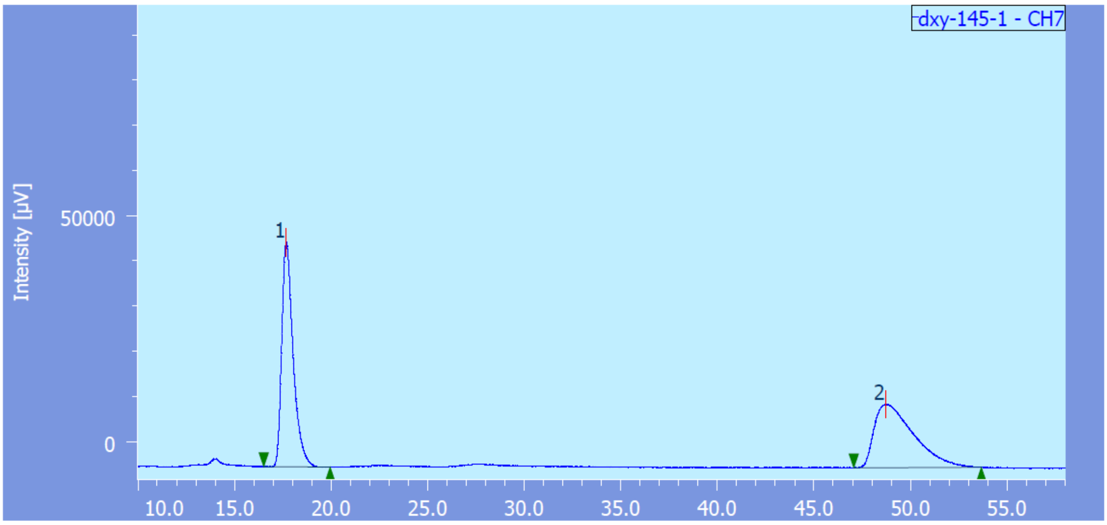

#	Peak Name	CH	tR [min]	Area [μV·sec]	Height [μV]	Area%	Height%	Quantity	NTP	Resolution	Symmetry Factor	Warning
1	Unknown	7	17.677	2043220	49741	50.595	77.990	N/A	4720	13.067	1.533	
2	Unknown	7	48.703	1995196	14038	49.405	22.010	N/A	2724	N/A	2.160	

HPLC spectrum of rac-**8**

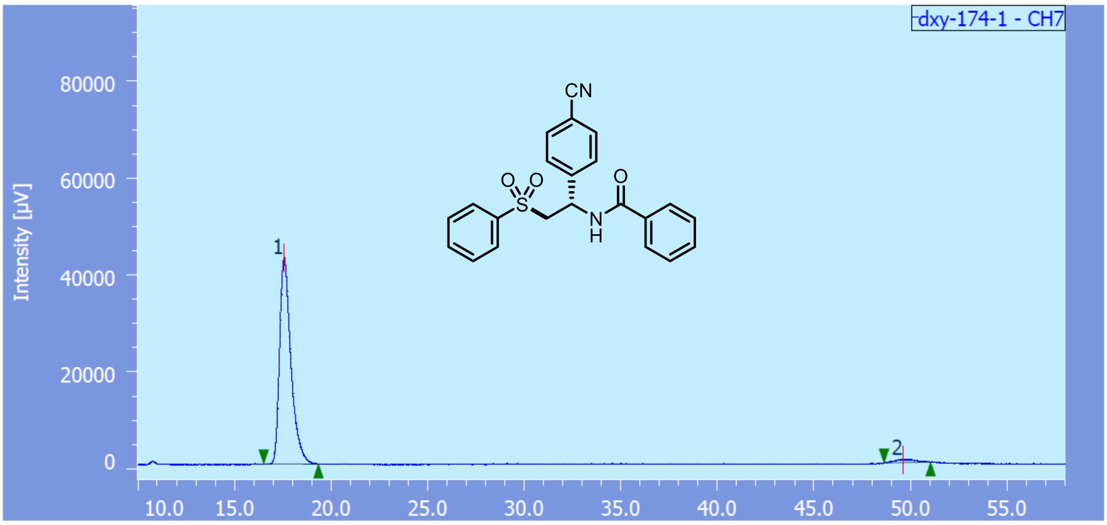

#	Peak Name	CH	tR [min]	Area [μV·sec]	Height [μV]	Area%	Height%	Quantity	NTP	Resolution	Symmetry Factor	Warning
1	Unknown	7	17.570	1731999	42498	97.190	98.489	N/A	4729	19.394	1.510	
2	Unknown	7	49.620	50069	652	2.810	1.511	N/A	7499	N/A	1.199	

HPLC spectrum of **8**

Ethyl (*S*)-4-(1-benzamido-2-(phenylsulfonyl)ethyl)benzoate (9).

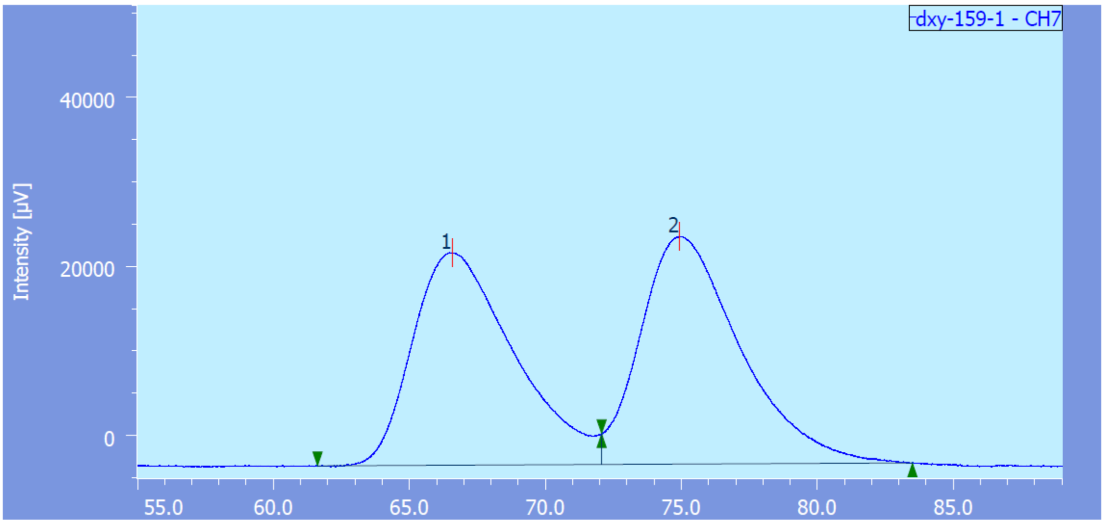

#	Peak Name	CH	tR [min]	Area [µV·sec]	Height [µV]	Area%	Height%	Quantity	NTP	Resolution	Symmetry Factor	Warning
1	Unknown	7	66.580	6537740	25159	48.715	48.285	N/A	1525	1.252	N/A	
2	Unknown	7	74.933	6882672	26946	51.285	51.715	N/A	2088	N/A	N/A	

HPLC spectrum of rac-**9**

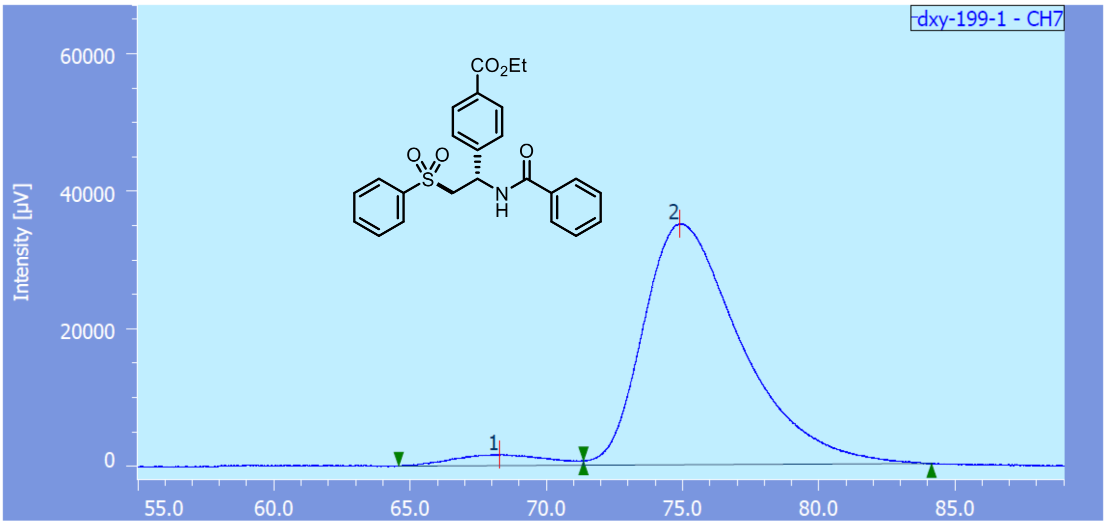

#	Peak Name	CH	tR [min]	Area [µV·sec]	Height [µV]	Area%	Height%	Quantity	NTP	Resolution	Symmetry Factor	Warning
1	Unknown	7	68.280	383598	1596	4.188	4.359	N/A	1530	0.995	N/A	
2	Unknown	7	74.903	8776750	35026	95.812	95.641	N/A	2212	N/A	1.579	

HPLC spectrum of **9**

(*S*)-*N*-(1-(4-Acetylphenyl)-2-(phenylsulfonyl)ethyl)benzamide (10).

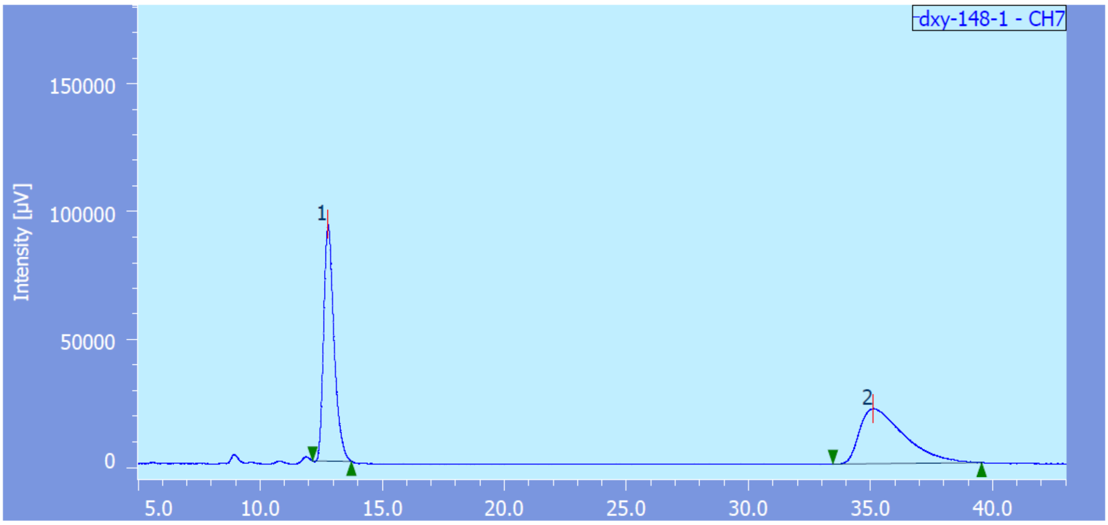

#	Peak Name	CH	tR [min]	Area [µV·sec]	Height [µV]	Area%	Height%	Quantity	NTP	Resolution	Symmetry Factor	Warning
1	Unknown	7	12.780	2730388	92406	50.022	81.115	N/A	4594	11.103	1.380	
2	Unknown	7	35.113	2728029	21514	49.978	18.885	N/A	1834	N/A	2.031	

HPLC spectrum of rac-**10**

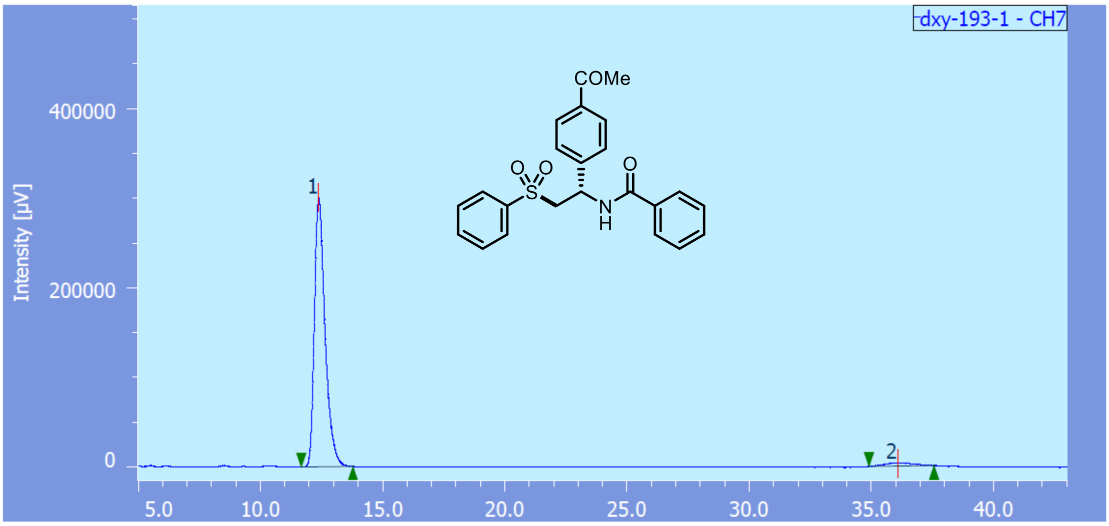

#	Peak Name	CH	tR [min]	Area [µV·sec]	Height [µV]	Area%	Height%	Quantity	NTP	Resolution	Symmetry Factor	Warning
1	Unknown	7	12.387	9120860	299920	96.894	98.911	N/A	4155	14.306	1.459	
2	Unknown	7	36.070	292372	3302	3.106	1.089	N/A	3199	N/A	1.139	

HPLC spectrum of **10**

(*S*)-*N*-(1-(4-Formylphenyl)-2-(phenylsulfonyl)ethyl)benzamide (11).

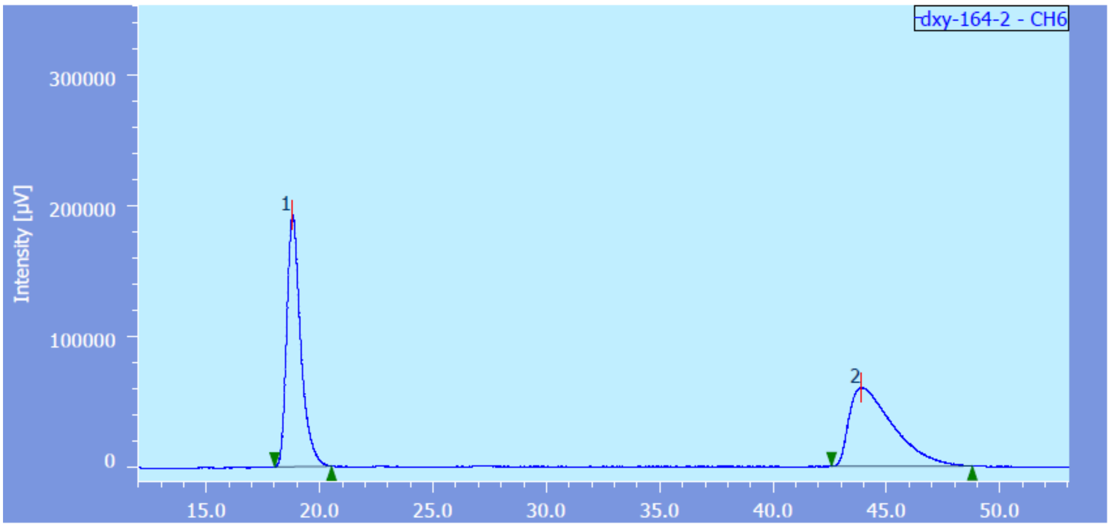

#	Peak Name	CH	tR [min]	Area [µV·sec]	Height [µV]	Area%	Height%	Quantity	NTP	Resolution	Symmetry Factor	Warning
1	Unknown	6	18.803	8208540	192494	50.350	76.239	N/A	4983	10.961	1.447	
2	Unknown	6	43.900	8094358	59993	49.650	23.761	N/A	2480	N/A	2.287	

HPLC spectrum of rac-**11**

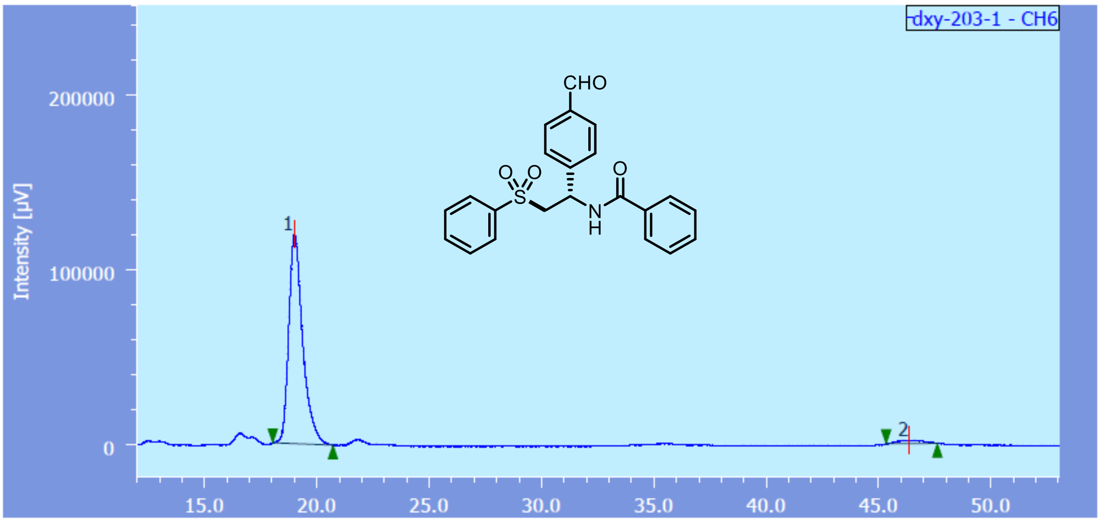

#	Peak Name	CH	tR [min]	Area [µV·sec]	Height [µV]	Area%	Height%	Quantity	NTP	Resolution	Symmetry Factor	Warning
1	Unknown	6	19.007	5176979	119694	96.975	98.342	N/A	5016	16.497	1.431	
2	Unknown	6	46.370	161473	2019	3.025	1.658	N/A	6780	N/A	1.112	

HPLC spectrum of **11**

(*S*)-*N*-(2-(Phenylsulfonyl)-1-(4-(4,4,5,5-tetramethyl-1,3,2-dioxaborolan-2-yl)phenyl)ethyl)benzamide (12).

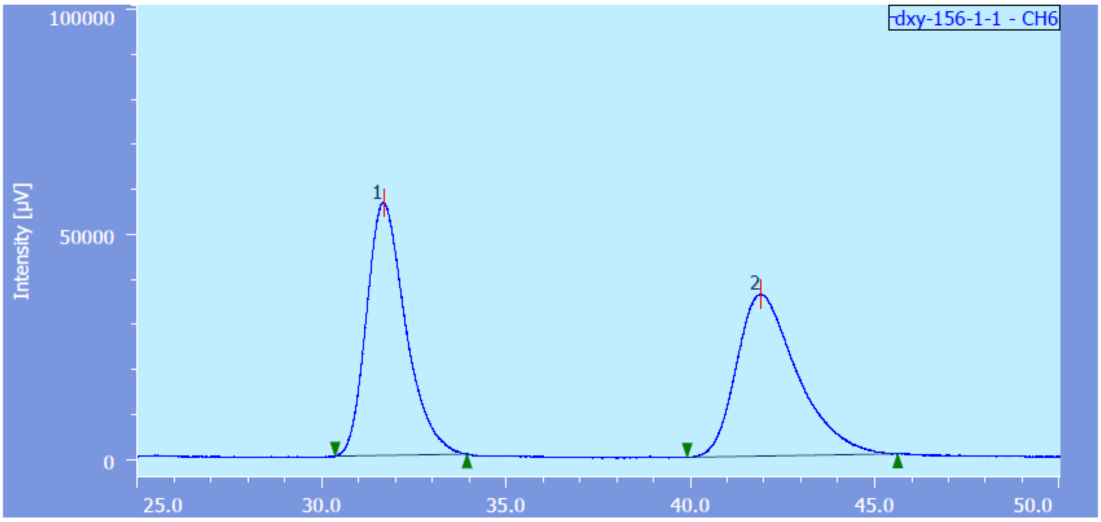

#	Peak Name	CH	tR [min]	Area [µV·sec]	Height [µV]	Area%	Height%	Quantity	NTP	Resolution	Symmetry Factor	Warning
1	Unknown	6	31.680	4135322	56081	50.085	60.954	N/A	4493	4.252	1.317	
2	Unknown	6	41.917	4121209	35924	49.915	39.046	N/A	3258	N/A	1.444	

HPLC spectrum of rac-**12**

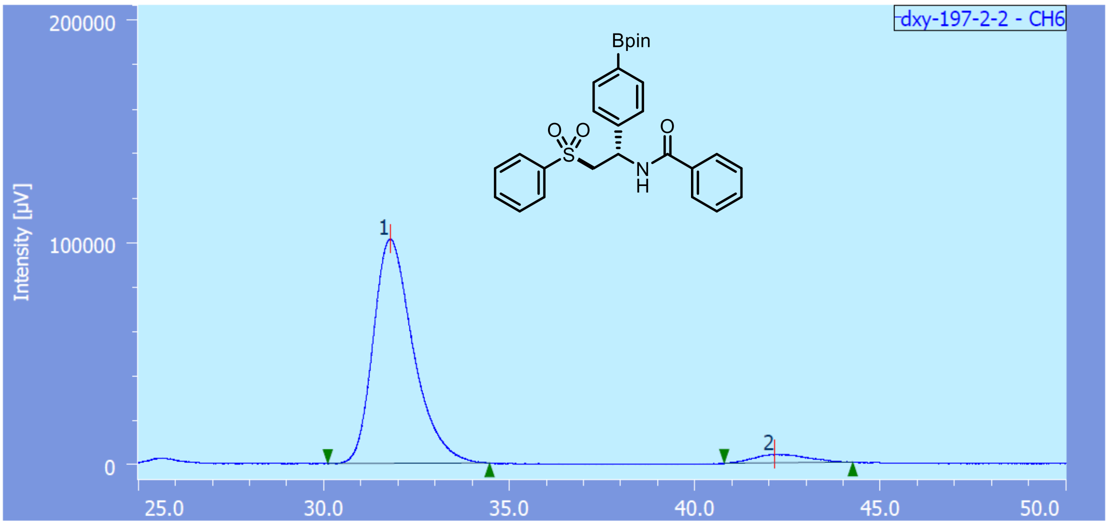

#	Peak Name	CH	tR [min]	Area [µV·sec]	Height [µV]	Area%	Height%	Quantity	NTP	Resolution	Symmetry Factor	Warning
1	Unknown	6	31.793	7567696	101039	95.181	96.302	N/A	4422	4.514	1.358	
2	Unknown	6	42.160	383183	3880	4.819	3.698	N/A	3921	N/A	1.282	

HPLC spectrum of **12**

(*S*)-*N*-(1-(4-(1H-Pyrrol-1-yl)phenyl)-2-(phenylsulfonyl)ethyl)benzamide (13).

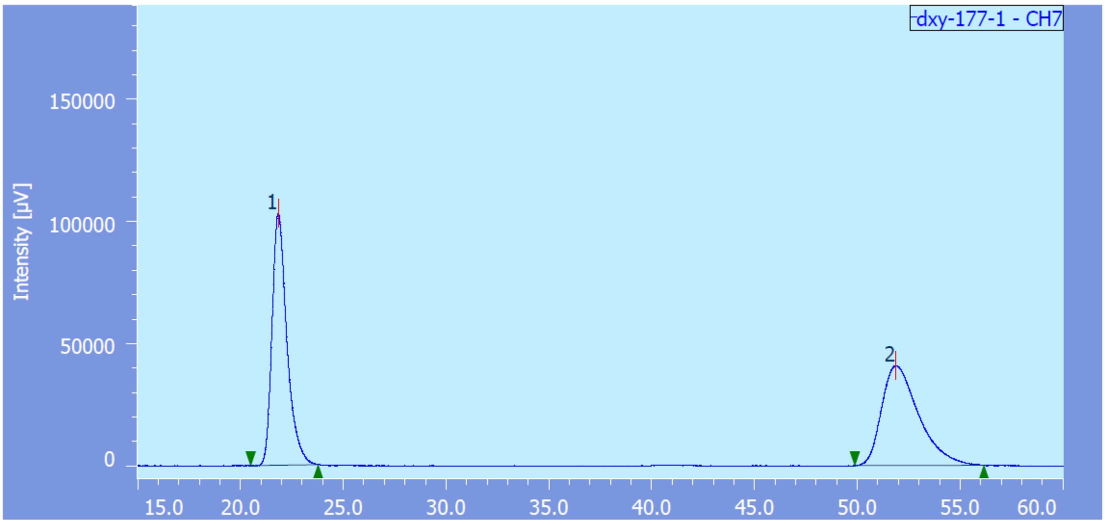

#	Peak Name	CH	tR [min]	Area [µV·sec]	Height [µV]	Area%	Height%	Quantity	NTP	Resolution	Symmetry Factor	Warning
1	Unknown	7	21.843	5172982	103013	50.231	71.571	N/A	4850	13.478	1.401	
2	Unknown	7	51.857	5125497	40918	49.769	28.429	N/A	4173	N/A	1.544	

HPLC spectrum of rac-**13**

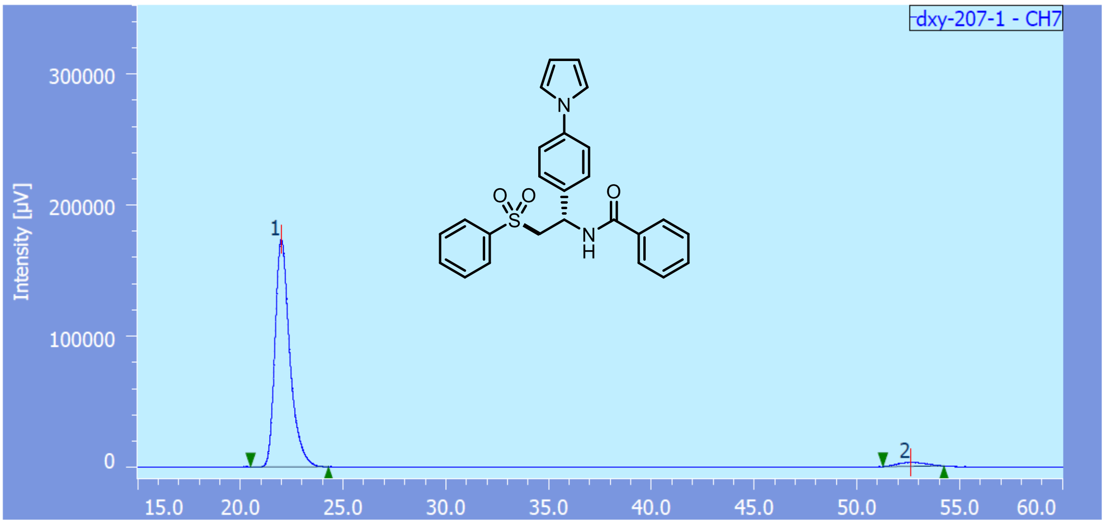

#	Peak Name	CH	tR [min]	Area [µV·sec]	Height [µV]	Area%	Height%	Quantity	NTP	Resolution	Symmetry Factor	Warning
1	Unknown	7	21.997	8696489	173335	96.876	98.278	N/A	4921	15.956	1.384	
2	Unknown	7	52.623	280451	3037	3.124	1.722	N/A	6581	N/A	1.078	

HPLC spectrum of **13**

(*S*)-*N*-(1-(3-Methoxyphenyl)-2-(phenylsulfonyl)ethyl)benzamide (14).

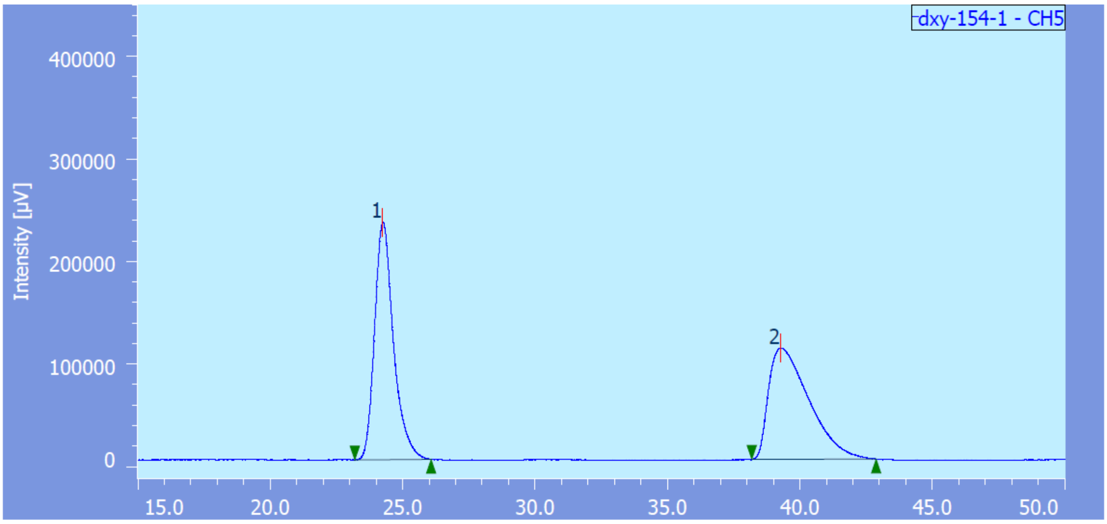

#	Peak Name	CH	tR [min]	Area [µV·sec]	Height [µV]	Area%	Height%	Quantity	NTP	Resolution	Symmetry Factor	Warning
1	Unknown	5	24.247	11498818	231746	49.913	68.096	N/A	6126	7.408	1.338	
2	Unknown	5	39.263	11539040	108578	50.087	31.904	N/A	3089	N/A	2.067	

HPLC spectrum of rac-**14**

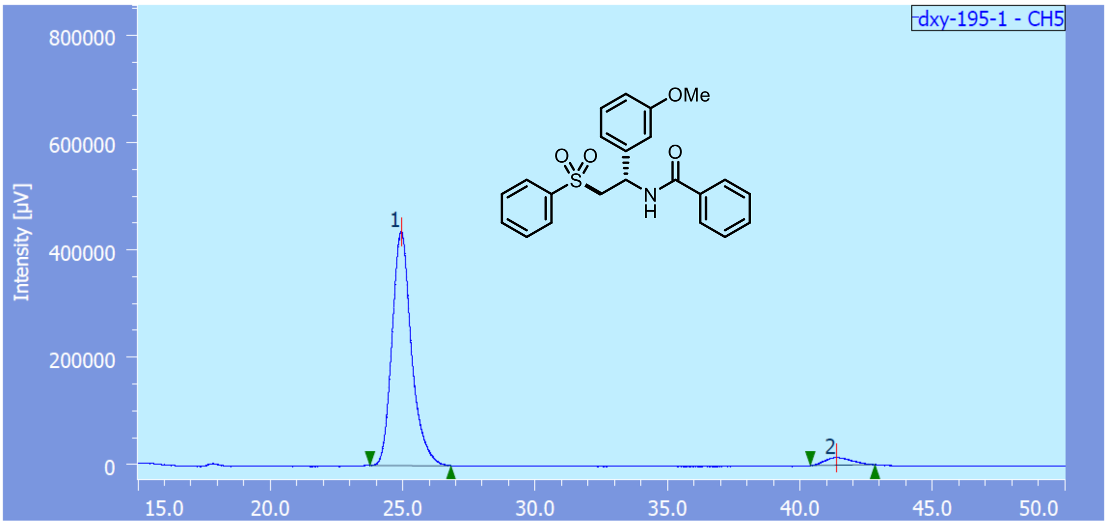

#	Peak Name	CH	tR [min]	Area [µV·sec]	Height [µV]	Area%	Height%	Quantity	NTP	Resolution	Symmetry Factor	Warning
1	Unknown	5	24.933	22332135	436825	95.604	96.861	N/A	6071	10.048	1.255	
2	Unknown	5	41.370	1026847	14154	4.396	3.139	N/A	6843	N/A	1.255	

HPLC spectrum of **14**

(*S*)-*N*-(2-(Phenylsulfonyl)-1-(*m*-tolyl)ethyl)benzamide (15).

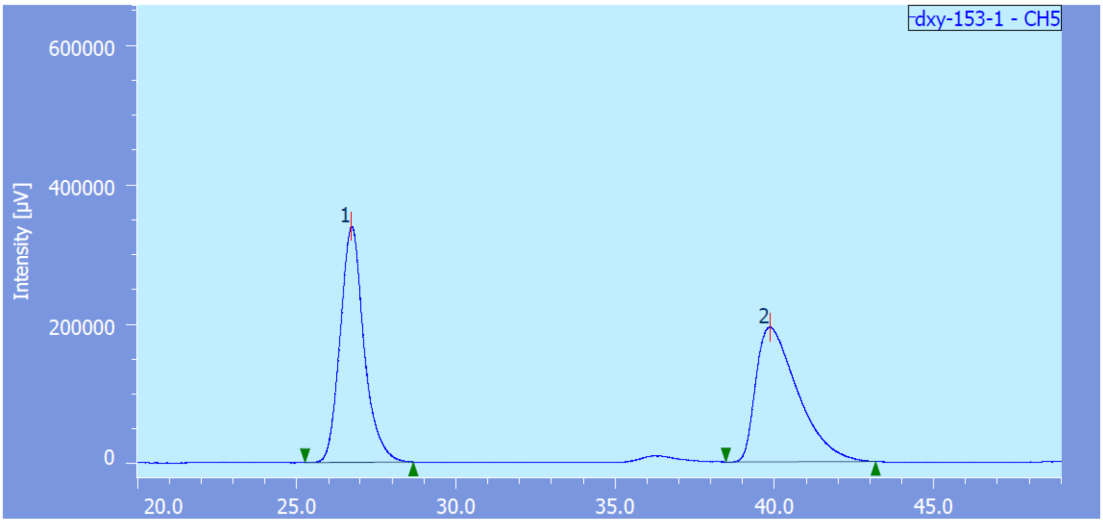

#	Peak Name	CH	tR [min]	Area [µV·sec]	Height [µV]	Area%	Height%	Quantity	NTP	Resolution	Symmetry Factor	Warning
1	Unknown	5	26.723	17595830	338403	49.852	63.696	N/A	6716	7.126	1.203	
2	Unknown	5	39.853	17700157	192873	50.148	36.304	N/A	4448	N/A	1.865	

HPLC spectrum of rac-**15**

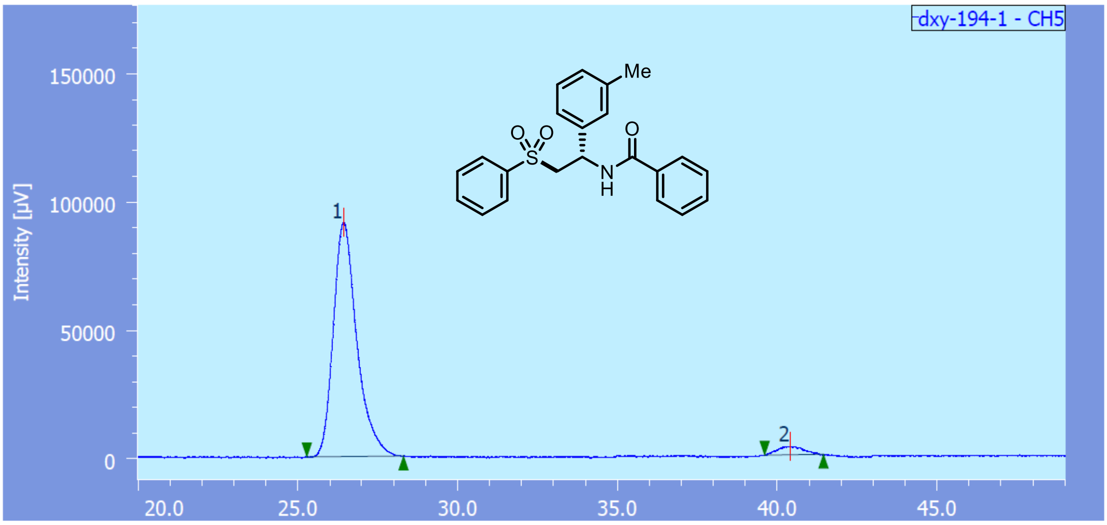

#	Peak Name	CH	tR [min]	Area [µV·sec]	Height [µV]	Area%	Height%	Quantity	NTP	Resolution	Symmetry Factor	Warning
1	Unknown	5	26.427	4671552	91219	96.095	96.431	N/A	6778	9.790	1.359	
2	Unknown	5	40.400	189842	3377	3.905	3.569	N/A	10483	N/A	1.094	

HPLC spectrum of **15**

Dimethyl (*S*)-5-(1-benzamido-2-(phenylsulfonyl)ethyl)isophthalate (16).

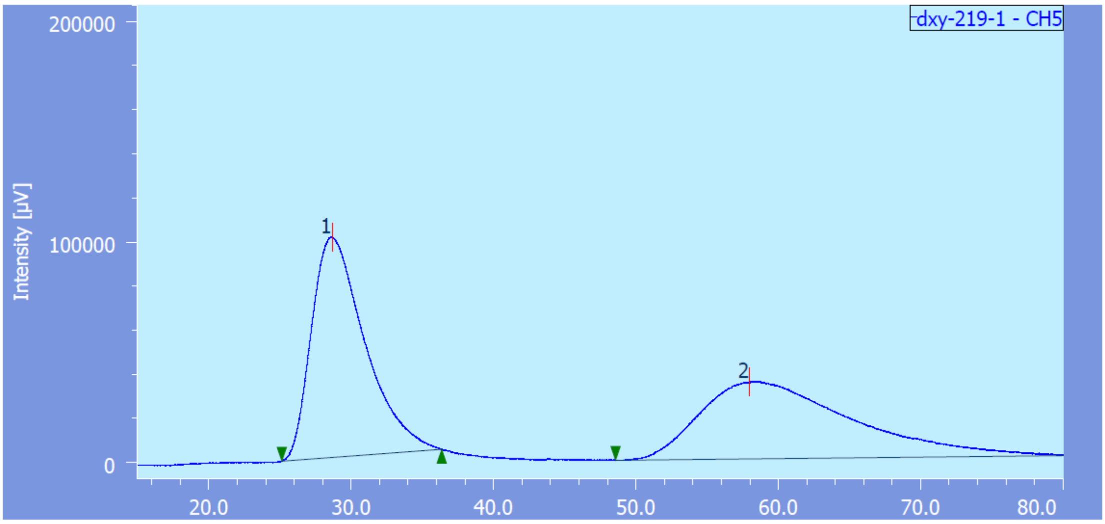

#	Peak Name	CH	tR [min]	Area [µV·sec]	Height [µV]	Area%	Height%	Quantity	NTP	Resolution	Symmetry Factor	Warning
1	Unknown	5	28.653	26099771	99883	50.176	74.038	N/A	286	2.280	1.582	
2	Unknown	5	57.967	25916502	35026	49.824	25.962	N/A	149	N/A	1.832	

HPLC spectrum of rac-**16**

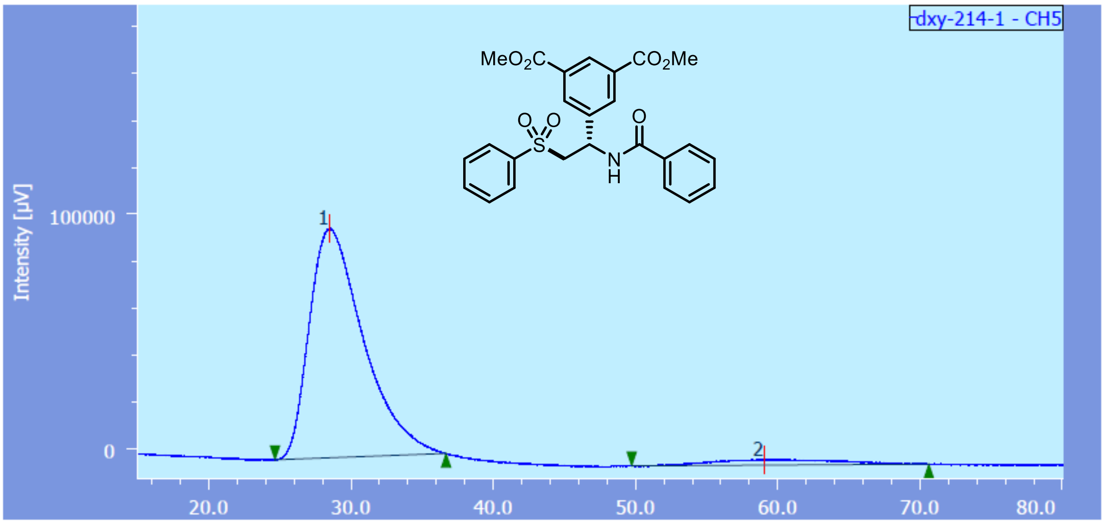

#	Peak Name	CH	tR [min]	Area [µV·sec]	Height [µV]	Area%	Height%	Quantity	NTP	Resolution	Symmetry Factor	Warning
1	Unknown	5	28.523	25922450	97829	94.927	97.652	N/A	278	2.371	1.606	
2	Unknown	5	59.060	1385420	2352	5.073	2.348	N/A	155	N/A	1.191	

HPLC spectrum of **16**

(*S*)-*N*-(1-(2-Methoxyphenyl)-2-(phenylsulfonyl)ethyl)benzamide (17).

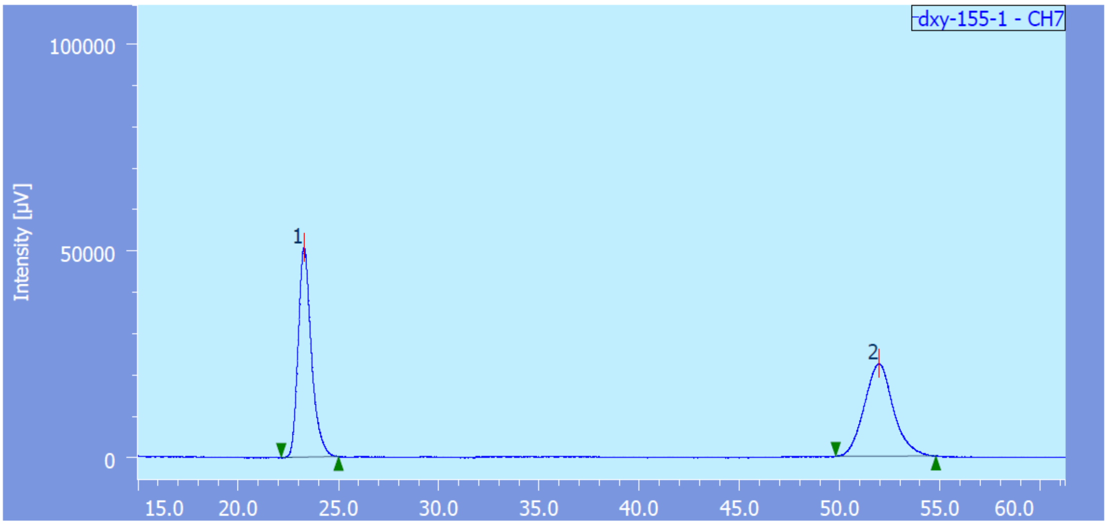

#	Peak Name	CH	tR [min]	Area [µV·sec]	Height [µV]	Area%	Height%	Quantity	NTP	Resolution	Symmetry Factor	Warning
1	Unknown	7	23.273	2321196	50697	50.660	69.333	N/A	6548	15.444	1.330	
2	Unknown	7	51.967	2260708	22424	49.340	30.667	N/A	6515	N/A	1.093	

HPLC spectrum of rac-**17**

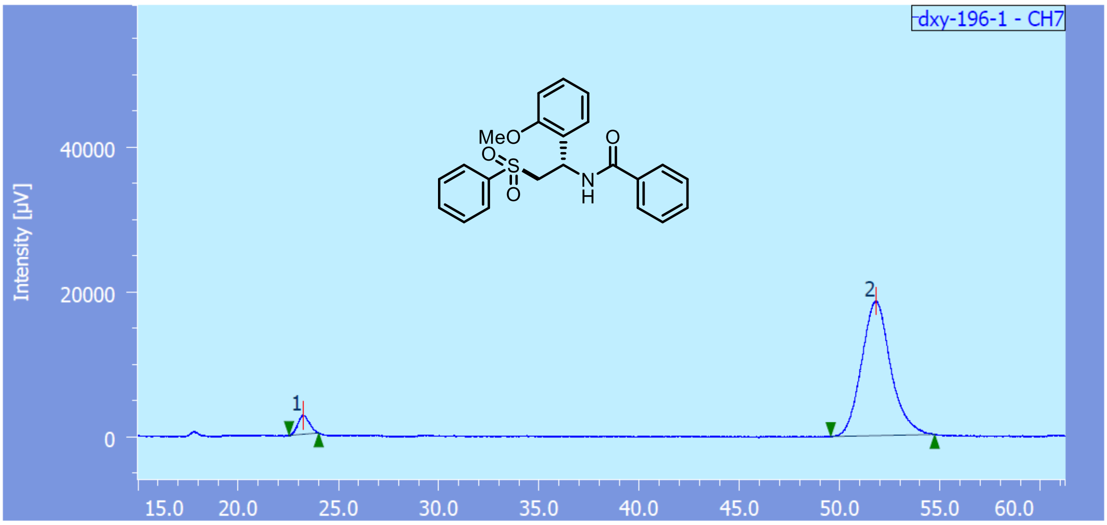

#	Peak Name	CH	tR [min]	Area [µV·sec]	Height [µV]	Area%	Height%	Quantity	NTP	Resolution	Symmetry Factor	Warning
1	Unknown	7	23.233	103336	2620	5.219	12.358	N/A	7436	15.672	1.091	
2	Unknown	7	51.797	1876766	18580	94.781	87.642	N/A	6463	N/A	1.149	

HPLC spectrum of **17**

(*S*)-*N*-(1-(2-Fluorophenyl)-2-(phenylsulfonyl)ethyl)benzamide (18).

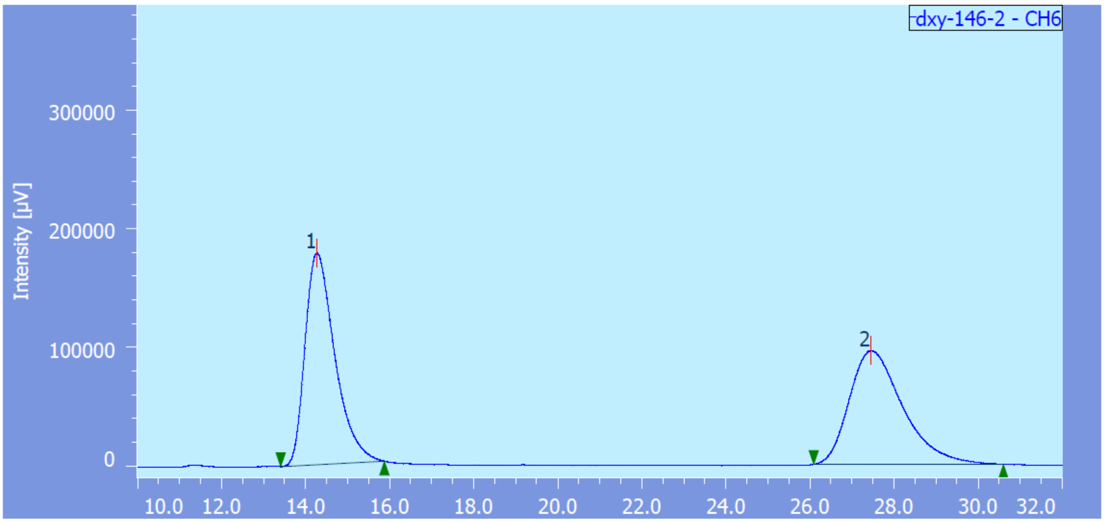

#	Peak Name	CH	tR [min]	Area [μV·sec]	Height [μV]	Area%	Height%	Quantity	NTP	Resolution	Symmetry Factor	Warning
1	Unknown	6	14.267	8795849	178651	50.903	65.063	N/A	2032	7.485	1.496	
2	Unknown	6	27.443	8483852	95931	49.097	34.937	N/A	2350	N/A	1.451	

HPLC spectrum of rac-**18**

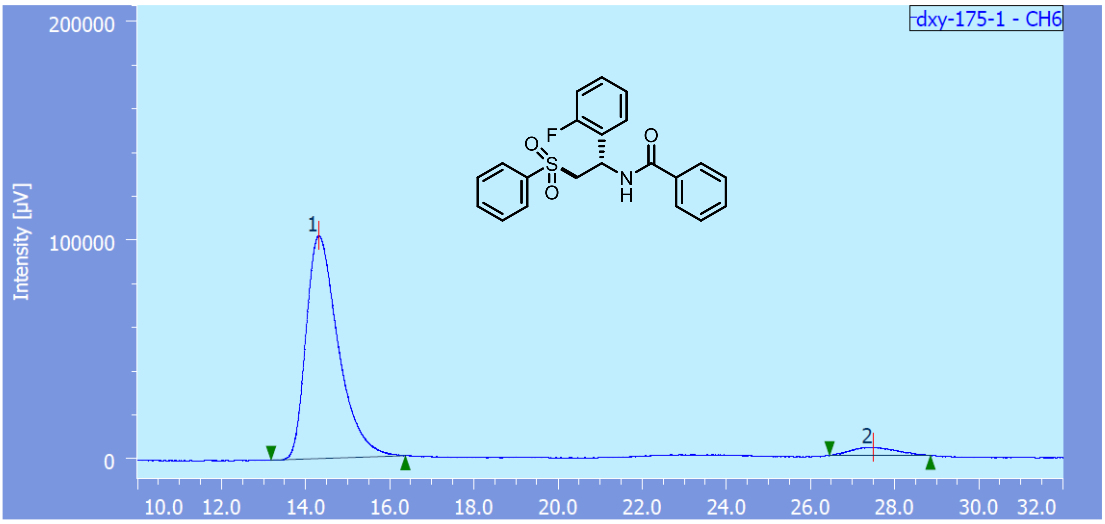

#	Peak Name	CH	tR [min]	Area [μV·sec]	Height [μV]	Area%	Height%	Quantity	NTP	Resolution	Symmetry Factor	Warning
1	Unknown	6	14.313	5421533	101914	95.039	96.493	N/A	1777	7.535	1.514	
2	Unknown	6	27.487	283001	3704	4.961	3.507	N/A	2621	N/A	1.164	

HPLC spectrum of **18**

(*S*)-*N*-(1-Phenyl-2-(phenylsulfonyl)ethyl)benzamide (19).

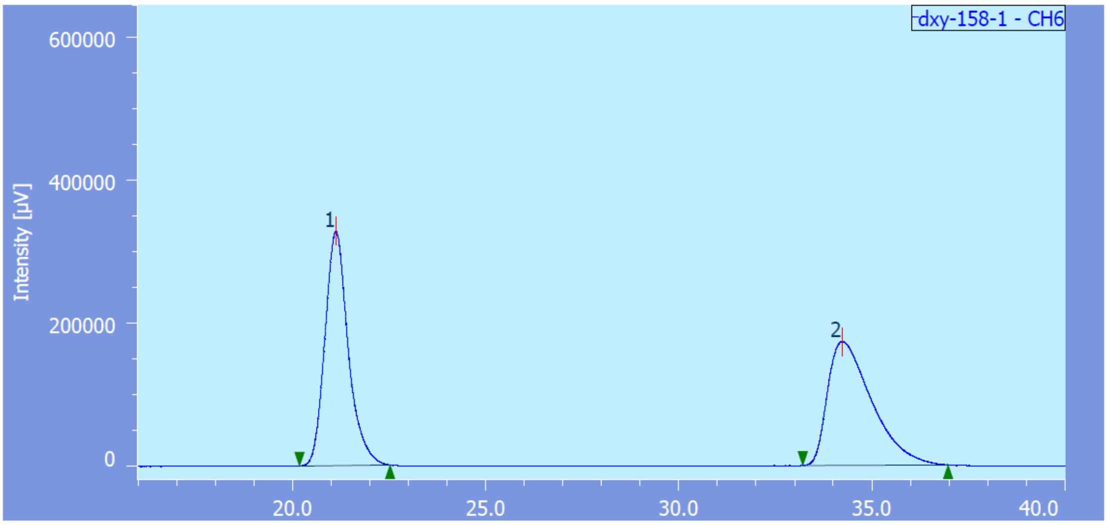

#	Peak Name	CH	tR [min]	Area [μV·sec]	Height [μV]	Area%	Height%	Quantity	NTP	Resolution	Symmetry Factor	Warning
1	Unknown	6	21.123	13750454	327724	49.972	65.415	N/A	6396	8.410	1.234	
2	Unknown	6	34.227	13765917	173265	50.028	34.585	N/A	4383	N/A	1.846	

HPLC spectrum of rac-**19**

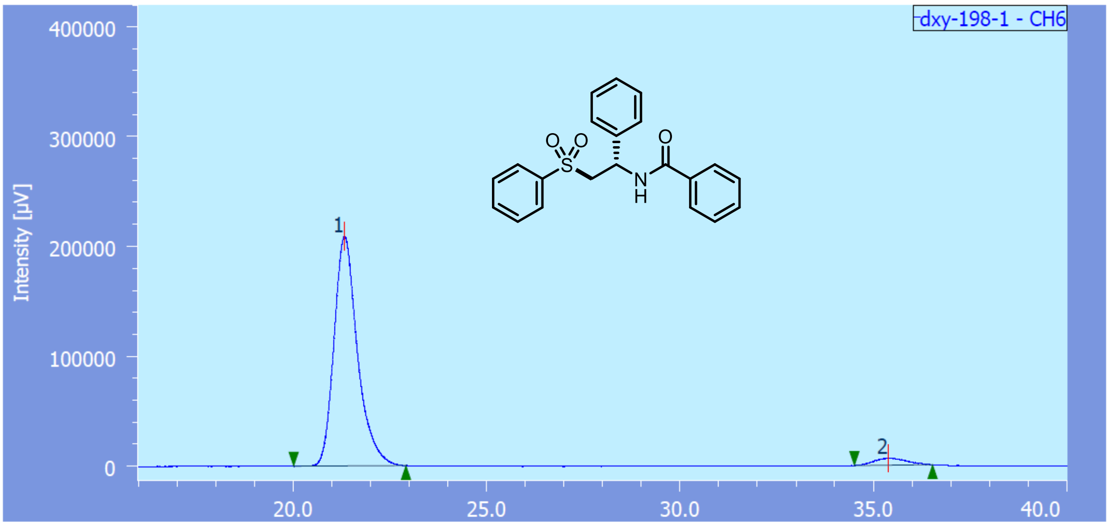

#	Peak Name	CH	tR [min]	Area [μV·sec]	Height [μV]	Area%	Height%	Quantity	NTP	Resolution	Symmetry Factor	Warning
1	Unknown	6	21.333	8802841	208765	95.950	97.042	N/A	6537	10.646	1.303	
2	Unknown	6	35.383	371534	6364	4.050	2.958	N/A	7914	N/A	1.162	

HPLC spectrum of **19**

(*S*)-*N*-(1-(Naphthale*N*-2-yl)-2-(phenylsulfonyl)ethyl)benzamide (20).

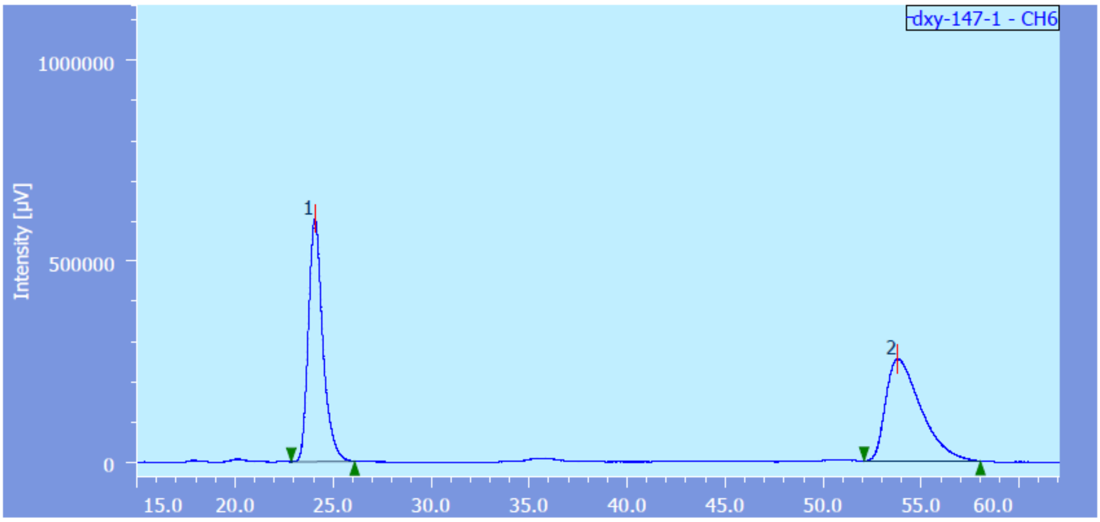

#	Peak Name	CH	tR [min]	Area [µV·sec]	Height [µV]	Area%	Height%	Quantity	NTP	Resolution	Symmetry Factor	Warning
1	Unknown	6	24.070	30980303	605123	49.360	70.458	N/A	5606	13.124	1.306	
2	Unknown	6	53.800	31783484	253719	50.640	29.542	N/A	4366	N/A	1.761	

HPLC spectrum of rac-**20**

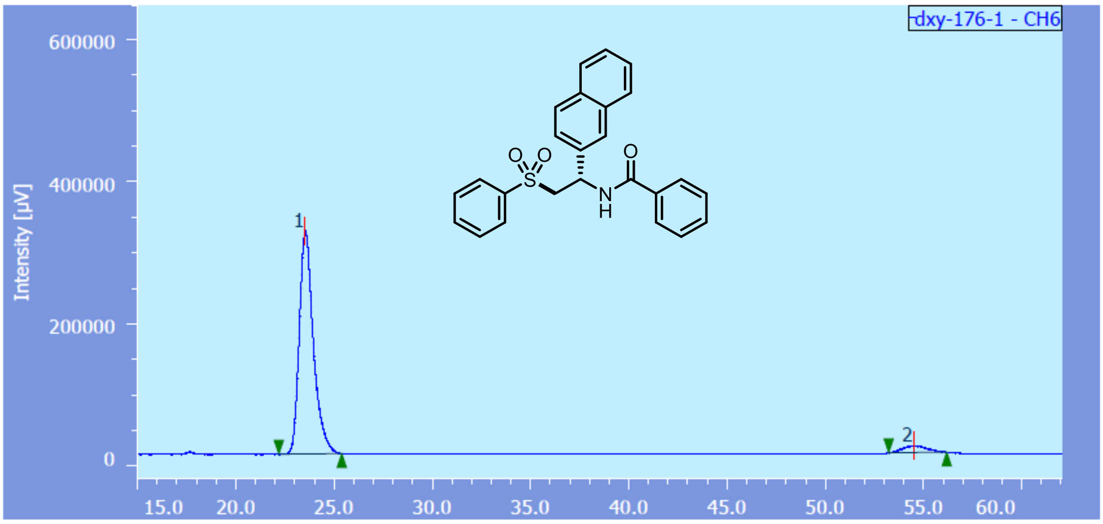

#	Peak Name	CH	tR [min]	Area [µV·sec]	Height [µV]	Area%	Height%	Quantity	NTP	Resolution	Symmetry Factor	Warning
1	Unknown	6	23.537	15590423	313046	94.849	97.070	N/A	5742	16.648	1.373	
2	Unknown	6	54.490	846642	9449	5.151	2.930	N/A	7686	N/A	1.145	

HPLC spectrum of **20**

(*S*)-*N*-(1-(4-(Tert-butyl)phenyl)-2-(phenylsulfonyl)ethyl)benzamide (21).

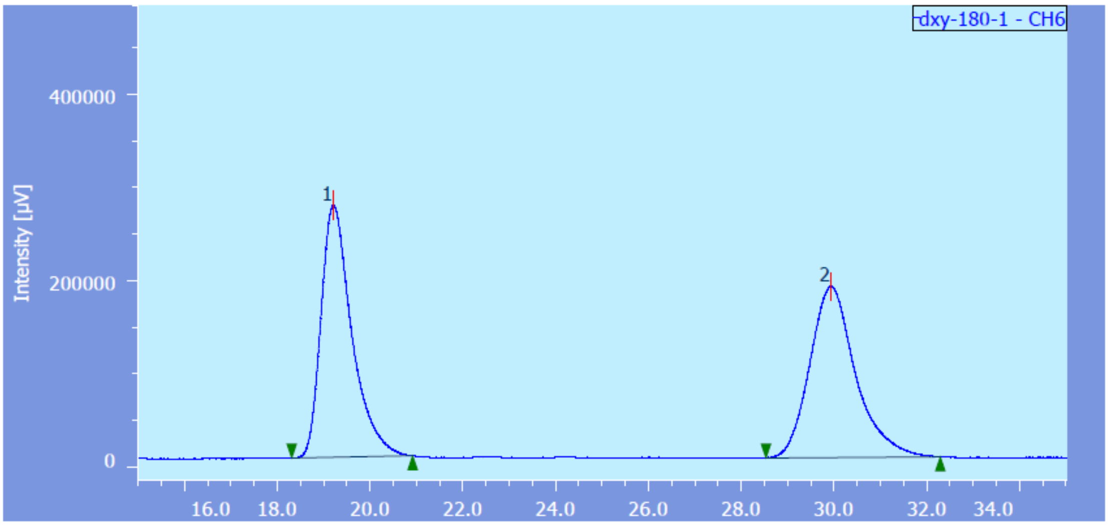

#	Peak Name	CH	tR [min]	Area [µV·sec]	Height [µV]	Area%	Height%	Quantity	NTP	Resolution	Symmetry Factor	Warning
1	Unknown	6	19.210	12467742	270360	49.732	59.573	N/A	4439	7.499	1.500	
2	Unknown	6	29.933	12602120	183473	50.268	40.427	N/A	4880	N/A	1.260	

HPLC spectrum of rac-**21**

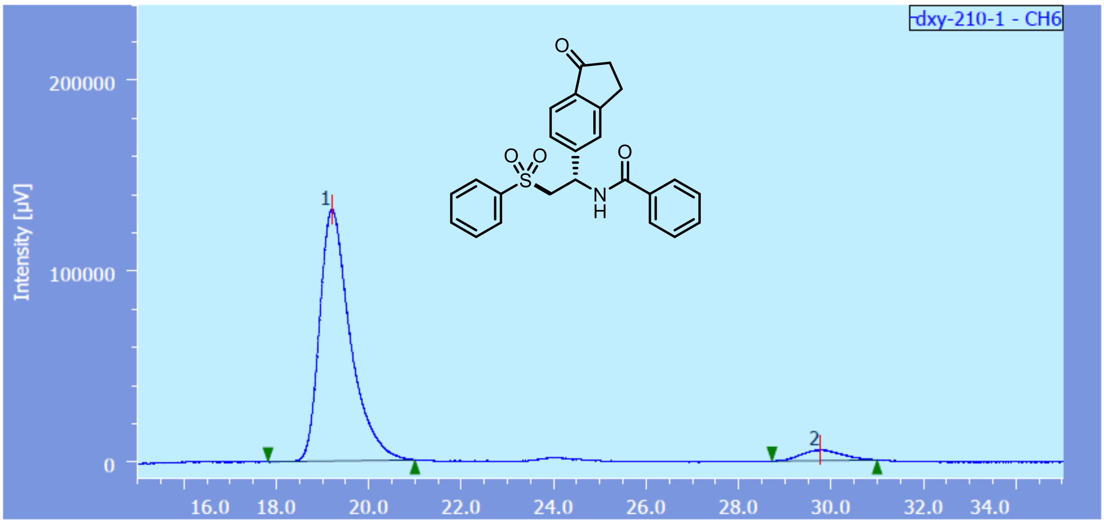

#	Peak Name	CH	tR [min]	Area [µV·sec]	Height [µV]	Area%	Height%	Quantity	NTP	Resolution	Symmetry Factor	Warning
1	Unknown	6	19.200	6075638	131615	94.566	95.833	N/A	4470	7.577	1.516	
2	Unknown	6	29.777	349088	5723	5.434	4.167	N/A	5208	N/A	1.113	

HPLC spectrum of **21**

(*S*)-*N*-(1-(2,3-dihydrobenzo[b][1,4]dioxin-6-yl)-2-(phenylsulfonyl)ethyl)benzamide (22).

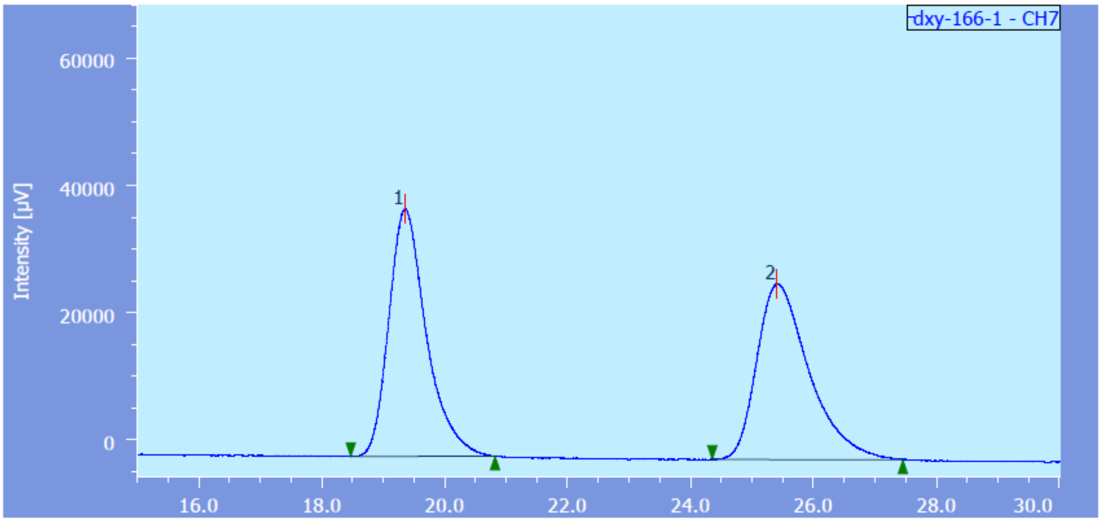

#	Peak Name	CH	tR [min]	Area [µV·sec]	Height [µV]	Area%	Height%	Quantity	NTP	Resolution	Symmetry Factor	Warning
1	Unknown	7	19.350	1648316	38856	50.188	58.474	N/A	5290	4.724	1.368	
2	Unknown	7	25.400	1635983	27594	49.812	41.526	N/A	4564	N/A	1.529	

HPLC spectrum of rac-**22**

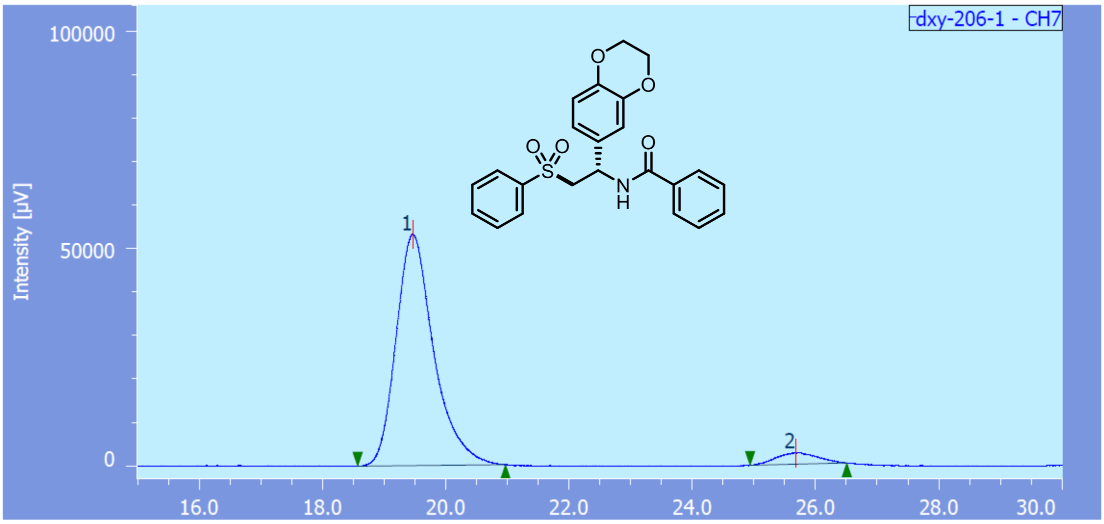

#	Peak Name	CH	tR [min]	Area [µV·sec]	Height [µV]	Area%	Height%	Quantity	NTP	Resolution	Symmetry Factor	Warning
1	Unknown	7	19.467	2257880	53188	94.859	95.276	N/A	5364	5.284	1.320	
2	Unknown	7	25.677	122378	2637	5.141	4.724	N/A	6306	N/A	1.079	

HPLC spectrum of **22**

(*S*)-*N*-(2-(Phenylsulfonyl)-1-(quinolin-7-yl)ethyl)benzamide (23).

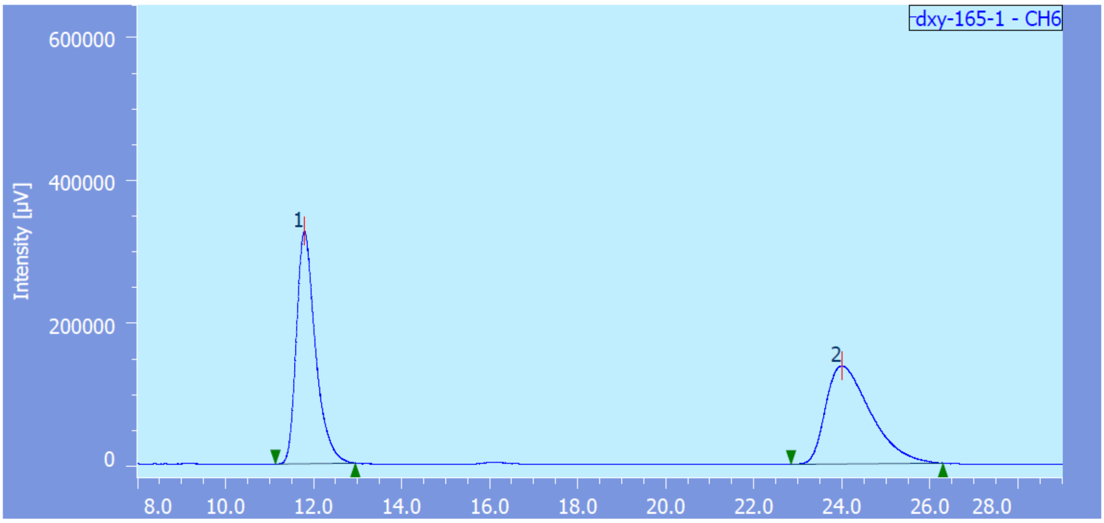

#	Peak Name	CH	tR [min]	Area [µV·sec]	Height [µV]	Area%	Height%	Quantity	NTP	Resolution	Symmetry Factor	Warning
1	Unknown	6	11.790	9754165	325250	50.030	70.266	N/A	3918	9.416	1.414	
2	Unknown	6	24.000	9742444	137634	49.970	29.734	N/A	2701	N/A	1.636	

HPLC spectrum of rac-**23**

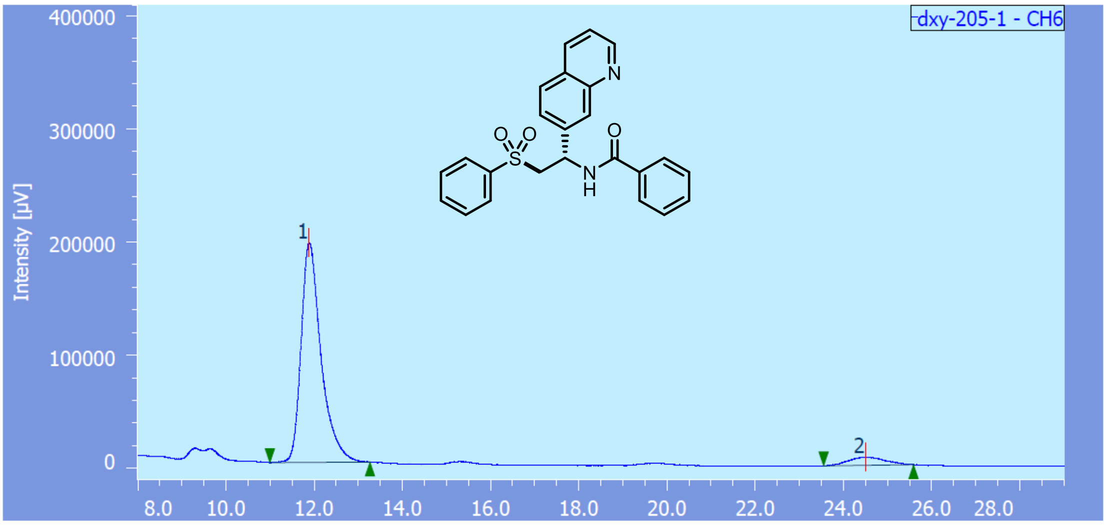

#	Peak Name	CH	tR [min]	Area [µV·sec]	Height [µV]	Area%	Height%	Quantity	NTP	Resolution	Symmetry Factor	Warning
1	Unknown	6	11.887	6112620	194347	93.648	96.421	N/A	3785	10.840	1.467	
2	Unknown	6	24.490	414626	7214	6.352	3.579	N/A	3949	N/A	1.131	

HPLC spectrum of **23**

(*S*)-*N*-(2-(Phenylsulfonyl)-1-(thiophen-3-yl)ethyl)benzamide (24).

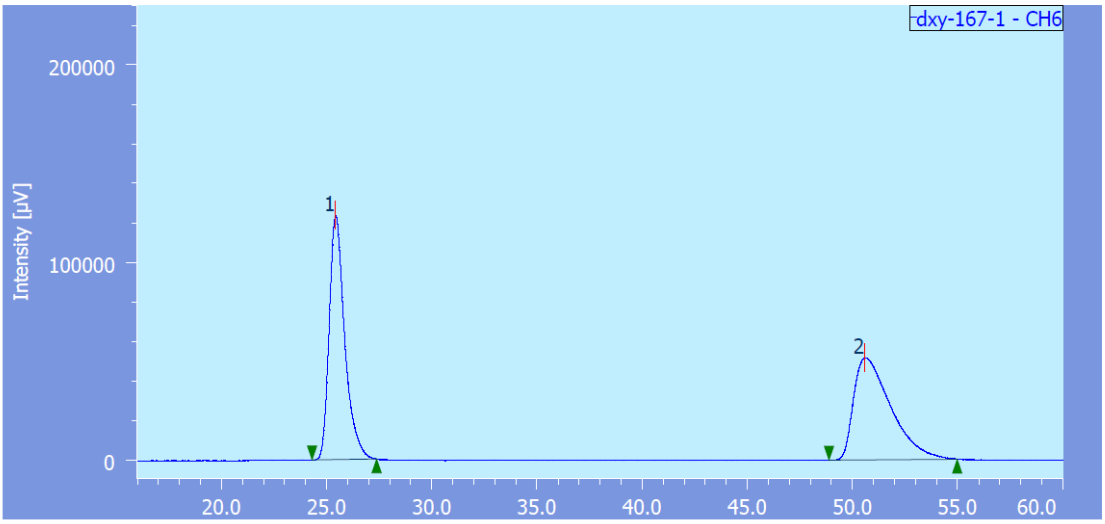

#	Peak Name	CH	tR [min]	Area [µV·sec]	Height [µV]	Area%	Height%	Quantity	NTP	Resolution	Symmetry Factor	Warning
1	Unknown	6	25.430	6343761	123491	49.938	70.510	N/A	6265	11.243	1.421	
2	Unknown	6	50.590	6359474	51649	50.062	29.490	N/A	3993	N/A	1.995	

HPLC spectrum of rac-**24**

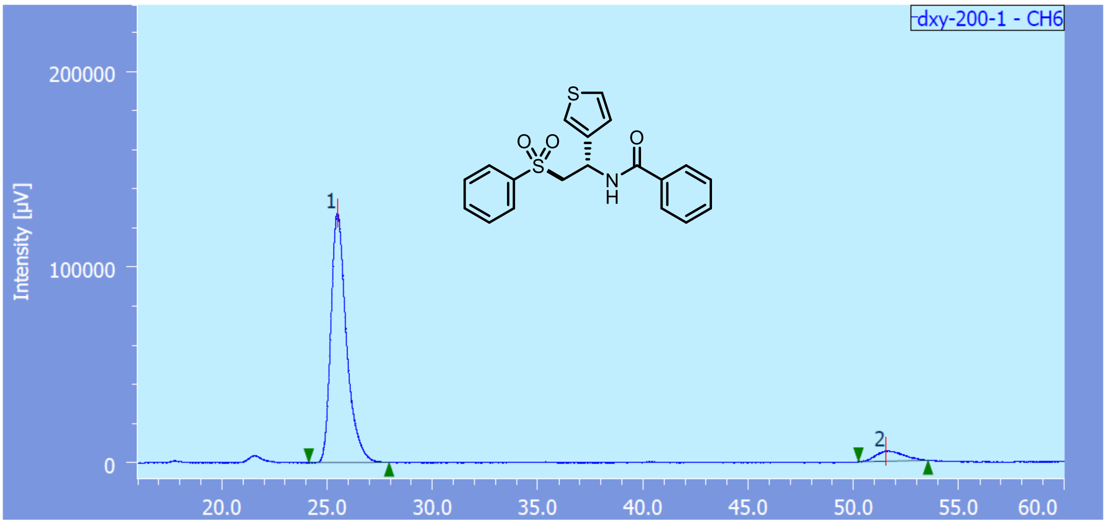

#	Peak Name	CH	tR [min]	Area [µV·sec]	Height [µV]	Area%	Height%	Quantity	NTP	Resolution	Symmetry Factor	Warning
1	Unknown	6	25.480	6571939	127497	93.145	96.044	N/A	6298	13.864	1.431	
2	Unknown	6	51.523	483665	5251	6.855	3.956	N/A	6891	N/A	1.333	

HPLC spectrum of **24**

(*S*)-*N*-(2-(Phenylsulfonyl)-1-(pyrimidin-5-yl)ethyl)benzamide (25)

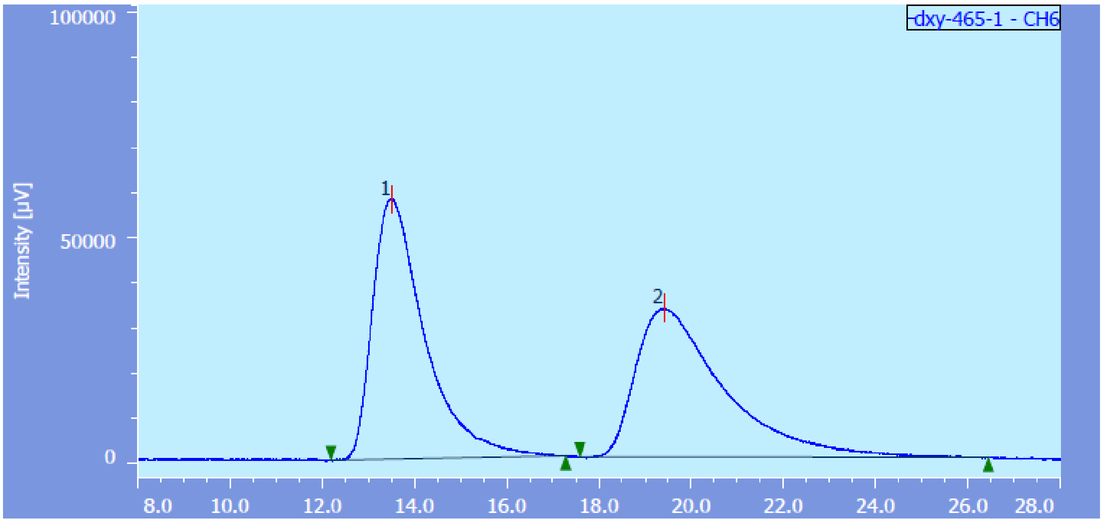

#	Peak Name	CH	tR [min]	Area [μV·sec]	Height [μV]	Area%	Height%	Quantity	NTP	Resolution	Symmetry Factor	Warning
1	Unknown	6	13.507	4520776	57581	50.982	63.617	N/A	787	2.309	1.853	
2	Unknown	6	19.407	4346622	32931	49.018	36.383	N/A	589	N/A	2.182	

HPLC spectrum of rac-**25**

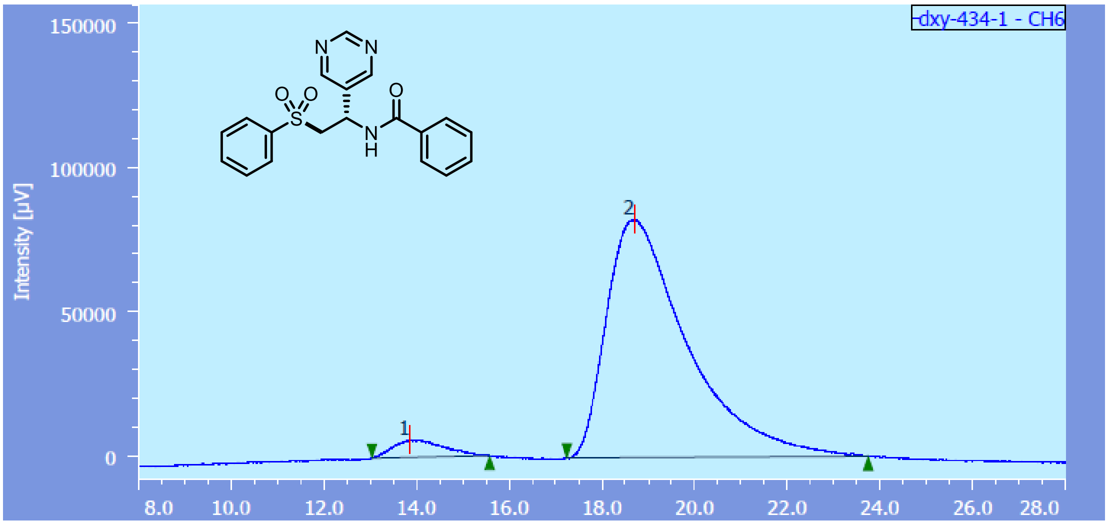

#	Peak Name	CH	tR [min]	Area [μV·sec]	Height [μV]	Area%	Height%	Quantity	NTP	Resolution	Symmetry Factor	Warning
1	Unknown	6	13.867	454649	6022	4.352	6.811	N/A	726	1.921	1.550	
2	Unknown	6	18.703	9993211	82392	95.648	93.189	N/A	626	N/A	1.982	

HPLC spectrum of **25**

(*S*)-*N*-(1-(Dibenzo[b,d]thiophen-2-yl)-2-(phenylsulfonyl)ethyl)benzamide (26)

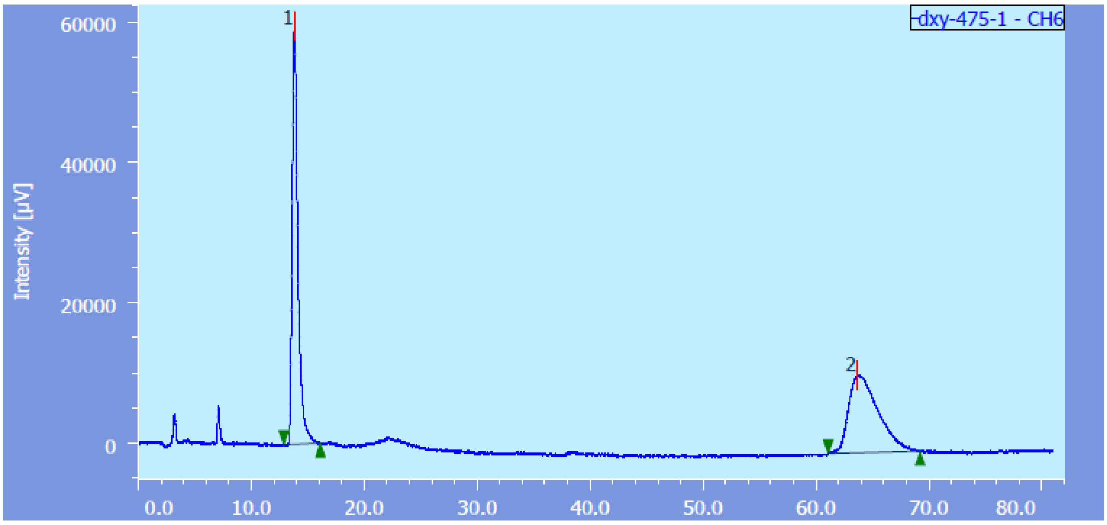

#	Peak Name	CH	tR [min]	Area [µV·sec]	Height [µV]	Area%	Height%	Quantity	NTP	Resolution	Symmetry Factor	Warning
1	Unknown	6	13.817	2038635	59380	50.120	84.289	N/A	4717	17.604	1.667	
2	Unknown	6	63.620	2028856	11068	49.880	15.711	N/A	2732	N/A	1.769	

HPLC spectrum of rac-**26**

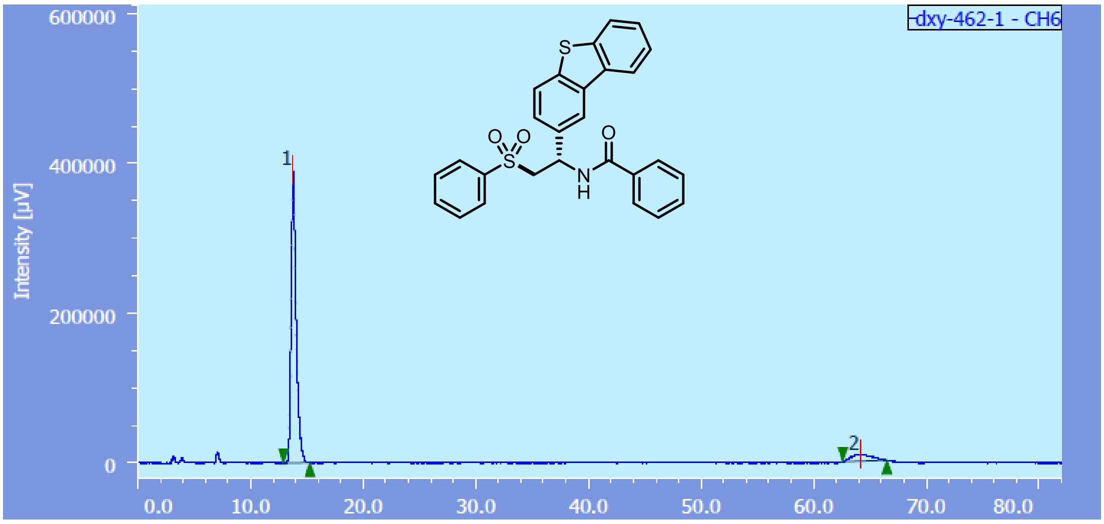

#	Peak Name	CH	tR [min]	Area [µV·sec]	Height [µV]	Area%	Height%	Quantity	NTP	Resolution	Symmetry Factor	Warning
1	Unknown	6	13.777	12557705	391163	91.806	97.862	N/A	4686	21.817	1.442	
2	Unknown	6	64.077	1120778	8546	8.194	2.138	N/A	4506	N/A	1.284	

HPLC spectrum of **26**

(*S*,*E*)-*N*-(3-Methyl-1-(phenylsulfonyl)pent-3-e*N*-2-yl)benzamide (27).

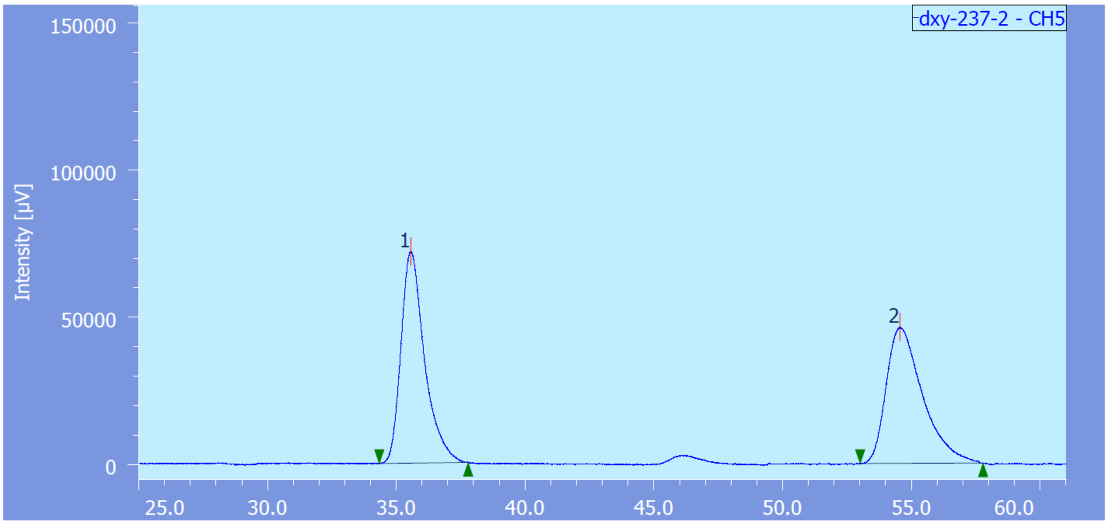

#	Peak Name	CH	tR [min]	Area [µV·sec]	Height [µV]	Area%	Height%	Quantity	NTP	Resolution	Symmetry Factor	Warning
1	Unknown	5	35.567	4580965	71906	50.171	60.853	N/A	8071	9.344	1.413	
2	Unknown	5	54.553	4549728	46257	49.829	39.147	N/A	7673	N/A	1.585	

HPLC spectrum of rac-**27**

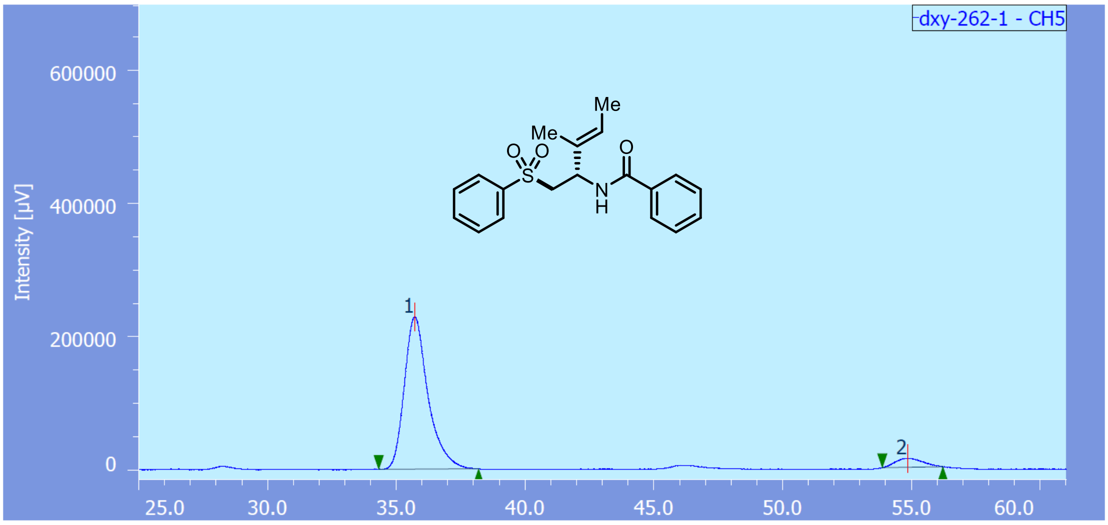

#	Peak Name	CH	tR [min]	Area [µV·sec]	Height [µV]	Area%	Height%	Quantity	NTP	Resolution	Symmetry Factor	Warning
1	Unknown	5	35.713	14619317	228447	93.656	94.432	N/A	8109	10.533	1.362	
2	Unknown	5	54.853	990227	13470	6.344	5.568	N/A	11372	N/A	1.186	

HPLC spectrum of **27**

(*S*)-*N*-(4-Methyl-1-(phenylsulfonyl)pent-3-en-2-yl)benzamide (28)

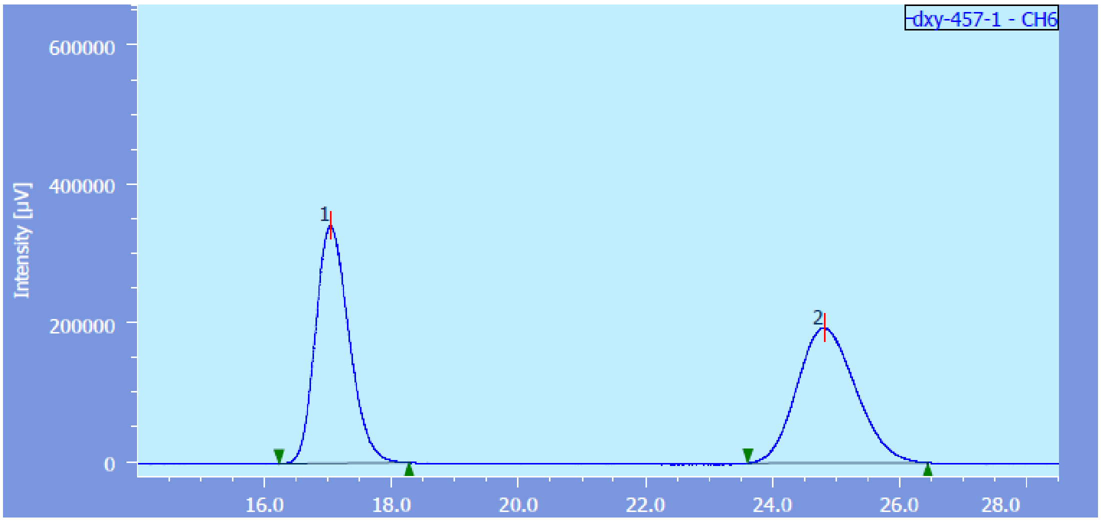

#	Peak Name	CH	tR [min]	Area [µV·sec]	Height [µV]	Area%	Height%	Quantity	NTP	Resolution	Symmetry Factor	Warning
1	Unknown	6	17.040	12503320	341471	50.010	63.741	N/A	5003	5.811	1.221	
2	Unknown	6	24.810	12498304	194248	49.990	36.259	N/A	3339	N/A	1.142	

HPLC spectrum of rac-**28**

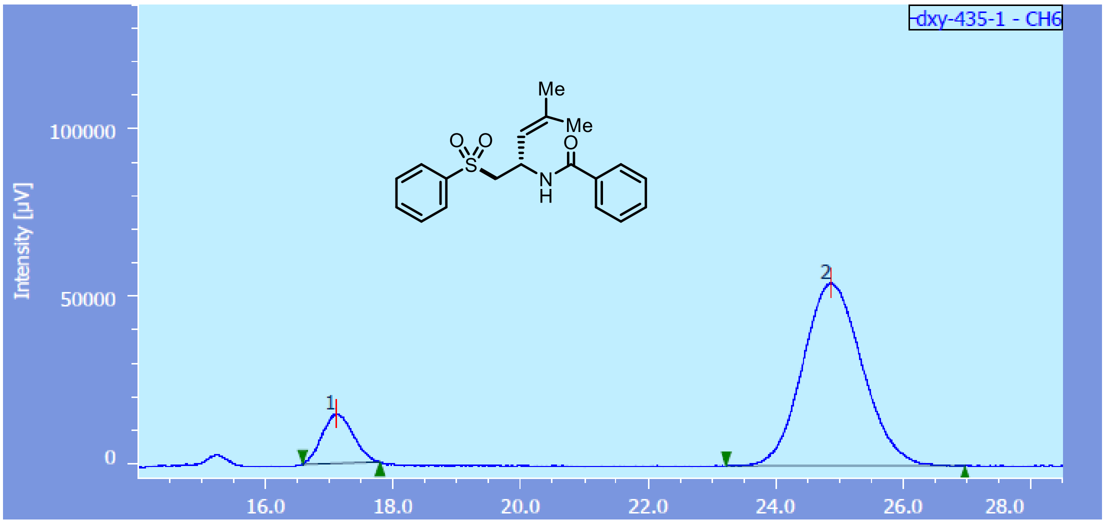

#	Peak Name	CH	tR [min]	Area [µV·sec]	Height [µV]	Area%	Height%	Quantity	NTP	Resolution	Symmetry Factor	Warning
1	Unknown	6	17.113	496660	14631	12.290	21.194	N/A	5414	5.873	1.115	
2	Unknown	6	24.873	3544665	54403	87.710	78.806	N/A	3349	N/A	1.089	

HPLC spectrum of **28**

(*S*)-*N*-(1-(1H-Inde*N*-2-yl)-2-(phenylsulfonyl)ethyl)benzamide (29).

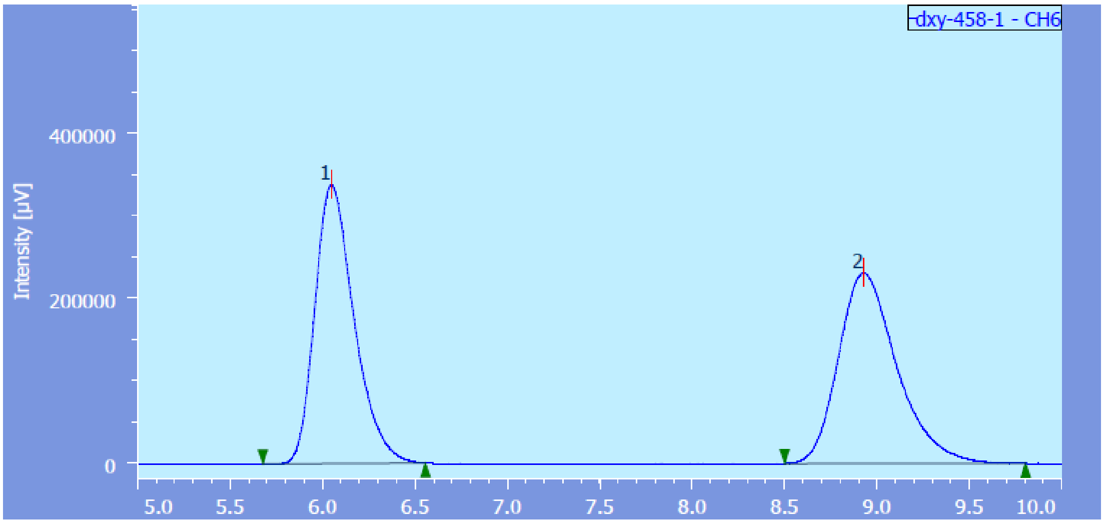

#	Peak Name	CH	tR [min]	Area [µV·sec]	Height [µV]	Area%	Height%	Quantity	NTP	Resolution	Symmetry Factor	Warning
1	Unknown	6	6.047	4996230	338387	49.560	59.462	N/A	4043	6.119	1.344	
2	Unknown	6	8.927	5085040	230694	50.440	40.538	N/A	4016	N/A	1.325	

HPLC spectrum of rac-**29**

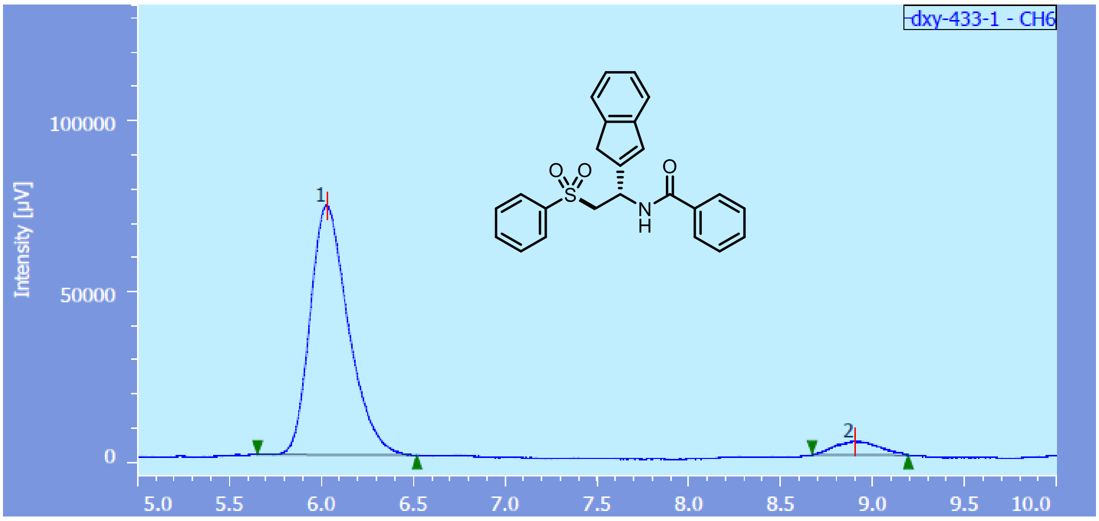

#	Peak Name	CH	tR [min]	Area [µV·sec]	Height [µV]	Area%	Height%	Quantity	NTP	Resolution	Symmetry Factor	Warning
1	Unknown	6	6.030	1052292	72737	93.995	94.854	N/A	4165	6.741	1.299	
2	Unknown	6	8.900	67231	3946	6.005	5.146	N/A	5501	N/A	1.129	

HPLC spectrum of **29**

(*S*)-*N*-(1-(4-Cyanophenyl)-2-(phenylsulfonyl)ethyl)-4-methoxybenzamide (30).

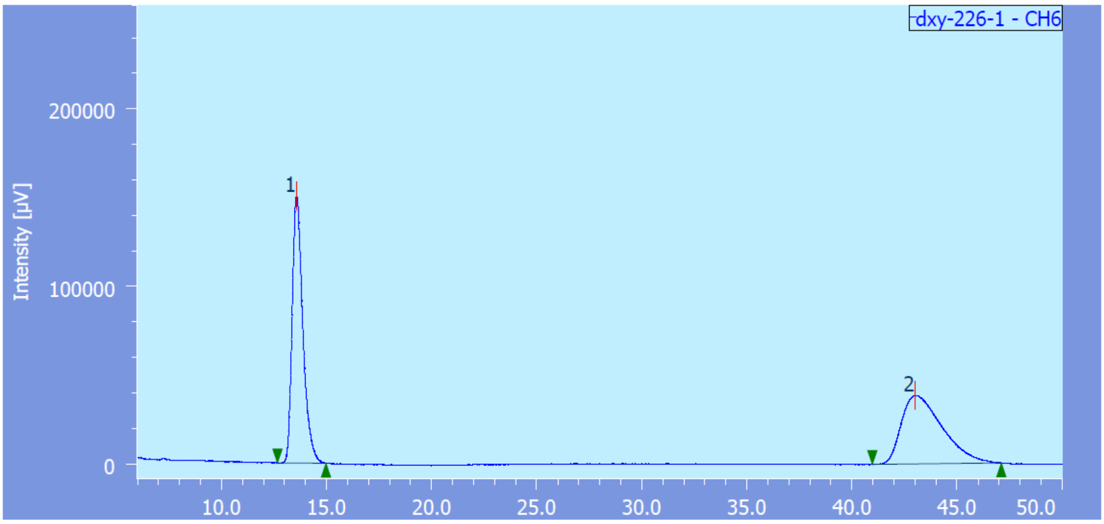

#	Peak Name	CH	tR [min]	Area [µV·sec]	Height [µV]	Area%	Height%	Quantity	NTP	Resolution	Symmetry Factor	Warning
1	Unknown	6	13.570	5309609	150331	50.380	79.591	N/A	3685	13.164	1.425	
2	Unknown	6	42.997	5229548	38549	49.620	20.409	N/A	2297	N/A	1.702	

HPLC spectrum of rac-**30**

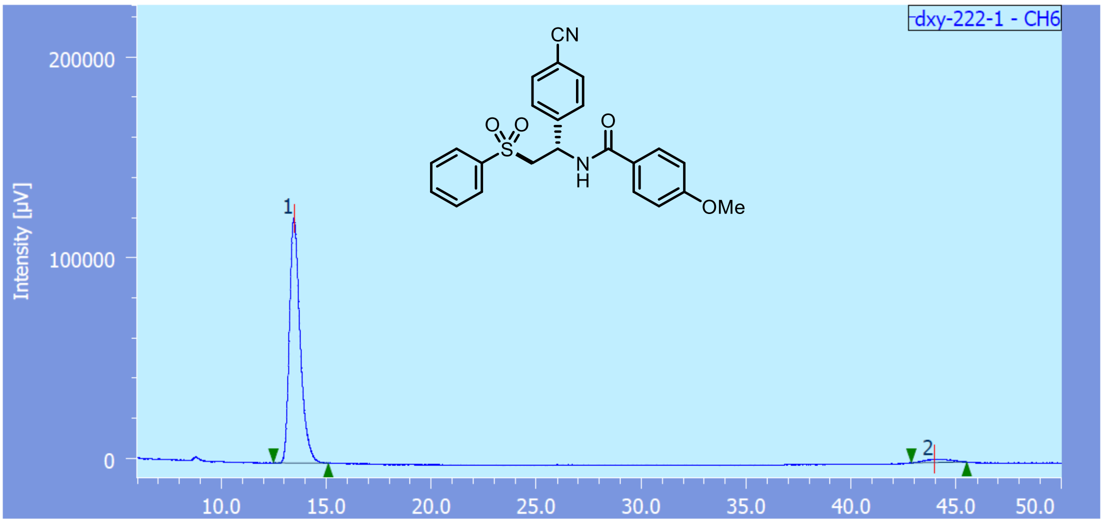

#	Peak Name	CH	tR [min]	Area [µV·sec]	Height [µV]	Area%	Height%	Quantity	NTP	Resolution	Symmetry Factor	Warning
1	Unknown	6	13.457	4339923	122027	96.812	98.662	N/A	3574	17.862	1.395	
2	Unknown	6	43.940	142896	1654	3.188	1.338	N/A	4857	N/A	1.261	

HPLC spectrum of **30**

(*S*)-4-Methoxy-*N*-(1-(3-methoxyphenyl)-2-(phenylsulfonyl)ethyl)benzamide (31).

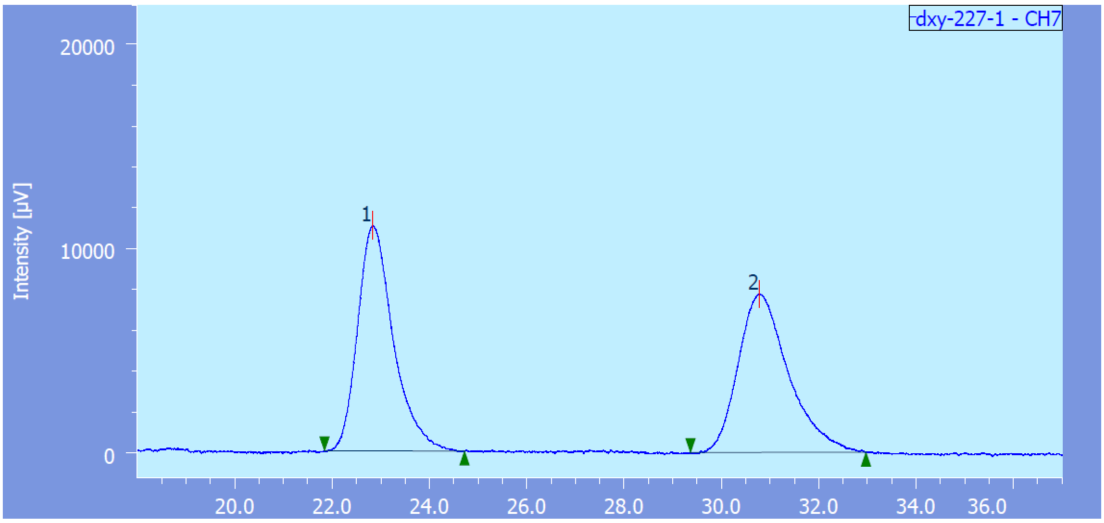

#	Peak Name	CH	tR [min]	Area [µV·sec]	Height [µV]	Area%	Height%	Quantity	NTP	Resolution	Symmetry Factor	Warning
1	Unknown	7	16.637	3907085	100411	50.240	65.262	N/A	4667	9.543	1.401	
2	Unknown	7	30.003	3869746	53446	49.760	34.738	N/A	4279	N/A	1.477	

HPLC spectrum of rac-**31**

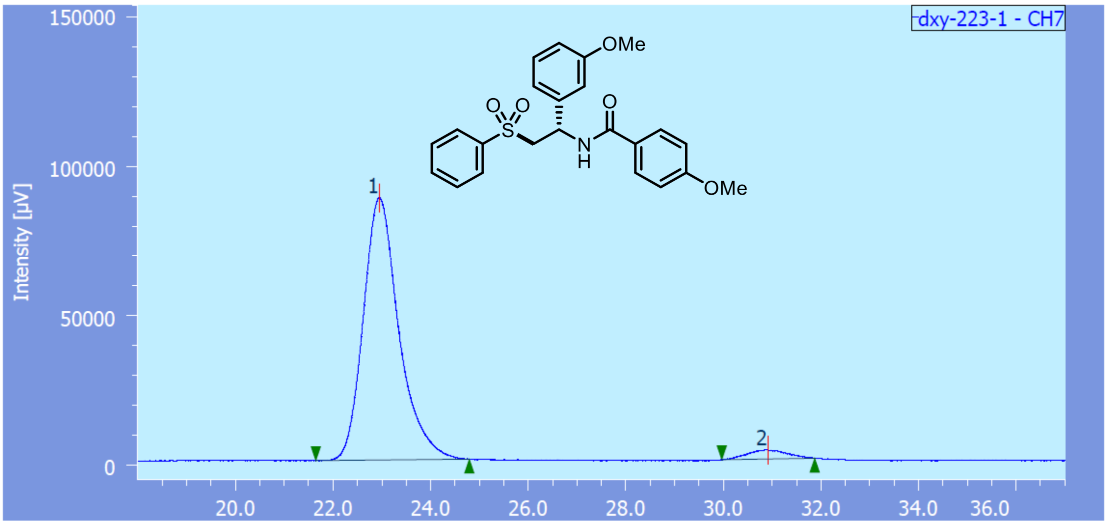

#	Peak Name	CH	tR [min]	Area [µV·sec]	Height [µV]	Area%	Height%	Quantity	NTP	Resolution	Symmetry Factor	Warning
1	Unknown	7	22.947	4452486	87812	96.259	96.657	N/A	5243	5.596	1.291	
2	Unknown	7	30.907	173032	3037	3.741	3.343	N/A	6083	N/A	1.029	

HPLC spectrum of **31**

(*S*)-*N*-(1-(4-Cyanophenyl)-2-(phenylsulfonyl)ethyl)-4-methylbenzamide (32).

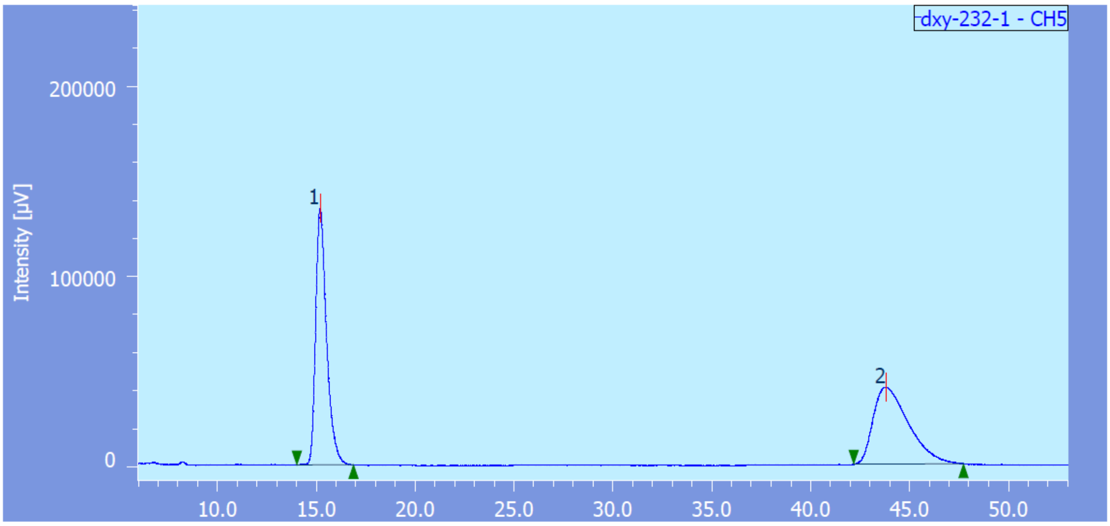

#	Peak Name	CH	tR [min]	Area [µV·sec]	Height [µV]	Area%	Height%	Quantity	NTP	Resolution	Symmetry Factor	Warning
1	Unknown	5	15.193	5169106	134853	50.742	76.974	N/A	3968	13.551	1.433	
2	Unknown	5	43.817	5018028	40341	49.258	23.026	N/A	2871	N/A	1.622	

HPLC spectrum of rac-**32**

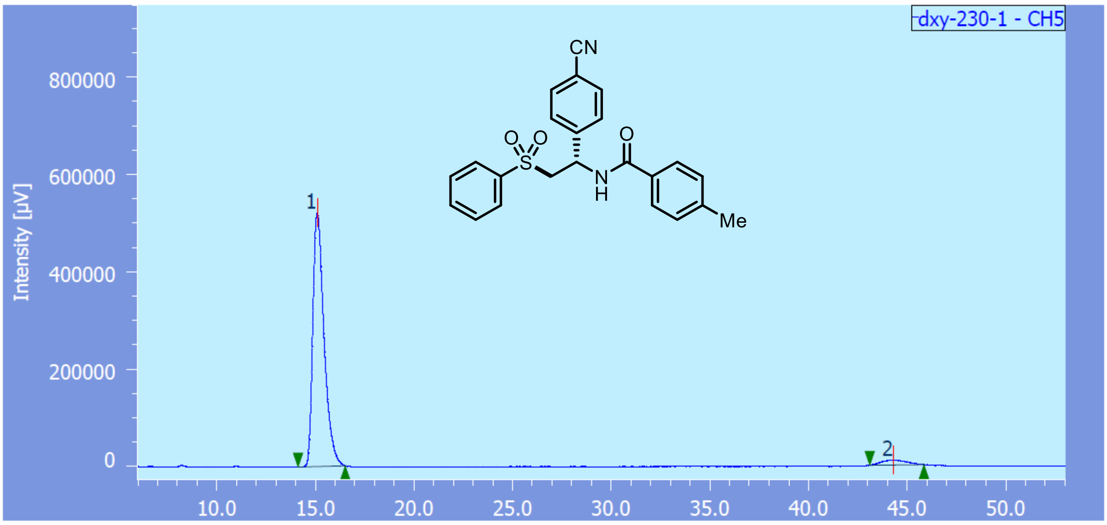

#	Peak Name	CH	tR [min]	Area [µV·sec]	Height [µV]	Area%	Height%	Quantity	NTP	Resolution	Symmetry Factor	Warning
1	Unknown	5	15.090	20577640	521480	95.837	98.137	N/A	3562	16.311	1.528	
2	Unknown	5	44.303	893765	9902	4.163	1.863	N/A	4717	N/A	1.136	

HPLC spectrum of **32**

(*S*)-*N*-(1-(4-Cyanophenyl)-2-(phenylsulfonyl)ethyl)-4-fluorobenzamide (33).

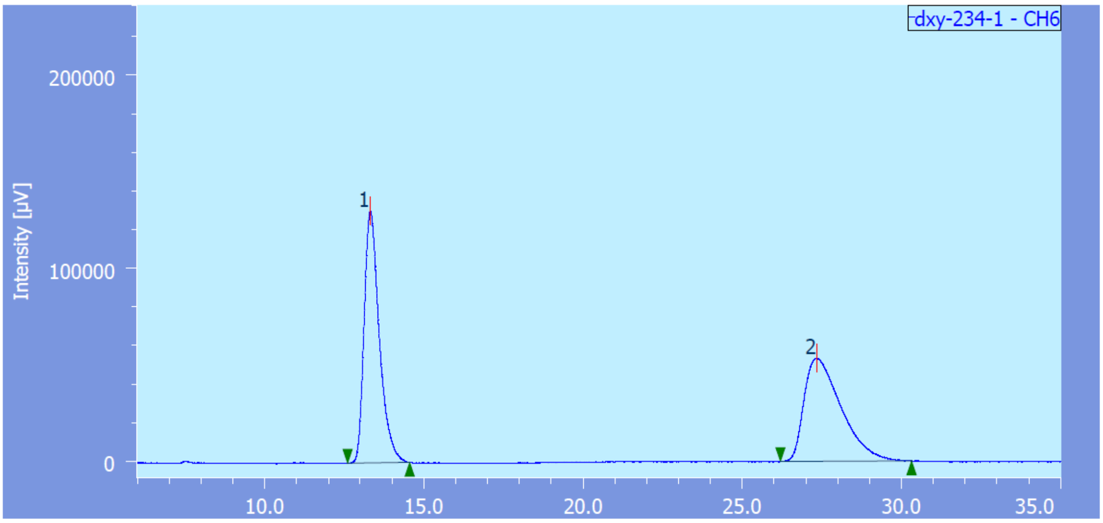

#	Peak Name	CH	tR [min]	Area [μV·sec]	Height [μV]	Area%	Height%	Quantity	NTP	Resolution	Symmetry Factor	Warning
1	Unknown	6	13.313	4312941	130340	49.964	70.972	N/A	4046	9.493	1.457	
2	Unknown	6	27.327	4319127	53311	50.036	29.028	N/A	2651	N/A	1.798	

HPLC spectrum of rac-**33**

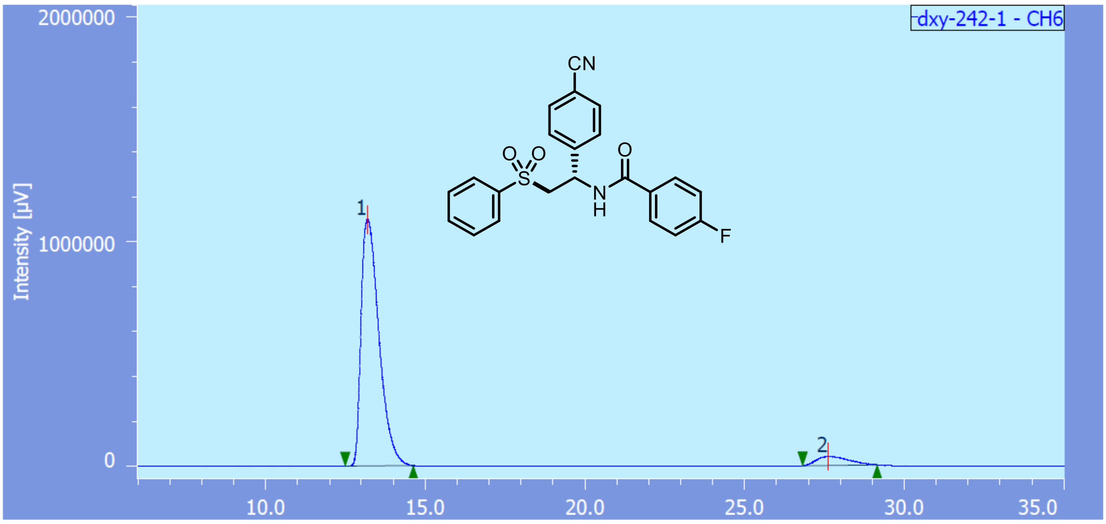

#	Peak Name	CH	tR [min]	Area [μV·sec]	Height [μV]	Area%	Height%	Quantity	NTP	Resolution	Symmetry Factor	Warning
1	Unknown	6	13.193	42608039	1098000	93.818	96.484	N/A	2690	9.762	1.694	
2	Unknown	6	27.617	2807477	40011	6.182	3.516	N/A	3225	N/A	1.466	

HPLC spectrum of **33**

(*S*)-4-Chloro-*N*-(1-(4-cyanophenyl)-2-(phenylsulfonyl)ethyl)benzamide (34).

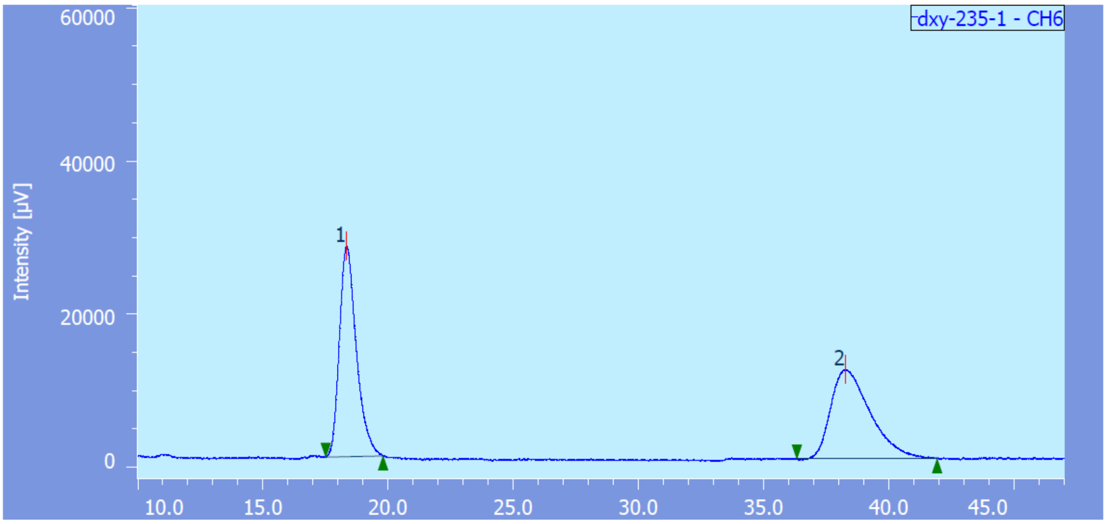

#	Peak Name	CH	tR [min]	Area [µV·sec]	Height [µV]	Area%	Height%	Quantity	NTP	Resolution	Symmetry Factor	Warning
1	Unknown	6	18.340	1292668	27499	50.106	70.277	N/A	3757	9.842	1.360	
2	Unknown	6	38.260	1287222	11630	49.894	29.723	N/A	2859	N/A	1.584	

HPLC spectrum of rac-**34**

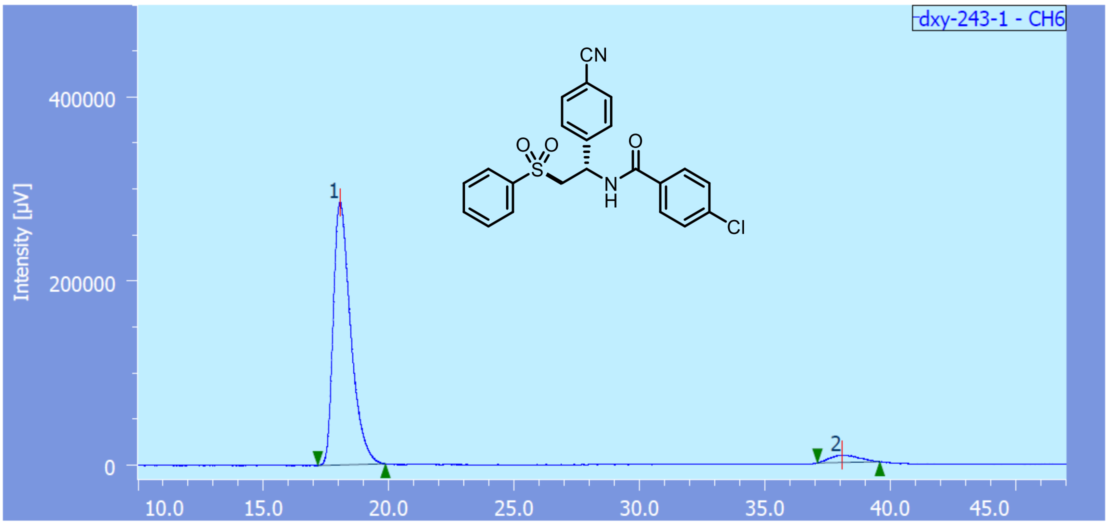

#	Peak Name	CH	tR [min]	Area [µV·sec]	Height [µV]	Area%	Height%	Quantity	NTP	Resolution	Symmetry Factor	Warning
1	Unknown	6	18.053	13809482	285274	95.567	97.313	N/A	3388	11.201	1.551	
2	Unknown	6	38.067	640640	7878	4.433	2.687	N/A	4225	N/A	1.260	

HPLC spectrum of **34**

(*S*)-*N*-(1-(4-Cyanophenyl)-2-(phenylsulfonyl)ethyl)-4-(trifluoromethyl)benzamide (35).

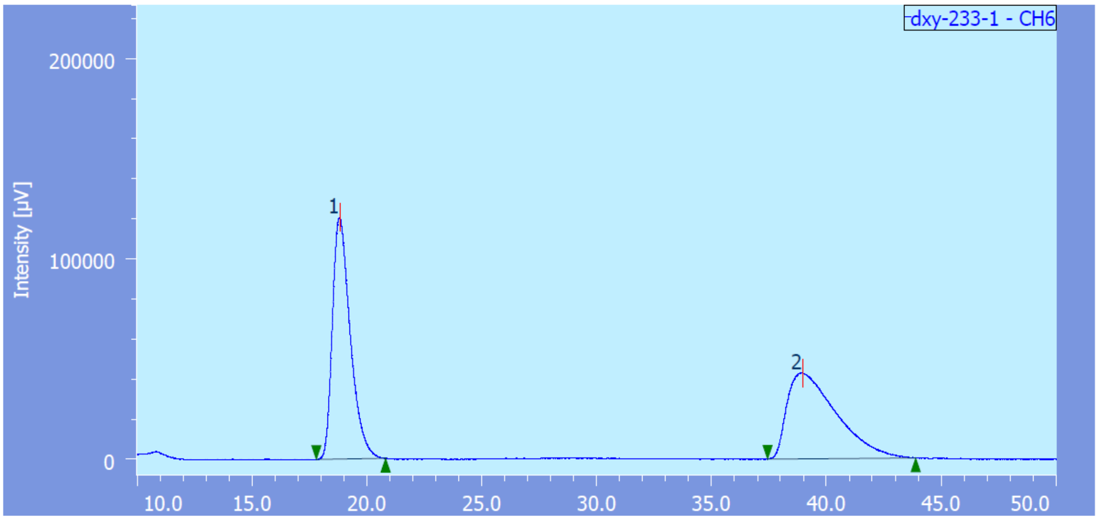

#	Peak Name	CH	tR [min]	Area [µV·sec]	Height [µV]	Area%	Height%	Quantity	NTP	Resolution	Symmetry Factor	Warning
1	Unknown	6	18.813	6520248	120580	50.430	73.826	N/A	2935	7.540	1.466	
2	Unknown	6	38.963	6409180	42749	49.570	26.174	N/A	1541	N/A	2.086	

HPLC spectrum of rac-**35**

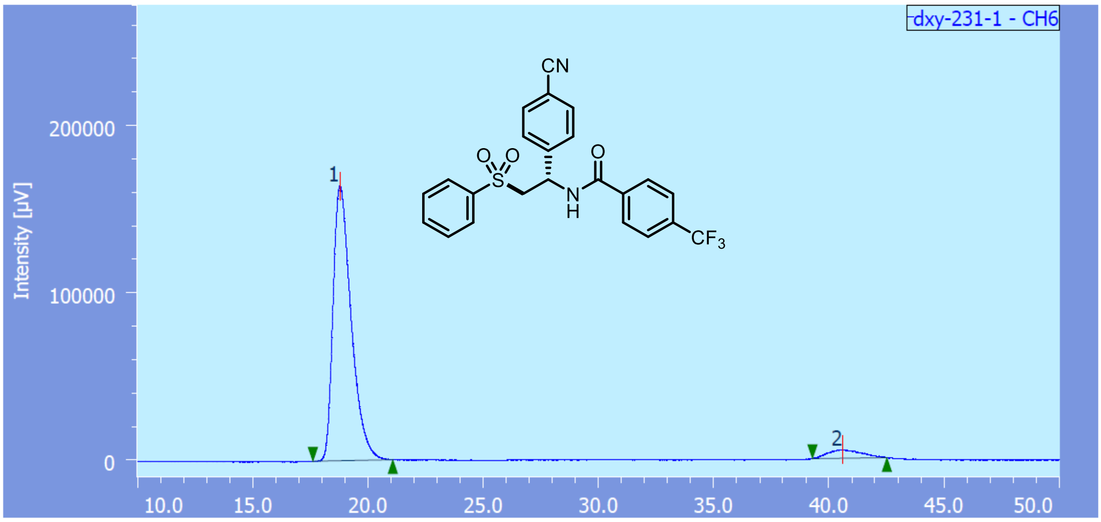

#	Peak Name	CH	tR [min]	Area [µV·sec]	Height [µV]	Area%	Height%	Quantity	NTP	Resolution	Symmetry Factor	Warning
1	Unknown	6	18.783	8978060	164107	94.615	97.064	N/A	2856	10.047	1.556	
2	Unknown	6	40.607	511022	4964	5.385	2.936	N/A	3032	N/A	1.227	

HPLC spectrum of **35**

(*S*)-4-Cyano-*N*-(1-(4-cyanophenyl)-2-(phenylsulfonyl)ethyl)benzamide (36).

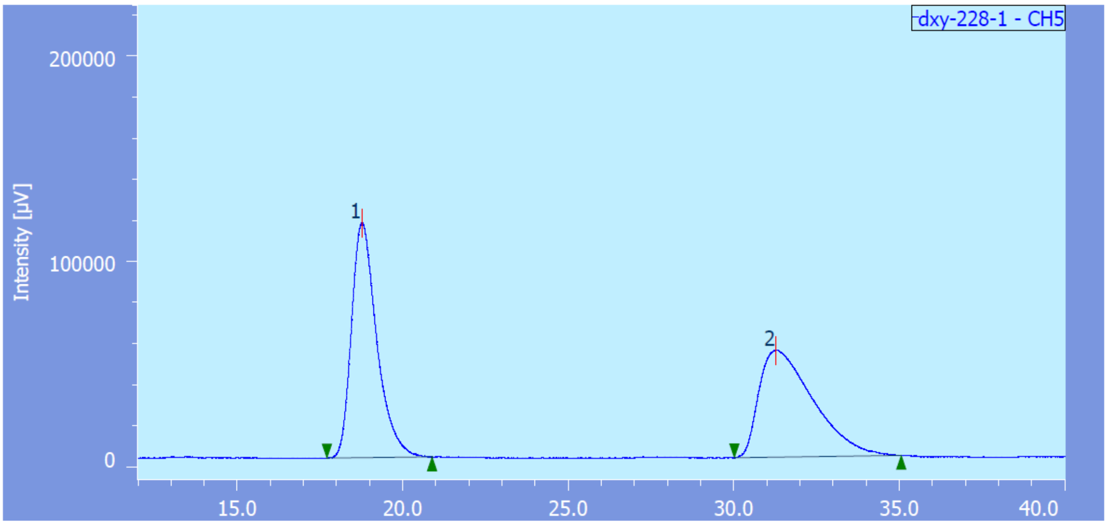

#	Peak Name	CH	tR [min]	Area [µV·sec]	Height [µV]	Area%	Height%	Quantity	NTP	Resolution	Symmetry Factor	Warning
1	Unknown	5	18.767	6073036	114329	50.579	68.720	N/A	3121	5.745	1.406	
2	Unknown	5	31.260	5933897	52040	49.421	31.280	N/A	1718	N/A	1.973	

HPLC spectrum of rac-**36**

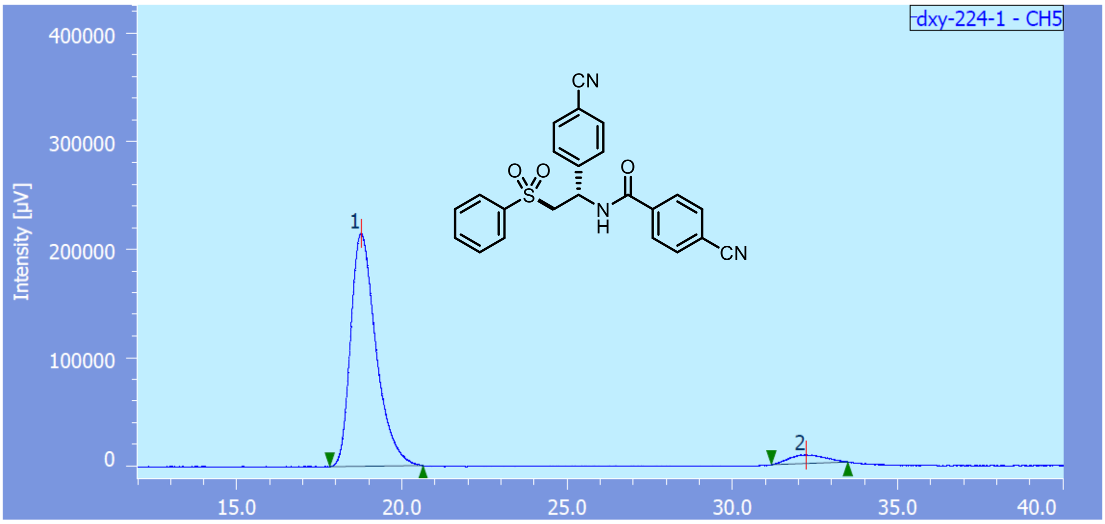

#	Peak Name	CH	tR [min]	Area [µV·sec]	Height [µV]	Area%	Height%	Quantity	NTP	Resolution	Symmetry Factor	Warning
1	Unknown	5	18.760	11447887	215286	94.927	96.406	N/A	3054	7.651	1.430	
2	Unknown	5	32.220	611796	8025	5.073	3.594	N/A	3528	N/A	1.115	

HPLC spectrum of **36**

(*S*)-4-Cyano-*N*-(1-(3-methoxyphenyl)-2-(phenylsulfonyl)ethyl)benzamide (37).

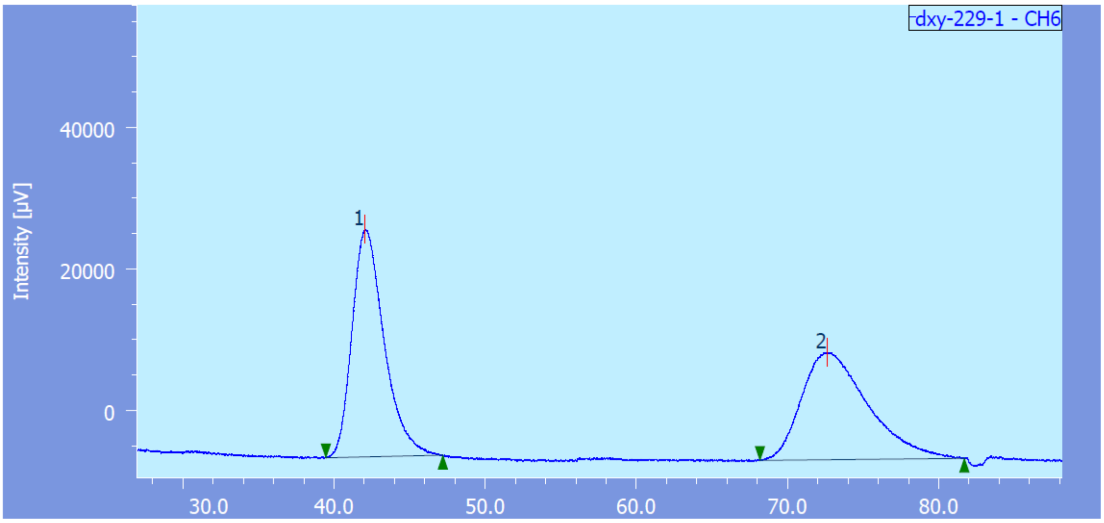

#	Peak Name	CH	tR [min]	Area [μV·sec]	Height [μV]	Area%	Height%	Quantity	NTP	Resolution	Symmetry Factor	Warning
1	Unknown	6	42.057	4740663	32173	50.681	67.988	N/A	2012	5.245	1.391	
2	Unknown	6	72.617	4613232	15148	49.319	32.012	N/A	1341	N/A	1.523	

HPLC spectrum of rac-**37**

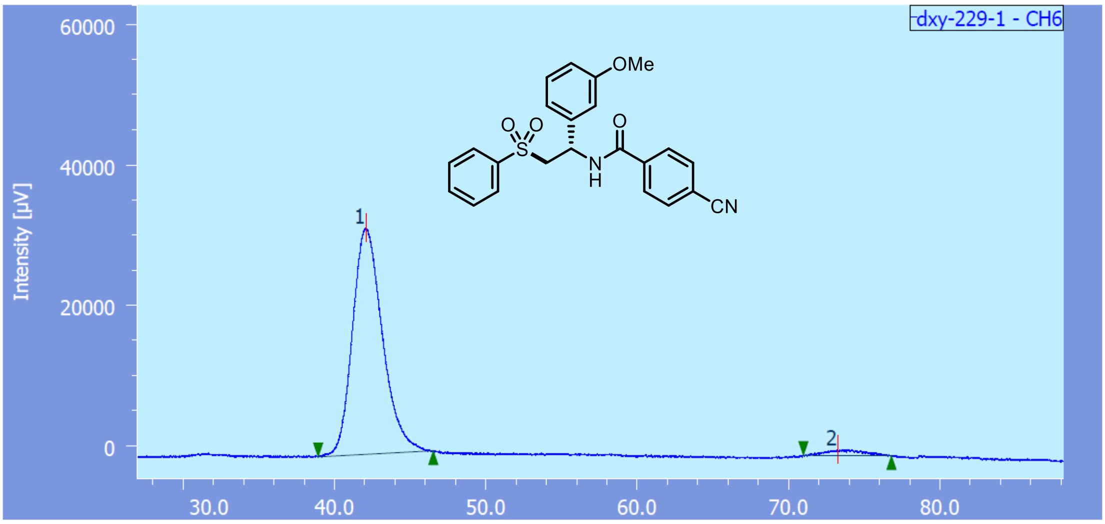

#	Peak Name	CH	tR [min]	Area [μV·sec]	Height [μV]	Area%	Height%	Quantity	NTP	Resolution	Symmetry Factor	Warning
1	Unknown	6	42.063	4430528	32252	96.804	97.469	N/A	2259	6.507	1.256	
2	Unknown	6	73.220	146271	838	3.196	2.531	N/A	2334	N/A	1.298	

HPLC spectrum of **37**

(*S*)-3-Chloro-*N*-(1-(4-cyanophenyl)-2-(phenylsulfonyl)ethyl)benzamide (38).

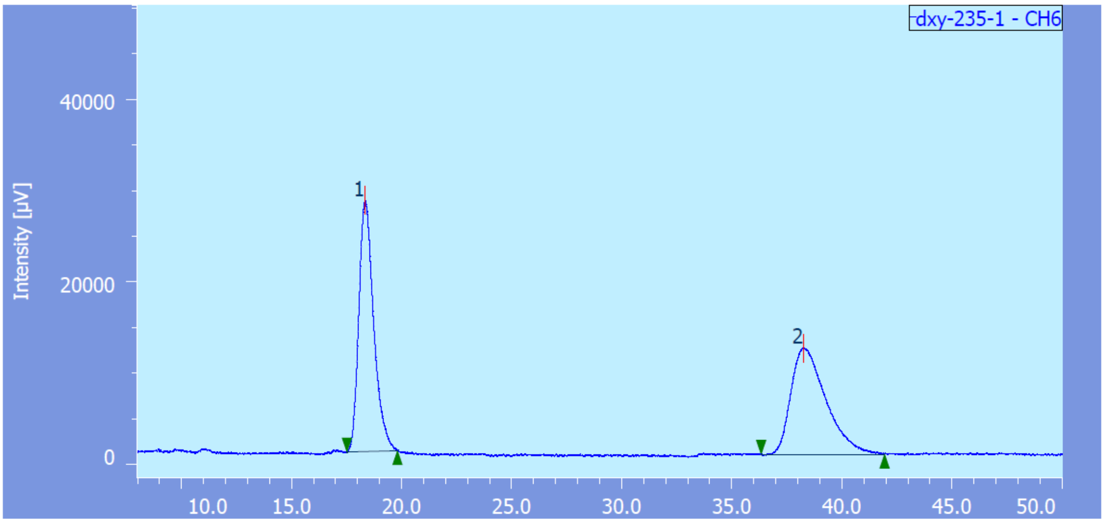

#	Peak Name	CH	tR [min]	Area [µV·sec]	Height [µV]	Area%	Height%	Quantity	NTP	Resolution	Symmetry Factor	Warning
1	Unknown	6	18.340	1292668	27499	50.106	70.277	N/A	3757	9.842	1.360	
2	Unknown	6	38.260	1287222	11630	49.894	29.723	N/A	2859	N/A	1.584	

HPLC spectrum of rac-**38**

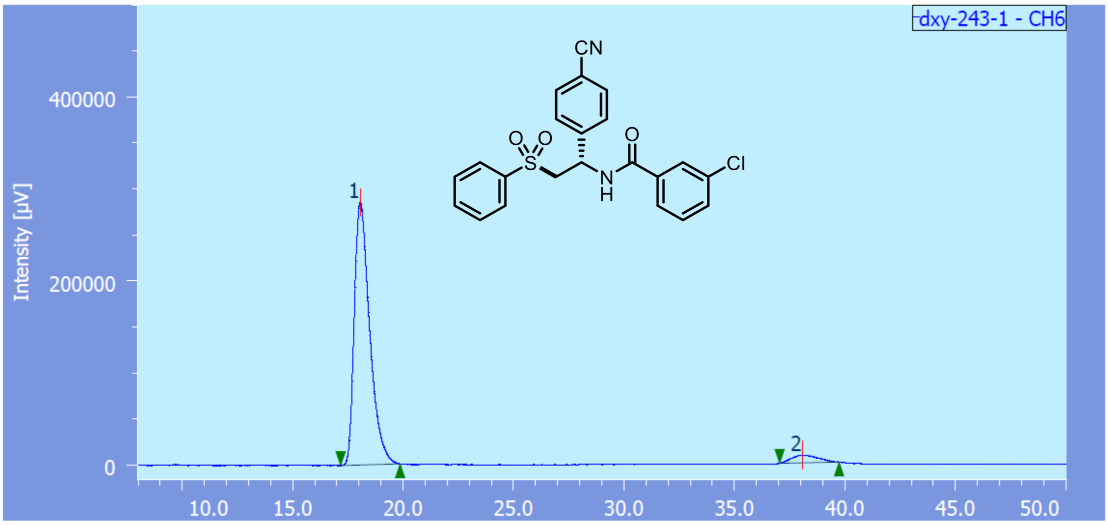

#	Peak Name	CH	tR [min]	Area [µV·sec]	Height [µV]	Area%	Height%	Quantity	NTP	Resolution	Symmetry Factor	Warning
1	Unknown	6	18.053	13809482	285274	95.167	97.192	N/A	3388	10.956	1.551	
2	Unknown	6	38.067	701303	8241	4.833	2.808	N/A	3950	N/A	1.302	

HPLC spectrum of **38**

(*S*)-*N*-(1-(4-Cyanophenyl)-2-(phenylsulfonyl)ethyl)-2-fluorobenzamide (39).

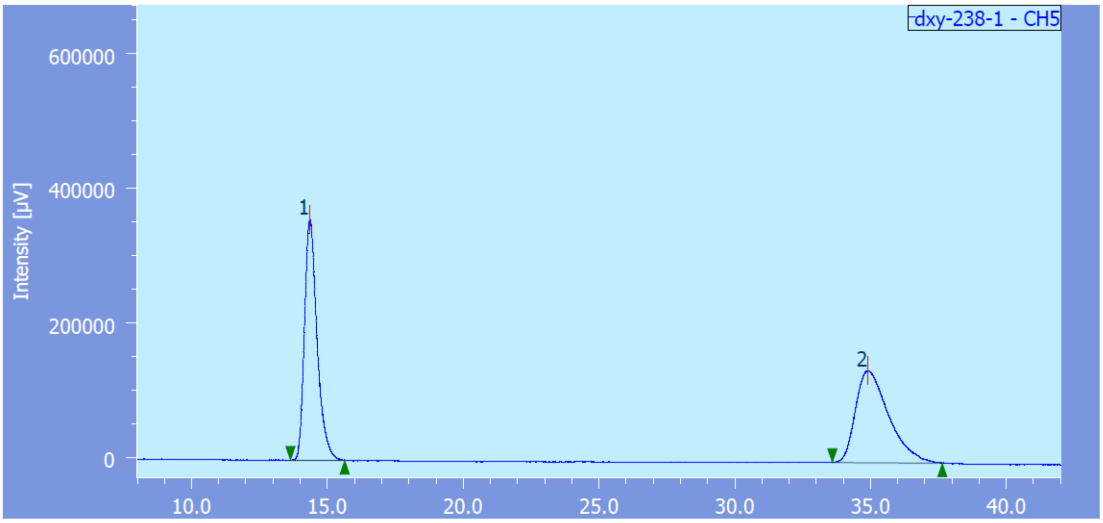

#	Peak Name	CH	tR [min]	Area [μV·sec]	Height [μV]	Area%	Height%	Quantity	NTP	Resolution	Symmetry Factor	Warning
1	Unknown	5	14.353	11501056	357757	49.753	72.371	N/A	5013	13.693	1.370	
2	Unknown	5	34.900	11615197	136580	50.247	27.629	N/A	4033	N/A	1.544	

HPLC spectrum of rac-**39**

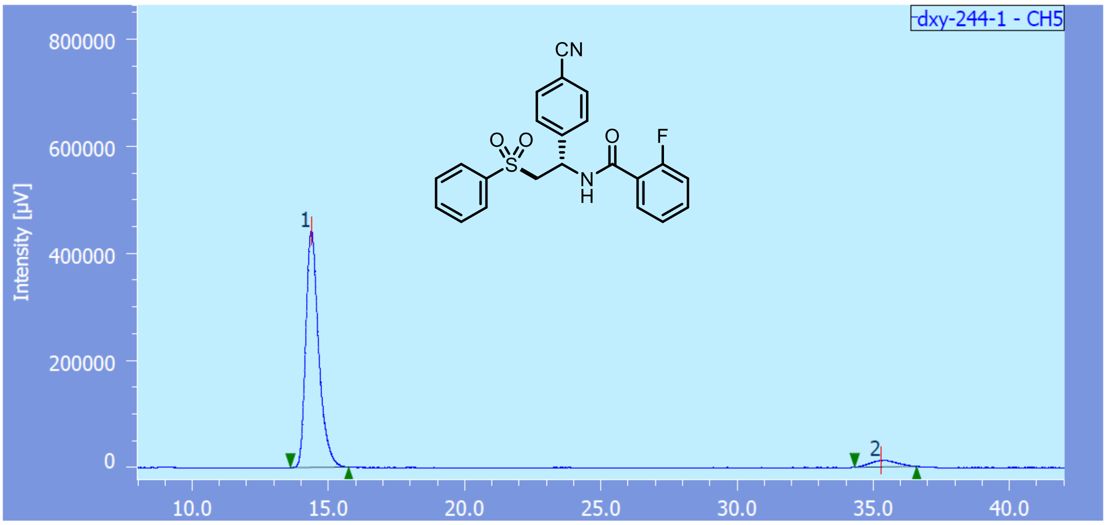

#	Peak Name	CH	tR [min]	Area [μV·sec]	Height [μV]	Area%	Height%	Quantity	NTP	Resolution	Symmetry Factor	Warning
1	Unknown	5	14.370	14542262	441586	94.642	97.367	N/A	4807	15.403	1.391	
2	Unknown	5	35.297	823337	11943	5.358	2.633	N/A	5548	N/A	1.181	

HPLC spectrum of **39**

(*S*)-2-Chloro-*N*-(1-(4-cyanophenyl)-2-(phenylsulfonyl)ethyl)benzamide (40).

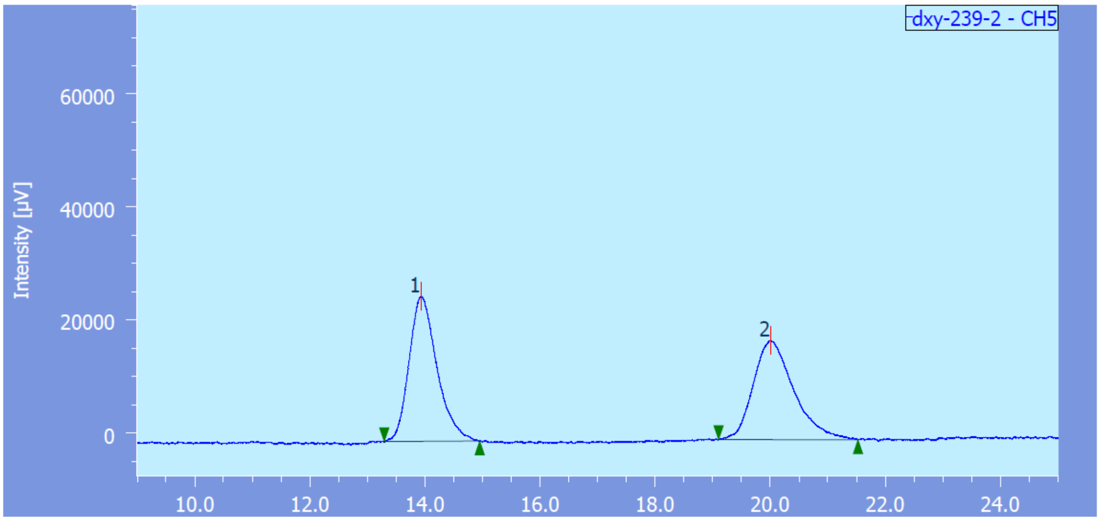

#	Peak Name	CH	tR [min]	Area [µV·sec]	Height [µV]	Area%	Height%	Quantity	NTP	Resolution	Symmetry Factor	Warning
1	Unknown	5	13.937	863387	25663	50.183	59.493	N/A	4192	5.790	1.281	
2	Unknown	5	20.007	857088	17473	49.817	40.507	N/A	4156	N/A	1.340	

HPLC spectrum of rac-**40**

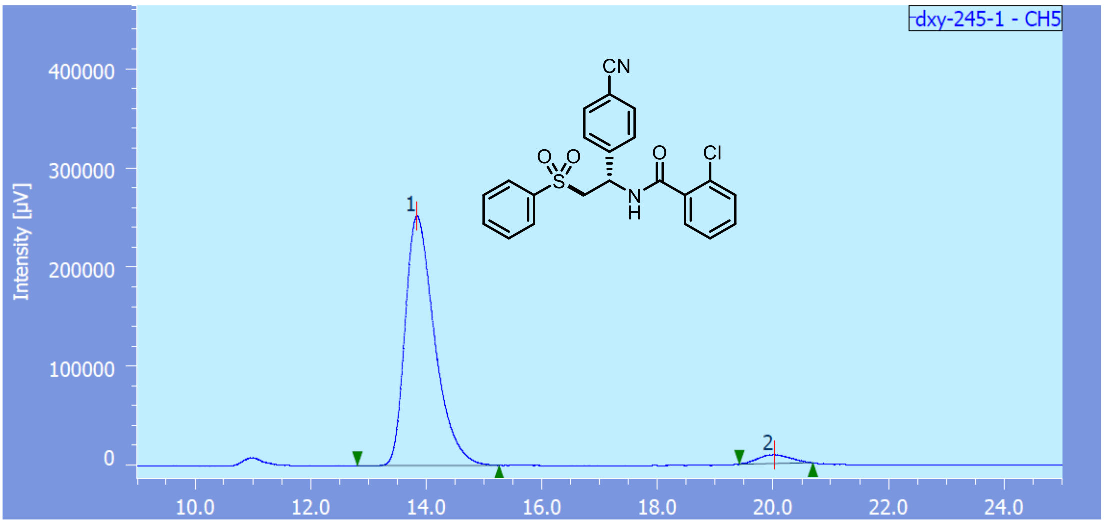

#	Peak Name	CH	tR [min]	Area [µV·sec]	Height [µV]	Area%	Height%	Quantity	NTP	Resolution	Symmetry Factor	Warning
1	Unknown	5	13.840	8915731	252047	96.185	96.547	N/A	3722	6.171	1.456	
2	Unknown	5	20.017	353579	9015	3.815	3.453	N/A	5299	N/A	1.057	

HPLC spectrum of **40**

(*S*)-*N*-(1-(4-Cyanophenyl)-2-(phenylsulfonyl)ethyl)-2-naphthamide (41).

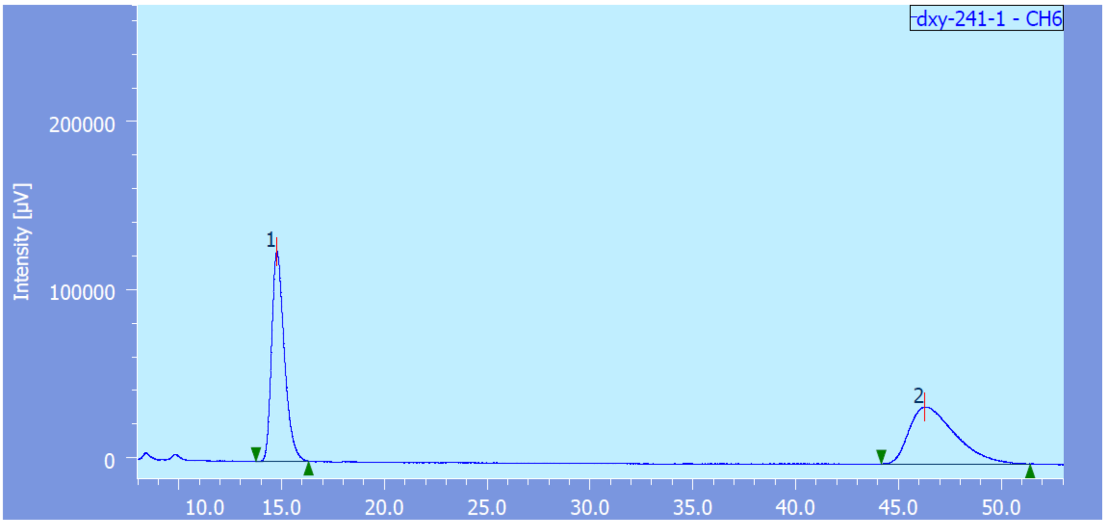

#	Peak Name	CH	tR [min]	Area [µV·sec]	Height [µV]	Area%	Height%	Quantity	NTP	Resolution	Symmetry Factor	Warning
1	Unknown	6	14.770	5378628	124912	50.337	78.612	N/A	2866	12.174	1.382	
2	Unknown	6	46.267	5306573	33984	49.663	21.388	N/A	2053	N/A	1.681	

HPLC spectrum of rac-**41**

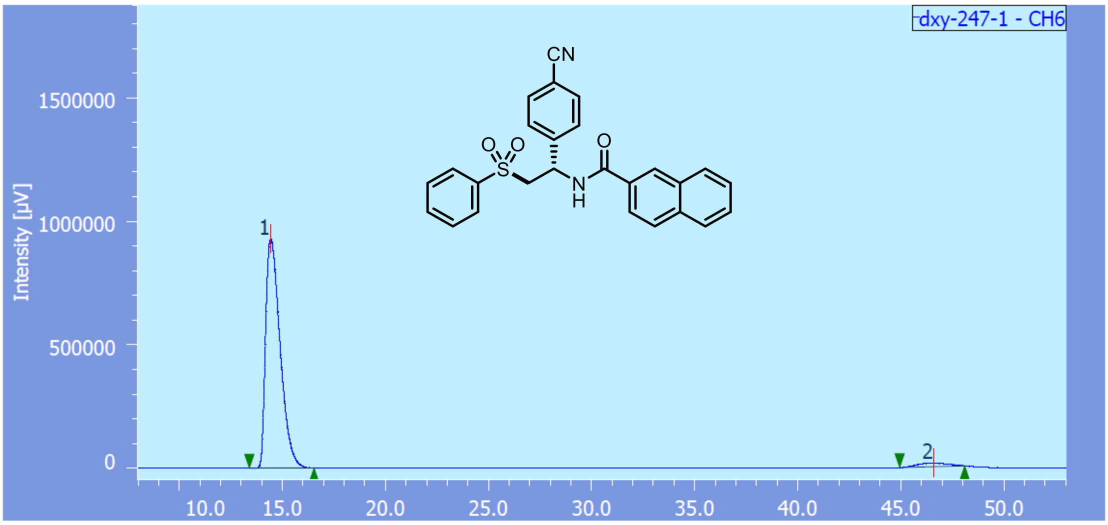

#	Peak Name	CH	tR [min]	Area [µV·sec]	Height [µV]	Area%	Height%	Quantity	NTP	Resolution	Symmetry Factor	Warning
1	Unknown	6	14.437	45309137	928220	96.611	98.418	N/A	2018	14.882	1.727	
2	Unknown	6	46.580	1589220	14923	3.389	1.582	N/A	3742	N/A	0.971	

HPLC spectrum of **41**

(*S*)-5-Chloro-*N*-(1-(4-cyanophenyl)-2-(phenylsulfonyl)ethyl)nicotinamide (42)

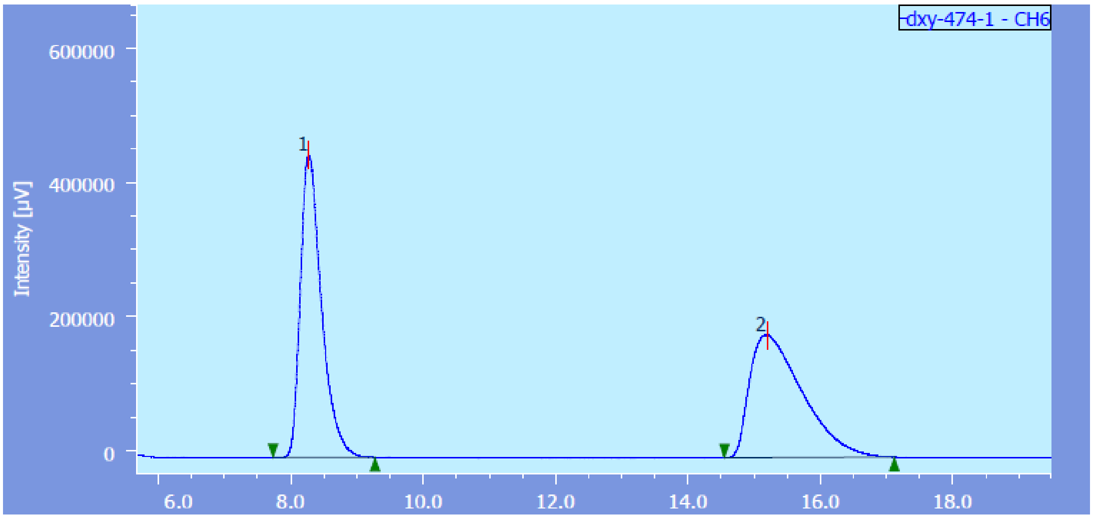

#	Peak Name	CH	tR [min]	Area [μV·sec]	Height [μV]	Area%	Height%	Quantity	NTP	Resolution	Symmetry Factor	Warning
1	Unknown	6	8.273	9970528	451087	49.528	71.181	N/A	3497	6.805	1.494	
2	Unknown	6	15.190	10160423	182634	50.472	28.819	N/A	1689	N/A	1.957	

HPLC spectrum of rac-**42**

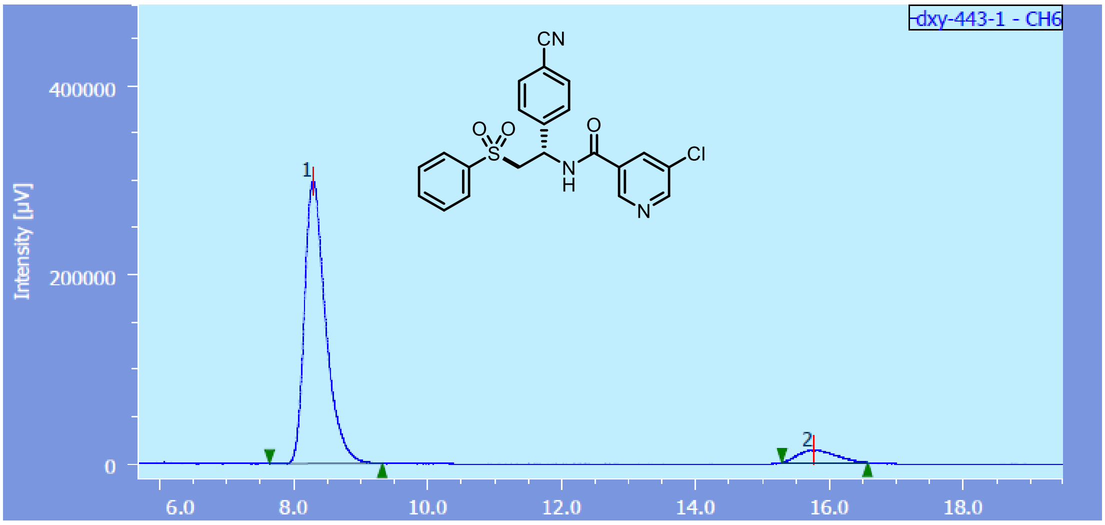

#	Peak Name	CH	tR [min]	Area [μV·sec]	Height [μV]	Area%	Height%	Quantity	NTP	Resolution	Symmetry Factor	Warning
1	Unknown	6	8.287	6557188	298820	92.886	95.790	N/A	3591	9.254	1.475	
2	Unknown	6	15.763	502206	13133	7.114	4.210	N/A	3491	N/A	1.313	

HPLC spectrum of **42**

(*S*)-*N*-(1-(4-Cyanophenyl)-2-(phenylsulfonyl)ethyl)pivalamide (43).

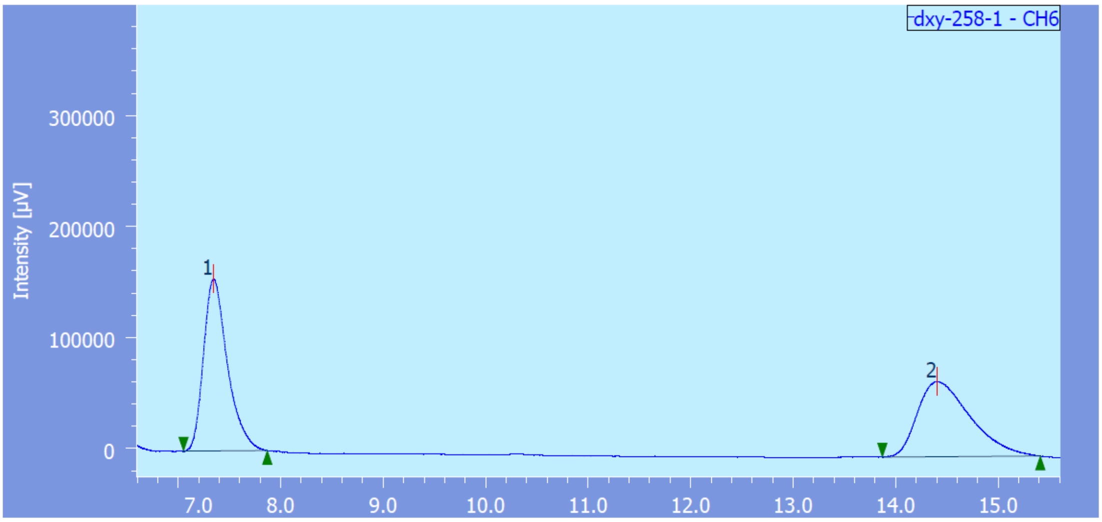

#	Peak Name	CH	tR [min]	Area [µV·sec]	Height [µV]	Area%	Height%	Quantity	NTP	Resolution	Symmetry Factor	Warning
1	Unknown	6	7.347	2518218	154782	50.875	69.588	N/A	5072	10.426	1.371	
2	Unknown	6	14.403	2431626	67644	49.125	30.412	N/A	3719	N/A	1.548	

HPLC spectrum of rac-**43**

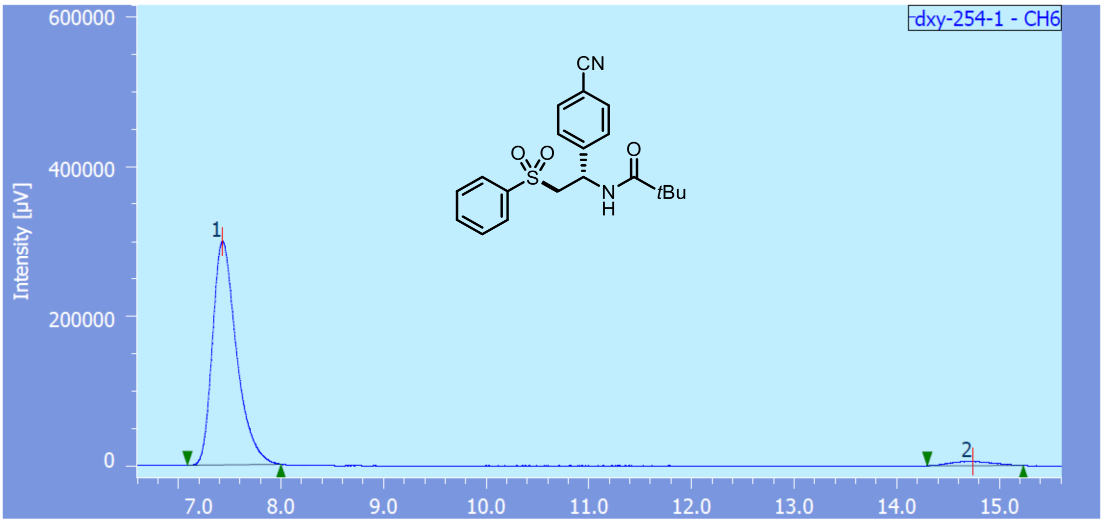

#	Peak Name	CH	tR [min]	Area [µV·sec]	Height [µV]	Area%	Height%	Quantity	NTP	Resolution	Symmetry Factor	Warning
1	Unknown	6	7.430	4858864	298245	96.733	98.159	N/A	5157	11.988	1.410	
2	Unknown	6	14.737	164091	5592	3.267	1.841	N/A	5317	N/A	1.033	

HPLC spectrum of **43**

Tert-butyl (*S*)-(2-(phenylsulfonyl)-1-(*p*-tolyl)ethyl)carbamate (44)

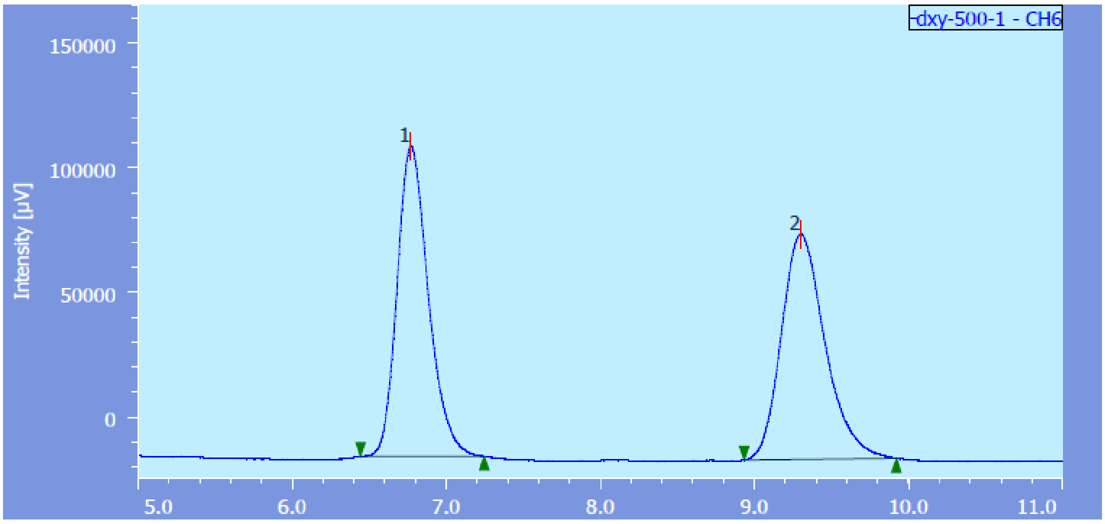

#	Peak Name	CH	tR [min]	Area [µV·sec]	Height [µV]	Area%	Height%	Quantity	NTP	Resolution	Symmetry Factor	Warning
1	Unknown	6	6.770	1756960	124545	49.698	57.982	N/A	5538	5.827	1.257	
2	Unknown	6	9.300	1778331	90255	50.302	42.018	N/A	5389	N/A	1.263	

HPLC spectrum of rac-**44**

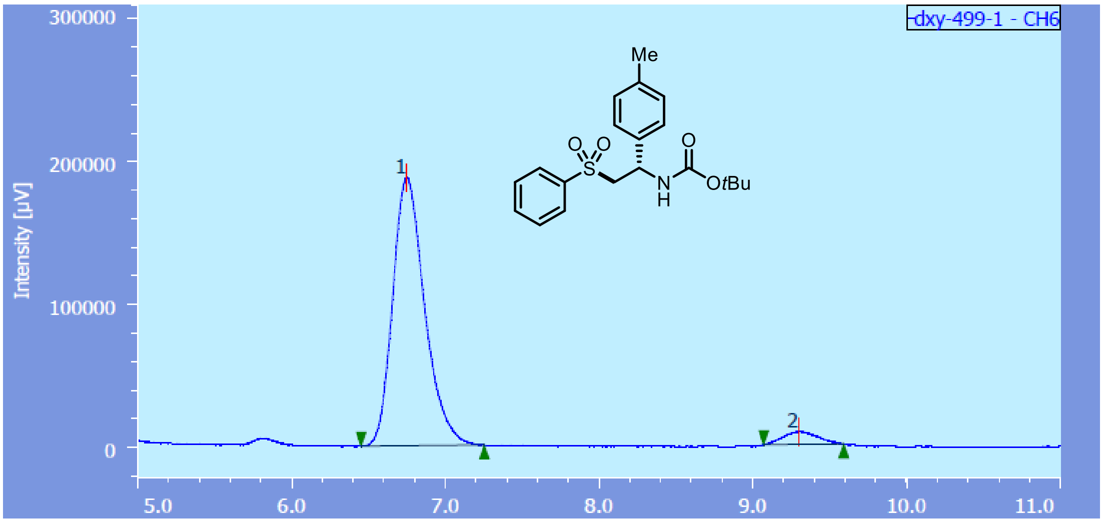

#	Peak Name	CH	tR [min]	Area [µV·sec]	Height [µV]	Area%	Height%	Quantity	NTP	Resolution	Symmetry Factor	Warning
1	Unknown	6	6.750	2703854	187317	94.952	95.471	N/A	5301	6.212	1.283	
2	Unknown	6	9.297	143762	8886	5.048	4.529	N/A	6791	N/A	1.148	

HPLC spectrum of **44**

Tert-butyl (*S*)-(1-(3-methoxyphenyl)-2-(phenylsulfonyl)ethyl)carbamate (45)

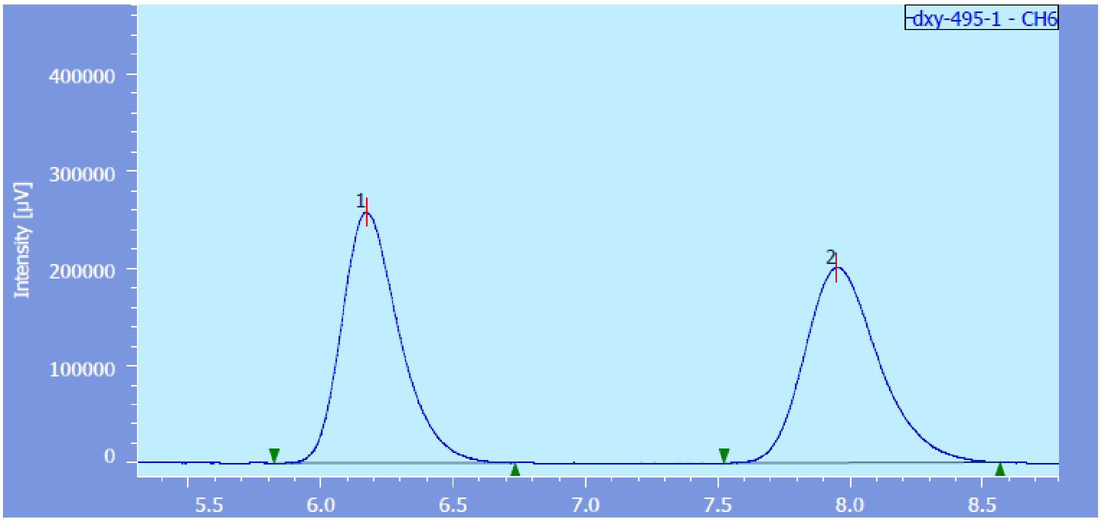

#	Peak Name	CH	tR [min]	Area [µV·sec]	Height [µV]	Area%	Height%	Quantity	NTP	Resolution	Symmetry Factor	Warning
1	Unknown	6	6.173	3899676	257849	49.834	56.254	N/A	4025	3.979	1.300	
2	Unknown	6	7.950	3925658	200518	50.166	43.746	N/A	3948	N/A	1.264	

HPLC spectrum of rac-**45**

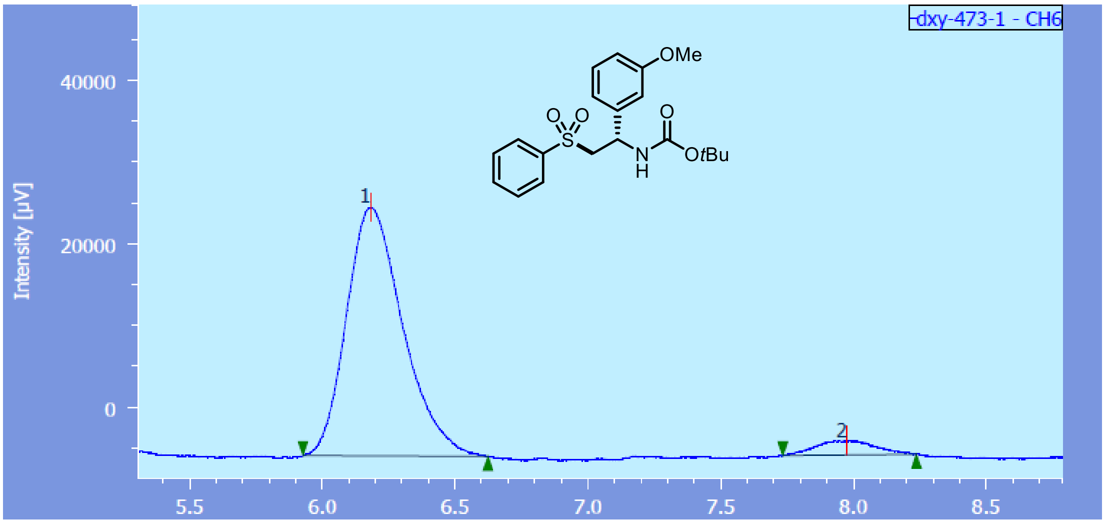

#	Peak Name	CH	tR [min]	Area [µV·sec]	Height [µV]	Area%	Height%	Quantity	NTP	Resolution	Symmetry Factor	Warning
1	Unknown	6	6.183	449100	30359	94.077	94.625	N/A	4161	4.328	1.290	
2	Unknown	6	7.977	28277	1724	5.923	5.375	N/A	5085	N/A	1.015	

HPLC spectrum of **45**

(*S*)-*N*-(1-(4-Cyanophenyl)-2-tosylethyl)-4-methoxybenzamide (46).

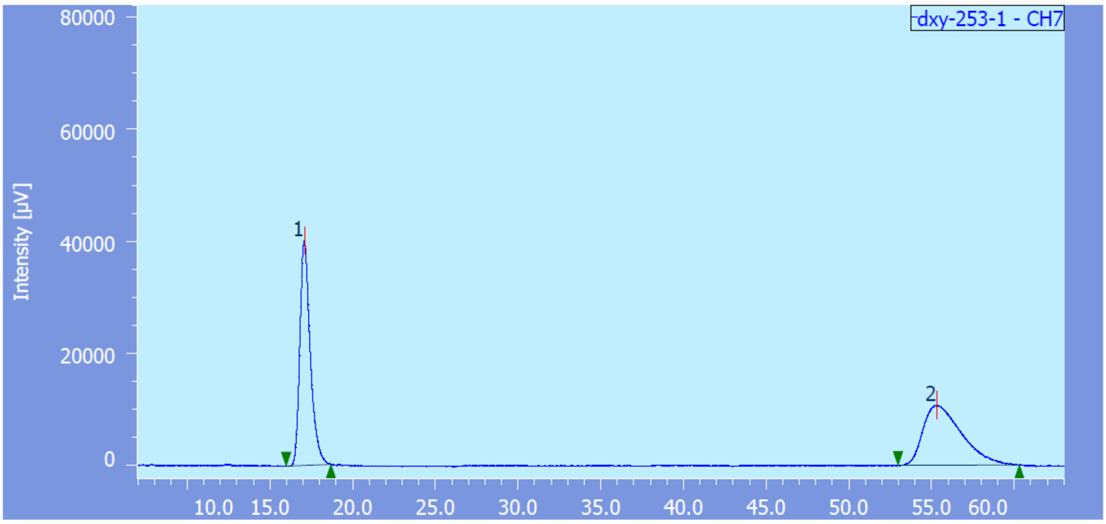

#	Peak Name	CH	tR [min]	Area [µV·sec]	Height [µV]	Area%	Height%	Quantity	NTP	Resolution	Symmetry Factor	Warning
1	Unknown	7	17.070	1747243	40025	50.212	78.911	N/A	3859	14.272	1.376	
2	Unknown	7	55.297	1732487	10696	49.788	21.089	N/A	2681	N/A	1.654	

HPLC spectrum of rac-**46**

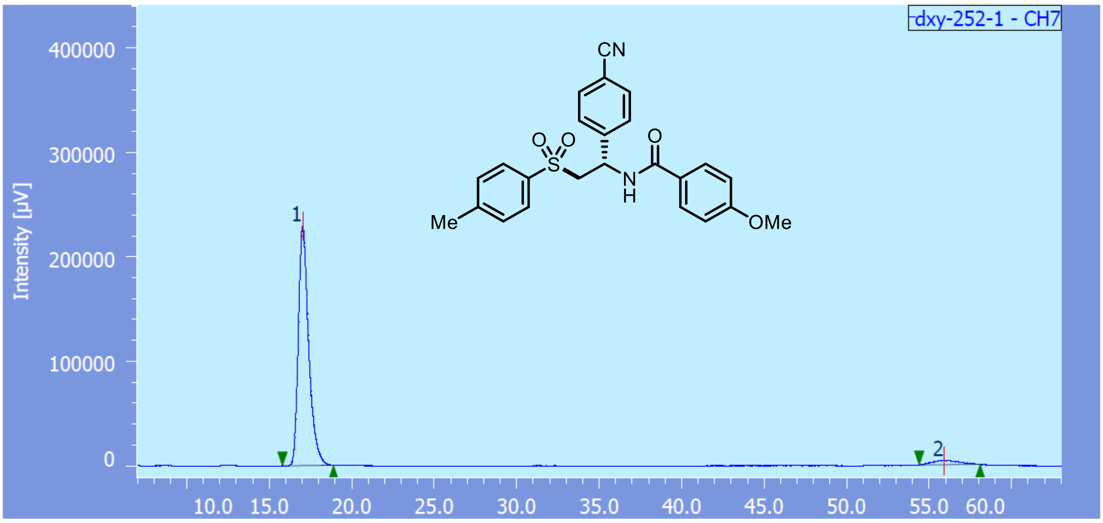

#	Peak Name	CH	tR [min]	Area [µV·sec]	Height [µV]	Area%	Height%	Quantity	NTP	Resolution	Symmetry Factor	Warning
1	Unknown	7	17.020	10000044	229187	95.583	98.338	N/A	3861	17.374	1.416	
2	Unknown	7	55.920	462157	3873	4.417	1.662	N/A	4343	N/A	1.181	

HPLC spectrum of **46**

(*S*)-*N*-(2-((4-Chlorophenyl)sulfonyl)-1-(4-cyanophenyl)ethyl)-4-methoxybenzamide (47).

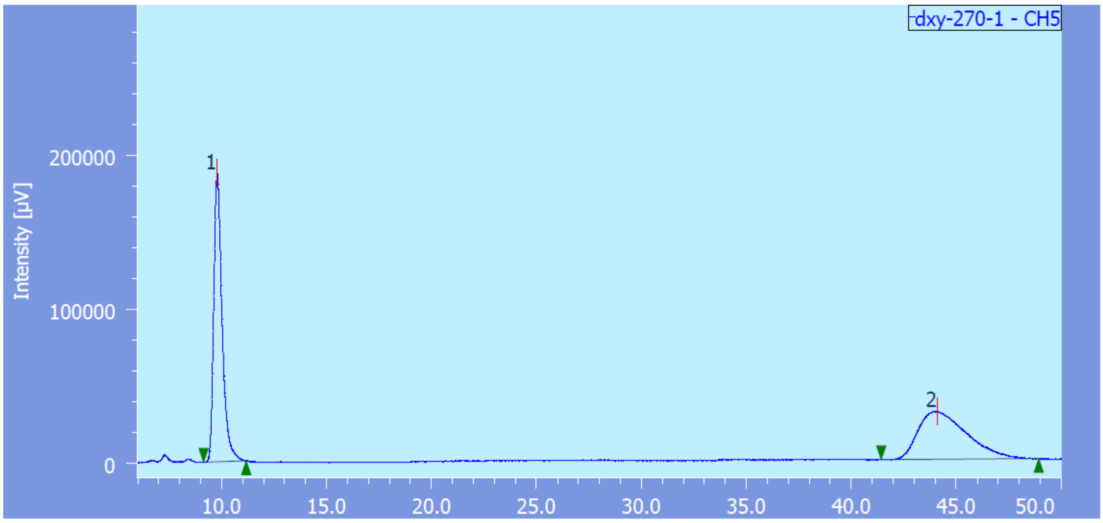

#	Peak Name	CH	tR [min]	Area [µV·sec]	Height [µV]	Area%	Height%	Quantity	NTP	Resolution	Symmetry Factor	Warning
1	Unknown	5	9.790	5204205	187348	50.959	85.776	N/A	3240	13.672	1.442	
2	Unknown	5	44.090	5008387	31067	49.041	14.224	N/A	1649	N/A	1.544	

HPLC spectrum of rac-**47**

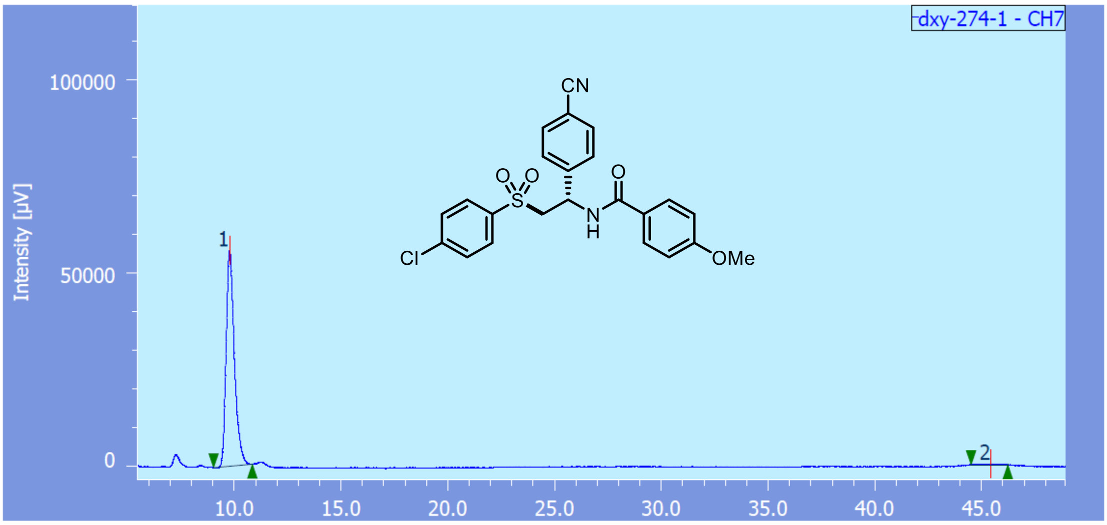

#	Peak Name	CH	tR [min]	Area [µV·sec]	Height [µV]	Area%	Height%	Quantity	NTP	Resolution	Symmetry Factor	Warning
1	Unknown	7	9.783	1497837	55744	99.249	99.609	N/A	3309	24.672	1.356	
2	Unknown	7	45.443	11329	219	0.751	0.391	N/A	6715	N/A	0.918	

HPLC spectrum of **47**

(*S*)-*N*-(2-((4-Acetamidophenyl)sulfonyl)-1-(4-cyanophenyl)ethyl)-4-methoxybenzamide (48).

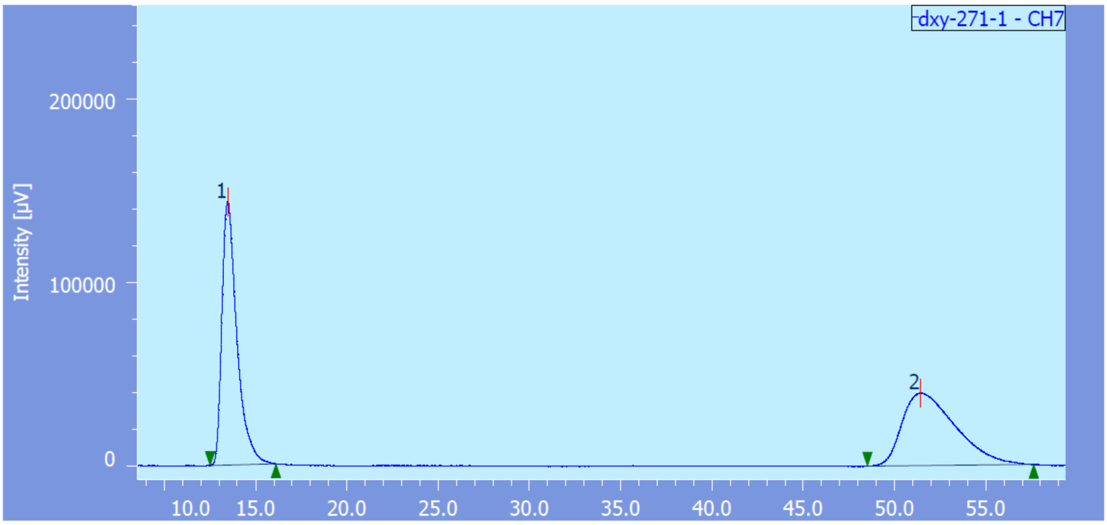

#	Peak Name	CH	tR [min]	Area [μV·sec]	Height [μV]	Area%	Height%	Quantity	NTP	Resolution	Symmetry Factor	Warning
1	Unknown	7	13.460	8178894	143384	50.404	78.437	N/A	1443	11.144	1.676	
2	Unknown	7	51.413	8047889	39418	49.596	21.563	N/A	1444	N/A	1.669	

HPLC spectrum of rac-**48**

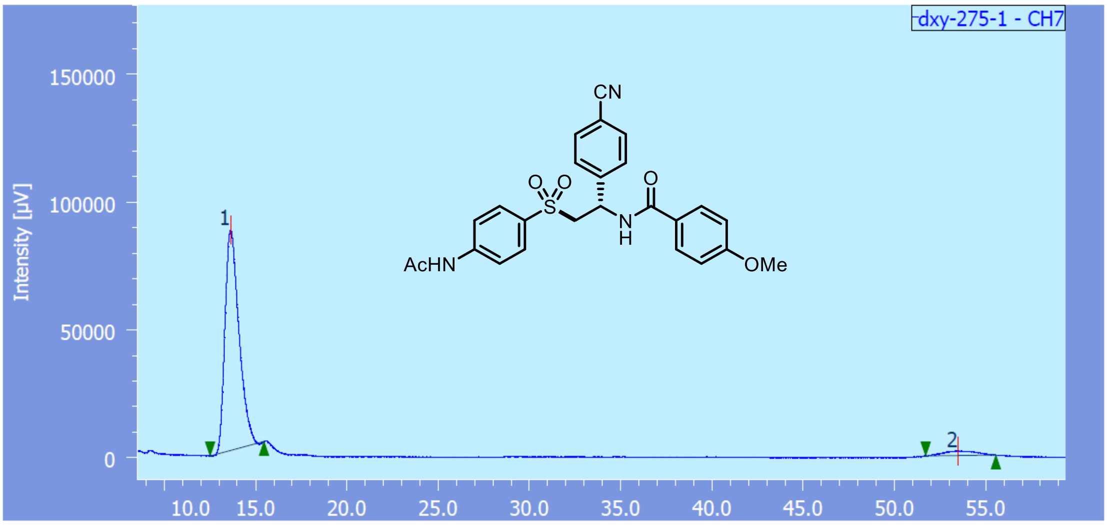

#	Peak Name	CH	tR [min]	Area [μV·sec]	Height [μV]	Area%	Height%	Quantity	NTP	Resolution	Symmetry Factor	Warning
1	Unknown	7	13.633	4589657	86269	95.167	97.988	N/A	1539	15.063	1.424	
2	Unknown	7	53.480	233072	1771	4.833	2.012	N/A	2986	N/A	1.117	

HPLC spectrum of **48**

Methyl (*S*)-3-((2-(4-cyanophenyl)-2-(4-methoxybenzamido)ethyl)sulfonyl)propanoate (49)

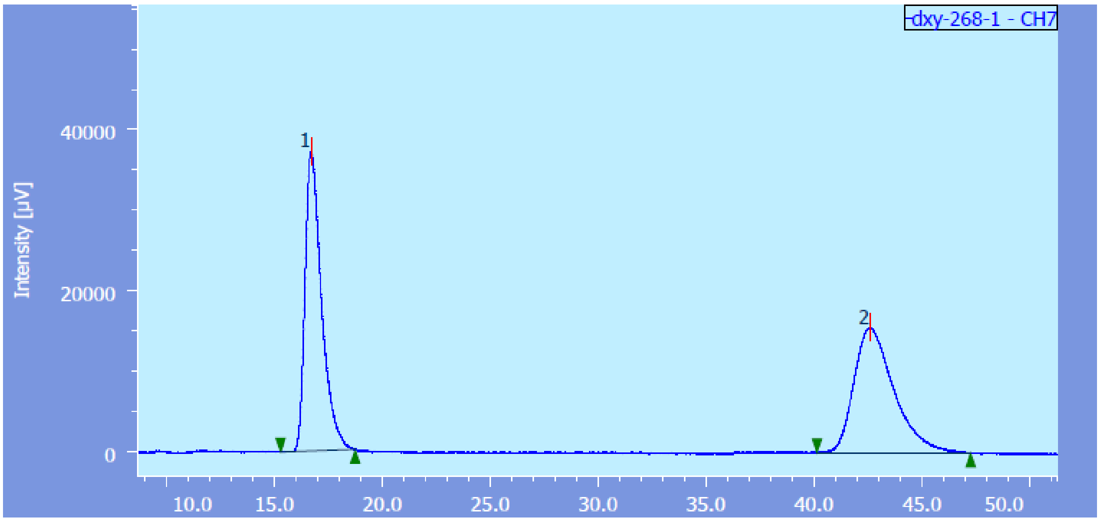

#	Peak Name	CH	tR [min]	Area [μV·sec]	Height [μV]	Area%	Height%	Quantity	NTP	Resolution	Symmetry Factor	Warning
1	Unknown	7	16.710	1977554	37159	49.681	70.546	N/A	2492	11.232	1.627	
2	Unknown	7	42.567	2002982	15515	50.319	29.454	N/A	2699	N/A	1.477	

HPLC spectrum of rac-**49**

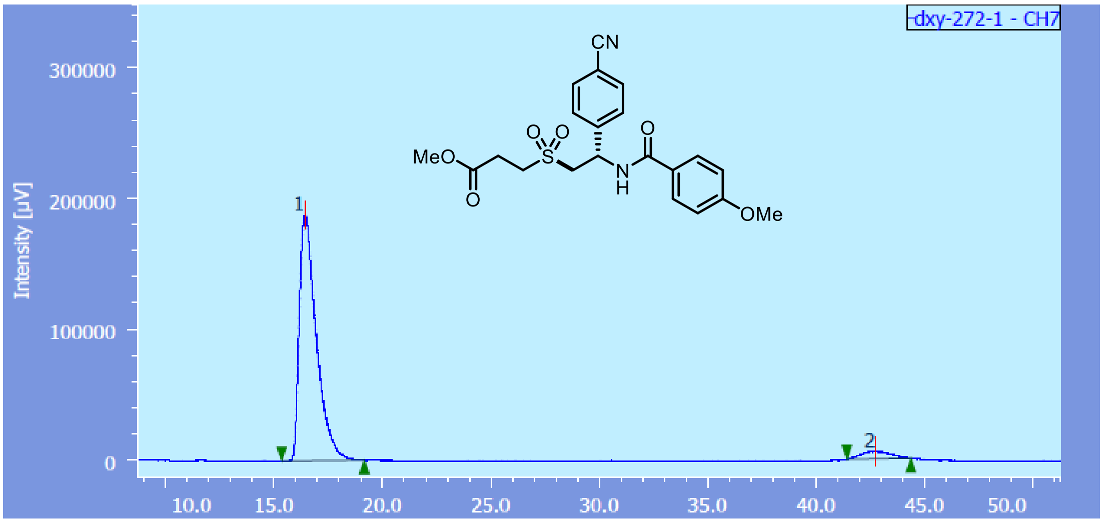

#	Peak Name	CH	tR [min]	Area [μV·sec]	Height [μV]	Area%	Height%	Quantity	NTP	Resolution	Symmetry Factor	Warning
1	Unknown	7	16.430	10207857	187724	94.844	96.990	N/A	2292	12.984	1.893	
2	Unknown	7	42.747	554893	5826	5.156	3.010	N/A	4034	N/A	1.105	

HPLC spectrum of **49**

Methyl (*S*)-3-((2-(4-methoxybenzamido)-2-(4-methoxyphenyl)ethyl)sulfonyl)propanoate (50)

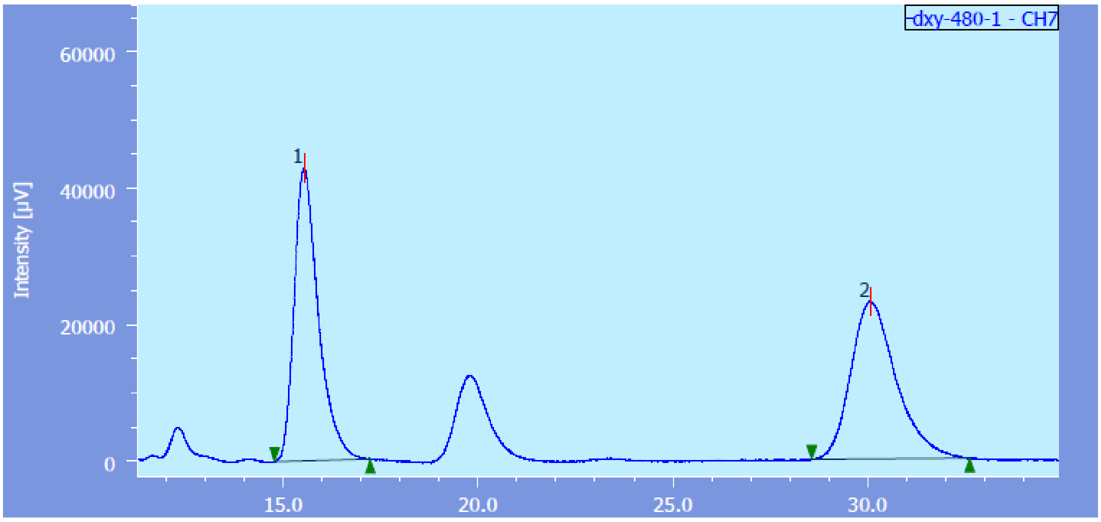

#	Peak Name	CH	tR [min]	Area [μV·sec]	Height [μV]	Area%	Height%	Quantity	NTP	Resolution	Symmetry Factor	Warning
1	Unknown	7	15.533	1851301	42879	49.726	64.987	N/A	3281	9.280	1.466	
2	Unknown	7	30.073	1871699	23101	50.274	35.013	N/A	3419	N/A	1.324	

HPLC spectrum of rac-**50**

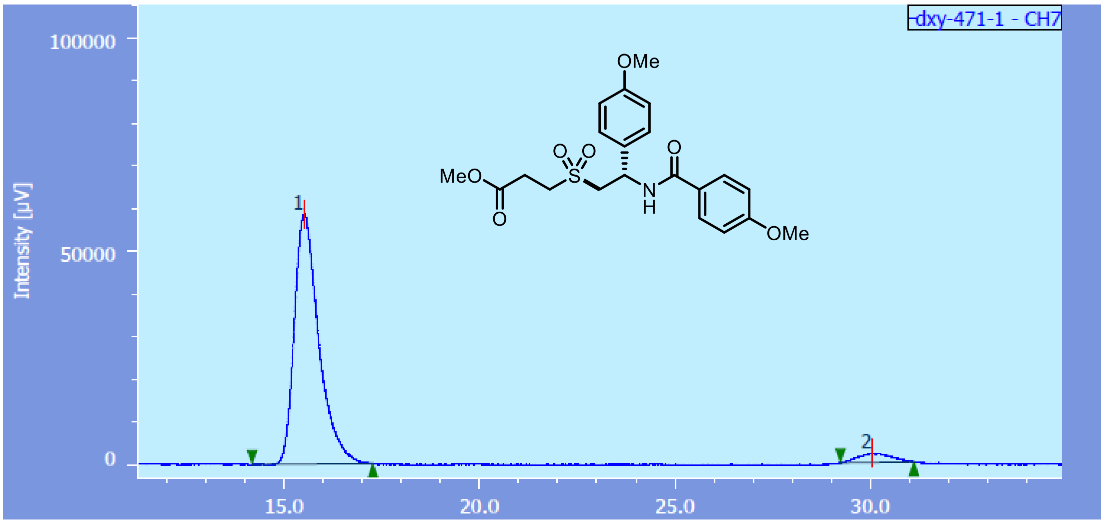

#	Peak Name	CH	tR [min]	Area [μV·sec]	Height [μV]	Area%	Height%	Quantity	NTP	Resolution	Symmetry Factor	Warning
1	Unknown	7	15.520	2541225	58690	95.336	96.500	N/A	3283	10.507	1.468	
2	Unknown	7	30.040	124324	2129	4.664	3.500	N/A	5068	N/A	1.166	

HPLC spectrum of **50**

Methyl (*S*)-3-((2-(4-methoxybenzamido)-2-(3-methoxyphenyl)ethyl)sulfonyl)propanoate (51)

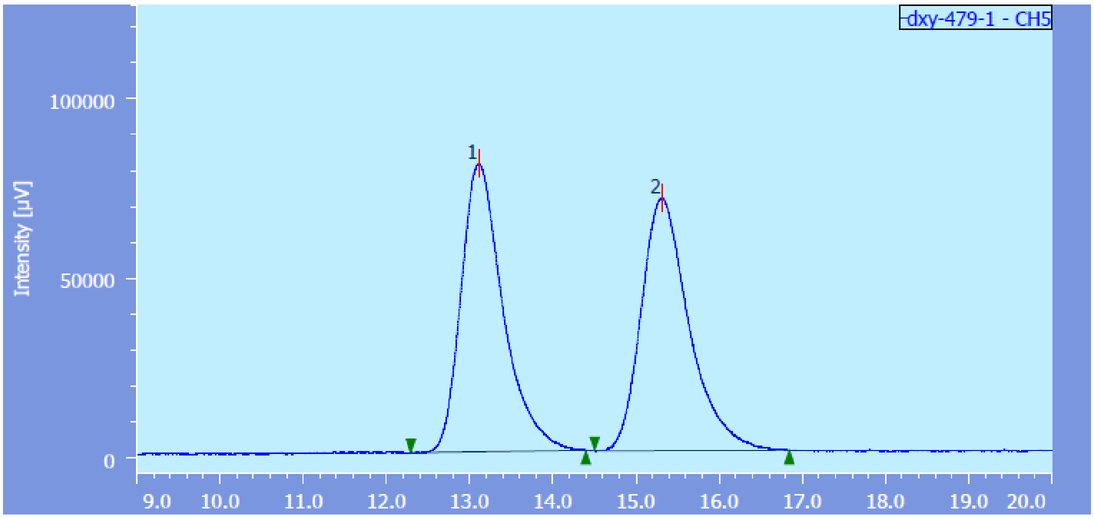

#	Peak Name	CH	tR [min]	Area [µV·sec]	Height [µV]	Area%	Height%	Quantity	NTP	Resolution	Symmetry Factor	Warning
1	Unknown	5	13.107	2814814	80222	49.940	53.288	N/A	3534	2.339	1.419	
2	Unknown	5	15.310	2821548	70322	50.060	46.712	N/A	3700	N/A	1.366	

HPLC spectrum of rac-**51**

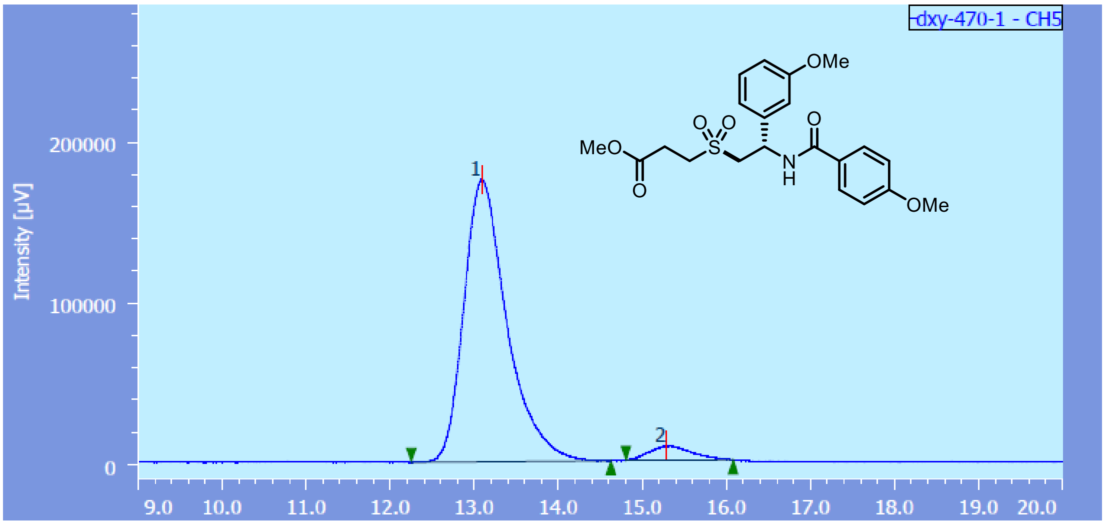

#	Peak Name	CH	tR [min]	Area [µV·sec]	Height [µV]	Area%	Height%	Quantity	NTP	Resolution	Symmetry Factor	Warning
1	Unknown	5	13.090	6239132	174908	95.228	95.172	N/A	3455	2.415	1.419	
2	Unknown	5	15.283	312620	8873	4.772	4.828	N/A	4316	N/A	1.354	

HPLC spectrum of **51**

(*S*)-4-(1-Benzamido-2-(phenylsulfonyl)ethyl)phenyl 4-(*N*,*N*-dipropylsulfamoyl)benzoate (52).

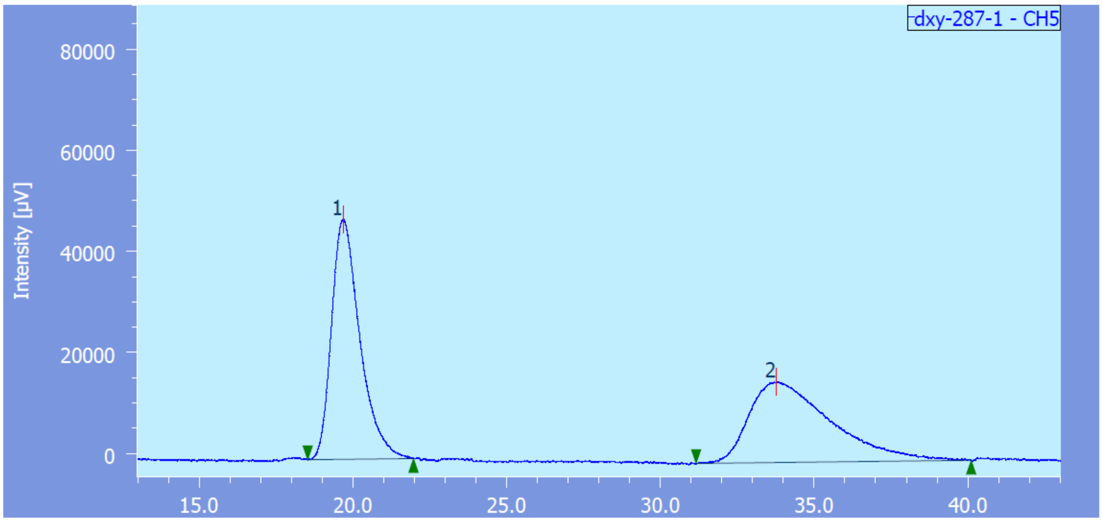

#	Peak Name	CH	tR [min]	Area [μV·sec]	Height [μV]	Area%	Height%	Quantity	NTP	Resolution	Symmetry Factor	Warning
1	Unknown	5	19.687	3046535	47444	50.742	74.822	N/A	2347	4.423	1.435	
2	Unknown	5	33.777	2957438	15965	49.258	25.178	N/A	805	N/A	1.818	

HPLC spectrum of rac-**52**

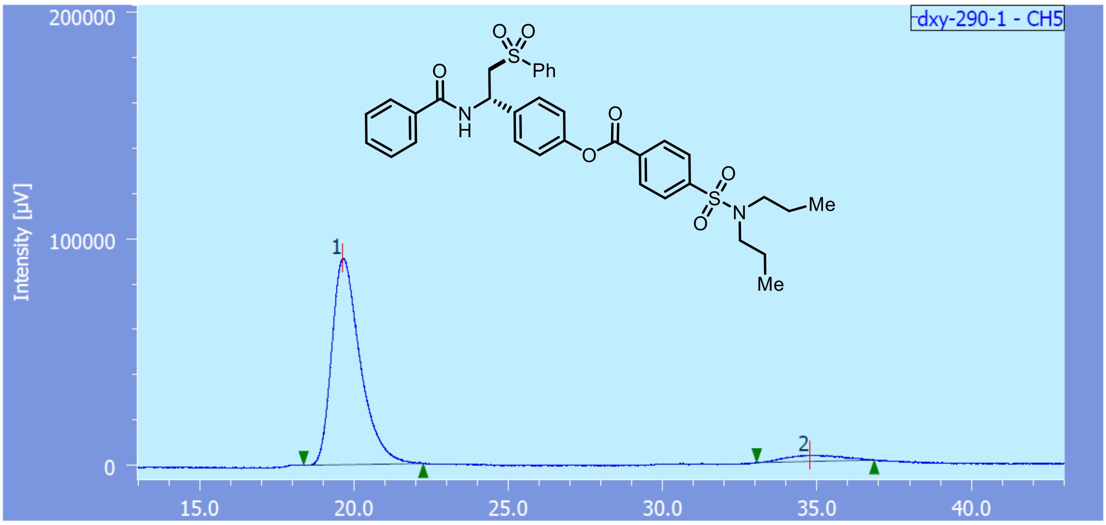

#	Peak Name	CH	tR [min]	Area [μV·sec]	Height [μV]	Area%	Height%	Quantity	NTP	Resolution	Symmetry Factor	Warning
1	Unknown	5	19.643	5834829	91147	94.475	96.967	N/A	2357	5.996	1.485	
2	Unknown	5	34.753	341257	2851	5.525	3.033	N/A	1637	N/A	1.123	

HPLC spectrum of **52**

4-((*S*)-1-Benzamido-2-(phenylsulfonyl)ethyl)phenyl (*3S,5S,7S*)-adamantane-1-carboxylate (53).

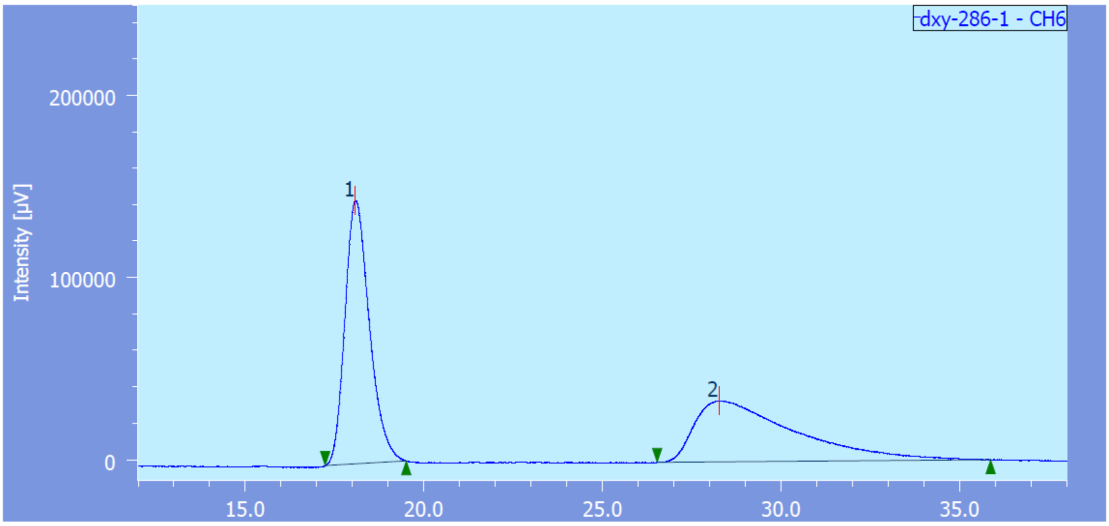

#	Peak Name	CH	tR [min]	Area [µV·sec]	Height [µV]	Area%	Height%	Quantity	NTP	Resolution	Symmetry Factor	Warning
1	Unknown	6	18.087	6888172	144422	51.382	81.200	N/A	3423	3.340	1.287	
2	Unknown	6	28.253	6517563	33437	48.618	18.800	N/A	539	N/A	2.766	

HPLC spectrum of rac-**53**

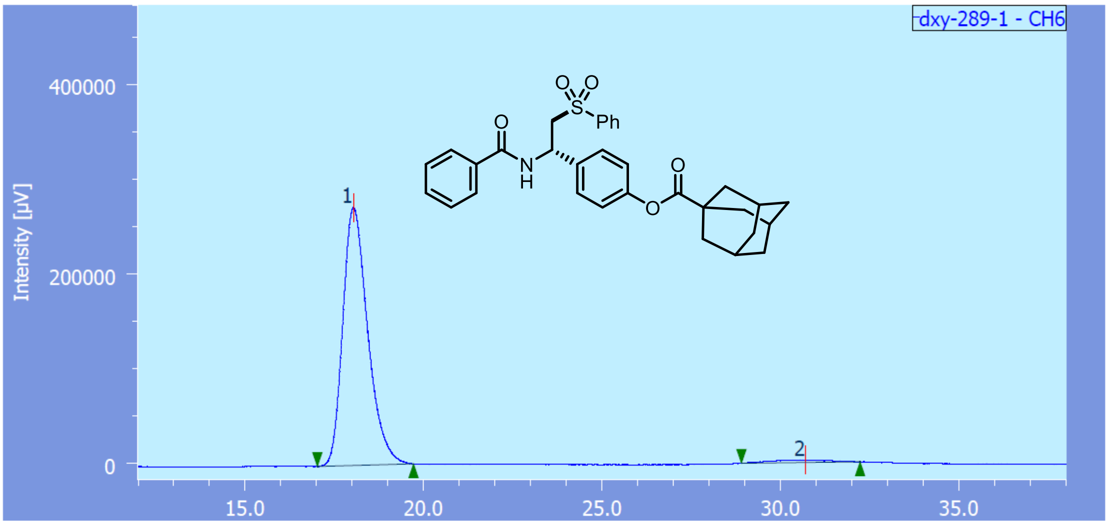

#	Peak Name	CH	tR [min]	Area [µV·sec]	Height [µV]	Area%	Height%	Quantity	NTP	Resolution	Symmetry Factor	Warning
1	Unknown	6	18.033	13200508	271982	97.714	99.072	N/A	3320	5.319	1.329	
2	Unknown	6	30.707	308858	2547	2.286	0.928	N/A	1213	N/A	0.913	

HPLC spectrum of **53**

(*3aR,5R,6S,6aR*)-5-((*S*)-2,2-Dimethyl-1,3-dioxolan-4-yl)-2,2-dimethyltetrahydrofuro[2,3-d][1,3]dioxol-6-yl 4-((*S*)-1-benzamido-2-(phenylsulfonyl)ethyl)benzoate (54).

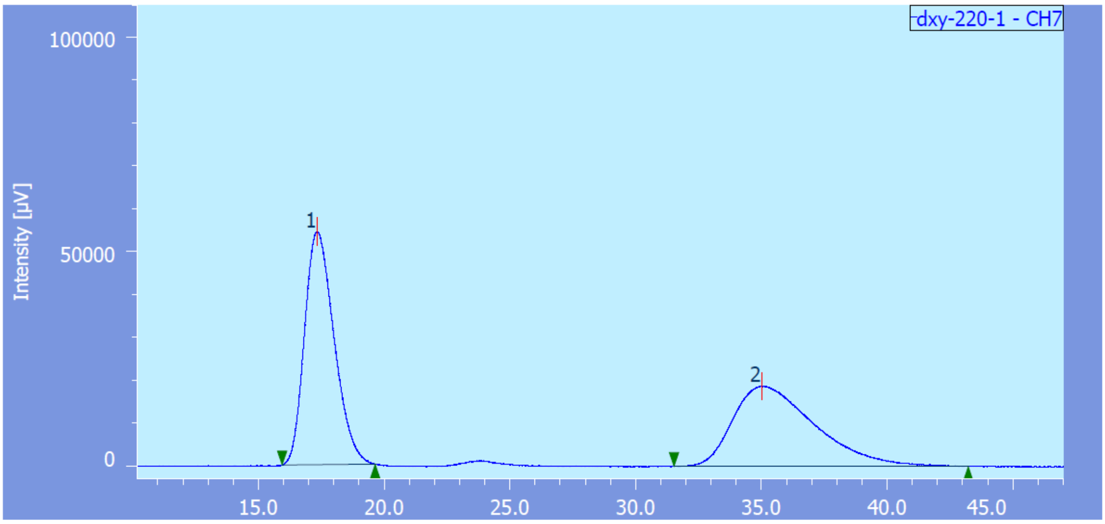

#	Peak Name	CH	tR [min]	Area [µV·sec]	Height [µV]	Area%	Height%	Quantity	NTP	Resolution	Symmetry Factor	Warning
1	Unknown	7	17.330	4438129	54283	51.198	74.403	N/A	1024	4.391	1.295	
2	Unknown	7	35.000	4230400	18676	48.802	25.597	N/A	562	N/A	1.634	

HPLC spectrum of rac-**54**

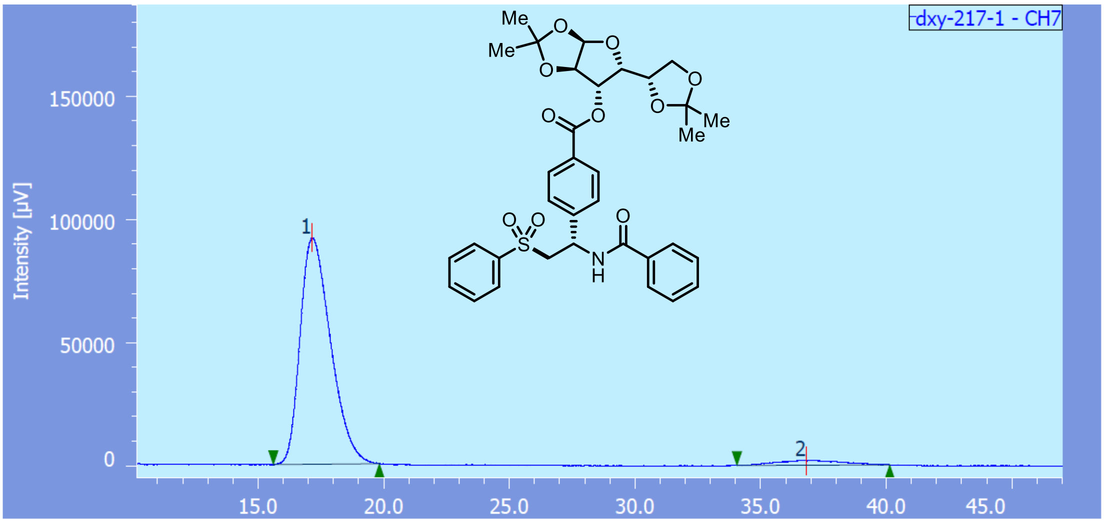

#	Peak Name	CH	tR [min]	Area [µV·sec]	Height [µV]	Area%	Height%	Quantity	NTP	Resolution	Symmetry Factor	Warning
1	Unknown	7	17.163	7763949	91949	95.728	98.044	N/A	949	5.134	1.388	
2	Unknown	7	36.827	346457	1835	4.272	1.956	N/A	730	N/A	1.112	

HPLC spectrum of **54**

Methyl (*S*)-3-(4-((*S*)-1-benzamido-2-(phenylsulfonyl)ethyl)phenyl)-2-((tert-butoxycarbonyl)amino)propanoate (55).

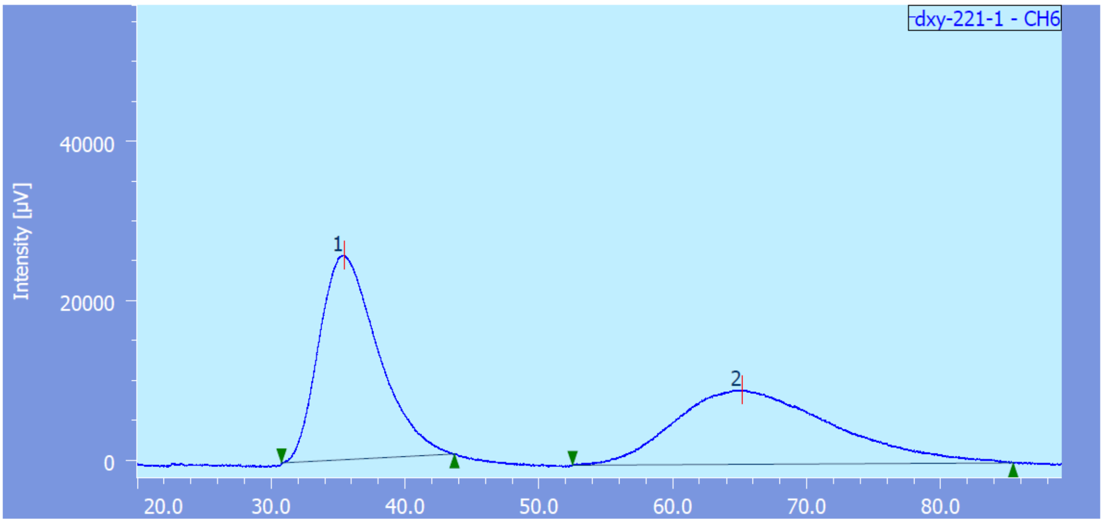

#	Peak Name	CH	tR [min]	Area [µV·sec]	Height [µV]	Area%	Height%	Quantity	NTP	Resolution	Symmetry Factor	Warning
1	Unknown	6	35.427	7857610	25628	50.818	73.328	N/A	310	2.009	1.409	
2	Unknown	6	65.167	7604628	9322	49.182	26.672	N/A	145	N/A	1.377	

HPLC spectrum of rac-**55**

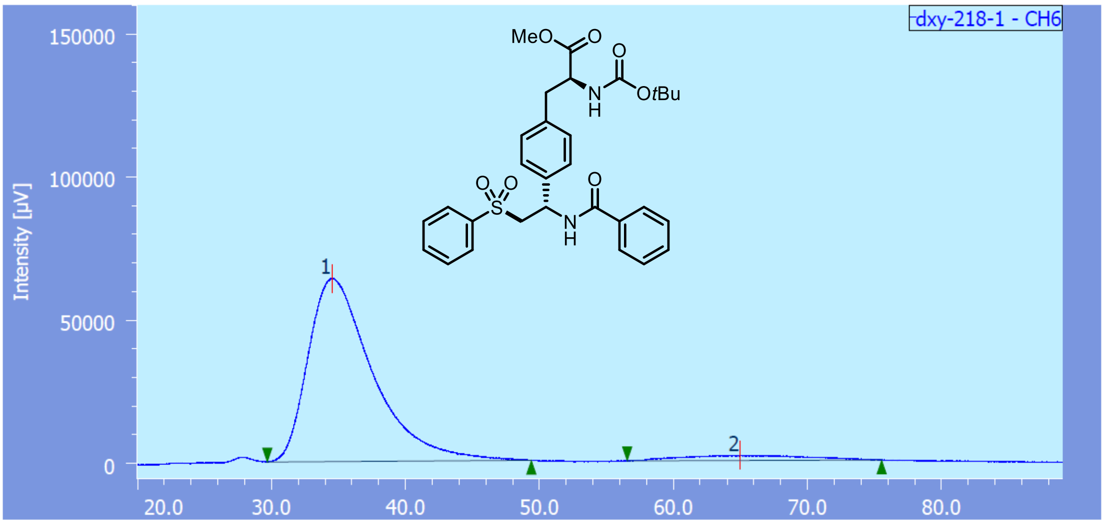

#	Peak Name	CH	tR [min]	Area [µV·sec]	Height [µV]	Area%	Height%	Quantity	NTP	Resolution	Symmetry Factor	Warning
1	Unknown	6	34.493	21476949	63942	94.904	97.326	N/A	273	2.109	1.734	
2	Unknown	6	64.950	1153217	1757	5.096	2.674	N/A	159	N/A	1.120	

HPLC spectrum of **55**

(*S*)-4-(1-Benzamido-2-(phenylsulfonyl)ethyl)phenyl (3-((tert-butoxycarbonyl)amino)propanoyl)glycinate (56)

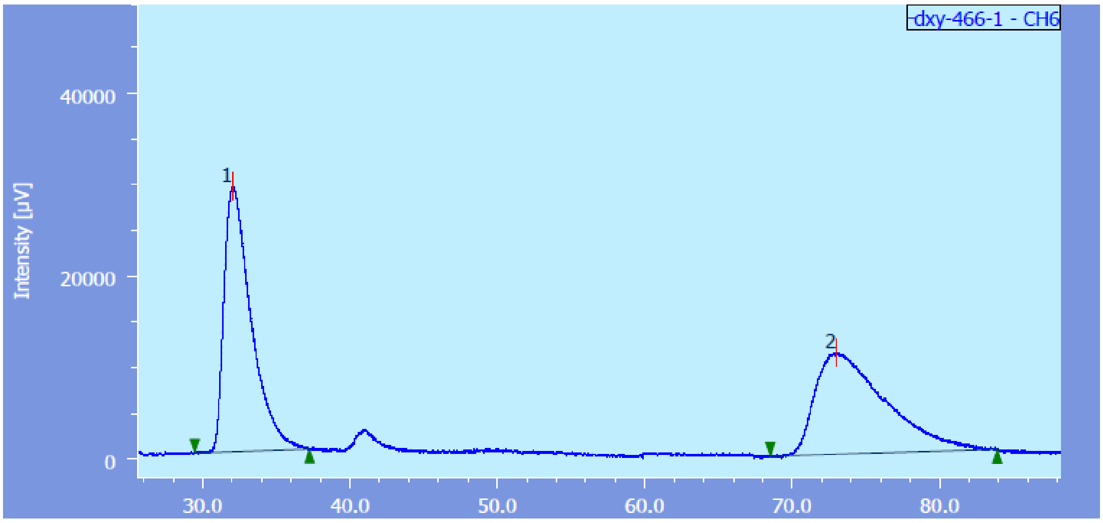

#	Peak Name	CH	tR [min]	Area [µV·sec]	Height [µV]	Area%	Height%	Quantity	NTP	Resolution	Symmetry Factor	Warning
1	Unknown	6	32.060	3686290	28922	50.315	72.393	N/A	1598	6.905	1.941	
2	Unknown	6	72.987	3640080	11029	49.685	27.607	N/A	1132	N/A	2.140	

HPLC spectrum of rac-**56**

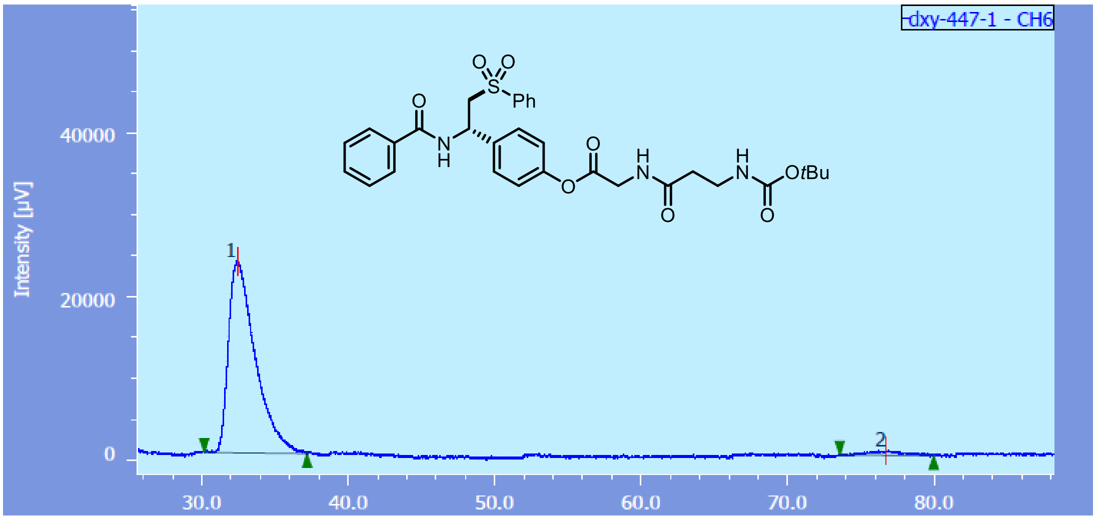

#	Peak Name	CH	tR [min]	Area [µV·sec]	Height [µV]	Area%	Height%	Quantity	NTP	Resolution	Symmetry Factor	Warning
1	Unknown	6	32.407	2972922	23334	96.921	97.563	N/A	1624	7.882	1.971	
2	Unknown	6	76.757	94446	583	3.079	2.437	N/A	1449	N/A	1.010	

HPLC spectrum of **56**

(*3S,5S,8R,9S,10S,13R,17R*)-10,13-Dimethyl-17-((R)-6-methylheptan-2-yl)hexadecahydro-1H-cyclopenta[a]phenanthren-3-yl 4-((*S*)-1-benzamido-2-(phenylsulfonyl)ethyl)benzoate (57).

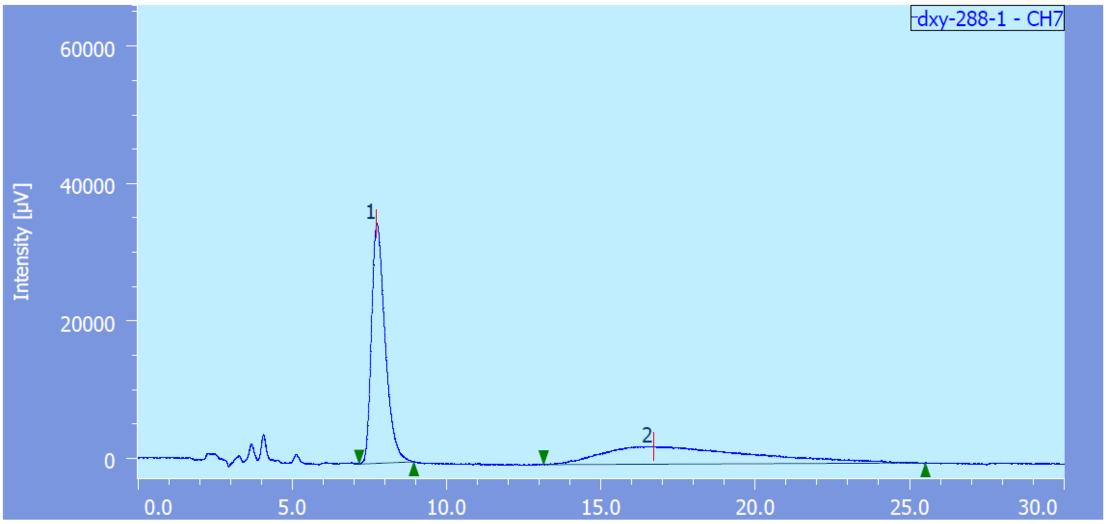

#	Peak Name	CH	tR [min]	Area [µV·sec]	Height [µV]	Area%	Height%	Quantity	NTP	Resolution	Symmetry Factor	Warning
1	Unknown	7	7.740	1073998	34865	55.621	93.097	N/A	1540	1.862	1.495	
2	Unknown	7	16.697	856937	2585	44.379	6.903	N/A	57	N/A	1.747	

HPLC spectrum of rac-**57**

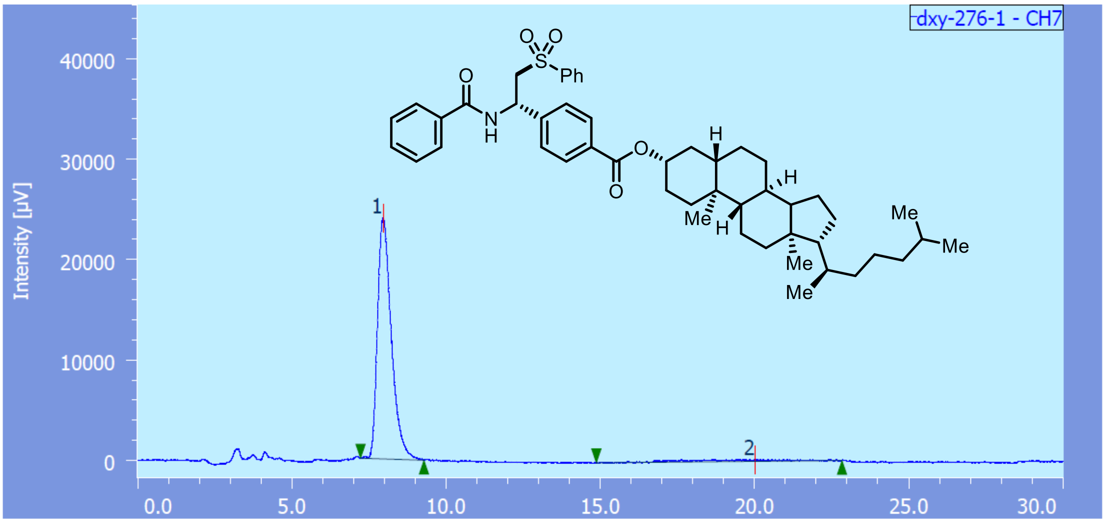

#	Peak Name	CH	tR [min]	Area [µV·sec]	Height [µV]	Area%	Height%	Quantity	NTP	Resolution	Symmetry Factor	Warning
1	Unknown	7	7.960	750673	24005	95.463	99.320	N/A	1592	2.522	1.470	
2	Unknown	7	20.020	35678	164	4.537	0.680	N/A	83	N/A	0.760	

HPLC spectrum of **57**

(*R*)-2,5,7,8-Tetramethyl-2-((*4R,8R*)-4,8,12-trimethyltridecyl)chroman-6-yl 4-((*S*)-1-benzamido-2-(phenylsulfonyl)ethyl)benzoate (58).

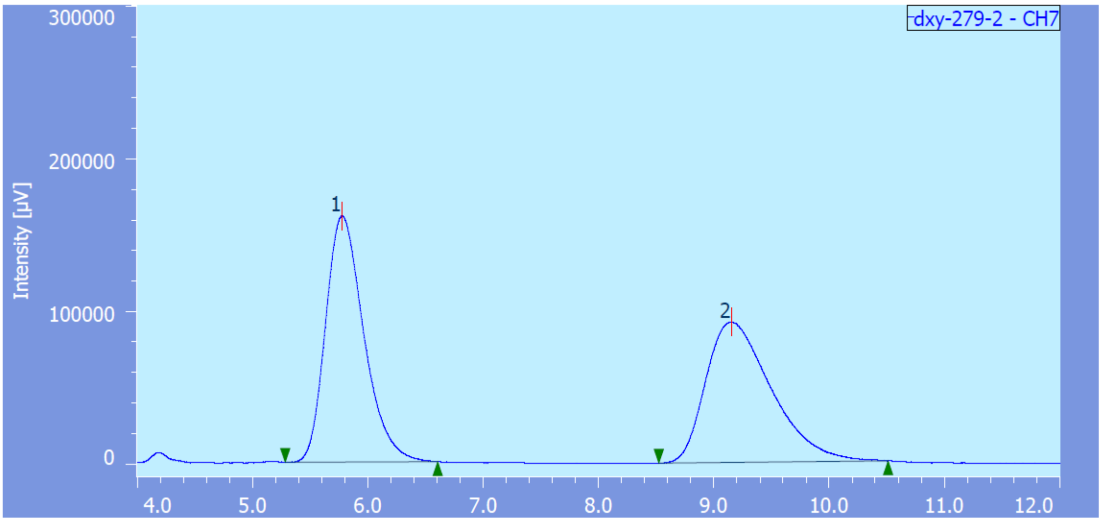

#	Peak Name	CH	tR [min]	Area [µV·sec]	Height [µV]	Area%	Height%	Quantity	NTP	Resolution	Symmetry Factor	Warning
1	Unknown	7	5.777	3723615	161458	50.570	63.686	N/A	1501	4.155	1.316	
2	Unknown	7	9.150	3639741	92065	49.430	36.314	N/A	1258	N/A	1.506	

HPLC spectrum of rac-**58**

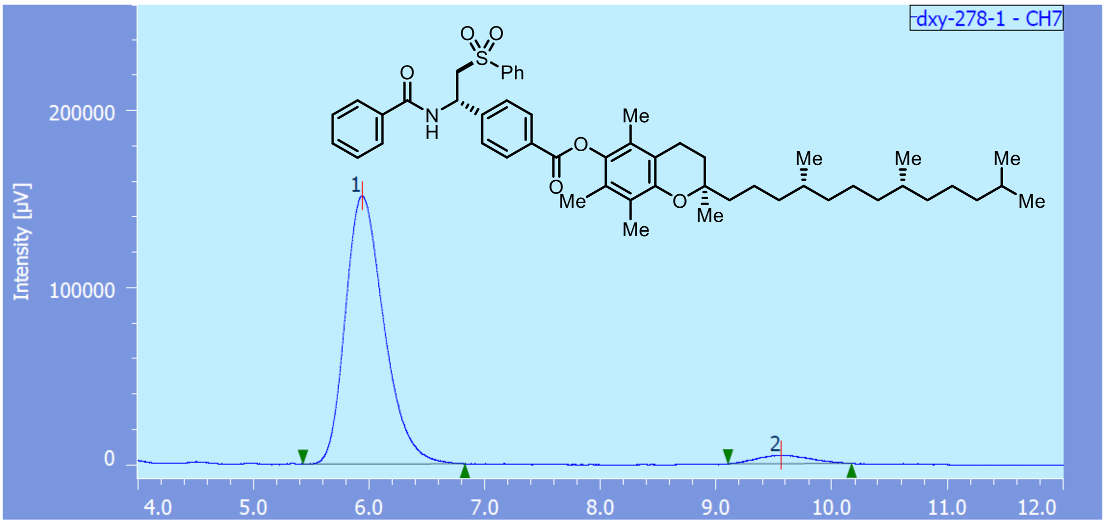

#	Peak Name	CH	tR [min]	Area [µV·sec]	Height [µV]	Area%	Height%	Quantity	NTP	Resolution	Symmetry Factor	Warning
1	Unknown	7	5.940	3503768	151237	95.785	96.996	N/A	1565	4.804	1.306	
2	Unknown	7	9.563	154164	4684	4.215	3.004	N/A	1760	N/A	1.160	

HPLC spectrum of **58**
